# Comparative transcriptome analysis of grapevine in response to copper stress

**DOI:** 10.1038/srep17749

**Published:** 2015-12-17

**Authors:** Xiangpeng Leng, Haifeng Jia, Xin Sun, Lingfei Shangguan, Qian Mu, Baoju Wang, Jinggui Fang

**Affiliations:** 1 College of Horticulture, Nanjing Agricultural University, Tongwei Road 6, Nanjing 210095, PR. China

## Abstract

Grapevine is one of the most economically important and widely cultivated fruit crop worldwide. With the industrialization and the popular application of cupric fungicides in grape industry, copper stress and copper pollution are also the factors affecting grape production and berry and wine quality. Here, 3,843 transcripts were significantly differently expressed genes in response to Cu stress by RNA-seq, which included 1,892 up-regulated and 1,951 down-regulated transcripts. During this study we found many known and novel Cu-induced and -repressed genes. Biological analysis of grape samples were indicated that exogenous Cu can influence chlorophylls metabolism and photosynthetic activities of grapevine. Most ROS detoxification systems, including antioxidant enzyme, stress-related proteins and secondary metabolites were strongly induced. Concomitantly, abscisic acid functioned as a negative regulator in Cu stress, in opposite action to ethylene, auxin, jasmonic acid, and brassinolide. This study also identified a set of Cu stress specifically activated genes coding copper transporter, P_1B_-type ATPase, multidrug transporters. Overall, this work was carried out to gain insights into the copper-regulated and stress-responsive mechanisms in grapevine at transcriptome level. This research can also provide some genetic information that can help us in better vinery management and breeding Cu-resistant grape cultivars.

Copper (Cu) is an important micronutrient essential for plant growth and plays a key role in many physiological processes, including photosynthetic and respiratory electron-transport chains, C and N metabolism ratio, hormone perception, cell wall metabolism and oxidative stress protection^1^,^2^,^3^. Nevertheless, it can easily lead to poisoning when their concentration rises to supra-optimal values not only to plants but also to animals and humans^4^,^5^. Cu phytotoxicity may result from alterations of numerous physiological processes caused at cellular/molecular level by inactivating enzymes, blocking functional groups of metabolically important molecules, displacing or substituting for essential elements and disrupting membrane integrity^6^. A rather common consequence of Cu poisoning causes the rapid and excessive accumulation of reactive oxygen species (ROS) due to interference with photosynthetic and respiratory electron transport activities, especially that of chloroplast membranes^7^. Increased levels of ROS exposes cells to oxidative stress leading to lipid peroxidation, biological macromolecule deterioration, membrane dismantling, ion leakage, and DNA-strand cleavage^8^,^9^,^10^. It has been previously reported that plants resort to a series of regulatory and defence network to adapt to the frequently changing availability of Cu. Due to over-use of Cu-based fungicides and bactericides, wastewater irrigation, and unconscionable Cu mining, Cu stress has become one of the serious environmental crises. Thus, great interest has been gained to understand the physiological and molecular mechanisms of Cu tolerance in plants.

Plants have a complex defence mechanism to enhancement the cell antioxidant systems which counteracts oxidative stress by Cu-exposed^11^,^12^. For example, plants possess very efficient enzymatic (superoxide dismutase, SOD; catalase, CAT; peroxidase, POD; glutathione-S-transferase, GST and the ascorbate-glutathione cycle, AsA-GSH) and non-enzymatic (ascorbate, AsA; glutathione, GSH; carotenoids, alkaloids and phenolic compounds) antioxidant defense systems which work in concert to control the cascades of uncontrolled oxidation and protect plant cells from oxidative damage by scavenging of ROS^11^,^13^,^14^,^15^. For example, anthocyanins, a subclass of phenolic compounds, have been shown to be highly effective scavengers of most types of oxidizing molecules. The effects of Cu stresses on plants depend on how a plant controls and speeds up its rate of ROS production and ROS scavenging when it is exposed to Cu stresses. Further, plants also have evolved a sophisticated defence mechanisms to control Cu uptake, accumulation, translocation and detoxification^16^. For example, copper transporter (COPT/CTR) family belong to high affinity Cu^+^ uptake transporters, regulate Cu uptake in root cells^17^,^18^. Eight CTR family members have been identified in grapevine^18^,^19^. The Cu inside the cell is tightly controlled by a variety of specific copper transport proteins and chaperones that deliver Cu to the secretory pathway^3^,^20^. Copper chaperone (CCH) may interact to move Cu from the cytoplasm into-Golgi vesicles^21^.

Grapevine is one of the most economically important and widely cultivated fruit crop worldwide^22^. Due to over-use of Cu-based fungicides and bactericides, wastewater irrigation, and unconscionable Cu mining, Cu stress has become one of the serious environmental crises to limit grape productivity and quality. Thus, investigating the genetic and molecular mechanisms of grapevine Cu avoidance and tolerance is becoming more and more important. With the availability of the complete grapevine genome and high-throughput RNA sequencing technologies (RNA-seq)^23^,^24^,^25^, it is more necessary and possible to perform the study on copper-regulated and stress-responsive gene networks. In recent years, numerous transcriptome datas have been used to characterization a large-scale genes governing the key developmental and metabolic processes in grapevine^26^,^27^. However, so far, the copper-regulated and stress-responsive mechanism in grapevine have not been studied in detail. In this study, we employed RNA-seq technology to present the transcriptome of grapevine leaves responding to Cu and gain insights into the copper-regulated and stress-responsive mechanisms in grapevine. The goals were to (i) construct a grapevine leaf transcriptome; (ii) compare and analyze differentially expressed genes under control and Cu stress conditions; and (iii) gain insight into copper-regulated and stress-responsive mechanism in grapevine. This study will provide a genetic resource and accelerate the progress of cultivating Cu-resistant plants for crop improvement.

## Results

### Global transcriptome analysis and expressed genes responding to Cu stress

To monitor the Cu stress acclimation rather than cell death, the concentration of Cu treatments had to be carefully selected. In preliminary Cu stress experiments, it was found that 100 μM Cu^2+^ was a proper concentration in causing some drastic decrease of several physiological and growth traits, but without causing the grapevine trees dead. In this study, 100 μM of CuSO_4_ solution was used to spray the grapevine trees for the study on their response to Cu^2+^. Twenty-four hours later after spraying, the young third and fourth leaves from the stem apex was harvested. The grapevine control/Cu-treated RNA samples were used for deep sequencing on an Illumina HiSeq 2500 platform. Sequencing control and Cu-treated samples generated 21.2 and 21.9 million reads, respectively. After trimming, 20.2 and 21.1 million clean reads remained, corresponding to 8.26 Gb clean data (Supplementary: Table S1). The dataset of each sample, including control and Cu-treated, was represented by at least 20 million reads, a tag density sufficient for quantitative analysis of gene expression. The sequence reads were aligned to the grapevine reference genome using SOAPaligner/soap2 software (http://soap.genomics.org.cn), allowing at least two base mismatches. From the total reads, 62.5% matched either to a unique (53.00%) or to multiple (9.50%) genomic locations were recorded (Supplementary: Table S1).

Transcriptome sequencing is one efficient technology that can be used to compare gene expression levels in different samples. In this study, a total of 23, 257 (76.12% of 30, 434) transcripts in the grapevine leaf were identified, including 21, 901 in control and 21, 542 in Cu-treated groups, respectively. Among all the expressed transcripts, the expression levels of 19, 414 transcripts showed no significant changes (|log_2_ fold-change (log_2_FC)| < 1) and 3, 843 transcripts were significantly regulated under Cu stress (|log_2_FC)| ≥ 1) and false discovery rate (FDR) < 0.001), including 1, 892 (49.23% of 3,843) up-regulated ones and 1, 951 (50.77% of 3,843) down-regulated (Supplementary: Table S2). Of the transcripts with varied expressions, 22 transcripts were expressed only in the control leaf, 91 transcripts expressed only in the Cu-treated leaf (Supplementary: Table S3), and 3,730 transcripts were expressed in both libraries, which indicated that Cu could activate or or repress quite a number of unique transcripts.

### Gene ontology (GO) and KEGG analysis of differentially-expressed genes

In order to determine the biological function of differentially expressed genes between control and Cu stress treatments, gene ontology (GO) based enrichment tests were performed. A total of 1, 631 (42.44% of 3, 843) transcripts were annotated in GO and classified into 43 functional groups, including 23 groups in biological process, 11 in molecular function, and nine in cellular component (Supplementary: Table S4 and Fig. S1). In the category of biological processes, “metabolic process” (GO: 0008152) with 1, 040 transcripts sequenced and “cellular process” (GO: 0009987) with 1, 003 transcripts were predominant. Among the molecular_function classes, the two main groups were “binging” (GO: 0005488, 1, 003 transcripts) and “catalytic activity” (GO: 0003824, 878 transcripts). Among the categories “cell part” (GO: 0044464) and “cell” (GO: 0005623) were most common in cellular component, and both had 1, 239 transcripts.

KEGG annotation results were retrieved from KEGG database based on sequence similarity, and 737 (19.18% of 3, 843) transcripts were assigned to 31 KEGG pathways (Supplementary: Table S5 and Fig. S2). Differentially Cu-expressed genes were found in this study, which appeared to be mainly involved in “Carbohydrate metabolism” (130 transcripts), “Translation” (109 transcripts) and “Amino acid metabolism” (100 transcripts).

### Chlorophylls metabolism and photosynthetic capabilities of grapevine in response to Cu stress

**Figure 1:**
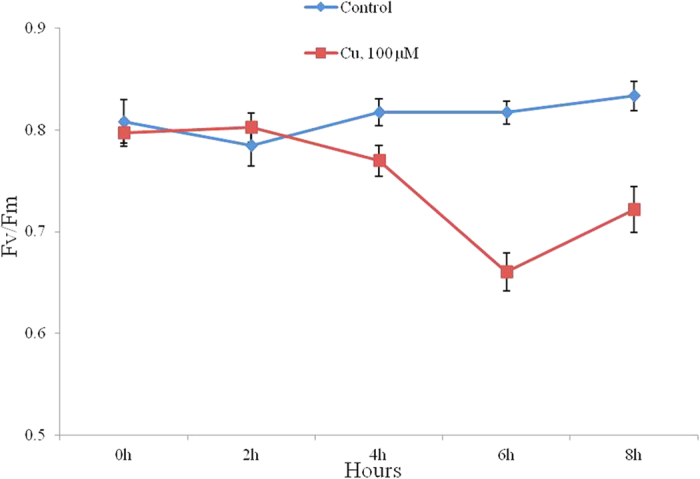
Physiological effects of Cu toxicity in grapevine. Changes in the photosynthetic yield (Fv/Fm) were monitored during 8 h in absence of Cu (diamonds) and in presence of 100 μM (squares) of CuSO_4_.Values represent means of three independent replicates and bars represent the standard error.

Chlorophylls are of important biomolecules, critical in photosynthesis, which allow plants to absorb energy from light. Cu stress had significant effect on chlorophyll (chl) contents and photosynthetic functions in leaves. In this study, we observed that both Chla and Chlb contents had a marked reduction under Cu stress conditions. At the 24 h after Cu treatment, Chla content was decreased from 1.76 ± 0.09 to 1.19 ± 0.06 and Chlb contents from 0.59 ± 0.04 to 0.42 ± 0.03 in the grapevine leaves (Table 1). Chla had a significantly degraded than Chlb under Cu stress. Furthermore, 100 μM Cu^2+^ led to a drastic decrease of photosynthetic activity (Fv/Fm) after 4 hours of treatment (Fig. 1).

**Figure 2:**
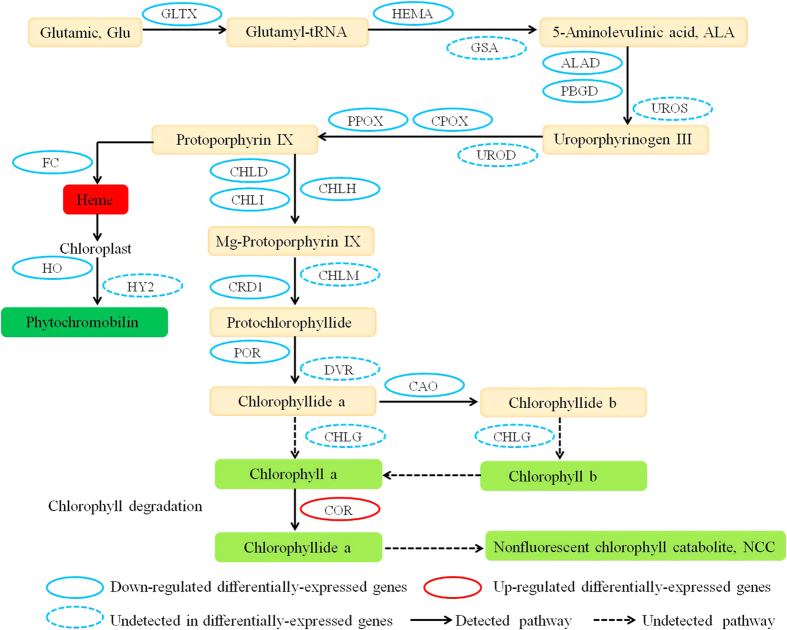
Differential expression analysis of Chlorophyll metabolic pathway in respond to Cu. GLTX, glutamyl-tRNA synthetase; HEMA, glutamyl-tRNA reductase; GSA, Glutamate-1-semialdehyde aminotransferase; ALAD, delta-aminolevulinic acid dehydratase; PBGD, porphobilinogen deaminase; UROS, uroporphyrinogen III synthase; UROD, uroporphyrinogen III decarboxylase; CPOX, coproporphyrinogen III oxidase; PPOX, protoporphyrinogen oxidase; CHLD, magnesium-chelatase D subunit; CHLH, magnesium-chelatase H subunit; CHLI, magnesium-chelatase I subunit; CHLM, Mg-proto IX methyltransferase; CRD1, magnesium-protoporphyrin IX monomethylester [oxidative] cyclase; POR, protochlorophyllide oxidoreductase; DVR, divinyl-protochlorophyllide reductase; CHLG, chlorophyll synthase; CAO, chlorophyllide a oxygenase; COR, chlorophyllase; FC, ferrochelatase; HO, hemeoxygenase; HY2,phytochromobilin synthase.

Chlorophyll metabolic pathway mainly comprised on three phases: (I) chlorophyll a synthesis from glutamate, (II) interconversion of chlorophyll a and chlorophyll b (chlorophyll cycle), and (III) chlorophyll degradation pathway. Of the grapevine transcriptome, 15 transcripts involving in chlorophyll metabolic showed significantly differences in respond to Cu signaling compared with control, including 14 that were down-regulated and one up-regulated. Meanwhile, 13 transcripts (glutamyl-tRNA synthetase, GLTX; glutamyl-tRNA reductase, HEMA; delta-aminolevulinic acid dehydratase, ALAD; porphobilinogen deaminase, PBGD; coproporphyrinogen III oxidase, CPOX; protoporphyrinogen oxidase, PPOX; magnesium-chelatase D subunit, CHLD; magnesium-chelatase H subunit, CHLH; magnesium-chelatase I subunit, CHLI; magnesium-protoporphyrin IX monomethylester [oxidative] cyclase, CRD1; protochlorophyllide oxidoreductase, POR) in chlorophyll a synthesis and one transcript (chlorophyllide a oxygenase, CAO) in chlorophyll cycle were significantly decreased, and single transcript (chlorophyllase, COR) in chlorophyll degradation pathway was significantly increased (Table 2, Fig. 2, Supplementary: Table S6). The expression levels of VIT_08s0040g00390.t01 (909.20 to 308.83 RPKM) and VIT_19s0014g03160.t01 (1190.11 to 431.48 RPKM) showed high abundances in chlorophyll a synthesis pathway (Supplementary: Table S6). The results indicated that exogenous Cu could reduce chlorophyll content because the chlorophyll synthesis was inhibited and degradation pathway was induced by Cu stress. In addition, Cu stress also affected the synthesis of phytochromobilin. The expression of ferrochelatase (VIT_04s0008g00800.t01, |log_2_FC| = 1.02439) and heme oxygenase (VIT_18s0001g11040.t01, |log_2_FC| = 1.66) were also inhibited by Cu stress (Table 2, Fig. 2, Supplementary: Table S6).

In grapevine transcriptome, 30 photosynthesis-related genes sensitive to Cu stress were identified, which were involved in PSII (seven transcripts), PSI (one), cytochrome b6-f complex (three), ATP synthase (five), photosynthetic electron transport chain (four), photosynthesis-antenna proteins (two) and eight other photosynthesis-related transcripts (Table 2, Supplementary: Table S7). Among the seven differentially expressed genes in photosystem II, two psbAs, one psbB and two psbPs were significantly decreased, while psbM and psbW were found to be increased compared with the those in control (Table 2, Supplementary: Table S7). psbW (VIT_07s0141g00570.t01, from 4600.90 to 9688.29 RPKM) had high expression abundance (Supplementary: Table S7). The expression levels of one psaB related to PSI and three transcripts involved in cytochrome b6-f complex (one petA and two petC) showed significant reduction with control group (Table 2, Supplementary: Table S7). Five ATP synthase genes were also significantly down-regulated (Table 2, Supplementary: Table S7). Of the genes involving in photosynthetic electron transport chain, the expression of petH (VIT_18s0001g14450.t01) and petJ (VIT_01s0011g01850.t01) was found as inhabitor, while petE (VIT_18s0001g06580.t01) and petF (VIT_13s0147g00110.t01) showed significant increase compared with the those in control group (Table 2, Supplementary: Table S7). Two high abundant photosynthesis-antenna proteins, LHCA4 (from 3094.39 to 1414.90 RPKM) and LHCA6 (from 3051.00 to 1371.04 RPKM), had also decreased expression. Moreover, eight other photosynthesis-related transcripts involved in thylakoid part, phytochrome binding protein and transcription factor showed decreased changes by Cu signaling pathways (Table 2, Supplementary: Table S7). For example, phytochrome-interacting factor 3 (PIF3), a nuclear-localized basic helix-loop-helix transcription factor, showed significant decrease expression compared with control.

### Reactive oxygen species (ROS) producing and scavenging system in response to Cu stress

Overproduction of reactive oxygen species (ROS) and over-expression of various antioxidative enzymes in the ROS scavenging system could occur during almost in all biotic and abiotic stress conditions^28^. In this study, after 24 h of Cu supply showed remarkable increment in Cu content from 10.51 ± 0.81 to 31.06 ± 1.21 μg/g DW in leaves (Table 1). This data also showed that the foliar Cu did exceed 30 μg/g DW, a critical toxicity concentration of Cu in plant leaves^29^. MDA, a product of lipid peroxidation, often considered an indicator of oxidative stress. 100 μM CuSO_4_ treatments led to a significant increase from 5.55 ± 0.19 to 9.77 ± 0.39 nmol/g of MDA content in the leaves (Table 1). Further, 100 μM CuSO_4_ caused a conspicuously increase of SOD, CAT and POD activity in grapevine leaves, which were of 112.2%, 194.5% and 263.1% increase in comparison to the control plants, respectively. This could confirm the fact that SOD, CAT and POD played important role in ROS scavenging system (Table 1). In transcriptome, many encoding genes involved in proteins synthesis, producing and detoxification ROS mechanisms were regulated under Cu stress. For instance, two NADPH oxidases and five amine oxidases showed unregulated expressions, might be possible to participate in the production of ROS under acute oxidative stress (Table 3). 69 transcripts with differentially-expressed profiles were identified as encoding enzymes in the ROS scavenging system. They were categorized into the Fe superoxide dismutase (Fe-SODs, two transcripts), peroxidase (POD, seven), catalase (CAT, three), glutathione-ascorbate (GSH-AsA) cycle (11), glutathione peroxidase (GPX, one), glutathione S-transferase (GST, 30), and the peroxiredoxin/thioredoxin (Prx/Trx) pathways (10), alternative oxidase (AOX, three) and polyphenol oxidase (PPO, two) (Table 3, Supplementary: Table S8).

**Figure 3:**
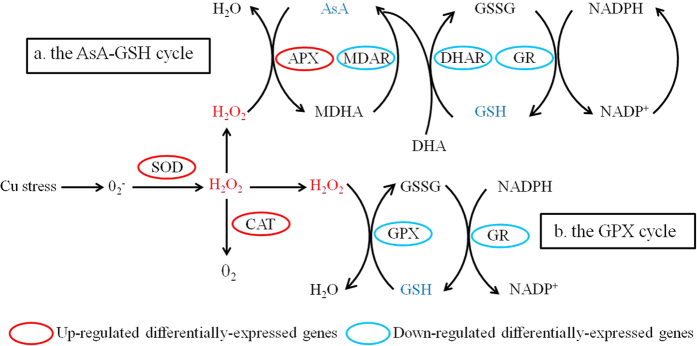
Pathways for reactive oxygen species (ROS) scavenging in plants. (**a**) The ascorbate-glutathione (AsA-GSH) cycle. (**b**) The glutathione peroxidase (GPX) cycle. Superoxide dismutase (SOD) acts as the first line of defense converting O_2_^−^ into H_2_O_2_. Catalase (CAT), ascorbate peroxidases (APX) and glutathione peroxidase (GPX) then detoxify H_2_O_2_. AsA and GSH are antioxidants in blue. Abbreviations: DHA, dehydroascorbate; GSH, glutathione; GSSG, oxidized glutathione; GR, glutathione reductase; MDAR, monodehydroascorbate reductase; DHAR, dehydroascorbate reductase.

**Figure 4:**
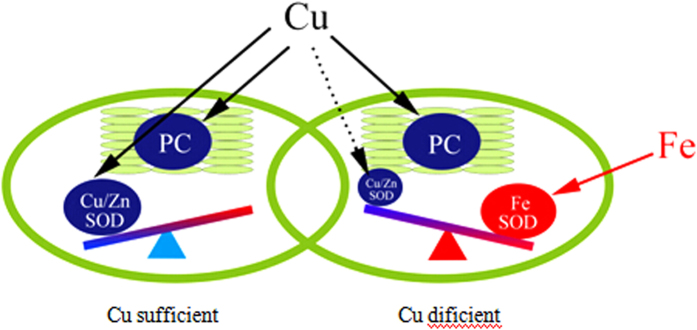
Substitution relation between Cu-Zn superoxide dismutase (Cu-Zn SOD) and Fe superoxide dismutase (Fe SOD) depending on Cu bioavailability in grapevine. Cu-Zn SOD is the predominant SOD within the chloroplast during Cu-replete conditions, whereas it is substituted by Fe-SOD upon Cu limitation. Cu proteins are represented in blue, while Fe proteins are in red. Solid and dotted lines represent, respectively, the main and minor Cu delivery pathways under Cu deficiency. PC, plastocyanin; SOD, superoxide dismutase.

SODs provide the first line of defense mechanism against highly toxic superoxide radicals by rapidly disproportionate O_2_^−^ into oxygen and H_2_O_2_ (Fig. 3). SODs are classified by metal cofactors into three types: copper-zinc (Cu/Zn-SOD), iron (Fe-SOD) and manganese (Mn-SOD), which are localized in different cellular compartments^13^. In our results, five Cu/Zn-SODs showed no changes of expression levels (|log_2_FC| < 1), while two Fe-SODs were remarkably down-regulated (|log_2_FC| > 2). One of the non-variable Cu-Zn SOD genes, VIT_06s0061g00750.t01 (from 764.22 to 915.59 RPKM) had high expression abundance and the two down-regulated Fe-SOD genes had low transcript abundance (Supplementary: Table S8). The results supported the fact that the function of Fe-SOD can be substituted by Cu/Zn-SOD when Cu levels are sufficient (Fig. 4). CAT and POD took part in the metabolism reducing H_2_O_2_ directly to water and oxygen (oxide donors) (Fig. 3). In this study, three CATs and seven PODs were up-regulated by Cu stress except VIT_18s0122g01320.t01 (CAT), suggesting the importance of CATs and PODs in scavenging ROS under Cu stress (Table 3, Supplementary: Table S8). Two up-regulated of CAT genes (VIT_00s0698g00010.t01, from 296.15 to 645.67 RPKM; VIT_04s0044g00020.t01, from 394.80 to 991.17 RPKM) had high abundance and the down-regulated ones had moderately transcript abundant (Supplementary: Table S8). Among the seven identified POD genes, VIT_06s0004g07770.t01 (from 12.52 to 114.99 RPKM) and VIT_18s0072g00160.t01 (from 9.69 to 212.71 RPKM) also had high abundance and others up-regulated genes were expressed at quite a low levels (Supplementary: Table S8). Further, the ascorbate-glutathione (AsA-GSH) cycle, the GPX pathway, the Prx/Trx pathway, the alternative oxidase (AO) and polyphenol oxidase (PPO) were identified, of which also played an essential roles in defense system against ROS and scavenge H_2_O_2_ by Cu stress. Totally, 11 AsA-GSH cycle genes were identified, including four up-regulated genes and seven down-regulated genes (Table 3, Fig. 3, Supplementary: Table S8). 30 Glutathione S-transferase (GSTs, 27 up-regulated, three down-regulated) were identified in the GPX pathway (Table 3, Supplementary: Table S8). Two transcripts, VIT_17s0000g06130.t01 (|log_2_FC| = 7.21) and VIT_08s0040g00920.t01 (|log_2_FC| = 8.70), had significantly induced expression in the Cu-treated sample (Supplementary: Table S8). Furthermore, there were two down-regulated Prxs and eight Trxs (three up-regulated, five down-regulated) in the Prx/Trx pathway (Table 3, Supplementary: Table S8). VIT_19s0027g01930.t01 (|log_2_FC| = 5.40), a down-regulated Prx, was only expression in the control (Supplementary: Table S8). In addition, all three alternative oxidase (AO) were significantly up-regulated in the cyanide-resistant respiration. Two polyphenol oxidase (PPO) were ubiquitous copper-containing enzymes and also had significantly up-regulated compared with control (Table 3, Supplementary: Table S8).

### Heat shock protein (HSP) and pathogenesis-related proteins (PR) in response to Cu stress

Heat shock proteins (HSPs), including HSP100s, HSP90s, HSP70s, HSP60s (cpn60s), and small heat-shock proteins (sHSPs), are stress-responsive proteins that function as molecular chaperones, protecting plants from damage under stress^30^. In the whole transcriptome, 49 transcripts were identified as HSPs in differentially-expressed genes, including one HSP101, three HSP90s, two HSP70s, 18 sHSPs, 20 other HSP transcripts and 5 heat stress transcription factors (Table 4, Supplementary: Table S9). All six high molecular weight HSPs (HMW HSPs) (HSP101, HSP90s and HSP70) were down-regulated (Table 4, Supplementary: Table S9). The down-regulated HSP70 was expressed at high abundance (VIT_17s0000g03310.t01, from 463.09 to 137.75 RPKM), while the others HMW HSPs had low transcript abundances (Supplementary: Table S9). 18 sHSPs transcripts differed in expression levels, in which 16 were up-regulated and two down-regulated. The up-regulated VIT_04s0008g01490.t01 (from33.14 to 364.19 RPKM) showed moderate transcript abundances and the other variable sHSPs were expressed at low abundances (Supplementary: Table S9). Among 20 other HSPs, six transcripts were up-regulated and 14 were down-regulated (Table 4, Supplementary: Table S9). Five transcripts were identified as heat stress transcription factor, including four up-regulated and one down-regulated ones (Supplementary: Table S9).

The Cu as micronutrient plays a multifaceted and pivotal role in plant immunity system. In the grapevine transcriptome, 75 differentially-expressed genes were detected to code disease resistance proteins, including 10 pathogenesis-related proteins PR-1 (10 up-regulated), nine beta-1, 3-glucanase (eight up-regulated, one down-regulated), 19 chitinase (19 up-regulated), 13 thaumatin-like proteins (13 up-regulated), two protease inhibitors (two up-regulated), four pathogenesis-related Bet v I family protein (four up-regulated), 10 lipid transfer proteins (nine up-regulated, one down-regulated), six germin-like proteins (six up-regulated) and two pathogenesis-related genes transcriptional activator (two up-regulated) (Table 4, Supplementary: Table S9). These results showed that most transcripts of pathogens resistance proteins were up-regulated except VIT_08s0007g06010.t01 and VIT_05s0020g03730.t01, which were of down-regulated ones (Table 4, Supplementary: Table S9). In this transcriptome, 8 transcripts were only expression in treatment group (VIT_03s0088g00890.t01, PR-1, |log_2_FC| = 10.06; VIT_05s0094g00320.t01, chitinase, |log_2_FC| = 7.49; VIT_02s0025g04290.t01, thaumatin-like protein, |log_2_FC| = 9.45; VIT_05s0020g05040.t01, protease inhibitor, |log_2_FC| = 6.38; VIT_05s0020g05000.t01, protease inhibitor, |log_2_FC| = 7.27; VIT_05s0077g01600.t01, pathogenesis-related Bet v I family protein, |log_2_FC| = 7.29, VIT_14s0128g00600.t01, germin-like protein, |log_2_FC| = 9.17 and VIT_09s0002g01320.t01, germin-like protein, |log_2_FC| = 10.07) (Supplementary: Table S9). Furthermore, 10 dirigent proteins and 13 proline related protein were up-regulated (Table 4, Supplementary: Table S9). These pathways played well-established roles in plant defense responses. Most of these genes were up-regulated by exogenous Cu, but most were expressed at a low or moderate abundance.

### Secondary metabolism biosynthetic pathways in response to Cu stress

It is widely known fact that the synthesis of secondary metabolites in plants is important parts of the defense responses of plants to biotic and abiotic stresses. In this study, 85 secondary metabolites related genes were identified after Cu stress treatment, all the genes were involved in shikimate acid (nine transcripts), alkaloid (two), anthocyanins (45), lignin (21) and terpenoid (eight) biosynthetic pathways (Table 5, Supplementary: Table S10).

**Figure 5:**
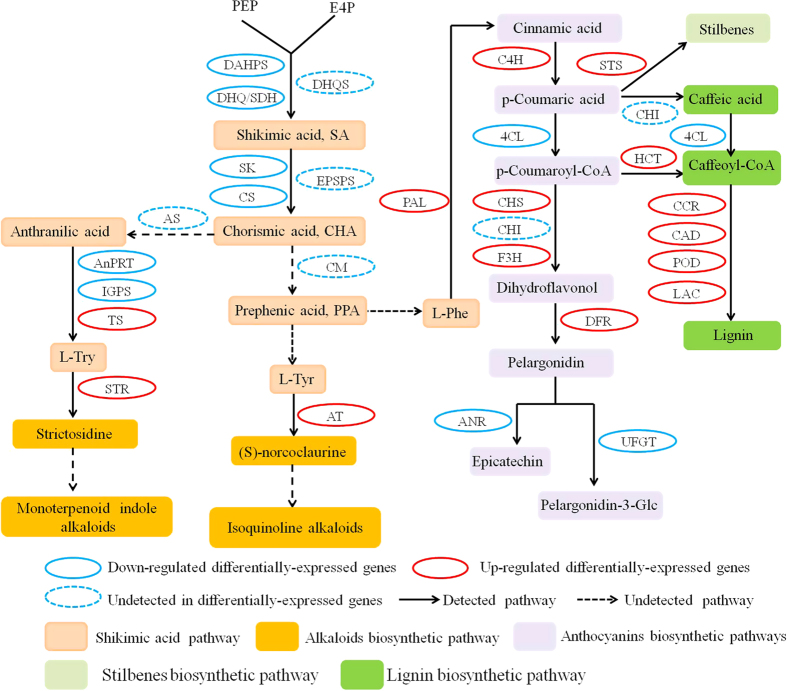
Differential expression genes related to secondary metabolism biosynthetic pathways under Cu stress. DAHPS, 3-deoxy-D-arabino-heptulosonate-7-phosphate synthase; DHQS, 3-dehydroquinate synthase; DHQ/SDH, bifunctional 3-dehydroquinate dehydratase/shikimate dehydrogenase; SK, shikimate kinase; EPSPS, 5 -enolpyruvylshikimate-3-phosphate synthase; CS, chorismate synthhase; AS, anthranilate synthase; CM, chorismate mutase; AnPRT, anthranilate phosphoribosyltransferase; IGPS, indole-3-glycerol phosphate synthase; TS, tryptophan synthase; STR, strictosidine synthase; AT, aminotransferase; PAL, phenylalanine ammonia-lyase; C4H, cinnamate-4-hydroxylase; C4L, 4-coumarate--CoA ligase; CHS/STS, chalcone and stilbene synthase; F3H, flavanone 3-dioxygenase; DFR, dihydroflavonol-4-reductase; UFGT, UDP glucose:flavonoid 3-o-glucosyltransferase; ANR, anthocyanidin reductase; HCT, hydroxycinnamoyl-Coenzyme A shikimate/quinate hydroxycinnamoyltransferase-like; CCR, cinnamoyl-CoA reductase; CAD, cinnamyl alcohol dehydrogenase; POD, peroxidase; LAC, laccase.

Shikimate acid is a key precursor for the synthesis of alkaloid and other secondary metabolism compounds. In the grapevine transcriptome, the shikimate acid pathway contained one down-regulated 3-deoxy-D-arabino-heptulosonate-7-phosphate synthase (DAHPS), two down-regulated bifunctional 3-dehydroquinate dehydratase/shikimate dehydrogenase (DHQ/SDH), one down-regulated shikimate kinase (SK), one down-regulated chorismate synthase (CS), two down-regulated anthranilate phosphoribosyltransferase (AnPRT), one down-regulated indole-3-glycerol phosphate synthase (IGPS) and one up-regulated tryptophan synthase (TS) (Table 5, Fig. 5, Supplementary: Table S10). All the nine identified shikimate acid genes had moderate and/or low transcript abundances (Supplementary: Table S10). In alkaloid biosynthesis pathway, one up-regulated strictosidine synthase (STR) and one up-regulated aminotransferase (AT) were identified as differentially-expressed genes (Table 5, Fig. 5, Supplementary: Table S10). Strictosidine synthase (STR) identified here in grapevine was reported to catalyze the condensation of tryptamine with secologanin to form strictosidine, is the central and first committed enzyme in alkaloid biosynthesis^31^.

Anthocyanins are one class of flavonoid compounds in grapevine that influence grape development and berry traits. They can also act as powerful antioxidants in scavenging diverse reactive oxygen species or inhibit their formation by chelating prooxidative metal ions^32^. A number of anthocyanins biosynthetic pathways related genes were induced by Cu stress. 39 and six transcripts, respectively, showed up-regulated and down-regulated expression in responding to Cu signaling compared with control (Table 5, Supplementary: Table S10), suggesting the Cu stress could induce the anthocyanins biosynthesis stronger. All the 12 PALs were obviously up-regulated (from 2.55 to 8.71 log_2_FC), among which VIT_00s2849g00010.t01 (log_2_FC = 8.55) and VIT_16s0039g01360.t01 (log_2_FC = 8.71) were significantly induced by Cu stress (Supplementary: Table S10). 21 chalcone and stilbene synthase (CHS/STS) were also obviously up-regulated (Supplementary: Table S10). Eight CHS/STS (VIT_16s0100g01150.t01, VIT_16s0100g01170.t01, VIT_16s0100g00900.t01, VIT_16s0100g01140.t01, VIT_16s0100g00770.t01, VIT_16s0100g00780.t01, VIT_16s0100g00910.t01 and VIT_16s0100g01100.t01) were significantly induced and expressed only under Cu treatment conditions (Supplementary: Table S10), indicating that they were genes specific to Cu metabolism or some other. In addition, cinnamate-4-hydroxylase (C4H), flavanone 3-dioxygenase (F3H) and dihydroflavonol-4-reductase (DFR) showed increased expression except VIT_18s0001g03470.t01 and VIT_19s0014g04990.t01. Two 4-coumarate-CoA ligase (C4L) showed decreased expression (Supplementary: Table S10).

Lignin is a phenolic polymer that reinforces the secondary cell wall, confers structural integrity to the plant, and also plays important roles in plant responses to various environment stresses^33^. Here in this study, 21 differentially expressed genes were involved to lignin biosynthetic pathways under Cu stress, including two up-regulated hydroxycinnamoyl-Coenzyme A shikimate/quinate hydroxycinnamoyltransferase-like (HCT), one up-regulated ferulic acid 5-hydroxylase (F5H), two cinnamoyl-CoA reductase (CCR, one up-regulated, one down-regulated), one up-regulated cinnamyl alcohol dehydrogenase (CAD), two caffeic acid 3-O-methyltransferase (COMT, one up-regulated, one down-regulated), two down-regulated caffeoyl-CoA O-methyltransferase (CCoAOMT), 7 up-regulated peroxidase (POD) and four laccase (LAC, one up-regulated, three down-regulated) (Table 5, Supplementary: Table S10). The results showed that most of transcripts in lignin biosynthetic were up-regulated. Among the four laccases, VIT_18s0117g00550.t01 was the one significantly induced by Cu stress (log_2_FC = 8.44) (Supplementary: Table S10).

Eight genes (4 up-regulated and 4 down-regulated) related to terpenoid biosynthesis were identified (Table 5, Supplementary: Table S10), and they were differentially expressed under Cu stress. Terpenoids play crucial roles in pollinator attraction, plant defense, and interaction with the surrounding environment. Cytosolic mevalonic-acid (MVA) and plastidial 2-C-methylerythritol 4-phosphate (MEP) pathway are responsible for the biosynthesis of these compounds^34^. From the sequencing data, two up-regulated hydroxymethylglutaryl-CoA synthase (HMGS) were identified. Contrarily, one down-regulated 1-deoxy-D-xylulose-5-phosphate synthase (DXPS) and one down-regulated 1-deoxy-D-xylulose and 5-phosphate reductoisomerase (DXPR) were identified as the genes involved in MEP pathway (Table 5, Supplementary: Table S10). DXPS and DXPR are rate-limiting enzyme in the MEP pathway. Isopentenyl diphosphate (IPP) and dimethylallyl diphosphate (DMAPP) are universal five carbon precursors in the synthesis of terpenoids. One isopentenyl-diphosphate isomerase (IDI), catalyzes IPP to form DMAPP^35^ and one terpene synthase (TPS), reported in the catalyzation of terpene formation^36^ were also up-regulated (Table 5, Supplementary: Table S10). In addition, two squalene epoxidase (SQE), catalyzing the synthesis of 2, 3-oxidosqualene, showed down-regulated expression. All eght transcripts had moderate and low expression abundance (Table 5, Supplementary: Table S10).

### Plant hormones in response to Cu stress

Plant hormones play pivotal roles in plant stress signaling and function as central integrators that link and re-program the complex developmental and stress adaptive signaling cascades. In this transcriptome, many genes involved in the auxin (IAA), ethylene (ET), jasmonic acid (JA), abscisic acid (ABA), brassinolide (BR) and gibberellin (GA) synthesis and signal transduction pathways were detected (Supplementary: Table S11). These pathways play well-established roles in plant defense against stress. Six auxin response factors (ARF) involving in transcriptional repressor and five WAT1-related protein involving in auxin transport were down-regulated. While most auxin-induced protein (Aux/IAA, two down-regulated and 10 up-regulated transcripts) and auxin synthesis-related genes (one down-regulated and five up-regulated transcripts) were up-regulated in response to Cu stress (Supplementary: Table S11). 26 ethylene-responsive transcription factors (ERF), being crucial to the ET signaling pathway, were identified and most of them were up-regulated (22 up-regulated and four down-regulated) under Cu treatment (Supplementary: Table S11). Five ACC oxidases (ACO) involving in the final step of ethylene production were found to be up-regulated too (Supplementary: Table S11). The jasmonate ZIM-domain proteins (JAZs) identified were three down-regulated and one up-regulated ones. Lipoxygenase (LOX), allene oxide synthase (AOS) and 12-oxophytodienoate reductase 2-like (OPR), 3 JA synthesis-related genes, were also up-regulated under Cu stress (Supplementary: Table S11). Two abscisic acid-responsive protein (one up-regulated and one down-regulated) and four ABA synthesis-related genes (4 down-regulated) involved in ABA pathways were identified under Cu stress (Supplementary: Table S11). The numbers of the genes invoving in brassinolide pathway and GA signal were fewest. Three up-regulated BRASSINOSTEROID INSENSITIVE 1-associated receptor kinase 1 (BAK1) were identified in brassinolide pathway (Supplementary: Table S11). Finally, 6 GA-related transcripts (4 up-regulated and 2 down-regulated transcripts) were also detected under the Cu stress (Supplementary: Table S11).

### Cu homeostasis pathways in response to Cu stress

**Figure 6:**
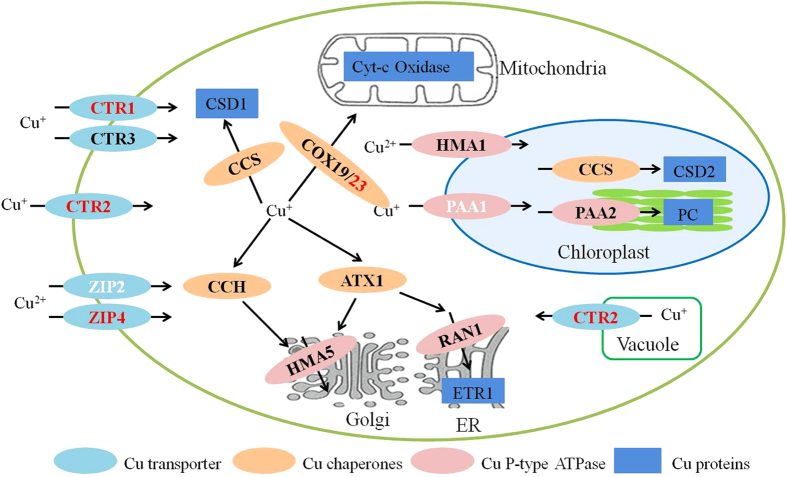
Differential expression genes related to Cu transport and distribution in grapevine under Cu stress. The red and white bold were up-regulated and down-regulated genes respectively. The black bold was no change in expression level. CTR, copper transporter; ZIP, zinc transporter; COX19/23, cytochrome c oxidase-assembly factor COX19/23; CCS, Cu/Zn-superoxide dismutase copper chaperone; CCH, copper transport protein CCH; ATX1, copper transport protein ATX1; PAA1/2, copper-transporting ATPase PAA1/2; HMA1, cadmium/zinc-transporting ATPase HMA1; HMA5,copper-transporting ATPase HMA5; RAN1, copper-transporting ATPase RAN1.

To avoid copper toxic effects, plants have developed complex homeostatic networks to control copper uptake, accumulation, distribution, and utilization in response to environmental Cu level variations^37^. Many genes involved in Cu uptake and trafficking via P-type ATPases and Cu chaperones were regulated by Cu stress. In plants, high affinity of copper uptake is mediated by the Ctr family of copper transporters. In our study, three up-regulated Cu transporters (CTR1, CTR2 and CTR8) were identified, probably to facilitate the Cu transportation under oxidative stress (Fig. 6, Supplementary: Table S12). ZIP2 and ZIP4, two ZIP genes involving in Cu transport, were down-regulated and up-regulated, respectively. No change was observed about Cu transporters CTR3 (Fig. 6, Supplementary: Table S12).

Copper chaperones have been demonstrated to deliver copper to their respective targets through direct protein-protein interactions, inserting copper into the active site of the respective enzyme or transport protein^38^. Most genes coding for proteins with important functions for copper chaperones were also identified and showed no obvious change of expression when treated with Cu, including a Cu/Zn-superoxide dismutase copper chaperone CCS (VIT_02s0025g04830.t01), cytochrome c oxidase assembly protein COX19 (VIT_09s0002g03960.t01), copper transport protein CCH (VIT_13s0074g00770.t01), and copper transport protein ATX1 (VIT_16s0098g00800.t01). Only one transcript, cytochrome c oxidase-assembly factor COX23 (VIT_15s0021g01120.t01), were up-regulated (Fig. 6, Supplementary: Table S12). Seven P-type heavy metal ATPases, involving in the transport of a range of essential and potentially toxic metals (i.e., Cu^2+^, Zn^2+^, Cd^2+^, Pb^2+^) across cell membranes^39^, were identified by Cu stress. Among them, two copper-transporting ATPase PAA1 (VIT_12s0142g00330.t01 and VIT_12s0142g00400.t01) were down-regulated after Cu stress treatment, among which a copper-transporting ATPase PAA1 (VIT_12s0142g00420.t01), copper-transporting ATPase PAA2 (VIT_04s0008g01960.t01), cadmium/zinc-transporting ATPase HMA1 (VIT_07s0129g01040.t01), copper-transporting ATPase HMA5 (VIT_02s0025g03630.t01) and copper-transporting ATPase RAN1 (VIT_01s0011g01360.t01) showed no change in their expression levels under Cu stress.

It is noteworthy that several additional stress-related transport systems were specifically up-regulated by Cu stress. They were ABC transporters and multidrug and toxic compound extrusion protein (MATE). 10 out of 13 ABC transporters and 8 out of 13 MATE were up-regulated, suggesting their importance in respond to Cu stress (Supplementary: Table S12). Additionally, 11 WRKY transcription factor (1 down-regulated and 10 up-regulated), a well known players in plant response to abiotic and biotic stress, were identified (Supplementary: Table S12).

### Quantitative real-time-PCR validation of differentially expressed transcripts from RNA-seq

To confirm the accuracy and reproducibility of this Illumina RNA-seq result, 20 transcripts were chosen randomly for quantitative real-time (qRT) PCR. Those genes were involved in transcription factor, metabolism, information transfer, transcription factor, and included up-regulated, downregulated, and unaffected transcripts. The primer sequences, gene functions, and RPKM and qRT-PCR result are listed in Supplementary: Table S13 and Supplementary Fig. S3. The qRT-PCR results generally agreed (85%) with the changes in transcript abundance determined by RNA-seq, suggesting the reliability of the RNA-seq data.

## Discussion

Cu is extremely toxic to plant growth at high concentrations, and induces oxidative stress potentially leading to physiological disorders that inhibit plant growth. Photosynthesis is a fundamental and vital metabolic process for plant growth development, and very sensitive to unfavorable conditions. Chlorophyll is the predominant light-absorbing pigment for photosynthesis in plants. In grapevine, Cu stress led to a significant inhibition in the photosynthetic activities and remarkable decrease in the chlorophyll content in transcriptomic level and physiological processes (Table 1 and Fig. 1). The result was consistent with previous studies that the Cu-induced chlorophyll loss was associated with the decrease of photosynthetic activities^40^. Further, transcriptomic data demonstrated that the application of exogenous Cu inhibited the activity of chlorophyll biosynthesis enzymes and induced the activity of chlorophyll degradation enzymes (Supplementary Table S6). The decline in chlorophyll content is believed to be due to a acceleration in chlorophyll degradation and/or the blocking of chlorophyll synthesis.

At the same time, photosynthetic activity (Fv/Fm), was significantly decreased in the leaves of Cu-stressed grapevine (Table 1 and Fig. 1), and the similar result was also observed in brown algae^41^. The values of Fv/Fm reflect the potential quantum efficiency of PSII and are usually used as a sensitive indicator of photosynthetic performance of higher plants. Furthermore, most photosynthesis-related genes, involving in PSII, PSI, cytochrome b6-f complex, ATP synthase, photosynthetic electron transport chain and photosynthesis-antenna proteins, were also significantly decreased (Supplementary Table S7). Interestingly, seven transcripts in PSII showed significantly differentially-expressed, while only one transcripts in PSI had differentially-expressed, suggested that PSII was more sensitive than PSI under Cu stresses. The result was consistent with previous reports that PSI was usually more stable than PSII under stresses^42^. In our study, curvature thylakoid 1 (CURT1) and thylakoid formation 1 (THF1) were down-regulated under Cu stress, which was consistent with a few previous studies Cu stress damage the structure and composition of the thylakoid membrane^43^,^44^. Altogether, these results in transcriptomic and physiological level suggested that Cu stress in grapevine was undoubtedly tightly tied to primary photosynthesis metabolic processes, and the decrease of photosynthetic activities was associated with chlorophyll loss and thylakoid membrane damage.

Cu stresses lead to excessive production of ROS causing progressive oxidative damage to cellular components and ultimately cell death. NADPH oxidase has been proposed to play a key role in the production and accumulation of ROS in plants under stress conditions^7^. In our study, two NADPH oxidases and five amine oxidases were significantly up-regulated (Supplementary Table S8) and contribute to ROS production in response to Cu stress. In order to avoid the oxidative damage, plants possess a complex antioxidative defense system comprising of nonenzymatic and enzymatic components. Significant increase in the activities of POD, CAT and SOD was observed in Cu-treated leaves indicating that the ROS-scavenging systems can have an important role in managing ROS generated in response to Cu stress. Among them, SODs constitute the first line of defence against ROS. Plant chloroplasts have two isozymes, copper/zinc SOD (Cu/Zn-SOD) and iron SOD (FeSOD)^45^. It is noteworthy that the expression of Fe-SOD was remarkably down-regulated in grapevine, and the result was consistent with the known facts that FeSOD was replaced with Cu/Zn-SOD when Cu was available (Fig. 4). In other words, FeSOD activity was decreased in a Cu-containing medium and increased under Cu-deficient conditions^45^,^46^. The antioxidative defense system also included POD, CAT, GST, polyphenol oxidase (PPO), alternative oxidase (AO), enzymes of AsA-GSH cycle ascorbate peroxidase (APX), monodehydroascorbate reductase (MDHAR), dehydroascorbate reductase (DHAR), and glutathione reductase (GR)^47^. These enzymes operated in different subcellular compartments and responded in concert when cells are exposed to Cu stress in graoevine. Furthermore, nonenzymic antioxidative defense system include the major cellular redox buffers ascorbate (AsA) and glutathione (GSH) as well as proline and phenolic compounds. These nonenzymic antioxidant also had a key role in defense against Cu stress caused by enhanced level of ROS in grapevine. These results using transcriptome and analytical techniques will help in broader understanding of ROS production and scavenging involved in cellular responses to Cu stress in grapevine. Improved understanding of these will be helpful in producing grapevine with in-built capacity of enhanced levels of tolerance to ROS using biotechnological approach.

Heat shock proteins (HSPs) and pathogenesis-related (PR) proteins are ubiquitous stress-related proteins, which were dramatically increased expression in response to variable environmental conditions. HSPs are expressed at low to moderate levels under normal growth conditions, members of the HSP family as molecular chaperones, participate in the early stages of protein synthesis, protein folding, protein aggregation, and the transport of newly synthesized proteins from the cytoplasm into different intracellular compartments^48^. Under conditions of stress, where protein folding/assembly events may be compromised, the increased expression and accumulation of the stress proteins facilitates the ability of cells to both repair and synthesize new proteins to replace those that were damaged after the particular metabolic insult^49^. In our study, several higher molecular weight HSPs (HSP70, HSP90s and HSP101) were down-regulated, while most sHSPs (sHSPs16–30 kDa) and heat stress transcription factors (HSFs) were up-regulated (Supplementary Table S9). These observations suggest that the HSP genes may play different important roles in plant development and stress responses. Furthermore, the relationship between Cu stress and HSPs will be interesting to investigate in future. Pathogenesis related (PR) proteins are one of the major sources of plant derived allergens and induce upon stress, pathogen attack and abiotic stimuli as a defense response. Currently PR-proteins encompasses 17 families according to their properties and functions, sunch as β-1,3-glucanases, chitinases, thaumatin-like proteins, peroxidases, as well as small proteins such as defensins, thionins and lipid transfer proteins (LTPs)^50^,^51^. In our study, most PR proteins were remarkably up-regulated, suggesting the important role of PR proteins in defense response and help the plant to adapt to Cu stress. Further, dirigent protein (DIR), involving in formation of lignin^52^, were also significantly up-regulated, suggesting the importance in Cu stress.

Mecondary metabolites play an indisputable important role in the adaptation of plants to the changing environment and in overcoming stress constraints. The shikimate pathway, a bridge between central metabolism and secondary metabolism, is the only known pathway for biosynthesis of chorismate and the aromatic amino acids Phe, Tyr and Try, which serve as key precursors for a wide range of secondary metabolites, such as alkaloid, phenolic and plant hormones^53^. In our transcriptome study, most of genes involving in shikimate pathway showed down-regulation, suggested that Cu stress had a negative impact in shikimate pathway. Further, Tyr and Try respectively leads to the synthesis of isoquinoline alkaloids and indole alkaloids, which have an important function in prevention and treatment of oxidative stress^54^. Try is also a precursor for indole-3-acetic acid (IAA, auxin). Phe serves as a precursor for a large family of secondary metabolites and PAL is the first and committed enzyme in the phenylpropanoid biosynthesis pathway, therefore a key step in the biosynthesis of the favonoids, lignins, stilbenes and many other compounds^55^. Chalcone synthase (CHS) catalyzes the first step in flavonoid biosynthesis, whose expression could be induced in response to environmental stress^56^. In our study, all 12 PAL and 21 chalcone and stilbene synthase were significantly up-regulated, suggested that the intimate connection between Cu stress and PAL and CHS/STS. Most related gene in the biosynthesis of the anthocyanins, lignins, stilbenes also were significantly up-regulated, illustrated mecondary metabolites that are often associated with improved plant resistance to Cu stress.

Phytohormones, such as salicylic acid (SA), ethylene (ET), jasmonic acid (JA) and abscisic acid (ABA), are crucial signaling molecules and play central roles in responses to abiotic and biotic stress signaling. For example, SA, JA and ET have fundamental roles in biotic stress signaling. ABA is involved in the response to abiotic stress and appears to function as a negative regulator in disease resistance, in opposite action to SA, ET and JA. In our study, IAA, ET, JA, ABA, BR and GA were identified in response to Cu stress. Most key enzymes for JA (LOX, AOS and OPR), ethylene (ACO) biosynthesis and ethylene-responsive transcription factor were significantly up-regulated (Supplementary Table S11), suggested that ethylene and JA play a positive role as a ignaling molecules in response to Cu stress. However, most ABA signaling were down-regulated, suggested the fact that ABA signaling may play a negative regulator in Cu stress, in opposite action to ET and JA. Further, BRs, the new class of steroidal hormones, confer tolerance in plants to cope with biotic and abiotic stress stress^57^. Three BAK1, which is also essential for BR signal transduction, were up-regulated, possibly to facilitate the important role of BRs in the plant response to Cu stress. Additionally, all ARFs, transcriptional activators and repressors^58^, were down-regulated and most AUX/IAA proteins and IAA synthase were up-regulated by Cu stress in IAA pathway. These observations imply that the IAA regulated genes may play positive roles in plant development and abiotic stress responses. Furthermore, the relationship between IAA and oxidative stress tolerance will be interesting to investigate in the future.

Higher plants have developed sophisticated mechanisms to efficiently acquire and utilize Cu. Cu transport toward the cytoplasm is mediated by the conserved CTR/COPT family of high-affinity Cu transport proteins. The up-regulation of CTR1 and CTR2, respectively high and low affinity copper transport proteins, facilitated the Cu transportation under oxidative stress. CTR2 was also an intracellular Cu transporter which mobilized Cu from the vacuolar storage compartment when Cu is extremely scarce^59^. Interestingly, CTR8, analogous to the high-affinity copper uptake protein CTR1, was also up-regulation in our study. While previous reports showed VvCTr8 decreased sharply when cells were grown in excess CuSO_4_^18^. Furthermore, the role of CTR8 in copper uptake and transport will be interesting to investigate in the future. In addition, ZIP2 and ZIP4 showed the different expression patten under Cu stress compared with control, supported the fact that ZIP2 and ZIP4 proteins may transport divalent Cu^2+^ ions. After entering the cytoplasm, Cu is delivered to specific Cu-containing proteins by specialized Cu chaperones. For example, the CCS1 Cu chaperone supply Cu to Cu/Zn-SOD. PAA1 ATPase, which is located in the inner chloroplast envelope, mediates Cu transport from the cytoplasm to plastid stroma, and the thylakoid-located PAA2 facilitates final Cu delivery to plastocyanin into the thylakoid lumen^60^,^61^,^62^. In our study, two PAA1 ATPase were down-regulated, suggested that such proteins play an important role in Cu compartmentalization and excretion in plant^41^. While CCS1 and PAA2 showed no notable changes. Additionally, ABC and MATE family of transporters are important multidrug transporters superfamilies^63^. ABC transporters, which act as primary active transporters, are best known for their role in the import of essential nutrients and the export of toxic molecules^64^. MATE family proteins, which are a group of secondary active transporters, function as proton-dependent efflux transporters^63^. In our study, most identified ABC and MATE family of transporters were up-regulation under Cu stress, which played important role in Cu-detoxification in grapevine.

## Conclusion

This study identified numerous genes that are differentially expressed between control and Cu stressed, and provided an overview of Cu stress acclimation in grapevine. Genome-wide transcriptome analysis results indicated that exogenous Cu down-regulated many genes related to chlorophyll synthesis and photosynthesis, and also up-regulated many genes related to stress tolerance, including antioxidant defense systems, as well as the stress-related proteins and secondary metabolites. Several different plant hormones involving in IAA, ET, JA, BR, and ABA signaling interactions were also identified in response to Cu stress. This expression results indicates several classes of genes that might have a specific roles under Cu stress. Finally we also observed, Cu stress specifically activated a set of genes coding for copper transporter, P_1B_-type ATPase, and multidrug transporters. This study will help to provide a foundation and clear understanding of complex molecular mechanisms involved in cellular responses to Cu stress. There is also an challenge urgent need to find and characterize the complex interactions between Cu stress and defense in whole plants, with better assess agronomic practices and management to enhance plant growth and accelerate the progress of cultivating Cu-resistant plants for crop improvement.

## Methods

### Plant material

Two-year old ‘Summer Black’ grapevine (hybrids of *V. vinifera* and *V. labrusca*) trees were grown under the standard field conditions at the Nanjing Agricultural University fruit farm, Nanjing, China were chosen as the experimental material, 100 μM concentration of CuSO_4_ were chosen as the copper stress treatment because it can led to a drastic decrease of several physiological and growth traits in preliminary experiments. Twenty-four hours after treatment, the young third and fourth leaves from the stem apex from both the Cu-treated and control groups were collected. Each type of samples had three replicates during deep sequencing and qRT-PCR. All the samples were immediately frozen in liquid nitrogen and stored at −80 °C for further use.

### RNA extraction, cDNA library construction and Illumina deep sequencing

Total RNA samples of Cu-treated and control were extracted using Trizol reagent (Invitrogen, Carlsbad, CA, USA) and subsequently used for mRNA purification and library construction with the Ultra™ RNA Library Prep Kit for Illumina (NEB, USA) following the manufacturer’s instructions. The samples were sequenced on an Illumina Hiseq™ 2500. Each sample yielded more than 4 Gb of data. Sequencing was completed by the Shanghai Hanyu Biotechnology Company (Shanghai, China).

### Analysis of gene expression level

After adaptor trimming and quality trimming, the clean reads were mapped to the *V. vinifera* transcriptome using Bowtie2^65^. Then, SAM tools and BamIndexStats.jar were used to calculate the gene expression level, and RPKM value from SAM files. Gene expression difference between log and early stationary phase were obtained by MARS (MA-plot-based method with Random Sampling model), a package from DEGseq^66^. We simply defined genes with at least 1-fold change between two samples and FDR (false discovery rate) less than 0.001 as differential expressed genes. Transcripts with |log_2_FC| < 1 were assumed have no change in expression levels.

### Determination of several important physiological traits

The concentrations of copper in leaves were determined by inductively coupled plasma-optical emission spectrometry (ICP-OES) with Perkin-Elmer Optima 2100 DV ICP-OES instrument at 324.75 nm wavelength. Maximum quantum yield of PSII (Fv/Fm) was measured using a Walz Phyto-PAM (Waltz, Germany) as previously described^41^. The chlorophyll a and b are respectively determined by spectrophotometric measurement at 663 and 645 nm. Malondialdehyde (MDA) content was measured as previously described by thiobarbituric acid method^67^. SOD activities were determined by monitoring its ability to inhibit photochemical reduction of nitroblue tetrazolium (NBT) at 560 nm^68^. POD activities were determined by using the guaiacol oxidation method^69^. CAT activities were determined by monitoring the disappearance of H_2_O_2_ and by measuring the decrease in absorbance at 240 nm^70^. Data are expressed as mean ± standard deviation (SD) and subjected to a one-way analysis of variance (ANOVA). All analysis was carried out in at least three replicates for each sample. Results were analyzed statistically using SPSS 15.0. A value of p < 0.05 was considered statistically significant.

### qRT-PCR Validation

qRT-PCR was performed to verify the expression patterns revealed by the RNA-seq study. The purified RNA samples were reverse-transcribed using the PrimeScript RT Reagent Kit with gDNA Eraser (Takara, Dalian, China) following the manufacturer’s protocol. Twenty transcripts were selected randomly for the qRT–PCR assay. Gene specific qRT–PCR primers were designed using Primer3 software (http://primer3.ut.ee/). Gene-specific primers were designed for 11 genes using the sequence information in the 3′ UTR, nine genes primers were designed to anneal in the coding sequence. qRT-PCR was carried out using an ABI PRISM 7500 real-time PCR system (Applied Biosystems, USA). Each reaction contains 10 μl 2 × SYBR Green Master Mix Reagent (Applied Biosystems, USA), 2.0 μl cDNA sample, and 400 nM of gene-specific primer in a final volume of 20 μl. PCR conditions were 2 min at 95 °C, followed by 40 cycles of heating at 95 °C for 10 s and annealing at 60 °C for 40 s. The relative mRNA level for each gene was calculated as ΔΔCT values^71^. A primer pair was also designed for TC81781 (The Institute for Genomic Research, Release 6.0), encoding an actin protein. At least three replicates of each cDNA sample were performed for qRT-PCR analysis.

## Additional Information

**How to cite this article**: Leng, X. *et al.* Comparative transcriptome analysis of grapevine in response to copper stress. *Sci. Rep.* **5**, 17749; doi: 10.1038/srep17749 (2015).

## Supplementary Material

Supplementary Information

## Figures and Tables

**Table 1:** Some physiological parameters in leaves of grapevine under Cu stress.

physiological and biochemical parameters	CK	Cu treatment	Range of increasing (%)
Cu contents (μg/g)	10.51 ± 0.81	31.06 ± 1.21	195.5
Chlorophyll contents (mg/g)	2.35 ± 0.11	1.61 ± 0.08	−31.5
Chla contents (mg/g)	1.76 ± 0.09	1.19 ± 0.06	−32.4
Chlb contents (mg/g)	0.59 ± 0.04	0.42 ± 0.03	−28.8
MDA content (nmol/g)	5.55 ± 0.19	9.77 ± 0.39	76.0
SOD activity (U g^−1^ min^−1^)	456.97 ± 23.04	969.46 ± 64.03	112.2
CAT activity (U g^−1^ min^−1^)	5.47 ± 1.05	16.11 ± 1.34	194.5
POD activity (U g^−1^ min^−1^)	15.69 ± 1.89	56.97 ± 2.70	263.1

**Table 2:** Information of differential expressed genes related to chlorophyll metabolic and photosynthesis of grapevine under Cu stress.

Trait	Description	No. of up-regulated	No. of down-regulated	Sun
chlorophylls metabolism	chlorophyll a synthesis	0	13	13
	chlorophyll cycle	0	1	1
	chlorophyll degradation	1	0	1
	phytochromobilin synthesis	0	2	0
photosystem II	psbA	0	2	2
	psbB	0	1	1
	psbP	0	2	2
	psbM	1	0	1
	psbW	1	0	1
photosystem I	psaB	0	1	1
cytochrome b6-f complex	petA	0	1	1
	petC	0	2	2
photosynthetic electron transport	petE	1	0	1
	petF	1	0	1
	petH	0	1	1
	petJ	0	1	1
F-type ATPase	ATPF0B	0	4	4
	ATPF0C	0	1	1
Photosynthesis-antenna proteins	LHCA4	0	1	1
	LHCB6	0	1	1
thylakoid part	THF1	0	1	1
	CURT1	0	2	2
	TPL	0	2	2
Others	PKS1	0	1	1
	PIF3	0	1	1
	HCF136	0	1	1

psbA, photosystem II P680 reaction center D1 protein; psbB, photosystem II CP47 chlorophyll apoprotein; psbP, photosystem II oxygen-evolving enhancer protein 2; psbM, photosystem II PsbM protein; psbW, photosystem II PsbW protein; psaB, photosystem I P700 chlorophyll a apoprotein A2; petA, apocytochrome f; petC, cytochrome b6-f complex iron-sulfur subunit; petE, plastocyanin; petF, ferredoxin; petH, ferredoxin–NADP^+^ reductase; petJ, cytochrome c6; ATPF0B, F-type H + -transporting ATPase subunit b; ATPF0C, F-type H + -transporting ATPase subunit C; LHCA4; light-harvesting complex I chlorophyll a/b binding protein 4; LHCB6; light-harvesting complex II chlorophyll a/b binding protein 6; THF1, thylakoid formation 1; CURT1, curvature thylakoid 1; TPL, thylakoid lumenal protein; PKS1, phytochrome kinase substrate 1; PIF3, phytochrome-interacting factor 3; HCF136, photosystem II stability/assembly factor HCF136.

**Table 3:** Information of differential expression genes related to ROS producing and scavenging system under Cu stress.

Trait	Description	No. of up-regulated	No. of down-regulated	Sun
ROS Producing	Rboh	2	0	2
	AO	5	0	5
ROS scavenging	Fe-SOD	0	2	2
	POD	7	0	7
	CAT	2	1	3
GSH-AsA cycle	APX	1	0	1
	MDAR	0	1	1
	DHAR	0	1	1
	GR	0	1	1
	Grx	3	4	7
GPX pathway	GPX	0	1	1
	GST	27	3	30
Prx/Trx pathway	Prx	0	2	2
	Trx	3	5	8
cyanide-resistant respiration	AOX	3	0	3
copper-containing enzymes	PPO	2	0	2

Rboh, respiratory burst oxidase; AO, amine oxidase; Fe-SOD, Fe superoxide dismutase; POD, peroxidase; CAT, catalase; APX, ascorbate peroxidases; MDAR, monodehydroascorbate reductase; DHAR, dehydroascorbate reductase; GR, glutathione reductase; Grx, Glutaredoxin; GPX glutathione peroxidases; GST, glutathione S-transferase; Prx, peroxiredoxin; Trx, thioredoxin; AOX, alternative oxidase; PPO, polyphenol oxidase.

**Table 4:** Information of differential expression genes related to heat shock proteins (HSPs) and pathogens resistance proteins.

Trait	Description	No. of up-regulated	No. of down-regulated	Sun
Heat shock proteins	HSP101	0	1	1
	HSP90s	0	3	3
	HSP70	0	2	2
	small HSP	16	2	18
	other HSP	6	14	20
	heat stress transcription factor	4	1	5
PR-1		10	0	10
PR-2	beta-1,3-glucanase	8	1	9
PR-3, 4, 8, 11	chitinase	19	0	19
PR-5	thaumatin-like protein	13	0	13
PR-6	protease inhibitor	2	0	2
PR-10	pathogenesis-related Bet v I family protein	4	0	4
PR-14	lipid transfer protein	9	1	10
PR-15	germin-like protein	6	0	6
PTI	transcriptional activator	2	0	2
dirigent protein		10	0	10
proline related protein		13	0	13

**Table 5:** Information of differential expression genes related to secondary metabolism biosynthetic pathways under Cu stress.

Trait	Description	No. of up-regulated	No. of down-regulated	Sun
shikimate acid pathway	DAHPS	0	1	1
	DHQ/SDH	0	2	2
	SK	0	1	1
	CS	0	1	1
	AnPRT	0	2	2
	IGPS	0	1	1
	TS	1	0	1
alkaloid biosynthetic pathways	STR	1	0	1
	AT	1	0	1
anthocyanins biosynthetic pathways	PAL	12	0	12
	C4H	1	0	1
	C4L	0	2	2
	CHS/STS	21	0	21
	F3H	4	1	5
	DFR	1	1	2
	UFGT	0	1	1
	ANR	0	1	1
lignin biosynthetic pathways	HCT	2	0	2
	F5H	1	0	1
	COMT	1	1	2
	CCoAOMT	0	2	2
	CCR	1	1	2
	CAD	1	0	1
	POD	7	0	7
	LAC	1	3	4
terpenoid biosynthetic pathways	HMGS	2	0	2
	DXPS	0	1	1
	DXPR	0	1	1
	IDI	1	0	1
	TPS	1	0	1
	SQE	0	2	2

DAHPS, 3-deoxy-D-arabino-heptulosonate-7-phosphate synthase; DHQ/SDH, Bifunctional 3-dehydroquinate dehydratase/shikimate dehydrogenase; SK, shikimate kinase; CS, chorismate synthase; AnPRT, anthranilate phosphoribosyltransferase; IGPS, indole-3-glycerol phosphate synthase; TS, tryptophan synthase; STR, strictosidine synthase; AT, aminotransferase; PAL, phenylalanine ammonia-lyase; C4H, cinnamate-4-hydroxylase; C4L, 4-coumarate–CoA ligase; CHS/STS, chalcone and stilbene synthase; F3H, flavanone 3-dioxygenase; DFR, dihydroflavonol-4-reductase; UFGT, UDP glucose:flavonoid 3-o-glucosyltransferase; ANR, anthocyanidin reductase; HCT, hydroxycinnamoyl-Coenzyme A shikimate/quinate hydroxycinnamoyltransferase-like; F5H, ferulic acid 5-hydroxylase; COMT, caffeic acid 3-O-methyltransferase; CCoAOMT, caffeoyl-CoA O-methyltransferase; CCR, cinnamoyl-CoA reductase; CAD, cinnamyl alcohol dehydrogenase; POD, peroxidase; LAC, laccase; HMGS, hydroxymethylglutaryl-CoA synthase; DXPS, 1-deoxy-D-xylulose-5-phosphate synthase; DXPR, 1-deoxy-D-xylulose 5-phosphate reductoisomerase; IDI, isopentenyl-diphosphate isomerase; TPS, terpene synthase; SQE, squalene epoxidase.
