# Supplementary material for: Comparative transcriptome analysis of grapevine in response to copper stress
Source: Sci Rep. 2015 Dec 17;5:17749. doi: 10.1038/srep17749 (PMC4682189; doi:10.1038/srep17749)
Supplement: Supplementary Information [file srep17749-s1.pdf]

## Manuscript title :

Comparative transcriptome analysis of grapevine (Vitis vinifera) in response to copper stress

## Author list:

Xiangpeng Leng, Haifeng Jia,  Xin Sun, Lingfei Shangguan, Qian Mu, Baoju Wang & Jinggui Fang*

## Supplementary figures

**Fig. S1** Gene Ontology (GO) functional annotation of transcripts.

**Fig. S2** KEGG Function classification results of transcripts.

**Fig. S3** Real time RT-PCR validation of differentially expressed transcripts from RNA-seq.

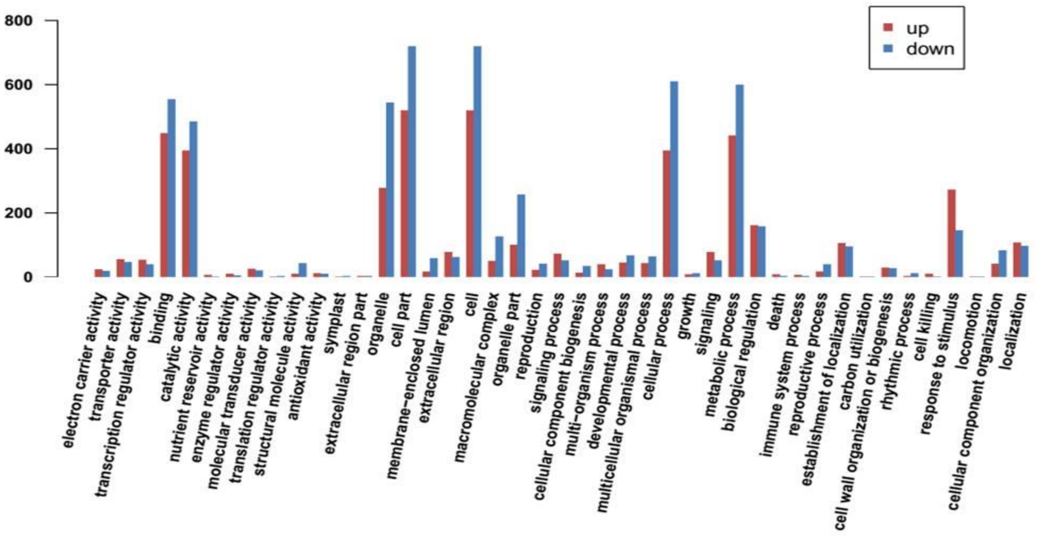


**Fig. S1** Gene Ontology (GO) functional annotation of transcripts.

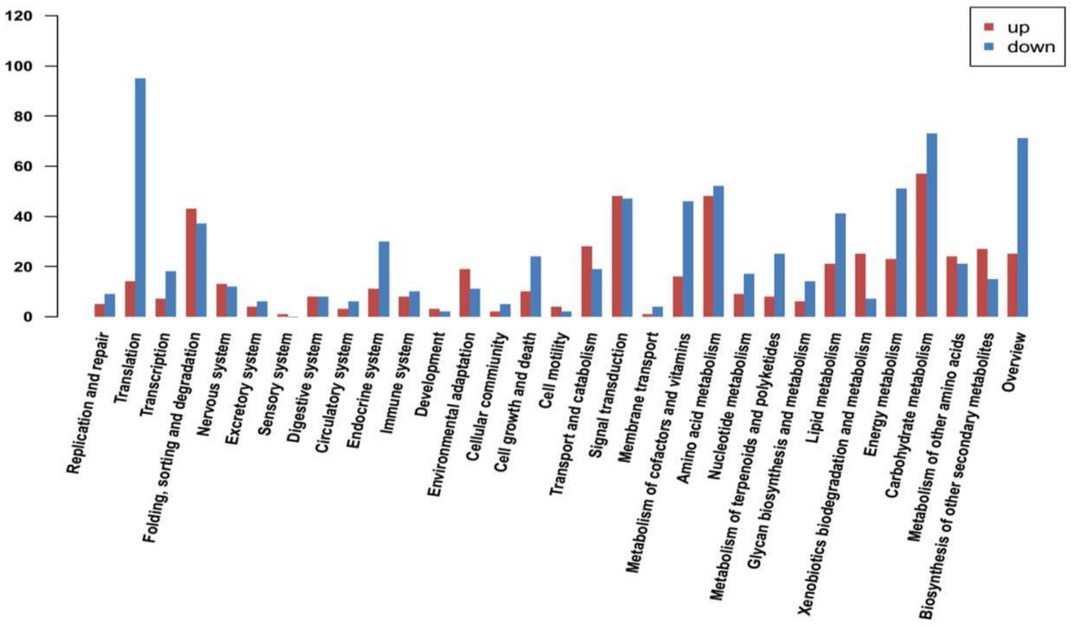


**Fig. S2** KEGG Function classification results of transcripts.

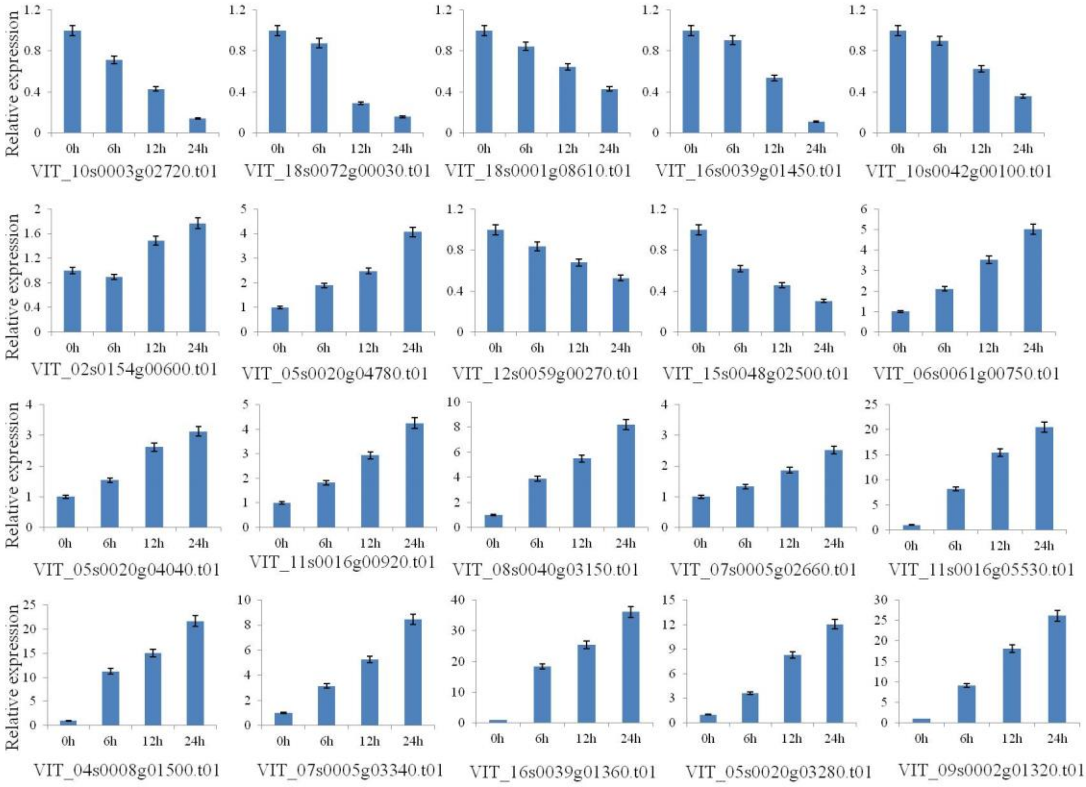


**Fig. S3** Real time RT-PCR validation of differentially expressed transcripts from RNA-seq.

## Supplementary tables

**Table S1** Number of reads sequenced and mapped to the grapevine genome.

**Table S2** Annotation of 3,843 significantly differentially-expressed genes transcripts according to the NCBI non-redundant, Gene Ontology, and the grapevine genome.

**Table S3** Transcripts expressed only in the Cu-treated or control group.

**Table S4** Gene Ontology (GO) functional annotation of transcripts.

**Table S5** KEGG pathway mapping.

**Table S6** Differently-expressed genes related to chlorophylls metabolism.

**Table S7** Differently-expressed genes related to photosynthesis.

**Table S8** Differently-expressed genes related to ROS producing and scavenging system.

**Table S9** Differently-expressed genes related to heat shock protein (HSP) and pathogenesis-related proteins (PR).

**Table S10** Differently-expressed genes related to secondary metabolism biosynthetic pathways.

**Table S11** Differently-expressed genes related to plant hormones.

**Table S12** Differently-expressed genes related to Cu homeostasis pathways.

**Table S13** Comparison of expression patterns between RNA-seq expression and qPCR.

Supplementary Table S1 Number of reads sequenced and mapped to the grapevine genome

| | Control | Cu treatment | Sum |
|---|---|---|---|
| Raw Reads | 21236463 | 21895350 | 43131813 |
| Clean Reads | 20230252 | 21060657 | 41290909 |
| Total mapped (percent of clean reads) | 10999416(54.37%) | 14808653(70.31%) | 25808069(62.50%) |
| Unique_match (percent of clean reads) | 9475421(46.84%) | 12409984(58.92%) | 21885405(53.00%) |
| Mutliple_match (percent of clean reads) | 1523995(7.53%) | 2398669(11.39%) | 3922664(9.50%) |
| Total unmapped (percent of clean reads) | 9230836(45.63%) | 6252004(29.69%) | 15482840(37.50%) |

Table S2 Annotation of 3,843 significantly differentially-expressed genes transcripts according to the NCBI non-redundant, Gene Ontology, and the grapevine genome

| #Gene | Length | Function | Cu-treated | RPKM_A | control | RPKM_B | log2(Fold_c | q-value(Benja | p_value | Result |
|---|---|---|---|---|---|---|---|---|---|---|
| VIT_15s0024g00900.t01 | 672 | trnF-ndhJ intergenic spacer | 0.5 | 0.0502441 | 69 | 9.3349112 | -7.537538 | 1.44E-18 | 2.20E-19 | down |
| VIT_10s0003g02720.t01 | 713 | glucan endo-1,3-beta-glucosidase 12-like | 0.5 | 0.0473549 | 42 | 5.355378 | -6.821331 | 2.96E-12 | 6.17E-13 | down |
| VIT_08s0056g00820.t01 | 1173 | cysteine synthase | 1 | 0.0575687 | 77 | 5.9679213 | -6.6958 | 1.08E-21 | 1.44E-22 | down |
| VIT_00s2301g00010.t01 | 272 | Putative uncharacterized protein | 1 | 0.248265 | 63 | 21.057268 | -6.406293 | 3.77E-18 | 5.84E-19 | down |
| VIT_05s0136g00380.t01 | 285 | NADH dehydrogenase [ubiquinone] complex I, | 0.5 | 0.1184703 | 31 | 9.8888823 | -6.38321 | 1.69E-09 | 4.32E-10 | down |
| VIT_00s1211g00020.t01 | 473 | tRNA (guanine(9)-N1)-methyltransferase-like | 0.5 | 0.0713828 | 28 | 5.3817964 | -6.236368 | 1.02E-08 | 2.76E-09 | down |
| VIT_07s0031g02860.t01 | 469 | elongation factor 2 | 0.5 | 0.0719916 | 26 | 5.040004 | -6.129453 | 3.43E-08 | 9.69E-09 | down |
| VIT_04s0008g03070.t01 | 311 | endonuclease V | 0.5 | 0.1085661 | 26 | 7.6005204 | -6.129453 | 3.43E-08 | 9.69E-09 | down |
| VIT_00s0365g00010.t01 | 69 | Putative uncharacterized protein [Source:UniProtKB/TrEMBL;Acc:F6GZA2] | 0.5 | 0.489334 | 24 | 31.622232 | -6.013976 | 1.17E-07 | 3.46E-08 | down |
| VIT_18s0122g00250.t01 | 1781 | laccase 9 | 3 | 0.1137475 | 132 | 6.7381455 | -5.888445 | 2.90E-37 | 2.48E-38 | down |
| VIT_13s0064g01840.t01 | 132 | disease resistance protein | 0.5 | 0.2557882 | 18 | 12.397352 | -5.598938 | 5.02E-06 | 1.76E-06 | down |
| VIT_18s0122g00650.t01 | 255 | bifunctional dihydroflavonol 4-reductase/flavanone 4-reductase-like | 0.5 | 0.132408 | 18 | 6.417453 | -5.598938 | 5.02E-06 | 1.76E-06 | down |
| VIT_19s0085g00320.t01 | 270 | Putative uncharacterized protein | 0.5 | 0.125052 | 17 | 5.7242096 | -5.516476 | 9.52E-06 | 3.45E-06 | down |
| VIT_18s0001g07510.t01 | 590 | mechanosensitive ion channel protein 3 | 1 | 0.1144544 | 34 | 5.2391071 | -5.516476 | 2.17E-10 | 5.20E-11 | down |
| VIT_18s0072g00870.t01 | 227 | protein RRP45A-like | 1 | 0.2974806 | 33 | 13.216561 | -5.473408 | 4.15E-10 | 1.01E-10 | down |
| VIT_18s0072g00030.t01 | 288 | FAR1-RELATED SEQUENCE 9-like | 0.5 | 0.1172363 | 16 | 5.0507732 | -5.429013 | 1.82E-05 | 6.80E-06 | down |
| VIT_05s0077g00640.t01 | 279 | Putative uncharacterized protein | 0.5 | 0.1210181 | 16 | 5.2137014 | -5.429013 | 1.82E-05 | 6.80E-06 | down |
| VIT_09s0018g00720.t01 | 379 | LRR receptor-like serine/threonine-protein kinase | 1 | 0.1781744 | 32 | 7.6761091 | -5.429013 | 7.88E-10 | 1.97E-10 | down |
| VIT_14s0030g00660.t01 | 2031 | bifunctional 3-dehydroquinate dehydratase/shikimate dehydrogenase | 7 | 0.2327408 | 217 | 9.7135993 | -5.38321 | 2.38E-60 | 1.29E-61 | down |
| VIT_17s0000g03470.t01 | 156 | cysteine desulfurase 1 | 0.5 | 0.2164362 | 15 | 8.7417228 | -5.335904 | 3.48E-05 | 1.35E-05 | down |
| VIT_15s0046g03640.t01 | 114 | Putative uncharacterized protein | 1 | 0.5923516 | 28 | 22.329734 | -5.236368 | 1.07E-08 | 2.91E-09 | down |
| VIT_09s0018g02000.t01 | 194 | Putative uncharacterized protein | 0.5 | 0.1740415 | 14 | 6.5607982 | -5.236368 | 6.73E-05 | 2.70E-05 | down |
| VIT_14s0006g03160.t01 | 246 | HEAT repeat-containing protein 5B | 0.5 | 0.1372522 | 14 | 5.1739628 | -5.236368 | 6.73E-05 | 2.70E-05 | down |
| VIT_19s0027g01930.t01 | 219 | peroxiredoxin | 0.5 | 0.1541737 | 13 | 5.3967166 | -5.129453 | 0.00013004 | 5.43E-05 | down |
| VIT_11s0052g01690.t01 | 354 | putative lipase ROG1 | 1 | 0.1907573 | 26 | 6.6772934 | -5.129453 | 4.01E-08 | 1.14E-08 | down |
| VIT_10s0003g03620.t01 | 208 | beta-amyrin synthase | 0.5 | 0.1623271 | 13 | 5.6821199 | -5.129453 | 0.00013004 | 5.43E-05 | down |
| VIT_05s0077g00380.t01 | 138 | Putative uncharacterized protein | 0.5 | 0.244667 | 12 | 7.9055581 | -5.013976 | 0.00025322 | 0.00010999 | down |
| VIT_08s0007g06840.t01 | 45 | Putative uncharacterized protein | 0.5 | 0.7503121 | 12 | 24.243711 | -5.013976 | 0.00025322 | 0.00010999 | down |
| VIT_05s0165g00250.t01 | 1584 | retrotransposon V12 | 6 | 0.2557882 | 136 | 7.8057404 | -4.931514 | 1.64E-37 | 1.40E-38 | down |
| VIT_00s1351g00020.t01 | 183 | Putative uncharacterized protein | 0.5 | 0.184503 | 11 | 5.464771 | -4.888445 | 0.00049554 | 0.00022473 | down |
| VIT_15s0046g02670.t01 | 126 | Putative uncharacterized protein | 0.5 | 0.2679686 | 11 | 7.9369293 | -4.888445 | 0.00049554 | 0.00022473 | down |
| VIT_01s0026g00480.t01 | 225 | WAT1-related protein | 1 | 0.3001248 | 21 | 8.485299 | -4.821331 | 1.12E-06 | 3.65E-07 | down |
| VIT_14s0036g01260.t01 | 267 | ATP synthase subunit a | 2 | 0.5058284 | 41 | 13.960564 | -4.786565 | 5.91E-12 | 1.26E-12 | down |
| VIT_18s0001g05330.t01 | 66 | Putative uncharacterized protein | 0.5 | 0.5115764 | 10 | 13.774836 | -4.750942 | 0.00096872 | 0.00046307 | down |
| VIT_17s0053g00630.t01 | 1104 | rhodanese-like domain-containing protein 8 | 4 | 0.244667 | 74 | 6.0938677 | -4.638467 | 1.77E-20 | 2.49E-21 | down |
| VIT_04s0023g00320.t01 | 1207 | auxin efflux carrier component 8 | 4 | 0.2237882 | 70 | 5.2725553 | -4.558297 | 2.67E-19 | 3.94E-20 | down |
| VIT_18s0001g03170.t01 | 1365 | WAT1-related protein | 5 | 0.2473556 | 83 | 5.528099 | -4.482125 | 1.58E-22 | 2.06E-23 | down |
| VIT_10s0003g02100.t01 | 783 | GDSL esterase/lipase | 39 | 3.3634679 | 609 | 70.710825 | -4.39391 | 1.65E-157 | 3.48E-159 | down |
| VIT_11s0052g00790.t01 | 1443 | serine carboxypeptidase-like 13 | 13 | 0.6083611 | 180 | 11.340613 | -4.220427 | 2.79E-46 | 1.97E-47 | down |
| VIT_07s0031g00720.t01 | 648 | ethylene-responsive transcription factor ERF109-like | 19 | 1.9799902 | 231 | 32.409128 | -4.032835 | 1.28E-57 | 7.30E-59 | down |
| VIT_09s0018g00080.t01 | 288 | Putative uncharacterized protein | 2 | 0.468945 | 23 | 7.2604865 | -3.952575 | 1.19E-06 | 3.91E-07 | down |
| VIT_18s0001g08610.t01 | 2778 | AP2-like ethylene-responsive transcription factor ANT | 17 | 0.4132388 | 187 | 6.1198353 | -3.888445 | 8.40E-46 | 6.01E-47 | down |
| VIT_18s0041g01320.t01 | 237 | TMV resistance protein N-like | 3 | 0.8547859 | 32 | 12.275297 | -3.844051 | 1.11E-08 | 3.03E-09 | down |
| VIT_11s0103g00390.t01 | 278 | Putative uncharacterized protein | 2 | 0.4858136 | 20 | 6.5405696 | -3.750942 | 9.23E-06 | 3.34E-06 | down |
| VIT_18s0001g09350.t01 | 974 | glycine-rich cell wall structural protein 2-like | 10 | 0.6933068 | 98 | 9.1473962 | -3.721795 | 8.40E-24 | 1.04E-24 | down |
| VIT_03s0091g01200.t01 | 1675 | serine carboxypeptidase-like 18 | 14 | 0.5644139 | 127 | 6.8931746 | -3.610343 | 6.86E-30 | 7.04E-31 | down |
| VIT_15s0021g00250.t01 | 553 | Putative uncharacterized protein | 5 | 0.6105614 | 45 | 7.3980584 | -3.598938 | 2.88E-11 | 6.44E-12 | down |
| VIT_03s0091g00390.t01 | 477 | snakin-1 | 6 | 0.8494099 | 54 | 10.292142 | -3.598938 | 2.69E-13 | 5.27E-14 | down |
| VIT_16s0039g01450.t01 | 1437 | growth-regulating factor 4-like | 12 | 0.5639089 | 104 | 6.5797129 | -3.544491 | 2.45E-24 | 2.97E-25 | down |
| VIT_11s0052g00750.t01 | 1649 | serine carboxypeptidase-like 18 | 37 | 1.5151845 | 319 | 17.58735 | -3.536973 | 1.96E-72 | 9.07E-74 | down |
| VIT_12s0142g00330.t01 | 1774 | copper-transporting ATPase PAA1 | 26 | 0.9897014 | 220 | 11.274556 | -3.509933 | 5.79E-50 | 3.80E-51 | down |
| VIT_00s1881g00010.t01 | 2590 | Putative uncharacterized protein | 43 | 1.1211227 | 347 | 12.180359 | -3.441541 | 4.88E-77 | 2.09E-78 | down |
| VIT_00s0279g00120.t01 | 546 | Putative uncharacterized protein | 6 | 0.7420669 | 47 | 7.8259233 | -3.39864 | 2.99E-11 | 6.70E-12 | down |
| VIT_07s0031g00710.t01 | 604 | ethylene-responsive transcription factor ERF109-like | 7 | 0.7826103 | 54 | 8.1280655 | -3.376546 | 1.07E-12 | 2.18E-13 | down |
| VIT_07s0130g00030.t01 | 565 | defensin-like protein 1 | 71 | 8.4858303 | 538 | 86.569359 | -3.350729 | 2.20E-116 | 6.35E-118 | down |
| VIT_08s0032g00960.t01 | 1424 | iron-sulfur cluster co-chaperone protein HscB | 11 | 0.5216355 | 82 | 5.2352115 | -3.327134 | 1.68E-18 | 2.56E-19 | down |
| VIT_00s0261g00040.t01 | 1616 | Putative uncharacterized protein | 18 | 0.7521693 | 134 | 7.5386541 | -3.325178 | 1.53E-29 | 1.58E-30 | down |
| VIT_10s0116g00790.t01 | 1432 | RNA pseudourine synthase 6 | 14 | 0.6601908 | 102 | 6.475712 | -3.294084 | 1.44E-22 | 1.87E-23 | down |
| VIT_15s0107g00560.t01 | 534 | Putative uncharacterized protein | 15 | 1.8968564 | 109 | 18.557335 | -3.290307 | 5.09E-24 | 6.26E-25 | down |
| VIT_14s0066g02230.t01 | 1151 | proline-, glutamic acid- and leucine-rich protein 1 | 13 | 0.7626978 | 92 | 7.2667945 | -3.252136 | 3.01E-20 | 4.28E-21 | down |
| VIT_18s0001g11960.t01 | 84 | Putative uncharacterized protein | 2 | 1.6078116 | 14 | 15.15232 | -3.236368 | 0.00053303 | 0.00024349 | down |
| VIT_10s0003g05190.t01 | 600 | putative wall-associated receptor kinase-like 11 | 5 | 0.5627341 | 34 | 5.1517887 | -3.194548 | 4.58E-08 | 1.31E-08 | down |
| VIT_00s0239g00110.t01 | 486 | protein MEI2-like 4-like | 6 | 0.8336801 | 40 | 7.482627 | -3.165979 | 3.13E-09 | 8.18E-10 | down |
| VIT_09s0002g08840.t01 | 699 | Putative uncharacterized protein | 9 | 0.8694603 | 57 | 7.4135813 | -3.091979 | 1.99E-12 | 4.10E-13 | down |
| VIT_17s0000g01010.t01 | 1430 | monoglyceride lipase-like | 15 | 0.7083366 | 91 | 5.7854311 | -3.029918 | 7.47E-19 | 1.12E-19 | down |
| VIT_03s0017g02210.t01 | 711 | Putative uncharacterized protein | 8 | 0.7598097 | 47 | 6.0097808 | -2.983602 | 3.89E-10 | 9.48E-11 | down |
| VIT_06s0009g00480.t01 | 1494 | aluminum-activated malate transporter 2-like | 27 | 1.2203871 | 158 | 9.6147249 | -2.977907 | 1.92E-31 | 1.89E-32 | down |
| VIT_10s0003g05480.t01 | 1740 | patellin-4-like | 79 | 3.0659304 | 449 | 23.459971 | -2.935804 | 2.50E-86 | 9.70E-88 | down |
| VIT_02s0012g03100.t01 | 93 | Putative uncharacterized protein | 3 | 2.1783254 | 17 | 16.618673 | -2.931514 | 0.0002592 | 0.00011298 | down |
| VIT_05s0020g03730.t01 | 759 | non-specific lipid-transfer protein | 8 | 0.7117585 | 43 | 5.1505909 | -2.855278 | 5.12E-09 | 1.36E-09 | down |
| VIT_19s0085g00510.t01 | 1948 | RNA helicase 36 | 22 | 0.7626375 | 118 | 5.5071059 | -2.852225 | 8.42E-23 | 1.08E-23 | down |
| VIT_18s0001g00040.t01 | 1029 | Putative uncharacterized protein | 12 | 0.7874996 | 64 | 5.6545099 | -2.844051 | 7.96E-13 | 1.60E-13 | down |
| VIT_18s0001g10500.t01 | 1509 | hydroxylase 1 (Hyd1) | 18 | 0.805504 | 96 | 5.783788 | -2.844051 | 1.07E-18 | 1.61E-19 | down |
| VIT_10s0116g01600.t01 | 372 | L-ascorbate oxidase | 7 | 1.2706898 | 37 | 9.0425133 | -2.831112 | 7.49E-08 | 2.18E-08 | down |
| VIT_18s0041g00370.t01 | 506 | cysteine proteinase inhibitor 8-like | 15 | 2.0018207 | 78 | 14.014398 | -2.807525 | 3.28E-15 | 5.85E-16 | down |
| VIT_13s0084g00440.t01 | 382 | THO complex subunit 1-like | 5 | 0.8838755 | 26 | 6.1878583 | -2.807525 | 8.30E-06 | 2.98E-06 | down |
| VIT_00s0199g00120.t01 | 887 | Putative uncharacterized protein | 15 | 1.1419631 | 78 | 7.994685 | -2.807525 | 3.28E-15 | 5.85E-16 | down |
| VIT_18s0001g02400.t01 | 2337 | laccase 9 | 138 | 3.9875378 | 709 | 27.581501 | -2.790131 | 4.71E-129 | 1.21E-130 | down |
| VIT_10s0116g00050.t01 | 810 | STRUBBELIG-RECEPTOR FAMILY 8-like | 15 | 1.2505201 | 77 | 8.6424341 | -2.788909 | 6.18E-15 | 1.12E-15 | down |
| VIT_00s2580g00010.t01 | 875 | U4/U6.U5 tri-snRNP-associated protein 2-like | 10 | 0.7717496 | 51 | 5.2989826 | -2.779511 | 3.12E-10 | 7.53E-11 | down |
| VIT_01s0150g00150.t01 | 1903 | glycyl-tRNA synthetase 1 | 22 | 0.7806715 | 110 | 5.2551397 | -2.750942 | 1.38E-20 | 1.92E-21 | down |
| VIT_19s0014g04990.t01 | 755 | dihydroflavonol-4-reductase | 12 | 1.0732941 | 60 | 7.2249471 | -2.750942 | 1.00E-11 | 2.17E-12 | down |
| VIT_07s0104g01420.t01 | 749 | glutaredoxin | 52 | 4.6881983 | 257 | 31.194762 | -2.734198 | 2.04E-46 | 1.44E-47 | down |
| VIT_14s0006g00520.t01 | 1792 | glucan endo-1,3-beta-glucosidase 2-like | 45 | 1.6957388 | 220 | 11.161307 | -2.71852 | 1.20E-39 | 9.78E-41 | down |
| VIT_16s0022g02310.t01 | 1321 | Gibberellin 20 oxidase 1 | 32 | 1.6358053 | 156 | 10.736239 | -2.714416 | 2.30E-28 | 2.46E-29 | down |
| VIT_13s0019g02280.t01 | 1745 | X-linked retinitis pigmentosa GTPase regulator-like | 35 | 1.3544315 | 168 | 8.7527439 | -2.692048 | 3.27E-30 | 3.34E-31 | down |
| VIT_19s0027g00800.t01 | 788 | chloroplast DNA | 50 | 4.2847771 | 239 | 27.574145 | -2.686024 | 1.90E-42 | 1.45E-43 | down |
| VIT_04s0008g01970.t01 | 3124 | LRR receptor-like serine/threonine-protein kinase | 44 | 0.9510998 | 210 | 6.1113709 | -2.683827 | 2.18E-37 | 1.86E-38 | down |
| VIT_00s0965g00010.t01 | 489 | magnesium-chelatase D subunit | 25 | 3.4523562 | 119 | 22.124246 | -2.679975 | 1.31E-21 | 1.76E-22 | down |
| VIT_10s0116g01580.t01 | 772 | glycine-rich cell wall structural protein 1 | 503 | 43.998222 | 2392 | 281.69183 | -2.678601 | 0 | 0 | down |
| VIT_12s0142g00240.t01 | 1138 | protodermal factor 1 | 151 | 8.9602294 | 716 | 57.200672 | -2.674425 | 1.12E-124 | 3.01E-126 | down |
| VIT_09s0002g03930.t01 | 2138 | ruBisCO large subunit-binding protein subunit beta | 227 | 7.1697267 | 1069 | 45.456959 | -2.664511 | 5.16E-185 | 9.39E-187 | down |
| VIT_09s0054g00350.t01 | 1207 | Putative uncharacterized protein | 24 | 1.3427291 | 113 | 8.5114107 | -2.66423 | 1.89E-20 | 2.66E-21 | down |
| VIT_18s0001g14760.t01 | 1026 | GDSL esterase/lipase EXL3-like | 32 | 2.1061391 | 150 | 13.291508 | -2.657832 | 9.92E-27 | 1.12E-27 | down |

| | | | | | | | | | | |
|---|---|---|---|---|---|---|---|---|---|---|
| VIT_13s0101g00230.t01 | 252 | Putative uncharacterized protein | 73 | 19.561708 | 342 | 123.38317 | -2.657041 | 1.28E-59 | 7.06E-61 | down |
| VIT_16s0039g02230.t01 | 1435 | UDP glucose:flavonoid 3-o-glucosyltransferase (UFGT) | 33 | 1.5529107 | 153 | 9.6932609 | -2.642007 | 4.48E-27 | 4.99E-28 | down |
| VIT_12s0059g01730.t01 | 369 | protein EPIDERMAL PATTERNING FACTOR 1 | 7 | 1.2810206 | 32 | 7.8841338 | -2.621659 | 1.73E-06 | 5.78E-07 | down |
| VIT_19s0014g01780.t01 | 1549 | basic leucine zipper 34-like | 25 | 1.0898658 | 114 | 6.6908887 | -2.618047 | 2.96E-20 | 4.20E-21 | down |
| VIT_05s0049g02320.t01 | 1074 | Putative uncharacterized protein | 25 | 1.5718828 | 114 | 9.6500806 | -2.618047 | 2.96E-20 | 4.20E-21 | down |
| VIT_19s0014g01280.t01 | 1705 | Putative uncharacterized protein | 27 | 1.0693597 | 123 | 6.5585993 | -2.61664 | 9.35E-22 | 1.25E-22 | down |
| VIT_14s0066g00660.t01 | 678 | mitogen-activated protein kinase kinase 3 | 9 | 0.8963905 | 41 | 5.4977443 | -2.61664 | 5.51E-08 | 1.59E-08 | down |
| VIT_10s0116g01610.t01 | 1800 | L-ascorbate oxidase | 74 | 2.7761547 | 335 | 16.92009 | -2.607577 | 2.96E-57 | 1.69E-58 | down |
| VIT_00s0313g00070.t01 | 926 | MADS-box protein SVP-like | 19 | 1.3855655 | 86 | 8.4434092 | -2.607351 | 1.76E-15 | 3.09E-16 | down |
| VIT_11s0052g01270.t01 | 1286 | probable xyloglucan endotransglucosylase/hydrolase protein 23-like | 39 | 2.0478969 | 176 | 12.44234 | -2.603043 | 1.66E-30 | 1.67E-31 | down |
| VIT_12s0059g01600.t01 | 1697 | lipid-A-disaccharide synthase-like | 22 | 0.8754378 | 99 | 5.3037583 | -2.598938 | 1.34E-17 | 2.13E-18 | down |
| VIT_06s0061g01570.t01 | 297 | peptidyl-prolyl cis-trans isomerase CYP71 | 4 | 0.9094692 | 18 | 5.5099344 | -2.598938 | 0.00042529 | 0.00019068 | down |
| VIT_10s0042g00100.t01 | 1255 | Fe Superoxide dismutase | 77 | 4.1431575 | 343 | 24.847389 | -2.584292 | 4.93E-58 | 2.78E-59 | down |
| VIT_15s0048g02600.t01 | 2112 | granule-bound starch synthase 1 | 98 | 3.1334055 | 436 | 18.768214 | -2.582488 | 1.86E-73 | 8.46E-75 | down |
| VIT_17s0000g03840.t01 | 679 | Putative uncharacterized protein | 9 | 0.8950704 | 40 | 5.3557536 | -2.581017 | 1.02E-07 | 3.01E-08 | down |
| VIT_19s0014g01110.t01 | 529 | epidermis-specific secreted glycoprotein EP1-like | 7 | 0.8935664 | 31 | 5.3276587 | -2.575855 | 3.21E-06 | 1.10E-06 | down |
| VIT_05s0020g01760.t01 | 355 | glutaredoxin | 12 | 2.2826395 | 53 | 13.573064 | -2.571971 | 7.80E-10 | 1.95E-10 | down |
| VIT_06s0061g00070.t01 | 887 | ubiquitin-conjugating enzyme E2 19-like | 25 | 1.9032719 | 110 | 11.274556 | -2.566517 | 3.52E-19 | 5.21E-20 | down |
| VIT_02s0241g00050.t01 | 2510 | Putative uncharacterized protein | 45 | 1.2106629 | 196 | 7.0992541 | -2.55187 | 3.81E-33 | 3.61E-34 | down |
| VIT_17s0000g04490.t01 | 1405 | calcium sensing receptor, chloroplastic-like | 740 | 35.566394 | 3222 | 208.48729 | -2.551373 | 0 | 0 | down |
| VIT_06s0004g01730.t01 | 1576 | Putative uncharacterized protein | 23 | 0.9854987 | 100 | 5.7686496 | -2.549308 | 2.06E-17 | 3.30E-18 | down |
| VIT_10s0003g05670.t01 | 1952 | pentatricopeptide repeat-containing protein | 32 | 1.1070178 | 139 | 6.4738906 | -2.547955 | 9.08E-24 | 1.12E-24 | down |
| VIT_13s0047g01150.t01 | 1050 | Putative uncharacterized protein | 30 | 1.9293739 | 130 | 11.256009 | -2.544491 | 2.87E-22 | 3.77E-23 | down |
| VIT_08s0040g00800.t01 | 1444 | WAT1-related protein | 51 | 2.3849947 | 220 | 13.851151 | -2.537948 | 7.36E-37 | 6.38E-38 | down |
| VIT_11s0065g00180.t01 | 1248 | chloroplast envelope membrane protein-like | 45 | 2.434907 | 194 | 14.132452 | -2.537073 | 1.31E-32 | 1.25E-33 | down |
| VIT_18s0072g00880.t01 | 1496 | exosome complex component rrp45-like | 29 | 1.3090338 | 125 | 7.5964169 | -2.536817 | 2.20E-21 | 2.98E-22 | down |
| VIT_12s0134g00650.t01 | 1309 | malonyl-coenzyme A:anthocyanin 3-O-glucoside-6"-O-malonyltransferase-like | 62 | 3.1984273 | 266 | 18.474486 | -2.5301 | 3.33E-44 | 2.46E-45 | down |
| VIT_13s0101g00210.t01 | 869 | Putative uncharacterized protein | 2235 | 173.67695 | 9503 | 994.19443 | -2.517122 | 0 | 0 | down |
| VIT_10s0042g01360.t01 | 762 | transmembrane protein 56-like | 14 | 1.2406735 | 59 | 7.0392666 | -2.504302 | 1.55E-10 | 3.68E-11 | down |
| VIT_19s0135g00180.t01 | 582 | cytochrome P450 CYP72A219-like | 18 | 2.0884975 | 75 | 11.715711 | -2.487907 | 5.38E-13 | 1.07E-13 | down |
| VIT_14s0066g01280.t01 | 2306 | Putative uncharacterized protein | 37 | 1.0834949 | 154 | 6.0714412 | -2.486347 | 1.55E-25 | 1.82E-26 | down |
| VIT_14s0108g01300.t01 | 922 | cell division topological specificity factor | 31 | 2.2704671 | 129 | 12.72006 | -2.486044 | 1.47E-21 | 1.97E-22 | down |
| VIT_04s0044g01110.t01 | 1380 | alcohol dehydrogenase 2 (ADH2) | 45 | 2.2020028 | 187 | 12.319495 | -2.484055 | 9.40E-31 | 9.44E-32 | down |
| VIT_15s0046g03600.t01 | 1001 | (+)-neomenthol dehydrogenase-like | 55 | 3.7103344 | 227 | 20.616842 | -2.474202 | 5.97E-37 | 5.16E-38 | down |
| VIT_08s0007g00520.t01 | 655 | UPF0161 protein | 16 | 1.649541 | 66 | 9.1607917 | -2.473408 | 1.67E-11 | 3.68E-12 | down |
| VIT_13s0047g00220.t01 | 539 | Putative uncharacterized protein | 26 | 3.2573845 | 107 | 18.047846 | -2.470041 | 6.06E-18 | 9.50E-19 | down |
| VIT_13s0067g02690.t01 | 1275 | N(2),N(2)-dimethylguanosine tRNA methyltransferase Trm1-like | 26 | 1.3770433 | 107 | 7.6296386 | -2.470041 | 6.06E-18 | 9.50E-19 | down |
| VIT_01s0026g00590.t01 | 1042 | Putative uncharacterized protein | 30 | 1.9441868 | 123 | 10.731681 | -2.464637 | 2.04E-20 | 2.88E-21 | down |
| VIT_16s0050g02270.t01 | 584 | Putative uncharacterized protein | 38 | 4.3939508 | 155 | 24.12955 | -2.45721 | 2.30E-25 | 2.71E-26 | down |
| VIT_05s0020g02890.t01 | 793 | uncharacterized GPI-anchored protein | 43 | 3.6616743 | 175 | 20.06297 | -2.45396 | 1.85E-28 | 1.97E-29 | down |
| VIT_12s0035g00700.t01 | 1318 | protein EI24 homolog-like | 20 | 1.0247054 | 81 | 5.5872741 | -2.446935 | 1.04E-13 | 2.00E-14 | down |
| VIT_10s0116g01670.t01 | 1191 | arogenate dehydratase/prephenate dehydratase 1 | 24 | 1.3607675 | 97 | 7.4044081 | -2.443964 | 3.51E-16 | 5.94E-17 | down |
| VIT_06s0004g01310.t01 | 1878 | enoyl-[acyl-carrier-protein] reductase [NADH] | 322 | 11.578298 | 1301 | 62.981367 | -2.443502 | 2.42E-204 | 3.83E-206 | down |
| VIT_14s0006g02840.t01 | 1264 | Putative uncharacterized protein | 32 | 1.7095718 | 129 | 9.2783982 | -2.440241 | 3.97E-21 | 5.43E-22 | down |
| VIT_15s0046g01010.t01 | 2577 | DEAD-box ATP-dependent RNA helicase 31-like | 206 | 5.3980543 | 826 | 29.140433 | -2.432511 | 2.35E-129 | 5.99E-131 | down |
| VIT_14s0006g01380.t01 | 324 | FAR1-RELATED SEQUENCE 5-like | 5 | 1.0421001 | 20 | 5.6119702 | -2.429013 | 0.0003454 | 0.00015335 | down |
| VIT_00s0227g00020.t01 | 708 | Putative uncharacterized protein | 18 | 1.7168158 | 72 | 9.2454831 | -2.429013 | 3.26E-12 | 6.84E-13 | down |
| VIT_11s0037g00800.t01 | 1418 | serine carboxypeptidase-like 29 | 83 | 3.9526313 | 327 | 20.965339 | -2.407121 | 3.66E-51 | 2.35E-52 | down |
| VIT_01s0010g03290.t01 | 698 | probable beta-1,3-galactosyltransferase 11 | 16 | 1.5479218 | 63 | 8.2056974 | -2.406293 | 1.01E-10 | 2.35E-11 | down |
| VIT_08s0105g00550.t01 | 1146 | protein SCO1 homolog 1 | 20 | 1.1785006 | 78 | 6.1878583 | -2.392488 | 6.32E-13 | 1.26E-13 | down |
| VIT_18s0001g09920.t01 | 1986 | cyclin-D3-1-like | 150 | 5.1003086 | 585 | 26.779779 | -2.392488 | 3.91E-90 | 1.45E-91 | down |
| VIT_11s0016g03700.t01 | 2541 | pentatricopeptide repeat-containing protein | 66 | 1.7539763 | 257 | 9.1951503 | -2.390244 | 4.18E-40 | 3.36E-41 | down |
| VIT_02s0012g02600.t01 | 517 | Putative uncharacterized protein | 9 | 1.1755373 | 35 | 6.154714 | -2.388372 | 2.11E-06 | 7.09E-07 | down |
| VIT_16s0098g01080.t01 | 1869 | growth-regulating factor 4 | 103 | 3.7214515 | 400 | 19.457232 | -2.386369 | 9.64E-62 | 5.13E-63 | down |
| VIT_05s0062g00840.t01 | 850 | ras-related protein RABA5c-like | 25 | 1.9861202 | 97 | 10.374882 | -2.38507 | 9.27E-16 | 1.60E-16 | down |
| VIT_14s0066g02120.t01 | 1271 | Putative uncharacterized protein | 234 | 12.432394 | 902 | 64.519554 | -2.375632 | 2.35E-137 | 5.58E-139 | down |
| VIT_02s0025g01260.t01 | 1334 | anthocyanidin reductase | 31 | 1.5692434 | 118 | 8.0418608 | -2.35746 | 1.08E-18 | 1.63E-19 | down |
| VIT_04s0023g03360.t01 | 1282 | glutamic acid-rich protein | 46 | 2.4230047 | 175 | 12.410246 | -2.356663 | 3.39E-27 | 3.76E-28 | down |
| VIT_00s0441g00020.t01 | 1664 | squalene epoxidase | 29 | 1.1768717 | 110 | 6.0099345 | -2.352392 | 1.86E-17 | 2.96E-18 | down |
| VIT_08s0007g08290.t01 | 2332 | GTP-binding protein TypA/BipA homolog-like | 226 | 6.5443171 | 857 | 33.410475 | -2.351986 | 5.01E-129 | 1.28E-130 | down |
| VIT_09s0002g02210.t01 | 1904 | protein HOTHEAD-like | 33 | 1.1703923 | 125 | 5.9686133 | -2.350404 | 1.13E-19 | 1.64E-20 | down |
| VIT_02s0025g02640.t01 | 1158 | beta-carotene isomerase D27 | 222 | 12.945799 | 836 | 65.633882 | -2.341957 | 2.96E-125 | 7.86E-127 | down |
| VIT_07s0141g00380.t01 | 1889 | U3 small nucleolar RNA-associated protein 18 homolog | 41 | 1.4656705 | 154 | 7.4117222 | -2.338248 | 7.61E-24 | 9.39E-25 | down |
| VIT_13s0064g00380.t01 | 2265 | Putative uncharacterized protein | 57 | 1.6993823 | 214 | 8.5896593 | -2.33759 | 1.01E-32 | 9.67E-34 | down |
| VIT_12s0028g02120.t01 | 285 | serine/threonine-protein kinase EDR1 | 15 | 3.5541098 | 56 | 17.863787 | -2.329478 | 2.47E-09 | 6.39E-10 | down |
| VIT_04s0008g03560.t01 | 1463 | probable lactoylglutathione lyase | 203 | 9.3699259 | 757 | 47.041583 | -2.327827 | 1.08E-112 | 3.21E-114 | down |
| VIT_07s0129g00790.t01 | 525 | Vitis rotundifolia chloroplast | 5374 | 691.23036 | 20011 | 3465.2922 | -2.325739 | 0 | 0 | down |
| VIT_18s0001g14320.t01 | 928 | nucleolar protein 58 | 17 | 1.2370447 | 63 | 6.1719578 | -2.318831 | 2.60E-10 | 6.25E-11 | down |
| VIT_09s0002g02110.t01 | 1053 | Putative uncharacterized protein | 83 | 5.3227267 | 306 | 26.419429 | -2.311362 | 9.58E-46 | 6.86E-47 | down |
| VIT_08s0032g00330.t01 | 1062 | nucleolar GTP-binding protein 1-like | 28 | 1.7804015 | 103 | 8.8174515 | -2.308159 | 4.50E-16 | 7.67E-17 | down |
| VIT_15s0046g00290.t01 | 2445 | auxin response factor 18 | 157 | 4.3361593 | 576 | 21.417757 | -2.304318 | 6.22E-85 | 2.44E-86 | down |
| VIT_02s0025g02960.t01 | 1208 | naringenin,2-oxoglutarate 3-dioxygenase-like | 30 | 1.677022 | 110 | 8.2785852 | -2.303483 | 4.71E-17 | 7.68E-18 | down |
| VIT_05s0062g00480.t01 | 1081 | xyloglucan endotransglycosylase XET1-like | 18 | 1.124427 | 66 | 5.550711 | -2.303483 | 1.13E-10 | 2.65E-11 | down |
| VIT_05s0020g02390.t01 | 2806 | AP-5 complex subunit mu | 47 | 1.1310834 | 172 | 5.5727704 | -2.300689 | 5.07E-26 | 5.83E-27 | down |
| VIT_11s0052g01680.t01 | 261 | psbA; photosystem II P680 reaction center D1 protein | 389 | 100.64531 | 1422 | 495.3241 | -2.299093 | 7.23E-208 | 1.13E-209 | down |
| VIT_07s0031g00240.t01 | 1086 | OTU domain-containing protein 3-like | 25 | 1.554514 | 91 | 7.618017 | -2.292952 | 3.19E-14 | 5.98E-15 | down |
| VIT_15s0048g01130.t01 | 2490 | subtilisin-like protease | 100 | 2.7119713 | 364 | 13.290227 | -2.292952 | 1.04E-53 | 6.32E-55 | down |
| VIT_18s0001g08650.t01 | 2032 | growth-regulating factor 6-like | 43 | 1.42899 | 156 | 6.9796118 | -2.288151 | 1.50E-23 | 1.87E-24 | down |
| VIT_13s0047g00510.t01 | 2923 | DNA gyrase subunit A | 94 | 2.1716182 | 341 | 10.606105 | -2.288053 | 2.96E-50 | 1.93E-51 | down |
| VIT_01s0011g04110.t01 | 192 | Putative uncharacterized protein | 8 | 2.8136703 | 29 | 13.73179 | -2.286994 | 2.84E-05 | 1.09E-05 | down |
| VIT_02s0154g00600.t01 | 1051 | probable pectinesterase 68-like | 46 | 2.9555585 | 166 | 14.359382 | -2.280491 | 6.78E-25 | 8.08E-26 | down |
| VIT_02s0154g00080.t01 | 2127 | monocopper oxidase-like protein SKU5 | 312 | 9.9053893 | 1125 | 48.08564 | -2.279321 | 6.42E-163 | 1.31E-164 | down |
| VIT_08s0007g08540.t01 | 4376 | magnesium-chelatase H subunit | 3689 | 56.926671 | 13233 | 274.92319 | -2.271852 | 0 | 0 | down |
| VIT_19s0027g01660.t01 | 1917 | peptidyl-prolyl cis-trans isomerase-like 4-like | 36 | 1.2681331 | 129 | 6.117838 | -2.270316 | 1.74E-19 | 2.54E-20 | down |
| VIT_03s0038g04680.t01 | 930 | isoflavone reductase homolog-like | 18 | 1.3069952 | 64 | 6.2564416 | -2.259088 | 3.62E-10 | 8.80E-11 | down |
| VIT_08s0032g00320.t01 | 293 | nucleolar GTP-binding protein 1-like | 9 | 2.0742416 | 32 | 9.9291651 | -2.259088 | 1.23E-05 | 4.51E-06 | down |
| VIT_16s0013g00330.t01 | 715 | Putative uncharacterized protein | 4030 | 380.61285 | 14327 | 1821.7115 | -2.258898 | 0 | 0 | down |
| VIT_04s0043g00700.t01 | 2869 | Putative uncharacterized protein | 137 | 3.2245897 | 486 | 15.400545 | -2.255794 | 5.18E-70 | 2.48E-71 | down |
| VIT_18s0001g04420.t01 | 1184 | phosphopantothenate--cysteine ligase 1-like | 24 | 1.3688126 | 85 | 6.5267593 | -2.253442 | 4.25E-13 | 8.41E-14 | down |
| VIT_02s0033g01060.t01 | 1604 | benzyl alcohol O-benzoyltransferase-like | 106 | 4.4625793 | 375 | 21.254812 | -2.25184 | 4.02E-54 | 2.41E-55 | down |
| VIT_18s0072g01190.t01 | 255 | CDK5 regulatory subunit-associated protein 1-like 1-like | 8 | 2.1185282 | 28 | 9.9827047 | -2.236368 | 5.09E-05 | 2.01E-05 | down |
| VIT_18s0001g10220.t01 | 457 | polyadenylate-binding protein 2-like | 12 | 1.7731664 | 42 | 8.3553272 | -2.236368 | 5.58E-07 | 1.77E-07 | down |
| VIT_02s0025g02100.t01 | 510 | Putative uncharacterized protein | 18 | 2.3833442 | 63 | 11.230543 | -2.236368 | 6.44E-10 | 1.60E-10 | down |
| VIT_00s0207g00040.t01 | 318 | phosphoglycolate phosphatase 1B | 71 | 15.077026 | 248 | 70.90142 | -2.233463 | 8.62E-36 | 7.66E-37 | down |
| VIT_11s0052g00860.t01 | 878 | pseudouridine-5'-monophosphatase-like | 56 | 4.3070306 | 193 | 19.984494 | -2.214116 | 8.63E-28 | 9.37E-29 | down |
| VIT_18s0072g00920.t01 | 1876 | caffeic acid 3-O-methyltransferase | 47 | 1.6918017 | 161 | 7.8023138 | -2.205342 | 2.99E-23 | 3.78E-24 | down |
| VIT_00s0220g00180.t01 | 914 | beta-hexosaminidase subunit B2-like | 59 | 4.359034 | 202 | 20.092573 | -2.204582 | 6.86E-29 | 7.23E-30 | down |

| | | | | | | | | | | |
|---|---|---|---|---|---|---|---|---|---|---|
| VIT_03s0038g00150.t01 | 2889 | beta-galactosidase 3-like | 69 | 1.6128203 | 236 | 7.4266821 | -2.203132 | 1.49E-33 | 1.39E-34 | down |
| VIT_03s0180g00150.t01 | 2271 | receptor protein kinase-like protein | 97 | 2.8842908 | 330 | 13.210741 | -2.195423 | 2.56E-46 | 1.81E-47 | down |
| VIT_10s0003g03440.t01 | 913 | Putative uncharacterized protein | 20 | 1.4792571 | 68 | 6.7712447 | -2.194548 | 2.13E-10 | 5.10E-11 | down |
| VIT_13s0019g00810.t01 | 2007 | methionine--tRNA ligase | 127 | 4.2730777 | 431 | 19.523617 | -2.191873 | 4.57E-60 | 2.50E-61 | down |
| VIT_18s0001g02700.t01 | 2176 | chlorophyllide a oxygenase | 433 | 13.437344 | 1468 | 61.33347 | -2.190427 | 2.79E-202 | 4.47E-204 | down |
| VIT_14s0030g00190.t01 | 742 | FANTASTIC FOUR 1-like | 26 | 2.3662133 | 88 | 10.782244 | -2.188005 | 4.44E-13 | 8.79E-14 | down |
| VIT_15s0045g00770.t01 | 390 | 30S ribosomal protein S15 | 8 | 1.3851915 | 27 | 6.2940405 | -2.183901 | 9.04E-05 | 3.69E-05 | down |
| VIT_14s0108g00700.t01 | 909 | aquaporin NIP2-1-like | 22 | 1.6343431 | 74 | 7.401133 | -2.179035 | 3.98E-11 | 9.01E-12 | down |
| VIT_17s0000g08480.t01 | 703 | transcription factor WER-like | 33 | 3.1698817 | 111 | 14.354829 | -2.179035 | 3.87E-16 | 6.56E-17 | down |
| VIT_17s0053g00590.t01 | 1562 | Putative uncharacterized protein | 81 | 3.5017766 | 272 | 15.831361 | -2.176626 | 5.86E-38 | 4.93E-39 | down |
| VIT_19s0027g00310.t01 | 1535 | pentatricopeptide repeat-containing protein | 28 | 1.2317827 | 94 | 5.5673669 | -2.176247 | 8.19E-14 | 1.56E-14 | down |
| VIT_18s0089g00360.t01 | 2115 | eukaryotic translation initiation factor 2D-like | 37 | 1.1813424 | 124 | 5.3301777 | -2.173756 | 7.49E-18 | 1.18E-18 | down |
| VIT_08s0058g00540.t01 | 2750 | probably inactive leucine-rich repeat receptor-like protein kinase IMK2-like | 86 | 2.1117874 | 287 | 9.488107 | -2.167656 | 8.49E-40 | 6.89E-41 | down |
| VIT_03s0063g00760.t01 | 393 | disease resistance RPP13-like protein 1 | 9 | 1.5464447 | 30 | 6.9399937 | -2.165979 | 3.87E-05 | 1.51E-05 | down |
| VIT_08s0007g07630.t01 | 1611 | tyrosine-specific transport protein-like | 39 | 1.6347581 | 130 | 7.3363186 | -2.165979 | 1.39E-18 | 2.11E-19 | down |
| VIT_08s0058g01310.t01 | 1246 | elongation factor P-like | 117 | 6.3409198 | 390 | 28.456202 | -2.165979 | 1.16E-53 | 7.09E-55 | down |
| VIT_07s0031g03000.t01 | 583 | ribulose-1,5-bisphosphate carboxylase/oxygenase large subunit (rbcL) gene | 11988 | 1388.5535 | 39927 | 6226.2779 | -2.164787 | 0 | 0 | down |
| VIT_03s0038g00300.t01 | 2700 | minichromosome maintenance complex component 4 (MCM4) | 64 | 1.6006658 | 213 | 7.1720979 | -2.163723 | 9.87E-30 | 1.02E-30 | down |
| VIT_00s0282g00030.t01 | 1639 | trichome birefringence-like 12 | 31 | 1.2772243 | 103 | 5.7133212 | -2.161318 | 6.47E-15 | 1.18E-15 | down |
| VIT_00s0216g00070.t01 | 1789 | Putative uncharacterized protein | 66 | 2.4912542 | 219 | 11.129205 | -2.159406 | 1.81E-30 | 1.83E-31 | down |
| VIT_12s0059g01900.t01 | 841 | Putative uncharacterized protein | 19 | 1.5256048 | 63 | 6.8104362 | -2.158366 | 1.54E-09 | 3.93E-10 | down |
| VIT_03s0091g00820.t01 | 1556 | ribosomal RNA large subunit methyltransferase N-like | 32 | 1.3887524 | 106 | 6.1933646 | -2.156934 | 2.79E-15 | 4.96E-16 | down |
| VIT_01s0011g04710.t01 | 1958 | Putative uncharacterized protein | 100 | 3.4488297 | 331 | 15.369002 | -2.155845 | 1.94E-45 | 1.40E-46 | down |
| VIT_15s0024g00790.t01 | 1151 | tRNA (guanine-N(7)-)-methyltransferase-like | 55 | 3.2267982 | 182 | 14.375615 | -2.155448 | 1.93E-25 | 2.27E-26 | down |
| VIT_03s0063g00110.t01 | 1180 | Putative uncharacterized protein | 73 | 4.177585 | 240 | 18.490966 | -2.14608 | 4.83E-33 | 4.58E-34 | down |
| VIT_18s0001g09190.t01 | 435 | activating signal cointegrator 1 complex subunit 2 | 11 | 1.7076068 | 36 | 7.5239104 | -2.139507 | 7.15E-06 | 2.55E-06 | down |
| VIT_19s0014g03330.t01 | 726 | peptidyl-prolyl cis-trans isomerase CYP37 | 81 | 7.5341253 | 264 | 33.059606 | -2.133558 | 5.48E-36 | 4.85E-37 | down |
| VIT_05s0020g01900.t01 | 769 | cuticle collagen 2 | 31 | 2.7221985 | 101 | 11.94058 | -2.133029 | 2.01E-14 | 3.73E-15 | down |
| VIT_16s0050g00960.t01 | 1957 | prolyl-tRNA synthetase-like | 257 | 8.8680216 | 836 | 38.837013 | -2.130748 | 1.19E-111 | 3.55E-113 | down |
| VIT_10s0003g02820.t01 | 598 | CAS1 domain-containing protein 1-like | 12 | 1.3550787 | 39 | 5.9291685 | -2.129453 | 3.08E-06 | 1.05E-06 | down |
| VIT_00s0287g00030.t01 | 373 | GATA transcription factor 26-like | 8 | 1.4483236 | 26 | 6.3371632 | -2.129453 | 0.00015945 | 6.73E-05 | down |
| VIT_11s0078g00430.t01 | 1087 | galacturonokinase-like | 45 | 2.795551 | 146 | 12.211069 | -2.126985 | 2.69E-20 | 3.82E-21 | down |
| VIT_11s0016g02420.t01 | 1089 | acyl-[acyl-carrier-protein]--UDP-N-acetylglucosamine O-acyltransferase-like | 41 | 2.5423797 | 133 | 11.103353 | -2.126744 | 1.39E-18 | 2.12E-19 | down |
| VIT_18s0001g14650.t01 | 532 | DNA-directed RNA polymerase I subunit RPA12 | 13 | 1.6501224 | 42 | 7.1774145 | -2.120891 | 1.32E-06 | 4.36E-07 | down |
| VIT_07s0005g04900.t01 | 1254 | protein LOW PSII ACCUMULATION 1 | 39 | 2.1001558 | 126 | 9.1348912 | -2.120891 | 1.33E-17 | 2.11E-18 | down |
| VIT_01s0026g01720.t01 | 1019 | single-stranded DNA-binding protein WHY1 | 155 | 10.271691 | 500 | 44.609381 | -2.118673 | 1.13E-66 | 5.63E-68 | down |
| VIT_07s0151g00640.t01 | 1980 | vicilin-like antimicrobial peptides 2-1-like | 40 | 1.3642038 | 129 | 5.9231795 | -2.118313 | 5.69E-18 | 8.89E-19 | down |
| VIT_07s0005g02460.t01 | 423 | probable cellulose synthase A catalytic subunit 8 [UDP-forming] | 9 | 1.4367678 | 29 | 6.2328691 | -2.117069 | 6.84E-05 | 2.74E-05 | down |
| VIT_11s0016g03710.t01 | 984 | probable protein ABIL5-like | 32 | 2.1960353 | 103 | 9.5163958 | -2.115514 | 1.52E-14 | 2.79E-15 | down |
| VIT_14s0060g00350.t01 | 1553 | aspartic proteinase-like | 61 | 2.6524232 | 196 | 11.474004 | -2.112986 | 1.15E-26 | 1.30E-27 | down |
| VIT_02s0087g00390.t01 | 1414 | carbon catabolite repressor protein 4 homolog 4-like | 47 | 2.2445686 | 151 | 9.7086291 | -2.112829 | 8.72E-21 | 1.21E-21 | down |
| VIT_06s0004g02740.t01 | 1420 | GATA transcription factor 8-like | 38 | 1.8070896 | 122 | 7.810914 | -2.111823 | 5.41E-17 | 8.84E-18 | down |
| VIT_14s0006g02870.t01 | 1318 | UDP-N-acetylenolpyruvoylglucosamine reductase-like | 44 | 2.2543519 | 141 | 9.7259957 | -2.109133 | 1.93E-19 | 2.83E-20 | down |
| VIT_11s0016g04250.t01 | 2013 | protein mrp homolog | 281 | 9.4264244 | 900 | 40.647057 | -2.108368 | 1.48E-118 | 4.23E-120 | down |
| VIT_02s0033g00680.t01 | 268 | Putative uncharacterized protein | 10 | 2.5197047 | 32 | 10.855393 | -2.107085 | 2.93E-05 | 1.12E-05 | down |
| VIT_00s0265g00010.t01 | 983 | probable phosphoglycerate mutase gpmB-like | 60 | 4.121755 | 192 | 17.757347 | -2.107085 | 4.71E-26 | 5.41E-27 | down |
| VIT_10s0116g00420.t01 | 1425 | chaperone protein dnaJ 50 | 31 | 1.4690321 | 99 | 6.3161248 | -2.104174 | 6.20E-14 | 1.18E-14 | down |
| VIT_14s0083g00940.t01 | 1863 | DUF246 domain-containing protein | 54 | 1.9573358 | 172 | 8.3935555 | -2.100391 | 2.33E-23 | 2.93E-24 | down |
| VIT_11s0037g00320.t01 | 1690 | serine carboxypeptidase 24-like | 38 | 1.518383 | 121 | 6.5092213 | -2.099949 | 9.50E-17 | 1.56E-17 | down |
| VIT_18s0001g00890.t01 | 1631 | pentatricopeptide repeat-containing protein | 29 | 1.2006833 | 92 | 5.1281916 | -2.094594 | 5.93E-13 | 1.18E-13 | down |
| VIT_11s0016g05110.t01 | 1449 | protein brittle-1, chloroplastic/amyloplastic-like | 151 | 7.0370884 | 479 | 30.053669 | -2.094491 | 5.33E-63 | 2.78E-64 | down |
| VIT_18s0001g08620.t01 | 741 | psaB; photosystem I P700 chlorophyll a apoprotein A2 | 2985 | 272.0261 | 9459 | 1160.5327 | -2.09297 | 0 | 0 | down |
| VIT_04s0008g00780.t01 | 696 | Putative uncharacterized protein | 18 | 1.746416 | 57 | 7.4455364 | -2.091979 | 1.94E-08 | 5.38E-09 | down |
| VIT_14s0006g00630.t01 | 906 | glutathione S-transferase DHAR3 | 220 | 16.397549 | 696 | 69.841155 | -2.090597 | 6.59E-91 | 2.44E-92 | down |
| VIT_08s0032g00420.t01 | 1398 | probable glycosyltransferase | 31 | 1.4974039 | 98 | 6.3730786 | -2.089527 | 1.08E-13 | 2.08E-14 | down |
| VIT_05s0102g00560.t01 | 580 | peptidyl-prolyl cis-trans isomerase E-like | 19 | 2.212127 | 60 | 9.404888 | -2.087977 | 8.31E-09 | 2.24E-09 | down |
| VIT_17s0053g00760.t01 | 522 | cold-inducible RNA-binding protein-like | 32 | 4.1396528 | 101 | 17.590624 | -2.087225 | 4.65E-14 | 8.76E-15 | down |
| VIT_15s0048g01910.t01 | 376 | ribosomal protein L2 (rpl2) and ribosomal protein L23 (rpl23) genes | 138 | 24.784244 | 435 | 105.17967 | -2.085361 | 5.44E-57 | 3.14E-58 | down |
| VIT_08s0040g00930.t01 | 1588 | G2/mitotic-specific cyclin S13-7 | 79 | 3.3593947 | 249 | 14.255394 | -2.085235 | 4.70E-33 | 4.45E-34 | down |
| VIT_16s0039g00990.t01 | 756 | glutathione S-transferase | 27 | 2.4117174 | 85 | 10.221803 | -2.083517 | 5.57E-12 | 1.18E-12 | down |
| VIT_18s0001g00630.t01 | 289 | pentatricopeptide repeat-containing protein | 7 | 1.6356284 | 22 | 6.9207827 | -2.08109 | 0.00065257 | 0.00030262 | down |
| VIT_02s0025g03230.t01 | 2455 | Putative uncharacterized protein | 64 | 1.7604063 | 201 | 7.4434613 | -2.080065 | 8.45E-27 | 9.50E-28 | down |
| VIT_13s0019g02630.t01 | 2118 | psbA; photosystem II P680 reaction center D1 protein | 8308 | 264.88354 | 26049 | 1118.1382 | -2.077668 | 0 | 0 | down |
| VIT_11s0016g04560.t01 | 1583 | sodium/pyruvate cotransporter BASS2 | 67 | 2.858106 | 210 | 12.060596 | -2.07717 | 6.67E-28 | 7.21E-29 | down |
| VIT_11s0016g04120.t01 | 170 | Putative uncharacterized protein | 15 | 5.9583606 | 47 | 25.135024 | -2.076712 | 4.26E-07 | 1.34E-07 | down |
| VIT_18s0001g14170.t01 | 1612 | cyclin-B2-4-like | 61 | 2.5553432 | 191 | 10.772058 | -2.075705 | 1.88E-25 | 2.20E-26 | down |
| VIT_09s0096g00010.t01 | 1311 | putative disease resistance protein RGA3 | 25 | 1.2877209 | 78 | 5.409066 | -2.07056 | 5.22E-11 | 1.19E-11 | down |
| VIT_09s0002g08340.t01 | 807 | Putative uncharacterized protein | 2380 | 199.15346 | 7419 | 835.7997 | -2.069277 | 0 | 0 | down |
| VIT_02s0012g01340.t01 | 1969 | ubiquitin carboxyl-terminal hydrolase 21 | 35 | 1.2003469 | 109 | 5.0328172 | -2.067915 | 6.34E-15 | 1.15E-15 | down |
| VIT_07s0141g00330.t01 | 1989 | F-box/LRR-repeat protein 17-like | 36 | 1.2222278 | 112 | 5.1193357 | -2.066443 | 2.73E-15 | 4.84E-16 | down |
| VIT_06s0004g00240.t01 | 2107 | ruBisCO large subunit-binding protein subunit beta | 2116 | 67.816531 | 6577 | 283.78777 | -2.065104 | 0 | 0 | down |
| VIT_16s0013g00260.t01 | 1178 | Fe Superoxide dismutase | 156 | 8.9425989 | 484 | 37.353426 | -2.062475 | 2.01E-62 | 1.06E-63 | down |
| VIT_08s0105g00280.t01 | 1107 | ribosome biogenesis protein NSA2 homolog | 98 | 5.9780962 | 304 | 24.966424 | -2.062231 | 1.40E-39 | 1.14E-40 | down |
| VIT_04s0008g02590.t01 | 796 | selenoprotein H | 80 | 6.7867424 | 248 | 28.324939 | -2.061282 | 1.86E-32 | 1.79E-33 | down |
| VIT_11s0037g01170.t01 | 1575 | Putative uncharacterized protein | 1156 | 49.563472 | 3575 | 206.36016 | -2.057815 | 0 | 0 | down |
| VIT_14s0066g00970.t01 | 1422 | anthranilate phosphoribosyltransferase | 98 | 4.6538344 | 303 | 19.371953 | -2.057478 | 2.44E-39 | 2.00E-40 | down |
| VIT_05s0049g01130.t01 | 1456 | protein tas-like | 166 | 7.6989439 | 513 | 32.03217 | -2.056789 | 7.05E-66 | 3.55E-67 | down |
| VIT_05s0029g01410.t01 | 402 | serine/threonine-protein phosphatase 6 regulatory ankyrin repeat subunit B-like | 12 | 2.0157638 | 37 | 8.3676989 | -2.053504 | 9.37E-06 | 3.39E-06 | down |
| VIT_04s0008g01220.t01 | 2163 | probable leucine-rich repeat receptor-like protein kinase | 329 | 10.271262 | 1014 | 42.619839 | -2.052912 | 5.18E-129 | 1.33E-130 | down |
| VIT_09s0018g01630.t01 | 1176 | tubby-like protein 8-like | 25 | 1.4355461 | 77 | 5.952697 | -2.051944 | 9.07E-11 | 2.11E-11 | down |
| VIT_00s0271g00060.t01 | 1785 | (3S,6E)-nerolidol synthase 1 | 113 | 4.2748873 | 348 | 17.724394 | -2.051778 | 7.14E-45 | 5.20E-46 | down |
| VIT_01s0026g02330.t01 | 1694 | phytochrome kinase substrate 1 | 43 | 1.7141132 | 132 | 7.0842014 | -2.047143 | 1.26E-17 | 2.00E-18 | down |
| VIT_15s0021g00850.t01 | 785 | Putative uncharacterized protein | 15 | 1.2903456 | 46 | 5.3274398 | -2.045685 | 7.42E-07 | 2.38E-07 | down |
| VIT_03s0091g00660.t01 | 2287 | QWRF motif-containing protein 2 | 49 | 1.4468195 | 150 | 5.9628717 | -2.043122 | 7.81E-20 | 1.13E-20 | down |
| VIT_06s0080g00030.t01 | 1560 | putative aryl-alcohol dehydrogenase C977.14c-like | 511 | 22.119777 | 1563 | 91.088752 | -2.041936 | 4.51E-197 | 7.55E-199 | down |
| VIT_01s0011g02930.t01 | 1619 | aspartic proteinase nepenthesin-1-like | 283 | 11.803859 | 865 | 48.573526 | -2.040912 | 2.33E-109 | 7.12E-111 | down |
| VIT_01s0011g04770.t01 | 2043 | U-box domain-containing protein 10-like | 71 | 2.3467911 | 217 | 9.6565444 | -2.040818 | 3.57E-28 | 3.82E-29 | down |
| VIT_05s0020g02620.t01 | 923 | 40S ribosomal protein S19 | 22 | 1.6095535 | 67 | 6.5993851 | -2.035671 | 1.98E-09 | 5.09E-10 | down |
| VIT_05s0077g02200.t01 | 788 | protein FAM136A-like | 22 | 1.8853019 | 67 | 7.7299905 | -2.035671 | 1.98E-09 | 5.09E-10 | down |
| VIT_04s0044g01180.t01 | 2771 | ATP-dependent zinc metalloprotease FtsH-like | 119 | 2.8999792 | 361 | 11.844072 | -2.030051 | 6.87E-46 | 4.90E-47 | down |
| VIT_12s0121g00230.t01 | 2327 | beta-xylosidase/alpha-L-arabinofuranosidase 2-like | 98 | 2.8438988 | 297 | 11.603538 | -2.028623 | 6.72E-38 | 5.66E-39 | down |
| VIT_19s0027g00500.t01 | 1683 | GTP-dependent nucleic acid-binding protein engD-like | 103 | 4.1327349 | 311 | 16.799898 | -2.023284 | 1.65E-39 | 1.35E-40 | down |
| VIT_00s0759g00010.t01 | 1346 | porphobilinogen deaminase | 822 | 41.239292 | 2476 | 167.23838 | -2.019815 | 0 | 0 | down |
| VIT_01s0026g00610.t01 | 1860 | protein NLRC3-like | 88 | 3.1948772 | 265 | 12.952789 | -2.01943 | 9.87E-34 | 9.18E-35 | down |
| VIT_13s0067g03620.t01 | 1613 | ribulose-1,5 bisphosphate carboxylase/oxygenase large subunit N-methyltransferase | 32 | 1.3396769 | 96 | 5.4108717 | -2.013976 | 7.36E-13 | 1.47E-13 | down |
| VIT_01s0011g00920.t01 | 1548 | pentatricopeptide repeat-containing protein | 32 | 1.3959294 | 96 | 5.6380724 | -2.013976 | 7.36E-13 | 1.47E-13 | down |
| VIT_10s0003g01050.t01 | 2022 | protein CHUP1 | 73 | 2.4379576 | 219 | 9.8467596 | -2.013976 | 5.86E-28 | 6.33E-29 | down |

| | | | | | | | | | | |
|---|---|---|---|---|---|---|---|---|---|---|
| VIT_01s0137g00370.t01 | 1004 | Putative uncharacterized protein | 42 | 2.8248801 | 126 | 11.409516 | -2.013976 | 1.53E-16 | 2.55E-17 | down |
| VIT_16s0022g00670.t01 | 2696 | beta-fructofuranosidase, soluble isoenzyme I-like | 882 | 22.091904 | 2639 | 88.991776 | -2.010154 | 0 | 0 | down |
| VIT_12s0059g01250.t01 | 1908 | endoglucanase 11-like | 209 | 7.3969445 | 625 | 29.780502 | -2.009367 | 1.07E-77 | 4.56E-79 | down |
| VIT_12s0059g00770.t01 | 1706 | L-ascorbate oxidase homolog-like | 174 | 6.8873898 | 520 | 27.711159 | -2.008438 | 9.14E-65 | 4.66E-66 | down |
| VIT_14s0081g00200.t01 | 1092 | GRF1-interacting factor 1-like | 38 | 2.3498785 | 113 | 9.4077589 | -2.001265 | 7.77E-15 | 1.42E-15 | down |
| VIT_00s2440g00010.t01 | 1100 | Putative uncharacterized protein | 1439 | 88.339015 | 4273 | 353.15925 | -1.999196 | 0 | 0 | down |
| VIT_18s0001g02530.t01 | 2030 | DEAD-box ATP-dependent RNA helicase 39-like | 129 | 4.2911937 | 383 | 17.152724 | -1.998987 | 1.12E-47 | 7.69E-49 | down |
| VIT_15s0048g01150.t01 | 1266 | transcription factor TCP9-like | 32 | 1.7068711 | 95 | 6.8221344 | -1.998869 | 1.26E-12 | 2.58E-13 | down |
| VIT_07s0005g02430.t01 | 1916 | chaperone activity of bc1 complex-like | 55 | 1.9384367 | 163 | 7.734326 | -1.996382 | 7.46E-21 | 1.03E-21 | down |
| VIT_12s0035g01050.t01 | 1356 | Putative uncharacterized protein | 27 | 1.3445858 | 80 | 5.363653 | -1.996054 | 8.61E-11 | 2.00E-11 | down |
| VIT_07s0031g03220.t01 | 1795 | patellin-5-like | 162 | 6.0944568 | 479 | 24.260594 | -1.993045 | 3.80E-59 | 2.11E-60 | down |
| VIT_01s0026g00700.t01 | 2062 | Putative uncharacterized protein | 97 | 3.1766365 | 286 | 12.609787 | -1.988972 | 1.24E-35 | 1.11E-36 | down |
| VIT_03s0180g00040.t01 | 1665 | cyclin-D3-2-like | 258 | 10.463812 | 759 | 41.443642 | -1.985742 | 9.90E-93 | 3.57E-94 | down |
| VIT_06s0004g02520.t01 | 1680 | Putative uncharacterized protein | 32 | 1.2862493 | 94 | 5.0868502 | -1.983602 | 2.17E-12 | 4.49E-13 | down |
| VIT_07s0005g04550.t01 | 1137 | ribosome biogenesis protein BRX1 homolog-like | 79 | 4.6919251 | 232 | 18.550597 | -1.983214 | 5.46E-29 | 5.74E-30 | down |
| VIT_08s0007g01700.t01 | 1352 | mitochondrial uncoupling protein 3-like | 85 | 4.2454788 | 249 | 16.743761 | -1.979624 | 5.73E-31 | 5.73E-32 | down |
| VIT_00s0269g00080.t01 | 379 | separase | 14 | 2.4944412 | 41 | 9.8350148 | -1.979211 | 5.13E-06 | 1.80E-06 | down |
| VIT_13s0067g01180.t01 | 1828 | protein ESKIMO 1 | 67 | 2.4750447 | 196 | 9.7478818 | -1.977634 | 1.45E-24 | 1.74E-25 | down |
| VIT_18s0001g03670.t01 | 1610 | Putative uncharacterized protein | 116 | 4.8653777 | 339 | 19.142744 | -1.976174 | 1.07E-41 | 8.26E-43 | down |
| VIT_18s0089g01060.t01 | 482 | vacuolar protein sorting-associated protein 9A-like | 12 | 1.6811972 | 35 | 6.601633 | -1.973334 | 2.80E-05 | 1.07E-05 | down |
| VIT_11s0052g01330.t01 | 850 | xyloglucan endotransglucosylase/hydrolase protein 22-like | 22 | 1.7477858 | 64 | 6.8452832 | -1.969582 | 1.01E-08 | 2.75E-09 | down |
| VIT_18s0001g14450.t01 | 1207 | petH; ferredoxin--NADP+ reductase | 595 | 33.288493 | 1729 | 130.23212 | -1.96799 | 1.03E-207 | 1.62E-209 | down |
| VIT_02s0025g04190.t01 | 2011 | DEAD-box ATP-dependent RNA helicase 47 | 228 | 7.6560933 | 660 | 29.837487 | -1.962446 | 1.60E-79 | 6.68E-81 | down |
| VIT_08s0040g02400.t01 | 2596 | interactor of constitutive active ROPs 2, chloroplastic-like | 55 | 1.4306798 | 159 | 5.5683023 | -1.960537 | 6.47E-20 | 9.30E-21 | down |
| VIT_03s0091g00230.t01 | 1661 | Putative uncharacterized protein | 128 | 5.2038501 | 369 | 20.197011 | -1.95649 | 1.01E-44 | 7.41E-46 | down |
| VIT_00s0475g00020.t01 | 1711 | hypothetical protein | 59 | 2.3285547 | 170 | 9.0329433 | -1.955761 | 3.74E-21 | 5.11E-22 | down |
| VIT_05s0077g01460.t01 | 850 | leucine-rich repeat extensin-like protein 3 | 124 | 9.8511561 | 357 | 38.183845 | -1.954597 | 3.03E-43 | 2.28E-44 | down |
| VIT_00s0396g00020.t01 | 1290 | mitochondrial genome | 72 | 3.7690095 | 207 | 14.588512 | -1.952575 | 1.79E-25 | 2.10E-26 | down |
| VIT_07s0104g00550.t01 | 1614 | ribosome biogenesis GTPase A-like | 127 | 5.3135483 | 365 | 20.559839 | -1.952081 | 4.07E-44 | 3.00E-45 | down |
| VIT_16s0039g00280.t01 | 769 | Putative uncharacterized protein | 31 | 2.7221985 | 89 | 10.521897 | -1.950551 | 1.47E-11 | 3.24E-12 | down |
| VIT_04s0008g02570.t01 | 1190 | replication factor A protein 2-like | 23 | 1.3051647 | 66 | 5.0422845 | -1.949846 | 7.42E-09 | 2.00E-09 | down |
| VIT_03s0017g01750.t01 | 402 | protein ABCI7 | 15 | 2.5197047 | 43 | 9.724623 | -1.948388 | 3.77E-06 | 1.30E-06 | down |
| VIT_14s0068g00300.t01 | 1340 | ninja-family protein AFP2-like | 75 | 3.7795571 | 215 | 14.586935 | -1.948388 | 2.41E-26 | 2.75E-27 | down |
| VIT_01s0146g00110.t01 | 1175 | peptidyl-prolyl cis-trans isomerase CYP20-2 | 660 | 37.93067 | 1886 | 145.92651 | -1.943805 | 7.74E-223 | 1.10E-224 | down |
| VIT_03s0038g01350.t01 | 916 | putative glutamine amidotransferase YLR126C-like | 61 | 4.4969577 | 174 | 17.269674 | -1.94122 | 2.01E-21 | 2.72E-22 | down |
| VIT_11s0037g00070.t01 | 1736 | NADP-dependent glyceraldehyde-3-phosphate dehydrogenase-like | 901 | 35.047699 | 2562 | 134.17135 | -1.936685 | 4.94E-301 | 5.46E-303 | down |
| VIT_07s0031g01420.t01 | 2984 | uncharacterized aarF domain-containing protein kinase | 978 | 22.132195 | 2777 | 84.607222 | -1.934634 | 0 | 0 | down |
| VIT_16s0022g00980.t01 | 1190 | adenylate kinase-like | 30 | 1.7023887 | 85 | 6.4938513 | -1.931514 | 5.83E-11 | 1.34E-11 | down |
| VIT_05s0020g00830.t01 | 1334 | CBL-interacting serine/threonine-protein kinase 1-like (CIPK19) | 34 | 1.7211057 | 96 | 6.5425308 | -1.926513 | 3.41E-12 | 7.15E-13 | down |
| VIT_16s0022g02080.t01 | 1750 | xyloglucan galactosyltransferase KATAMARI1 homolog | 178 | 6.8685711 | 501 | 26.027356 | -1.921947 | 5.74E-59 | 3.20E-60 | down |
| VIT_05s0020g04060.t01 | 1480 | protein TIFY 4B-like | 32 | 1.4600667 | 90 | 5.528549 | -1.920867 | 1.83E-11 | 4.06E-12 | down |
| VIT_11s0016g02400.t01 | 2174 | K(+) efflux antiporter 4-like | 132 | 4.1001414 | 371 | 15.514749 | -1.919895 | 7.20E-44 | 5.36E-45 | down |
| VIT_00s0214g00160.t01 | 1039 | F-box protein PP2-B1-like | 36 | 2.3397605 | 101 | 8.8376378 | -1.9173 | 1.07E-12 | 2.18E-13 | down |
| VIT_19s0015g02800.t01 | 96 | Putative uncharacterized protein | 659 | 463.55218 | 1847 | 1749.1459 | -1.915847 | 4.32E-214 | 6.44E-216 | down |
| VIT_05s0051g00690.t01 | 449 | glycine-rich cell wall structural protein 2-like | 10 | 1.5039663 | 28 | 5.6694648 | -1.91444 | 0.00026044 | 0.00011361 | down |
| VIT_07s0005g00100.t01 | 1481 | tetratricopeptide repeat domain containing protein, expressed | 165 | 7.5233857 | 462 | 28.360722 | -1.91444 | 3.58E-54 | 2.15E-55 | down |
| VIT_09s0002g08330.t01 | 792 | Putative uncharacterized protein | 4851 | 413.60953 | 13561 | 1556.6713 | -1.912123 | 0 | 0 | down |
| VIT_19s0015g00650.t01 | 1996 | ribosomal RNA small subunit methyltransferase B-like | 82 | 2.7741999 | 229 | 10.430505 | -1.910665 | 2.68E-27 | 2.96E-28 | down |
| VIT_04s0008g04350.t01 | 1438 | syntaxin-32-like | 45 | 2.1131877 | 125 | 7.9028092 | -1.902945 | 2.59E-15 | 4.59E-16 | down |
| VIT_08s0007g04380.t01 | 2125 | probable polyadenylate-binding protein | 134 | 4.2582417 | 372 | 15.915283 | -1.902083 | 1.89E-43 | 1.41E-44 | down |
| VIT_07s0005g00470.t01 | 1295 | peter Pan-like protein | 65 | 3.3894406 | 180 | 12.636684 | -1.898499 | 1.65E-21 | 2.22E-22 | down |
| VIT_06s0004g00110.t01 | 1102 | Putative uncharacterized protein | 39 | 2.3898325 | 108 | 8.9098939 | -1.898499 | 2.45E-13 | 4.79E-14 | down |
| VIT_10s0071g01010.t01 | 1399 | Putative uncharacterized protein | 150 | 7.2403238 | 415 | 26.968746 | -1.897162 | 3.43E-48 | 2.33E-49 | down |
| VIT_19s0090g00470.t01 | 911 | mimitin | 30 | 2.223757 | 83 | 8.2830463 | -1.897162 | 1.68E-10 | 3.98E-11 | down |
| VIT_03s0091g00240.t01 | 2693 | alpha-L-fucosidase 2-like | 94 | 2.3570888 | 260 | 8.7774298 | -1.896792 | 1.45E-30 | 1.46E-31 | down |
| VIT_10s0003g02170.t01 | 2447 | subtilisin-like protease-like | 63 | 1.7385654 | 173 | 6.4275062 | -1.886362 | 1.51E-20 | 2.12E-21 | down |
| VIT_08s0056g01170.t01 | 2873 | zinc finger protein VAR3 | 312 | 7.3333669 | 856 | 27.087474 | -1.885078 | 1.52E-97 | 5.19E-99 | down |
| VIT_02s0025g02190.t01 | 1256 | short-chain dehydrogenase TIC 32 | 35 | 1.881754 | 96 | 6.9488345 | -1.884693 | 7.12E-12 | 1.53E-12 | down |
| VIT_05s0029g00720.t01 | 626 | methyltransferase-like protein 13 | 31 | 3.3440426 | 85 | 12.344542 | -1.884208 | 1.22E-10 | 2.87E-11 | down |
| VIT_07s0151g00340.t01 | 2409 | sulfate transporter 3.1-like | 385 | 10.79216 | 1055 | 39.814937 | -1.883326 | 7.14E-120 | 2.00E-121 | down |
| VIT_12s0142g00310.t01 | 1116 | oxygen-evolving enhancer protein 3-1 | 23 | 1.3917079 | 63 | 5.1322373 | -1.882731 | 3.66E-08 | 1.04E-08 | down |
| VIT_19s0014g04930.t01 | 1856 | (E)-beta-caryophyllene synthase | 80 | 2.9106934 | 219 | 10.72745 | -1.881872 | 1.20E-25 | 1.40E-26 | down |
| VIT_10s0116g00100.t01 | 2388 | protein STRUBBELIG-RECEPTOR FAMILY 8-like | 87 | 2.4601941 | 238 | 9.0609348 | -1.880888 | 9.27E-28 | 1.01E-28 | down |
| VIT_11s0016g01180.t01 | 2692 | Putative uncharacterized protein | 55 | 1.37966 | 150 | 5.0657829 | -1.876472 | 7.70E-18 | 1.21E-18 | down |
| VIT_11s0016g04370.t01 | 1428 | syntaxin-32-like | 51 | 2.4117174 | 139 | 8.849464 | -1.875529 | 1.34E-16 | 2.22E-17 | down |
| VIT_00s0225g00070.t01 | 1762 | MATE efflux family protein | 36 | 1.3796885 | 98 | 5.0565062 | -1.873798 | 5.19E-12 | 1.10E-12 | down |
| VIT_18s0001g03610.t01 | 2033 | Putative uncharacterized protein | 100 | 3.321598 | 272 | 12.163594 | -1.87262 | 2.13E-31 | 2.10E-32 | down |
| VIT_19s0015g01230.t01 | 1296 | Putative uncharacterized protein | 32 | 1.6673602 | 87 | 6.1030176 | -1.871957 | 8.90E-11 | 2.06E-11 | down |
| VIT_17s0000g07190.t01 | 2937 | heat shock protein 101 | 186 | 4.2765489 | 505 | 15.632117 | -1.869994 | 2.57E-57 | 1.47E-58 | down |
| VIT_03s0038g00050.t01 | 2721 | BEL1-like homeodomain protein 3-like | 84 | 2.0846598 | 228 | 7.6179247 | -1.869586 | 1.97E-26 | 2.24E-27 | down |
| VIT_01s0011g02100.t01 | 1142 | ATP-dependent Clp protease proteolytic subunit 2 | 52 | 3.0748341 | 141 | 11.224923 | -1.868125 | 9.73E-17 | 1.60E-17 | down |
| VIT_08s0056g00980.t01 | 1141 | F-box protein SKIP24-like | 31 | 1.8346807 | 84 | 6.6930491 | -1.867135 | 2.06E-10 | 4.92E-11 | down |
| VIT_11s0052g00490.t01 | 437 | Putative uncharacterized protein | 17 | 2.6269507 | 46 | 9.5698861 | -1.865113 | 3.40E-06 | 1.17E-06 | down |
| VIT_09s0002g06010.t01 | 1143 | acyl-protein thioesterase 2-like | 61 | 3.6038611 | 165 | 13.124056 | -1.864598 | 2.34E-19 | 3.44E-20 | down |
| VIT_07s0005g05150.t01 | 281 | Putative uncharacterized protein | 10 | 2.4031347 | 27 | 8.735501 | -1.861973 | 0.00044376 | 0.0001994 | down |
| VIT_09s0002g08350.t01 | 267 | Putative uncharacterized protein | 43 | 10.87531 | 116 | 39.498181 | -1.86073 | 6.79E-14 | 1.29E-14 | down |
| VIT_13s0067g01850.t01 | 509 | Putative uncharacterized protein | 29 | 3.8473762 | 78 | 13.931799 | -1.856435 | 1.11E-09 | 2.80E-10 | down |
| VIT_08s0007g08760.t01 | 1782 | NADPH-dependent thioredoxin reductase 3-like | 425 | 16.105183 | 1143 | 58.313472 | -1.856304 | 2.66E-127 | 6.94E-129 | down |
| VIT_04s0008g04710.t01 | 1555 | 3-ketoacyl-CoA synthase 11-like | 38 | 1.650204 | 102 | 5.9634853 | -1.853511 | 2.73E-12 | 5.69E-13 | down |
| VIT_00s1546g00010.t01 | 685 | tafazzin homolog | 22 | 2.1687853 | 59 | 7.8305418 | -1.852225 | 1.43E-07 | 4.27E-08 | down |
| VIT_07s0129g00250.t01 | 2233 | BTB/POZ domain-containing protein NPY2-like | 140 | 4.2337358 | 375 | 15.267675 | -1.850477 | 3.13E-42 | 2.39E-43 | down |
| VIT_08s0007g07270.t01 | 972 | putative mitochondrial 2-oxoglutarate/malate carrier protein-like | 28 | 1.9452535 | 75 | 7.0149628 | -1.850477 | 2.57E-09 | 6.67E-10 | down |
| VIT_19s0027g01850.t01 | 620 | Putative uncharacterized protein | 21 | 2.2872416 | 56 | 8.2115797 | -1.844051 | 3.32E-07 | 1.03E-07 | down |
| VIT_03s0063g02080.t01 | 1211 | probable 1-acyl-sn-glycerol-3-phosphate acyltransferase 5-like | 41 | 2.2862523 | 109 | 8.1830033 | -1.839646 | 6.20E-13 | 1.24E-13 | down |
| VIT_04s0008g01110.t01 | 1415 | heat shock factor protein HSF30 | 76 | 3.6269502 | 202 | 12.978524 | -1.839297 | 4.79E-23 | 6.11E-24 | down |
| VIT_18s0001g10540.t01 | 1904 | protein CYPRO4-like | 73 | 2.5890495 | 194 | 9.2632878 | -1.839102 | 3.58E-22 | 4.71E-23 | down |
| VIT_00s1455g00020.t01 | 548 | ATP-dependent Clp protease proteolytic subunit-related protein 4 | 29 | 3.5735666 | 77 | 12.774401 | -1.837819 | 1.86E-09 | 4.78E-10 | down |
| VIT_03s0038g04080.t01 | 1894 | glutamyl-tRNA synthetase | 267 | 9.5195349 | 708 | 33.984717 | -1.835923 | 5.04E-78 | 2.14E-79 | down |
| VIT_14s0060g00720.t01 | 1068 | ABC transporter I family member 20-like | 111 | 7.0183685 | 294 | 25.026865 | -1.83427 | 6.01E-33 | 5.72E-34 | down |
| VIT_05s0077g01770.t01 | 2429 | pumilio homolog 24-like | 88 | 2.4464683 | 233 | 8.7208492 | -1.833768 | 2.58E-26 | 2.94E-27 | down |
| VIT_12s0028g01670.t01 | 2590 | probable alpha,alpha-trehalose-phosphate synthase [UDP-forming] 7-like | 68 | 1.7729382 | 180 | 6.3183418 | -1.833404 | 1.44E-20 | 2.02E-21 | down |
| VIT_12s0035g02010.t01 | 766 | Putative uncharacterized protein | 22719 | 2002.8337 | 60104 | 7133.538 | -1.832575 | 0 | 0 | down |
| VIT_19s0090g00300.t01 | 2131 | polyadenylate-binding protein-interacting protein 4 | 108 | 3.4223526 | 285 | 12.15883 | -1.828944 | 7.59E-32 | 7.40E-33 | down |
| VIT_00s0194g00310.t01 | 1203 | pentatricopeptide repeat-containing protein | 47 | 2.6382544 | 124 | 9.3710106 | -1.828621 | 1.85E-14 | 3.43E-15 | down |
| VIT_11s0016g02090.t01 | 1146 | Putative uncharacterized protein | 47 | 2.7694765 | 124 | 9.837108 | -1.828621 | 1.85E-14 | 3.43E-15 | down |
| VIT_17s0000g05070.t01 | 1809 | protein COBRA-like | 678 | 25.309034 | 1788 | 89.858532 | -1.828003 | 1.35E-194 | 2.29E-196 | down |
| VIT_07s0005g00350.t01 | 351 | Putative uncharacterized protein | 11 | 2.1162648 | 29 | 7.5114063 | -1.827563 | 0.00031963 | 0.00014097 | down |

| | | | | | | | | | | |
|---|---|---|---|---|---|---|---|---|---|---|
| VIT_14s0060g02090.t01 | 1392 | brain acid soluble protein 1 | 49 | 2.3770663 | 129 | 8.4252122 | -1.825531 | 5.74E-15 | 1.04E-15 | down |
| VIT_01s0011g01080.t01 | 1815 | ribulose-1,5 bisphosphate carboxylase/oxygenase large subunit N-methyltransferase | 227 | 8.4456615 | 597 | 29.903917 | -1.824052 | 2.28E-65 | 1.15E-66 | down |
| VIT_07s0129g00470.t01 | 1120 | uncharacterized protein ycf36 | 135 | 8.1395461 | 355 | 28.816465 | -1.823873 | 2.84E-39 | 2.32E-40 | down |
| VIT_00s1682g00020.t01 | 1636 | cytochrome P450 93A3-like | 143 | 5.9025161 | 376 | 20.894641 | -1.823731 | 1.57E-41 | 1.22E-42 | down |
| VIT_05s0020g04780.t01 | 1057 | transcription factor bHLH92-like | 133 | 8.4969115 | 349 | 30.017935 | -1.820814 | 1.53E-38 | 1.28E-39 | down |
| VIT_00s2731g00010.t01 | 684 | oleoyl-acyl carrier protein thioesterase | 114 | 11.254681 | 299 | 39.74161 | -1.820125 | 3.79E-33 | 3.58E-34 | down |
| VIT_12s0142g00400.t01 | 825 | copper-transporting ATPase PAA1 | 29 | 2.3737146 | 76 | 8.3751003 | -1.81896 | 3.12E-09 | 8.14E-10 | down |
| VIT_09s0002g03350.t01 | 2163 | Putative uncharacterized protein | 54 | 1.6858607 | 141 | 5.9264274 | -1.813677 | 4.03E-16 | 6.85E-17 | down |
| VIT_02s0025g02650.t01 | 1502 | cellulase CEL2-like | 64 | 2.8773619 | 167 | 10.108272 | -1.812718 | 7.01E-19 | 1.05E-19 | down |
| VIT_05s0051g00530.t01 | 787 | glycine-rich cell wall structural protein | 23 | 1.9735019 | 60 | 6.9311754 | -1.812342 | 1.73E-07 | 5.21E-08 | down |
| VIT_15s0021g01430.t01 | 3314 | glycyl-tRNA synthetase 2, chloroplastic/mitochondrial-like | 74 | 1.5078692 | 193 | 5.2946247 | -1.812017 | 1.22E-21 | 1.63E-22 | down |
| VIT_08s0007g07820.t01 | 2232 | probable inactive leucine-rich repeat receptor-like protein kinase | 74 | 2.2388344 | 193 | 7.8612841 | -1.812017 | 1.22E-21 | 1.63E-22 | down |
| VIT_00s0804g00020.t01 | 1478 | Putative uncharacterized protein | 66 | 3.0154626 | 172 | 10.579969 | -1.810884 | 2.18E-19 | 3.20E-20 | down |
| VIT_00s2608g00020.t01 | 124 | mitochondrial genome | 387 | 210.75298 | 1008 | 739.04217 | -1.810104 | 1.34E-108 | 4.13E-110 | down |
| VIT_12s0142g00200.t01 | 1260 | ribosomal L1 domain-containing protein 1-like | 101 | 5.4129657 | 262 | 18.904323 | -1.804225 | 7.79E-29 | 8.22E-30 | down |
| VIT_05s0094g01200.t01 | 532 | geraniol 8-hydroxylase-like | 27 | 3.4271773 | 70 | 11.962358 | -1.803409 | 1.68E-08 | 4.62E-09 | down |
| VIT_07s0031g01060.t01 | 1208 | chloroplast DNA | 4218 | 235.7893 | 10915 | 821.46143 | -1.800695 | 0 | 0 | down |
| VIT_05s0020g01270.t01 | 1066 | protein FAM179B-like | 29 | 1.837068 | 75 | 6.3963826 | -1.799851 | 5.20E-09 | 1.38E-09 | down |
| VIT_07s0005g00510.t01 | 1372 | monoglyceride lipase-like | 48 | 2.3624987 | 124 | 8.2167098 | -1.798247 | 3.74E-14 | 7.01E-15 | down |
| VIT_08s0056g01000.t01 | 1653 | MATE efflux family protein | 331 | 13.521958 | 854 | 46.969441 | -1.796418 | 3.38E-91 | 1.24E-92 | down |
| VIT_15s0021g01850.t01 | 1468 | FKBP-type peptidyl-prolyl cis-trans isomerase 6 | 61 | 2.8060036 | 157 | 9.7230825 | -1.792897 | 1.45E-17 | 2.31E-18 | down |
| VIT_17s0000g00460.t01 | 1292 | ATPF0C, atpF; F-type H+-transporting ATPase subunit C | 35 | 1.8293212 | 90 | 6.3330128 | -1.791584 | 1.57E-10 | 3.72E-11 | down |
| VIT_15s0021g02410.t01 | 1231 | malate dehydrogenase, cytoplasmic-like | 28 | 1.535976 | 72 | 5.3174672 | -1.791584 | 1.20E-08 | 3.29E-09 | down |
| VIT_07s0031g01360.t01 | 1879 | pentatricopeptide repeat-containing protein | 86 | 3.0906947 | 221 | 10.692909 | -1.790651 | 3.03E-24 | 3.70E-25 | down |
| VIT_14s0006g00850.t01 | 2460 | valyl-tRNA synthetase-like | 65 | 1.7842787 | 167 | 6.1717985 | -1.79035 | 1.40E-18 | 2.13E-19 | down |
| VIT_00s0215g00090.t01 | 1683 | Putative uncharacterized protein | 37 | 1.4845747 | 95 | 5.1318016 | -1.789416 | 4.91E-11 | 1.12E-11 | down |
| VIT_14s0060g01050.t01 | 1993 | pentatricopeptide repeat-containing protein | 173 | 5.8616954 | 443 | 20.208161 | -1.785548 | 2.21E-47 | 1.52E-48 | down |
| VIT_06s0009g02520.t01 | 2856 | replication factor C subunit 1-like | 100 | 2.3644288 | 256 | 8.1491467 | -1.785157 | 8.39E-28 | 9.10E-29 | down |
| VIT_11s0016g02350.t01 | 1384 | Putative uncharacterized protein | 102 | 4.9767809 | 261 | 17.144893 | -1.784494 | 2.60E-28 | 2.78E-29 | down |
| VIT_01s0011g00550.t01 | 1274 | monoacylglycerol lipase ABHD6 | 165 | 8.7457883 | 422 | 30.114343 | -1.78379 | 3.98E-45 | 2.89E-46 | down |
| VIT_11s0052g01600.t01 | 1529 | anthocyanidin 3-O-glucosyltransferase 2-like | 149 | 6.5805657 | 381 | 22.654155 | -1.783492 | 7.91E-41 | 6.23E-42 | down |
| VIT_07s0031g01560.t01 | 972 | Putative uncharacterized protein | 126 | 8.7536408 | 322 | 30.117574 | -1.78265 | 1.21E-34 | 1.10E-35 | down |
| VIT_12s0055g00530.t01 | 223 | Putative uncharacterized protein | 56 | 16.957726 | 143 | 58.299059 | -1.78153 | 5.79E-16 | 9.92E-17 | down |
| VIT_06s0080g00440.t01 | 1503 | 28S ribosomal protein S9 | 152 | 6.8291877 | 388 | 23.469461 | -1.780999 | 1.76E-41 | 1.36E-42 | down |
| VIT_06s0009g03350.t01 | 1672 | phospholipase C 4-like | 134 | 5.4119399 | 342 | 18.596029 | -1.780777 | 1.11E-36 | 9.67E-38 | down |
| VIT_13s0064g00360.t01 | 1969 | notchless protein homolog-like | 97 | 3.3266757 | 247 | 11.404641 | -1.777468 | 1.04E-26 | 1.17E-27 | down |
| VIT_07s0005g03630.t01 | 1871 | probable methyltransferase PMT8-like | 77 | 2.7790821 | 196 | 9.5238524 | -1.776937 | 2.09E-21 | 2.83E-22 | down |
| VIT_17s0000g06440.t01 | 2110 | galactoside 2-alpha-L-fucosyltransferase-like | 77 | 2.4642951 | 196 | 8.4450843 | -1.776937 | 2.09E-21 | 2.83E-22 | down |
| VIT_18s0001g02540.t01 | 866 | two-component response regulator ARR9-like | 74 | 5.7702984 | 188 | 19.736509 | -1.774149 | 1.56E-20 | 2.18E-21 | down |
| VIT_03s0063g02020.t01 | 1928 | protein TIC 62, chloroplastic-like | 354 | 12.398829 | 899 | 42.391915 | -1.773585 | 2.73E-94 | 9.69E-96 | down |
| VIT_09s0054g01520.t01 | 2610 | beta-amyrin synthase-like | 136 | 3.5187049 | 345 | 12.017357 | -1.772003 | 9.51E-37 | 8.26E-38 | down |
| VIT_06s0061g00270.t01 | 2083 | ruBisCO large subunit-binding protein subunit alpha | 4184 | 135.63971 | 10605 | 462.86226 | -1.770803 | 0 | 0 | down |
| VIT_15s0021g01940.t01 | 1370 | KH domain-containing protein | 30 | 1.4787172 | 76 | 5.0433998 | -1.77005 | 6.21E-09 | 1.66E-09 | down |
| VIT_02s0012g02540.t01 | 2033 | pentatricopeptide repeat-containing protein | 75 | 2.4911985 | 190 | 8.4966278 | -1.77005 | 1.12E-20 | 1.56E-21 | down |
| VIT_12s0142g00220.t01 | 1543 | Putative uncharacterized protein | 90 | 3.9387737 | 228 | 13.433813 | -1.77005 | 1.34E-24 | 1.61E-25 | down |
| VIT_05s0020g01840.t01 | 1187 | divinyl chlorophyllide a 8-vinyl-reductase | 334 | 19.001163 | 846 | 64.796272 | -1.769823 | 1.59E-88 | 5.99E-90 | down |
| VIT_13s0139g00250.t01 | 1691 | translation initiation factor eIF-2B subunit beta-like | 79 | 3.1547716 | 200 | 10.752681 | -1.769089 | 1.08E-21 | 1.44E-22 | down |
| VIT_06s0004g00260.t01 | 1347 | Putative uncharacterized protein | 501 | 25.116237 | 1266 | 85.446934 | -1.766408 | 1.10E-131 | 2.76E-133 | down |
| VIT_17s0000g01850.t01 | 2142 | Putative uncharacterized protein | 109 | 3.4363032 | 275 | 11.671955 | -1.764117 | 2.54E-29 | 2.64E-30 | down |
| VIT_12s0034g02090.t01 | 1055 | chloroplast DNA | 25 | 1.6001916 | 63 | 5.4289828 | -1.762437 | 1.49E-07 | 4.45E-08 | down |
| VIT_00s0233g00180.t01 | 1535 | flavin-containing monooxygenase FMO GS-OX-like 3 | 108 | 4.7511618 | 272 | 16.109828 | -1.761589 | 5.87E-29 | 6.18E-30 | down |
| VIT_00s0415g00030.t01 | 2305 | heat shock 70 kDa protein | 313 | 9.1697575 | 788 | 31.080333 | -1.761046 | 5.84E-82 | 2.39E-83 | down |
| VIT_06s0004g06950.t01 | 1288 | probable N-acetyltransferase HLS1-like | 31 | 1.6252878 | 78 | 5.5056565 | -1.760219 | 4.46E-09 | 1.18E-09 | down |
| VIT_13s0019g02250.t01 | 2516 | probable methyltransferase PMT11-like | 66 | 1.7714045 | 166 | 5.998295 | -1.759659 | 4.60E-18 | 7.15E-19 | down |
| VIT_09s0002g00180.t01 | 1302 | Putative uncharacterized protein | 35 | 1.8152711 | 88 | 6.1447195 | -1.759162 | 4.33E-10 | 1.06E-10 | down |
| VIT_00s0508g00040.t01 | 1238 | Putative uncharacterized protein | 72 | 3.9273201 | 181 | 13.291938 | -1.758934 | 1.37E-19 | 2.01E-20 | down |
| VIT_03s0063g00460.t01 | 979 | ethylene-responsive transcription factor ERF109 | 903 | 62.285865 | 2262 | 210.05851 | -1.753815 | 2.34E-232 | 3.21E-234 | down |
| VIT_05s0020g04100.t01 | 2389 | dymeclin-like | 111 | 3.1375545 | 278 | 10.579351 | -1.753539 | 2.16E-29 | 2.24E-30 | down |
| VIT_18s0001g12950.t01 | 2326 | phosphatidylinositol/phosphatidylcholine transfer protein | 145 | 4.2096185 | 363 | 14.1882 | -1.75293 | 4.75E-38 | 3.99E-39 | down |
| VIT_00s0391g00060.t01 | 644 | Putative uncharacterized protein | 60 | 6.2914366 | 150 | 21.175602 | -1.750942 | 2.55E-16 | 4.28E-17 | down |
| VIT_14s0030g00210.t01 | 1479 | putative ERD6-like transporter | 36 | 1.6436857 | 90 | 5.5322871 | -1.750942 | 3.10E-10 | 7.49E-11 | down |
| VIT_07s0005g02510.t01 | 616 | phytochrome-interacting factor 3 | 18 | 1.9732233 | 45 | 6.6414388 | -1.750942 | 1.14E-05 | 4.15E-06 | down |
| VIT_09s0002g03420.t01 | 464 | cytochrome b5-like | 14 | 2.0374854 | 35 | 6.8577309 | -1.750942 | 0.00011875 | 4.91E-05 | down |
| VIT_17s0000g03310.t01 | 2528 | heat shock protein 70 | 5157 | 137.75409 | 12877 | 463.09277 | -1.749206 | 0 | 0 | down |
| VIT_06s0004g05940.t01 | 1483 | mitochondrial uncoupling protein 2-like | 123 | 5.6007786 | 307 | 18.820346 | -1.748594 | 3.14E-32 | 3.03E-33 | down |
| VIT_04s0079g00690.t01 | 831 | Glutathione S-transferase | 505 | 41.036924 | 1260 | 137.84782 | -1.748082 | 3.45E-129 | 8.81E-131 | down |
| VIT_19s0014g01470.t01 | 2827 | Thioredoxin | 447 | 10.677416 | 1115 | 35.857452 | -1.74771 | 2.16E-114 | 6.34E-116 | down |
| VIT_18s0001g00110.t01 | 1477 | Putative uncharacterized protein | 57 | 2.6060264 | 142 | 8.7405391 | -1.745871 | 1.89E-15 | 3.32E-16 | down |
| VIT_14s0006g03260.t01 | 1432 | Putative uncharacterized protein | 92 | 4.3383966 | 229 | 14.538608 | -1.744655 | 3.12E-24 | 3.81E-25 | down |
| VIT_17s0000g08530.t01 | 2366 | probable boron transporter 2-like | 170 | 4.8519758 | 423 | 16.253841 | -1.744136 | 7.16E-44 | 5.32E-45 | down |
| VIT_04s0008g04310.t01 | 1110 | psbP; photosystem II oxygen-evolving enhancer protein 2 | 169 | 10.281303 | 420 | 34.399861 | -1.74238 | 1.66E-43 | 1.24E-44 | down |
| VIT_08s0105g00430.t01 | 1671 | omega-3 fatty acid desaturase | 1274 | 51.484609 | 3165 | 172.19782 | -1.741854 | 0 | 0 | down |
| VIT_07s0005g02420.t01 | 864 | suppressor of disruption of TFIIS-like | 31 | 2.4228827 | 77 | 8.102282 | -1.741604 | 7.37E-09 | 1.98E-09 | down |
| VIT_13s0074g00370.t01 | 1094 | RING-H2 finger protein ATL8 | 110 | 6.7898442 | 273 | 22.686928 | -1.740411 | 1.37E-28 | 1.45E-29 | down |
| VIT_19s0027g00450.t01 | 2440 | pentatricopeptide repeat-containing protein | 160 | 4.4280712 | 397 | 14.792142 | -1.740081 | 4.19E-41 | 3.28E-42 | down |
| VIT_11s0037g00890.t01 | 961 | putative glutamine amidotransferase YLR126C-like | 98 | 6.8863189 | 243 | 22.988639 | -1.739116 | 1.54E-25 | 1.79E-26 | down |
| VIT_00s0324g00040.t01 | 2037 | dnaJ homolog 1 | 406 | 13.459206 | 1006 | 44.899068 | -1.738092 | 1.64E-102 | 5.40E-104 | down |
| VIT_19s0014g01630.t01 | 1595 | tRNA dimethylallyltransferase 9-like | 65 | 2.7519283 | 161 | 9.1768907 | -1.737563 | 2.89E-17 | 4.67E-18 | down |
| VIT_07s0031g02510.t01 | 1467 | sirtuin 2 (SRT2) | 40 | 1.8412566 | 99 | 6.1352951 | -1.736442 | 4.96E-11 | 1.13E-11 | down |
| VIT_17s0000g05840.t01 | 2335 | protein IQ-DOMAIN 31-like | 110 | 3.1811947 | 272 | 10.590401 | -1.735117 | 2.25E-28 | 2.41E-29 | down |
| VIT_13s0067g01690.t01 | 2285 | probable methyltransferase PMT23-like | 68 | 2.0095886 | 168 | 6.6842618 | -1.733868 | 6.42E-18 | 1.01E-18 | down |
| VIT_05s0049g01110.t01 | 506 | aldo-keto reductase | 17 | 2.2687302 | 42 | 7.5462145 | -1.733868 | 2.62E-05 | 1.00E-05 | down |
| VIT_18s0001g08590.t01 | 689 | Putative uncharacterized protein | 17 | 1.6661502 | 42 | 5.5419224 | -1.733868 | 2.62E-05 | 1.00E-05 | down |
| VIT_14s0066g02200.t01 | 1270 | Putative uncharacterized protein | 117 | 6.2210914 | 289 | 20.688285 | -1.733574 | 4.75E-30 | 4.88E-31 | down |
| VIT_07s0095g00190.t01 | 558 | G-type lectin S-receptor-like serine/threonine-protein kinase CES101 | 32 | 3.8725784 | 79 | 12.871325 | -1.732794 | 5.27E-09 | 1.40E-09 | down |
| VIT_13s0047g00300.t01 | 695 | serrate RNA effector molecule-like | 30 | 2.9148814 | 74 | 9.680043 | -1.731576 | 1.70E-08 | 4.69E-09 | down |
| VIT_11s0052g01640.t01 | 677 | UDP-glycosyltransferase 78D2-like | 28 | 2.7928898 | 69 | 9.265968 | -1.730183 | 5.48E-08 | 1.57E-08 | down |
| VIT_12s0059g02250.t01 | 1045 | 50S ribosomal protein L6 | 408 | 26.365033 | 1005 | 87.433959 | -1.729568 | 1.03E-101 | 3.41E-103 | down |
| VIT_06s0009g03240.t01 | 2855 | rho GTPase-activating protein 7 | 272 | 6.433499 | 670 | 21.335315 | -1.729568 | 4.09E-68 | 1.99E-69 | down |
| VIT_12s0028g02370.t01 | 1380 | Putative uncharacterized protein | 379 | 18.545757 | 933 | 61.465714 | -1.728693 | 2.02E-94 | 7.15E-96 | down |
| VIT_15s0048g03000.t01 | 753 | ras-related protein RABA3-like | 26 | 2.3316471 | 64 | 7.7270793 | -1.728574 | 1.76E-07 | 5.30E-08 | down |
| VIT_01s0127g00320.t01 | 370 | CRS2-associated factor 2 | 26 | 4.7452169 | 64 | 15.725651 | -1.728574 | 1.76E-07 | 5.30E-08 | down |
| VIT_01s0026g01460.t01 | 609 | thioredoxin | 70 | 7.761849 | 172 | 25.676837 | -1.725995 | 3.29E-18 | 5.08E-19 | down |
| VIT_01s0011g02440.t01 | 1179 | HD domain-containing protein 2 homolog | 40 | 2.2910292 | 98 | 7.556882 | -1.721795 | 8.17E-11 | 1.89E-11 | down |
| VIT_17s0000g01720.t01 | 1807 | nucleolar protein 56-like | 234 | 8.7446443 | 573 | 28.828818 | -1.72104 | 5.39E-58 | 3.05E-59 | down |
| VIT_09s0054g00140.t01 | 649 | 60S ribosomal protein L7-2 | 58 | 6.0348675 | 142 | 19.891797 | -1.72078 | 3.64E-15 | 6.53E-16 | down |
| VIT_08s0007g06200.t01 | 1048 | rhodanese-like domain-containing protein 9 | 526 | 33.892914 | 1286 | 111.5604 | -1.718769 | 8.90E-129 | 2.30E-130 | down |

| | | | | | | | | | | |
|---|---|---|---|---|---|---|---|---|---|---|
| VIT_13s0019g02170.t01 | 858 | tropinone reductase homolog | 43 | 3.3842747 | 105 | 11.125829 | -1.716994 | 1.81E-11 | 4.02E-12 | down |
| VIT_00s0194g00270.t01 | 2326 | pentatricopeptide repeat-containing protein | 376 | 10.915976 | 918 | 35.880901 | -1.716775 | 4.98E-92 | 1.81E-93 | down |
| VIT_00s0229g00020.t01 | 2033 | asparaginyl-tRNA synthetase | 202 | 6.7096279 | 493 | 22.046513 | -1.716246 | 7.56E-50 | 4.98E-51 | down |
| VIT_05s0020g01810.t01 | 1476 | Putative uncharacterized protein | 132 | 6.0390972 | 322 | 19.833524 | -1.715536 | 6.82E-33 | 6.50E-34 | down |
| VIT_05s0020g04880.t01 | 1590 | protein WVD2-like 1 | 94 | 3.9922265 | 229 | 13.093891 | -1.713628 | 1.17E-23 | 1.46E-24 | down |
| VIT_01s0011g02720.t01 | 401 | Putative uncharacterized protein | 314 | 52.877354 | 764 | 173.21255 | -1.711822 | 1.96E-76 | 8.48E-78 | down |
| VIT_08s0007g05120.t01 | 2520 | DNA gyrase subunit B | 163 | 4.3678881 | 396 | 14.286473 | -1.709642 | 4.99E-40 | 4.02E-41 | down |
| VIT_07s0095g00710.t01 | 1518 | bystin-like | 35 | 1.5569717 | 85 | 5.0907003 | -1.709121 | 1.92E-09 | 4.95E-10 | down |
| VIT_11s0016g02630.t01 | 1112 | Putative uncharacterized protein | 89 | 5.404676 | 216 | 17.659538 | -1.708168 | 2.79E-22 | 3.66E-23 | down |
| VIT_15s0048g01040.t01 | 2202 | flap endonuclease GEN-like 1 | 54 | 1.6560021 | 131 | 5.4085936 | -1.707549 | 6.23E-14 | 1.18E-14 | down |
| VIT_06s0004g00610.t01 | 1707 | protochlorophyllide-dependent translocon component 52 | 315 | 12.461246 | 764 | 40.690236 | -1.707234 | 3.77E-76 | 1.64E-77 | down |
| VIT_04s0008g00530.t01 | 1232 | serine/threonine-protein kinase Aurora-1-like | 59 | 3.2338938 | 143 | 10.552508 | -1.706242 | 4.26E-15 | 7.68E-16 | down |
| VIT_07s0031g01550.t01 | 217 | Putative uncharacterized protein | 19 | 5.9125974 | 46 | 19.272075 | -1.704648 | 1.34E-05 | 4.93E-06 | down |
| VIT_07s0005g01140.t01 | 726 | Putative uncharacterized protein | 50 | 4.6506947 | 121 | 15.15232 | -1.704021 | 6.49E-13 | 1.30E-13 | down |
| VIT_18s0001g00670.t01 | 846 | U11/U12 small nuclear ribonucleoprotein 25 kDa protein-like | 24 | 1.9156904 | 58 | 6.2328691 | -1.702032 | 9.24E-07 | 3.00E-07 | down |
| VIT_00s0299g00050.t01 | 353 | probable iron/ascorbate oxidoreductase DDB | 12 | 2.2955723 | 29 | 7.4688488 | -1.702032 | 0.00062898 | 0.00029124 | down |
| VIT_01s0026g00600.t01 | 420 | protein NLRC3 | 24 | 3.8587478 | 58 | 12.554779 | -1.702032 | 9.24E-07 | 3.00E-07 | down |
| VIT_08s0040g03370.t01 | 2818 | pentatricopeptide repeat-containing protein | 147 | 3.5225794 | 355 | 11.45296 | -1.701016 | 9.65E-36 | 8.59E-37 | down |
| VIT_13s0106g00740.t01 | 1479 | sodium/bile acid cotransporter 7-like | 85 | 3.8809245 | 205 | 12.601321 | -1.699103 | 4.75E-21 | 6.52E-22 | down |
| VIT_00s2342g00010.t01 | 420 | U-box domain-containing protein 9-like | 39 | 6.2704652 | 94 | 20.347401 | -1.6982 | 3.07E-10 | 7.40E-11 | down |
| VIT_15s0046g02910.t01 | 957 | 50S ribosomal protein L21 | 813 | 57.36712 | 1959 | 186.10278 | -1.697804 | 2.01E-192 | 3.50E-194 | down |
| VIT_00s0125g00080.t01 | 1000 | mitochondrial genome | 71 | 4.7944941 | 171 | 15.54628 | -1.697119 | 1.04E-17 | 1.64E-18 | down |
| VIT_17s0000g03890.t01 | 1540 | cytochrome P450 71D10-like | 64 | 2.806362 | 154 | 9.0913918 | -1.6958 | 4.85E-16 | 8.27E-17 | down |
| VIT_11s0016g05230.t01 | 1094 | NADP-dependent alkenal double bond reductase P1-like | 74 | 4.5677133 | 178 | 14.79221 | -1.695294 | 2.29E-18 | 3.53E-19 | down |
| VIT_11s0016g04380.t01 | 2136 | Putative uncharacterized protein | 67 | 2.1181563 | 161 | 6.852594 | -1.693841 | 1.08E-16 | 1.78E-17 | down |
| VIT_14s0036g01270.t01 | 276 | ATP synthase, A subunit family protein, putative, expressed | 67 | 16.392688 | 161 | 53.033119 | -1.693841 | 1.08E-16 | 1.78E-17 | down |
| VIT_18s0001g12700.t01 | 653 | isoflavone reductase homolog IRL, putative, expressed | 15 | 1.5511812 | 36 | 5.0120996 | -1.692048 | 0.00013872 | 5.82E-05 | down |
| VIT_10s0116g00970.t01 | 3301 | leucyl-tRNA synthetase-like | 167 | 3.4162952 | 400 | 11.01653 | -1.689165 | 9.26E-40 | 7.54E-41 | down |
| VIT_19s0015g01900.t01 | 786 | regulator of ribonuclease-like protein 3 | 107 | 9.1927548 | 256 | 29.61064 | -1.687547 | 8.82E-26 | 1.02E-26 | down |
| VIT_13s0101g00200.t01 | 1389 | chloroplast DNA | 2211 | 107.49071 | 5285 | 345.91797 | -1.686218 | 0 | 0 | down |
| VIT_08s0007g02220.t01 | 1761 | protein root UVB sensitive 5 | 41 | 1.5722042 | 98 | 5.0593776 | -1.686171 | 1.56E-10 | 3.69E-11 | down |
| VIT_11s0037g00280.t01 | 1524 | APO protein 2 | 108 | 4.7854549 | 258 | 15.390939 | -1.685353 | 6.29E-26 | 7.25E-27 | down |
| VIT_11s0016g05610.t01 | 1414 | elongation factor Ts | 80 | 3.8205424 | 191 | 12.280451 | -1.684514 | 1.84E-19 | 2.70E-20 | down |
| VIT_18s0122g01410.t01 | 1743 | carboxypeptidase A2-like | 65 | 2.5182591 | 155 | 8.0847144 | -1.68277 | 5.64E-16 | 9.67E-17 | down |
| VIT_12s0059g02670.t01 | 1812 | beta-amylase-like | 76 | 2.8323038 | 181 | 9.0813571 | -1.680932 | 1.91E-18 | 2.92E-19 | down |
| VIT_12s0028g02630.t01 | 1071 | Rac-like GTP-binding protein ARAC5 | 29 | 1.8284916 | 69 | 5.8571992 | -1.679557 | 1.05E-07 | 3.09E-08 | down |
| VIT_07s0129g00230.t01 | 1362 | probable glucan endo-1,3-beta-glucosidase A6-like | 61 | 3.0243857 | 145 | 9.6787945 | -1.678185 | 5.80E-15 | 1.05E-15 | down |
| VIT_08s0058g01450.t01 | 1780 | serine/threonine-protein kinase STN8 | 381 | 14.454045 | 905 | 46.223087 | -1.67714 | 7.99E-88 | 3.03E-89 | down |
| VIT_04s0008g01340.t01 | 1248 | Putative uncharacterized protein | 48 | 2.5972341 | 114 | 8.3046367 | -1.676941 | 5.53E-12 | 1.18E-12 | down |
| VIT_00s0674g00010.t01 | 460 | splicing factor U2af large subunit B-like | 16 | 2.348803 | 38 | 7.5102802 | -1.676941 | 9.92E-05 | 4.06E-05 | down |
| VIT_11s0016g04830.t01 | 1530 | methyltransferase domain containing protein, expressed | 110 | 4.8549605 | 261 | 15.508845 | -1.67556 | 5.20E-26 | 5.98E-27 | down |
| VIT_06s0004g05580.t01 | 1012 | DNA-directed RNA polymerase subunit alpha, putative, expressed | 207 | 13.812563 | 491 | 44.109421 | -1.675106 | 5.18E-48 | 3.54E-49 | down |
| VIT_13s0067g03200.t01 | 1960 | peptide transporter PTR2, putative, expressed | 175 | 6.0292934 | 415 | 19.249631 | -1.67477 | 1.01E-40 | 7.94E-42 | down |
| VIT_10s0003g04950.t01 | 1174 | esterase/lipase/thioesterase family protein, putative, expressed | 57 | 3.2786209 | 135 | 10.454326 | -1.672939 | 6.03E-14 | 1.14E-14 | down |
| VIT_13s0019g01840.t01 | 1692 | protein FIZZY-RELATED 3-like | 57 | 2.2748823 | 135 | 7.25377 | -1.672939 | 6.03E-14 | 1.14E-14 | down |
| VIT_01s0137g00710.t01 | 1334 | GDSL esterase/lipase | 115 | 5.8213868 | 272 | 18.537171 | -1.670986 | 5.81E-27 | 6.50E-28 | down |
| VIT_07s0005g04760.t01 | 1410 | ribokinase-like | 77 | 3.687704 | 182 | 11.734988 | -1.670022 | 2.21E-18 | 3.40E-19 | down |
| VIT_13s0101g00220.t01 | 1863 | chloroplast DNA | 227680 | 8252.7078 | 538108 | 26259.531 | -1.669902 | 0 | 0 | down |
| VIT_18s0001g14080.t01 | 1440 | probable diphthine synthase-like | 42 | 1.9695692 | 99 | 6.2503318 | -1.666053 | 1.81E-10 | 4.30E-11 | down |
| VIT_00s0201g00080.t01 | 504 | WAT1-related protein | 14 | 1.8757802 | 33 | 5.952697 | -1.666053 | 0.0003194 | 0.00014082 | down |
| VIT_06s0009g02800.t01 | 573 | dihydropyrimidinase | 14 | 1.6499009 | 33 | 5.2358801 | -1.666053 | 0.0003194 | 0.00014082 | down |
| VIT_05s0124g00270.t01 | 1756 | Putative uncharacterized protein | 45 | 1.7305034 | 106 | 5.48797 | -1.665081 | 4.03E-11 | 9.12E-12 | down |
| VIT_17s0000g07900.t01 | 1982 | protease Do-like 9-like | 96 | 3.2707852 | 226 | 10.366572 | -1.66423 | 1.85E-22 | 2.41E-23 | down |
| VIT_08s0032g00010.t01 | 333 | maturase K, putative, expressed | 245 | 49.682826 | 576 | 157.25651 | -1.662301 | 1.51E-55 | 8.90E-57 | down |
| VIT_18s0001g08110.t01 | 2324 | Glutamyl-tRNA reductase | 1839 | 53.435521 | 4320 | 168.99661 | -1.661123 | 0 | 0 | down |
| VIT_05s0124g00090.t01 | 864 | transport protein GOT1B-like | 43 | 3.3607728 | 101 | 10.627669 | -1.66096 | 1.29E-10 | 3.03E-11 | down |
| VIT_18s0001g11040.t01 | 1142 | heme oxygenase 2 | 69 | 4.0800683 | 162 | 12.89672 | -1.660339 | 2.37E-16 | 3.97E-17 | down |
| VIT_12s0028g02640.t01 | 2255 | GTP-binding protein TypA/BipA homolog-like | 101 | 3.0245396 | 237 | 9.5550326 | -1.659545 | 2.08E-23 | 2.61E-24 | down |
| VIT_10s0003g04940.t01 | 1997 | 2-succinylbenzoate--CoA ligase, chloroplastic/peroxisomal-like | 61 | 2.0627007 | 143 | 6.5101103 | -1.658147 | 1.54E-14 | 2.84E-15 | down |
| VIT_01s0150g00550.t01 | 1563 | histone-lysine N-methyltransferase ASHR2-like | 64 | 2.7650656 | 150 | 8.7249441 | -1.657832 | 3.40E-15 | 6.09E-16 | down |
| VIT_01s0011g01650.t01 | 944 | probable FKBP-type peptidyl-prolyl cis-trans isomerase 7 | 38 | 2.7182916 | 89 | 8.5713333 | -1.656819 | 1.85E-09 | 4.76E-10 | down |
| VIT_07s0129g00680.t01 | 2234 | pentatricopeptide, putative, expressed | 59 | 1.7834186 | 138 | 5.6159895 | -1.654895 | 4.96E-14 | 9.38E-15 | down |
| VIT_18s0122g01240.t01 | 1628 | serine/threonine-protein kinase PBS1-like | 245 | 10.162396 | 573 | 31.998572 | -1.654767 | 6.51E-55 | 3.88E-56 | down |
| VIT_00s2472g00010.t01 | 638 | enhancer of mRNA-decapping protein 4-like | 18 | 1.9051811 | 42 | 5.9849287 | -1.651406 | 5.01E-05 | 1.98E-05 | down |
| VIT_01s0026g00430.t01 | 393 | Putative uncharacterized protein | 18 | 3.0928895 | 42 | 9.7159912 | -1.651406 | 5.01E-05 | 1.98E-05 | down |
| VIT_13s0084g00700.t01 | 800 | DNA-directed RNA polymerase III subunit RPC9-like | 30 | 2.5323032 | 70 | 7.9549678 | -1.651406 | 1.21E-07 | 3.60E-08 | down |
| VIT_04s0008g04150.t01 | 1256 | dehydration-responsive protein RD22-like | 33 | 1.7742252 | 77 | 5.5735443 | -1.651406 | 2.69E-08 | 7.54E-09 | down |
| VIT_00s0629g00010.t01 | 814 | NADH-ubiquinone oxidoreductase chain 4, putative, expressed | 39 | 3.2353751 | 91 | 10.163595 | -1.651406 | 1.32E-09 | 3.35E-10 | down |
| VIT_07s0129g01120.t01 | 1124 | small RNA degrading nuclease 3-like | 36 | 2.1628213 | 84 | 6.7942785 | -1.651406 | 5.93E-09 | 1.59E-09 | down |
| VIT_03s0038g03250.t01 | 1424 | uncharacterized protein ycf39 | 339 | 16.075858 | 789 | 50.37295 | -1.647754 | 8.40E-75 | 3.73E-76 | down |
| VIT_00s0252g00010.t01 | 1479 | DNA repair protein recA homolog 2 | 43 | 1.9632912 | 100 | 6.1469856 | -1.646605 | 2.09E-10 | 4.98E-11 | down |
| VIT_07s0031g00820.t01 | 1198 | methionine aminopeptidase 1D (MAP1D) | 262 | 14.768246 | 609 | 46.21584 | -1.645889 | 7.31E-58 | 4.14E-59 | down |
| VIT_15s0046g00460.t01 | 1286 | probable sugar phosphate/phosphate translocator | 81 | 4.2533243 | 188 | 13.290682 | -1.643752 | 1.49E-18 | 2.27E-19 | down |
| VIT_11s0016g04350.t01 | 1679 | tRNA (guanine-N(1)-)-methyltransferase | 53 | 2.1316192 | 123 | 6.6601619 | -1.643608 | 1.65E-12 | 3.37E-13 | down |
| VIT_04s0023g03030.t01 | 936 | probable salt tolerance-like protein | 103 | 7.4309753 | 239 | 23.214131 | -1.64338 | 2.76E-23 | 3.49E-24 | down |
| VIT_14s0006g00650.t01 | 1250 | WD repeat-containing protein 75-like | 47 | 2.5390561 | 109 | 7.9276936 | -1.642609 | 3.31E-11 | 7.44E-12 | down |
| VIT_02s0025g03690.t01 | 2406 | RNA-binding protein NOB1-like | 94 | 2.6382544 | 218 | 8.2374206 | -1.642609 | 2.55E-21 | 3.47E-22 | down |
| VIT_08s0007g08220.t01 | 304 | zinc finger CCCH domain-containing protein 30-like | 22 | 4.886901 | 51 | 15.252006 | -1.642007 | 7.87E-06 | 2.82E-06 | down |
| VIT_07s0005g00990.t01 | 439 | protein DEHYDRATION-INDUCED 19 homolog 3-like | 22 | 3.3840954 | 51 | 10.561754 | -1.642007 | 7.87E-06 | 2.82E-06 | down |
| VIT_19s0027g01950.t01 | 443 | RNA polymerase II C-terminal domain phosphatase-like 4 | 19 | 2.8962385 | 44 | 9.0298248 | -1.640518 | 3.55E-05 | 1.38E-05 | down |
| VIT_01s0011g05860.t01 | 1571 | aspartate-semialdehyde dehydrogenase-like | 315 | 13.540005 | 729 | 42.187298 | -1.63958 | 1.05E-68 | 5.10E-70 | down |
| VIT_06s0004g01370.t01 | 1093 | biotin--protein ligase, putative, expressed | 32 | 1.9770346 | 74 | 6.1551966 | -1.638467 | 6.12E-08 | 1.77E-08 | down |
| VIT_05s0029g01340.t01 | 1743 | Putative uncharacterized protein | 61 | 2.3632893 | 141 | 7.3544821 | -1.637828 | 4.06E-14 | 7.64E-15 | down |
| VIT_07s0031g00620.t01 | 2288 | zeaxanthin epoxidase | 864 | 25.500117 | 1997 | 79.351002 | -1.637745 | 2.19E-186 | 3.93E-188 | down |
| VIT_14s0066g01270.t01 | 1155 | GDSL esterase/lipase | 29 | 1.6955104 | 67 | 5.2737944 | -1.637122 | 2.75E-07 | 8.47E-08 | down |
| VIT_17s0000g02660.t01 | 909 | transcription repressor MYB6-like (MYBC2-L2), mRNA gb\|GQ903730.1\| Vitis vini | 29 | 2.1543614 | 67 | 6.7010258 | -1.637122 | 2.75E-07 | 8.47E-08 | down |
| VIT_06s0004g00580.t01 | 1646 | dihydrolipoyllysine-residue acetyltransferase component of acetoin cleaving system-l | 87 | 3.5692245 | 201 | 11.101882 | -1.637122 | 1.18E-19 | 1.72E-20 | down |
| VIT_18s0001g02550.t01 | 1518 | Delta-aminolevulinic acid dehydratase | 1580 | 70.286151 | 3650 | 218.60066 | -1.636985 | 0 | 0 | down |
| VIT_19s0014g04900.t01 | 1815 | (E)-beta-caryophyllene synthase | 55 | 2.0463057 | 127 | 6.3614697 | -1.636338 | 8.32E-13 | 1.67E-13 | down |
| VIT_05s0020g00440.t01 | 2762 | boron transporter | 453 | 11.075389 | 1046 | 34.430108 | -1.636313 | 6.73E-98 | 2.30E-99 | down |
| VIT_01s0011g03830.t01 | 1881 | metallo-beta-lactamase family protein, putative, expressed | 68 | 2.4412067 | 157 | 7.588243 | -1.636171 | 1.42E-15 | 2.47E-16 | down |
| VIT_06s0004g08310.t01 | 1660 | Putative uncharacterized protein | 52 | 2.1153376 | 120 | 6.5720904 | -1.635464 | 3.73E-12 | 7.85E-13 | down |
| VIT_10s0003g04670.t01 | 2559 | START domain containing protein, expressed | 335 | 8.8401364 | 772 | 27.426942 | -1.633453 | 2.55E-72 | 1.18E-73 | down |
| VIT_00s0888g00010.t01 | 234 | sphingoid long-chain bases kinase 1-like | 23 | 6.637376 | 53 | 20.591614 | -1.633372 | 5.58E-06 | 1.97E-06 | down |
| VIT_04s0069g01000.t01 | 1495 | integral membrane protein, putative, expressed | 93 | 4.2007438 | 214 | 13.013765 | -1.631322 | 9.41E-21 | 1.31E-21 | down |
| VIT_03s0063g02380.t01 | 719 | organ-specific protein P4-like | 40 | 3.7567781 | 92 | 11.632935 | -1.630647 | 1.52E-09 | 3.87E-10 | down |
| VIT_16s0022g02320.t01 | 2216 | phosphoglucomutase, putative, expressed | 311 | 9.4770916 | 715 | 29.333687 | -1.630042 | 7.57E-67 | 3.75E-68 | down |

| | | | | | | | | | | |
|---|---|---|---|---|---|---|---|---|---|---|
| VIT_07s0005g01430.t01 | 2560 | protein PROLIFERA-like | 77 | 2.0311182 | 177 | 6.2858451 | -1.629833 | 2.48E-17 | 4.00E-18 | down |
| VIT_08s0056g01530.t01 | 3040 | anthranilate phosphoribosyltransferase, putative, expressed | 77 | 1.7104153 | 177 | 5.2933432 | -1.629833 | 2.48E-17 | 4.00E-18 | down |
| VIT_07s0005g01790.t01 | 2224 | long chain acyl-CoA synthetase 1-like | 94 | 2.8541547 | 216 | 8.829769 | -1.629312 | 6.68E-21 | 9.22E-22 | down |
| VIT_14s0128g00480.t01 | 1251 | eukaryotic translation initiation factor 3 subunit J-like | 156 | 8.4207686 | 358 | 26.016932 | -1.627427 | 6.75E-34 | 6.25E-35 | down |
| VIT_19s0014g00410.t01 | 1218 | protein trichome birefringence-like 42 | 58 | 3.2156232 | 133 | 9.9273818 | -1.626315 | 2.98E-13 | 5.84E-14 | down |
| VIT_13s0019g02490.t01 | 4571 | cucumisin-like | 3860 | 57.024374 | 8831 | 175.64227 | -1.622989 | 0 | 0 | down |
| VIT_08s0007g08940.t01 | 2677 | nuclear pore complex protein | 181 | 4.5657765 | 414 | 14.059904 | -1.622655 | 7.02E-39 | 5.79E-40 | down |
| VIT_11s0016g03850.t01 | 1039 | protein Mpv17-like | 39 | 2.5347405 | 89 | 7.7876214 | -1.619345 | 3.45E-09 | 9.03E-10 | down |
| VIT_07s0005g01080.t01 | 868 | Putative uncharacterized protein | 25 | 1.9449334 | 57 | 5.9701536 | -1.618047 | 2.82E-06 | 9.61E-07 | down |
| VIT_07s0104g01150.t01 | 1483 | CUGBP Elav-like family member 5 | 93 | 4.234735 | 212 | 12.99646 | -1.617775 | 2.44E-20 | 3.46E-21 | down |
| VIT_18s0089g01190.t01 | 1343 | hypothetical protein | 43 | 2.1621055 | 98 | 6.6340759 | -1.617459 | 5.44E-10 | 1.34E-10 | down |
| VIT_05s0062g00610.t01 | 1083 | glycosyl hydrolases family 16, putative, expressed | 76 | 4.7388131 | 173 | 14.522722 | -1.615714 | 9.15E-17 | 1.51E-17 | down |
| VIT_14s0030g00650.t01 | 2067 | bifunctional 3-dehydroquinate dehydratase/shikimate dehydrogenase | 70 | 2.2868728 | 159 | 6.9933783 | -1.612613 | 1.86E-15 | 3.26E-16 | down |
| VIT_08s0007g01680.t01 | 2306 | neutral ceramidase-like | 203 | 5.94458 | 460 | 18.135474 | -1.609168 | 1.39E-42 | 1.05E-43 | down |
| VIT_00s0878g00020.t01 | 2891 | probable galactinol--sucrose galactosyltransferase 2-like | 353 | 8.2453872 | 799 | 25.12633 | -1.607541 | 3.91E-73 | 1.79E-74 | down |
| VIT_05s0020g01960.t01 | 2124 | transporter, monovalent cation:proton antiporter-2 family, putative, expressed | 168 | 5.3412046 | 380 | 16.265202 | -1.606552 | 2.81E-35 | 2.53E-36 | down |
| VIT_01s0150g00390.t01 | 3183 | MCM2 - Putative minichromosome maintenance MCM complex subunit 2, expresse | 166 | 3.5217287 | 375 | 10.710876 | -1.604721 | 9.06E-35 | 8.23E-36 | down |
| VIT_08s0007g03990.t01 | 2409 | glucomannan 4-beta-mannosyltransferase 9-like | 404 | 11.32476 | 912 | 34.41822 | -1.603692 | 4.63E-83 | 1.87E-84 | down |
| VIT_11s0052g01030.t01 | 1031 | mitochondrial outer membrane protein porin of 36 kDa-like | 144 | 9.4316629 | 325 | 28.658606 | -1.603384 | 3.10E-30 | 3.16E-31 | down |
| VIT_18s0041g01360.t01 | 1853 | protein TIC 40 | 308 | 11.224312 | 695 | 34.098852 | -1.603096 | 1.76E-63 | 9.05E-65 | down |
| VIT_12s0028g00970.t01 | 1509 | chloroplast DNA | 368 | 16.468082 | 830 | 50.005667 | -1.602419 | 1.39E-75 | 6.05E-77 | down |
| VIT_19s0014g00100.t01 | 860 | chalcone--flavonone isomerase-like | 158 | 12.406323 | 356 | 37.634133 | -1.600966 | 5.95E-33 | 5.66E-34 | down |
| VIT_15s0046g02860.t01 | 1296 | riboflavin biosynthesis protein RibD-like | 107 | 5.5752355 | 241 | 16.90606 | -1.600436 | 1.23E-22 | 1.60E-23 | down |
| VIT_17s0000g09920.t01 | 1074 | aminopeptidase M1 | 44 | 2.7665138 | 99 | 8.3803332 | -1.598938 | 6.20E-10 | 1.53E-10 | down |
| VIT_18s0001g08530.t01 | 1058 | Putative uncharacterized protein | 40 | 2.5530467 | 90 | 7.7336981 | -1.598938 | 3.94E-09 | 1.03E-09 | down |
| VIT_07s0197g00040.t01 | 1033 | LOB domain-containing protein 36-like | 32 | 2.0918672 | 72 | 6.3366913 | -1.598938 | 1.59E-07 | 4.77E-08 | down |
| VIT_04s0008g02860.t01 | 1071 | cation transport regulator-like protein 2 | 233 | 14.690984 | 524 | 44.480759 | -1.59825 | 6.62E-48 | 4.52E-49 | down |
| VIT_00s0179g00150.t01 | 2094 | heat stress transcription factor A-6b | 189 | 6.0949419 | 425 | 18.451965 | -1.59809 | 4.84E-39 | 3.99E-40 | down |
| VIT_15s0046g01070.t01 | 2035 | ubiquitin carboxyl-terminal hydrolase, family 1, putative, expressed | 169 | 5.6079836 | 380 | 16.976555 | -1.59799 | 5.17E-35 | 4.68E-36 | down |
| VIT_14s0060g00330.t01 | 1509 | aspartic proteinase oryzasin-1-like | 609 | 27.252886 | 1369 | 82.479227 | -1.597622 | 1.46E-123 | 3.96E-125 | down |
| VIT_19s0014g01300.t01 | 1814 | Putative uncharacterized protein | 81 | 3.0153115 | 182 | 9.1214625 | -1.596958 | 2.63E-17 | 4.23E-18 | down |
| VIT_04s0023g00080.t01 | 2353 | protein LUTEIN DEFICIENT 5 | 304 | 8.7244107 | 683 | 26.389378 | -1.596828 | 4.67E-62 | 2.47E-63 | down |
| VIT_13s0073g00550.t01 | 1537 | lysosomal beta glucosidase-like | 73 | 3.2072546 | 164 | 9.7006392 | -1.596741 | 1.07E-15 | 1.85E-16 | down |
| VIT_00s0271g00030.t01 | 1547 | (3S,6E)-nerolidol synthase 1 | 90 | 3.9285894 | 202 | 11.871113 | -1.595372 | 4.61E-19 | 6.86E-20 | down |
| VIT_02s0025g01040.t01 | 2323 | probable inactive receptor kinase | 312 | 9.0696354 | 700 | 27.395498 | -1.594822 | 1.83E-63 | 9.47E-65 | down |
| VIT_08s0007g05750.t01 | 2099 | probable methyltransferase PMT7-like | 87 | 2.798925 | 195 | 8.4460286 | -1.5934 | 2.07E-18 | 3.18E-19 | down |
| VIT_10s0003g04050.t01 | 1841 | Putative uncharacterized protein | 58 | 2.1274465 | 130 | 6.4197769 | -1.5934 | 1.24E-12 | 2.52E-13 | down |
| VIT_00s1596g00010.t01 | 790 | Putative uncharacterized protein | 195 | 16.668325 | 437 | 50.290357 | -1.593173 | 6.08E-40 | 4.92E-41 | down |
| VIT_15s0021g01280.t01 | 629 | cyclin-U4-1-like | 50 | 5.3678924 | 112 | 16.18817 | -1.592512 | 4.94E-11 | 1.13E-11 | down |
| VIT_11s0037g01180.t01 | 4929 | chloroplast DNA | 99487 | 1362.9878 | 222719 | 4107.9847 | -1.591658 | 0 | 0 | down |
| VIT_07s0129g00340.t01 | 1660 | GRAS family transcription factor domain containing protein, expressed | 85 | 3.4577635 | 190 | 10.40581 | -1.589478 | 6.62E-18 | 1.04E-18 | down |
| VIT_04s0008g01230.t01 | 1219 | harpin-induced protein 1 domain containing protein, expressed | 136 | 7.5338964 | 304 | 22.672544 | -1.589478 | 5.29E-28 | 5.70E-29 | down |
| VIT_09s0002g05200.t01 | 1630 | D-glycerate 3-kinase, chloroplastic-like | 230 | 9.528503 | 514 | 28.668561 | -1.589148 | 1.24E-46 | 8.71E-48 | down |
| VIT_05s0020g00690.t01 | 1497 | pentatricopeptide repeat-containing protein | 103 | 4.646221 | 230 | 13.96807 | -1.588003 | 2.03E-21 | 2.75E-22 | down |
| VIT_00s0370g00020.t01 | 1465 | clathrin light chain 1-like | 244 | 11.246999 | 544 | 33.759161 | -1.585739 | 3.82E-49 | 2.55E-50 | down |
| VIT_03s0017g01930.t01 | 1872 | cytochrome P450-like | 188 | 6.7816668 | 419 | 20.348788 | -1.585231 | 4.56E-38 | 3.83E-39 | down |
| VIT_15s0048g02410.t01 | 2566 | protein LHY-like | 228 | 6.0001573 | 508 | 17.998546 | -1.584808 | 6.38E-46 | 4.54E-47 | down |
| VIT_16s0013g01510.t01 | 1699 | WD repeat-containing protein WRAP73-like | 97 | 3.855341 | 216 | 11.558214 | -1.583988 | 4.11E-20 | 5.88E-21 | down |
| VIT_11s0016g03510.t01 | 3731 | elongation factor TS family protein | 730 | 13.21241 | 1625 | 39.596654 | -1.583485 | 1.21E-144 | 2.76E-146 | down |
| VIT_05s0077g00540.t01 | 1188 | Putative uncharacterized protein | 142 | 8.071539 | 316 | 24.18249 | -1.583047 | 6.64E-29 | 6.99E-30 | down |
| VIT_08s0007g00080.t01 | 1277 | Putative uncharacterized protein | 98 | 5.1822651 | 218 | 15.520152 | -1.582488 | 2.91E-20 | 4.14E-21 | down |
| VIT_00s0532g00060.t01 | 1424 | membrane protein, putative, expressed | 46 | 2.1813848 | 102 | 6.5120924 | -1.577877 | 5.00E-10 | 1.23E-10 | down |
| VIT_03s0038g00320.t01 | 1281 | Putative uncharacterized protein | 69 | 3.6373442 | 153 | 10.858571 | -1.577877 | 1.74E-14 | 3.21E-15 | down |
| VIT_14s0066g01960.t01 | 1166 | metalloendoproteinase 1 precursor, putative, expressed | 60 | 3.4748587 | 133 | 10.370112 | -1.577405 | 9.97E-13 | 2.02E-13 | down |
| VIT_19s0014g01310.t01 | 1471 | rab3 GTPase-activating protein non-catalytic subunit-like | 52 | 2.3871247 | 115 | 7.1074783 | -1.574064 | 3.97E-11 | 8.99E-12 | down |
| VIT_19s0014g01860.t01 | 1394 | RNA exonuclease 4-like | 38 | 1.8407943 | 84 | 5.4783135 | -1.573403 | 2.02E-08 | 5.59E-09 | down |
| VIT_03s0038g03430.t01 | 919 | expansin-like A2 | 775 | 56.946972 | 1713 | 169.46196 | -1.57327 | 5.16E-151 | 1.13E-152 | down |
| VIT_03s0088g00990.t01 | 1698 | CBS domain containing protein, expressed | 143 | 5.6869943 | 316 | 16.919198 | -1.572923 | 1.21E-28 | 1.28E-29 | down |
| VIT_04s0044g01530.t01 | 1409 | glucosyltransferase, putative, expressed | 62 | 2.9714275 | 137 | 8.8397493 | -1.572849 | 5.01E-13 | 9.94E-14 | down |
| VIT_02s0025g03030.t01 | 1384 | zinc finger, C3HC4 type domain containing protein, expressed | 58 | 2.8299343 | 128 | 8.4082236 | -1.571032 | 3.16E-12 | 6.62E-13 | down |
| VIT_15s0024g01170.t01 | 1201 | Putative uncharacterized protein | 131 | 7.365678 | 289 | 21.876871 | -1.570516 | 3.12E-26 | 3.57E-27 | down |
| VIT_08s0058g00670.t01 | 1766 | methyltransferase domain containing protein, expressed | 904 | 34.567039 | 1994 | 102.65139 | -1.570284 | 3.54E-175 | 6.66E-177 | down |
| VIT_16s0022g01800.t01 | 1464 | probable rRNA-processing protein EBP2 homolog | 49 | 2.2601614 | 108 | 6.7067644 | -1.569191 | 1.78E-10 | 4.22E-11 | down |
| VIT_08s0032g00780.t01 | 1753 | calcium-dependent protein kinase 20-like | 55 | 2.1186793 | 121 | 6.2752904 | -1.566517 | 1.41E-11 | 3.10E-12 | down |
| VIT_07s0031g00060.t01 | 2153 | probable beta-1,4-xylosyltransferase IRX14H-like | 261 | 8.186173 | 574 | 24.238081 | -1.566014 | 6.97E-51 | 4.50E-52 | down |
| VIT_19s0014g03350.t01 | 1016 | peptidyl-prolyl cis-trans isomerase CYP37 | 121 | 8.0422229 | 266 | 23.802266 | -1.565433 | 4.05E-24 | 4.96E-25 | down |
| VIT_16s0039g00520.t01 | 3179 | DNA-binding protein SMUBP-2-like | 111 | 2.3578539 | 244 | 6.9779792 | -1.565335 | 3.27E-22 | 4.30E-23 | down |
| VIT_07s0104g00400.t01 | 1505 | RNA exonuclease 4 | 56 | 2.512673 | 123 | 7.430174 | -1.564173 | 1.00E-11 | 2.17E-12 | down |
| VIT_17s0000g05690.t01 | 1073 | mitochondrial outer membrane protein porin of 36 kDa-like | 78 | 4.9088451 | 171 | 14.488611 | -1.561464 | 7.77E-16 | 1.34E-16 | down |
| VIT_04s0044g00710.t01 | 1686 | UTP--glucose-1-phosphate uridylyltransferase-like | 542 | 21.708317 | 1188 | 64.060341 | -1.561184 | 7.87E-104 | 2.54E-105 | down |
| VIT_18s0001g08580.t01 | 2847 | probable xyloglucan glycosyltransferase 12-like | 722 | 17.125142 | 1582 | 50.518376 | -1.560692 | 6.58E-138 | 1.55E-139 | down |
| VIT_00s0304g00050.t01 | 985 | protein SCO1 homolog 2, mitochondrial-like | 42 | 2.8793702 | 92 | 8.4914522 | -1.560258 | 5.05E-09 | 1.34E-09 | down |
| VIT_11s0052g00890.t01 | 1371 | afadin- and alpha-actinin-binding protein | 100 | 4.9254622 | 219 | 14.522354 | -1.559944 | 5.96E-20 | 8.57E-21 | down |
| VIT_06s0004g05070.t01 | 1052 | zinc transporter 2 | 37 | 2.3750373 | 81 | 7.000026 | -1.55941 | 4.54E-08 | 1.30E-08 | down |
| VIT_08s0007g06390.t01 | 1564 | enoyl-[acyl-carrier-protein] reductase [NADH] | 128 | 5.5265953 | 280 | 16.276149 | -1.558297 | 3.59E-25 | 4.24E-26 | down |
| VIT_05s0077g00060.t01 | 1613 | carboxyl-terminal-processing protease-like | 160 | 6.6983843 | 350 | 19.727136 | -1.558297 | 3.32E-31 | 3.30E-32 | down |
| VIT_08s0040g00390.t01 | 1460 | magnesium-protoporphyrin IX monomethylester [oxidative] cyclase | 6677 | 308.82537 | 14601 | 909.20145 | -1.557809 | 0 | 0 | down |
| VIT_00s2077g00020.t01 | 1362 | probable galactinol--sucrose galactosyltransferase 2-like | 86 | 4.263888 | 188 | 12.549058 | -1.557338 | 3.06E-17 | 4.94E-18 | down |
| VIT_18s0072g00090.t01 | 203 | UDP-galactose transporter 2-like | 38 | 12.640726 | 83 | 37.1717 | -1.556125 | 3.22E-08 | 9.07E-09 | down |
| VIT_00s0265g00080.t01 | 650 | 50S ribosomal protein L21, chloroplast precursor, putative, expressed | 49 | 5.0905788 | 107 | 14.96583 | -1.555771 | 2.83E-10 | 6.83E-11 | down |
| VIT_19s0090g00710.t01 | 812 | glycine-rich cell wall structural protein 1-like | 126 | 10.478496 | 275 | 30.789812 | -1.555021 | 1.14E-24 | 1.37E-25 | down |
| VIT_09s0002g02670.t01 | 1954 | RNA methyltransferase protein, putative, expressed | 148 | 5.1147169 | 323 | 15.028247 | -1.55495 | 8.68E-29 | 9.16E-30 | down |
| VIT_09s0002g04880.t01 | 2363 | glycosyl transferase, group 1 domain containing protein, expressed | 77 | 2.2004497 | 168 | 6.4636217 | -1.554544 | 1.75E-15 | 3.07E-16 | down |
| VIT_01s0146g00150.t01 | 1492 | Bcl-2-associated athanogene-like protein | 94 | 4.2544505 | 205 | 12.491524 | -1.553905 | 1.21E-18 | 1.83E-19 | down |
| VIT_17s0000g00930.t01 | 1329 | Putative uncharacterized protein | 100 | 5.0811201 | 218 | 14.912892 | -1.553342 | 9.49E-20 | 1.38E-20 | down |
| VIT_17s0000g01840.t01 | 1581 | 3-oxoacyl-[acyl-carrier-protein] reductase 1 | 286 | 12.215707 | 623 | 35.825029 | -1.552231 | 1.92E-54 | 1.15E-55 | down |
| VIT_05s0020g01440.t01 | 801 | Putative uncharacterized protein | 45 | 3.7937127 | 98 | 11.123051 | -1.55187 | 1.79E-09 | 4.59E-10 | down |
| VIT_05s0094g00820.t01 | 2321 | rhodanese-like domain containing protein, putative, expressed | 1303 | 37.909994 | 2832 | 110.92986 | -1.548998 | 1.33E-243 | 1.75E-245 | down |
| VIT_15s0024g00640.t01 | 1996 | RNA polymerase sigma factor rpoD-like | 569 | 19.250241 | 1236 | 56.297396 | -1.548192 | 1.09E-106 | 3.41E-108 | down |
| VIT_11s0016g04690.t01 | 1165 | Putative uncharacterized protein | 181 | 10.491488 | 393 | 30.668815 | -1.547553 | 1.42E-34 | 1.30E-35 | down |
| VIT_17s0000g04810.t01 | 1935 | DUF246 domain-containing protein | 457 | 15.948494 | 992 | 46.608065 | -1.547159 | 1.01E-85 | 3.94E-87 | down |
| VIT_14s0068g00810.t01 | 2587 | transcription-repair-coupling factor-like | 94 | 2.4536684 | 204 | 7.1690913 | -1.54685 | 1.92E-18 | 2.94E-19 | down |
| VIT_08s0040g01360.t01 | 1584 | histidyl-tRNA synthetase-like | 166 | 7.076807 | 360 | 20.662254 | -1.545827 | 1.05E-31 | 1.03E-32 | down |
| VIT_19s0027g00210.t01 | 1385 | DNA repair protein recA homolog 1 | 66 | 3.2179449 | 143 | 9.3867799 | -1.544491 | 3.18E-13 | 6.26E-14 | down |
| VIT_10s0003g00660.t01 | 852 | ATP synthase mitochondrial F1 complex assembly factor 1-like | 24 | 1.9021996 | 52 | 5.5487368 | -1.544491 | 1.63E-05 | 6.06E-06 | down |
| VIT_00s2887g00010.t01 | 194 | syntaxin-51-like | 18 | 6.2654926 | 39 | 18.276509 | -1.544491 | 0.00020807 | 8.93E-05 | down |
| VIT_09s0002g02340.t01 | 1337 | putative oxidoreductase GLYR1-like | 479 | 24.192934 | 1037 | 70.514385 | -1.543332 | 3.01E-89 | 1.13E-90 | down |

| | | | | | | | | | | |
|---|---|---|---|---|---|---|---|---|---|---|
| VIT_00s1238g00010.t01 | 372 | Putative uncharacterized protein | 43 | 7.8056659 | 93 | 22.728479 | -1.541908 | 5.67E-09 | 1.51E-09 | down |
| VIT_10s0003g04070.t01 | 1748 | putative hydrolase C777.06c-like | 111 | 4.2881108 | 240 | 12.48246 | -1.541488 | 2.10E-21 | 2.85E-22 | down |
| VIT_09s0054g00070.t01 | 563 | 60S ribosomal protein L7-4-like | 81 | 9.7154085 | 175 | 28.259211 | -1.540375 | 6.96E-16 | 1.20E-16 | down |
| VIT_07s0031g02040.t01 | 1135 | Putative uncharacterized protein | 126 | 7.49651 | 272 | 21.7873 | -1.539196 | 4.60E-24 | 5.65E-25 | down |
| VIT_18s0001g15710.t01 | 1192 | Putative uncharacterized protein | 199 | 11.273565 | 429 | 32.719858 | -1.537223 | 2.75E-37 | 2.35E-38 | down |
| VIT_08s0056g01150.t01 | 1224 | NADH-cytochrome b5 reductase, putative, expressed | 195 | 10.758151 | 420 | 31.195952 | -1.535929 | 1.75E-36 | 1.53E-37 | down |
| VIT_00s0733g00010.t01 | 1405 | ATP synthase, putative, expressed | 887 | 42.63161 | 1908 | 123.46175 | -1.534069 | 4.61E-162 | 9.48E-164 | down |
| VIT_10s0003g05770.t01 | 964 | oxidoreductase, short chain dehydrogenase/reductase family domain containing prote | 53 | 3.7126438 | 114 | 10.751231 | -1.533983 | 1.13E-10 | 2.64E-11 | down |
| VIT_07s0031g02480.t01 | 2926 | beta-galactosidase 3-like | 446 | 10.293071 | 959 | 29.797145 | -1.533501 | 7.64E-82 | 3.13E-83 | down |
| VIT_02s0025g01960.t01 | 537 | Putative uncharacterized protein | 20 | 2.5150125 | 43 | 7.2798854 | -1.53335 | 0.00010381 | 4.27E-05 | down |
| VIT_18s0001g00090.t01 | 1103 | Putative uncharacterized protein | 284 | 17.387105 | 610 | 50.278776 | -1.531932 | 2.48E-52 | 1.56E-53 | down |
| VIT_15s0048g01490.t01 | 1796 | cytochrome P450 76C4-like | 48 | 1.8047595 | 103 | 5.2138828 | -1.530552 | 1.00E-09 | 2.53E-10 | down |
| VIT_00s1547g00010.t01 | 790 | Putative uncharacterized protein | 186 | 15.899018 | 399 | 45.917282 | -1.5301 | 1.60E-34 | 1.46E-35 | down |
| VIT_17s0000g07800.t01 | 973 | thiol-disulphide oxidoreductase DCC, putative, expressed | 146 | 10.132683 | 313 | 29.24569 | -1.529208 | 2.79E-27 | 3.09E-28 | down |
| VIT_00s0332g00110.t01 | 1960 | Putative uncharacterized protein | 2810 | 96.813226 | 6022 | 279.32837 | -1.528686 | 0 | 0 | down |
| VIT_04s0008g00480.t01 | 1795 | pentatricopeptide, putative, expressed | 63 | 2.3700665 | 135 | 6.837537 | -1.528549 | 2.26E-12 | 4.68E-13 | down |
| VIT_07s0104g00610.t01 | 1487 | glutamine synthetase, catalytic domain containing protein, expressed | 205 | 9.309521 | 439 | 26.840087 | -1.527611 | 8.59E-38 | 7.26E-39 | down |
| VIT_03s0063g01100.t01 | 1286 | Putative uncharacterized protein | 142 | 7.456445 | 304 | 21.491315 | -1.527194 | 1.78E-26 | 2.02E-27 | down |
| VIT_16s0050g02520.t01 | 2001 | RNA polymerase sigma factor, putative, expressed | 166 | 5.6020302 | 355 | 16.129156 | -1.525649 | 1.06E-30 | 1.07E-31 | down |
| VIT_00s0854g00040.t01 | 795 | NADH-ubiquinone oxidoreductase chain 4, putative, expressed | 29 | 2.4632887 | 62 | 7.090142 | -1.525229 | 2.88E-06 | 9.84E-07 | down |
| VIT_08s0040g00640.t01 | 437 | chloroplast | 4580 | 707.73143 | 9785 | 2035.6812 | -1.524238 | 0 | 0 | down |
| VIT_08s0007g03780.t01 | 2030 | Putative uncharacterized protein | 88 | 2.9273259 | 188 | 8.419614 | -1.524171 | 9.75E-17 | 1.61E-17 | down |
| VIT_12s0028g03160.t01 | 590 | nuclear cap-binding protein subunit 2-like | 88 | 10.071986 | 188 | 28.969181 | -1.524171 | 9.75E-17 | 1.61E-17 | down |
| VIT_01s0146g00300.t01 | 579 | peptidase, T1 family, putative, expressed | 15 | 1.7494323 | 32 | 5.0246034 | -1.522123 | 0.00093945 | 0.000446 | down |
| VIT_19s0014g01330.t01 | 3403 | peptide-N4-(N-acetyl-beta-glucosaminyl)asparagine amidase A-like | 593 | 11.76731 | 1265 | 33.795506 | -1.522047 | 1.69E-106 | 5.31E-108 | down |
| VIT_07s0005g00940.t01 | 3337 | kinesin-4-like | 129 | 2.6104654 | 275 | 7.4921568 | -1.521074 | 6.47E-24 | 7.97E-25 | down |
| VIT_19s0014g01290.t01 | 1617 | phosphoesterase, putative, expressed | 61 | 2.5474417 | 130 | 7.3090967 | -1.520644 | 7.11E-12 | 1.53E-12 | down |
| VIT_06s0004g05040.t01 | 1487 | protein-S-isoprenylcysteine O-methyltransferase. tax, putative, expressed | 115 | 5.2224142 | 245 | 14.979092 | -1.520161 | 2.09E-21 | 2.83E-22 | down |
| VIT_18s0001g14730.t01 | 1256 | prostaglandin E synthase 2-like | 108 | 5.8065552 | 230 | 16.648249 | -1.519616 | 3.75E-20 | 5.35E-21 | down |
| VIT_07s0031g02450.t01 | 1011 | amino acid permease 2-like | 31 | 2.0705941 | 66 | 5.9350332 | -1.519211 | 1.44E-06 | 4.76E-07 | down |
| VIT_08s0007g07430.t01 | 1200 | probable galactose-1-phosphate uridyl transferase-like | 71 | 3.9954118 | 151 | 11.440001 | -1.517671 | 1.41E-13 | 2.72E-14 | down |
| VIT_00s0229g00160.t01 | 1123 | formyltetrahydrofolate deformylase-like | 71 | 4.2693625 | 151 | 12.2244 | -1.517671 | 1.41E-13 | 2.72E-14 | down |
| VIT_01s0010g01890.t01 | 1366 | DEAD-box ATP-dependent RNA helicase 10-like | 40 | 1.9773964 | 85 | 5.6571618 | -1.516476 | 4.04E-08 | 1.15E-08 | down |
| VIT_01s0127g00300.t01 | 2001 | CRS2-associated factor 2, chloroplast precursor, putative, expressed | 73 | 2.4635434 | 155 | 7.0423075 | -1.515313 | 7.02E-14 | 1.34E-14 | down |
| VIT_11s0052g01390.t01 | 2896 | aminotransferase, putative, expressed | 123 | 2.8680783 | 261 | 8.193554 | -1.514405 | 1.29E-22 | 1.68E-23 | down |
| VIT_13s0019g02390.t01 | 2085 | folylpolyglutamate synthase | 214 | 6.9309403 | 454 | 19.796124 | -1.514095 | 1.48E-38 | 1.23E-39 | down |
| VIT_06s0061g00010.t01 | 1963 | magnesium-chelatase D subunit | 373 | 12.831368 | 791 | 36.634187 | -1.513516 | 2.15E-66 | 1.08E-67 | down |
| VIT_12s0059g00300.t01 | 1803 | transcription initiation factor TFIID subunit 6-like | 186 | 6.9662918 | 394 | 19.866935 | -1.511906 | 1.59E-33 | 1.49E-34 | down |
| VIT_07s0031g02460.t01 | 1335 | Putative uncharacterized protein | 205 | 10.369481 | 434 | 29.555536 | -1.511085 | 8.49E-37 | 7.37E-38 | down |
| VIT_12s0028g00250.t01 | 849 | protein-S-isoprenylcysteine O-methyltransferase B-like | 35 | 2.7838434 | 74 | 7.9241813 | -1.509184 | 3.60E-07 | 1.12E-07 | down |
| VIT_02s0025g02550.t01 | 1496 | embryogenesis-associated protein EMB8-like | 315 | 14.218815 | 666 | 40.473709 | -1.509184 | 8.06E-56 | 4.73E-57 | down |
| VIT_15s0046g02890.t01 | 1132 | Putative uncharacterized protein | 44 | 2.6247666 | 93 | 7.4690763 | -1.508741 | 1.01E-08 | 2.73E-09 | down |
| VIT_18s0001g09070.t01 | 1284 | A-kinase anchor protein 17A-like | 63 | 3.313294 | 133 | 9.4170958 | -1.507016 | 5.61E-12 | 1.20E-12 | down |
| VIT_10s0003g04600.t01 | 2604 | cleavage and polyadenylation specificity factor CPSF30-like | 108 | 2.800704 | 228 | 7.9602048 | -1.507016 | 9.36E-20 | 1.35E-20 | down |
| VIT_13s0067g00080.t01 | 3602 | Putative uncharacterized protein | 172 | 3.2245505 | 363 | 9.1620633 | -1.506574 | 8.21E-31 | 8.24E-32 | down |
| VIT_00s0125g00060.t01 | 572 | mitochondrial genome | 4741 | 559.70395 | 10004 | 1590.0399 | -1.506327 | 0 | 0 | down |
| VIT_16s0098g01290.t01 | 1777 | major facilitator superfamily domain-containing protein | 642 | 24.396754 | 1354 | 69.272619 | -1.505596 | 3.94E-112 | 1.17E-113 | down |
| VIT_14s0108g01290.t01 | 1605 | Putative uncharacterized protein | 203 | 8.5409355 | 428 | 24.243711 | -1.505145 | 4.24E-36 | 3.75E-37 | down |
| VIT_05s0020g02880.t01 | 1563 | glucose-1-phosphate adenylyltransferase large subunit 1 | 362 | 15.639902 | 763 | 44.380882 | -1.504707 | 1.57E-63 | 8.09E-65 | down |
| VIT_18s0041g00730.t01 | 1436 | eukaryotic peptide chain release factor subunit 1-3-like | 227 | 10.674704 | 478 | 30.262432 | -1.503332 | 3.99E-40 | 3.21E-41 | down |
| VIT_15s0021g02450.t01 | 2341 | elongation factor G, mitochondrial-like | 124 | 3.5768828 | 261 | 10.136067 | -1.502723 | 2.28E-22 | 2.98E-23 | down |
| VIT_17s0000g06450.t01 | 1325 | ribulose-phosphate 3-epimerase | 2080 | 106.00635 | 4377 | 300.32469 | -1.502372 | 0 | 0 | down |
| VIT_04s0023g03610.t01 | 1290 | MLO17 protein (MLO17) | 49 | 2.5650203 | 103 | 7.2590182 | -1.500804 | 1.77E-09 | 4.54E-10 | down |
| VIT_04s0008g00860.t01 | 1334 | pollen-specific protein SF21-like | 98 | 4.9608339 | 206 | 14.039181 | -1.500804 | 7.51E-18 | 1.18E-18 | down |
| VIT_05s0062g00120.t01 | 731 | 40S ribosomal protein S8-like | 108 | 9.9767898 | 227 | 28.231818 | -1.500674 | 1.47E-19 | 2.15E-20 | down |
| VIT_18s0122g01250.t01 | 255 | serine/threonine-protein kinase PBS1-like | 59 | 15.624145 | 124 | 44.209121 | -1.500567 | 3.52E-11 | 7.94E-12 | down |
| VIT_02s0025g04680.t01 | 1723 | protein FIZZY-RELATED 2-like | 71 | 2.7826431 | 149 | 7.8619697 | -1.498435 | 3.51E-13 | 6.91E-14 | down |
| VIT_14s0108g01180.t01 | 1133 | Putative uncharacterized protein | 93 | 5.5429056 | 195 | 15.647144 | -1.497185 | 6.73E-17 | 1.10E-17 | down |
| VIT_05s0020g01090.t01 | 962 | transcription factor HY5-like | 63 | 4.4223175 | 132 | 12.474675 | -1.496128 | 8.80E-12 | 1.91E-12 | down |
| VIT_14s0006g01880.t01 | 1194 | ATP-dependent Clp protease proteolytic subunit 3 | 286 | 16.175069 | 599 | 45.609243 | -1.495554 | 1.19E-49 | 7.86E-51 | down |
| VIT_18s0001g14230.t01 | 1694 | HXK2 | 139 | 5.5409705 | 291 | 15.617444 | -1.494948 | 1.24E-24 | 1.49E-25 | down |
| VIT_10s0003g02730.t01 | 1842 | carboxyl-terminal-processing protease-like | 54 | 1.9796507 | 113 | 5.5772382 | -1.494305 | 3.13E-10 | 7.57E-11 | down |
| VIT_18s0001g14540.t01 | 2652 | DEAD-box ATP-dependent RNA helicase 28-like | 141 | 3.5902942 | 295 | 10.112973 | -1.494033 | 6.16E-25 | 7.34E-26 | down |
| VIT_01s0011g05610.t01 | 807 | caleosin related protein, putative, expressed | 33 | 2.7613716 | 69 | 7.7733089 | -1.493144 | 1.13E-06 | 3.71E-07 | down |
| VIT_11s0016g00560.t01 | 860 | peroxiredoxin | 3032 | 238.07577 | 6339 | 670.12014 | -1.492999 | 0 | 0 | down |
| VIT_11s0037g01190.t01 | 1323 | chloroplast DNA | 45 | 2.2968737 | 94 | 6.4594923 | -1.491749 | 1.12E-08 | 3.05E-09 | down |
| VIT_18s0001g02250.t01 | 1037 | ras-related protein, putative, expressed | 45 | 2.9303413 | 94 | 8.2409916 | -1.491749 | 1.12E-08 | 3.05E-09 | down |
| VIT_01s0010g02670.t01 | 1462 | 3-oxoacyl-reductase, chloroplast precursor, putative, expressed | 160 | 7.3902147 | 334 | 20.769664 | -1.49079 | 5.25E-28 | 5.66E-29 | down |
| VIT_00s0271g00070.t01 | 1271 | pentatricopeptide containing protein, putative, expressed | 46 | 2.4439748 | 96 | 6.8668262 | -1.490414 | 7.90E-09 | 2.13E-09 | down |
| VIT_05s0020g00810.t01 | 1224 | 50S ribosomal protein L3-2 | 139 | 7.6686307 | 290 | 21.540062 | -1.489982 | 1.95E-24 | 2.35E-25 | down |
| VIT_05s0020g02240.t01 | 1196 | Putative uncharacterized protein | 280 | 15.809251 | 584 | 44.392749 | -1.489555 | 3.77E-48 | 2.56E-49 | down |
| VIT_11s0052g01040.t01 | 381 | mitochondrial outer membrane protein porin 4 | 47 | 8.3302364 | 98 | 23.384682 | -1.489134 | 5.57E-09 | 1.48E-09 | down |
| VIT_09s0002g06380.t01 | 1461 | probable polygalacturonase | 106 | 4.8993684 | 221 | 13.752208 | -1.488996 | 7.33E-19 | 1.10E-19 | down |
| VIT_01s0010g03350.t01 | 1635 | putative ribosome biogenesis GTPase RsgA-like | 83 | 3.4280313 | 173 | 9.6196378 | -1.488602 | 5.35E-15 | 9.68E-16 | down |
| VIT_14s0128g00080.t01 | 1296 | GDSL esterase/lipase | 95 | 4.9499755 | 198 | 13.889626 | -1.488515 | 5.27E-17 | 8.61E-18 | down |
| VIT_07s0141g00720.t01 | 313 | fimbrin-like protein 2-like | 24 | 5.1778724 | 50 | 14.52299 | -1.487907 | 4.07E-05 | 1.59E-05 | down |
| VIT_19s0090g01720.t01 | 1944 | WRKY transcription factor 20 | 96 | 3.3347203 | 200 | 9.3532837 | -1.487907 | 3.72E-17 | 6.04E-18 | down |
| VIT_09s0070g00260.t01 | 286 | 30S ribosomal protein S17-like | 36 | 8.5000389 | 75 | 23.841062 | -1.487907 | 4.01E-07 | 1.25E-07 | down |
| VIT_08s0007g08020.t01 | 457 | Putative uncharacterized protein | 134 | 19.800358 | 279 | 55.503245 | -1.487046 | 1.74E-23 | 2.18E-24 | down |
| VIT_01s0011g03080.t01 | 1275 | peptidase, M24 family protein, putative, expressed | 271 | 14.353029 | 564 | 40.216039 | -1.486416 | 2.14E-46 | 1.51E-47 | down |
| VIT_14s0066g00160.t01 | 1144 | methyltransferase domain containing protein, expressed | 37 | 2.1840378 | 77 | 6.119206 | -1.486347 | 2.83E-07 | 8.71E-08 | down |
| VIT_04s0008g03890.t01 | 1263 | GTPase of unknown function domain containing protein, putative, expressed | 275 | 14.703265 | 572 | 41.173999 | -1.485597 | 5.32E-47 | 3.70E-48 | down |
| VIT_18s0001g01020.t01 | 1922 | ATPF0B, atpF; F-type H+-transporting ATPase subunit b | 365 | 12.824012 | 759 | 35.90201 | -1.485217 | 5.17E-62 | 2.74E-63 | down |
| VIT_14s0066g01410.t01 | 1624 | peptidyl-prolyl cis-trans isomerase, chloroplast precursor, putative, expressed | 682 | 28.35847 | 1418 | 79.381733 | -1.485027 | 4.84E-115 | 1.41E-116 | down |
| VIT_10s0003g03790.t01 | 622 | protein TIFY 5A (JASMONATE-ZIM-DOMAIN PROTEIN 8) | 76 | 8.2510202 | 158 | 23.093889 | -1.484867 | 9.64E-14 | 1.85E-14 | down |
| VIT_16s0098g01670.t01 | 967 | ribosomal L9, putative, expressed | 51 | 3.5614606 | 106 | 9.9657448 | -1.484509 | 1.39E-09 | 3.53E-10 | down |
| VIT_05s0029g00730.t01 | 2181 | methyltransferase domain containing protein, expressed | 81 | 2.5079207 | 168 | 7.0029978 | -1.481481 | 1.69E-14 | 3.12E-15 | down |
| VIT_17s0000g08790.t01 | 1570 | Putative uncharacterized protein | 110 | 4.7312672 | 228 | 13.202785 | -1.480544 | 2.85E-19 | 4.21E-20 | down |
| VIT_18s0001g12540.t01 | 912 | maf, putative, expressed | 70 | 5.1830768 | 145 | 14.454515 | -1.47964 | 1.22E-12 | 2.49E-13 | down |
| VIT_05s0077g00700.t01 | 1584 | ATP binding protein, putative, expressed | 142 | 6.0536542 | 294 | 16.874174 | -1.478939 | 1.69E-24 | 2.04E-25 | down |
| VIT_14s0108g01440.t01 | 1581 | Putative uncharacterized protein | 144 | 6.1505657 | 298 | 17.13621 | -1.478257 | 8.39E-25 | 1.00E-25 | down |
| VIT_04s0008g06920.t01 | 1726 | monogalactosyldiacylglycerol synthase, putative, expressed | 309 | 12.089327 | 639 | 33.658165 | -1.477223 | 5.88E-52 | 3.72E-53 | down |
| VIT_19s0085g00380.t01 | 1662 | phosphoglycerate kinase protein, putative, expressed | 8079 | 328.25476 | 16703 | 913.6794 | -1.476872 | 0 | 0 | down |
| VIT_06s0004g06730.t01 | 1350 | omega-3 fatty acid desaturase, chloroplast precursor, putative, expressed | 91 | 4.5518932 | 188 | 12.660605 | -1.475808 | 5.22E-16 | 8.92E-17 | down |
| VIT_00s0771g00020.t01 | 791 | Putative uncharacterized protein | 140 | 11.951874 | 289 | 33.216337 | -1.474656 | 5.30E-24 | 6.52E-25 | down |
| VIT_07s0005g01760.t01 | 1759 | glycerol-3-phosphate acyltransferase, putative, expressed | 1735 | 66.606725 | 3577 | 184.87725 | -1.472828 | 1.57E-285 | 1.86E-287 | down |
| VIT_17s0000g09620.t01 | 1307 | rhodanese family protein, putative, expressed | 217 | 11.211626 | 447 | 31.092977 | -1.471593 | 1.89E-36 | 1.65E-37 | down |

| | | | | | | | | | | |
|---|---|---|---|---|---|---|---|---|---|---|
| VIT_13s0067g00670.t01 | 2712 | nuclear pore complex protein NUP88 | 121 | 3.0128682 | 249 | 8.3471849 | -1.470152 | 9.71E-21 | 1.35E-21 | down |
| VIT_05s0020g01110.t01 | 1594 | pectinacetylesterase domain containing protein, expressed | 87 | 3.6856609 | 179 | 10.209279 | -1.469886 | 3.27E-15 | 5.85E-16 | down |
| VIT_10s0116g00600.t01 | 1035 | P21-Rho-binding domain containing protein, putative, expressed | 35 | 2.2835585 | 72 | 6.3244464 | -1.469655 | 8.88E-07 | 2.87E-07 | down |
| VIT_15s0048g02980.t01 | 985 | Putative uncharacterized protein | 1005 | 68.899215 | 2067 | 190.78078 | -1.469356 | 1.05E-164 | 2.10E-166 | down |
| VIT_06s0009g00240.t01 | 1530 | outer envelope protein of 80 kDa | 71 | 3.1336563 | 146 | 8.6754457 | -1.469091 | 1.35E-12 | 2.76E-13 | down |
| VIT_11s0016g03820.t01 | 1143 | chloroplast processing peptidase-like | 197 | 11.638699 | 405 | 32.213593 | -1.46874 | 5.00E-33 | 4.74E-34 | down |
| VIT_03s0017g01730.t01 | 840 | protein ABCI7 | 36 | 2.8940608 | 74 | 8.0090832 | -1.468542 | 6.28E-07 | 2.00E-07 | down |
| VIT_07s0104g00380.t01 | 1036 | protein PALE CRESS | 74 | 4.8234347 | 152 | 13.338722 | -1.467488 | 4.78E-13 | 9.47E-14 | down |
| VIT_14s0060g01080.t01 | 2357 | solute carrier family 40 member 1-like | 186 | 5.3289029 | 382 | 14.734458 | -1.467284 | 3.64E-31 | 3.62E-32 | down |
| VIT_03s0063g00390.t01 | 1408 | LAG1 longevity assurance homolog 2 | 38 | 1.822491 | 78 | 5.0364244 | -1.466488 | 3.13E-07 | 9.67E-08 | down |
| VIT_00s0203g00100.t01 | 2233 | probable serine/threonine-protein kinase abkC | 153 | 4.6268684 | 314 | 12.784134 | -1.466246 | 8.93E-26 | 1.04E-26 | down |
| VIT_01s0244g00030.t01 | 707 | acyl carrier protein 4 | 957 | 91.406476 | 1964 | 252.55295 | -1.466218 | 4.82E-156 | 1.02E-157 | down |
| VIT_16s0039g00380.t01 | 500 | chloroplast DNA | 5143 | 694.5939 | 10545 | 1917.3745 | -1.464891 | 0 | 0 | down |
| VIT_05s0102g00430.t01 | 1389 | Putative uncharacterized protein | 61 | 2.9655963 | 125 | 8.1815981 | -1.46406 | 6.77E-11 | 1.56E-11 | down |
| VIT_05s0124g00560.t01 | 2086 | probable UDP-sugar transporter protein SLC35A5-like | 163 | 5.2766434 | 334 | 14.556687 | -1.46399 | 2.73E-27 | 3.02E-28 | down |
| VIT_19s0014g03160.t01 | 1481 | protochlorophyllide oxidoreductase | 9463 | 431.47757 | 19387 | 1190.1068 | -1.463734 | 0 | 0 | down |
| VIT_08s0032g00290.t01 | 1395 | RNA recognition motif containing protein, putative, expressed | 165 | 7.987193 | 338 | 22.027888 | -1.463571 | 1.36E-27 | 1.49E-28 | down |
| VIT_10s0071g01020.t01 | 1474 | lipoyl synthase | 62 | 2.8403944 | 127 | 7.833153 | -1.463502 | 4.78E-11 | 1.09E-11 | down |
| VIT_13s0064g01360.t01 | 1613 | chaperone protein dnaJ 1 | 166 | 6.9495737 | 340 | 19.163504 | -1.463365 | 9.63E-28 | 1.05E-28 | down |
| VIT_07s0130g00410.t01 | 1963 | T-complex protein 1 subunit gamma-like | 197 | 6.776889 | 403 | 18.664447 | -1.461598 | 1.23E-32 | 1.17E-33 | down |
| VIT_13s0067g01150.t01 | 1296 | gibberellin 2-beta-dioxygenase | 66 | 3.4389303 | 135 | 9.4701998 | -1.461435 | 1.19E-11 | 2.60E-12 | down |
| VIT_07s0104g01240.t01 | 2002 | protease Do-like 2 | 327 | 11.029812 | 668 | 30.334914 | -1.459571 | 2.55E-53 | 1.56E-54 | down |
| VIT_04s0044g01450.t01 | 848 | Putative uncharacterized protein | 141 | 11.228137 | 288 | 30.876425 | -1.459387 | 1.43E-23 | 1.79E-24 | down |
| VIT_14s0060g00850.t01 | 889 | psbP; photosystem II oxygen-evolving enhancer protein 2 | 192 | 14.584244 | 392 | 40.088027 | -1.458761 | 1.10E-31 | 1.08E-32 | down |
| VIT_17s0000g04680.t01 | 1257 | protease 4-like | 612 | 32.877636 | 1249 | 90.335309 | -1.458183 | 9.69E-99 | 3.30E-100 | down |
| VIT_00s0283g00070.t01 | 1609 | (+)-neomenthol dehydrogenase-like | 50 | 2.0984489 | 102 | 5.7633434 | -1.457583 | 4.81E-09 | 1.27E-09 | down |
| VIT_02s0033g00980.t01 | 3247 | chloroplast DNA | 482 | 10.024188 | 983 | 27.52337 | -1.457172 | 8.05E-78 | 3.42E-79 | down |
| VIT_06s0061g00040.t01 | 1752 | hexokinase-3-like | 103 | 3.9699731 | 210 | 10.897216 | -1.456758 | 1.96E-17 | 3.13E-18 | down |
| VIT_14s0066g02240.t01 | 990 | serine/threonine-protein phosphatase PP2A catalytic subunit-like | 159 | 10.84542 | 324 | 29.753646 | -1.455981 | 2.69E-26 | 3.07E-27 | down |
| VIT_13s0067g00390.t01 | 1852 | peptidase M50 family protein, putative, expressed | 484 | 17.647729 | 986 | 48.402334 | -1.455594 | 6.25E-78 | 2.66E-79 | down |
| VIT_11s0149g00040.t01 | 1994 | adenylate kinase family protein, putative, expressed | 139 | 4.707324 | 283 | 12.903028 | -1.454731 | 4.47E-23 | 5.70E-24 | down |
| VIT_05s0077g01450.t01 | 1155 | GATA transcription factor 16-like | 309 | 18.065956 | 629 | 49.510696 | -1.454467 | 5.19E-50 | 3.40E-51 | down |
| VIT_10s0003g01760.t01 | 1027 | pentatricopeptide repeat-containing protein | 29 | 1.9068301 | 59 | 5.2229028 | -1.453676 | 1.12E-05 | 4.07E-06 | down |
| VIT_14s0030g01650.t01 | 1694 | Putative uncharacterized protein | 697 | 27.784579 | 1418 | 76.101497 | -1.45364 | 1.83E-111 | 5.52E-113 | down |
| VIT_04s0008g05010.t01 | 1561 | Putative uncharacterized protein | 3037 | 131.37912 | 6178 | 359.81178 | -1.453506 | 0 | 0 | down |
| VIT_00s0245g00050.t01 | 1388 | oleoyl-acyl carrier protein thioesterase | 444 | 21.601203 | 903 | 59.146446 | -1.45318 | 2.78E-71 | 1.31E-72 | down |
| VIT_12s0034g02120.t01 | 1091 | mitochondrial genome | 239 | 14.793046 | 486 | 40.498775 | -1.452959 | 8.18E-39 | 6.76E-40 | down |
| VIT_08s0007g02230.t01 | 1370 | thylakoid ADP,ATP carrier protein | 184 | 9.0694656 | 374 | 24.818836 | -1.452346 | 4.36E-30 | 4.47E-31 | down |
| VIT_05s0124g00280.t01 | 1645 | solute carrier family 35 member F1-like | 63 | 2.586182 | 128 | 7.0741529 | -1.451734 | 5.26E-11 | 1.20E-11 | down |
| VIT_06s0004g04390.t01 | 1350 | UDP-glucuronic acid decarboxylase 1-like | 163 | 8.1533912 | 331 | 22.290746 | -1.450973 | 1.04E-26 | 1.17E-27 | down |
| VIT_19s0015g01160.t01 | 2471 | phosphatidylinositol/phosphatidylcholine transfer protein SFH13 | 203 | 5.5476332 | 412 | 15.158452 | -1.450178 | 5.73E-33 | 5.45E-34 | down |
| VIT_13s0019g03350.t01 | 1467 | sedoheptulose-1,7-bisphosphatase | 2837 | 130.59113 | 5757 | 356.7767 | -1.449965 | 0 | 0 | down |
| VIT_03s0091g01290.t01 | 1674 | serine carboxypeptidase-like 18-like | 137 | 5.5264921 | 278 | 15.09801 | -1.449922 | 1.40E-22 | 1.82E-23 | down |
| VIT_17s0000g07700.t01 | 1114 | ribosomal RNA small subunit methyltransferase G-like | 35 | 2.1216185 | 71 | 5.7943341 | -1.449478 | 1.38E-06 | 4.57E-07 | down |
| VIT_02s0033g01030.t01 | 1620 | benzyl alcohol O-benzoyltransferase-like | 287 | 11.963309 | 582 | 32.661667 | -1.448982 | 4.27E-46 | 3.03E-47 | down |
| VIT_01s0026g00530.t01 | 1153 | WAT1-related protein | 36 | 2.1084225 | 73 | 5.7560416 | -1.448913 | 9.77E-07 | 3.18E-07 | down |
| VIT_09s0054g00720.t01 | 1660 | tetratricopeptide repeat containing protein, putative, expressed | 74 | 3.0102882 | 150 | 8.215113 | -1.448379 | 1.16E-12 | 2.36E-13 | down |
| VIT_04s0008g01740.t01 | 1355 | thermospermine synthase ACAULIS5-like | 40 | 1.993449 | 81 | 5.4347065 | -1.446935 | 2.43E-07 | 7.45E-08 | down |
| VIT_11s0037g00520.t01 | 1228 | translation initiation factor IF-3-like | 320 | 17.596895 | 648 | 47.974119 | -1.446935 | 4.21E-51 | 2.71E-52 | down |
| VIT_08s0007g00530.t01 | 1380 | NAD dependent epimerase/dehydratase family protein, putative, expressed | 1329 | 65.032483 | 2691 | 177.28214 | -1.446815 | 2.52E-209 | 3.91E-211 | down |
| VIT_05s0077g00450.t01 | 1433 | 30S ribosomal protein S1 | 1651 | 77.801026 | 3340 | 211.89985 | -1.445521 | 2.66E-259 | 3.33E-261 | down |
| VIT_18s0122g01370.t01 | 2873 | pre-rRNA-processing protein TSR1 homolog-like | 218 | 5.123955 | 441 | 13.955112 | -1.445464 | 4.74E-35 | 4.28E-36 | down |
| VIT_12s0034g02180.t01 | 1355 | aminomethyltransferase-like | 89 | 4.4354241 | 180 | 12.077126 | -1.445133 | 6.19E-15 | 1.12E-15 | down |
| VIT_18s0001g01940.t01 | 818 | Putative uncharacterized protein | 234 | 19.317325 | 473 | 52.570028 | -1.444345 | 1.78E-37 | 1.51E-38 | down |
| VIT_12s0059g02010.t01 | 1234 | formimidoyltransferase-cyclodeaminase-like | 329 | 18.003842 | 665 | 48.993319 | -1.44428 | 2.84E-52 | 1.78E-53 | down |
| VIT_01s0010g02740.t01 | 1673 | 4-coumarate--CoA ligase-like 9 | 143 | 5.7719763 | 289 | 15.704795 | -1.444068 | 2.70E-23 | 3.41E-24 | down |
| VIT_07s0031g00110.t01 | 1183 | protein IRX15-LIKE | 52 | 2.9682675 | 105 | 8.0692826 | -1.442819 | 3.74E-09 | 9.81E-10 | down |
| VIT_13s0019g04200.t01 | 2621 | patatin group A-3-like | 266 | 6.8532892 | 537 | 18.626774 | -1.442509 | 2.50E-42 | 1.90E-43 | down |
| VIT_18s0001g11990.t01 | 1856 | methyltransferase-like protein 2-like | 56 | 2.0374854 | 113 | 5.5351685 | -1.441838 | 9.28E-10 | 2.33E-10 | down |
| VIT_12s0057g00200.t01 | 857 | vesicle-associated protein 4-2-like | 56 | 4.4125704 | 113 | 11.987483 | -1.441838 | 9.28E-10 | 2.33E-10 | down |
| VIT_04s0008g05510.t01 | 1885 | Putative uncharacterized protein | 1061 | 38.009178 | 2138 | 103.11616 | -1.439851 | 2.88E-165 | 5.71E-167 | down |
| VIT_19s0090g01230.t01 | 1876 | 65-kDa microtubule-associated protein 1-like | 652 | 23.46925 | 1313 | 63.63005 | -1.438937 | 9.96E-102 | 3.30E-103 | down |
| VIT_10s0003g00600.t01 | 1106 | GDSL esterase/lipase | 149 | 9.0973643 | 300 | 24.660195 | -1.438664 | 5.19E-24 | 6.38E-25 | down |
| VIT_18s0122g00330.t01 | 2057 | Putative uncharacterized protein | 454 | 14.904109 | 914 | 40.396364 | -1.438515 | 4.58E-71 | 2.17E-72 | down |
| VIT_08s0007g01450.t01 | 2082 | Putative uncharacterized protein | 2774 | 89.97258 | 5582 | 243.74711 | -1.437828 | 0 | 0 | down |
| VIT_13s0019g03330.t01 | 2024 | adenylosuccinate lyase-like | 95 | 3.1695495 | 191 | 8.5793272 | -1.436587 | 1.20E-15 | 2.08E-16 | down |
| VIT_12s0028g01740.t01 | 1270 | Putative uncharacterized protein | 325 | 17.28081 | 653 | 46.745503 | -1.435657 | 6.73E-51 | 4.34E-52 | down |
| VIT_18s0001g08550.t01 | 2002 | squalene epoxidase | 612 | 20.642952 | 1228 | 55.76538 | -1.43372 | 1.10E-94 | 3.90E-96 | down |
| VIT_18s0072g00860.t01 | 1177 | Putative uncharacterized protein | 174 | 9.9829117 | 349 | 26.957483 | -1.433153 | 1.32E-27 | 1.44E-28 | down |
| VIT_14s0006g03240.t01 | 1308 | E3 ubiquitin-protein ligase HERC2-like | 204 | 10.531903 | 409 | 28.427976 | -1.432545 | 3.70E-32 | 3.59E-33 | down |
| VIT_08s0217g00020.t01 | 1432 | proliferation-associated protein 2G4-like | 425 | 20.041506 | 852 | 54.091241 | -1.432404 | 6.90E-66 | 3.47E-67 | down |
| VIT_07s0005g06110.t01 | 1398 | Putative uncharacterized protein | 228 | 11.013164 | 457 | 29.719356 | -1.432174 | 8.45E-36 | 7.50E-37 | down |
| VIT_16s0013g00370.t01 | 328 | Putative uncharacterized protein | 23 | 4.7352012 | 46 | 12.750123 | -1.429013 | 0.00014055 | 5.90E-05 | down |
| VIT_04s0069g00790.t01 | 2452 | probable cyclic nucleotide-gated ion channel 14-like | 84 | 2.3133602 | 168 | 6.2290123 | -1.429013 | 8.57E-14 | 1.64E-14 | down |
| VIT_19s0015g01250.t01 | 1531 | Putative uncharacterized protein | 43 | 1.8966086 | 86 | 5.1068562 | -1.429013 | 1.33E-07 | 3.97E-08 | down |
| VIT_19s0014g00180.t01 | 1182 | ribokinase-like | 71 | 4.0562556 | 142 | 10.921977 | -1.429013 | 7.88E-12 | 1.70E-12 | down |
| VIT_12s0059g01140.t01 | 1174 | Putative uncharacterized protein | 34 | 1.9556686 | 68 | 5.2658828 | -1.429013 | 3.04E-06 | 1.04E-06 | down |
| VIT_08s0007g00930.t01 | 1596 | probable E3 ubiquitin-protein ligase HERC3-like | 159 | 6.7274221 | 318 | 18.114427 | -1.429013 | 3.82E-25 | 4.53E-26 | down |
| VIT_08s0007g03030.t01 | 537 | ubiquitin-60S ribosomal protein L40-like | 134 | 16.850584 | 268 | 45.372309 | -1.429013 | 2.33E-21 | 3.16E-22 | down |
| VIT_17s0000g00330.t01 | 1657 | transcription factor ICE1-like | 85 | 3.4640237 | 170 | 9.327318 | -1.429013 | 6.05E-14 | 1.15E-14 | down |
| VIT_16s0022g00760.t01 | 1060 | GRF zinc finger family protein, expressed | 30 | 1.9111723 | 60 | 5.1460708 | -1.429013 | 1.22E-05 | 4.49E-06 | down |
| VIT_01s0011g03500.t01 | 1175 | homocysteine S-methyltransferase 1-like | 89 | 5.1148934 | 178 | 13.772491 | -1.429013 | 1.50E-14 | 2.76E-15 | down |
| VIT_11s0078g00380.t01 | 930 | isoamylase 1 | 27 | 1.9604928 | 54 | 5.2788726 | -1.429013 | 3.49E-05 | 1.35E-05 | down |
| VIT_13s0067g01800.t01 | 1761 | multiple inositol polyphosphate phosphatase 1-like | 68 | 2.6075581 | 136 | 7.0211771 | -1.429013 | 2.23E-11 | 4.97E-12 | down |
| VIT_10s0003g01460.t01 | 1934 | nucleobase-ascorbate transporter 2-like | 102 | 3.5614606 | 204 | 9.589679 | -1.429013 | 1.62E-16 | 2.71E-17 | down |
| VIT_05s0020g03190.t01 | 1047 | phosphate metabolism protein 8-like | 686 | 44.244763 | 1370 | 118.9609 | -1.426909 | 8.79E-105 | 2.80E-106 | down |
| VIT_16s0100g00380.t01 | 1093 | CBF4 transcription factor | 921 | 56.901526 | 1839 | 152.96495 | -1.426662 | 2.54E-140 | 5.95E-142 | down |
| VIT_02s0025g00210.t01 | 1848 | fringe-related protein, putative, expressed | 234 | 8.5506343 | 467 | 22.974459 | -1.425928 | 2.50E-36 | 2.20E-37 | down |
| VIT_11s0016g02960.t01 | 2106 | glucan endo-1,3-beta-glucosidase 13-like | 224 | 7.1824745 | 446 | 19.253375 | -1.422558 | 1.28E-34 | 1.17E-35 | down |
| VIT_06s0004g02120.t01 | 1927 | 2-oxoglutarate/malate translocator | 2840 | 99.522452 | 5653 | 266.70284 | -1.422139 | 0 | 0 | down |
| VIT_00s0333g00020.t01 | 1414 | Putative uncharacterized protein | 190 | 9.0737881 | 378 | 24.303721 | -1.4214 | 1.85E-29 | 1.91E-30 | down |
| VIT_08s0040g00160.t01 | 1275 | Putative uncharacterized protein | 85 | 4.5018724 | 169 | 12.050551 | -1.420502 | 9.38E-14 | 1.80E-14 | down |
| VIT_07s0031g01190.t01 | 2456 | formation of crista junctions protein 1-like | 161 | 4.426719 | 320 | 11.845462 | -1.420025 | 4.57E-25 | 5.43E-26 | down |
| VIT_09s0002g02240.t01 | 1883 | chaperonin CPN60-like 2 | 74 | 2.6537857 | 147 | 7.097369 | -1.419232 | 4.32E-12 | 9.12E-13 | down |
| VIT_14s0066g01040.t01 | 1611 | DNA (cytosine-5)-methyltransferase DRM2-like | 73 | 3.0599319 | 145 | 8.1828169 | -1.419098 | 6.10E-12 | 1.30E-12 | down |
| VIT_14s0068g02130.t01 | 2396 | fidgetin-like protein 1-like | 71 | 2.001041 | 141 | 5.3501095 | -1.418818 | 1.22E-11 | 2.66E-12 | down |
| VIT_05s0020g00780.t01 | 1111 | rRNA-processing protein fcf2-like | 68 | 4.1331322 | 135 | 11.047146 | -1.418366 | 3.46E-11 | 7.79E-12 | down |

| | | | | | | | | | | |
|---|---|---|---|---|---|---|---|---|---|---|
| VIT_02s0033g01350.t01 | 1435 | TIM21-like protein | 133 | 6.2587007 | 264 | 16.725627 | -1.418125 | 7.94E-21 | 1.10E-21 | down |
| VIT_00s1707g00010.t01 | 644 | mitochondrial genome | 329 | 34.498044 | 653 | 92.184454 | -1.418009 | 5.60E-50 | 3.68E-51 | down |
| VIT_14s0030g02190.t01 | 4237 | OsFtsH1  FtsH protease, homologue of AtFtsH1/5, expressed | 189 | 3.0122276 | 375 | 8.0464289 | -1.417518 | 4.05E-29 | 4.24E-30 | down |
| VIT_03s0038g00740.t01 | 1476 | cell division inhibitor, putative, expressed | 240 | 10.980177 | 476 | 29.319122 | -1.416941 | 1.15E-36 | 9.98E-38 | down |
| VIT_14s0006g01240.t01 | 1189 | 5-AMP-activated protein kinase beta-1 subunit-related, putative, expressed | 111 | 6.3041359 | 220 | 16.821751 | -1.415957 | 1.69E-17 | 2.70E-18 | down |
| VIT_01s0026g00820.t01 | 2814 | IAA-amino acid hydrolase ILR1-like 4 | 233 | 5.5913448 | 461 | 14.893858 | -1.41345 | 2.04E-35 | 1.83E-36 | down |
| VIT_00s0179g00100.t01 | 3032 | ETO1-like protein 1-like | 508 | 11.314073 | 1005 | 30.134725 | -1.413309 | 5.18E-76 | 2.25E-77 | down |
| VIT_16s0098g01780.t01 | 2240 | soluble starch synthase, chloroplastic/amyloplastic-like | 476 | 14.349718 | 941 | 38.191963 | -1.412247 | 3.68E-71 | 1.74E-72 | down |
| VIT_14s0108g00380.t01 | 1587 | probable receptor-like protein kinase | 124 | 5.2762966 | 245 | 14.03523 | -1.411455 | 2.82E-19 | 4.16E-20 | down |
| VIT_07s0005g01670.t01 | 934 | glycine-rich cell wall structural protein | 70 | 5.0609915 | 138 | 13.432677 | -1.408255 | 2.66E-11 | 5.96E-12 | down |
| VIT_08s0007g07960.t01 | 1061 | chaperone protein dnaJ 6 | 34 | 2.1639538 | 67 | 5.7410297 | -1.40764 | 4.71E-06 | 1.65E-06 | down |
| VIT_09s0002g06060.t01 | 2321 | WD repeat-containing protein 43 | 67 | 1.9493243 | 132 | 5.1704598 | -1.407318 | 7.56E-11 | 1.74E-11 | down |
| VIT_07s0104g00660.t01 | 2030 | oxidoreductase, 2OG-Fe oxygenase family protein, putative, expressed | 133 | 4.4242539 | 262 | 11.733717 | -1.407154 | 1.89E-20 | 2.66E-21 | down |
| VIT_04s0043g00560.t01 | 2075 | carboxyl-terminal peptidase, putative, expressed | 264 | 8.5915252 | 520 | 22.783247 | -1.406987 | 1.50E-39 | 1.22E-40 | down |
| VIT_08s0040g01260.t01 | 1766 | Putative uncharacterized protein | 63 | 2.4089861 | 124 | 6.3835367 | -1.40593 | 3.03E-10 | 7.31E-11 | down |
| VIT_03s0038g00710.t01 | 463 | chloroplast | 567 | 82.696382 | 1116 | 219.13592 | -1.40593 | 1.18E-83 | 4.73E-85 | down |
| VIT_14s0066g01800.t01 | 3110 | phosphatidylinositolglycan class N family protein, expressed | 93 | 2.0193286 | 183 | 5.3495971 | -1.405555 | 1.38E-14 | 2.53E-15 | down |
| VIT_05s0094g01510.t01 | 2264 | Putative uncharacterized protein | 184 | 5.4881484 | 362 | 14.536589 | -1.405297 | 8.60E-28 | 9.33E-29 | down |
| VIT_12s0034g01000.t01 | 984 | mRNA turnover protein 4 homolog-like | 182 | 12.489951 | 358 | 33.076405 | -1.405035 | 1.73E-27 | 1.89E-28 | down |
| VIT_07s0104g00510.t01 | 1406 | NAD dependent epimerase/dehydratase family protein, putative, expressed | 2437 | 117.04548 | 4793 | 309.92205 | -1.404836 | 0 | 0 | down |
| VIT_05s0049g01930.t01 | 1236 | RNA recognition motif containing protein, putative, expressed | 571 | 31.196228 | 1123 | 82.602208 | -1.404809 | 4.53E-84 | 1.80E-85 | down |
| VIT_14s0083g00950.t01 | 677 | U3 small nucleolar ribonucleoprotein protein IMP3-like | 90 | 8.9771459 | 177 | 23.769222 | -1.404766 | 3.92E-14 | 7.37E-15 | down |
| VIT_16s0050g00030.t01 | 1044 | cleavage and polyadenylation specificity factor subunit 5-like | 144 | 9.3142188 | 283 | 24.64429 | -1.403747 | 6.34E-22 | 8.40E-23 | down |
| VIT_14s0068g01640.t01 | 1140 | 60S ribosomal protein L7a-like | 454 | 26.892764 | 892 | 71.136153 | -1.403365 | 7.25E-67 | 3.58E-68 | down |
| VIT_11s0016g02490.t01 | 1378 | pollen-specific protein SF21-like | 170 | 8.3307509 | 334 | 22.035739 | -1.403327 | 1.13E-25 | 1.32E-26 | down |
| VIT_05s0051g00680.t01 | 747 | glycine-rich cell wall structural protein 2-like | 449 | 40.589171 | 882 | 107.34414 | -1.403077 | 4.15E-66 | 2.08E-67 | down |
| VIT_12s0028g02520.t01 | 1713 | TCP family transcription factor, putative, expressed | 222 | 8.7514508 | 436 | 23.139795 | -1.402782 | 3.53E-33 | 3.33E-34 | down |
| VIT_06s0004g02640.t01 | 2595 | receptor-like serine/threonine-protein kinase NCRK-like | 109 | 2.8364399 | 214 | 7.4973327 | -1.402296 | 8.11E-17 | 1.33E-17 | down |
| VIT_08s0007g06010.t01 | 429 | Beta-1,3-glucanase | 27 | 4.2500194 | 53 | 11.231789 | -1.402046 | 5.40E-05 | 2.14E-05 | down |
| VIT_01s0010g00680.t01 | 2598 | heat shock protein 90 | 910 | 23.653025 | 1785 | 62.46395 | -1.400999 | 1.45E-132 | 3.61E-134 | down |
| VIT_09s0002g01450.t01 | 1302 | Putative uncharacterized protein | 100 | 5.186489 | 196 | 13.685966 | -1.399867 | 1.86E-15 | 3.27E-16 | down |
| VIT_19s0085g00680.t01 | 1142 | Putative uncharacterized protein | 95 | 5.6174853 | 186 | 14.807346 | -1.398317 | 1.06E-14 | 1.93E-15 | down |
| VIT_08s0007g01570.t01 | 1431 | fructose-1,6-bisphosphatase | 2595 | 122.45659 | 5069 | 322.04238 | -1.39498 | 0 | 0 | down |
| VIT_06s0004g05050.t01 | 1744 | abscisic acid 8'-hydroxylase 2-like | 64 | 2.4780949 | 125 | 6.5161925 | -1.394798 | 3.29E-10 | 7.97E-11 | down |
| VIT_08s0007g03300.t01 | 1896 | iron-sulfur cluster biosynthesis family protein | 247 | 8.7971716 | 482 | 23.112082 | -1.393536 | 3.22E-36 | 2.84E-37 | down |
| VIT_18s0089g00340.t01 | 2243 | Sad1 / UNC-like C-terminal domain containing protein, putative, expressed | 82 | 2.468704 | 160 | 6.4851658 | -1.39339 | 9.84E-13 | 1.99E-13 | down |
| VIT_13s0064g01750.t01 | 1620 | vicianin hydrolase-like | 61 | 2.5427242 | 119 | 6.6782446 | -1.393094 | 9.30E-10 | 2.34E-10 | down |
| VIT_11s0016g03440.t01 | 1648 | probable lactoylglutathione lyase, chloroplast-like | 833 | 34.132825 | 1625 | 89.645095 | -1.393065 | 9.73E-120 | 2.74E-121 | down |
| VIT_02s0025g01740.t01 | 2720 | auxin response factor 9 | 345 | 8.5651433 | 673 | 22.49451 | -1.393024 | 3.88E-50 | 2.53E-51 | down |
| VIT_08s0007g00300.t01 | 408 | protein notum homolog-like | 19 | 3.1446903 | 37 | 8.2446445 | -1.390539 | 0.00088326 | 0.00041748 | down |
| VIT_17s0000g07470.t01 | 2636 | microtubule-associated protein TORTIFOLIA1-like | 166 | 4.2525274 | 323 | 11.140059 | -1.389364 | 1.67E-24 | 2.02E-25 | down |
| VIT_06s0004g07710.t01 | 1557 | putative tRNA pseudouridine synthase-like | 73 | 3.1660567 | 142 | 8.2914427 | -1.388936 | 2.22E-11 | 4.95E-12 | down |
| VIT_07s0191g00040.t01 | 2684 | tRNA (cytosine(34)-C(5))-methyltransferase-like | 91 | 2.2895141 | 177 | 5.9954409 | -1.388824 | 6.58E-14 | 1.25E-14 | down |
| VIT_15s0046g02560.t01 | 2608 | myc anthocyanin regulatory protein (MYCA1) | 127 | 3.2883692 | 247 | 8.6103289 | -1.388696 | 5.61E-19 | 8.37E-20 | down |
| VIT_12s0035g01880.t01 | 1315 | ZF-HD homeobox protein | 140 | 7.189302 | 272 | 18.805008 | -1.387193 | 9.32E-21 | 1.29E-21 | down |
| VIT_14s0108g00150.t01 | 2235 | protein IQ-DOMAIN 31-like | 531 | 16.043586 | 1031 | 41.938366 | -1.386274 | 9.82E-76 | 4.29E-77 | down |
| VIT_12s0035g01780.t01 | 1121 | 3-oxoacyl-[acyl-carrier-protein] reductase FabG-like | 135 | 8.1322852 | 262 | 21.248391 | -1.385621 | 5.29E-20 | 7.60E-21 | down |
| VIT_17s0000g05140.t01 | 1452 | rhomboid protein 1 | 50 | 2.3253473 | 97 | 6.0734504 | -1.38507 | 4.29E-08 | 1.22E-08 | down |
| VIT_03s0063g01880.t01 | 2207 | long chain acyl-CoA synthetase 2-like | 330 | 10.097086 | 640 | 26.3638 | -1.384619 | 2.66E-47 | 1.84E-48 | down |
| VIT_00s0524g00010.t01 | 472 | spotted leaf 11, putative, expressed | 66 | 9.4424867 | 128 | 24.654622 | -1.384619 | 2.53E-10 | 6.06E-11 | down |
| VIT_12s0035g01100.t01 | 1242 | KH domain-containing protein | 179 | 9.7323088 | 347 | 25.400265 | -1.38399 | 4.25E-26 | 4.88E-27 | down |
| VIT_13s0106g00500.t01 | 1608 | Putative uncharacterized protein | 177 | 7.4331289 | 343 | 19.392708 | -1.383473 | 8.54E-26 | 9.88E-27 | down |
| VIT_01s0113g00420.t01 | 2567 | probable S-acyltransferase | 256 | 6.7343943 | 496 | 17.566538 | -1.38321 | 7.82E-37 | 6.77E-38 | down |
| VIT_05s0020g03380.t01 | 2576 | serine/threonine-protein kinase WNK1-like | 111 | 2.9097894 | 215 | 7.587924 | -1.38279 | 1.47E-16 | 2.45E-17 | down |
| VIT_13s0019g01960.t01 | 985 | E3 ubiquitin-protein ligase | 126 | 8.6381106 | 244 | 22.520808 | -1.382471 | 1.22E-18 | 1.85E-19 | down |
| VIT_03s0063g00300.t01 | 1797 | DJ-1 family protein, putative, expressed | 155 | 5.8246263 | 300 | 15.177616 | -1.381708 | 1.18E-22 | 1.53E-23 | down |
| VIT_05s0051g00860.t01 | 1335 | PTAC5, putative, expressed | 278 | 14.062028 | 538 | 36.637968 | -1.381535 | 8.52E-40 | 6.93E-41 | down |
| VIT_07s0031g00210.t01 | 2238 | polyadenylate-binding protein 2-like | 1052 | 31.742425 | 2035 | 82.667481 | -1.380908 | 9.19E-148 | 2.06E-149 | down |
| VIT_16s0050g01440.t01 | 1722 | Putative uncharacterized protein | 75 | 2.9411187 | 145 | 7.6553531 | -1.380104 | 1.70E-11 | 3.76E-12 | down |
| VIT_02s0087g00470.t01 | 1116 | benzyl alcohol O-benzoyltransferase-like | 165 | 9.9839913 | 319 | 25.987043 | -1.380104 | 5.59E-24 | 6.88E-25 | down |
| VIT_18s0001g12510.t01 | 2557 | cytosolic purine 5'-nucleotidase-like | 253 | 6.6815041 | 489 | 17.386353 | -1.379711 | 3.42E-36 | 3.01E-37 | down |
| VIT_03s0088g00950.t01 | 1249 | powdery mildew resistant protein 5, putative, expressed | 59 | 3.1898776 | 114 | 8.2979877 | -1.37926 | 2.87E-09 | 7.47E-10 | down |
| VIT_14s0171g00230.t01 | 1789 | mTERF domain containing protein, expressed | 162 | 6.1148966 | 313 | 15.906124 | -1.379182 | 1.59E-23 | 1.99E-24 | down |
| VIT_06s0061g00080.t01 | 1651 | astaxanthin synthase KC28, putative, expressed | 86 | 3.5175139 | 166 | 9.1409511 | -1.377788 | 5.79E-13 | 1.15E-13 | down |
| VIT_06s0004g04050.t01 | 1784 | laccase 17 | 86 | 3.2552777 | 166 | 8.4594789 | -1.377788 | 5.79E-13 | 1.15E-13 | down |
| VIT_14s0068g00260.t01 | 1687 | serine carboxypeptidase-like 25 | 172 | 6.8849027 | 332 | 17.891773 | -1.377788 | 7.48E-25 | 8.94E-26 | down |
| VIT_17s0000g01110.t01 | 1316 | neurofilament medium polypeptide | 515 | 26.426265 | 994 | 68.669023 | -1.377687 | 2.23E-72 | 1.03E-73 | down |
| VIT_04s0008g01320.t01 | 986 | Putative uncharacterized protein | 100 | 6.8486903 | 193 | 17.795523 | -1.377614 | 6.71E-15 | 1.22E-15 | down |
| VIT_03s0063g02510.t01 | 1689 | probable receptor-like serine/threonine-protein kinase | 57 | 2.278923 | 110 | 5.9209775 | -1.377483 | 5.74E-09 | 1.53E-09 | down |
| VIT_06s0004g07170.t01 | 1377 | protein FLC EXPRESSOR | 57 | 2.7952803 | 110 | 7.2625497 | -1.377483 | 5.74E-09 | 1.53E-09 | down |
| VIT_13s0047g01050.t01 | 3388 | valyl-tRNA synthetase-like | 326 | 6.4976848 | 629 | 16.878646 | -1.377202 | 3.88E-46 | 2.75E-47 | down |
| VIT_17s0000g09740.t01 | 2253 | abhydrolase domain-containing protein FAM108C1-like | 141 | 4.2261253 | 272 | 10.975848 | -1.376925 | 1.55E-20 | 2.17E-21 | down |
| VIT_01s0011g03940.t01 | 239 | chloroplast | 182 | 51.423062 | 351 | 133.51793 | -1.376546 | 3.51E-26 | 4.03E-27 | down |
| VIT_14s0060g01580.t01 | 1849 | probable protein phosphatase 2C 5-like | 516 | 18.845047 | 995 | 48.92339 | -1.376339 | 2.41E-72 | 1.12E-73 | down |
| VIT_08s0040g02630.t01 | 525 | 60S ribosomal protein L23a-like | 94 | 12.090743 | 181 | 31.343655 | -1.374271 | 5.45E-14 | 1.03E-14 | down |
| VIT_00s0396g00030.t01 | 747 | mitochondrial genome | 65 | 5.8759379 | 125 | 15.213172 | -1.37243 | 5.47E-10 | 1.35E-10 | down |
| VIT_13s0047g01060.t01 | 333 | valine--tRNA ligase | 26 | 5.2724632 | 50 | 13.650738 | -1.37243 | 0.00011808 | 4.88E-05 | down |
| VIT_11s0206g00110.t01 | 1802 | lysosomal Pro-X carboxypeptidase-like | 454 | 17.01318 | 873 | 44.044312 | -1.372303 | 2.64E-63 | 1.37E-64 | down |
| VIT_01s0011g00780.t01 | 2655 | probable methyltransferase PMT5-like | 269 | 6.8418287 | 517 | 17.703388 | -1.371572 | 7.17E-38 | 6.04E-39 | down |
| VIT_00s1764g00020.t01 | 1548 | probable LRR receptor-like serine/threonine-protein kinase | 140 | 6.1071913 | 269 | 15.798349 | -1.371193 | 3.35E-20 | 4.78E-21 | down |
| VIT_15s0046g02030.t01 | 1727 | probable serine/threonine-protein kinase | 165 | 6.451728 | 317 | 16.687731 | -1.37103 | 1.31E-23 | 1.64E-24 | down |
| VIT_11s0016g02030.t01 | 366 | petA; apocytochrome f | 76 | 14.022226 | 146 | 36.266208 | -1.370911 | 1.84E-11 | 4.08E-12 | down |
| VIT_05s0020g00640.t01 | 856 | S-adenosyl-L-methionine-dependent methyltransferase-like | 163 | 12.858736 | 313 | 33.243056 | -1.370304 | 2.64E-23 | 3.32E-24 | down |
| VIT_02s0012g02030.t01 | 2950 | homeobox-leucine zipper protein ROC3-like | 163 | 3.7312129 | 313 | 9.6461207 | -1.370304 | 2.64E-23 | 3.32E-24 | down |
| VIT_17s0000g09910.t01 | 1201 | protein CbbY-like | 1784 | 100.30817 | 3424 | 259.19172 | -1.369581 | 5.03E-245 | 6.57E-247 | down |
| VIT_08s0058g00370.t01 | 1792 | Putative uncharacterized protein | 543 | 20.461915 | 1042 | 52.864008 | -1.369345 | 3.94E-75 | 1.73E-76 | down |
| VIT_18s0041g00720.t01 | 129 | eukaryotic peptide chain release factor subunit 1-3 | 37 | 19.368521 | 71 | 50.037893 | -1.369307 | 3.91E-06 | 1.36E-06 | down |
| VIT_16s0050g00880.t01 | 1230 | rRNA 2'-O-methyltransferase fibrillarin 2-like | 879 | 48.257876 | 1686 | 124.61859 | -1.368683 | 6.34E-121 | 1.74E-122 | down |
| VIT_01s0011g03380.t01 | 1936 | dihydrolipoyllysine-residue acetyltransferase component of pyruvate dehydrogenase ( | 608 | 21.207168 | 1166 | 54.754973 | -1.368438 | 7.00E-84 | 2.78E-85 | down |
| VIT_02s0025g01470.t01 | 878 | Putative uncharacterized protein | 568 | 43.685596 | 1089 | 112.76225 | -1.368055 | 2.29E-78 | 9.66E-80 | down |
| VIT_06s0061g01230.t01 | 2675 | glucomannan 4-beta-mannosyltransferase 2-like | 876 | 22.113871 | 1679 | 57.063352 | -1.367613 | 2.76E-120 | 7.64E-122 | down |
| VIT_03s0180g00120.t01 | 2090 | Citrate transporter protein, putative, expressed | 288 | 9.3053057 | 552 | 24.011714 | -1.367613 | 3.42E-40 | 2.73E-41 | down |
| VIT_07s0104g01120.t01 | 1697 | rhoGAP domain containing protein, expressed | 775 | 30.839285 | 1485 | 79.556375 | -1.367208 | 1.93E-106 | 6.07E-108 | down |
| VIT_09s0054g00620.t01 | 1523 | kelch repeat-containing protein | 131 | 5.8083909 | 251 | 14.983187 | -1.367134 | 7.71E-19 | 1.16E-19 | down |
| VIT_04s0008g06260.t01 | 1307 | exosome complex component RRP42-like | 83 | 4.2883177 | 159 | 11.059918 | -1.366857 | 2.50E-12 | 5.19E-13 | down |
| VIT_14s0081g00490.t01 | 1802 | FAD dependent oxidoreductase, putative, expressed | 189 | 7.0825795 | 362 | 18.263506 | -1.366617 | 1.10E-26 | 1.25E-27 | down |
| VIT_07s0005g04110.t01 | 3136 | cellulose synthase A catalytic subunit 4 [UDP-forming]-like | 128 | 2.7562484 | 245 | 7.1026498 | -1.365651 | 2.18E-18 | 3.35E-19 | down |

| | | | | | | | | | | |
|---|---|---|---|---|---|---|---|---|---|---|
| VIT_08s0040g02290.t01 | 2050 | importin subunit alpha-2-like | 69 | 2.2728966 | 132 | 5.8539693 | -1.364883 | 2.09E-10 | 4.99E-11 | down |
| VIT_03s0038g04610.t01 | 2778 | adoMet-dependent rRNA methyltransferase spb1-like | 234 | 5.688111 | 447 | 14.628697 | -1.36278 | 1.43E-32 | 1.37E-33 | down |
| VIT_18s0001g07960.t01 | 1751 | serine hydroxymethyltransferase, mitochondrial-like | 167 | 6.4404286 | 319 | 16.562844 | -1.362722 | 1.53E-23 | 1.92E-24 | down |
| VIT_01s0010g03510.t01 | 876 | anthocyanin O-methyltransferase (FAOMT) | 89 | 6.8607302 | 170 | 17.643112 | -1.362671 | 4.77E-13 | 9.46E-14 | down |
| VIT_00s1683g00010.t01 | 1067 | wound responsive protein, putative, expressed | 44 | 2.7846634 | 84 | 7.1572344 | -1.361899 | 5.27E-07 | 1.66E-07 | down |
| VIT_02s0012g00590.t01 | 1624 | Putative uncharacterized protein | 66 | 2.744368 | 126 | 7.053666 | -1.361899 | 5.90E-10 | 1.46E-10 | down |
| VIT_18s0001g07060.t01 | 1121 | phosphoribosylaminoimidazole-succinocarboxamide synthase | 65 | 3.9155447 | 124 | 10.05649 | -1.360842 | 8.34E-10 | 2.09E-10 | down |
| VIT_07s0130g00120.t01 | 3135 | tetratricopeptide repeat protein 27 homolog | 97 | 2.0893858 | 185 | 5.3649361 | -1.360482 | 4.49E-14 | 8.47E-15 | down |
| VIT_13s0019g04730.t01 | 2240 | glycerol-3-phosphate dehydrogenase SDP6 | 85 | 2.5624497 | 162 | 6.5750244 | -1.359473 | 1.91E-12 | 3.93E-13 | down |
| VIT_17s0000g04310.t01 | 1962 | DEAD-box ATP-dependent RNA helicase 51-like | 159 | 5.4724596 | 303 | 14.040223 | -1.359305 | 2.47E-22 | 3.24E-23 | down |
| VIT_05s0077g01060.t01 | 2218 | aldehyde dehydrogenase 22A1-like | 230 | 7.0024616 | 438 | 17.953244 | -1.35831 | 8.83E-32 | 8.63E-33 | down |
| VIT_05s0049g02140.t01 | 1706 | glutamyl-tRNA(Gln) amidotransferase subunit B | 115 | 4.5520105 | 219 | 11.670661 | -1.35831 | 2.01E-16 | 3.37E-17 | down |
| VIT_00s0396g00060.t01 | 906 | mitochondrion | 94 | 7.0062253 | 179 | 17.962021 | -1.35824 | 1.28E-13 | 2.46E-14 | down |
| VIT_02s0025g02090.t01 | 2145 | nephrocystin-3-like | 289 | 9.0981897 | 550 | 23.311261 | -1.357376 | 1.31E-39 | 1.07E-40 | down |
| VIT_16s0039g00970.t01 | 866 | Glutathione S-transferase | 41 | 3.1970572 | 78 | 8.1885515 | -1.356864 | 1.49E-06 | 4.94E-07 | down |
| VIT_14s0066g00200.t01 | 1605 | pentatricopeptide repeat-containing protein | 121 | 5.0909025 | 230 | 13.028163 | -1.35564 | 3.81E-17 | 6.17E-18 | down |
| VIT_13s0047g01120.t01 | 1469 | Putative uncharacterized protein | 50 | 2.2984373 | 95 | 5.8793888 | -1.355013 | 1.01E-07 | 2.96E-08 | down |
| VIT_06s0080g00580.t01 | 1944 | rhoGAP domain containing protein, expressed | 60 | 2.0842002 | 114 | 5.3313717 | -1.355013 | 4.74E-09 | 1.25E-09 | down |
| VIT_03s0091g00460.t01 | 662 | zinc finger SWIM domain-containing protein 7-like | 20 | 2.0401235 | 38 | 5.2186237 | -1.355013 | 0.0009532 | 0.00045438 | down |
| VIT_17s0053g00240.t01 | 466 | lysM domain containing protein, putative, expressed | 30 | 4.3473017 | 57 | 11.120372 | -1.355013 | 4.46E-05 | 1.75E-05 | down |
| VIT_04s0079g00680.t01 | 1794 | phytoene synthase | 414 | 15.583405 | 786 | 39.83185 | -1.353912 | 5.99E-56 | 3.51E-57 | down |
| VIT_17s0000g08850.t01 | 1072 | SEC14 cytosolic factor-like | 137 | 8.6299887 | 260 | 22.050017 | -1.353349 | 3.41E-19 | 5.05E-20 | down |
| VIT_13s0084g00790.t01 | 885 | thioredoxin | 251 | 19.152034 | 476 | 48.898333 | -1.352288 | 3.19E-34 | 2.93E-35 | down |
| VIT_08s0007g06680.t01 | 2562 | protein trichome birefringence | 450 | 11.860905 | 853 | 30.269154 | -1.351634 | 1.76E-60 | 9.55E-62 | down |
| VIT_00s0181g00240.t01 | 816 | ribosomal RNA-processing protein 17 | 85 | 7.0341757 | 161 | 17.937672 | -1.350539 | 2.91E-12 | 6.07E-13 | down |
| VIT_14s0083g00080.t01 | 1169 | Putative uncharacterized protein | 204 | 11.7842 | 386 | 30.01948 | -1.349045 | 7.61E-28 | 8.24E-29 | down |
| VIT_11s0016g04330.t01 | 704 | dihydrofolate reductase | 27 | 2.5898556 | 51 | 6.5860935 | -1.346551 | 0.0001271 | 5.29E-05 | down |
| VIT_18s0122g00620.t01 | 1174 | reductase, putative, expressed | 366 | 21.052197 | 691 | 53.510662 | -1.345856 | 7.17E-49 | 4.81E-50 | down |
| VIT_14s0128g00740.t01 | 2209 | sugar transporter superfamily, putative, expressed | 132 | 4.0351776 | 249 | 10.247879 | -1.344621 | 2.95E-18 | 4.55E-19 | down |
| VIT_08s0040g00010.t01 | 1612 | TKL_IRAK_CrRLK1L-1.5 - The CrRLK1L-1 subfamily has homology to the CrRI | 845 | 35.397787 | 1593 | 89.842352 | -1.343737 | 3.70E-111 | 1.12E-112 | down |
| VIT_14s0030g00630.t01 | 850 | ribosomal L18p/L5e family protein, expressed | 43 | 3.4161267 | 81 | 8.6635616 | -1.342599 | 1.13E-06 | 3.71E-07 | down |
| VIT_00s0838g00020.t01 | 513 | ribosomal protein, putative, expressed | 867 | 114.12642 | 1633 | 289.40044 | -1.342434 | 9.24E-114 | 2.73E-115 | down |
| VIT_00s0332g00080.t01 | 2177 | mitochondrial genome | 77 | 2.3884532 | 145 | 6.0553597 | -1.342136 | 4.62E-11 | 1.05E-11 | down |
| VIT_07s0151g01060.t01 | 1098 | fatty-acid-binding protein 1 | 102 | 6.2731009 | 192 | 15.897516 | -1.341551 | 2.80E-14 | 5.22E-15 | down |
| VIT_00s0813g00010.t01 | 322 | nucleobase-ascorbate transporter, putative, expressed | 51 | 10.695442 | 96 | 27.10477 | -1.341551 | 1.08E-07 | 3.19E-08 | down |
| VIT_00s0220g00070.t01 | 1562 | beta-glucosidase 42-like | 467 | 20.189255 | 878 | 51.102701 | -1.339812 | 2.10E-61 | 1.12E-62 | down |
| VIT_13s0064g00190.t01 | 1028 | RNA polymerase sigma factor rpoD-like | 75 | 4.92666 | 141 | 12.46971 | -1.339746 | 9.24E-11 | 2.15E-11 | down |
| VIT_05s0077g01210.t01 | 2090 | dihydrolipoyl dehydrogenase-like | 408 | 13.182516 | 767 | 33.364103 | -1.339671 | 9.32E-54 | 5.65E-55 | down |
| VIT_03s0063g02680.t01 | 611 | MYB family transcription factor, putative, expressed | 58 | 6.4101948 | 109 | 16.218686 | -1.339217 | 1.45E-08 | 3.97E-09 | down |
| VIT_00s0282g00020.t01 | 1692 | K(+) efflux antiporter 3 | 156 | 6.2259938 | 293 | 15.743368 | -1.338368 | 3.79E-21 | 5.19E-22 | down |
| VIT_07s0141g00290.t01 | 1122 | auxin-responsive protein IAA16-like | 695 | 41.828895 | 1305 | 105.74212 | -1.337978 | 1.25E-90 | 4.64E-92 | down |
| VIT_00s0246g00240.t01 | 1017 | mitochondrial genome | 49 | 3.2535656 | 92 | 8.2242679 | -1.337866 | 2.16E-07 | 6.56E-08 | down |
| VIT_14s0068g01700.t01 | 628 | ATPF0B, atpF; F-type H+-transporting ATPase subunit b | 790 | 84.947752 | 1483 | 214.69003 | -1.337608 | 7.93E-103 | 2.60E-104 | down |
| VIT_18s0001g02810.t01 | 2651 | long chain acyl-CoA synthetase 9 | 357 | 9.0937483 | 669 | 22.942818 | -1.335096 | 8.85E-47 | 6.18E-48 | down |
| VIT_00s0494g00010.t01 | 2354 | growth-regulating factor 1-like | 87 | 2.4957279 | 163 | 6.2952288 | -1.334798 | 3.35E-12 | 7.04E-13 | down |
| VIT_02s0012g01170.t01 | 2154 | pyruvate kinase isozyme G, chloroplastic-like | 125 | 3.9187608 | 234 | 9.8764423 | -1.333594 | 5.09E-17 | 8.31E-18 | down |
| VIT_13s0074g00390.t01 | 1746 | cytochrome P450 77A2-like | 429 | 16.591953 | 803 | 41.812071 | -1.333436 | 7.70E-56 | 4.51E-57 | down |
| VIT_02s0012g01350.t01 | 1570 | glycine-rich cell wall structural protein 1.8-like | 1052 | 45.248119 | 1969 | 114.01879 | -1.333342 | 1.44E-135 | 3.46E-137 | down |
| VIT_16s0098g01540.t01 | 722 | thylakoid lumenal 17.4 kDa protein | 413 | 38.627562 | 773 | 97.335815 | -1.33334 | 8.77E-54 | 5.31E-55 | down |
| VIT_18s0001g03200.t01 | 1082 | transmembrane emp24 domain-containing protein 10-like | 70 | 4.3687302 | 131 | 11.007138 | -1.333153 | 5.21E-10 | 1.28E-10 | down |
| VIT_01s0011g05380.t01 | 1750 | probable pectinesterase/pectinesterase inhibitor 6-like | 108 | 4.1674476 | 202 | 10.494064 | -1.332337 | 8.01E-15 | 1.46E-15 | down |
| VIT_11s0052g01200.t01 | 935 | probable xyloglucan endotransglucosylase/hydrolase protein 23-like | 123 | 8.8833739 | 230 | 22.363851 | -1.331989 | 1.02E-16 | 1.69E-17 | down |
| VIT_10s0003g01550.t01 | 1677 | leucine rich repeat family protein, putative, expressed | 123 | 4.952865 | 230 | 12.468814 | -1.331989 | 1.02E-16 | 1.69E-17 | down |
| VIT_10s0003g03850.t01 | 727 | protease Do-like 5 | 46 | 4.2727538 | 86 | 10.754604 | -1.331716 | 6.10E-07 | 1.94E-07 | down |
| VIT_10s0092g00790.t01 | 379 | RNA recognition motif containing protein, putative, expressed | 107 | 19.064658 | 200 | 47.975682 | -1.331403 | 1.14E-14 | 2.08E-15 | down |
| VIT_03s0017g01330.t01 | 1489 | prenyltransferase, putative, expressed | 191 | 8.6620984 | 357 | 21.79736 | -1.331365 | 2.45E-25 | 2.89E-26 | down |
| VIT_18s0001g13150.t01 | 2473 | homeodomain protein, putative, expressed | 84 | 2.2937158 | 157 | 5.7717287 | -1.331317 | 9.44E-12 | 2.05E-12 | down |
| VIT_10s0003g00080.t01 | 1665 | zinc finger C3HC4 type family protein, putative, expressed | 106 | 4.2990854 | 198 | 10.811385 | -1.33045 | 1.61E-14 | 2.97E-15 | down |
| VIT_01s0011g00240.t01 | 1948 | kinase, pfkB family, putative, expressed | 113 | 3.9171837 | 211 | 9.8474521 | -1.329934 | 2.16E-15 | 3.80E-16 | down |
| VIT_16s0100g00410.t01 | 1274 | TraB family protein, putative, expressed | 128 | 6.7846115 | 239 | 17.05528 | -1.32988 | 2.72E-17 | 4.40E-18 | down |
| VIT_06s0061g01340.t01 | 1764 | phosphoglycerate mutase, putative, expressed | 158 | 6.048434 | 295 | 15.203858 | -1.329804 | 4.37E-21 | 5.99E-22 | down |
| VIT_11s0016g00190.t01 | 2265 | ternary complex factor MIP1, putative, expressed | 75 | 2.2360294 | 140 | 5.6194033 | -1.329478 | 1.40E-10 | 3.30E-11 | down |
| VIT_02s0087g00180.t01 | 1723 | putrescine-binding periplasmic protein-related, putative, expressed | 75 | 2.9394118 | 140 | 7.3870856 | -1.329478 | 1.40E-10 | 3.30E-11 | down |
| VIT_04s0023g00760.t01 | 1279 | biotin carboxyl carrier protein of acetyl-CoA carboxylase 1, chloroplastic-like | 390 | 20.591051 | 728 | 51.747719 | -1.329478 | 1.71E-50 | 1.11E-51 | down |
| VIT_07s0005g00300.t01 | 2531 | zinc finger CCCH domain-containing protein 31-like | 82 | 2.1877926 | 153 | 5.495784 | -1.328849 | 1.88E-11 | 4.17E-12 | down |
| VIT_05s0102g00450.t01 | 1063 | calmodulin-like protein 5 | 290 | 18.422526 | 541 | 46.269454 | -1.328589 | 9.57E-38 | 8.09E-39 | down |
| VIT_02s0025g05170.t01 | 911 | Putative uncharacterized protein | 104 | 7.7090241 | 194 | 19.360373 | -1.328487 | 3.23E-14 | 6.04E-15 | down |
| VIT_01s0011g02870.t01 | 2278 | uncharacterized protein At1g26090, chloroplast precursor, putative, expressed | 96 | 2.8457842 | 179 | 7.1438065 | -1.327867 | 3.42E-13 | 6.75E-14 | down |
| VIT_11s0052g01670.t01 | 384 | OsFtsH1  FtsH protease, homologue of AtFtsH1/5, expressed | 66 | 11.60639 | 123 | 29.120864 | -1.327134 | 2.08E-09 | 5.35E-10 | down |
| VIT_13s0019g02480.t01 | 3192 | cucumisin-like | 283 | 5.9869826 | 527 | 15.009911 | -1.326014 | 1.09E-36 | 9.45E-38 | down |
| VIT_01s0010g02170.t01 | 1254 | CAAX amino terminal protease family protein, putative, expressed | 144 | 7.7544214 | 268 | 19.429769 | -1.325178 | 3.71E-19 | 5.49E-20 | down |
| VIT_11s0052g01320.t01 | 891 | probable xyloglucan endotransglucosylase/hydrolase protein 23-like | 43 | 3.2589312 | 80 | 8.1628658 | -1.324677 | 1.72E-06 | 5.74E-07 | down |
| VIT_14s0068g00910.t01 | 1268 | histidine biosynthesis bifunctional protein hisIE, chloroplastic-like | 215 | 11.449952 | 400 | 28.679469 | -1.324677 | 4.75E-28 | 5.11E-29 | down |
| VIT_14s0006g03070.t01 | 2086 | NADPH-dependent diflavin oxidoreductase 1-like | 120 | 3.8846454 | 223 | 9.7189854 | -1.323023 | 4.38E-16 | 7.46E-17 | down |
| VIT_13s0064g00170.t01 | 1312 | L1P family of ribosomal proteins domain containing protein, expressed | 748 | 38.499244 | 1390 | 96.318861 | -1.322988 | 7.90E-95 | 2.79E-96 | down |
| VIT_10s0003g00890.t01 | 1573 | magnesium-chelatase I subunit | 1572 | 67.485157 | 2921 | 168.82362 | -1.322875 | 2.45E-198 | 4.04E-200 | down |
| VIT_01s0010g01260.t01 | 400 | chloroplast DNA | 192414 | 32483.373 | 357260 | 81199.766 | -1.321774 | 0 | 0 | down |
| VIT_19s0014g02520.t01 | 669 | chloroplast 50S ribosomal protein L14, putative, expressed | 264 | 26.647855 | 490 | 66.588669 | -1.321257 | 5.31E-34 | 4.91E-35 | down |
| VIT_18s0122g00990.t01 | 3093 | glutamate receptor 3.3-like | 125 | 2.7290691 | 232 | 6.8192787 | -1.32121 | 1.17E-16 | 1.94E-17 | down |
| VIT_19s0090g00440.t01 | 1703 | Putative uncharacterized protein | 97 | 3.8462856 | 180 | 9.6092221 | -1.320954 | 3.67E-13 | 7.24E-14 | down |
| VIT_07s0255g00010.t01 | 1256 | nucleoporin p58/p45, putative, expressed | 90 | 4.838796 | 167 | 12.088077 | -1.320865 | 2.73E-12 | 5.69E-13 | down |
| VIT_13s0019g01830.t01 | 2073 | IQ calmodulin-binding motif family protein, putative, expressed | 242 | 7.883163 | 449 | 19.691437 | -1.320722 | 3.19E-31 | 3.16E-32 | down |
| VIT_08s0032g00840.t01 | 1712 | sucrose-phosphatase 1-like | 137 | 5.4038247 | 254 | 13.488397 | -1.319666 | 4.19E-18 | 6.51E-19 | down |
| VIT_06s0004g06570.t01 | 1909 | vacuolar cation/proton exchanger 3-like | 484 | 17.120793 | 897 | 42.718588 | -1.319114 | 2.99E-61 | 1.60E-62 | down |
| VIT_06s0004g00170.t01 | 1197 | antitermination NusB domain-containing protein, putative, expressed | 333 | 18.786009 | 617 | 46.862061 | -1.318762 | 1.99E-42 | 1.51E-43 | down |
| VIT_10s0116g00950.t01 | 1477 | cbbY protein-related, putative, expressed | 88 | 4.0233389 | 163 | 10.033154 | -1.31831 | 5.45E-12 | 1.16E-12 | down |
| VIT_12s0059g00270.t01 | 1564 | protochlorophyllide oxidoreductase | 848 | 36.613694 | 1569 | 91.204563 | -1.316723 | 3.73E-106 | 1.18E-107 | down |
| VIT_18s0001g04790.t01 | 1662 | aspartic proteinase-like protein 1-like | 130 | 5.2819803 | 240 | 13.128364 | -1.313536 | 4.74E-17 | 7.72E-18 | down |
| VIT_01s0011g03910.t01 | 1815 | probable protein phosphatase 2C 15-like | 181 | 6.7342059 | 334 | 16.730164 | -1.312872 | 2.74E-23 | 3.46E-24 | down |
| VIT_17s0000g08170.t01 | 2095 | T-complex protein 1 subunit gamma-like | 373 | 12.0229 | 688 | 29.856217 | -1.312246 | 7.64E-47 | 5.33E-48 | down |
| VIT_05s0077g01710.t01 | 2302 | WD repeat-containing protein 43-like | 109 | 3.1974637 | 201 | 7.9381831 | -1.311881 | 1.97E-14 | 3.66E-15 | down |
| VIT_12s0028g01980.t01 | 1652 | 50S ribosomal protein L1-like | 179 | 7.3169053 | 330 | 18.16077 | -1.31152 | 5.47E-23 | 7.00E-24 | down |
| VIT_01s0010g03660.t01 | 927 | Putative uncharacterized protein | 164 | 11.946716 | 302 | 29.618126 | -1.309866 | 4.34E-21 | 5.95E-22 | down |
| VIT_08s0040g01150.t01 | 709 | 10 kDa chaperonin-like | 56 | 5.3336711 | 103 | 13.207523 | -1.308159 | 6.65E-08 | 1.93E-08 | down |
| VIT_08s0056g01610.t01 | 181 | ripening-related protein-like (GRIP32) | 55 | 20.519584 | 101 | 50.730971 | -1.305865 | 9.39E-08 | 2.76E-08 | down |
| VIT_15s0024g01460.t01 | 2940 | ATP-dependent zinc metalloprotease FtsH-like | 330 | 7.5796832 | 606 | 18.739399 | -1.305865 | 4.47E-41 | 3.50E-42 | down |
| VIT_01s0137g00760.t01 | 2122 | ATP-binding region, ATPase-like domain-containing protein, putative, expressed | 226 | 7.191964 | 415 | 17.780055 | -1.305802 | 1.89E-28 | 2.02E-29 | down |

| | | | | | | | | | | |
|---|---|---|---|---|---|---|---|---|---|---|
| VIT_04s0210g00190.t01 | 1475 | serine acetyltransferase | 79 | 3.6167585 | 145 | 8.9373004 | -1.305142 | 1.21E-10 | 2.84E-11 | down |
| VIT_14s0108g01640.t01 | 554 | NADPH-dependent oxidoreductase, putative, expressed | 521 | 63.505655 | 956 | 156.88394 | -1.304741 | 3.88E-64 | 1.99E-65 | down |
| VIT_18s0041g02170.t01 | 1425 | Putative uncharacterized protein | 72 | 3.4119454 | 132 | 8.4214997 | -1.303483 | 8.99E-10 | 2.26E-10 | down |
| VIT_19s0014g02670.t01 | 2726 | probable serine/threonine-protein kinase | 360 | 8.9178691 | 660 | 22.01144 | -1.303483 | 1.59E-44 | 1.16E-45 | down |
| VIT_09s0054g00010.t01 | 936 | 60S ribosomal protein L7-4-like | 288 | 20.777873 | 528 | 51.284774 | -1.303483 | 7.98E-36 | 7.08E-37 | down |
| VIT_18s0122g01220.t01 | 2861 | bromodomain domain containing protein, expressed | 90 | 2.124267 | 165 | 5.2432004 | -1.303483 | 6.20E-12 | 1.33E-12 | down |
| VIT_09s0002g06970.t01 | 1308 | palmitoyl-monogalactosyldiacylglycerol delta-7 desaturase | 282 | 14.558808 | 517 | 35.93463 | -1.303483 | 4.24E-35 | 3.83E-36 | down |
| VIT_16s0050g00650.t01 | 440 | Putative uncharacterized protein | 24 | 3.6833502 | 44 | 9.0913918 | -1.303483 | 0.0005467 | 0.00025021 | down |
| VIT_12s0028g02450.t01 | 1233 | mitochondrial genome | 1906 | 104.38648 | 3493 | 257.55257 | -1.302932 | 1.50E-231 | 2.06E-233 | down |
| VIT_18s0001g07460.t01 | 1234 | MADS-box protein SVP-like | 178 | 9.7406802 | 326 | 24.017777 | -1.302008 | 1.76E-22 | 2.30E-23 | down |
| VIT_01s0011g02180.t01 | 2498 | nucleolin 2 | 1206 | 32.60163 | 2208 | 80.35946 | -1.301524 | 2.11E-146 | 4.75E-148 | down |
| VIT_03s0091g00700.t01 | 1431 | ATP-dependent Clp protease proteolytic subunit-related protein 1 | 558 | 26.331707 | 1021 | 64.865905 | -1.300659 | 4.13E-68 | 2.01E-69 | down |
| VIT_06s0009g01150.t01 | 3502 | la domain containing protein, putative, expressed | 223 | 4.3000466 | 408 | 10.591913 | -1.300539 | 8.09E-28 | 8.77E-29 | down |
| VIT_18s0001g02090.t01 | 1347 | replication factor C subunit 4-like, transcript variant 1 | 41 | 2.0554206 | 75 | 5.0620221 | -1.30028 | 5.19E-06 | 1.82E-06 | down |
| VIT_19s0014g02810.t01 | 1977 | PAP-associated domain-containing protein 5-like | 93 | 3.1765868 | 170 | 7.8175852 | -1.299246 | 3.32E-12 | 6.97E-13 | down |
| VIT_07s0104g01470.t01 | 1465 | peptide chain release factor 1-like | 151 | 6.9602328 | 276 | 17.12781 | -1.299133 | 3.91E-19 | 5.79E-20 | down |
| VIT_16s0050g01180.t01 | 1545 | transcription factor ASG4-like | 127 | 5.5508524 | 232 | 13.651799 | -1.29831 | 3.06E-16 | 5.15E-17 | down |
| VIT_15s0046g01120.t01 | 1147 | ribosome biogenesis regulatory protein homolog-like | 98 | 5.7696185 | 179 | 14.187961 | -1.298119 | 8.95E-13 | 1.80E-13 | down |
| VIT_19s0085g00020.t01 | 2891 | nucleolar complex protein 3 homolog | 121 | 2.8263225 | 221 | 6.949836 | -1.298053 | 1.61E-15 | 2.82E-16 | down |
| VIT_06s0004g02940.t01 | 2728 | zinc finger CCCH domain-containing protein 24-like | 132 | 3.2674881 | 241 | 8.0316181 | -1.297509 | 8.17E-17 | 1.34E-17 | down |
| VIT_17s0000g02270.t01 | 982 | Down syndrome critical region protein 3 homolog | 40 | 2.7506349 | 73 | 6.7583666 | -1.29691 | 7.33E-06 | 2.62E-06 | down |
| VIT_13s0147g00100.t01 | 747 | RPA3 - Putative single-stranded DNA binding complex subunit 3, expressed | 57 | 5.1527456 | 104 | 12.657359 | -1.296563 | 7.10E-08 | 2.06E-08 | down |
| VIT_09s0018g01610.t01 | 1218 | Putative uncharacterized protein | 564 | 31.269163 | 1029 | 76.806586 | -1.296489 | 2.67E-68 | 1.30E-69 | down |
| VIT_11s0118g00420.t01 | 1867 | heparanase-like protein 3-like (LOC100255842), mRNA | 91 | 3.2914065 | 166 | 8.0834014 | -1.296258 | 6.61E-12 | 1.42E-12 | down |
| VIT_13s0067g02560.t01 | 751 | Putative uncharacterized protein | 85 | 7.6429925 | 155 | 18.763858 | -1.295747 | 3.47E-11 | 7.83E-12 | down |
| VIT_05s0020g02360.t01 | 1563 | probable protein phosphatase 2C 35-like | 51 | 2.2034117 | 93 | 5.4094653 | -1.295747 | 3.73E-07 | 1.16E-07 | down |
| VIT_19s0014g03850.t01 | 965 | petC; cytochrome b6-f complex iron-sulfur subunit | 3846 | 269.13266 | 7010 | 660.42131 | -1.295069 | 0 | 0 | down |
| VIT_06s0004g02910.t01 | 1849 | ruvB-like 1-like | 163 | 5.9529898 | 297 | 14.603263 | -1.294604 | 2.10E-20 | 2.96E-21 | down |
| VIT_07s0141g00390.t01 | 1983 | glutathione reductase | 465 | 15.834877 | 847 | 38.832117 | -1.294145 | 3.11E-56 | 1.81E-57 | down |
| VIT_18s0001g12830.t01 | 1642 | mannan endo-1,4-beta-mannosidase 7-like | 274 | 11.26839 | 499 | 27.628529 | -1.293877 | 1.55E-33 | 1.45E-34 | down |
| VIT_05s0029g01350.t01 | 1611 | TBC1 domain family member 13-like | 89 | 3.7306019 | 162 | 9.1421817 | -1.29313 | 1.31E-11 | 2.88E-12 | down |
| VIT_14s0036g00630.t01 | 1118 | RNA recognition motif containing protein, putative, expressed | 50 | 3.0200396 | 91 | 7.39997 | -1.292952 | 5.27E-07 | 1.66E-07 | down |
| VIT_08s0007g03270.t01 | 1065 | heme-binding-like protein | 50 | 3.1703327 | 91 | 7.7682315 | -1.292952 | 5.27E-07 | 1.66E-07 | down |
| VIT_13s0156g00650.t01 | 1600 | box C/D snoRNA protein 1-like | 50 | 2.1102527 | 91 | 5.1707291 | -1.292952 | 5.27E-07 | 1.66E-07 | down |
| VIT_05s0051g00440.t01 | 1654 | GDT1-like protein 2 | 427 | 17.433188 | 777 | 42.708654 | -1.292692 | 1.39E-51 | 8.86E-53 | down |
| VIT_00s1530g00010.t01 | 784 | probable galactinol--sucrose galactosyltransferase 2-like | 83 | 7.1490194 | 151 | 17.510206 | -1.292379 | 6.92E-11 | 1.59E-11 | down |
| VIT_17s0053g00010.t01 | 405 | Putative uncharacterized protein | 33 | 5.5022885 | 60 | 13.468729 | -1.29151 | 5.50E-05 | 2.18E-05 | down |
| VIT_02s0025g03210.t01 | 1345 | transmembrane protein 120A, putative, expressed | 225 | 11.29652 | 409 | 27.645942 | -1.291189 | 1.38E-27 | 1.51E-28 | down |
| VIT_04s0008g00790.t01 | 602 | 60S ribosomal protein L28-1, putative, expressed | 115 | 12.899884 | 209 | 31.563138 | -1.290883 | 1.28E-14 | 2.35E-15 | down |
| VIT_18s0001g09380.t01 | 1153 | syntaxin 6, N-terminal domain containing protein, expressed | 104 | 6.0909983 | 189 | 14.902628 | -1.290816 | 2.54E-13 | 4.98E-14 | down |
| VIT_18s0001g04340.t01 | 2308 | serine hydroxymethyltransferase 1-like | 376 | 11.001109 | 683 | 26.903902 | -1.290166 | 2.48E-45 | 1.79E-46 | down |
| VIT_06s0061g00900.t01 | 3058 | coiled-coil domain-containing protein 132 | 119 | 2.6278098 | 216 | 6.4216502 | -1.289083 | 4.81E-15 | 8.68E-16 | down |
| VIT_09s0054g01770.t01 | 2109 | Complex I intermediate-associated protein 30 domain containing protein, putative, ex | 297 | 9.5096452 | 539 | 23.234994 | -1.288836 | 6.22E-36 | 5.51E-37 | down |
| VIT_18s0001g00480.t01 | 1998 | T-complex protein 1 subunit beta-like | 523 | 17.676271 | 949 | 43.181836 | -1.288611 | 1.78E-62 | 9.34E-64 | down |
| VIT_14s0030g01270.t01 | 2059 | probable nucleolar protein 5-2-like | 377 | 12.364298 | 684 | 30.201612 | -1.288445 | 2.64E-45 | 1.91E-46 | down |
| VIT_05s0020g04700.t01 | 1189 | Putative uncharacterized protein | 43 | 2.4421427 | 78 | 5.9640753 | -1.288151 | 3.91E-06 | 1.36E-06 | down |
| VIT_14s0081g00700.t01 | 529 | peptidyl-prolyl cis-trans isomerase CYP19-3-like | 129 | 16.467152 | 234 | 40.21523 | -1.288151 | 3.47E-16 | 5.86E-17 | down |
| VIT_00s0505g00040.t01 | 6409 | Putative uncharacterized protein | 1672 | 17.616939 | 3032 | 43.009986 | -1.287708 | 1.80E-197 | 2.98E-199 | down |
| VIT_07s0205g00030.t01 | 2805 | zinc finger family protein, putative, expressed | 91 | 2.1907508 | 165 | 5.3478775 | -1.287541 | 9.91E-12 | 2.15E-12 | down |
| VIT_18s0001g11080.t01 | 1458 | RNA recognition motif containing protein, putative, expressed | 334 | 15.469397 | 605 | 37.724911 | -1.286101 | 4.46E-40 | 3.58E-41 | down |
| VIT_19s0014g03630.t01 | 659 | Putative uncharacterized protein | 37 | 3.79141 | 67 | 9.2431449 | -1.285649 | 2.07E-05 | 7.79E-06 | down |
| VIT_05s0051g00420.t01 | 1521 | xylosyltransferase 1-like | 179 | 7.9470924 | 324 | 19.366278 | -1.285048 | 6.42E-22 | 8.51E-23 | down |
| VIT_14s0171g00340.t01 | 1502 | F-box/kelch-repeat protein | 47 | 2.1130626 | 85 | 5.1449288 | -1.283816 | 1.48E-06 | 4.90E-07 | down |
| VIT_12s0059g01870.t01 | 1790 | indole-3-acetic acid-amido synthetase GH3.5 | 141 | 5.3192515 | 255 | 12.951424 | -1.283816 | 1.86E-17 | 2.97E-18 | down |
| VIT_04s0044g00030.t01 | 2053 | phosphatidylinositide phosphatase SAC1-like | 146 | 4.8022896 | 264 | 11.69083 | -1.283583 | 4.96E-18 | 7.74E-19 | down |
| VIT_15s0046g03700.t01 | 1246 | methionine adenosyltransferase 2 subunit beta-like | 224 | 12.139881 | 405 | 29.550671 | -1.283437 | 4.40E-27 | 4.90E-28 | down |
| VIT_04s0008g07080.t01 | 1299 | aspartic proteinase nepenthesin-2-like | 57 | 2.9631262 | 103 | 7.2087248 | -1.282624 | 1.06E-07 | 3.14E-08 | down |
| VIT_05s0020g02510.t01 | 1218 | guanylate kinase-like | 88 | 4.8788765 | 159 | 11.868073 | -1.282465 | 2.78E-11 | 6.24E-12 | down |
| VIT_16s0039g02110.t01 | 2081 | ATPase family AAA domain-containing protein 3-B-like | 82 | 2.6608857 | 148 | 6.4657664 | -1.280915 | 1.46E-10 | 3.45E-11 | down |
| VIT_12s0035g00690.t01 | 1902 | TCP family transcription factor, putative, expressed | 164 | 5.822611 | 296 | 14.148538 | -1.280915 | 5.04E-20 | 7.22E-21 | down |
| VIT_08s0040g03320.t01 | 1786 | arginine biosynthesis bifunctional protein ArgJ | 87 | 3.2894421 | 157 | 7.9918729 | -1.280691 | 3.93E-11 | 8.89E-12 | down |
| VIT_17s0000g05570.t01 | 1958 | LRR receptor-like serine/threonine-protein kinase FEI 1 | 87 | 3.0004819 | 157 | 7.2898289 | -1.280691 | 3.93E-11 | 8.89E-12 | down |
| VIT_03s0063g00480.t01 | 2086 | protein CYPRO4-like | 1361 | 44.058354 | 2456 | 107.03959 | -1.280657 | 9.49E-159 | 1.99E-160 | down |
| VIT_06s0004g06140.t01 | 1125 | ribosomal protein L4, putative, expressed | 1151 | 69.088736 | 2077 | 167.8473 | -1.280627 | 2.32E-134 | 5.63E-136 | down |
| VIT_09s0002g05150.t01 | 829 | auxin-induced protein 22A-like | 97 | 7.9013563 | 175 | 19.19172 | -1.280312 | 2.84E-12 | 5.92E-13 | down |
| VIT_06s0004g05910.t01 | 1437 | probable sugar phosphate/phosphate translocator | 102 | 4.7932253 | 184 | 11.641031 | -1.28015 | 7.63E-13 | 1.53E-13 | down |
| VIT_07s0005g06490.t01 | 816 | 60S ribosomal protein L13a-4-like | 1034 | 85.568678 | 1865 | 207.78732 | -1.279953 | 1.22E-120 | 3.36E-122 | down |
| VIT_06s0004g02050.t01 | 2986 | origin recognition complex subunit 1-like | 142 | 3.2113156 | 256 | 7.7943613 | -1.279266 | 1.98E-17 | 3.16E-18 | down |
| VIT_00s0211g00070.t01 | 1802 | serine hydroxymethyltransferase | 86 | 3.2227611 | 155 | 7.8200096 | -1.278873 | 5.54E-11 | 1.27E-11 | down |
| VIT_06s0009g00890.t01 | 3063 | ubiquitin carboxyl-terminal hydrolase 8-like | 101 | 2.2266852 | 182 | 5.4020023 | -1.278597 | 1.07E-12 | 2.18E-13 | down |
| VIT_14s0068g00410.t01 | 1592 | ribosome biogenesis protein WDR12 homolog-like | 90 | 3.8175426 | 162 | 9.2512906 | -1.27701 | 2.09E-11 | 4.65E-12 | down |
| VIT_03s0017g01020.t01 | 1773 | Putative uncharacterized protein | 75 | 2.856518 | 135 | 6.9223795 | -1.27701 | 1.08E-09 | 2.73E-10 | down |
| VIT_11s0118g00410.t01 | 2075 | probable anion transporter 6 | 160 | 5.206985 | 288 | 12.618414 | -1.27701 | 2.01E-19 | 2.94E-20 | down |
| VIT_12s0057g01240.t01 | 1570 | indole-3-glycerol phosphate synthase | 320 | 13.763686 | 576 | 33.354405 | -1.27701 | 8.52E-38 | 7.19E-39 | down |
| VIT_09s0002g03240.t01 | 790 | phosphopantothenoylcysteine decarboxylase subunit SIS2 | 30 | 2.5643577 | 54 | 6.2143691 | -1.27701 | 0.00015483 | 6.53E-05 | down |
| VIT_12s0028g00350.t01 | 1902 | T-complex protein 1 subunit epsilon-like | 466 | 16.544736 | 838 | 40.055659 | -1.275634 | 1.96E-54 | 1.17E-55 | down |
| VIT_05s0094g00880.t01 | 587 | Putative uncharacterized protein | 232 | 26.689124 | 417 | 64.584504 | -1.274936 | 1.41E-27 | 1.54E-28 | down |
| VIT_01s0011g02740.t01 | 3319 | phosphoenolpyruvate carboxylase, putative, expressed | 227 | 4.6185223 | 408 | 11.17592 | -1.27489 | 5.27E-27 | 5.88E-28 | down |
| VIT_06s0004g08380.t01 | 971 | Putative uncharacterized protein | 1236 | 85.957482 | 2221 | 207.95037 | -1.274544 | 1.43E-142 | 3.31E-144 | down |
| VIT_02s0025g03970.t01 | 2714 | S1 RNA binding domain containing protein, expressed | 241 | 5.9964145 | 433 | 14.504689 | -1.274347 | 1.42E-28 | 1.50E-29 | down |
| VIT_02s0241g00090.t01 | 1185 | immature colon carcinoma transcript 1 protein precursor, putative, expressed | 118 | 6.7243158 | 212 | 16.264768 | -1.274291 | 1.53E-14 | 2.83E-15 | down |
| VIT_16s0039g01520.t01 | 893 | chaperone DnaJ-domain containing-like | 54 | 4.0834453 | 97 | 9.875308 | -1.274039 | 2.99E-07 | 9.21E-08 | down |
| VIT_05s0062g00960.t01 | 1539 | xyloglucan galactosyltransferase KATAMARI1, putative, expressed | 54 | 2.3694065 | 97 | 5.730117 | -1.274039 | 2.99E-07 | 9.21E-08 | down |
| VIT_02s0025g02890.t01 | 909 | L11 domain containing ribosomal protein, putative, expressed | 864 | 64.185112 | 1552 | 155.22376 | -1.274039 | 7.93E-100 | 2.68E-101 | down |
| VIT_06s0009g01720.t01 | 1197 | nucleoside-triphosphatase, putative, expressed | 54 | 3.0463798 | 97 | 7.3672932 | -1.274039 | 2.99E-07 | 9.21E-08 | down |
| VIT_13s0084g00310.t01 | 1739 | Putative uncharacterized protein | 93 | 3.6113353 | 167 | 8.7306637 | -1.273559 | 1.12E-11 | 2.44E-12 | down |
| VIT_18s0001g06690.t01 | 1334 | thebaine 6-O-demethylase-like | 176 | 8.9092528 | 316 | 21.535831 | -1.273363 | 4.06E-21 | 5.56E-22 | down |
| VIT_00s0394g00010.t01 | 662 | carboxyl-terminal peptidase, putative, expressed | 44 | 4.4882716 | 79 | 10.849244 | -1.273363 | 4.16E-06 | 1.45E-06 | down |
| VIT_03s0088g00500.t01 | 2204 | aspartyl-tRNA synthetase-like | 122 | 3.7379431 | 219 | 9.0336424 | -1.273063 | 5.77E-15 | 1.04E-15 | down |
| VIT_16s0022g01840.t01 | 1055 | clp protease-related protein | 405 | 25.923104 | 727 | 62.648738 | -1.273047 | 3.25E-47 | 2.25E-48 | down |
| VIT_06s0004g06220.t01 | 1830 | sodium-dependent phosphate transport protein 1 | 502 | 18.524098 | 901 | 44.761442 | -1.272853 | 2.93E-58 | 1.65E-59 | down |
| VIT_13s0067g02420.t01 | 570 | Putative uncharacterized protein | 34 | 4.0279911 | 61 | 9.7293842 | -1.272288 | 5.84E-05 | 2.32E-05 | down |
| VIT_02s0033g01050.t01 | 905 | transferase family protein, putative, expressed | 416 | 31.040535 | 746 | 74.941196 | -1.271606 | 2.44E-48 | 1.65E-49 | down |
| VIT_08s0032g00930.t01 | 1941 | fasciclin domain containing protein, expressed | 1067 | 37.121313 | 1913 | 89.602434 | -1.27129 | 1.97E-122 | 5.37E-124 | down |
| VIT_12s0028g00960.t01 | 1563 | phytoene synthase | 327 | 14.127757 | 586 | 34.085448 | -1.270624 | 3.83E-38 | 3.21E-39 | down |
| VIT_16s0098g00330.t01 | 2181 | probable plastid-lipid-associated protein 14 | 330 | 10.217455 | 591 | 24.635546 | -1.269706 | 2.03E-38 | 1.69E-39 | down |
| VIT_15s0048g02200.t01 | 1300 | caffeoyl-CoA O-methyltransferase | 43 | 2.2336213 | 77 | 5.3849013 | -1.269535 | 5.86E-06 | 2.07E-06 | down |

| | | | | | | | | | | |
|---|---|---|---|---|---|---|---|---|---|---|
| VIT_11s0016g05570.t01 | 969 | thylakoid lumenal 16.5 kDa protein | 552 | 38.468012 | 988 | 92.696543 | -1.268856 | 1.76E-63 | 9.06E-65 | down |
| VIT_06s0080g00210.t01 | 1479 | histone deacetylase, putative, expressed | 138 | 6.3007951 | 247 | 15.183055 | -1.268856 | 1.18E-16 | 1.95E-17 | down |
| VIT_17s0000g02230.t01 | 1562 | protein TIFY 6B-like (JASMONATE-ZIM-DOMAIN PROTEIN 3) | 1891 | 81.751352 | 3384 | 196.96075 | -1.268594 | 2.98E-215 | 4.42E-217 | down |
| VIT_07s0151g00860.t01 | 1209 | haloalkane dehalogenase-like | 95 | 5.3061772 | 170 | 12.783595 | -1.268549 | 8.41E-12 | 1.82E-12 | down |
| VIT_07s0130g00110.t01 | 2270 | Putative uncharacterized protein | 71 | 2.112112 | 127 | 5.0863734 | -1.267951 | 4.30E-09 | 1.13E-09 | down |
| VIT_15s0024g01010.t01 | 2183 | amidase, putative, expressed | 146 | 4.5163081 | 261 | 10.86969 | -1.267095 | 1.67E-17 | 2.66E-18 | down |
| VIT_00s0204g00220.t01 | 962 | ATP-dependent Clp protease proteolytic subunit-related protein 4 | 254 | 17.829661 | 454 | 42.905321 | -1.266877 | 1.20E-29 | 1.23E-30 | down |
| VIT_08s0007g07490.t01 | 1858 | Putative uncharacterized protein | 61 | 2.2170147 | 109 | 5.333486 | -1.26646 | 6.02E-08 | 1.74E-08 | down |
| VIT_12s0055g00090.t01 | 396 | ATP synthase protein 9, mitochondrial, putative, expressed | 258 | 43.995571 | 461 | 105.83666 | -1.266409 | 4.48E-30 | 4.59E-31 | down |
| VIT_00s0407g00030.t01 | 2372 | ubiquitin carboxyl-terminal hydrolase family protein, expressed | 75 | 2.1351629 | 134 | 5.1359464 | -1.266284 | 1.62E-09 | 4.14E-10 | down |
| VIT_18s0001g02060.t01 | 1893 | cyclin-A1-1-like | 89 | 3.1748546 | 159 | 7.6361928 | -1.266163 | 4.42E-11 | 1.00E-11 | down |
| VIT_11s0016g02210.t01 | 1350 | GATA zinc finger domain containing protein, expressed | 337 | 16.857011 | 602 | 40.540873 | -1.266028 | 6.08E-39 | 5.01E-40 | down |
| VIT_16s0050g00800.t01 | 813 | mttA/Hcf106 family protein, putative, expressed | 280 | 23.256906 | 500 | 55.912618 | -1.265515 | 1.69E-32 | 1.63E-33 | down |
| VIT_14s0083g01180.t01 | 891 | AGAP003732-PA, putative, expressed | 56 | 4.2441895 | 100 | 10.203582 | -1.265515 | 2.24E-07 | 6.84E-08 | down |
| VIT_02s0012g00750.t01 | 1095 | haloacid dehalogenase-like hydrolase family protein, putative, expressed | 98 | 6.0436096 | 175 | 14.529622 | -1.265515 | 4.50E-12 | 9.51E-13 | down |
| VIT_14s0060g02410.t01 | 2322 | acyl-CoA-binding domain-containing protein 4-like | 331 | 9.6260967 | 591 | 23.139589 | -1.26534 | 3.22E-38 | 2.70E-39 | down |
| VIT_19s0014g01740.t01 | 1471 | sodium/calcium exchanger protein, putative, expressed | 177 | 8.1254054 | 316 | 19.530114 | -1.265189 | 6.45E-21 | 8.89E-22 | down |
| VIT_06s0009g00810.t01 | 1910 | lysosomal beta glucosidase-like | 647 | 22.874697 | 1155 | 54.976741 | -1.265069 | 1.02E-73 | 4.62E-75 | down |
| VIT_00s0194g00030.t01 | 2147 | heat shock cognate protein 80 | 1967 | 61.866673 | 3511 | 148.67199 | -1.264899 | 2.72E-222 | 3.88E-224 | down |
| VIT_19s0015g00490.t01 | 974 | Putative uncharacterized protein | 836 | 57.960452 | 1492 | 139.26444 | -1.264686 | 6.53E-95 | 2.30E-96 | down |
| VIT_06s0004g03070.t01 | 2140 | zinc finger protein JACKDAW-like | 334 | 10.53943 | 596 | 25.319951 | -1.264478 | 1.71E-38 | 1.42E-39 | down |
| VIT_01s0026g02340.t01 | 2121 | probable methyltransferase PMT2-like | 403 | 12.830655 | 719 | 30.819004 | -1.264225 | 3.25E-46 | 2.30E-47 | down |
| VIT_07s0104g00690.t01 | 2158 | GPI transamidase component PIG-T-like | 88 | 2.753694 | 157 | 6.6142192 | -1.264203 | 6.23E-11 | 1.43E-11 | down |
| VIT_14s0083g01100.t01 | 1352 | alpha-1,4-glucan-protein synthase [UDP-forming] 2-like | 161 | 8.0414363 | 287 | 19.299034 | -1.263004 | 4.77E-19 | 7.10E-20 | down |
| VIT_07s0005g03680.t01 | 971 | ABC transporter I family member 19-like | 46 | 3.1990649 | 82 | 7.6775914 | -1.263004 | 3.13E-06 | 1.07E-06 | down |
| VIT_05s0020g00920.t01 | 2198 | chloroplast unusual positioning protein, putative, expressed | 146 | 4.4854871 | 260 | 10.754149 | -1.261557 | 2.49E-17 | 4.01E-18 | down |
| VIT_05s0094g01340.t01 | 1116 | probable thiol methyltransferase 2-like | 82 | 4.9617411 | 146 | 11.893756 | -1.261286 | 3.27E-10 | 7.92E-11 | down |
| VIT_05s0051g00350.t01 | 1050 | PHD-finger domain containing protein, putative, expressed | 41 | 2.636811 | 73 | 6.3206819 | -1.261286 | 1.17E-05 | 4.27E-06 | down |
| VIT_12s0057g00020.t01 | 1236 | thylakoid formation 1 | 956 | 52.230462 | 1700 | 125.04341 | -1.259466 | 2.50E-107 | 7.74E-109 | down |
| VIT_00s0199g00110.t01 | 877 | protein CURVATURE THYLAKOID 1D | 338 | 26.025648 | 601 | 62.302468 | -1.259355 | 1.44E-38 | 1.20E-39 | down |
| VIT_16s0098g01520.t01 | 2424 | WD repeat-containing protein YMR102C | 81 | 2.2565078 | 144 | 5.4008268 | -1.259088 | 4.62E-10 | 1.13E-10 | down |
| VIT_08s0007g03890.t01 | 1245 | ribosome production factor 2 homolog-like | 108 | 5.8578581 | 192 | 14.02046 | -1.259088 | 4.84E-13 | 9.60E-14 | down |
| VIT_08s0007g01730.t01 | 1131 | ataxin-3 homolog-like | 36 | 2.1494351 | 64 | 5.1445541 | -1.259088 | 4.37E-05 | 1.71E-05 | down |
| VIT_06s0004g00830.t01 | 1384 | serine/threonine-protein phosphatase PP2A catalytic subunit-like | 810 | 39.521496 | 1440 | 94.592515 | -1.259088 | 5.19E-91 | 1.92E-92 | down |
| VIT_01s0011g04250.t01 | 650 | zinc finger protein CONSTANS-LIKE 12-like | 45 | 4.6750214 | 80 | 11.189405 | -1.259088 | 4.40E-06 | 1.54E-06 | down |
| VIT_06s0004g05920.t01 | 999 | proliferating cell nuclear antigen-like | 211 | 14.262689 | 375 | 34.126846 | -1.258661 | 1.97E-24 | 2.38E-25 | down |
| VIT_11s0016g03280.t01 | 1576 | protein phosphatase 1 regulatory subunit pprA-like | 67 | 2.8708006 | 119 | 6.864693 | -1.257742 | 1.71E-08 | 4.70E-09 | down |
| VIT_04s0043g00380.t01 | 1594 | Putative uncharacterized protein | 326 | 13.810638 | 579 | 33.023311 | -1.257705 | 4.04E-37 | 3.47E-38 | down |
| VIT_18s0001g07140.t01 | 1102 | ZF-HD homeobox protein, putative, expressed | 223 | 13.664939 | 396 | 32.669611 | -1.25747 | 1.05E-25 | 1.22E-26 | down |
| VIT_04s0008g01410.t01 | 990 | ran-binding protein 1 homolog c-like | 352 | 24.009986 | 625 | 57.39515 | -1.257294 | 5.71E-40 | 4.62E-41 | down |
| VIT_03s0167g00070.t01 | 928 | MADS-box protein SVP-like | 80 | 5.8213868 | 142 | 13.911397 | -1.256833 | 6.48E-10 | 1.61E-10 | down |
| VIT_18s0076g00210.t01 | 1986 | embryogenesis-associated protein EMB8-like | 71 | 2.4141461 | 126 | 5.7679525 | -1.256546 | 6.41E-09 | 1.72E-09 | down |
| VIT_13s0019g01070.t01 | 1723 | ribosomal RNA large subunit methyltransferase N-like | 439 | 17.205357 | 779 | 41.103855 | -1.256416 | 1.63E-49 | 1.08E-50 | down |
| VIT_03s0038g02430.t01 | 1360 | WD-40 repeat-containing protein MSI2-like | 177 | 8.7885818 | 314 | 20.990419 | -1.256029 | 1.44E-20 | 2.01E-21 | down |
| VIT_15s0048g02350.t01 | 1557 | prohibitin-2, putative, expressed | 296 | 12.837709 | 525 | 30.654982 | -1.255734 | 1.13E-33 | 1.06E-34 | down |
| VIT_07s0005g02590.t01 | 2370 | polygalacturonase, putative, expressed | 97 | 2.7638078 | 172 | 6.5979721 | -1.255365 | 9.42E-12 | 2.04E-12 | down |
| VIT_00s0125g00030.t01 | 27351 | Putative uncharacterized protein | 3253 | 8.0314747 | 5767 | 19.169338 | -1.255064 | 0 | 0 | down |
| VIT_00s0332g00060.t01 | 1670 | ATP synthase subunit beta, putative, expressed | 1185 | 47.916636 | 2100 | 114.32289 | -1.254516 | 1.42E-131 | 3.57E-133 | down |
| VIT_08s0007g08450.t01 | 3162 | leucine-rich repeat receptor-like tyrosine-protein kinase | 136 | 2.9044338 | 241 | 6.9292391 | -1.25444 | 5.21E-16 | 8.91E-17 | down |
| VIT_17s0000g08990.t01 | 1840 | alliin lyase precursor, putative, expressed | 61 | 2.2387029 | 108 | 5.3362517 | -1.253164 | 8.99E-08 | 2.63E-08 | down |
| VIT_01s0127g00470.t01 | 1366 | glycosyl transferase 8 domain containing protein, putative, expressed | 596 | 29.463206 | 1055 | 70.215361 | -1.252872 | 2.04E-66 | 1.02E-67 | down |
| VIT_04s0008g01200.t01 | 1006 | translation initiation factor SUI1, putative, expressed | 100 | 6.7125334 | 177 | 15.995789 | -1.252763 | 5.04E-12 | 1.07E-12 | down |
| VIT_11s0016g02170.t01 | 1706 | WD-40 repeat-containing protein MSI4-like | 582 | 23.037131 | 1030 | 54.889411 | -1.252567 | 7.71E-65 | 3.92E-66 | down |
| VIT_00s0125g00070.t01 | 393 | mitochondrial genome | 8697 | 1494.3811 | 15391 | 3560.4481 | -1.252511 | 0 | 0 | down |
| VIT_17s0000g04620.t01 | 627 | mediator of RNA polymerase II transcription subunit 11 | 39 | 4.2003116 | 69 | 10.004881 | -1.252136 | 2.32E-05 | 8.81E-06 | down |
| VIT_08s0032g00730.t01 | 1751 | F-box protein | 78 | 3.0081044 | 138 | 7.1651174 | -1.252136 | 1.29E-09 | 3.27E-10 | down |
| VIT_05s0051g00340.t01 | 2136 | chaperonin CPN60 | 549 | 17.356236 | 971 | 41.328377 | -1.251679 | 4.11E-61 | 2.21E-62 | down |
| VIT_06s0080g00760.t01 | 874 | Pollen Ole e I allergen and extensin family protein precursor, putative, expressed | 642 | 49.603011 | 1135 | 118.06327 | -1.251061 | 3.16E-71 | 1.49E-72 | down |
| VIT_00s0220g00170.t01 | 1098 | endonuclease/exonuclease/phosphatase family domain containing protein, expressed | 43 | 2.6445425 | 76 | 6.2927666 | -1.250676 | 8.76E-06 | 3.16E-06 | down |
| VIT_08s0040g02700.t01 | 1159 | heavy metal-associated domain containing protein, expressed | 73 | 4.253279 | 129 | 10.118978 | -1.250416 | 4.81E-09 | 1.27E-09 | down |
| VIT_04s0023g00170.t01 | 1521 | R3H domain-containing protein 2-like | 60 | 2.6638298 | 106 | 6.3358812 | -1.250043 | 1.27E-07 | 3.76E-08 | down |
| VIT_03s0063g02360.t01 | 710 | organ-specific protein S2-like | 278 | 26.440575 | 491 | 62.871456 | -1.249652 | 2.51E-31 | 2.48E-32 | down |
| VIT_10s0116g01910.t01 | 426 | sufB/sufD domain containing protein, putative, expressed | 141 | 22.350846 | 249 | 53.139825 | -1.249464 | 2.08E-16 | 3.48E-17 | down |
| VIT_00s0332g00050.t01 | 530 | Putative uncharacterized protein | 932 | 118.7475 | 1645 | 282.17622 | -1.248699 | 1.62E-102 | 5.35E-104 | down |
| VIT_03s0038g04420.t01 | 1301 | chaperone protein dnaJ 10 | 140 | 7.2666657 | 247 | 17.260367 | -1.248098 | 2.94E-16 | 4.94E-17 | down |
| VIT_18s0089g01390.t01 | 1234 | Putative uncharacterized protein | 406 | 22.217507 | 716 | 52.750701 | -1.247493 | 4.25E-45 | 3.08E-46 | down |
| VIT_19s0015g00140.t01 | 888 | S10/S20 domain containing ribosomal protein, putative, expressed | 1364 | 103.72557 | 2405 | 246.22519 | -1.247207 | 3.35E-149 | 7.40E-151 | down |
| VIT_08s0007g04870.t01 | 942 | RNA recognition motif containing protein, expressed | 194 | 13.907058 | 342 | 33.006964 | -1.246953 | 4.55E-22 | 6.02E-23 | down |
| VIT_15s0021g00500.t01 | 2103 | NAD-dependent malic enzyme, mitochondrial precursor, putative, expressed | 564 | 18.110243 | 994 | 42.9712 | -1.246564 | 3.83E-62 | 2.02E-63 | down |
| VIT_07s0104g01160.t01 | 1296 | mitochondrial carrier protein, putative, expressed | 46 | 2.3968302 | 81 | 5.6821199 | -1.245302 | 4.66E-06 | 1.63E-06 | down |
| VIT_18s0001g06010.t01 | 1828 | mitochondrion protein, putative, expressed | 326 | 12.042755 | 574 | 28.547368 | -1.245192 | 2.98E-36 | 2.61E-37 | down |
| VIT_05s0049g01180.t01 | 2360 | methyltransferase, putative, expressed | 975 | 27.898256 | 1716 | 66.105205 | -1.244589 | 2.36E-106 | 7.45E-108 | down |
| VIT_16s0050g01460.t01 | 2205 | dnaJ protein ERDJ2A | 308 | 9.4324946 | 542 | 22.347094 | -1.244376 | 2.97E-34 | 2.73E-35 | down |
| VIT_13s0067g01470.t01 | 2861 | low photochemical bleaching 1 protein, putative, expressed | 104 | 2.4547085 | 183 | 5.8151859 | -1.244274 | 2.83E-12 | 5.91E-13 | down |
| VIT_11s0016g00820.t01 | 2594 | protein trichome birefringence-like 14 | 369 | 9.6059614 | 649 | 22.746003 | -1.243611 | 8.95E-41 | 7.05E-42 | down |
| VIT_14s0030g00010.t01 | 771 | tim44-like domain containing protein, expressed | 29 | 2.5399669 | 51 | 6.0137611 | -1.243458 | 0.00032734 | 0.00014448 | down |
| VIT_02s0033g01000.t01 | 1770 | transferase family protein, putative, expressed | 4903 | 187.05661 | 8621 | 442.80728 | -1.243204 | 0 | 0 | down |
| VIT_05s0062g01080.t01 | 1382 | eukaryotic translation initiation factor 3 subunit H, putative, expressed | 248 | 12.11792 | 436 | 28.68196 | -1.243002 | 1.05E-27 | 1.14E-28 | down |
| VIT_10s0003g05060.t01 | 2532 | chloroplast DNA | 260 | 6.9341637 | 457 | 16.409029 | -1.242696 | 5.56E-29 | 5.84E-30 | down |
| VIT_06s0061g00150.t01 | 2588 | RNA-binding zinc finger protein, putative, expressed | 696 | 18.160567 | 1223 | 42.962798 | -1.242279 | 7.79E-76 | 3.39E-77 | down |
| VIT_08s0007g03630.t01 | 1705 | calmodulin-binding protein, putative, expressed | 107 | 4.237833 | 188 | 10.024526 | -1.242135 | 1.51E-12 | 3.09E-13 | down |
| VIT_14s0083g00530.t01 | 2184 | cell division protease ftsH homolog 4, putative, expressed | 218 | 6.7404409 | 383 | 15.943237 | -1.24203 | 1.94E-24 | 2.35E-25 | down |
| VIT_08s0007g05420.t01 | 1148 | tubulin folding cofactor B, putative, expressed | 41 | 2.4117174 | 72 | 5.7019182 | -1.241386 | 1.74E-05 | 6.49E-06 | down |
| VIT_05s0020g02130.t01 | 2482 | 1-deoxy-D-xylulose-5-phosphate synthase | 1375 | 37.409798 | 2414 | 88.423125 | -1.241008 | 1.34E-148 | 2.99E-150 | down |
| VIT_11s0052g01300.t01 | 920 | glycosyl hydrolases family 16, putative, expressed | 339 | 24.882632 | 595 | 58.797588 | -1.240618 | 2.49E-37 | 2.13E-38 | down |
| VIT_03s0017g02330.t01 | 1561 | coproporphyrinogen III oxidase | 1103 | 47.715233 | 1935 | 112.69598 | -1.239914 | 4.06E-119 | 1.15E-120 | down |
| VIT_10s0116g01260.t01 | 942 | prefoldin, putative, expressed | 69 | 4.9463248 | 121 | 11.677902 | -1.239352 | 1.90E-08 | 5.26E-09 | down |
| VIT_05s0136g00140.t01 | 2705 | ELMO/CED-12 family protein, putative, expressed | 433 | 10.809487 | 759 | 25.509672 | -1.238746 | 3.14E-47 | 2.17E-48 | down |
| VIT_13s0156g00010.t01 | 385 | serine/arginine-rich SC35-like splicing factor SCL33 | 97 | 17.01357 | 170 | 40.143808 | -1.238492 | 2.08E-11 | 4.62E-12 | down |
| VIT_04s0008g07400.t01 | 1313 | NAD dependent epimerase/dehydratase family protein, putative, expressed | 165 | 8.4860124 | 289 | 20.010756 | -1.237617 | 1.32E-18 | 2.00E-19 | down |
| VIT_09s0002g06160.t01 | 1131 | protein kinase family protein, putative, expressed | 40 | 2.3882612 | 70 | 5.6268561 | -1.236368 | 2.45E-05 | 9.32E-06 | down |
| VIT_12s0028g01140.t01 | 1804 | pentatricopeptide, putative, expressed | 168 | 6.2886466 | 294 | 14.816348 | -1.236368 | 6.99E-19 | 1.05E-19 | down |
| VIT_12s0035g00320.t01 | 1691 | phosphomethylpyrimidine kinase/thiamin-phosphate pyrophosphorylase, putative, ex | 104 | 4.1531171 | 182 | 9.7849397 | -1.236368 | 4.20E-12 | 8.86E-13 | down |
| VIT_00s2377g00010.t01 | 839 | Putative uncharacterized protein | 84 | 6.7608573 | 147 | 15.928899 | -1.236368 | 5.42E-10 | 1.33E-10 | down |
| VIT_19s0090g01400.t01 | 1095 | wax synthase isoform 1, putative, expressed | 48 | 2.9601353 | 84 | 6.9742183 | -1.236368 | 3.49E-06 | 1.20E-06 | down |
| VIT_05s0094g00450.t01 | 759 | ribosomal protein L51, putative, expressed | 60 | 5.3381887 | 105 | 12.577024 | -1.236368 | 1.88E-07 | 5.68E-08 | down |
| VIT_01s0010g02280.t01 | 948 | Putative uncharacterized protein | 307 | 21.868273 | 537 | 51.498706 | -1.235697 | 1.38E-33 | 1.29E-34 | down |

| | | | | | | | | | | |
|---|---|---|---|---|---|---|---|---|---|---|
| VIT_12s0035g01950.t01 | 1148 | 2-C-methyl-D-erythritol 4-phosphate cytidylyltransferase, putative, expressed | 127 | 7.4704416 | 222 | 17.580914 | -1.234745 | 1.70E-14 | 3.14E-15 | down |
| VIT_00s1286g00020.t01 | 802 | elongation factor, putative, expressed | 361 | 30.396059 | 631 | 71.529529 | -1.234655 | 3.08E-39 | 2.52E-40 | down |
| VIT_06s0004g02080.t01 | 1718 | tRNA methyltransferase, putative, expressed | 95 | 3.7340909 | 166 | 8.7844647 | -1.234197 | 4.12E-11 | 9.34E-12 | down |
| VIT_15s0024g00660.t01 | 1131 | Putative uncharacterized protein | 269 | 16.061057 | 470 | 37.780319 | -1.234068 | 1.83E-29 | 1.90E-30 | down |
| VIT_01s0026g02530.t01 | 1954 | galactosyltransferase family protein, putative, expressed | 75 | 2.5919173 | 131 | 6.0950477 | -1.233618 | 5.35E-09 | 1.42E-09 | down |
| VIT_15s0048g02190.t01 | 1714 | PAP fibrillin family domain containing protein, expressed | 142 | 5.5945089 | 248 | 13.154406 | -1.233463 | 4.84E-16 | 8.25E-17 | down |
| VIT_13s0019g02240.t01 | 651 | 40S ribosomal protein S7, putative, expressed | 71 | 7.3648143 | 124 | 17.316937 | -1.233463 | 1.42E-08 | 3.90E-09 | down |
| VIT_06s0004g06760.t01 | 1704 | NADH-ubiquinone oxidoreductase, mitochondrial precursor, putative, expressed | 426 | 16.882022 | 744 | 39.694809 | -1.233463 | 5.14E-46 | 3.66E-47 | down |
| VIT_01s0127g00340.t01 | 624 | plant-specific domain TIGR01589 family protein, expressed | 327 | 35.387315 | 571 | 83.192062 | -1.233214 | 1.53E-35 | 1.37E-36 | down |
| VIT_16s0039g00660.t01 | 1927 | RNA polymerase sigma factor, putative, expressed | 472 | 16.540351 | 824 | 38.875489 | -1.232871 | 8.14E-51 | 5.26E-52 | down |
| VIT_16s0039g02490.t01 | 1650 | scramblase, putative, expressed | 121 | 4.9520597 | 211 | 11.625962 | -1.231249 | 8.95E-14 | 1.71E-14 | down |
| VIT_11s0037g00850.t01 | 3011 | tetratricopeptide repeat domain containing protein, putative, expressed | 565 | 12.671328 | 985 | 29.741019 | -1.230886 | 2.14E-60 | 1.16E-61 | down |
| VIT_07s0005g03930.t01 | 1039 | DAG protein, chloroplast precursor, putative, expressed | 525 | 34.121507 | 915 | 80.063748 | -1.230468 | 3.82E-56 | 2.23E-57 | down |
| VIT_18s0072g00330.t01 | 1103 | protein of unknown function domain containing protein, expressed | 287 | 17.570771 | 500 | 41.212111 | -1.229891 | 4.03E-31 | 4.02E-32 | down |
| VIT_07s0005g02730.t01 | 879 | protein RADIALIS-like 4 | 31 | 2.3815366 | 54 | 5.5851553 | -1.229705 | 0.00024491 | 0.00010596 | down |
| VIT_08s0056g01340.t01 | 702 | exosome component 10-like | 58 | 5.5792436 | 101 | 13.080208 | -1.229244 | 3.72E-07 | 1.16E-07 | down |
| VIT_16s0050g00910.t01 | 1574 | MATE efflux family protein | 58 | 2.4883285 | 101 | 5.8337393 | -1.229244 | 3.72E-07 | 1.16E-07 | down |
| VIT_19s0015g00770.t01 | 1982 | AGC_PVPK_like_kin82y.14 - ACG kinases include homologs to PKA, PKG and PI | 232 | 7.9043976 | 404 | 18.531394 | -1.229244 | 2.53E-25 | 2.97E-26 | down |
| VIT_13s0047g00080.t01 | 1462 | major facilitator superfamily antiporter, putative, expressed | 85 | 3.9260515 | 148 | 9.2033241 | -1.229076 | 5.71E-10 | 1.41E-10 | down |
| VIT_19s0027g00700.t01 | 1268 | Lojap-related-like | 274 | 14.592031 | 477 | 34.200267 | -1.228827 | 1.07E-29 | 1.11E-30 | down |
| VIT_19s0014g02090.t01 | 1017 | exonuclease, putative, expressed | 50 | 3.3199649 | 87 | 7.7772968 | -1.228101 | 2.61E-06 | 8.86E-07 | down |
| VIT_14s0066g01780.t01 | 784 | Putative uncharacterized protein | 580 | 49.957003 | 1009 | 117.00528 | -1.227815 | 1.32E-61 | 7.05E-63 | down |
| VIT_10s0003g05020.t01 | 1458 | GTP-binding protein, putative, expressed | 506 | 23.435673 | 880 | 54.872598 | -1.22738 | 7.99E-54 | 4.84E-55 | down |
| VIT_19s0014g04970.t01 | 754 | ribosomal protein L24, putative, expressed | 558 | 49.974366 | 969 | 116.83765 | -1.225245 | 5.19E-59 | 2.89E-60 | down |
| VIT_17s0000g02280.t01 | 2269 | Sel1 repeat domain containing protein, putative, expressed | 239 | 7.1129188 | 415 | 16.628152 | -1.225114 | 7.49E-26 | 8.65E-27 | down |
| VIT_17s0119g00090.t01 | 2276 | Putative uncharacterized protein | 140 | 4.1537487 | 243 | 9.7065387 | -1.224543 | 1.42E-15 | 2.48E-16 | down |
| VIT_15s0046g02390.t01 | 2308 | transporter, major facilitator family, putative, expressed | 382 | 11.176659 | 663 | 26.116086 | -1.22445 | 1.19E-40 | 9.36E-42 | down |
| VIT_04s0008g01900.t01 | 882 | Putative uncharacterized protein | 219 | 16.767178 | 380 | 39.169262 | -1.224082 | 9.90E-24 | 1.23E-24 | down |
| VIT_03s0017g00870.t01 | 1284 | transferase family protein, putative, expressed | 283 | 14.883527 | 491 | 34.765369 | -1.223934 | 2.36E-30 | 2.40E-31 | down |
| VIT_05s0020g04770.t01 | 2970 | chloroplastic group IIA intron splicing facilitator CRS1, chloroplast precursor, putat | 139 | 3.1604054 | 241 | 7.3771899 | -1.222962 | 2.00E-15 | 3.52E-16 | down |
| VIT_14s0060g01950.t01 | 2541 | pectinesterase, putative, expressed | 90 | 2.3917858 | 156 | 5.581492 | -1.222563 | 2.27E-10 | 5.43E-11 | down |
| VIT_12s0035g00880.t01 | 827 | glyoxalase family protein, putative, expressed | 30 | 2.4496283 | 52 | 5.7164737 | -1.222563 | 0.00034402 | 0.00015269 | down |
| VIT_18s0001g15370.t01 | 1786 | probable glucuronosyltransferase | 71 | 2.6844872 | 123 | 6.2611489 | -1.221781 | 2.10E-08 | 5.85E-09 | down |
| VIT_05s0020g02870.t01 | 1822 | DEAD-box ATP-dependent RNA helicase 58 | 71 | 2.6314457 | 123 | 6.1374379 | -1.221781 | 2.10E-08 | 5.85E-09 | down |
| VIT_17s0000g08660.t01 | 1544 | anthranilate phosphoribosyltransferase | 422 | 18.456511 | 731 | 43.042794 | -1.221642 | 1.46E-44 | 1.07E-45 | down |
| VIT_08s0007g03390.t01 | 2083 | cell division protein ftsZ homolog 2-1 | 727 | 23.568372 | 1258 | 54.906245 | -1.220118 | 7.92E-76 | 3.45E-77 | down |
| VIT_07s0031g01700.t01 | 1754 | ras-related protein, putative, expressed | 192 | 7.3919 | 332 | 17.208336 | -1.21909 | 9.64E-21 | 1.34E-21 | down |
| VIT_04s0023g02000.t01 | 1813 | transmembrane protein 53-like | 114 | 4.2461124 | 197 | 9.8786772 | -1.218175 | 9.84E-13 | 1.99E-13 | down |
| VIT_10s0003g03810.t01 | 521 | protein TIFY 5A-like (JASMONATE-ZIM-DOMAIN PROTEIN 8) | 687 | 89.043753 | 1187 | 207.13017 | -1.217951 | 2.07E-71 | 9.72E-73 | down |
| VIT_15s0107g00550.t01 | 1051 | tetratricopeptide repeat domain containing protein, expressed | 172 | 11.051219 | 297 | 25.691183 | -1.217068 | 1.27E-18 | 1.92E-19 | down |
| VIT_17s0000g08220.t01 | 2421 | protein EXECUTER 1 | 724 | 20.194273 | 1250 | 46.940271 | -1.21688 | 4.85E-75 | 2.14E-76 | down |
| VIT_19s0090g01210.t01 | 1845 | trigger factor-like | 248 | 9.076946 | 428 | 21.090058 | -1.216284 | 2.44E-26 | 2.78E-27 | down |
| VIT_08s0007g02490.t01 | 1073 | 2-Cys peroxiredoxin | 4547 | 286.16049 | 7842 | 664.44263 | -1.21532 | 0 | 0 | down |
| VIT_14s0083g00640.t01 | 1395 | zinc finger protein CONSTANS-LIKE 2-like | 115 | 5.5668315 | 198 | 12.903911 | -1.21288 | 1.03E-12 | 2.09E-13 | down |
| VIT_08s0007g01610.t01 | 1174 | transcription termination factor nusG family protein, expressed | 93 | 5.3493288 | 160 | 12.390312 | -1.211783 | 1.78E-10 | 4.22E-11 | down |
| VIT_02s0025g02250.t01 | 1539 | dof zinc finger protein DOF4.6-like | 89 | 3.905133 | 153 | 9.0382257 | -1.210668 | 4.71E-10 | 1.15E-10 | down |
| VIT_18s0001g01270.t01 | 2783 | probable methyltransferase PMT26-like | 929 | 22.541715 | 1597 | 52.17015 | -1.210627 | 7.46E-95 | 2.63E-96 | down |
| VIT_02s0025g04130.t01 | 1468 | inactive rhomboid protein 1-like | 1962 | 90.252116 | 3372 | 208.82952 | -1.210293 | 2.82E-199 | 4.62E-201 | down |
| VIT_01s0011g04090.t01 | 1957 | Putative uncharacterized protein | 85 | 2.9330032 | 146 | 6.7825406 | -1.209447 | 1.24E-09 | 3.15E-10 | down |
| VIT_18s0001g09530.t01 | 1668 | Putative uncharacterized protein | 251 | 10.161601 | 431 | 23.491546 | -1.209014 | 2.81E-26 | 3.21E-27 | down |
| VIT_11s0078g00310.t01 | 1828 | isoamylase 1 | 106 | 3.9157424 | 182 | 9.0516045 | -1.208888 | 1.01E-11 | 2.19E-12 | down |
| VIT_09s0002g03880.t01 | 3052 | ATP-dependent zinc metalloprotease FtsH-like | 286 | 6.3279924 | 491 | 14.626059 | -1.208721 | 8.86E-30 | 9.12E-31 | down |
| VIT_12s0028g03370.t01 | 1476 | tryptophanyl-tRNA synthetas | 162 | 7.4116192 | 278 | 17.123353 | -1.208105 | 2.60E-17 | 4.19E-18 | down |
| VIT_14s0083g00610.t01 | 2056 | pentatricopeptide repeat-containing protein | 95 | 3.120218 | 163 | 7.2076695 | -1.207886 | 1.33E-10 | 3.12E-11 | down |
| VIT_05s0094g01110.t01 | 1665 | acetolactate synthase small subunit 2 | 471 | 19.10254 | 808 | 44.119186 | -1.207642 | 2.79E-48 | 1.89E-49 | down |
| VIT_14s0030g01860.t01 | 2129 | trihelix transcription factor GTL2-like | 130 | 4.1233684 | 223 | 9.5226884 | -1.207546 | 4.32E-14 | 8.13E-15 | down |
| VIT_04s0023g01970.t01 | 1164 | putative glucose-6-phosphate 1-epimerase-like | 281 | 16.301883 | 482 | 37.646485 | -1.207477 | 3.32E-29 | 3.47E-30 | down |
| VIT_18s0001g05270.t01 | 1714 | endoplasmic reticulum-Golgi intermediate compartment protein 3-like | 151 | 5.9490905 | 259 | 13.737867 | -1.207417 | 3.46E-16 | 5.85E-17 | down |
| VIT_02s0154g00040.t01 | 1001 | thylakoid lumenal protein | 519 | 35.012065 | 890 | 80.832554 | -1.207084 | 4.89E-53 | 3.00E-54 | down |
| VIT_02s0087g00680.t01 | 382 | protein decapping 5-like | 28 | 4.9497027 | 48 | 11.423738 | -1.206621 | 0.00068272 | 0.0003175 | down |
| VIT_18s0001g00100.t01 | 2197 | Putative uncharacterized protein | 91 | 2.7970213 | 156 | 6.4554261 | -1.206621 | 3.51E-10 | 8.52E-11 | down |
| VIT_09s0002g07210.t01 | 1760 | chaperone protein DnaJ-like | 412 | 15.807711 | 706 | 36.468878 | -1.206037 | 3.17E-42 | 2.42E-43 | down |
| VIT_07s0005g04420.t01 | 2563 | xyloglucan galactosyltransferase KATAMARI1-like | 206 | 5.4275403 | 353 | 12.521503 | -1.206037 | 1.24E-21 | 1.66E-22 | down |
| VIT_08s0056g01520.t01 | 1747 | xylosyltransferase 1-like | 115 | 4.4451803 | 197 | 10.251884 | -1.205575 | 1.52E-12 | 3.11E-13 | down |
| VIT_01s0127g00700.t01 | 1242 | histone-lysine N-methyltransferase SETD1B | 757 | 41.158423 | 1296 | 94.866697 | -1.204714 | 1.47E-76 | 6.38E-78 | down |
| VIT_01s0137g00620.t01 | 2482 | DUF246 domain-containing protein | 281 | 7.6452024 | 481 | 17.618692 | -1.20448 | 4.90E-29 | 5.14E-30 | down |
| VIT_01s0026g01020.t01 | 2446 | microtubule-associated protein SPIRAL2-like | 246 | 6.7914592 | 421 | 15.647898 | -1.204175 | 1.55E-25 | 1.81E-26 | down |
| VIT_08s0007g00770.t01 | 1727 | NDP1, putative, expressed | 97 | 3.792834 | 166 | 8.7386858 | -1.20414 | 9.93E-11 | 2.31E-11 | down |
| VIT_00s0260g00070.t01 | 409 | 60S ribosomal protein L32-1-like | 45 | 7.4297406 | 77 | 17.115823 | -1.203947 | 1.44E-05 | 5.30E-06 | down |
| VIT_07s0104g00590.t01 | 1761 | Putative uncharacterized protein | 1100 | 42.181088 | 1882 | 97.1607 | -1.203777 | 1.23E-110 | 3.72E-112 | down |
| VIT_02s0025g03550.t01 | 2443 | probable serine/threonine-protein kinase | 152 | 4.2015019 | 260 | 9.6756523 | -1.203454 | 3.63E-16 | 6.14E-17 | down |
| VIT_13s0019g02090.t01 | 1141 | thylakoid lumenal 15 kDa protein 1 | 528 | 31.248755 | 903 | 71.950278 | -1.203202 | 1.57E-53 | 9.60E-55 | down |
| VIT_18s0122g00550.t01 | 1144 | cyclin-dependent kinase B2-1-like | 62 | 3.659739 | 106 | 8.423842 | -1.202738 | 3.06E-07 | 9.45E-08 | down |
| VIT_13s0067g03290.t01 | 878 | 50S ribosomal protein L10, chloroplastic-like | 1346 | 103.52256 | 2301 | 238.26073 | -1.202596 | 7.30E-135 | 1.77E-136 | down |
| VIT_06s0004g02040.t01 | 1768 | RNA recognition motif containing protein, putative, expressed | 110 | 4.2014081 | 188 | 9.667317 | -1.202243 | 5.64E-12 | 1.20E-12 | down |
| VIT_07s0129g01080.t01 | 1020 | microtubule-associated protein RP/EB family member 1-like | 55 | 3.6412203 | 94 | 8.3783414 | -1.202243 | 1.52E-06 | 5.06E-07 | down |
| VIT_14s0171g00250.t01 | 985 | 50S ribosomal protein L13-like | 82 | 5.6216275 | 140 | 12.921775 | -1.200745 | 3.45E-09 | 9.04E-10 | down |
| VIT_19s0014g01490.t01 | 1278 | probable beta-1,3-galactosyltransferase 14-like | 116 | 6.1293099 | 198 | 14.085255 | -1.200389 | 1.60E-12 | 3.27E-13 | down |
| VIT_01s0010g01410.t01 | 1474 | zinc finger Ran-binding domain-containing protein 2, putative, expressed | 116 | 5.3142863 | 198 | 12.212317 | -1.200389 | 1.60E-12 | 3.27E-13 | down |
| VIT_06s0009g00210.t01 | 1656 | protein IQ-DOMAIN 1-like | 177 | 7.2176759 | 302 | 16.579712 | -1.199813 | 1.60E-18 | 2.45E-19 | down |
| VIT_07s0151g00900.t01 | 612 | 40S ribosomal protein S24-2-like | 211 | 23.281742 | 360 | 53.478775 | -1.199767 | 7.20E-22 | 9.58E-23 | down |
| VIT_12s0028g03810.t01 | 1676 | Putative uncharacterized protein | 85 | 3.4247538 | 145 | 7.8654642 | -1.199532 | 1.83E-09 | 4.70E-10 | down |
| VIT_00s2608g00010.t01 | 2148 | psbB; photosystem II CP47 chlorophyll apoprotein | 5455 | 171.49242 | 9299 | 393.57939 | -1.19851 | 0 | 0 | down |
| VIT_08s0007g05040.t01 | 830 | glutamyl-tRNA reductase-binding protein | 71 | 5.776499 | 121 | 13.253716 | -1.19813 | 4.58E-08 | 1.31E-08 | down |
| VIT_04s0008g05590.t01 | 793 | ATPF0B, atpF; F-type H+-transporting ATPase subunit b | 2765 | 235.45417 | 4711 | 540.09517 | -1.197767 | 1.26E-273 | 1.53E-275 | down |
| VIT_09s0018g01970.t01 | 1268 | expressed protein | 499 | 26.574539 | 850 | 60.943872 | -1.197437 | 4.49E-50 | 2.94E-51 | down |
| VIT_10s0092g00760.t01 | 954 | mitochondrial genome | 1652 | 116.93543 | 2813 | 268.07217 | -1.196909 | 1.77E-163 | 3.59E-165 | down |
| VIT_18s0001g09710.t01 | 1119 | chromatin modification-related protein EAF3, putative, expressed | 37 | 2.2328322 | 63 | 5.1184779 | -1.19684 | 0.0001013 | 4.16E-05 | down |
| VIT_04s0008g03260.t01 | 1062 | Thioredoxin | 37 | 2.3526734 | 63 | 5.3931985 | -1.19684 | 0.0001013 | 4.16E-05 | down |
| VIT_09s0018g00290.t01 | 1635 | Putative uncharacterized protein | 588 | 24.28533 | 1001 | 55.660447 | -1.196567 | 1.00E-58 | 5.61E-60 | down |
| VIT_15s0046g00320.t01 | 2413 | helix-loop-helix DNA-binding domain containing protein, expressed | 779 | 21.800406 | 1326 | 49.959326 | -1.196399 | 1.83E-77 | 7.82E-79 | down |
| VIT_04s0044g01240.t01 | 2088 | H-BTB2 - Bric-a-Brac, Tramtrack, Broad Complex BTB domain with H family con | 419 | 13.550895 | 713 | 31.044839 | -1.195965 | 4.38E-42 | 3.35E-43 | down |
| VIT_00s0250g00040.t01 | 1630 | Putative uncharacterized protein | 87 | 3.6042598 | 148 | 8.2547606 | -1.195523 | 1.37E-09 | 3.47E-10 | down |
| VIT_05s0049g01480.t01 | 641 | ribosomal protein L28 protein, putative, expressed | 522 | 54.991671 | 888 | 125.94627 | -1.195523 | 3.87E-52 | 2.44E-53 | down |
| VIT_11s0016g03900.t01 | 1700 | cbxX, chromosomal, putative, expressed | 97 | 3.8530732 | 165 | 8.8239979 | -1.195423 | 1.46E-10 | 3.44E-11 | down |
| VIT_12s0028g03740.t01 | 626 | protein of unknown function DUF1421 domain containing protein, expressed | 50 | 5.3936171 | 85 | 12.344542 | -1.194548 | 5.66E-06 | 2.00E-06 | down |
| VIT_16s0039g01020.t01 | 1343 | adenylate cyclase, terminal-differentiation specific | 100 | 5.0281524 | 170 | 11.508091 | -1.194548 | 7.74E-11 | 1.79E-11 | down |

| | | | | | | | | | | |
|---|---|---|---|---|---|---|---|---|---|---|
| VIT_15s0046g01350.t01 | 2187 | tubulin/FtsZ domain containing protein, putative, expressed | 120 | 3.7052448 | 204 | 8.4803106 | -1.194548 | 8.87E-13 | 1.79E-13 | down |
| VIT_10s0116g01640.t01 | 2201 | glucan endo-1,3-beta-glucosidase precursor, putative, expressed | 366 | 11.229114 | 622 | 25.692166 | -1.194084 | 9.32E-37 | 8.09E-38 | down |
| VIT_11s0016g02880.t01 | 2099 | no apical meristem protein, putative, expressed | 73 | 2.3485233 | 124 | 5.3708079 | -1.193385 | 3.42E-08 | 9.65E-09 | down |
| VIT_03s0097g00560.t01 | 525 | transcription factor GTE7-like | 179 | 23.023862 | 304 | 52.643488 | -1.193125 | 1.76E-18 | 2.68E-19 | down |
| VIT_06s0004g03400.t01 | 2584 | myosin heavy chain-related, putative, expressed | 338 | 8.8330082 | 574 | 20.195274 | -1.193041 | 5.95E-34 | 5.50E-35 | down |
| VIT_03s0091g00830.t01 | 1308 | splicing factor, putative, expressed | 86 | 4.4399201 | 146 | 10.147884 | -1.192573 | 1.92E-09 | 4.92E-10 | down |
| VIT_02s0025g00720.t01 | 1422 | GDT1-like protein 1 | 334 | 15.861027 | 567 | 36.250486 | -1.192514 | 1.58E-33 | 1.48E-34 | down |
| VIT_01s0011g03010.t01 | 1868 | serine/threonine-protein kinase SNT7, chloroplast precursor, putative, expressed | 598 | 21.617664 | 1015 | 49.399158 | -1.192276 | 3.36E-59 | 1.87E-60 | down |
| VIT_17s0000g01070.t01 | 1361 | hydrolase, alpha/beta fold family domain containing protein, expressed | 66 | 3.2746905 | 112 | 7.4815274 | -1.191974 | 1.70E-07 | 5.12E-08 | down |
| VIT_00s0375g00030.t01 | 2316 | alpha-galactosidase precursor, putative, expressed | 168 | 4.8984104 | 285 | 11.187593 | -1.191514 | 2.33E-17 | 3.74E-18 | down |
| VIT_01s0011g06330.t01 | 1800 | microtubule associated protein, putative, expressed | 191 | 7.1654803 | 324 | 16.364505 | -1.191435 | 1.38E-19 | 2.02E-20 | down |
| VIT_08s0007g05710.t01 | 2141 | FAD-binding and arabino-lactone oxidase domains containing protein, putative, expr | 217 | 6.8442759 | 368 | 15.626493 | -1.191024 | 4.36E-22 | 5.76E-23 | down |
| VIT_01s0244g00020.t01 | 1781 | tRNA nucleotidyltransferase/polyA polymerase family protein, putative, expressed | 105 | 3.9811618 | 178 | 9.0862871 | -1.190501 | 3.06E-11 | 6.88E-12 | down |
| VIT_16s0098g00600.t01 | 587 | MAP7 domain-containing protein 1-like | 321 | 36.927625 | 544 | 84.254125 | -1.190047 | 4.16E-32 | 4.04E-33 | down |
| VIT_14s0128g00750.t01 | 2708 | peptidase, M24 family protein, putative, expressed | 304 | 7.580701 | 515 | 17.289759 | -1.189515 | 1.98E-30 | 2.01E-31 | down |
| VIT_10s0071g00990.t01 | 732 | CHCH domain containing protein, expressed | 49 | 4.5203227 | 83 | 10.308545 | -1.189343 | 7.96E-06 | 2.86E-06 | down |
| VIT_09s0002g05120.t01 | 2191 | zinc finger family protein, putative, expressed | 101 | 3.1128876 | 171 | 7.095518 | -1.188655 | 8.10E-11 | 1.87E-11 | down |
| VIT_18s0001g08640.t01 | 1685 | RNA-binding protein Musashi homolog 2-like | 114 | 4.5686658 | 193 | 10.413286 | -1.188581 | 4.60E-12 | 9.73E-13 | down |
| VIT_05s0094g00390.t01 | 1084 | Putative uncharacterized protein | 270 | 16.819726 | 457 | 38.3281 | -1.188248 | 4.49E-27 | 5.00E-28 | down |
| VIT_09s0002g04230.t01 | 756 | Putative uncharacterized protein | 52 | 4.644789 | 88 | 10.582572 | -1.188005 | 4.22E-06 | 1.47E-06 | down |
| VIT_00s0580g00010.t01 | 2280 | CSLC7 - cellulose synthase-like family C, expressed | 195 | 5.7754284 | 330 | 13.158593 | -1.188005 | 7.67E-20 | 1.11E-20 | down |
| VIT_17s0000g01790.t01 | 2106 | 4-coumarate--CoA ligase-like 9 | 149 | 4.7776281 | 252 | 10.878588 | -1.187125 | 2.16E-15 | 3.82E-16 | down |
| VIT_19s0014g04710.t01 | 1019 | DAG protein, chloroplast precursor, putative, expressed | 993 | 65.805093 | 1679 | 149.7983 | -1.18675 | 1.31E-96 | 4.53E-98 | down |
| VIT_14s0068g00720.t01 | 3255 | TUDOR protein with multiple SNc domains, putative, expressed | 487 | 10.103281 | 823 | 22.986837 | -1.185984 | 8.47E-48 | 5.81E-49 | down |
| VIT_07s0005g00240.t01 | 1388 | WD domain, G-beta repeat domain containing protein, expressed | 174 | 8.4653365 | 294 | 19.256983 | -1.185742 | 9.60E-18 | 1.51E-18 | down |
| VIT_10s0003g02860.t01 | 1072 | UDP-N-acetylglucosamine transferase subunit ALG14, putative, expressed | 58 | 3.6535718 | 98 | 8.3111604 | -1.185742 | 1.19E-06 | 3.89E-07 | down |
| VIT_01s0010g02430.t01 | 1115 | mitotic spindle checkpoint protein MAD2, putative, expressed | 58 | 3.5126718 | 98 | 7.9906403 | -1.185742 | 1.19E-06 | 3.89E-07 | down |
| VIT_18s0122g00490.t01 | 324 | splicing factor U2af small subunit B | 29 | 6.0441806 | 49 | 13.749327 | -1.185742 | 0.00071541 | 0.00033334 | down |
| VIT_15s0048g00550.t01 | 1016 | YABBY domain containing protein, putative, expressed | 438 | 29.111518 | 740 | 66.21683 | -1.185608 | 4.67E-43 | 3.51E-44 | down |
| VIT_08s0056g00120.t01 | 1794 | cytidylyltransferase domain containing protein, expressed | 135 | 5.081545 | 228 | 11.554277 | -1.185088 | 5.42E-14 | 1.03E-14 | down |
| VIT_08s0056g01070.t01 | 1708 | MATE efflux family protein | 241 | 9.5282604 | 407 | 21.663914 | -1.185009 | 3.98E-24 | 4.87E-25 | down |
| VIT_01s0026g01490.t01 | 2090 | peptide transporter PTR2, putative, expressed | 106 | 3.4248695 | 179 | 7.7864073 | -1.184909 | 3.20E-11 | 7.19E-12 | down |
| VIT_18s0001g12240.t01 | 1912 | eukaryotic peptide chain release factor subunit 1-1, putative, expressed | 545 | 19.24833 | 920 | 43.74519 | -1.184391 | 3.38E-53 | 2.07E-54 | down |
| VIT_03s0017g02300.t01 | 1375 | Putative uncharacterized protein | 285 | 13.996731 | 481 | 31.803341 | -1.184088 | 2.75E-28 | 2.94E-29 | down |
| VIT_01s0010g03490.t01 | 868 | caffeoyl-CoA O-methyltransferase, putative, expressed | 32 | 2.4895147 | 54 | 5.655935 | -1.183901 | 0.00037834 | 0.00016865 | down |
| VIT_07s0104g01220.t01 | 772 | Putative uncharacterized protein | 48 | 4.1986375 | 81 | 9.5388955 | -1.183901 | 1.12E-05 | 4.08E-06 | down |
| VIT_14s0068g02160.t01 | 772 | ribosome, putative, expressed | 1396 | 122.11037 | 2354 | 277.21679 | -1.182829 | 2.16E-134 | 5.26E-136 | down |
| VIT_07s0104g00730.t01 | 1120 | Putative uncharacterized protein | 277 | 16.701143 | 467 | 37.907857 | -1.18255 | 1.93E-27 | 2.12E-28 | down |
| VIT_01s0146g00250.t01 | 1787 | protein kinase domain containing protein, expressed | 374 | 14.132907 | 630 | 32.051353 | -1.181327 | 1.32E-36 | 1.15E-37 | down |
| VIT_17s0000g09390.t01 | 1036 | 50S ribosomal protein L19, chloroplast precursor, putative, expressed | 180 | 11.732679 | 303 | 26.589688 | -1.180334 | 3.95E-18 | 6.12E-19 | down |
| VIT_05s0094g00150.t01 | 896 | membrane-associated 30 kDa protein, chloroplast precursor, putative, expressed | 41 | 3.0900129 | 69 | 7.0011834 | -1.179986 | 5.61E-05 | 2.22E-05 | down |
| VIT_15s0046g01090.t01 | 1728 | rhodanese-like domain-containing protein 4 | 2282 | 89.177716 | 3839 | 201.97832 | -1.179445 | 1.05E-217 | 1.55E-219 | down |
| VIT_16s0100g00490.t01 | 1602 | CCB2, putative, expressed | 195 | 8.2197109 | 328 | 18.614086 | -1.179235 | 1.65E-19 | 2.41E-20 | down |
| VIT_09s0002g01800.t01 | 2088 | 2-oxo acid dehydrogenases acyltransferase domain containing protein, expressed | 217 | 7.0180052 | 365 | 15.892519 | -1.179215 | 1.38E-21 | 1.85E-22 | down |
| VIT_19s0090g01150.t01 | 592 | SWIB/MDM2 domain containing protein, expressed | 44 | 5.0189794 | 74 | 11.36424 | -1.179035 | 2.96E-05 | 1.14E-05 | down |
| VIT_18s0001g07970.t01 | 2679 | pentatricopeptide, putative, expressed | 110 | 2.7727098 | 185 | 6.2781167 | -1.179035 | 1.77E-11 | 3.92E-12 | down |
| VIT_09s0002g07290.t01 | 1853 | signal recognition particle 54 kDa protein, putative, expressed | 132 | 4.8104195 | 222 | 10.892007 | -1.179035 | 1.50E-13 | 2.90E-14 | down |
| VIT_02s0012g01050.t01 | 1128 | protein ABCI7 | 383 | 22.928419 | 644 | 51.904754 | -1.17873 | 2.88E-37 | 2.47E-38 | down |
| VIT_05s0051g00070.t01 | 1590 | uncharacterized protein all1601, putative, expressed | 182 | 7.72963 | 306 | 17.496641 | -1.178607 | 2.93E-18 | 4.53E-19 | down |
| VIT_07s0005g03730.t01 | 2346 | serine/threonine-protein phosphatase 2A regulatory subunit B'' subunit alpha-like | 153 | 4.4040056 | 257 | 9.959453 | -1.17725 | 1.76E-15 | 3.08E-16 | down |
| VIT_08s0007g08130.t01 | 1805 | adenylosuccinate synthetase 2 | 527 | 19.715957 | 885 | 44.575522 | -1.176888 | 1.04E-50 | 6.73E-52 | down |
| VIT_04s0008g01950.t01 | 990 | YGGT family protein, putative, expressed | 870 | 59.342864 | 1461 | 134.1669 | -1.176882 | 4.37E-83 | 1.76E-84 | down |
| VIT_08s0007g06540.t01 | 1643 | nudix hydrolase 23 | 56 | 2.3016268 | 94 | 5.2014049 | -1.176247 | 2.33E-06 | 7.90E-07 | down |
| VIT_08s0007g01520.t01 | 1889 | protein kinase family protein, putative, expressed | 230 | 8.2220539 | 386 | 18.577434 | -1.17598 | 1.13E-22 | 1.46E-23 | down |
| VIT_17s0000g03720.t01 | 966 | LOB domain-containing protein 41-like | 149 | 10.415823 | 250 | 23.528447 | -1.175629 | 4.62E-15 | 8.33E-16 | down |
| VIT_12s0057g00940.t01 | 2010 | glucose-6-phosphate 1-dehydrogenase, chloroplast precursor, putative, expressed | 239 | 8.029459 | 401 | 18.137553 | -1.175605 | 1.68E-23 | 2.10E-24 | down |
| VIT_03s0038g04010.t01 | 1284 | pantothenate kinase 4, putative, expressed | 155 | 8.151755 | 260 | 18.40936 | -1.175257 | 1.30E-15 | 2.27E-16 | down |
| VIT_14s0066g01050.t01 | 1775 | CGMC_GSK.1 - CGMC includes CDA, MAPK, GSK3, and CLKC kinases, expres | 266 | 10.119702 | 446 | 22.843722 | -1.174631 | 5.50E-26 | 6.33E-27 | down |
| VIT_00s0246g00190.t01 | 568 | NADPH-dependent oxidoreductase, putative, expressed | 200 | 23.777495 | 335 | 53.620004 | -1.173175 | 9.49E-20 | 1.38E-20 | down |
| VIT_01s0137g00290.t01 | 2582 | oxysterol-binding protein, putative, expressed | 203 | 5.3091408 | 340 | 11.971624 | -1.173069 | 5.02E-20 | 7.20E-21 | down |
| VIT_06s0080g00780.t01 | 991 | no apical meristem protein, putative, expressed | 86 | 5.8601568 | 144 | 13.210499 | -1.172674 | 4.11E-09 | 1.08E-09 | down |
| VIT_17s0000g00370.t01 | 1404 | serine carboxypeptidase-like 45-like | 86 | 4.1363358 | 144 | 9.3245044 | -1.172674 | 4.11E-09 | 1.08E-09 | down |
| VIT_11s0052g00420.t01 | 1256 | NADP-dependent oxidoreductase, putative, expressed | 150 | 8.06466 | 251 | 18.168307 | -1.171738 | 4.81E-15 | 8.69E-16 | down |
| VIT_12s0142g00130.t01 | 2333 | CGMC_MAPKCMGC_2.4 - CGMC includes CDA, MAPK, GSK3, and CLKC kir | 220 | 6.3678436 | 368 | 14.340472 | -1.171216 | 1.56E-21 | 2.10E-22 | down |
| VIT_11s0118g00810.t01 | 1086 | peptidyl-prolyl cis-trans isomerase, putative, expressed | 400 | 24.872223 | 669 | 56.004982 | -1.17102 | 2.78E-38 | 2.32E-39 | down |
| VIT_16s0039g02670.t01 | 1294 | translocase of chloroplast 34, putative, expressed | 116 | 6.0535224 | 194 | 13.630062 | -1.170945 | 7.30E-12 | 1.57E-12 | down |
| VIT_08s0007g01380.t01 | 2083 | ATROPGEF7/ROPGEF7, putative, expressed | 134 | 4.3441016 | 224 | 9.7766287 | -1.170279 | 1.63E-13 | 3.16E-14 | down |
| VIT_01s0026g01430.t01 | 2099 | Putative uncharacterized protein | 1196 | 38.477176 | 1999 | 86.582621 | -1.170075 | 2.57E-112 | 7.63E-114 | down |
| VIT_08s0007g01270.t01 | 787 | dynein light chain LC6 | 73 | 6.2637234 | 122 | 14.09339 | -1.169926 | 7.33E-08 | 2.13E-08 | down |
| VIT_05s0020g01670.t01 | 2056 | spo0B-associated GTP-binding protein, putative, expressed | 279 | 9.1635876 | 466 | 20.605975 | -1.169078 | 6.56E-27 | 7.35E-28 | down |
| VIT_12s0057g01260.t01 | 1324 | Putative uncharacterized protein | 336 | 17.137037 | 561 | 38.521683 | -1.168553 | 3.66E-32 | 3.55E-33 | down |
| VIT_18s0001g01620.t01 | 2183 | ELMO/CED-12 family protein, putative, expressed | 251 | 7.7643379 | 419 | 17.449808 | -1.168276 | 2.83E-24 | 3.44E-25 | down |
| VIT_07s0031g02310.t01 | 2755 | leucine rich repeat containing protein, expressed | 169 | 4.1423763 | 282 | 9.3058892 | -1.167685 | 1.12E-16 | 1.85E-17 | down |
| VIT_02s0012g01660.t01 | 3925 | P-glycoprotein-like | 404 | 6.9506616 | 674 | 15.611715 | -1.167407 | 2.24E-38 | 1.87E-39 | down |
| VIT_18s0041g02110.t01 | 1724 | Putative uncharacterized protein | 277 | 10.84993 | 462 | 24.363242 | -1.16702 | 1.29E-26 | 1.46E-27 | down |
| VIT_05s0077g02100.t01 | 550 | Putative uncharacterized protein | 48 | 5.8933603 | 80 | 13.223843 | -1.165979 | 1.64E-05 | 6.08E-06 | down |
| VIT_04s0044g01190.t01 | 1959 | ataxin-10 | 87 | 2.9989502 | 145 | 6.7292078 | -1.165979 | 4.28E-09 | 1.13E-09 | down |
| VIT_04s0008g04340.t01 | 1379 | glutamic acid-rich protein | 108 | 5.2886391 | 180 | 11.866936 | -1.165979 | 5.08E-11 | 1.16E-11 | down |
| VIT_10s0092g00780.t01 | 448 | mitochondrial genome | 75 | 11.304925 | 125 | 25.366606 | -1.165979 | 5.44E-08 | 1.56E-08 | down |
| VIT_17s0000g06970.t01 | 3430 | diacylglycerol kinase 1, putative, expressed | 329 | 6.4771838 | 548 | 14.525022 | -1.165102 | 2.69E-31 | 2.66E-32 | down |
| VIT_01s0010g01200.t01 | 2093 | FAD-linked oxidoreductase protein, putative, expressed | 1414 | 45.620981 | 2354 | 102.25101 | -1.164346 | 4.38E-131 | 1.10E-132 | down |
| VIT_00s0323g00080.t01 | 2534 | elongation factor, putative, expressed | 784 | 20.892668 | 1305 | 46.820309 | -1.164138 | 4.71E-73 | 2.16E-74 | down |
| VIT_14s0068g01020.t01 | 978 | CCB1, putative, expressed | 521 | 35.973551 | 867 | 80.595467 | -1.163762 | 7.77E-49 | 5.21E-50 | down |
| VIT_10s0003g00420.t01 | 2570 | auxin response factor 3 | 357 | 9.3803606 | 594 | 21.012789 | -1.163552 | 9.07E-34 | 8.43E-35 | down |
| VIT_09s0002g07500.t01 | 1472 | hydrolase, alpha/beta fold family domain containing protein, expressed | 309 | 14.175393 | 514 | 31.745757 | -1.163175 | 2.42E-29 | 2.52E-30 | down |
| VIT_15s0045g00630.t01 | 1555 | ribosomal protein S15 containing protein, expressed | 184 | 7.9904617 | 306 | 17.890456 | -1.162839 | 6.79E-18 | 1.07E-18 | down |
| VIT_14s0006g01890.t01 | 1578 | ell-associated factor Eaf-like | 77 | 3.2950967 | 128 | 7.374513 | -1.162227 | 4.04E-08 | 1.15E-08 | down |
| VIT_17s0000g08680.t01 | 3465 | Putative uncharacterized protein | 290 | 5.6517013 | 482 | 12.646611 | -1.161994 | 1.55E-27 | 1.69E-28 | down |
| VIT_07s0005g01740.t01 | 1751 | zinc knuckle family protein, putative, expressed | 142 | 5.4762926 | 236 | 12.253389 | -1.161909 | 4.95E-14 | 9.35E-15 | down |
| VIT_15s0024g00630.t01 | 444 | pyruvate decarboxylase 2 | 68 | 10.342139 | 113 | 23.138002 | -1.16173 | 2.70E-07 | 8.30E-08 | down |
| VIT_14s0171g00310.t01 | 2279 | histone-lysine N-methyltransferase setd3-like | 502 | 14.87455 | 834 | 33.269946 | -1.161374 | 7.27E-47 | 5.07E-48 | down |
| VIT_15s0024g00050.t01 | 1276 | deoxyribonuclease tatD, putative, expressed | 59 | 3.1223802 | 98 | 6.9824169 | -1.16108 | 1.80E-06 | 6.04E-07 | down |
| VIT_06s0009g01600.t01 | 1605 | DUF630/DUF632 domains containing protein, putative, expressed | 56 | 2.3561202 | 93 | 5.2679092 | -1.160817 | 3.41E-06 | 1.17E-06 | down |
| VIT_02s0025g01500.t01 | 1012 | nucleoside diphosphate kinase, putative, expressed | 445 | 29.693674 | 739 | 66.38872 | -1.160783 | 1.31E-41 | 1.02E-42 | down |
| VIT_16s0022g01340.t01 | 2322 | deoxyribodipyrimidine photolyase family protein, expressed | 218 | 6.3398462 | 362 | 14.173488 | -1.160675 | 6.54E-21 | 9.02E-22 | down |
| VIT_01s0011g03760.t01 | 1037 | Putative uncharacterized protein | 53 | 3.4512908 | 88 | 7.7149708 | -1.160525 | 6.42E-06 | 2.28E-06 | down |
| VIT_07s0031g02750.t01 | 2221 | CBS domain-containing protein CBSCBSPB3-like | 362 | 11.006379 | 601 | 24.6012 | -1.160389 | 5.20E-34 | 4.80E-35 | down |

| | | | | | | | | | | |
|---|---|---|---|---|---|---|---|---|---|---|
| VIT_07s0005g05610.t01 | 1612 | 3-ketoacyl-CoA thiolase, peroxisomal precursor, putative, expressed | 194 | 8.1268293 | 322 | 18.160224 | -1.160018 | 1.06E-18 | 1.59E-19 | down |
| VIT_03s0180g00170.t01 | 1100 | reticulon domain containing protein, putative, expressed | 97 | 5.9547494 | 161 | 13.306492 | -1.160018 | 6.64E-10 | 1.65E-10 | down |
| VIT_19s0090g01690.t01 | 1276 | STE_PAK_Ste20_Slob_Wnk.5 - STE kinases include homologs to sterile 7, sterile 1 | 47 | 2.4873198 | 78 | 5.5574338 | -1.159827 | 2.29E-05 | 8.68E-06 | down |
| VIT_18s0001g12840.t01 | 1811 | glucose-1-phosphate adenylyltransferase large subunit, putative, expressed | 138 | 5.1457073 | 229 | 11.496017 | -1.159693 | 1.31E-13 | 2.53E-14 | down |
| VIT_13s0067g00620.t01 | 1133 | cinnamoyl-CoA reductase 1-like | 44 | 2.62245 | 73 | 5.8576487 | -1.159406 | 4.32E-05 | 1.70E-05 | down |
| VIT_12s0142g00670.t01 | 2502 | DEAD-box ATP-dependent RNA helicase 53-like | 566 | 15.276138 | 939 | 34.119971 | -1.159337 | 1.72E-52 | 1.07E-53 | down |
| VIT_04s0008g01690.t01 | 1313 | nitrilase, putative, expressed | 214 | 11.006101 | 355 | 24.580686 | -1.159222 | 1.73E-20 | 2.43E-21 | down |
| VIT_08s0040g03220.t01 | 1294 | myb-like DNA-binding domain containing protein, expressed | 79 | 4.1226575 | 131 | 9.2038046 | -1.158656 | 3.00E-08 | 8.44E-09 | down |
| VIT_00s1217g00010.t01 | 1766 | 3-deoxy-D-arabino-heptulosonate-7-phosphate synthase 03 | 494 | 18.88951 | 819 | 42.16223 | -1.158366 | 7.43E-46 | 5.30E-47 | down |
| VIT_03s0017g00680.t01 | 1717 | NAD dependent epimerase/dehydratase family protein, putative, expressed | 820 | 32.249872 | 1359 | 71.958075 | -1.157863 | 2.20E-75 | 9.62E-77 | down |
| VIT_13s0156g00360.t01 | 1975 | ENT domain containing protein, expressed | 122 | 4.1713552 | 202 | 9.2985374 | -1.156488 | 4.38E-12 | 9.25E-13 | down |
| VIT_19s0014g05110.t01 | 1096 | Putative uncharacterized protein | 148 | 9.1187562 | 245 | 20.32291 | -1.156198 | 2.02E-14 | 3.75E-15 | down |
| VIT_06s0004g07380.t01 | 1471 | malonyl CoA-acyl carrier protein transacylase, mitochondrial precursor, putative, ex | 391 | 17.949342 | 647 | 39.987291 | -1.155611 | 2.65E-36 | 2.32E-37 | down |
| VIT_07s0104g00460.t01 | 2283 | ATP/GTP/Ca++ binding protein, putative, expressed | 165 | 4.8804793 | 273 | 10.871441 | -1.155448 | 6.27E-16 | 1.08E-16 | down |
| VIT_14s0066g00790.t01 | 3090 | RNA-binding motif protein, putative, expressed | 191 | 4.1740662 | 316 | 9.2973456 | -1.155365 | 2.89E-18 | 4.46E-19 | down |
| VIT_18s0089g00590.t01 | 1382 | acetyl-CoA acetyltransferase | 81 | 3.957869 | 134 | 8.8150977 | -1.155253 | 2.22E-08 | 6.20E-09 | down |
| VIT_16s0039g00740.t01 | 2011 | SET domain containing protein, expressed | 159 | 5.3391177 | 263 | 11.889786 | -1.15505 | 2.23E-15 | 3.94E-16 | down |
| VIT_01s0011g00210.t01 | 720 | probable cytochrome b5 isoform 2-like | 104 | 9.7540569 | 172 | 21.718325 | -1.154839 | 1.95E-10 | 4.64E-11 | down |
| VIT_14s0060g00820.t01 | 1321 | 3-beta hydroxysteroid dehydrogenase/isomerase family protein, putative, expressed | 2065 | 105.56056 | 3415 | 235.02727 | -1.154757 | 2.39E-187 | 4.27E-189 | down |
| VIT_01s0011g02430.t01 | 1249 | HD domain containing protein 2, putative, expressed | 101 | 5.4606379 | 167 | 12.155824 | -1.154506 | 3.67E-10 | 8.92E-11 | down |
| VIT_17s0000g09070.t01 | 1846 | histone deacetylase, putative, expressed | 124 | 4.5360145 | 205 | 10.096074 | -1.154297 | 3.25E-12 | 6.81E-13 | down |
| VIT_07s0151g00960.t01 | 1001 | WD domain, G-beta repeat domain containing protein, expressed | 98 | 6.6111413 | 162 | 14.713341 | -1.154154 | 6.90E-10 | 1.72E-10 | down |
| VIT_12s0055g01110.t01 | 1098 | LHCB6; light-harvesting complex II chlorophyll a/b binding protein 6 | 22293 | 1371.0416 | 36848 | 3050.9982 | -1.154009 | 0 | 0 | down |
| VIT_04s0044g01690.t01 | 2675 | tRNA uridine 5-carboxymethylaminomethyl modification enzyme gidA, putative, exp | 118 | 2.978809 | 195 | 6.6273697 | -1.153701 | 1.15E-11 | 2.51E-12 | down |
| VIT_15s0048g01660.t01 | 1051 | RNA polymerase-associated protein LEO1 | 66 | 4.2405839 | 109 | 9.4287507 | -1.152804 | 5.30E-07 | 1.67E-07 | down |
| VIT_14s0060g01460.t01 | 1773 | exopolyphosphatase-like | 66 | 2.5137359 | 109 | 5.5891805 | -1.152804 | 5.30E-07 | 1.67E-07 | down |
| VIT_10s0003g02830.t01 | 1568 | CAS1 domain-containing protein 1 precursor, putative, expressed | 83 | 3.5745097 | 137 | 7.9433716 | -1.152006 | 1.65E-08 | 4.55E-09 | down |
| VIT_07s0005g03510.t01 | 1416 | rho termination factor, N-terminal domain containing protein, expressed | 163 | 7.7733602 | 269 | 17.271076 | -1.151748 | 1.23E-15 | 2.14E-16 | down |
| VIT_03s0038g00350.t01 | 3854 | kinesin motor domain containing protein, putative, expressed | 183 | 3.2064452 | 302 | 7.1240278 | -1.151718 | 2.02E-17 | 3.23E-18 | down |
| VIT_08s0105g00350.t01 | 1222 | dnaJ homolog subfamily B member 1 | 223 | 12.323047 | 368 | 27.378332 | -1.151676 | 5.45E-21 | 7.49E-22 | down |
| VIT_05s0094g00060.t01 | 1669 | Putative uncharacterized protein | 80 | 3.2368166 | 132 | 7.1903158 | -1.15148 | 3.12E-08 | 8.78E-09 | down |
| VIT_01s0010g00590.t01 | 1410 | chlorophyll(ide) b reductase NOL | 131 | 6.273886 | 216 | 13.927238 | -1.150478 | 9.54E-13 | 1.93E-13 | down |
| VIT_00s0555g00020.t01 | 1665 | cytochrome P450 93A1-like | 279 | 11.315517 | 460 | 25.117359 | -1.150382 | 6.31E-26 | 7.28E-27 | down |
| VIT_01s0026g00640.t01 | 2016 | DEAD-box ATP-dependent RNA helicase 22-like | 162 | 5.4263641 | 267 | 12.040683 | -1.149859 | 1.72E-15 | 3.02E-16 | down |
| VIT_12s0035g01420.t01 | 1134 | plus-3 domain containing protein, putative, expressed | 159 | 9.4682238 | 262 | 21.004803 | -1.149554 | 3.23E-15 | 5.78E-16 | down |
| VIT_09s0018g01880.t01 | 905 | cell wall integrity protein scw1 | 204 | 15.221801 | 336 | 33.753675 | -1.148906 | 3.45E-19 | 5.10E-20 | down |
| VIT_04s0023g02460.t01 | 1874 | WD domain, G-beta repeat domain containing protein, expressed | 153 | 5.5132322 | 252 | 12.225351 | -1.148906 | 1.15E-14 | 2.12E-15 | down |
| VIT_04s0023g02990.t01 | 1786 | Putative uncharacterized protein | 150 | 5.6714518 | 247 | 12.573201 | -1.148562 | 2.18E-14 | 4.05E-15 | down |
| VIT_17s0053g00090.t01 | 2509 | plastid division protein CDP1, chloroplastic-like | 130 | 3.4988646 | 214 | 7.7543158 | -1.148113 | 1.33E-12 | 2.72E-13 | down |
| VIT_18s0001g09580.t01 | 1384 | putative cyclin-A3-1-like | 48 | 2.3420146 | 79 | 5.1894505 | -1.147832 | 2.38E-05 | 9.03E-06 | down |
| VIT_09s0002g00920.t01 | 1956 | transmembrane protein 87A-like | 414 | 14.292754 | 681 | 31.652545 | -1.147038 | 1.02E-37 | 8.66E-39 | down |
| VIT_13s0064g00200.t01 | 984 | uncharacterized UPF0114 domain containing protein, expressed | 276 | 18.940805 | 454 | 41.946055 | -1.147038 | 1.73E-25 | 2.03E-26 | down |
| VIT_07s0005g04940.t01 | 3340 | Putative uncharacterized protein | 329 | 6.6517187 | 541 | 14.725877 | -1.146555 | 3.73E-30 | 3.82E-31 | down |
| VIT_15s0048g01750.t01 | 1546 | fasciclin-like arabinogalactan protein, putative, expressed | 2237 | 97.710433 | 3678 | 216.28809 | -1.14637 | 2.24E-199 | 3.65E-201 | down |
| VIT_13s0019g04390.t01 | 1767 | GATA zinc finger domain containing protein, expressed | 2565 | 98.024642 | 4217 | 216.96887 | -1.146272 | 1.87E-228 | 2.59E-230 | down |
| VIT_11s0052g00600.t01 | 2772 | pentatricopeptide, putative, expressed | 101 | 2.4604389 | 166 | 5.4443399 | -1.145841 | 5.33E-10 | 1.31E-10 | down |
| VIT_00s0734g00030.t01 | 598 | Putative uncharacterized protein | 101 | 11.405245 | 166 | 25.236974 | -1.145841 | 5.33E-10 | 1.31E-10 | down |
| VIT_18s0001g04190.t01 | 1257 | co-chaperone GrpE protein, putative, expressed | 101 | 5.4258844 | 166 | 12.006134 | -1.145841 | 5.33E-10 | 1.31E-10 | down |
| VIT_12s0057g00400.t01 | 2046 | threonyl-tRNA synthetase, putative, expressed | 230 | 7.5911339 | 378 | 16.796413 | -1.145766 | 2.30E-21 | 3.13E-22 | down |
| VIT_12s0028g01250.t01 | 1362 | ATP-dependent Clp protease proteolytic subunit 4 | 516 | 25.583328 | 848 | 56.60426 | -1.145707 | 1.24E-46 | 8.70E-48 | down |
| VIT_11s0016g02530.t01 | 1425 | methyltransferase, putative, expressed | 157 | 7.4399365 | 258 | 16.460204 | -1.14562 | 6.32E-15 | 1.15E-15 | down |
| VIT_04s0043g00160.t01 | 3067 | receptor protein kinase TMK1 precursor, putative, expressed | 569 | 12.528034 | 935 | 27.71585 | -1.145551 | 2.66E-51 | 1.70E-52 | down |
| VIT_17s0000g03980.t01 | 1060 | peptidyl-tRNA hydrolase, mitochondrial precursor protein, putative, expressed | 95 | 6.0520455 | 156 | 13.379784 | -1.14456 | 1.89E-09 | 4.85E-10 | down |
| VIT_11s0052g01190.t01 | 1238 | glycosyl hydrolases family 16, putative, expressed | 120 | 6.5455334 | 197 | 14.466916 | -1.144175 | 1.24E-11 | 2.71E-12 | down |
| VIT_17s0000g06990.t01 | 622 | 60S ribosomal protein L8, putative, expressed | 917 | 99.555073 | 1505 | 219.9766 | -1.143783 | 8.62E-82 | 3.54E-83 | down |
| VIT_05s0020g04520.t01 | 1444 | AT-rich interactive domain-containing protein 2-like | 117 | 5.4714585 | 192 | 12.088277 | -1.143611 | 2.33E-11 | 5.20E-12 | down |
| VIT_19s0090g00810.t01 | 1832 | imidazole glycerol phosphate synthase hisHF, chloroplast precursor, putative, expres | 89 | 3.2805675 | 146 | 7.2453231 | -1.143105 | 6.72E-09 | 1.80E-09 | down |
| VIT_08s0056g00230.t01 | 1769 | Putative uncharacterized protein | 114 | 4.3517252 | 187 | 9.6104594 | -1.143018 | 4.40E-11 | 9.98E-12 | down |
| VIT_13s0073g00600.t01 | 2652 | protein kinase, putative, expressed | 111 | 2.8264018 | 182 | 6.2391904 | -1.142392 | 8.29E-11 | 1.92E-11 | down |
| VIT_02s0154g00070.t01 | 1039 | YABBY domain containing protein, putative, expressed | 172 | 11.178856 | 282 | 24.675385 | -1.1423 | 3.87E-16 | 6.56E-17 | down |
| VIT_12s0035g00760.t01 | 1560 | ATP phosphoribosyltransferase, putative, expressed | 355 | 15.366968 | 582 | 33.917885 | -1.142214 | 3.59E-32 | 3.48E-33 | down |
| VIT_09s0002g00990.t01 | 2044 | lipase class 3 family protein, putative, expressed | 97 | 3.2046108 | 159 | 7.0720709 | -1.141984 | 1.40E-09 | 3.56E-10 | down |
| VIT_15s0048g01300.t01 | 2046 | glycosyl transferase family 8, putative, expressed | 310 | 10.231528 | 507 | 22.528522 | -1.138731 | 5.06E-28 | 5.44E-29 | down |
| VIT_08s0007g05890.t01 | 2361 | GTP binding protein, putative, expressed | 96 | 2.7457418 | 157 | 6.0455252 | -1.138672 | 1.96E-09 | 5.04E-10 | down |
| VIT_04s0008g06610.t01 | 1643 | enoyl-CoA hydratase/isomerase family protein, putative, expressed | 159 | 6.5349761 | 260 | 14.386865 | -1.138498 | 6.80E-15 | 1.24E-15 | down |
| VIT_18s0001g13100.t01 | 1712 | glycosyl transferase, group 1 domain containing protein, expressed | 137 | 5.4038247 | 224 | 11.895279 | -1.138336 | 5.66E-13 | 1.12E-13 | down |
| VIT_07s0005g02620.t01 | 1893 | bifunctional aminoacyl-tRNA synthetase, putative, expressed | 452 | 16.12398 | 739 | 35.491487 | -1.138265 | 2.35E-40 | 1.88E-41 | down |
| VIT_08s0007g01940.t01 | 1993 | glycosyl transferase, group 1 domain containing protein, expressed | 356 | 12.062217 | 582 | 26.548871 | -1.138155 | 5.41E-32 | 5.26E-33 | down |
| VIT_04s0044g00890.t01 | 1736 | amino acid kinase, putative, expressed | 153 | 5.9514961 | 250 | 13.092442 | -1.13741 | 2.44E-14 | 4.53E-15 | down |
| VIT_18s0001g13750.t01 | 1946 | RCC2, putative, expressed | 306 | 10.618497 | 500 | 23.359177 | -1.13741 | 1.34E-27 | 1.46E-28 | down |
| VIT_06s0061g00770.t01 | 898 | ribosomal L9, putative, expressed | 489 | 36.771976 | 799 | 80.891114 | -1.137375 | 1.70E-43 | 1.27E-44 | down |
| VIT_01s0010g02570.t01 | 2323 | FAD binding domain containing protein, expressed | 90 | 2.616241 | 147 | 5.7530546 | -1.136833 | 6.97E-09 | 1.87E-09 | down |
| VIT_08s0007g03160.t01 | 2028 | signal recognition particle 54 kDa protein, putative, expressed | 829 | 27.603937 | 1354 | 60.698937 | -1.136797 | 5.73E-73 | 2.64E-74 | down |
| VIT_17s0000g08390.t01 | 1675 | 1-deoxy-D-xylulose 5-phosphate reductoisomerase | 631 | 25.438939 | 1030 | 55.905275 | -1.135946 | 1.14E-55 | 6.75E-57 | down |
| VIT_18s0001g05700.t01 | 3228 | non-lysosomal glucosylceramidase, putative, expressed | 125 | 2.6149352 | 204 | 5.7454892 | -1.135655 | 7.08E-12 | 1.52E-12 | down |
| VIT_11s0016g01910.t01 | 1405 | WD domain, G-beta repeat domain containing protein, expressed | 133 | 6.3923384 | 217 | 14.041509 | -1.135282 | 1.49E-12 | 3.04E-13 | down |
| VIT_08s0040g02740.t01 | 1336 | pectate lyase family protein, expressed | 76 | 3.8414181 | 124 | 8.4381181 | -1.135282 | 1.19E-07 | 3.53E-08 | down |
| VIT_11s0037g01400.t01 | 1869 | RING-H2 finger protein, putative, expressed | 103 | 3.7214515 | 168 | 8.1720375 | -1.13483 | 5.72E-10 | 1.41E-10 | down |
| VIT_14s0066g01300.t01 | 1510 | membrane associated DUF588 domain containing protein, putative, expressed | 252 | 11.269588 | 411 | 24.745444 | -1.134728 | 8.52E-23 | 1.10E-23 | down |
| VIT_13s0073g00050.t01 | 1879 | amino acid permease family protein, putative, expressed | 65 | 2.3359902 | 106 | 5.1287255 | -1.134566 | 1.07E-06 | 3.50E-07 | down |
| VIT_13s0067g03810.t01 | 948 | cytochrome c oxidase subunit 6b-1-like | 300 | 21.369648 | 489 | 46.89547 | -1.133885 | 6.87E-27 | 7.70E-28 | down |
| VIT_03s0091g00250.t01 | 325 | Putative uncharacterized protein | 108 | 22.440103 | 176 | 49.233383 | -1.133558 | 2.25E-10 | 5.39E-11 | down |
| VIT_14s0068g02200.t01 | 1687 | uncharacterized RNA methyltransferase pc1998, putative, expressed | 170 | 6.8048457 | 277 | 14.927774 | -1.133365 | 1.09E-15 | 1.90E-16 | down |
| VIT_14s0066g00140.t01 | 1159 | RNA recognition motif containing protein, putative, expressed | 2254 | 131.32727 | 3671 | 287.95944 | -1.132699 | 3.17E-195 | 5.37E-197 | down |
| VIT_09s0002g00210.t01 | 2201 | endonuclease, putative, expressed | 342 | 10.492779 | 557 | 23.007293 | -1.132694 | 1.96E-30 | 1.98E-31 | down |
| VIT_15s0048g00910.t01 | 877 | Putative uncharacterized protein | 35 | 2.6949635 | 57 | 5.9088863 | -1.13262 | 0.00042598 | 0.00019106 | down |
| VIT_00s0246g00180.t01 | 876 | NADPH-dependent oxidoreductase, putative, expressed | 510 | 39.314297 | 830 | 86.139899 | -1.131628 | 8.68E-45 | 6.33E-46 | down |
| VIT_03s0038g01320.t01 | 3538 | PPR repeat domain containing protein, putative, expressed | 654 | 12.48258 | 1064 | 27.340986 | -1.131149 | 4.33E-57 | 2.49E-58 | down |
| VIT_13s0019g04930.t01 | 938 | 50S ribosomal protein L15, putative, expressed | 99 | 7.1271648 | 161 | 15.604628 | -1.130574 | 1.50E-09 | 3.83E-10 | down |
| VIT_18s0001g12490.t01 | 1238 | S-adenosyl-L-methionine-dependent methyltransferase Mvan_1345-like | 123 | 6.7091718 | 200 | 14.687224 | -1.130355 | 1.38E-11 | 3.02E-12 | down |
| VIT_07s0005g00950.t01 | 1291 | mitochondrial carrier protein, putative, expressed | 248 | 12.972088 | 403 | 28.37979 | -1.129453 | 3.25E-22 | 4.28E-23 | down |
| VIT_13s0047g00100.t01 | 510 | Putative uncharacterized protein | 80 | 10.592641 | 130 | 23.174136 | -1.129453 | 6.55E-08 | 1.90E-08 | down |
| VIT_02s0025g04710.t01 | 1550 | PGR5-like protein 1A | 1709 | 74.455161 | 2777 | 162.88255 | -1.129388 | 3.59E-147 | 8.05E-149 | down |
| VIT_06s0004g01880.t01 | 2353 | outer envelope protein of 80 kDa | 269 | 7.7199555 | 437 | 16.884565 | -1.129041 | 5.54E-24 | 6.81E-25 | down |
| VIT_17s0000g06350.t01 | 932 | LHCA4; light-harvesting complex I chlorophyll a/b binding protein 4 | 19528 | 1414.9018 | 31722 | 3094.3898 | -1.128953 | 0 | 0 | down |
| VIT_04s0008g02640.t01 | 843 | PAP fibrillin family domain containing protein, expressed | 420 | 33.643886 | 682 | 73.550761 | -1.128396 | 8.10E-37 | 7.03E-38 | down |
| VIT_19s0090g01770.t01 | 2360 | mTERF family protein, expressed | 146 | 4.177585 | 237 | 9.1299146 | -1.127932 | 1.76E-13 | 3.41E-14 | down |

| | | | | | | | | | | |
|---|---|---|---|---|---|---|---|---|---|---|
| VIT_16s0050g00780.t01 | 1840 | Citrate transporter protein, putative, expressed | 212 | 7.78041 | 344 | 16.99695 | -1.127358 | 4.58E-19 | 6.82E-20 | down |
| VIT_13s0156g00320.t01 | 1087 | zinc finger C-x8-C-x5-C-x3-H type family protein, expressed | 246 | 15.282345 | 399 | 33.371346 | -1.126744 | 6.37E-22 | 8.45E-23 | down |
| VIT_08s0007g01920.t01 | 1691 | MYB family transcription factor, putative, expressed | 103 | 4.1131833 | 167 | 8.9784886 | -1.126217 | 8.26E-10 | 2.06E-10 | down |
| VIT_15s0048g02500.t01 | 824 | curvature thylakoid 1 | 5040 | 413.03587 | 8170 | 901.41591 | -1.125926 | 0 | 0 | down |
| VIT_00s0475g00010.t01 | 1433 | glycosyl transferase 8 domain containing protein, putative, expressed | 145 | 6.8329187 | 235 | 14.909121 | -1.125621 | 2.46E-13 | 4.80E-14 | down |
| VIT_18s0122g01320.t01 | 1614 | Catalase | 3464 | 144.93017 | 5613 | 316.17089 | -1.125347 | 4.55E-295 | 5.15E-297 | down |
| VIT_00s1674g00010.t01 | 523 | 4-nitrophenylphosphatase, putative, expressed | 831 | 107.29606 | 1346 | 233.97731 | -1.124772 | 2.50E-71 | 1.18E-72 | down |
| VIT_15s0046g03350.t01 | 1028 | cleavage and polyadenylation specificity factor subunit 5-like | 142 | 9.3278096 | 230 | 20.340663 | -1.124756 | 4.64E-13 | 9.20E-14 | down |
| VIT_07s0141g00270.t01 | 930 | auxin-induced protein 22D | 276 | 20.040593 | 447 | 43.697335 | -1.12462 | 2.33E-24 | 2.82E-25 | down |
| VIT_17s0000g04300.t01 | 806 | RNA-binding protein pno1-like | 84 | 7.0376666 | 136 | 15.340314 | -1.124159 | 3.59E-08 | 1.02E-08 | down |
| VIT_01s0011g01230.t01 | 1707 | GTP-binding protein ERG-like | 84 | 3.322999 | 136 | 7.2432881 | -1.124159 | 3.59E-08 | 1.02E-08 | down |
| VIT_14s0060g02210.t01 | 2629 | translation initiation factor IF-2-like | 152 | 3.9042484 | 246 | 8.5069698 | -1.1236 | 7.12E-14 | 1.36E-14 | down |
| VIT_17s0053g00980.t01 | 542 | mitochondrial import inner membrane translocase subunit TIM14-like | 34 | 4.2360792 | 55 | 9.225582 | -1.12291 | 0.00059286 | 0.00027347 | down |
| VIT_03s0038g02280.t01 | 1582 | senescence-associated protein, putative, expressed | 489 | 20.873094 | 791 | 45.456959 | -1.122857 | 3.29E-42 | 2.52E-43 | down |
| VIT_19s0014g00400.t01 | 1091 | ribosomal protein L13, putative, expressed | 1022 | 63.257291 | 1653 | 137.74583 | -1.122705 | 3.97E-87 | 1.53E-88 | down |
| VIT_00s0211g00160.t01 | 757 | serine hydroxymethyltransferase, mitochondrial precursor, putative, expressed | 347 | 30.95409 | 561 | 67.374779 | -1.122079 | 3.35E-30 | 3.42E-31 | down |
| VIT_02s0154g00400.t01 | 1891 | scarecrow transcription factor family protein, putative, expressed | 224 | 7.9990964 | 362 | 17.403933 | -1.121504 | 7.54E-20 | 1.09E-20 | down |
| VIT_17s0000g10260.t01 | 1979 | uncharacterized aarF domain-containing protein kinase 1-like | 263 | 8.9741722 | 425 | 19.524212 | -1.121414 | 4.10E-23 | 5.22E-24 | down |
| VIT_10s0071g01150.t01 | 3063 | katanin p80 WD40 repeat-containing subunit B1 homolog 1, putative, expressed | 289 | 6.3714061 | 467 | 13.861182 | -1.121367 | 2.73E-25 | 3.22E-26 | down |
| VIT_05s0020g00740.t01 | 1725 | domain of unknown function, DUF250 domain containing protein, expressed | 182 | 7.1247025 | 294 | 15.494894 | -1.120891 | 2.62E-16 | 4.40E-17 | down |
| VIT_02s0012g02670.t01 | 839 | Putative uncharacterized protein | 161 | 12.95831 | 260 | 28.173562 | -1.120464 | 1.53E-14 | 2.82E-15 | down |
| VIT_04s0008g06700.t01 | 953 | Putative uncharacterized protein | 109 | 7.7235692 | 176 | 16.789978 | -1.120261 | 3.37E-10 | 8.16E-11 | down |
| VIT_06s0004g03310.t01 | 1829 | hydrolase, alpha/beta fold family protein, putative, expressed | 384 | 14.177575 | 620 | 30.818277 | -1.120175 | 3.51E-33 | 3.31E-34 | down |
| VIT_09s0002g01520.t01 | 1364 | Putative uncharacterized protein | 57 | 2.8219215 | 92 | 6.1320238 | -1.119685 | 7.43E-06 | 2.65E-06 | down |
| VIT_15s0048g01740.t01 | 1804 | growth-regulating factor 9 | 93 | 3.4812151 | 150 | 7.5593612 | -1.118673 | 7.72E-09 | 2.08E-09 | down |
| VIT_05s0094g00460.t01 | 1981 | eukaryotic translation initiation factor 2A, putative, expressed | 155 | 5.2836211 | 250 | 11.473235 | -1.118673 | 5.45E-14 | 1.03E-14 | down |
| VIT_09s0002g01490.t01 | 1639 | GTP-binding protein, putative, expressed | 142 | 5.8505115 | 229 | 12.702433 | -1.11847 | 6.70E-13 | 1.34E-13 | down |
| VIT_15s0048g02820.t01 | 1545 | transcription factor MYC7E, putative, expressed | 129 | 5.6382674 | 208 | 12.239544 | -1.118226 | 8.09E-12 | 1.75E-12 | down |
| VIT_00s1365g00010.t01 | 1237 | fimbrin-like protein 2, putative, expressed | 67 | 3.6575439 | 108 | 7.9375126 | -1.117812 | 1.15E-06 | 3.76E-07 | down |
| VIT_00s0510g00020.t01 | 1144 | methyltransferase-like protein 2 | 67 | 3.9548792 | 108 | 8.5827824 | -1.117812 | 1.15E-06 | 3.76E-07 | down |
| VIT_13s0064g00900.t01 | 892 | Putative uncharacterized protein | 95 | 7.1918926 | 153 | 15.593979 | -1.116546 | 5.70E-09 | 1.52E-09 | down |
| VIT_00s0875g00010.t01 | 705 | phosphoglycerate mutase, putative, expressed | 82 | 7.8543306 | 132 | 17.02218 | -1.115856 | 7.00E-08 | 2.03E-08 | down |
| VIT_11s0052g00510.t01 | 1892 | nucleolar protein,Nop52 containing protein, expressed | 210 | 7.4951893 | 338 | 16.241493 | -1.115647 | 1.86E-18 | 2.84E-19 | down |
| VIT_18s0072g00970.t01 | 1455 | protease Do-like 1 | 720 | 33.41596 | 1158 | 72.356231 | -1.11458 | 1.31E-60 | 7.05E-62 | down |
| VIT_19s0090g00530.t01 | 1173 | heterodimeric geranylgeranyl pyrophosphate synthase small subunit | 74 | 4.2600839 | 119 | 9.2231511 | -1.114378 | 3.35E-07 | 1.04E-07 | down |
| VIT_11s0016g02600.t01 | 772 | caffeoyl-CoA O-methyltransferase | 51 | 4.4610523 | 82 | 9.6566596 | -1.11414 | 2.64E-05 | 1.01E-05 | down |
| VIT_17s0000g08580.t01 | 1378 | Putative uncharacterized protein | 107 | 5.2434726 | 172 | 11.347746 | -1.113811 | 6.53E-10 | 1.62E-10 | down |
| VIT_16s0022g02170.t01 | 3569 | alpha-L-fucosidase 1-like | 219 | 4.1436399 | 352 | 8.966573 | -1.113658 | 4.00E-19 | 5.94E-20 | down |
| VIT_08s0058g00510.t01 | 1915 | MATE efflux protein | 257 | 9.062516 | 413 | 19.607022 | -1.113387 | 3.03E-22 | 3.99E-23 | down |
| VIT_14s0171g00220.t01 | 1115 | ribosome recycling factor, putative, expressed | 94 | 5.6929508 | 151 | 12.312109 | -1.112829 | 7.97E-09 | 2.15E-09 | down |
| VIT_14s0108g00750.t01 | 1068 | ZF-HD protein dimerisation region containing protein, expressed | 533 | 33.700815 | 856 | 72.867335 | -1.112489 | 6.19E-45 | 4.49E-46 | down |
| VIT_17s0000g05830.t01 | 1394 | Putative uncharacterized protein | 137 | 6.636548 | 220 | 14.347964 | -1.112341 | 2.45E-12 | 5.08E-13 | down |
| VIT_00s0174g00190.t01 | 1051 | ribosomal L18p/L5e family protein, putative, expressed | 733 | 47.096182 | 1177 | 101.81321 | -1.112243 | 2.19E-61 | 1.17E-62 | down |
| VIT_12s0057g01270.t01 | 2231 | acyl-CoA synthetase protein, putative, expressed | 436 | 13.196883 | 700 | 28.525209 | -1.11204 | 6.59E-37 | 5.70E-38 | down |
| VIT_00s0246g00220.t01 | 1318 | ATPF0B, atpF; F-type H+-transporting ATPase subunit b | 474 | 24.285518 | 761 | 52.492786 | -1.112023 | 4.92E-40 | 3.97E-41 | down |
| VIT_04s0023g02690.t01 | 3250 | beta-galactosidase, putative, expressed | 1269 | 26.367121 | 2037 | 56.982046 | -1.111767 | 1.70E-105 | 5.42E-107 | down |
| VIT_08s0007g04400.t01 | 2009 | EAD-box ATP-dependent RNA helicase 38-like | 200 | 6.7225571 | 321 | 14.526315 | -1.111587 | 1.74E-17 | 2.77E-18 | down |
| VIT_08s0007g03240.t01 | 1161 | alpha carbonic anhydrase 1 | 501 | 29.140027 | 804 | 62.958475 | -1.111398 | 3.37E-42 | 2.57E-43 | down |
| VIT_03s0063g02090.t01 | 864 | DUF677 domain containing protein, putative, expressed | 169 | 13.208619 | 271 | 28.515824 | -1.110283 | 6.58E-15 | 1.20E-15 | down |
| VIT_08s0040g00530.t01 | 934 | Mpv17 / PMP22 family domain containing protein, expressed | 68 | 4.9163917 | 109 | 10.609868 | -1.109735 | 1.18E-06 | 3.88E-07 | down |
| VIT_10s0003g04270.t01 | 1011 | BAG domain-containing protein, putative, expressed | 574 | 38.339388 | 920 | 82.730766 | -1.109597 | 5.08E-48 | 3.47E-49 | down |
| VIT_06s0004g02410.t01 | 1998 | NAD kinase, putative, expressed | 78 | 2.6362316 | 125 | 5.6878077 | -1.109396 | 1.83E-07 | 5.54E-08 | down |
| VIT_01s0010g00580.t01 | 1148 | translin, putative, expressed | 78 | 4.5881452 | 125 | 9.8991635 | -1.109396 | 1.83E-07 | 5.54E-08 | down |
| VIT_13s0019g05250.t01 | 1748 | lactate/malate dehydrogenase, putative, expressed | 1547 | 59.763129 | 2478 | 128.8814 | -1.108716 | 1.39E-127 | 3.62E-129 | down |
| VIT_03s0063g01060.t01 | 1425 | MYB family transcription factor, putative, expressed | 263 | 12.463078 | 421 | 26.85948 | -1.107771 | 1.76E-22 | 2.30E-23 | down |
| VIT_01s0011g01800.t01 | 1282 | inositol-1-monophosphatase, putative, expressed | 130 | 6.8476219 | 208 | 14.750464 | -1.107085 | 1.20E-11 | 2.62E-12 | down |
| VIT_08s0007g05630.t01 | 1757 | putative UPF0481 protein | 70 | 2.690362 | 112 | 5.7953095 | -1.107085 | 8.78E-07 | 2.84E-07 | down |
| VIT_19s0014g02920.t01 | 1813 | PPR repeat domain containing protein, putative, expressed | 90 | 3.352194 | 144 | 7.220962 | -1.107085 | 2.10E-08 | 5.83E-09 | down |
| VIT_17s0000g01690.t01 | 1707 | argininosuccinate synthase, chloroplast precursor, putative, expressed | 504 | 19.937994 | 806 | 42.927134 | -1.10637 | 5.32E-42 | 4.09E-43 | down |
| VIT_19s0014g01710.t01 | 1958 | sodium/calcium exchanger protein, putative, expressed | 1716 | 59.181918 | 2743 | 127.36306 | -1.105719 | 1.60E-140 | 3.74E-142 | down |
| VIT_05s0049g01940.t01 | 1936 | nuclear transport factor 2, putative, expressed | 254 | 8.8595733 | 406 | 19.065625 | -1.105665 | 1.19E-21 | 1.59E-22 | down |
| VIT_06s0004g02330.t01 | 2576 | ferredoxin--nitrite reductase, putative, expressed | 1138 | 29.831895 | 1819 | 64.197367 | -1.105658 | 1.80E-93 | 6.45E-95 | down |
| VIT_09s0018g01950.t01 | 2428 | CSLC2 - cellulose synthase-like family C, expressed | 249 | 6.9252445 | 398 | 14.902693 | -1.105636 | 3.03E-21 | 4.12E-22 | down |
| VIT_18s0001g04890.t01 | 2096 | sulfate transporter, putative, expressed | 296 | 9.5364092 | 473 | 20.516356 | -1.105256 | 4.92E-25 | 5.84E-26 | down |
| VIT_05s0094g01620.t01 | 1213 | methionyl-tRNA synthetase, putative, expressed | 393 | 21.878432 | 628 | 47.068376 | -1.105249 | 6.71E-33 | 6.40E-34 | down |
| VIT_04s0044g00530.t01 | 1091 | PAP fibrillin family domain containing protein, expressed | 87 | 5.3849162 | 139 | 11.582983 | -1.105011 | 3.96E-08 | 1.12E-08 | down |
| VIT_14s0030g00710.t01 | 1962 | transporter, monovalent cation:proton antiporter-2 family, putative, expressed | 119 | 4.0957402 | 190 | 8.8041001 | -1.104051 | 1.08E-10 | 2.52E-11 | down |
| VIT_02s0025g04550.t01 | 1939 | HOTHEAD precursor, putative, expressed | 1146 | 39.910875 | 1829 | 85.756346 | -1.103462 | 1.11E-93 | 3.97E-95 | down |
| VIT_12s0034g01210.t01 | 1285 | PAP fibrillin family domain containing protein, expressed | 267 | 14.031128 | 426 | 30.139556 | -1.103027 | 1.39E-22 | 1.80E-23 | down |
| VIT_06s0080g00890.t01 | 1352 | Putative uncharacterized protein | 377 | 18.829947 | 601 | 40.413657 | -1.101814 | 2.25E-31 | 2.22E-32 | down |
| VIT_02s0025g04640.t01 | 2375 | ubiquitin interaction motif family protein, expressed | 165 | 4.691425 | 263 | 10.06752 | -1.10161 | 2.50E-14 | 4.65E-15 | down |
| VIT_15s0046g01640.t01 | 1392 | protein ABIL1 | 118 | 5.7243637 | 188 | 12.278604 | -1.100959 | 1.50E-10 | 3.54E-11 | down |
| VIT_01s0010g03740.t01 | 1260 | mTERF domain containing protein, expressed | 86 | 4.6090599 | 137 | 9.8850847 | -1.100781 | 5.51E-08 | 1.59E-08 | down |
| VIT_11s0052g00660.t01 | 1369 | aldose 1-epimerase, putative, expressed | 297 | 14.649994 | 473 | 31.411456 | -1.100391 | 7.29E-25 | 8.70E-26 | down |
| VIT_06s0009g03430.t01 | 923 | zinc finger, C3HC4 type domain containing protein, expressed | 255 | 18.656189 | 406 | 39.990304 | -1.099996 | 1.76E-21 | 2.37E-22 | down |
| VIT_10s0003g04560.t01 | 5658 | AIR9 protein, putative, expressed | 426 | 5.0842992 | 678 | 10.894245 | -1.099445 | 3.80E-35 | 3.43E-36 | down |
| VIT_00s0227g00060.t01 | 2143 | WD domain, G-beta repeat domain containing protein, expressed | 159 | 5.01025 | 253 | 10.733188 | -1.099124 | 8.87E-14 | 1.70E-14 | down |
| VIT_05s0020g03000.t01 | 1517 | 2-hydroxyisoflavanone dehydratase-like | 88 | 3.9172522 | 140 | 8.3902099 | -1.098865 | 4.07E-08 | 1.16E-08 | down |
| VIT_15s0048g02260.t01 | 1824 | endonuclease/exonuclease/phosphatase family domain containing protein, expressed | 254 | 9.4035822 | 404 | 20.136635 | -1.09854 | 2.45E-21 | 3.32E-22 | down |
| VIT_14s0066g00320.t01 | 1831 | TruB family pseudouridylate synthase, putative, expressed | 205 | 7.5604903 | 326 | 16.186749 | -1.098262 | 2.02E-17 | 3.24E-18 | down |
| VIT_01s0011g04800.t01 | 1210 | nicotinate-nucleotide pyrophosphorylase, putative, expressed | 78 | 4.3530502 | 124 | 9.3167982 | -1.097808 | 2.63E-07 | 8.07E-08 | down |
| VIT_19s0014g01550.t01 | 1478 | DAG protein, chloroplast precursor, putative, expressed | 781 | 35.682974 | 1241 | 76.335705 | -1.097122 | 2.82E-63 | 1.47E-64 | down |
| VIT_03s0038g04280.t01 | 1311 | BCCIP, putative, expressed | 97 | 4.9963573 | 154 | 10.679438 | -1.095887 | 8.74E-09 | 2.36E-09 | down |
| VIT_03s0017g00640.t01 | 1995 | citrate transporter, putative, expressed | 647 | 21.900086 | 1027 | 46.8013 | -1.095612 | 2.02E-52 | 1.26E-53 | down |
| VIT_09s0002g04070.t01 | 1602 | Putative uncharacterized protein | 126 | 5.3111978 | 200 | 11.350052 | -1.09559 | 4.51E-11 | 1.02E-11 | down |
| VIT_18s0001g05620.t01 | 1968 | mitochondrial carrier protein, putative, expressed | 150 | 5.1469578 | 238 | 10.994671 | -1.095013 | 5.97E-13 | 1.19E-13 | down |
| VIT_08s0007g07400.t01 | 1812 | glutamate dehydrogenase protein, putative, expressed | 273 | 10.173934 | 433 | 21.725015 | -1.09448 | 1.16E-22 | 1.50E-23 | down |
| VIT_15s0048g02840.t01 | 2024 | CAMK_CAMK_like.30 - CAMK includes calcium/calmodulin depedent protein kina | 123 | 4.1037325 | 195 | 8.758999 | -1.093829 | 8.48E-11 | 1.96E-11 | down |
| VIT_18s0001g15000.t01 | 1635 | ACT domain containing protein, putative, expressed | 161 | 6.6495547 | 255 | 14.179235 | -1.09245 | 9.40E-14 | 1.80E-14 | down |
| VIT_12s0057g01140.t01 | 2359 | ELMO/CED-12 family protein, putative, expressed | 156 | 4.4656132 | 247 | 9.5191766 | -1.091979 | 2.41E-13 | 4.70E-14 | down |
| VIT_16s0098g00990.t01 | 687 | Putative uncharacterized protein | 48 | 4.7181196 | 76 | 10.057435 | -1.091979 | 7.16E-05 | 2.88E-05 | down |
| VIT_04s0008g03750.t01 | 2084 | eukaryotic translation initiation factor 3 subunit E-interacting protein, putative, expre | 640 | 20.737992 | 1013 | 44.191842 | -1.091504 | 2.06E-51 | 1.31E-52 | down |
| VIT_06s0004g04460.t01 | 1585 | inner membrane protein, putative, expressed | 1867 | 79.542547 | 2955 | 169.49566 | -1.09145 | 3.42E-148 | 7.62E-150 | down |
| VIT_19s0014g03530.t01 | 2005 | cytochrome c-type biogenesis ccda-like chloroplastic protein 2 | 280 | 9.4303562 | 443 | 20.087215 | -1.090893 | 4.80E-23 | 6.13E-24 | down |
| VIT_01s0011g02150.t01 | 1503 | photosystem II stability/assembly factor HCF136 | 894 | 40.166407 | 1414 | 85.530459 | -1.090449 | 2.84E-71 | 1.34E-72 | down |
| VIT_14s0068g01010.t01 | 1998 | Putative uncharacterized protein | 105 | 3.5487733 | 166 | 7.5534086 | -1.089807 | 2.61E-09 | 6.77E-10 | down |
| VIT_00s0391g00020.t01 | 1268 | protein CbbY-like | 515 | 27.426628 | 814 | 58.36272 | -1.08947 | 2.18E-41 | 1.70E-42 | down |

| | | | | | | | | | | |
|---|---|---|---|---|---|---|---|---|---|---|
| VIT_04s0008g03280.t01 | 639 | Putative uncharacterized protein | 81 | 8.5598983 | 128 | 18.211239 | -1.089163 | 2.01E-07 | 6.08E-08 | down |
| VIT_16s0022g01440.t01 | 2524 | transketolase, chloroplast precursor, putative, expressed | 11018 | 294.7799 | 17411 | 627.14034 | -1.08915 | 0 | 0 | down |
| VIT_06s0009g01610.t01 | 1438 | Putative uncharacterized protein | 57 | 2.6767044 | 90 | 5.6900227 | -1.087977 | 1.53E-05 | 5.68E-06 | down |
| VIT_04s0023g00350.t01 | 1140 | lysine ketoglutarate reductase trans-splicing related 1, putative, expressed | 57 | 3.3764043 | 90 | 7.1774145 | -1.087977 | 1.53E-05 | 5.68E-06 | down |
| VIT_17s0000g02840.t01 | 1065 | BCAS2 protein, putative, expressed | 152 | 9.6378114 | 240 | 20.487643 | -1.087977 | 6.31E-13 | 1.26E-13 | down |
| VIT_13s0073g00530.t01 | 618 | periplasmic beta-glucosidase precursor, putative, expressed | 38 | 4.1522124 | 60 | 8.8265939 | -1.087977 | 0.00046863 | 0.00021126 | down |
| VIT_18s0001g04150.t01 | 1856 | galactosyltransferase, putative, expressed | 140 | 5.0937134 | 221 | 10.825418 | -1.087633 | 5.49E-12 | 1.17E-12 | down |
| VIT_02s0025g00280.t01 | 2120 | heat shock protein 83 | 147 | 4.682372 | 232 | 9.9490702 | -1.087322 | 1.60E-12 | 3.28E-13 | down |
| VIT_05s0094g01460.t01 | 1203 | Putative uncharacterized protein | 45 | 2.5259883 | 71 | 5.3656593 | -1.086908 | 0.00013472 | 5.64E-05 | down |
| VIT_01s0026g02280.t01 | 1296 | protein HGH1 homolog | 97 | 5.0541855 | 153 | 10.732893 | -1.086488 | 1.25E-08 | 3.42E-09 | down |
| VIT_07s0130g00470.t01 | 1382 | FYVE zinc finger family protein, expressed | 123 | 6.0100974 | 194 | 12.762156 | -1.086412 | 1.21E-10 | 2.84E-11 | down |
| VIT_06s0009g00310.t01 | 1990 | protein FAF-like | 156 | 5.293659 | 246 | 11.238605 | -1.086126 | 3.44E-13 | 6.78E-14 | down |
| VIT_06s0004g05230.t01 | 2110 | serine/arginine repetitive matrix protein 1 | 4840 | 154.89855 | 7632 | 328.84124 | -1.086068 | 0 | 0 | down |
| VIT_15s0048g01900.t01 | 573 | tRNA-Lys (trnK) | 281 | 33.115868 | 443 | 70.287723 | -1.08575 | 7.07E-23 | 9.08E-24 | down |
| VIT_04s0008g05780.t01 | 2372 | WD domain, G-beta repeat domain containing protein, expressed | 118 | 3.359323 | 186 | 7.1290003 | -1.085529 | 3.08E-10 | 7.43E-11 | down |
| VIT_12s0059g01920.t01 | 2755 | STRUBBELIG-RECEPTOR FAMILY 1 precursor, putative, expressed | 177 | 4.3384651 | 279 | 9.2068904 | -1.085529 | 8.33E-15 | 1.52E-15 | down |
| VIT_18s0001g05630.t01 | 2621 | KH domain-containing protein, putative, expressed | 719 | 18.524492 | 1133 | 39.300064 | -1.085098 | 7.56E-57 | 4.37E-58 | down |
| VIT_18s0001g00440.t01 | 1461 | ATP-binding domain-containing protein, putative, expressed | 139 | 6.4246434 | 219 | 13.627754 | -1.084859 | 7.61E-12 | 1.64E-12 | down |
| VIT_07s0031g01760.t01 | 1012 | chloroplast lumen common family protein, putative, expressed | 127 | 8.4743745 | 200 | 17.967177 | -1.084185 | 6.63E-11 | 1.52E-11 | down |
| VIT_19s0014g02470.t01 | 1636 | Putative uncharacterized protein | 237 | 9.7824917 | 373 | 20.727929 | -1.083302 | 2.30E-19 | 3.39E-20 | down |
| VIT_18s0001g10130.t01 | 2097 | ZOS6-01 - C2H2 zinc finger protein, expressed | 197 | 6.3438403 | 310 | 13.439826 | -1.083086 | 2.86E-16 | 4.81E-17 | down |
| VIT_12s0059g01670.t01 | 1288 | nuclear ribonuclease Z, putative, expressed | 333 | 17.458737 | 524 | 36.986718 | -1.083058 | 9.23E-27 | 1.04E-27 | down |
| VIT_08s0007g01790.t01 | 3006 | FG-GAP repeat-containing protein, putative, expressed | 204 | 4.5827444 | 321 | 9.7083724 | -1.083018 | 8.29E-17 | 1.36E-17 | down |
| VIT_04s0008g06470.t01 | 1673 | cysteinyl-tRNA synthetase, putative, expressed | 75 | 3.0272603 | 118 | 6.4123385 | -1.082838 | 7.09E-07 | 2.27E-07 | down |
| VIT_15s0046g01410.t01 | 994 | harpin-induced protein 1 domain containing protein, expressed | 82 | 5.5707275 | 129 | 11.798687 | -1.082689 | 2.06E-07 | 6.25E-08 | down |
| VIT_06s0009g00690.t01 | 3401 | protein kinase domain containing protein, expressed | 288 | 5.7183443 | 453 | 12.109381 | -1.082456 | 2.94E-23 | 3.71E-24 | down |
| VIT_08s0032g00630.t01 | 557 | chloroplast DNA | 220 | 26.671776 | 346 | 56.474355 | -1.082282 | 5.14E-18 | 8.02E-19 | down |
| VIT_16s0039g02720.t01 | 1429 | glutamate dehydrogenase protein, putative, expressed | 56 | 2.6463071 | 88 | 5.5986177 | -1.08109 | 2.13E-05 | 8.04E-06 | down |
| VIT_07s0005g05860.t01 | 2220 | GTPase of unknown function domain containing protein, putative, expressed | 196 | 5.9619392 | 308 | 12.613282 | -1.08109 | 3.97E-16 | 6.73E-17 | down |
| VIT_04s0008g00180.t01 | 1785 | NADP-dependent malic enzyme, chloroplast precursor, putative, expressed | 231 | 8.7389288 | 363 | 18.488377 | -1.08109 | 8.16E-19 | 1.23E-19 | down |
| VIT_06s0080g00570.t01 | 1543 | suppressor protein SRP40 | 77 | 3.3698397 | 121 | 7.129348 | -1.08109 | 5.24E-07 | 1.65E-07 | down |
| VIT_02s0025g01220.t01 | 1833 | HMG1/2, putative, expressed | 121 | 4.4576642 | 190 | 9.4237012 | -1.080006 | 2.34E-10 | 5.60E-11 | down |
| VIT_12s0028g02130.t01 | 2496 | protein kinase domain containing protein, expressed | 228 | 6.168431 | 358 | 13.039737 | -1.079939 | 1.53E-18 | 2.34E-19 | down |
| VIT_12s0028g00300.t01 | 2606 | outer envelope protein 64, | 93 | 2.4098665 | 146 | 5.0934121 | -1.079679 | 3.28E-08 | 9.25E-09 | down |
| VIT_14s0006g00910.t01 | 2010 | ATA15 protein, putative, expressed | 179 | 6.0136953 | 281 | 12.709856 | -1.079624 | 8.79E-15 | 1.61E-15 | down |
| VIT_00s0218g00090.t01 | 1255 | exostosin family domain containing protein, expressed | 130 | 6.9949412 | 204 | 14.778039 | -1.079071 | 5.03E-11 | 1.15E-11 | down |
| VIT_16s0050g00810.t01 | 1690 | F-box protein SKIP23-like | 109 | 4.3553618 | 171 | 9.1989822 | -1.078682 | 2.04E-09 | 5.24E-10 | down |
| VIT_08s0105g00470.t01 | 919 | PHF5-like protein domain containing protein, expressed | 51 | 3.7474781 | 80 | 7.9141604 | -1.078516 | 5.44E-05 | 2.16E-05 | down |
| VIT_12s0134g00050.t01 | 399 | DNA-directed RNA polymerase subunit beta, putative, expressed | 44 | 7.4467063 | 69 | 15.721956 | -1.078106 | 0.00018828 | 8.01E-05 | down |
| VIT_04s0008g04240.t01 | 694 | 2Fe-2S iron-sulfur cluster binding domain containing protein, expressed | 213 | 20.725479 | 334 | 43.75396 | -1.078008 | 2.53E-17 | 4.07E-18 | down |
| VIT_01s0011g04180.t01 | 1187 | lipid phosphatase protein, putative, expressed | 125 | 7.1112138 | 196 | 15.011902 | -1.077939 | 1.28E-10 | 3.00E-11 | down |
| VIT_05s0094g01130.t01 | 1029 | NADP-dependent oxidoreductase, putative, expressed | 118 | 7.7437456 | 185 | 16.345068 | -1.077752 | 4.39E-10 | 1.07E-10 | down |
| VIT_14s0030g01000.t01 | 568 | probable disease resistance protein | 37 | 4.3988366 | 58 | 9.2834634 | -1.077541 | 0.00064929 | 0.00030097 | down |
| VIT_00s0207g00070.t01 | 9261 | phosphoglycolate phosphatase-like | 689 | 5.0239555 | 1080 | 10.602206 | -1.077469 | 1.26E-53 | 7.67E-55 | down |
| VIT_07s0104g01410.t01 | 1281 | OTU domain-containing protein 6B-like | 113 | 5.9568101 | 177 | 12.561876 | -1.07644 | 1.11E-09 | 2.81E-10 | down |
| VIT_11s0037g01100.t01 | 1890 | GPI mannosyltransferase 3-like | 92 | 3.2870815 | 144 | 6.9267747 | -1.075377 | 4.55E-08 | 1.30E-08 | down |
| VIT_06s0004g07730.t01 | 3252 | protein TIC110 | 551 | 11.441567 | 862 | 24.098339 | -1.074649 | 7.88E-43 | 5.98E-44 | down |
| VIT_08s0056g00010.t01 | 897 | seryl-tRNA synthetase, putative, expressed | 241 | 18.142998 | 377 | 38.210197 | -1.074545 | 2.56E-19 | 3.77E-20 | down |
| VIT_04s0023g02150.t01 | 996 | phosphatase, putative, expressed | 165 | 11.186882 | 258 | 23.549991 | -1.073919 | 1.50E-13 | 2.91E-14 | down |
| VIT_17s0000g06420.t01 | 2572 | heat shock protein DnaJ | 142 | 3.7282225 | 222 | 7.8471577 | -1.073682 | 8.23E-12 | 1.78E-12 | down |
| VIT_00s0471g00010.t01 | 1960 | U-box domain containing protein, expressed | 135 | 4.6511692 | 211 | 9.7871615 | -1.073297 | 2.83E-11 | 6.33E-12 | down |
| VIT_07s0005g02540.t01 | 1842 | protoporphyrinogen oxidase | 2100 | 76.986418 | 3282 | 161.98669 | -1.073199 | 4.37E-160 | 9.09E-162 | down |
| VIT_05s0029g00480.t01 | 1833 | transferase family protein, putative, expressed | 183 | 6.7417566 | 286 | 14.18515 | -1.073185 | 6.84E-15 | 1.25E-15 | down |
| VIT_01s0011g04000.t01 | 2114 | Putative uncharacterized protein | 247 | 7.8899893 | 386 | 16.600176 | -1.073103 | 1.03E-19 | 1.50E-20 | down |
| VIT_04s0008g05940.t01 | 1346 | mitochondrial glycoprotein, putative, expressed | 48 | 2.4081338 | 75 | 5.0657829 | -1.07287 | 0.00010243 | 4.21E-05 | down |
| VIT_16s0022g02110.t01 | 2434 | DEAD-box ATP-dependent RNA helicase 7-like | 2086 | 57.87329 | 3258 | 121.69168 | -1.072261 | 1.07E-158 | 2.25E-160 | down |
| VIT_12s0057g00390.t01 | 1355 | probable threonine--tRNA ligase | 73 | 3.6380445 | 114 | 7.6488462 | -1.072079 | 1.36E-06 | 4.50E-07 | down |
| VIT_12s0059g01150.t01 | 1799 | FAD dependent oxidoreductase, putative, expressed | 146 | 5.4803227 | 228 | 11.522164 | -1.072079 | 4.52E-12 | 9.55E-13 | down |
| VIT_06s0009g02540.t01 | 520 | Putative uncharacterized protein | 66 | 8.5708725 | 103 | 18.007949 | -1.07112 | 4.69E-06 | 1.64E-06 | down |
| VIT_18s0001g08860.t01 | 2689 | cirhin-like | 157 | 3.942696 | 245 | 8.2833432 | -1.071031 | 7.21E-13 | 1.44E-13 | down |
| VIT_19s0015g01110.t01 | 1152 | SFT2, putative, expressed | 125 | 7.3272663 | 195 | 15.389075 | -1.07056 | 1.82E-10 | 4.32E-11 | down |
| VIT_15s0021g01910.t01 | 1343 | AATF, putative, expressed | 50 | 2.5140762 | 78 | 5.2801829 | -1.07056 | 7.55E-05 | 3.05E-05 | down |
| VIT_09s0002g01440.t01 | 717 | EARLY flowering protein, putative, expressed | 109 | 10.265776 | 170 | 21.555601 | -1.07022 | 2.90E-09 | 7.56E-10 | down |
| VIT_05s0020g01750.t01 | 320 | glutaredoxin | 240 | 50.646065 | 374 | 106.25564 | -1.069017 | 5.07E-19 | 7.56E-20 | down |
| VIT_09s0002g08960.t01 | 1120 | deSI-like protein | 43 | 2.5925962 | 67 | 5.4386004 | -1.068838 | 0.00026071 | 0.00011375 | down |
| VIT_06s0009g02670.t01 | 601 | methyltransferase-like protein 22 | 52 | 5.8426963 | 81 | 12.252957 | -1.068424 | 5.59E-05 | 2.22E-05 | down |
| VIT_16s0050g01070.t01 | 1390 | septum site-determining protein minD, putative, expressed | 183 | 8.8903884 | 285 | 18.640623 | -1.068132 | 9.76E-15 | 1.79E-15 | down |
| VIT_11s0016g00130.t01 | 1403 | omega-6 fatty acid desaturase, chloroplast precursor, putative, expressed | 576 | 27.723576 | 897 | 58.125292 | -1.068053 | 4.28E-44 | 3.17E-45 | down |
| VIT_06s0009g02680.t01 | 1057 | Putative uncharacterized protein | 79 | 5.0470377 | 123 | 10.579387 | -1.067747 | 5.51E-07 | 1.75E-07 | down |
| VIT_19s0014g00030.t01 | 876 | frigida, putative, expressed | 79 | 6.0898617 | 123 | 12.76531 | -1.067747 | 5.51E-07 | 1.75E-07 | down |
| VIT_04s0023g02120.t01 | 1745 | GTP-binding protein, putative, expressed | 196 | 7.5848166 | 305 | 15.890398 | -1.066969 | 1.15E-15 | 2.00E-16 | down |
| VIT_13s0019g04190.t01 | 1679 | chorismate synthase | 304 | 12.226646 | 473 | 25.611842 | -1.066782 | 1.08E-23 | 1.34E-24 | down |
| VIT_18s0117g00190.t01 | 2270 | DUF246 domain-containing protein | 117 | 3.4805225 | 182 | 7.2891335 | -1.066443 | 8.65E-10 | 2.17E-10 | down |
| VIT_08s0007g00070.t01 | 989 | RNA recognition motif containing protein, putative, expressed | 72 | 4.9160993 | 112 | 10.29561 | -1.066443 | 1.89E-06 | 6.34E-07 | down |
| VIT_02s0012g03140.t01 | 1116 | chloroplast DNA | 287 | 17.366094 | 446 | 36.332981 | -1.065006 | 2.40E-22 | 3.14E-23 | down |
| VIT_11s0016g02080.t01 | 1265 | esterase/lipase/thioesterase, putative, expressed | 334 | 17.82955 | 519 | 37.29986 | -1.0649 | 7.99E-26 | 9.24E-27 | down |
| VIT_02s0025g00390.t01 | 557 | expressed protein | 65 | 7.8802973 | 101 | 16.485288 | -1.064857 | 6.50E-06 | 2.31E-06 | down |
| VIT_15s0048g00390.t01 | 1948 | O-sialoglycoprotein endopeptidase, putative, expressed | 121 | 4.1945064 | 188 | 8.7740331 | -1.064739 | 4.74E-10 | 1.16E-10 | down |
| VIT_07s0104g00060.t01 | 2662 | helicase, putative, expressed | 112 | 2.8411516 | 174 | 5.9425326 | -1.064602 | 2.20E-09 | 5.68E-10 | down |
| VIT_04s0044g02040.t01 | 1345 | ubiquitin carboxyl-terminal hydrolase, family 1, putative, expressed | 168 | 8.434735 | 261 | 17.642032 | -1.064602 | 1.62E-13 | 3.14E-14 | down |
| VIT_06s0009g03390.t01 | 617 | Putative uncharacterized protein | 197 | 21.560831 | 306 | 45.088588 | -1.06435 | 1.18E-15 | 2.05E-16 | down |
| VIT_05s0020g03160.t01 | 1798 | transmembrane protein 49, putative, expressed | 358 | 13.445526 | 556 | 28.113536 | -1.064139 | 1.47E-27 | 1.61E-28 | down |
| VIT_00s1203g00010.t01 | 696 | auxin response factor 6-like | 38 | 3.6868783 | 59 | 7.7067832 | -1.063729 | 0.00066617 | 0.0003093 | down |
| VIT_05s0051g00830.t01 | 2277 | dihydroxy-acid dehydratase, putative, expressed | 449 | 13.315815 | 697 | 27.829161 | -1.063457 | 2.97E-34 | 2.73E-35 | down |
| VIT_17s0053g00440.t01 | 324 | Putative uncharacterized protein | 134 | 27.928283 | 208 | 58.36449 | -1.063364 | 5.57E-11 | 1.28E-11 | down |
| VIT_14s0030g02230.t01 | 1707 | BHLH transcription factor, putative, expressed | 192 | 7.5954262 | 298 | 15.871322 | -1.06322 | 2.98E-15 | 5.31E-16 | down |
| VIT_12s0035g01080.t01 | 1922 | amine oxidase, flavin-containing, domain containing protein, expressed | 221 | 7.7646759 | 343 | 16.224492 | -1.063176 | 2.18E-17 | 3.49E-18 | down |
| VIT_18s0072g00780.t01 | 2277 | ubiquitin interaction motif-containing protein, putative, expressed | 366 | 10.854317 | 568 | 22.678571 | -1.063061 | 4.38E-28 | 4.71E-29 | down |
| VIT_10s0003g04720.t01 | 1655 | G-patch domain containing protein, expressed | 147 | 5.997963 | 228 | 12.524697 | -1.062231 | 6.57E-12 | 1.41E-12 | down |
| VIT_16s0098g00540.t01 | 3160 | tRNA-binding arm, putative, expressed | 147 | 3.1413382 | 228 | 6.5596118 | -1.062231 | 6.57E-12 | 1.41E-12 | down |
| VIT_18s0072g00950.t01 | 1601 | probable magnesium transporter NIPA4 | 69 | 2.9103298 | 107 | 6.0760707 | -1.061956 | 3.56E-06 | 1.23E-06 | down |
| VIT_02s0012g01730.t01 | 747 | Putative uncharacterized protein | 69 | 6.2375341 | 107 | 13.022475 | -1.061956 | 3.56E-06 | 1.23E-06 | down |
| VIT_02s0012g01580.t01 | 827 | Putative uncharacterized protein | 89 | 7.2672306 | 138 | 15.170642 | -1.061805 | 1.22E-07 | 3.61E-08 | down |
| VIT_05s0062g00640.t01 | 1691 | UDP-glucoronosyl and UDP-glucosyl transferase domain containing protein, express | 287 | 11.461006 | 445 | 23.924715 | -1.061768 | 3.41E-22 | 4.49E-23 | down |
| VIT_02s0241g00110.t01 | 1627 | aminomethyltransferase, putative, expressed | 109 | 4.5240083 | 169 | 9.4434248 | -1.061709 | 4.13E-09 | 1.09E-09 | down |
| VIT_02s0012g01800.t01 | 1193 | shikimate kinase | 149 | 8.4339354 | 231 | 17.603617 | -1.061594 | 4.87E-12 | 1.03E-12 | down |
| VIT_18s0001g13560.t01 | 1992 | LTV1, putative, expressed | 80 | 2.7119713 | 124 | 5.6593001 | -1.061282 | 5.65E-07 | 1.79E-07 | down |

| | | | | | | | | | | |
|---|---|---|---|---|---|---|---|---|---|---|
| VIT_04s0008g01290.t01 | 1135 | putative GATA transcription factor 22-like | 260 | 15.468989 | 403 | 32.280448 | -1.061282 | 3.47E-20 | 4.94E-21 | down |
| VIT_10s0116g00940.t01 | 2624 | early-responsive to dehydration protein-related, putative, expressed | 264 | 6.7939843 | 409 | 14.170653 | -1.060576 | 1.89E-20 | 2.67E-21 | down |
| VIT_17s0000g02190.t01 | 1350 | SURF1, putative, expressed | 51 | 2.551061 | 79 | 5.3201478 | -1.060369 | 7.74E-05 | 3.13E-05 | down |
| VIT_10s0116g01560.t01 | 2144 | U3 small nucleolar RNA-associated protein 6 homolog-like | 82 | 2.5826973 | 127 | 5.3852927 | -1.060146 | 4.17E-07 | 1.31E-07 | down |
| VIT_04s0008g03350.t01 | 3635 | Putative uncharacterized protein | 425 | 7.8953058 | 658 | 16.457045 | -1.059638 | 3.28E-32 | 3.17E-33 | down |
| VIT_06s0009g02780.t01 | 1938 | Putative uncharacterized protein | 177 | 6.1674259 | 274 | 12.85367 | -1.05944 | 4.93E-14 | 9.30E-15 | down |
| VIT_18s0001g12780.t01 | 1147 | ornithine carbamoyltransferase, putative, expressed | 323 | 19.016192 | 500 | 39.631176 | -1.059407 | 1.02E-24 | 1.23E-25 | down |
| VIT_01s0011g01460.t01 | 2045 | bifunctional purine biosynthesis protein purH, putative, expressed | 292 | 9.6421522 | 452 | 20.094421 | -1.059368 | 1.90E-22 | 2.49E-23 | down |
| VIT_17s0000g04520.t01 | 1209 | expressed protein | 772 | 43.119671 | 1195 | 89.861151 | -1.059351 | 1.21E-57 | 6.87E-59 | down |
| VIT_11s0016g04130.t01 | 1443 | transcription factor like protein, putative, expressed | 241 | 11.27808 | 373 | 23.500271 | -1.059156 | 1.05E-18 | 1.59E-19 | down |
| VIT_06s0009g01030.t01 | 1682 | MBTB3 - Bric-a-Brac, Tramtrack, Broad Complex BTB domain with Meprin and T | 159 | 6.3834517 | 246 | 13.296566 | -1.058645 | 1.07E-12 | 2.18E-13 | down |
| VIT_00s2582g00010.t01 | 482 | Putative uncharacterized protein | 192 | 26.899155 | 297 | 56.019572 | -1.05837 | 4.22E-15 | 7.60E-16 | down |
| VIT_08s0040g03240.t01 | 2709 | PPR repeat domain containing protein, putative, expressed | 203 | 5.0602442 | 314 | 10.537826 | -1.058298 | 6.73E-16 | 1.16E-16 | down |
| VIT_07s0005g03790.t01 | 1633 | succinyl-CoA ligase beta-chain, mitochondrial precursor, putative, expressed | 408 | 16.871684 | 631 | 35.129628 | -1.058084 | 7.35E-31 | 7.37E-32 | down |
| VIT_17s0000g10280.t01 | 905 | nucleoside diphosphate kinase, putative, expressed | 646 | 48.202369 | 999 | 100.35691 | -1.057964 | 2.97E-48 | 2.01E-49 | down |
| VIT_05s0020g02940.t01 | 1800 | protein Kinase-associated protein phosphatase, putative, expressed | 97 | 3.6390135 | 150 | 7.5761598 | -1.057919 | 3.62E-08 | 1.03E-08 | down |
| VIT_07s0005g00250.t01 | 675 | co-chaperone protein SBA1, putative, expressed | 335 | 33.513939 | 518 | 69.768014 | -1.057804 | 1.65E-25 | 1.93E-26 | down |
| VIT_18s0001g09260.t01 | 2399 | QWRF motif-containing protein 2 | 196 | 5.5170925 | 303 | 11.482667 | -1.057478 | 2.31E-15 | 4.09E-16 | down |
| VIT_14s0030g01740.t01 | 1720 | zeta-carotene desaturase | 559 | 21.946628 | 864 | 45.668387 | -1.057197 | 8.04E-42 | 6.21E-43 | down |
| VIT_01s0011g03440.t01 | 506 | protein NIM1-INTERACTING 2 | 692 | 92.350664 | 1069 | 192.06913 | -1.056431 | 1.91E-51 | 1.22E-52 | down |
| VIT_08s0007g06790.t01 | 943 | transmembrane and coiled-coil domains protein 1-like | 268 | 19.191439 | 414 | 39.913427 | -1.056411 | 1.47E-20 | 2.05E-21 | down |
| VIT_04s0008g01120.t01 | 744 | glutaredoxin | 101 | 9.1671193 | 156 | 19.062596 | -1.056204 | 1.98E-08 | 5.48E-09 | down |
| VIT_13s0019g02850.t01 | 725 | 18.2 kDa class I heat shock protein | 101 | 9.407361 | 156 | 19.562167 | -1.056204 | 1.98E-08 | 5.48E-09 | down |
| VIT_01s0011g05000.t01 | 2383 | DUF246 domain-containing protein | 90 | 2.5503684 | 139 | 5.3029939 | -1.056101 | 1.25E-07 | 3.70E-08 | down |
| VIT_15s0048g01590.t01 | 3035 | cytochrome P450 76C4-like | 239 | 5.3176977 | 369 | 11.053455 | -1.055624 | 2.02E-18 | 3.09E-19 | down |
| VIT_18s0001g01770.t01 | 844 | Putative uncharacterized protein | 206 | 16.481974 | 318 | 34.254296 | -1.055396 | 5.09E-16 | 8.69E-17 | down |
| VIT_01s0137g00300.t01 | 2881 | protein SDA1 homolog | 173 | 4.0549667 | 267 | 8.4255522 | -1.055081 | 1.28E-13 | 2.48E-14 | down |
| VIT_07s0005g01030.t01 | 3520 | cellulose synthase-like protein D5-like | 236 | 4.5274513 | 364 | 9.4013256 | -1.054165 | 3.79E-18 | 5.87E-19 | down |
| VIT_18s0041g00680.t01 | 2223 | transmembrane 9 superfamily member 3-like | 177 | 5.3767302 | 273 | 11.164867 | -1.054165 | 7.00E-14 | 1.33E-14 | down |
| VIT_00s0271g00100.t01 | 642 | Putative uncharacterized protein | 59 | 6.2058522 | 91 | 12.886552 | -1.054165 | 2.30E-05 | 8.70E-06 | down |
| VIT_19s0014g02710.t01 | 2523 | hydrolase, acting on carbon-nitrogen, putative, expressed | 166 | 4.4429894 | 256 | 9.2247178 | -1.053974 | 4.44E-13 | 8.78E-14 | down |
| VIT_11s0016g00350.t01 | 2534 | conserved oligomeric Golgi complex subunit 3-like | 166 | 4.4237026 | 256 | 9.1846736 | -1.053974 | 4.44E-13 | 8.78E-14 | down |
| VIT_01s0010g02310.t01 | 204 | chloroplast 50S ribosomal protein L23, putative | 61 | 20.192222 | 94 | 41.891707 | -1.052865 | 1.70E-05 | 6.32E-06 | down |
| VIT_16s0013g00290.t01 | 1222 | OR, putative, expressed | 124 | 6.8522772 | 191 | 14.209949 | -1.052246 | 5.09E-10 | 1.25E-10 | down |
| VIT_11s0065g00640.t01 | 2506 | cycloartenol Synthase-like | 350 | 9.431297 | 539 | 19.554111 | -1.051944 | 2.85E-26 | 3.27E-27 | down |
| VIT_11s0052g00960.t01 | 576 | syntaxin 6, N-terminal domain containing protein, expressed | 52 | 6.0962856 | 80 | 12.626933 | -1.050502 | 7.93E-05 | 3.21E-05 | down |
| VIT_11s0052g01280.t01 | 1005 | probable xyloglucan endotransglucosylase/hydrolase protein 23-like | 65 | 4.3674882 | 100 | 9.046161 | -1.050502 | 9.24E-06 | 3.34E-06 | down |
| VIT_11s0016g00740.t01 | 2210 | inactive leucine-rich repeat receptor-like protein kinase | 119 | 3.6361277 | 183 | 7.528166 | -1.049896 | 1.29E-09 | 3.26E-10 | down |
| VIT_12s0028g03100.t01 | 1822 | transcription activator GLK1-like | 430 | 15.936925 | 661 | 32.982492 | -1.049327 | 7.42E-32 | 7.24E-33 | down |
| VIT_18s0001g02730.t01 | 2411 | myosin heavy chain-related, putative, expressed | 175 | 4.901458 | 269 | 10.143444 | -1.049265 | 1.34E-13 | 2.59E-14 | down |
| VIT_01s0026g02350.t01 | 1500 | CAAX amino terminal protease family protein, putative, expressed | 270 | 12.155056 | 415 | 25.152851 | -1.049165 | 2.17E-20 | 3.07E-21 | down |
| VIT_05s0062g00830.t01 | 1500 | 30S ribosomal protein S1 homolog-like | 244 | 10.984569 | 375 | 22.728479 | -1.049023 | 1.59E-18 | 2.43E-19 | down |
| VIT_01s0011g04480.t01 | 764 | histone chaperone ASF1B-like | 41 | 3.6238895 | 63 | 7.4968283 | -1.048741 | 0.00050365 | 0.00022897 | down |
| VIT_09s0002g03190.t01 | 2039 | DNA repair protein RadA homolog | 84 | 2.781932 | 129 | 5.7517878 | -1.047923 | 4.37E-07 | 1.37E-07 | down |
| VIT_08s0056g01640.t01 | 1888 | 3-isopropylmalate dehydratase-like | 607 | 21.710566 | 932 | 44.879116 | -1.047647 | 2.19E-44 | 1.61E-45 | down |
| VIT_02s0154g00250.t01 | 1858 | oxysterol-binding protein-related protein 2A-like | 99 | 3.5981058 | 152 | 7.4375218 | -1.047584 | 3.79E-08 | 1.08E-08 | down |
| VIT_04s0023g00290.t01 | 1834 | protein TRIGALACTOSYLDIACYLGLYCEROL 3 | 256 | 9.4259488 | 393 | 19.481554 | -1.047399 | 2.58E-19 | 3.81E-20 | down |
| VIT_01s0011g00410.t01 | 1537 | protein of unknown function DUF1675 domain containing protein, expressed | 86 | 3.7784095 | 132 | 7.8078316 | -1.047143 | 3.23E-07 | 9.99E-08 | down |
| VIT_14s0081g00460.t01 | 2797 | SAC domain containing protein, putative, expressed | 101 | 2.4384472 | 155 | 5.0381327 | -1.046926 | 2.80E-08 | 7.86E-09 | down |
| VIT_00s0561g00030.t01 | 1687 | RNA recognition motif containing protein, expressed | 131 | 5.243734 | 201 | 10.832067 | -1.046642 | 2.10E-10 | 5.00E-11 | down |
| VIT_07s0151g01110.t01 | 1084 | ribosome recycling factor, putative, expressed | 382 | 23.796798 | 586 | 49.147192 | -1.046342 | 3.14E-28 | 3.37E-29 | down |
| VIT_18s0001g03830.t01 | 1288 | SNARE associated Golgi protein, putative, expressed | 744 | 39.006907 | 1141 | 80.537873 | -1.045938 | 5.74E-54 | 3.47E-55 | down |
| VIT_13s0084g00110.t01 | 990 | ABC transporter I family member 6 | 313 | 21.349789 | 480 | 44.079475 | -1.045885 | 2.68E-23 | 3.38E-24 | down |
| VIT_09s0018g00560.t01 | 635 | cytochrome c oxidase assembly factor 5 | 60 | 6.3806066 | 92 | 13.17178 | -1.045685 | 2.35E-05 | 8.91E-06 | down |
| VIT_19s0085g01030.t01 | 1450 | ICE-like protease p20 domain containing protein, putative, expressed | 2301 | 107.16009 | 3528 | 221.20297 | -1.045603 | 6.81E-165 | 1.36E-166 | down |
| VIT_04s0008g06780.t01 | 1009 | Glutathione peroxidase | 732 | 48.989652 | 1122 | 101.09556 | -1.045171 | 5.04E-53 | 3.11E-54 | down |
| VIT_11s0118g00230.t01 | 1860 | C2 domain containing protein, expressed | 77 | 2.7955176 | 118 | 5.7676571 | -1.04487 | 1.50E-06 | 4.97E-07 | down |
| VIT_14s0066g02620.t01 | 1521 | RNA 3-terminal phosphate cyclase, putative, expressed | 64 | 2.8414185 | 98 | 5.8577015 | -1.043723 | 1.28E-05 | 4.70E-06 | down |
| VIT_15s0021g01350.t01 | 2172 | retinoblastoma-binding protein-like, putative, expressed | 113 | 3.5132016 | 173 | 7.2413019 | -1.043463 | 4.52E-09 | 1.20E-09 | down |
| VIT_17s0000g02430.t01 | 1939 | phosphoethanolamine N-methyltransferase-like | 113 | 3.9353655 | 173 | 8.1114532 | -1.043463 | 4.52E-09 | 1.20E-09 | down |
| VIT_11s0016g05260.t01 | 934 | Putative uncharacterized protein | 243 | 17.56887 | 372 | 36.209826 | -1.04336 | 3.12E-18 | 4.83E-19 | down |
| VIT_14s0068g01090.t01 | 1869 | histone-lysine N-methyltransferase, putative, expressed | 130 | 4.6969777 | 199 | 9.679973 | -1.04327 | 2.90E-10 | 6.99E-11 | down |
| VIT_18s0041g01220.t01 | 1286 | acetyltransferase, GNAT family, putative, expressed | 83 | 4.3583446 | 127 | 8.9782796 | -1.042659 | 6.05E-07 | 1.92E-07 | down |
| VIT_09s0002g04810.t01 | 2952 | coatomer subunit beta, putative, expressed | 681 | 15.578126 | 1042 | 32.090888 | -1.042642 | 3.98E-49 | 2.66E-50 | down |
| VIT_10s0003g03980.t01 | 1475 | putative ALA-interacting subunit 2-like | 68 | 3.1131592 | 104 | 6.4102016 | -1.04199 | 6.97E-06 | 2.49E-06 | down |
| VIT_01s0011g02960.t01 | 1342 | isoflavone reductase homolog IRL, putative, expressed | 85 | 4.2771143 | 130 | 8.8068624 | -1.04199 | 4.47E-07 | 1.40E-07 | down |
| VIT_14s0066g02410.t01 | 1937 | aldehyde dehydrogenase, putative, expressed | 140 | 4.8807084 | 214 | 10.044181 | -1.041197 | 6.39E-11 | 1.47E-11 | down |
| VIT_12s0028g03190.t01 | 1551 | reticulon domain containing protein, putative, expressed | 106 | 4.6150723 | 162 | 9.4958444 | -1.040943 | 1.56E-08 | 4.29E-09 | down |
| VIT_12s0059g00760.t01 | 1714 | adenosine 5'-phosphosulfate reductase | 443 | 17.453292 | 677 | 35.909406 | -1.040863 | 3.47E-32 | 3.36E-33 | down |
| VIT_14s0006g00250.t01 | 1225 | cysteine-rich repeat secretory protein precursor, putative, expressed | 178 | 9.8122444 | 272 | 20.1866 | -1.040743 | 1.43E-13 | 2.77E-14 | down |
| VIT_02s0154g00240.t01 | 2234 | oxysterol-binding protein, putative, expressed | 89 | 2.6902416 | 136 | 5.5345984 | -1.040743 | 2.44E-07 | 7.45E-08 | down |
| VIT_01s0026g00330.t01 | 1701 | NHL repeat-containing protein, putative, expressed | 197 | 7.8207131 | 301 | 16.087648 | -1.040581 | 6.69E-15 | 1.22E-15 | down |
| VIT_03s0063g02040.t01 | 2389 | uncharacterized protein ycf44, putative, expressed | 430 | 12.15449 | 657 | 25.002279 | -1.04057 | 2.98E-31 | 2.96E-32 | down |
| VIT_03s0038g02620.t01 | 2182 | RNA recognition motif containing protein, putative, expressed | 561 | 17.361712 | 857 | 35.707254 | -1.040308 | 1.94E-40 | 1.55E-41 | down |
| VIT_06s0004g03650.t01 | 1449 | response regulator receiver domain containing protein, expressed | 294 | 13.701351 | 449 | 28.171393 | -1.039913 | 1.14E-21 | 1.53E-22 | down |
| VIT_13s0320g00060.t01 | 1666 | carboxyl-terminal peptidase, putative, expressed | 1028 | 41.667991 | 1569 | 85.62061 | -1.039019 | 4.56E-73 | 2.09E-74 | down |
| VIT_06s0004g02700.t01 | 1544 | lysine ketoglutarate reductase trans-splicing related 1, putative, expressed | 135 | 5.904334 | 206 | 12.129707 | -1.038698 | 1.62E-10 | 3.83E-11 | down |
| VIT_13s0019g01230.t01 | 1347 | dolichyl-phosphate beta-glycosyltransferase, putative, expressed | 78 | 3.9103124 | 119 | 8.0317418 | -1.038429 | 1.53E-06 | 5.08E-07 | down |
| VIT_03s0097g00530.t01 | 2340 | DnaK family protein, putative, expressed | 472 | 13.62105 | 720 | 27.973513 | -1.038224 | 5.01E-34 | 4.61E-35 | down |
| VIT_08s0040g02190.t01 | 919 | Putative uncharacterized protein | 59 | 4.3353178 | 90 | 8.9034305 | -1.038224 | 3.25E-05 | 1.25E-05 | down |
| VIT_16s0098g01240.t01 | 903 | Putative uncharacterized protein | 40 | 2.9912774 | 61 | 6.1414717 | -1.037823 | 0.00069728 | 0.00032465 | down |
| VIT_08s0007g06880.t01 | 717 | gb protein, putative, expressed | 80 | 7.5345145 | 122 | 15.469314 | -1.037823 | 1.13E-06 | 3.70E-07 | down |
| VIT_06s0061g01590.t01 | 1941 | peptidylprolyl isomerase domain and WD repeat-containing protein 1-like | 80 | 2.7832287 | 122 | 5.7143215 | -1.037823 | 1.13E-06 | 3.70E-07 | down |
| VIT_18s0001g00190.t01 | 2163 | ATP-dependent zinc metalloprotease FTSH | 5550 | 173.26902 | 8463 | 355.71174 | -1.037695 | 0 | 0 | down |
| VIT_00s0357g00070.t01 | 859 | (3R)-hydroxymyristoyl-[acyl-carrier-protein] dehydratase-like | 42 | 3.3017225 | 64 | 6.7735631 | -1.036696 | 0.00051429 | 0.00023429 | down |
| VIT_00s0455g00040.t01 | 2490 | glycosyl transferase 8 domain containing protein, putative, expressed | 506 | 13.722575 | 771 | 28.150454 | -1.036607 | 2.81E-36 | 2.47E-37 | down |
| VIT_16s0039g01410.t01 | 1503 | Cyclopropane-fatty-acyl-phospholipid synthase, putative, expressed | 728 | 32.708215 | 1109 | 67.081527 | -1.036263 | 1.05E-51 | 6.64E-53 | down |
| VIT_04s0023g01810.t01 | 658 | ribosomal protein L6, putative, expressed | 130 | 13.341415 | 198 | 27.357076 | -1.036002 | 4.10E-10 | 9.98E-11 | down |
| VIT_05s0020g01540.t01 | 1875 | histone-arginine methyltransferase CARM1, putative, expressed | 203 | 7.3110408 | 309 | 14.982614 | -1.035141 | 3.80E-15 | 6.82E-16 | down |
| VIT_04s0008g02040.t01 | 1707 | G-protein alpha subunit, putative, expressed | 113 | 4.4702248 | 172 | 9.1606291 | -1.035099 | 6.38E-09 | 1.71E-09 | down |
| VIT_07s0104g01270.t01 | 910 | KIP1, putative, expressed | 69 | 5.1202615 | 105 | 10.490067 | -1.034735 | 7.12E-06 | 2.54E-06 | down |
| VIT_14s0060g02220.t01 | 1399 | F-actin-capping protein subunit alpha, putative, expressed | 140 | 6.7576355 | 213 | 13.84179 | -1.03444 | 9.02E-11 | 2.09E-11 | down |
| VIT_12s0055g00330.t01 | 2337 | Putative uncharacterized protein | 119 | 3.438529 | 181 | 7.0412576 | -1.034042 | 2.57E-09 | 6.67E-10 | down |
| VIT_04s0008g00900.t01 | 1149 | MYB family transcription factor, putative, expressed | 48 | 2.8210167 | 73 | 5.7760801 | -1.033876 | 0.0002068 | 8.85E-05 | down |
| VIT_02s0025g01240.t01 | 1520 | UDP-glucoronosyl/UDP-glucosyl transferase, putative, expressed | 400 | 17.770549 | 608 | 36.365567 | -1.033085 | 1.15E-28 | 1.22E-29 | down |
| VIT_06s0004g04910.t01 | 1333 | PGR5-like protein 1B | 100 | 5.065873 | 152 | 10.366778 | -1.033085 | 5.48E-08 | 1.57E-08 | down |
| VIT_09s0002g03150.t01 | 1087 | GDSL-like lipase/acylhydrolase, putative, expressed | 1068 | 66.347743 | 1623 | 135.7436 | -1.032765 | 8.25E-75 | 3.65E-76 | down |

| | | | | | | | | | | |
|---|---|---|---|---|---|---|---|---|---|---|
| VIT_07s0151g00950.t01 | 845 | expressed protein | 102 | 8.1513193 | 155 | 16.676517 | -1.032713 | 4.04E-08 | 1.15E-08 | down |
| VIT_08s0007g02120.t01 | 1725 | starch binding domain containing protein, putative, expressed | 77 | 3.0142972 | 117 | 6.1663353 | -1.032592 | 2.11E-06 | 7.12E-07 | down |
| VIT_10s0003g00180.t01 | 916 | drought induced 19 protein, putative, expressed | 104 | 7.6669443 | 158 | 15.681658 | -1.032355 | 2.99E-08 | 8.39E-09 | down |
| VIT_11s0016g01950.t01 | 2103 | CK1_CaseinKinase_1.3 - CK1 includes the casein kinase 1 kinases, expressed | 836 | 26.844261 | 1270 | 54.902841 | -1.032267 | 1.06E-58 | 5.94E-60 | down |
| VIT_12s0059g01340.t01 | 1874 | armadillo/beta-catenin-like repeat family protein, expressed | 1360 | 49.006509 | 2064 | 100.13144 | -1.03085 | 1.32E-94 | 4.67E-96 | down |
| VIT_18s0001g14980.t01 | 1522 | dehydrogenase E1 component domain containing protein, expressed | 58 | 2.5733436 | 88 | 5.2565209 | -1.030464 | 4.50E-05 | 1.77E-05 | down |
| VIT_05s0020g01200.t01 | 2531 | ATKINESIN-13A/KINESIN-13A, putative, expressed | 358 | 9.5515824 | 543 | 19.504645 | -1.030006 | 1.36E-25 | 1.58E-26 | down |
| VIT_18s0001g08390.t01 | 394 | chloroplast DNA | 217 | 37.191865 | 329 | 75.915429 | -1.029406 | 6.43E-16 | 1.11E-16 | down |
| VIT_01s0010g02370.t01 | 2593 | signal recognition particle 72 kDa protein, putative, expressed | 287 | 7.4741847 | 435 | 15.25166 | -1.028978 | 1.10E-20 | 1.54E-21 | down |
| VIT_18s0001g11750.t01 | 964 | ribosomal protein, putative, expressed | 877 | 61.433747 | 1329 | 125.33672 | -1.028706 | 4.86E-61 | 2.61E-62 | down |
| VIT_14s0128g00050.t01 | 1069 | thylakoid lumen protein, putative, expressed | 1310 | 82.751911 | 1985 | 168.81583 | -1.028586 | 1.07E-90 | 3.97E-92 | down |
| VIT_03s0038g01780.t01 | 1101 | triosephosphate isomerase, chloroplast precursor, putative, expressed | 2293 | 140.63751 | 3474 | 286.8619 | -1.028375 | 4.85E-158 | 1.02E-159 | down |
| VIT_09s0018g01780.t01 | 1407 | uridylyltransferase-related, putative, expressed | 1349 | 64.744413 | 2043 | 132.00933 | -1.027812 | 3.33E-93 | 1.20E-94 | down |
| VIT_07s0141g00590.t01 | 1496 | CorA-like magnesium transporter protein, putative, expressed | 247 | 11.149357 | 374 | 22.728479 | -1.027541 | 6.75E-18 | 1.06E-18 | down |
| VIT_01s0010g03300.t01 | 2692 | mannosyl-oligosaccharide glucosidase, putative, expressed | 107 | 2.6840658 | 162 | 5.4710456 | -1.027397 | 2.24E-08 | 6.25E-09 | down |
| VIT_13s0019g04380.t01 | 2359 | auxin response factor 18 | 144 | 4.1221045 | 218 | 8.4015405 | -1.027273 | 6.93E-11 | 1.59E-11 | down |
| VIT_18s0072g00400.t01 | 848 | thioredoxin | 325 | 25.880458 | 492 | 52.747226 | -1.027232 | 3.42E-23 | 4.34E-24 | down |
| VIT_15s0021g02210.t01 | 1277 | Putative uncharacterized protein | 325 | 17.186083 | 492 | 35.027132 | -1.027232 | 3.42E-23 | 4.34E-24 | down |
| VIT_09s0002g03510.t01 | 2770 | RNA polymerase II-associated factor 1 homolog | 185 | 4.5099986 | 280 | 9.1898545 | -1.026915 | 1.17E-13 | 2.25E-14 | down |
| VIT_15s0021g01300.t01 | 1880 | pentatricopeptide repeat-containing protein | 261 | 9.3749099 | 395 | 19.101594 | -1.026816 | 8.07E-19 | 1.21E-19 | down |
| VIT_13s0019g00010.t01 | 2431 | probable sulfate transporter 4.2-like | 113 | 3.1389032 | 171 | 6.3950144 | -1.026687 | 9.02E-09 | 2.44E-09 | down |
| VIT_14s0006g02300.t01 | 282 | DNA-directed RNA polymerase subunit beta, putative, expressed | 39 | 9.3389907 | 59 | 19.020997 | -1.026254 | 0.00096491 | 0.00046009 | down |
| VIT_08s0007g04530.t01 | 2367 | carotene epsilon-monooxygenase | 279 | 7.9595843 | 422 | 16.208565 | -1.025991 | 5.22E-20 | 7.49E-21 | down |
| VIT_18s0001g03470.t01 | 1236 | flavonol synthase/flavanone 3-hydroxylase | 121 | 6.6107593 | 183 | 13.460556 | -1.02585 | 2.68E-09 | 6.95E-10 | down |
| VIT_15s0021g01580.t01 | 1215 | proteasome subunit, putative, expressed | 162 | 9.0037449 | 245 | 18.332436 | -1.025801 | 4.55E-12 | 9.63E-13 | down |
| VIT_02s0012g02790.t01 | 1147 | secretory carrier-associated membrane protein, putative, expressed | 172 | 10.126269 | 260 | 20.608211 | -1.025117 | 1.00E-12 | 2.03E-13 | down |
| VIT_12s0028g01820.t01 | 1752 | aminotransferase, classes I and II, domain containing protein, expressed | 131 | 5.0491891 | 198 | 10.274518 | -1.024947 | 5.87E-10 | 1.45E-10 | down |
| VIT_02s0109g00360.t01 | 1111 | translocase of chloroplast 34, putative, expressed | 315 | 19.146127 | 476 | 38.951417 | -1.024623 | 2.20E-22 | 2.88E-23 | down |
| VIT_04s0008g00800.t01 | 1681 | ferrochelatase | 92 | 3.6957668 | 139 | 7.5175696 | -1.024393 | 2.59E-07 | 7.93E-08 | down |
| VIT_11s0052g00410.t01 | 1140 | NADP-dependent oxidoreductase, putative, expressed | 49 | 2.902523 | 74 | 5.9014297 | -1.023757 | 0.00021038 | 9.03E-05 | down |
| VIT_18s0001g10440.t01 | 3069 | cold-induced protein, putative, expressed | 202 | 4.4446639 | 305 | 9.0351075 | -1.023467 | 1.04E-14 | 1.91E-15 | down |
| VIT_04s0008g03590.t01 | 1227 | phosphorylase domain containing protein, putative, expressed | 308 | 16.950816 | 465 | 34.45393 | -1.023314 | 7.57E-22 | 1.01E-22 | down |
| VIT_17s0000g01380.t01 | 1700 | ThiF family domain containing protein, putative, expressed | 157 | 6.2364174 | 237 | 12.67447 | -1.023136 | 1.14E-11 | 2.50E-12 | down |
| VIT_03s0038g02930.t01 | 1738 | transporter, major facilitator family, putative, expressed | 420 | 16.31864 | 634 | 33.164225 | -1.023107 | 2.16E-29 | 2.24E-30 | down |
| VIT_18s0041g00420.t01 | 1806 | patellin protein, putative, expressed | 503 | 18.807656 | 759 | 38.208009 | -1.022555 | 5.86E-35 | 5.30E-36 | down |
| VIT_18s0122g01380.t01 | 2633 | PPR repeat containing protein, expressed | 169 | 4.3343132 | 255 | 8.804804 | -1.022487 | 1.87E-12 | 3.85E-13 | down |
| VIT_14s0030g01300.t01 | 1002 | acyltransferase-like protein | 114 | 7.6828362 | 172 | 15.605982 | -1.022388 | 9.19E-09 | 2.48E-09 | down |
| VIT_02s0012g00740.t01 | 2203 | dynamin-related protein 1E-like | 244 | 7.4792797 | 368 | 15.18671 | -1.021838 | 1.78E-17 | 2.84E-18 | down |
| VIT_19s0014g02510.t01 | 168 | chloroplast DNA | 67 | 26.930844 | 101 | 54.656581 | -1.021136 | 1.36E-05 | 5.01E-06 | down |
| VIT_06s0009g01130.t01 | 2010 | molybdopterin biosynthesis protein CNX1, putative, expressed | 213 | 7.1559614 | 321 | 14.519088 | -1.020733 | 2.33E-15 | 4.13E-16 | down |
| VIT_01s0010g02390.t01 | 678 | Putative uncharacterized protein | 217 | 21.612972 | 327 | 43.847863 | -1.020609 | 1.27E-15 | 2.22E-16 | down |
| VIT_00s1286g00010.t01 | 1229 | hsp70-Hsp90 organizing protein 3-like | 168 | 9.2308532 | 253 | 18.715396 | -1.01969 | 2.58E-12 | 5.36E-13 | down |
| VIT_01s0026g02120.t01 | 1226 | ATEB1A-like microtubule associated protein, putative, expressed | 87 | 4.7919605 | 131 | 9.714293 | -1.019493 | 6.54E-07 | 2.09E-07 | down |
| VIT_18s0001g04160.t01 | 2109 | CPuORF13 - conserved peptide uORF-containing transcript, expressed | 1153 | 36.917915 | 1736 | 74.834785 | -1.019388 | 2.82E-78 | 1.19E-79 | down |
| VIT_10s0003g04480.t01 | 2288 | DUF630/DUF632 domains containing protein, putative, expressed | 91 | 2.6857762 | 137 | 5.4437092 | -1.019251 | 3.57E-07 | 1.11E-07 | down |
| VIT_19s0027g00380.t01 | 1319 | amine oxidase-related, putative, expressed | 93 | 4.7612677 | 140 | 9.6496956 | -1.019138 | 2.64E-07 | 8.08E-08 | down |
| VIT_02s0025g03930.t01 | 1129 | transcription initiation factor IIE subunit beta, putative, expressed | 95 | 5.6821685 | 143 | 11.515226 | -1.019029 | 1.95E-07 | 5.90E-08 | down |
| VIT_02s0033g01020.t01 | 1820 | transferase family protein, putative, expressed | 2398 | 88.973819 | 3609 | 180.2793 | -1.018781 | 1.19E-161 | 2.44E-163 | down |
| VIT_11s0052g00430.t01 | 1770 | transporter-related, putative, expressed | 105 | 4.0059034 | 158 | 8.1154797 | -1.018549 | 4.28E-08 | 1.22E-08 | down |
| VIT_07s0005g00500.t01 | 681 | LSM domain containing protein, expressed | 210 | 20.823639 | 316 | 42.186194 | -1.018549 | 4.35E-15 | 7.83E-16 | down |
| VIT_13s0067g02220.t01 | 1368 | transmembrane amino acid transporter protein, putative, expressed | 121 | 5.972879 | 182 | 12.095273 | -1.017945 | 3.76E-09 | 9.87E-10 | down |
| VIT_02s0025g00340.t01 | 1727 | monodehydroascorbate reductase | 393 | 15.366843 | 591 | 31.111827 | -1.017642 | 3.08E-27 | 3.41E-28 | down |
| VIT_09s0002g01910.t01 | 4697 | myosin, putative, expressed | 179 | 2.573457 | 269 | 5.2066945 | -1.01666 | 5.79E-13 | 1.15E-13 | down |
| VIT_06s0004g06870.t01 | 1580 | peptidase, M24 family protein, putative, expressed | 231 | 9.8727772 | 347 | 19.966538 | -1.016056 | 2.14E-16 | 3.58E-17 | down |
| VIT_14s0060g00230.t01 | 1908 | pectinacetylesterase domain containing protein, expressed | 1199 | 42.435103 | 1801 | 85.815496 | -1.01598 | 9.77E-81 | 4.05E-82 | down |
| VIT_19s0015g00530.t01 | 1355 | fasciclin-like arabinogalactan protein 8 precursor, putative, expressed | 484 | 24.120733 | 727 | 48.778168 | -1.015962 | 3.50E-33 | 3.30E-34 | down |
| VIT_15s0024g00070.t01 | 2069 | methylenetetrahydrofolate reductase, putative, expressed | 1204 | 39.29619 | 1808 | 79.445318 | -1.015573 | 5.40E-81 | 2.23E-82 | down |
| VIT_13s0019g00250.t01 | 724 | 60S ribosomal protein L23A, putative, expressed | 969 | 90.379442 | 1455 | 182.70684 | -1.015464 | 2.37E-65 | 1.20E-66 | down |
| VIT_13s0067g01930.t01 | 1605 | leaf senescence related protein, putative, expressed | 92 | 3.8707688 | 138 | 7.8168976 | -1.013976 | 3.63E-07 | 1.13E-07 | down |
| VIT_04s0008g03630.t01 | 1175 | U-box domain-containing protein 25 | 128 | 7.3562511 | 192 | 14.855721 | -1.013976 | 1.54E-09 | 3.93E-10 | down |
| VIT_06s0080g00560.t01 | 1196 | N-carbamoylputrescine amidase, putative, expressed | 84 | 4.7427753 | 126 | 9.5778876 | -1.013976 | 1.22E-06 | 4.01E-07 | down |
| VIT_03s0063g00520.t01 | 779 | protein disulfide-isomerase SCO2 | 70 | 6.0679924 | 105 | 12.254122 | -1.013976 | 1.02E-05 | 3.71E-06 | down |
| VIT_14s0030g01870.t01 | 2189 | LSTK-1-like kinase, putative, expressed | 96 | 2.9614876 | 144 | 5.9806323 | -1.013976 | 1.98E-07 | 6.01E-08 | down |
| VIT_09s0002g07130.t01 | 1061 | Putative uncharacterized protein | 48 | 3.0549935 | 72 | 6.1694647 | -1.013976 | 0.00029023 | 0.00012734 | down |
| VIT_03s0063g00500.t01 | 550 | TP53RK-binding protein, putative, expressed | 72 | 8.8400404 | 108 | 17.852187 | -1.013976 | 7.54E-06 | 2.70E-06 | down |
| VIT_03s0063g02290.t01 | 1179 | dehydrodolichyl diphosphate syntase complex subunit NUS1 | 144 | 8.2477052 | 216 | 16.655985 | -1.013976 | 1.37E-10 | 3.21E-11 | down |
| VIT_14s0060g00910.t01 | 1539 | xaa-Pro aminopeptidase, putative, expressed | 74 | 3.2469645 | 111 | 6.5571442 | -1.013976 | 5.55E-06 | 1.96E-06 | down |
| VIT_04s0008g00520.t01 | 2147 | 6-phosphofructokinase, putative, expressed | 148 | 4.6549403 | 222 | 9.4005075 | -1.013976 | 7.46E-11 | 1.72E-11 | down |
| VIT_04s0008g06850.t01 | 773 | integral membrane transporter family protein, putative, expressed | 40 | 3.4943382 | 60 | 7.0567077 | -1.013976 | 0.00097932 | 0.00046928 | down |
| VIT_15s0046g01540.t01 | 1406 | F-box domain and kelch repeat containing protein, expressed | 86 | 4.1304519 | 129 | 8.3413196 | -1.013976 | 9.02E-07 | 2.92E-07 | down |
| VIT_01s0137g00690.t01 | 2195 | eukaryotic translation initiation factor 4B, putative, expressed | 371 | 11.413631 | 556 | 23.028765 | -1.012679 | 1.72E-25 | 2.02E-26 | down |
| VIT_04s0008g06040.t01 | 1683 | aminotransferase, classes I and II, domain containing protein, expressed | 672 | 26.963086 | 1007 | 54.397097 | -1.012544 | 2.51E-45 | 1.82E-46 | down |
| VIT_17s0000g00980.t01 | 1099 | ribosomal protein L7Ae, putative, expressed | 1036 | 63.65705 | 1552 | 128.38799 | -1.012118 | 2.82E-69 | 1.37E-70 | down |
| VIT_04s0159g00020.t01 | 3174 | Putative uncharacterized protein | 199 | 4.2338025 | 298 | 8.5357112 | -1.011557 | 3.87E-14 | 7.27E-15 | down |
| VIT_11s0016g00370.t01 | 2731 | AT-rich interactive domain-containing protein 4-like | 187 | 4.6238565 | 280 | 9.3210901 | -1.011402 | 2.41E-13 | 4.69E-14 | down |
| VIT_01s0011g00600.t01 | 2021 | triose phosphate/phosphate translocator | 181 | 6.04779 | 271 | 12.190832 | -1.011317 | 5.99E-13 | 1.19E-13 | down |
| VIT_18s0001g14250.t01 | 3625 | TMV resistance protein N-like | 623 | 11.605517 | 932 | 23.374282 | -1.010111 | 7.34E-42 | 5.66E-43 | down |
| VIT_18s0122g00260.t01 | 3975 | tetratricopeptide repeat domain containing protein, expressed | 218 | 3.7034271 | 326 | 7.4560848 | -1.009557 | 2.55E-15 | 4.52E-16 | down |
| VIT_18s0001g10420.t01 | 1772 | remorin C-terminal domain containing protein, putative, expressed | 97 | 3.6965149 | 145 | 7.4393443 | -1.00901 | 2.02E-07 | 6.11E-08 | down |
| VIT_17s0000g02310.t01 | 1771 | zinc finger family protein, putative, expressed | 95 | 3.6223423 | 142 | 7.2895405 | -1.008905 | 2.73E-07 | 8.38E-08 | down |
| VIT_01s0010g01900.t01 | 1217 | PPR repeat domain containing protein, putative, expressed | 237 | 13.150498 | 354 | 26.444969 | -1.007876 | 1.69E-16 | 2.82E-17 | down |
| VIT_19s0014g03930.t01 | 2022 | tRNA methyltransferase, putative, expressed | 79 | 2.6383377 | 118 | 5.30556 | -1.007876 | 3.09E-06 | 1.06E-06 | down |
| VIT_10s0003g00010.t01 | 825 | 30S ribosomal protein S5 | 1465 | 119.91351 | 2188 | 241.11473 | -1.007726 | 1.36E-96 | 4.70E-98 | down |
| VIT_14s0030g01830.t01 | 1397 | protein kinase PKN/PRK1, effector, putative, expressed | 75 | 3.6253447 | 112 | 7.2887321 | -1.00755 | 5.65E-06 | 2.00E-06 | down |
| VIT_02s0109g00080.t01 | 1697 | phosphoribulokinase/Uridine kinase family protein, expressed | 6318 | 251.40981 | 9434 | 505.41067 | -1.007415 | 0 | 0 | down |
| VIT_15s0024g00260.t01 | 883 | transporter, monovalent cation:proton antiporter-2 family, putative, expressed | 73 | 5.5827297 | 109 | 11.222669 | -1.007373 | 7.67E-06 | 2.75E-06 | down |
| VIT_12s0059g02440.t01 | 1541 | traB domain-containing protein-like | 138 | 6.0472913 | 206 | 12.153321 | -1.00699 | 4.75E-10 | 1.16E-10 | down |
| VIT_17s0000g08590.t01 | 1542 | Putative uncharacterized protein | 67 | 2.9340997 | 100 | 5.8958442 | -1.00678 | 1.90E-05 | 7.15E-06 | down |
| VIT_15s0024g00920.t01 | 741 | nodulin, putative, expressed | 65 | 5.9235164 | 97 | 11.901012 | -1.006559 | 2.59E-05 | 9.85E-06 | down |
| VIT_14s0066g00720.t01 | 1329 | WD repeat-containing protein 82-B-like | 63 | 3.2011057 | 94 | 6.4303298 | -1.006322 | 3.50E-05 | 1.36E-05 | down |
| VIT_05s0124g00400.t01 | 1130 | 2-isopropylmalate synthase B, putative, expressed | 177 | 10.577408 | 264 | 21.240066 | -1.005802 | 1.54E-12 | 3.14E-13 | down |
| VIT_00s0252g00070.t01 | 1570 | eukaryotic translation initiation factor 3 subunit E, putative, expressed | 407 | 17.505689 | 607 | 35.149521 | -1.005681 | 1.99E-27 | 2.19E-28 | down |
| VIT_08s0007g04340.t01 | 946 | peptidyl-prolyl cis-trans isomerase, FKBP-type, putative, expressed | 629 | 44.899753 | 938 | 90.14509 | -1.005541 | 8.11E-42 | 6.27E-43 | down |
| VIT_11s0078g00250.t01 | 1643 | cytidylyltransferase domain containing protein, expressed | 218 | 8.9599043 | 325 | 17.983581 | -1.005125 | 3.56E-15 | 6.38E-16 | down |
| VIT_18s0001g05930.t01 | 2219 | pentatricopeptide, putative, expressed | 100 | 3.0431765 | 149 | 6.1046299 | -1.004326 | 1.52E-07 | 4.54E-08 | down |
| VIT_11s0016g02550.t01 | 2108 | serine/threonine protein phosphatase 2A 55 kDa regulatory subunit B, putative, expr | 147 | 4.7090269 | 219 | 9.4450417 | -1.004128 | 1.44E-10 | 3.38E-11 | down |
| VIT_03s0038g00910.t01 | 1043 | ribosome-binding factor A, chloroplast precursor, putative, expressed | 382 | 24.732243 | 569 | 49.597334 | -1.00387 | 1.06E-25 | 1.23E-26 | down |

| | | | | | | | | | | |
|---|---|---|---|---|---|---|---|---|---|---|
| VIT_18s0001g14100.t01 | 1260 | Abscisic acid-responsive protein | 190 | 10.182807 | 283 | 20.419555 | -1.003816 | 2.53E-13 | 4.94E-14 | down |
| VIT_12s0035g01160.t01 | 3421 | leucyl-tRNA synthetase, cytoplasmic, putative, expressed | 677 | 13.363494 | 1008 | 26.787848 | -1.003281 | 1.06E-44 | 7.73E-46 | down |
| VIT_09s0002g09050.t01 | 1105 | mitochondrial glycoprotein, putative, expressed | 45 | 2.7500126 | 67 | 5.5124276 | -1.00325 | 0.00054334 | 0.00024849 | down |
| VIT_18s0001g09370.t01 | 1228 | arginine N-methyltransferase 2-like | 86 | 4.7291657 | 128 | 9.4763693 | -1.002749 | 1.26E-06 | 4.15E-07 | down |
| VIT_05s0077g01840.t01 | 903 | 60S ribosomal protein L19-like | 1141 | 85.326187 | 1698 | 170.95441 | -1.002551 | 1.55E-74 | 6.91E-76 | down |
| VIT_18s0001g06370.t01 | 1287 | thylakoid lumenal 29 kDa protein | 1219 | 63.960169 | 1814 | 128.14129 | -1.00249 | 1.49E-79 | 6.18E-81 | down |
| VIT_08s0007g03960.t01 | 1614 | bifunctional enolase 2/transcriptional activator-like | 3463 | 144.88833 | 5153 | 290.25986 | -1.002404 | 1.98E-224 | 2.81E-226 | down |
| VIT_05s0077g01880.t01 | 1067 | DAG protein, chloroplast precursor, putative, expressed | 121 | 7.6578242 | 180 | 15.336931 | -1.002003 | 7.38E-09 | 1.98E-09 | down |
| VIT_15s0048g00870.t01 | 1416 | AT hook motif domain containing protein, expressed | 78 | 3.7197675 | 116 | 7.4477503 | -1.001592 | 4.24E-06 | 1.48E-06 | down |
| VIT_13s0064g01660.t01 | 1754 | beta-glucosidase 13-like | 425 | 16.362279 | 632 | 32.758036 | -1.001475 | 2.53E-28 | 2.70E-29 | down |
| VIT_08s0007g01850.t01 | 2743 | Putative uncharacterized protein | 347 | 8.5425614 | 516 | 17.10229 | -1.001449 | 2.56E-23 | 3.22E-24 | down |
| VIT_04s0043g00740.t01 | 3832 | protein PIR-like | 191 | 3.365831 | 284 | 6.7378791 | -1.001332 | 2.57E-13 | 5.02E-14 | down |
| VIT_07s0005g05500.t01 | 893 | Putative uncharacterized protein | 152 | 11.494142 | 226 | 23.008449 | -1.001265 | 7.97E-11 | 1.84E-11 | down |
| VIT_14s0060g00900.t01 | 718 | Putative uncharacterized protein | 703 | 66.117333 | 1045 | 132.319 | -1.00092 | 3.96E-46 | 2.81E-47 | down |
| VIT_10s0003g01100.t01 | 1136 | vignain-like | 202 | 12.007635 | 75 | 6.0022393 | 1.0003793 | 1.50E-07 | 4.51E-08 | up |
| VIT_18s0001g02680.t01 | 1125 | BTB/POZ domain-containing protein | 299 | 17.947465 | 111 | 8.9701732 | 1.0005723 | 1.20E-10 | 2.82E-11 | up |
| VIT_12s0059g02470.t01 | 1430 | peroxisome biogenesis protein 22-like | 2048 | 96.711553 | 760 | 48.317886 | 1.0011309 | 1.02E-66 | 5.05E-68 | up |
| VIT_06s0004g07270.t01 | 747 | UDP-glycosyltransferase 87A1-like | 151 | 13.650256 | 56 | 6.8155012 | 1.0020363 | 6.16E-06 | 2.18E-06 | up |
| VIT_11s0037g00730.t01 | 4075 | Putative uncharacterized protein | 321 | 5.3193904 | 119 | 2.6549095 | 1.0025982 | 2.21E-11 | 4.92E-12 | up |
| VIT_06s0004g03320.t01 | 1933 | IBR domain containing protein, expressed | 494 | 17.257566 | 183 | 8.6069565 | 1.0036539 | 5.95E-17 | 9.74E-18 | up |
| VIT_09s0018g01660.t01 | 2235 | integral membrane transporter family protein, putative, expressed | 270 | 8.1577554 | 100 | 4.0677368 | 1.0039459 | 9.02E-10 | 2.26E-10 | up |
| VIT_08s0058g00860.t01 | 844 | protein LURP-one-related 15-like | 208 | 16.641993 | 77 | 8.2942792 | 1.0046397 | 8.71E-08 | 2.55E-08 | up |
| VIT_18s0072g01170.t01 | 3150 | multiple C2 and transmembrane domain-containing protein 2-like | 927 | 19.872551 | 343 | 9.8995155 | 1.0053473 | 5.85E-31 | 5.86E-32 | up |
| VIT_08s0007g06400.t01 | 1297 | zinc finger C-x8-C-x5-C-x3-H type family protein, expressed | 1509 | 78.565831 | 558 | 39.113312 | 1.0062423 | 7.90E-50 | 5.21E-51 | up |
| VIT_13s0064g01470.t01 | 1528 | siroheme synthase, putative, expressed | 1025 | 45.298618 | 379 | 22.549983 | 1.0063407 | 3.55E-34 | 3.26E-35 | up |
| VIT_01s0146g00410.t01 | 1030 | GRAM domain containing protein, expressed | 165 | 10.817606 | 61 | 5.3842223 | 1.0065714 | 2.00E-06 | 6.74E-07 | up |
| VIT_14s0006g01620.t01 | 1361 | MYB family transcription factor, putative, expressed | 138 | 6.84708 | 51 | 3.4067669 | 1.0070856 | 1.48E-05 | 5.47E-06 | up |
| VIT_00s0216g00040.t01 | 1105 | ER lumen protein retaining receptor-like | 490 | 29.944581 | 181 | 14.891782 | 1.0077786 | 6.26E-17 | 1.02E-17 | up |
| VIT_03s0038g02310.t01 | 954 | flavonoid-related R2R3 MYB 4a repressor transcription factor (MYB4A) | 352 | 24.916024 | 130 | 12.388689 | 1.0080503 | 1.80E-12 | 3.70E-13 | up |
| VIT_10s0003g03870.t01 | 1833 | aminotransferase, putative, expressed | 149 | 5.4891898 | 55 | 2.7279135 | 1.0087953 | 6.31E-06 | 2.24E-06 | up |
| VIT_16s0022g02200.t01 | 2518 | subtilisin-like protease | 5790 | 155.27705 | 2137 | 77.157681 | 1.0089629 | 1.85E-189 | 3.27E-191 | up |
| VIT_15s0021g00650.t01 | 1228 | purine permease, putative, expressed | 206 | 11.328001 | 76 | 5.6265942 | 1.0095595 | 8.92E-08 | 2.61E-08 | up |
| VIT_02s0241g00140.t01 | 704 | OsCML7 - Calmodulin-related calcium sensor protein, expressed | 5053 | 484.68668 | 1863 | 240.58612 | 1.010499 | 7.72E-166 | 1.52E-167 | up |
| VIT_08s0007g02920.t01 | 1402 | WD repeat-containing protein LWD1-like | 494 | 23.793777 | 182 | 11.801949 | 1.0115591 | 3.70E-17 | 5.99E-18 | up |
| VIT_07s0031g01380.t01 | 1765 | cytochrome P450 71A1-like | 3598 | 137.65782 | 1325 | 68.249825 | 1.0121893 | 1.19E-118 | 3.39E-120 | up |
| VIT_01s0026g01080.t01 | 1180 | serine/threonine-protein phosphatase PP2A-2 catalytic subunit | 1252 | 71.648444 | 461 | 35.518064 | 1.0123824 | 6.37E-42 | 4.91E-43 | up |
| VIT_01s0011g04640.t01 | 1395 | Putative uncharacterized protein | 546 | 26.430348 | 201 | 13.099425 | 1.012692 | 6.98E-19 | 1.05E-19 | up |
| VIT_08s0007g05980.t01 | 1426 | IQ calmodulin-binding motif family protein, putative, expressed | 174 | 8.2397525 | 64 | 4.080288 | 1.01393 | 8.81E-07 | 2.85E-07 | up |
| VIT_17s0000g09570.t01 | 1589 | cytochrome P450 71A4-like | 185 | 7.8619862 | 68 | 3.8905893 | 1.0149051 | 3.77E-07 | 1.18E-07 | up |
| VIT_12s0142g00120.t01 | 747 | CS domain containing protein, putative, expressed | 351 | 31.730065 | 129 | 15.699994 | 1.0150865 | 1.43E-12 | 2.93E-13 | up |
| VIT_12s0059g01050.t01 | 670 | glycine-rich protein DOT1-like | 234 | 23.584436 | 86 | 11.669548 | 1.0150865 | 9.42E-09 | 2.55E-09 | up |
| VIT_03s0063g01900.t01 | 587 | mitochondrial import receptor subunit TOM6 homolog | 1875 | 215.69874 | 689 | 106.71157 | 1.0153012 | 1.42E-62 | 7.45E-64 | up |
| VIT_16s0039g02550.t01 | 712 | seed specific protein Bn15D1B, putative, expressed | 991 | 93.989233 | 364 | 46.478463 | 1.0159331 | 1.39E-33 | 1.30E-34 | up |
| VIT_01s0011g03200.t01 | 1181 | GDT1-like protein 4 | 1305 | 74.61825 | 479 | 36.873638 | 1.0169388 | 5.67E-44 | 4.21E-45 | up |
| VIT_17s0000g05410.t01 | 2339 | probable beta-1,3-galactosyltransferase 19-like | 218 | 6.2937678 | 80 | 3.109497 | 1.0172427 | 2.97E-08 | 8.35E-09 | up |
| VIT_08s0040g01820.t01 | 447 | Putative uncharacterized protein | 387 | 58.463914 | 142 | 28.880931 | 1.0174291 | 8.49E-14 | 1.62E-14 | up |
| VIT_07s0104g00160.t01 | 633 | small nuclear ribonucleoprotein Sm D1-like | 1088 | 116.06723 | 399 | 57.305929 | 1.0182044 | 6.66E-37 | 5.76E-38 | up |
| VIT_13s0019g02810.t01 | 1416 | UDP-glycosyltransferase 76C4-like | 180 | 8.5840788 | 66 | 4.2375131 | 1.0184455 | 5.09E-07 | 1.60E-07 | up |
| VIT_13s0156g00150.t01 | 711 | aspartic proteinase CDR1-like | 1610 | 152.9117 | 590 | 75.441929 | 1.0192603 | 3.31E-54 | 1.98E-55 | up |
| VIT_01s0011g04040.t01 | 810 | Putative uncharacterized protein | 142 | 11.838257 | 52 | 5.836449 | 1.0202939 | 8.73E-06 | 3.14E-06 | up |
| VIT_19s0014g02880.t01 | 1565 | glycosyl hydrolase family 5 protein, putative, expressed | 3403 | 146.83583 | 1246 | 72.382582 | 1.0204896 | 8.99E-114 | 2.65E-115 | up |
| VIT_00s0978g00020.t01 | 356 | Putative uncharacterized protein | 112 | 21.244791 | 41 | 10.470423 | 1.0207894 | 8.49E-05 | 3.45E-05 | up |
| VIT_02s0033g00180.t01 | 1853 | scopoletin glucosyltransferase | 481 | 17.528877 | 176 | 8.635105 | 1.021448 | 5.47E-17 | 8.93E-18 | up |
| VIT_04s0008g07130.t01 | 839 | aspartic proteinase nepenthesin-2 precursor, putative, expressed | 164 | 13.199769 | 60 | 6.5015912 | 1.0216479 | 1.59E-06 | 5.31E-07 | up |
| VIT_11s0016g04630.t01 | 1697 | GRAS family transcription factor domain containing protein, expressed | 1534 | 61.041888 | 561 | 30.05463 | 1.0222123 | 6.09E-52 | 3.85E-53 | up |
| VIT_18s0001g15450.t01 | 1529 | dehydrogenase, putative, expressed | 793 | 35.022742 | 290 | 17.24332 | 1.0222545 | 2.41E-27 | 2.65E-28 | up |
| VIT_04s0008g01470.t01 | 501 | WRKY transcription factor 50 | 93 | 12.535154 | 34 | 6.1698068 | 1.0226825 | 0.00035354 | 0.00015714 | up |
| VIT_16s0098g01710.t01 | 541 | Putative uncharacterized protein | 427 | 53.298508 | 156 | 26.215473 | 1.0236765 | 2.96E-15 | 5.27E-16 | up |
| VIT_19s0015g01190.t01 | 782 | ubiquitin-conjugating enzyme, putative, expressed | 2568 | 221.75464 | 938 | 109.0502 | 1.0239719 | 1.50E-86 | 5.80E-88 | up |
| VIT_17s0000g07350.t01 | 2151 | exo70 exocyst complex subunit domain containing protein, expressed | 315 | 9.8890503 | 115 | 4.8605767 | 1.0247045 | 1.47E-11 | 3.23E-12 | up |
| VIT_01s0010g02260.t01 | 1439 | probable carboxylesterase 11-like | 337 | 15.81443 | 123 | 7.7709603 | 1.0250768 | 2.72E-12 | 5.66E-13 | up |
| VIT_11s0016g02360.t01 | 1150 | tetraspanin family protein, putative, expressed | 2277 | 133.70561 | 831 | 65.695187 | 1.0252004 | 5.42E-77 | 2.33E-78 | up |
| VIT_09s0070g00140.t01 | 1588 | CAMK_KIN1/SNF1/Nim1_like.34 - CAMK includes calcium/calmodulin depedent | 285 | 12.119335 | 104 | 5.9540601 | 1.0253649 | 1.42E-10 | 3.34E-11 | up |
| VIT_17s0000g08290.t01 | 1358 | dof zinc finger domain containing protein, putative, expressed | 540 | 26.852111 | 197 | 13.188543 | 1.0257503 | 4.61E-19 | 6.86E-20 | up |
| VIT_18s0041g00950.t01 | 1533 | UDP-glucoronosyl and UDP-glucosyl transferase domain containing protein, express | 340 | 14.976875 | 124 | 7.3537676 | 1.0261811 | 2.06E-12 | 4.26E-13 | up |
| VIT_00s0333g00060.t01 | 993 | Putative uncharacterized protein | 85 | 5.7803498 | 31 | 2.8381988 | 1.0261811 | 0.00062795 | 0.0002907 | up |
| VIT_03s0063g01910.t01 | 635 | CASP-like protein | 85 | 9.0391927 | 31 | 4.4383172 | 1.0261811 | 0.00062795 | 0.0002907 | up |
| VIT_04s0023g01430.t01 | 2357 | zinc finger protein NUTCRACKER | 2216 | 63.488434 | 808 | 31.166078 | 1.0265172 | 4.12E-75 | 1.81E-76 | up |
| VIT_17s0000g00850.t01 | 1079 | FRIGIDA-like protein 2 | 129 | 8.0733301 | 47 | 3.9601058 | 1.0276249 | 2.09E-05 | 7.86E-06 | up |
| VIT_04s0079g00810.t01 | 806 | Putative uncharacterized protein | 656 | 54.960825 | 239 | 26.958345 | 1.0276717 | 5.39E-23 | 6.89E-24 | up |
| VIT_01s0011g03190.t01 | 660 | ATP-dependent Clp protease adaptor protein ClpS containing protein, expressed | 173 | 17.700544 | 63 | 8.6781467 | 1.0283348 | 7.01E-07 | 2.24E-07 | up |
| VIT_18s0001g10810.t01 | 1309 | probable carboxylesterase 2 | 228 | 11.761959 | 83 | 5.7645952 | 1.0288371 | 1.01E-08 | 2.73E-09 | up |
| VIT_13s0064g01110.t01 | 453 | Putative uncharacterized protein | 717 | 106.8822 | 261 | 52.380866 | 1.0289098 | 4.42E-25 | 5.25E-26 | up |
| VIT_18s0122g01420.t01 | 380 | mitochondrial genome | 503 | 89.385862 | 183 | 43.782229 | 1.0297013 | 6.14E-18 | 9.62E-19 | up |
| VIT_17s0000g05960.t01 | 764 | putative pectinesterase/pectinesterase inhibitor 26 | 143 | 12.639419 | 52 | 6.1878583 | 1.0304181 | 6.76E-06 | 2.41E-06 | up |
| VIT_11s0016g02480.t01 | 1209 | inorganic pyrophosphatase 3-like | 99 | 5.5295952 | 36 | 2.7071142 | 1.0304181 | 0.00020398 | 8.72E-05 | up |
| VIT_01s0150g00570.t01 | 295 | NADH dehydrogenase [ubiquinone] 1 alpha subcomplex subunit 2-like | 429 | 98.201861 | 156 | 48.076512 | 1.0304181 | 1.79E-15 | 3.14E-16 | up |
| VIT_03s0017g02240.t01 | 1109 | proline rich protein 1 (VPRP1) | 110 | 6.6980068 | 40 | 3.2791314 | 1.0304181 | 8.68E-05 | 3.53E-05 | up |
| VIT_07s0031g02950.t01 | 1534 | ammonium transporter protein, putative, expressed | 520 | 22.890877 | 189 | 11.201258 | 1.0311119 | 1.51E-18 | 2.31E-19 | up |
| VIT_00s0684g00030.t01 | 1073 | rhodanese-like domain containing protein, putative, expressed | 732 | 46.067623 | 266 | 22.53784 | 1.0314039 | 1.11E-25 | 1.29E-26 | up |
| VIT_04s0023g00600.t01 | 1124 | isopentenyl-diphosphate isomerase | 3234 | 194.29344 | 1175 | 95.039015 | 1.0316454 | 4.24E-110 | 1.29E-111 | up |
| VIT_11s0016g01720.t01 | 1728 | threonine synthase, chloroplast precursor, putative, expressed | 402 | 15.709659 | 146 | 7.6813842 | 1.0322136 | 1.31E-14 | 2.41E-15 | up |
| VIT_05s0020g04040.t01 | 1206 | chlorophyllase | 1173 | 65.680303 | 426 | 32.113871 | 1.0322642 | 1.38E-40 | 1.09E-41 | up |
| VIT_15s0046g01400.t01 | 577 | ubiquitin-conjugating enzyme, putative, expressed | 2452 | 286.96511 | 890 | 140.23117 | 1.0330682 | 7.04E-84 | 2.80E-85 | up |
| VIT_04s0008g03050.t01 | 1280 | amino acid kinase, putative, expressed | 1433 | 75.599803 | 520 | 36.933779 | 1.0334416 | 1.88E-49 | 1.25E-50 | up |
| VIT_09s0002g07620.t01 | 975 | dehydrogenase, putative, expressed | 102 | 7.0644767 | 37 | 3.4500666 | 1.0339585 | 0.0001541 | 6.50E-05 | up |
| VIT_14s0128g00770.t01 | 1115 | F-box and other domain containing protein, expressed | 295 | 17.866175 | 107 | 8.7244746 | 1.0340907 | 4.82E-11 | 1.10E-11 | up |
| VIT_02s0236g00040.t01 | 1345 | peptidyl-prolyl cis-trans isomerase, cyclophilin type, putative, expressed | 273 | 13.706444 | 99 | 6.6918051 | 1.034387 | 2.62E-10 | 6.30E-11 | up |
| VIT_08s0007g01660.t01 | 1646 | protein SSUH2 homolog | 789 | 32.369174 | 286 | 15.796707 | 1.0349967 | 9.49E-28 | 1.03E-28 | up |
| VIT_01s0026g00750.t01 | 704 | Putative uncharacterized protein | 538 | 51.605271 | 195 | 25.182122 | 1.0351186 | 2.88E-19 | 4.26E-20 | up |
| VIT_02s0025g02580.t01 | 1009 | mediator of RNA polymerase II transcription subunit 10, putative, expressed | 447 | 29.915812 | 162 | 14.596684 | 1.0352675 | 3.42E-16 | 5.77E-17 | up |
| VIT_12s0034g02200.t01 | 1419 | cysteine-rich receptor-like protein kinase 6 precursor, putative, expressed | 149 | 7.090687 | 54 | 3.4597263 | 1.0352675 | 3.89E-06 | 1.35E-06 | up |
| VIT_00s0287g00040.t01 | 935 | GATA zinc finger domain containing protein, expressed | 138 | 9.9667122 | 50 | 4.8617068 | 1.0356548 | 9.11E-06 | 3.29E-06 | up |
| VIT_02s0025g04180.t01 | 783 | Putative uncharacterized protein | 265 | 22.854333 | 96 | 11.146534 | 1.0358726 | 4.65E-10 | 1.14E-10 | up |
| VIT_05s0020g01370.t01 | 1033 | Putative uncharacterized protein | 105 | 6.8639391 | 38 | 3.3443648 | 1.0373045 | 0.00011732 | 4.84E-05 | up |
| VIT_02s0025g04560.t01 | 2404 | amine oxidase | 1012 | 28.426965 | 366 | 13.841304 | 1.0382802 | 1.74E-35 | 1.56E-36 | up |
| VIT_01s0150g00320.t01 | 1454 | nudix hydrolase 13 | 2367 | 109.93052 | 855 | 53.460385 | 1.0400499 | 6.80E-82 | 2.78E-83 | up |

| | | | | | | | | | | |
|---|---|---|---|---|---|---|---|---|---|---|
| VIT_07s0031g01730.t01 | 722 | iron-sulfur assembly protein IscA | 1509 | 141.13557 | 545 | 68.626157 | 1.0402512 | 1.43E-52 | 8.91E-54 | up |
| VIT_17s0000g05780.t01 | 1469 | Putative uncharacterized protein | 587 | 26.983653 | 212 | 13.12032 | 1.0402827 | 4.38E-21 | 6.00E-22 | up |
| VIT_18s0001g12130.t01 | 1747 | UDP-D-xylose:L-fucose alpha-1,3-D-xylosyltransferase 2 | 1053 | 40.70239 | 380 | 19.775208 | 1.0414206 | 4.65E-37 | 4.01E-38 | up |
| VIT_05s0020g04000.t01 | 809 | RING finger protein 126-like | 1312 | 109.51403 | 473 | 53.154862 | 1.0428421 | 5.03E-46 | 3.57E-47 | up |
| VIT_05s0049g00160.t01 | 916 | enoyl-CoA hydratase/isomerase family protein, putative, expressed | 677 | 49.908859 | 244 | 24.217244 | 1.0432612 | 2.91E-24 | 3.55E-25 | up |
| VIT_12s0134g00200.t01 | 1299 | methyltransferase domain containing protein, expressed | 161 | 8.3695319 | 58 | 4.0592819 | 1.0439224 | 1.29E-06 | 4.25E-07 | up |
| VIT_07s0141g00920.t01 | 1694 | cytochrome P450 94A1-like | 1361 | 54.253675 | 490 | 26.297414 | 1.0447999 | 7.53E-48 | 5.16E-49 | up |
| VIT_11s0016g03020.t01 | 1283 | pectinesterase, putative, expressed | 200 | 10.526592 | 72 | 5.1019502 | 1.0449177 | 5.85E-08 | 1.69E-08 | up |
| VIT_19s0015g01300.t01 | 1593 | amino acid transporter, putative, expressed | 514 | 21.788723 | 185 | 10.558113 | 1.0452296 | 9.85E-19 | 1.48E-19 | up |
| VIT_08s0007g05800.t01 | 2973 | phospholipase, patatin family, putative, expressed | 289 | 6.5642842 | 104 | 3.1803052 | 1.0454725 | 5.11E-11 | 1.17E-11 | up |
| VIT_14s0066g01930.t01 | 1223 | viral-response family protein, expressed | 264 | 14.576791 | 95 | 7.0619969 | 1.045525 | 3.66E-10 | 8.91E-11 | up |
| VIT_12s0134g00560.t01 | 1672 | uncharacterized GPI-anchored protein | 1537 | 62.075759 | 553 | 30.069017 | 1.0457523 | 5.56E-54 | 3.36E-55 | up |
| VIT_08s0040g00980.t01 | 1655 | tubulin beta-1 | 13620 | 555.72963 | 4899 | 269.11618 | 1.046154 | 0 | 0 | up |
| VIT_11s0016g03940.t01 | 1113 | heat stress transcription factor C-1 | 545 | 33.066314 | 196 | 16.009998 | 1.0463891 | 7.82E-20 | 1.13E-20 | up |
| VIT_05s0020g04420.t01 | 687 | calmodulin-like protein 11 | 153 | 15.039006 | 55 | 7.2784068 | 1.0470146 | 2.29E-06 | 7.72E-07 | up |
| VIT_05s0062g00300.t01 | 1593 | UDP-glycosyltransferase 75D1-like | 1088 | 46.120878 | 391 | 22.314715 | 1.0474246 | 1.31E-38 | 1.09E-39 | up |
| VIT_06s0004g08260.t01 | 688 | 60S ribosomal protein L10-like | 12494 | 1226.3022 | 4490 | 593.31903 | 1.0474346 | 0 | 0 | up |
| VIT_01s0011g02310.t01 | 1473 | Putative uncharacterized protein | 142 | 6.5098359 | 51 | 3.1477324 | 1.0483083 | 5.34E-06 | 1.88E-06 | up |
| VIT_03s0063g01340.t01 | 1173 | naringenin,2-oxoglutarate 3-dioxygenase | 142 | 8.1747556 | 51 | 3.952779 | 1.0483083 | 5.34E-06 | 1.88E-06 | up |
| VIT_14s0108g01570.t01 | 938 | Putative uncharacterized protein | 78 | 5.6153419 | 28 | 2.7138483 | 1.0490338 | 0.00086627 | 0.00040883 | up |
| VIT_08s0007g07900.t01 | 642 | Putative uncharacterized protein | 1126 | 118.43711 | 404 | 57.210627 | 1.0497661 | 4.59E-40 | 3.70E-41 | up |
| VIT_14s0066g02060.t01 | 1794 | ATP-dependent zinc metalloprotease FtsH-like | 1023 | 38.506819 | 367 | 18.598332 | 1.0499407 | 1.67E-36 | 1.46E-37 | up |
| VIT_11s0118g00650.t01 | 859 | Putative uncharacterized protein | 145 | 11.398804 | 52 | 5.50352 | 1.0504559 | 4.05E-06 | 1.41E-06 | up |
| VIT_01s0127g00380.t01 | 917 | pyrrolidone-carboxylate peptidase, putative, expressed | 792 | 58.323058 | 284 | 28.156546 | 1.050596 | 1.62E-28 | 1.72E-29 | up |
| VIT_11s0016g05400.t01 | 937 | Putative uncharacterized protein | 265 | 19.098125 | 95 | 9.2175263 | 1.0509795 | 2.83E-10 | 6.83E-11 | up |
| VIT_00s0347g00040.t01 | 436 | EF hand family protein, putative, expressed | 678 | 105.00927 | 243 | 50.669913 | 1.0513155 | 1.37E-24 | 1.65E-25 | up |
| VIT_07s0141g00730.t01 | 1239 | histone H1 | 3552 | 193.59141 | 1273 | 93.408731 | 1.0513857 | 1.25E-124 | 3.37E-126 | up |
| VIT_00s0455g00010.t01 | 607 | ras-related protein, putative, expressed | 190 | 21.137292 | 68 | 10.184755 | 1.0533793 | 1.06E-07 | 3.12E-08 | up |
| VIT_17s0000g07580.t01 | 1140 | response regulator receiver domain containing protein, expressed | 123 | 7.2859251 | 44 | 3.5089582 | 1.0540694 | 2.21E-05 | 8.36E-06 | up |
| VIT_02s0012g02090.t01 | 973 | Putative uncharacterized protein | 260 | 18.044504 | 93 | 8.6896139 | 1.0541955 | 3.80E-10 | 9.25E-11 | up |
| VIT_08s0007g01470.t01 | 844 | Copper transporter(CTR2) | 439 | 35.124206 | 157 | 16.911712 | 1.0544429 | 2.24E-16 | 3.76E-17 | up |
| VIT_04s0023g03540.t01 | 1372 | thaumatin-like protein | 193 | 9.4992133 | 69 | 4.5722014 | 1.0549191 | 8.01E-08 | 2.34E-08 | up |
| VIT_11s0118g00550.t01 | 1006 | Putative uncharacterized protein | 168 | 11.277056 | 60 | 5.4223012 | 1.0564133 | 5.74E-07 | 1.82E-07 | up |
| VIT_08s0007g04780.t01 | 1014 | aquaporin protein, putative, expressed | 6477 | 431.34065 | 2313 | 207.38056 | 1.056547 | 1.97E-228 | 2.75E-230 | up |
| VIT_11s0037g01130.t01 | 1085 | tetraspanin family protein, putative, expressed | 37365 | 2325.5179 | 13342 | 1117.9479 | 1.0566991 | 0 | 0 | up |
| VIT_15s0046g01510.t01 | 1847 | E1-BTB2 - Bric-a-Brac, Tramtrack, and Broad Complex domain with E1 subfamily | 2436 | 89.06249 | 869 | 42.774334 | 1.0580726 | 1.25E-86 | 4.83E-88 | up |
| VIT_13s0019g03430.t01 | 1468 | transferase family protein, putative, expressed | 286 | 13.156017 | 102 | 6.3169071 | 1.0584325 | 4.09E-11 | 9.28E-12 | up |
| VIT_04s0023g03470.t01 | 910 | chaperone protein dnaJ 11 | 735 | 54.541916 | 262 | 26.175216 | 1.0591639 | 6.99E-27 | 7.84E-28 | up |
| VIT_00s0477g00040.t01 | 1330 | clathrin assembly protein, putative, expressed | 559 | 28.382106 | 199 | 13.602909 | 1.0610664 | 9.32E-21 | 1.29E-21 | up |
| VIT_01s0011g04170.t01 | 1816 | lipid phosphatase protein, putative, expressed | 1006 | 37.40818 | 358 | 17.922457 | 1.0615853 | 1.51E-36 | 1.32E-37 | up |
| VIT_18s0001g09340.t01 | 1306 | acyl-CoA-binding domain-containing protein 3-like (ACBP) | 163 | 8.4280843 | 58 | 4.0375247 | 1.0617337 | 7.70E-07 | 2.47E-07 | up |
| VIT_12s0055g01090.t01 | 1581 | Putative uncharacterized protein | 1473 | 62.915162 | 524 | 30.132127 | 1.0621052 | 4.43E-53 | 2.72E-54 | up |
| VIT_06s0004g06390.t01 | 1892 | UDP-glucoronosyl and UDP-glucosyl transferase domain containing protein, express | 1316 | 46.969853 | 468 | 22.488221 | 1.0625656 | 1.45E-47 | 9.95E-49 | up |
| VIT_16s0100g00520.t01 | 1265 | F-box domain containing protein, expressed | 633 | 33.790734 | 225 | 16.17046 | 1.063267 | 1.94E-23 | 2.44E-24 | up |
| VIT_17s0000g07400.t01 | 2102 | lipase, putative, expressed | 498 | 15.998567 | 177 | 7.6554536 | 1.0633829 | 1.13E-18 | 1.71E-19 | up |
| VIT_03s0132g00200.t01 | 1899 | aldehyde dehydrogenase, putative, expressed | 668 | 23.753956 | 237 | 11.346287 | 1.0659476 | 8.99E-25 | 1.08E-25 | up |
| VIT_08s0007g01030.t01 | 1014 | oxidoreductase, aldo/keto reductase family protein, putative, expressed | 877 | 58.404469 | 311 | 27.883854 | 1.0666488 | 3.12E-32 | 3.02E-33 | up |
| VIT_14s0108g00970.t01 | 1237 | pentatricopeptide repeat-containing protein, putative, expressed | 392 | 21.399361 | 139 | 10.215873 | 1.0667553 | 5.29E-15 | 9.56E-16 | up |
| VIT_11s0016g04000.t01 | 711 | Putative uncharacterized protein | 395 | 37.515604 | 140 | 17.901475 | 1.0674123 | 4.00E-15 | 7.20E-16 | up |
| VIT_08s0058g00200.t01 | 1318 | transcription factor like protein, putative, expressed | 999 | 51.184035 | 354 | 24.418457 | 1.0677218 | 1.24E-36 | 1.08E-37 | up |
| VIT_01s0011g03420.t01 | 821 | Putative uncharacterized protein | 5019 | 412.81786 | 1778 | 196.88788 | 1.0681311 | 2.37E-180 | 4.42E-182 | up |
| VIT_14s0068g01840.t01 | 711 | Putative uncharacterized protein | 96 | 9.1177163 | 34 | 4.347501 | 1.0684862 | 0.00016292 | 6.88E-05 | up |
| VIT_18s0001g12070.t01 | 739 | psbM; photosystem II PsbM protein | 2850 | 260.42631 | 1009 | 124.1301 | 1.0690223 | 6.67E-103 | 2.19E-104 | up |
| VIT_19s0014g00960.t01 | 934 | ras-related protein, putative, expressed | 82 | 5.92859 | 29 | 2.822809 | 1.0705575 | 0.00050411 | 0.00022925 | up |
| VIT_08s0007g08490.t01 | 3361 | insulin-degrading enzyme, putative, expressed | 354 | 7.1124495 | 125 | 3.3812079 | 1.0728078 | 9.09E-14 | 1.74E-14 | up |
| VIT_03s0180g00260.t01 | 1268 | cinnamyl alcohol dehydrogenase 1 | 1759 | 93.67658 | 621 | 44.524876 | 1.0730768 | 3.01E-64 | 1.54E-65 | up |
| VIT_01s0010g03750.t01 | 575 | membrane protein, putative, expressed | 1179 | 138.46194 | 416 | 65.774243 | 1.0738948 | 1.90E-43 | 1.42E-44 | up |
| VIT_00s0194g00160.t01 | 858 | Putative uncharacterized protein | 1128 | 88.778184 | 398 | 42.17219 | 1.0739132 | 1.30E-41 | 1.01E-42 | up |
| VIT_13s0074g00750.t01 | 706 | 60S acidic ribosomal protein, putative, expressed | 411 | 39.311676 | 145 | 18.672122 | 1.074072 | 7.73E-16 | 1.33E-16 | up |
| VIT_07s0141g00570.t01 | 578 | psbW; photosystem II PsbW protein | 82926 | 9688.2943 | 29251 | 4600.9049 | 1.0743251 | 0 | 0 | up |
| VIT_11s0206g00070.t01 | 2082 | calmodulin binding protein, putative, expressed | 190 | 6.1625055 | 67 | 2.925664 | 1.0747529 | 6.36E-08 | 1.84E-08 | up |
| VIT_01s0026g02540.t01 | 820 | zinc finger, C3HC4 type domain containing protein, expressed | 2005 | 165.11441 | 707 | 78.385536 | 1.0748066 | 2.70E-73 | 1.23E-74 | up |
| VIT_18s0001g06760.t01 | 1341 | clathrin assembly protein, putative, expressed | 122 | 6.1434948 | 43 | 2.9152114 | 1.0754591 | 1.73E-05 | 6.46E-06 | up |
| VIT_14s0006g01600.t01 | 720 | Putative uncharacterized protein | 701 | 65.746095 | 247 | 31.188525 | 1.0758899 | 2.53E-26 | 2.88E-27 | up |
| VIT_00s0218g00010.t01 | 1160 | dehydrogenase, putative, expressed | 298 | 17.347733 | 105 | 8.229277 | 1.0759095 | 8.14E-12 | 1.76E-12 | up |
| VIT_12s0057g01540.t01 | 1460 | Regulator of chromosome condensation domain containing protein, expressed | 159 | 7.3540861 | 56 | 3.4871092 | 1.0765145 | 7.94E-07 | 2.55E-07 | up |
| VIT_03s0063g01770.t01 | 405 | Putative uncharacterized protein | 1397 | 232.93021 | 492 | 110.44357 | 1.0765883 | 1.69E-51 | 1.08E-52 | up |
| VIT_06s0009g01740.t01 | 1564 | FAD binding domain containing protein, expressed | 233 | 10.060131 | 82 | 4.7665865 | 1.0776206 | 1.67E-09 | 4.27E-10 | up |
| VIT_01s0026g01300.t01 | 2517 | HIPL1 protein-like | 884 | 23.716658 | 311 | 11.233305 | 1.0781183 | 4.92E-33 | 4.66E-34 | up |
| VIT_06s0004g00940.t01 | 825 | Putative uncharacterized protein | 1263 | 103.37936 | 444 | 48.928217 | 1.0792096 | 7.76E-47 | 5.42E-48 | up |
| VIT_00s0301g00080.t01 | 455 | internal alternative NAD(P)H-ubiquinone oxidoreductase A1 | 427 | 63.372512 | 150 | 29.971621 | 1.0802601 | 1.48E-16 | 2.46E-17 | up |
| VIT_01s0011g06620.t01 | 2053 | peptide transporter PTR2, putative, expressed | 1629 | 53.581711 | 572 | 25.330132 | 1.0808861 | 2.88E-60 | 1.57E-61 | up |
| VIT_04s0008g01330.t01 | 907 | green ripe-like, putative, expressed | 77 | 5.7328144 | 27 | 2.706368 | 1.0828855 | 0.00067529 | 0.00031372 | up |
| VIT_04s0079g00800.t01 | 1430 | DJ-1 family protein, putative, expressed | 1044 | 49.300225 | 366 | 23.268877 | 1.0831927 | 4.05E-39 | 3.33E-40 | up |
| VIT_03s0038g04400.t01 | 2135 | zinc finger, C3HC4 type domain containing protein, expressed | 528 | 16.700154 | 185 | 7.8777868 | 1.0839992 | 2.51E-20 | 3.56E-21 | up |
| VIT_08s0007g06670.t01 | 1035 | homeobox associated leucine zipper, putative, expressed | 80 | 5.2195622 | 28 | 2.4595069 | 1.0855597 | 0.00051093 | 0.00023269 | up |
| VIT_04s0008g03690.t01 | 703 | protein PEROXIN-4-like | 320 | 30.738247 | 112 | 14.484152 | 1.0855597 | 9.04E-13 | 1.82E-13 | up |
| VIT_12s0028g03900.t01 | 430 | Putative uncharacterized protein | 386 | 60.618236 | 135 | 28.542742 | 1.0866279 | 3.30E-15 | 5.90E-16 | up |
| VIT_01s0026g01550.t01 | 1343 | homeobox-leucine zipper protein HAT5-like | 123 | 6.1846274 | 43 | 2.91087 | 1.0872363 | 1.33E-05 | 4.89E-06 | up |
| VIT_12s0057g00990.t01 | 1973 | alpha-1,6-mannosyl-glycoprotein 2-beta-N-acetylglucosaminyltransferase-like | 535 | 18.310961 | 187 | 8.6167778 | 1.0874871 | 1.11E-20 | 1.54E-21 | up |
| VIT_18s0001g00490.t01 | 1264 | enoyl-CoA hydratase 2 | 355 | 18.965562 | 124 | 8.9187704 | 1.0884654 | 4.16E-14 | 7.84E-15 | up |
| VIT_01s0137g00650.t01 | 729 | nitrilase-associated protein, putative, expressed | 564 | 52.243952 | 197 | 24.567959 | 1.088486 | 8.94E-22 | 1.19E-22 | up |
| VIT_07s0031g00920.t01 | 1934 | inositol-3-phosphate synthase-like | 22442 | 783.59117 | 7838 | 368.45051 | 1.0886303 | 0 | 0 | up |
| VIT_07s0141g00040.t01 | 1498 | probable protein phosphatase 2C 10-like | 169 | 7.6183222 | 59 | 3.5807217 | 1.0892229 | 2.65E-07 | 8.13E-08 | up |
| VIT_19s0015g00380.t01 | 1491 | cysteine synthase 2-like | 129 | 5.8424703 | 45 | 2.7438808 | 1.0903607 | 7.61E-06 | 2.72E-06 | up |
| VIT_12s0057g00210.t01 | 665 | Putative uncharacterized protein | 172 | 17.465911 | 60 | 8.2027595 | 1.0903607 | 2.01E-07 | 6.09E-08 | up |
| VIT_04s0008g00720.t01 | 1796 | vacuolar-processing enzyme-like | 4280 | 160.92439 | 1491 | 75.47475 | 1.092317 | 1.41E-159 | 2.93E-161 | up |
| VIT_14s0068g01860.t01 | 545 | Putative uncharacterized protein | 112 | 13.877332 | 39 | 6.5057666 | 1.0929392 | 3.10E-05 | 1.19E-05 | up |
| VIT_10s0042g00070.t01 | 979 | proteasome subunit alpha type-4-like | 2198 | 151.61056 | 764 | 70.948144 | 1.0955334 | 9.28E-83 | 3.75E-84 | up |
| VIT_13s0019g02260.t01 | 1874 | BTB/POZ and MATH domain-containing protein 3-like | 475 | 17.116244 | 165 | 8.0046939 | 1.096448 | 1.03E-18 | 1.55E-19 | up |
| VIT_15s0048g02530.t01 | 896 | protein NDR1-like | 360 | 27.13182 | 125 | 12.683303 | 1.0970553 | 1.84E-14 | 3.41E-15 | up |
| VIT_12s0028g01260.t01 | 1041 | V-type proton ATPase subunit D-like | 2232 | 144.78644 | 775 | 67.683272 | 1.0970553 | 3.29E-84 | 1.30E-85 | up |
| VIT_00s0301g00180.t01 | 1053 | quinone oxidoreductase PIG3-like | 242 | 15.519275 | 84 | 7.2523923 | 1.0975323 | 4.33E-10 | 1.06E-10 | up |
| VIT_05s0094g00090.t01 | 879 | pentatricopeptide repeat-containing protein | 291 | 22.355715 | 101 | 10.446309 | 1.0976504 | 6.58E-12 | 1.41E-12 | up |
| VIT_16s0115g00340.t01 | 1638 | UDP-glucoronosyl and UDP-glucosyl transferase domain containing protein, express | 124 | 5.1120163 | 43 | 2.3866291 | 1.0989181 | 1.02E-05 | 3.69E-06 | up |
| VIT_07s0005g02130.t01 | 2066 | Putative uncharacterized protein | 7825 | 255.76344 | 2713 | 119.38502 | 1.0991882 | 3.68E-294 | 4.18E-296 | up |
| VIT_14s0060g01490.t01 | 758 | dnaJ homolog subfamily B member 3 | 8711 | 776.03847 | 3020 | 362.2164 | 1.0992763 | 0 | 0 | up |

| | | | | | | | | | | |
|---|---|---|---|---|---|---|---|---|---|---|
| VIT_11s0016g05520.t01 | 667 | umecyanin-like | 3034 | 307.16674 | 1051 | 143.25416 | 1.1004449 | 1.02E-114 | 2.97E-116 | up |
| VIT_13s0156g00220.t01 | 1647 | PRLI-interacting factor A, putative, expressed | 1377 | 56.457908 | 477 | 26.33026 | 1.1004539 | 1.41E-52 | 8.79E-54 | up |
| VIT_14s0108g01460.t01 | 681 | EID1-like F-box protein 2-like | 644 | 63.85916 | 223 | 29.770637 | 1.1010035 | 3.73E-25 | 4.41E-26 | up |
| VIT_11s0016g04230.t01 | 721 | Putative uncharacterized protein | 1843 | 172.6134 | 638 | 80.448099 | 1.1014143 | 3.83E-70 | 1.83E-71 | up |
| VIT_17s0000g09470.t01 | 956 | high-affinity nitrate transporter 3.2-like | 2081 | 146.99367 | 720 | 68.470733 | 1.1021947 | 3.66E-79 | 1.53E-80 | up |
| VIT_03s0063g01930.t01 | 740 | Putative uncharacterized protein | 370 | 33.764043 | 128 | 15.725651 | 1.102368 | 6.09E-15 | 1.10E-15 | up |
| VIT_00s0912g00010.t01 | 645 | Putative uncharacterized protein | 402 | 42.087272 | 139 | 19.592302 | 1.1030971 | 3.75E-16 | 6.35E-17 | up |
| VIT_13s0147g00110.t01 | 631 | petF; ferredoxin | 810 | 86.684231 | 280 | 40.34215 | 1.1034816 | 1.76E-31 | 1.73E-32 | up |
| VIT_11s0037g00810.t01 | 2068 | Putative uncharacterized protein | 1065 | 34.776311 | 368 | 16.178105 | 1.1040623 | 4.19E-41 | 3.28E-42 | up |
| VIT_03s0063g00130.t01 | 672 | 40S ribosomal protein S16-like | 18931 | 1902.3426 | 6539 | 884.65195 | 1.1045952 | 0 | 0 | up |
| VIT_08s0007g02560.t01 | 1014 | transcription repressor OFP13 | 1022 | 68.060852 | 353 | 31.64952 | 1.1046416 | 1.60E-39 | 1.31E-40 | up |
| VIT_11s0016g04750.t01 | 2712 | potassium channel AKT1-like | 249 | 6.2000345 | 86 | 2.8829635 | 1.1047237 | 1.90E-10 | 4.51E-11 | up |
| VIT_01s0010g03830.t01 | 652 | Putative uncharacterized protein | 2603 | 269.59449 | 899 | 125.35523 | 1.1047688 | 3.60E-99 | 1.22E-100 | up |
| VIT_15s0046g03650.t01 | 995 | short-chain dehydrogenase/reductase, putative, expressed | 139 | 9.4335719 | 48 | 4.385797 | 1.1049651 | 2.53E-06 | 8.58E-07 | up |
| VIT_03s0063g01730.t01 | 1436 | plant viral response family protein, putative, expressed | 307 | 14.436715 | 106 | 6.7109159 | 1.1051609 | 1.26E-12 | 2.57E-13 | up |
| VIT_18s0001g08750.t01 | 568 | Putative uncharacterized protein | 113 | 13.434285 | 39 | 6.2423289 | 1.1057633 | 2.37E-05 | 8.99E-06 | up |
| VIT_15s0021g02620.t01 | 480 | protein transport protein Sec61 subunit beta, putative, expressed | 757 | 106.49742 | 261 | 49.434443 | 1.10723 | 1.21E-29 | 1.25E-30 | up |
| VIT_04s0023g00590.t01 | 758 | regulator of ribonuclease-like protein 2 | 3803 | 338.79857 | 1311 | 157.2403 | 1.1074568 | 4.68E-145 | 1.07E-146 | up |
| VIT_02s0025g04880.t01 | 1485 | ripening-related P-450 enzyme-like (GFH2) | 1117 | 50.793854 | 385 | 23.570275 | 1.1076853 | 2.75E-43 | 2.06E-44 | up |
| VIT_11s0206g00030.t01 | 1136 | chitotriosidase-1 | 206 | 12.24541 | 71 | 5.6821199 | 1.1077399 | 7.18E-09 | 1.93E-09 | up |
| VIT_13s0147g00230.t01 | 1505 | proteins of unknown function domain containing protein, putative, expressed | 354 | 15.883683 | 122 | 7.3697661 | 1.1078547 | 1.90E-14 | 3.52E-15 | up |
| VIT_14s0006g02310.t01 | 471 | Putative uncharacterized protein | 740 | 106.09508 | 255 | 49.220911 | 1.1080145 | 4.93E-29 | 5.17E-30 | up |
| VIT_17s0000g08830.t01 | 895 | heme-binding protein 2-like | 180 | 13.581068 | 62 | 6.2979474 | 1.1086433 | 6.75E-08 | 1.96E-08 | up |
| VIT_01s0011g03690.t01 | 713 | niversal stress protein A-like protein | 871 | 82.492235 | 300 | 38.2527 | 1.1086967 | 5.03E-34 | 4.63E-35 | up |
| VIT_13s0019g02700.t01 | 798 | Putative uncharacterized protein | 308 | 26.063472 | 106 | 12.076285 | 1.1098526 | 9.63E-13 | 1.95E-13 | up |
| VIT_12s0028g02140.t01 | 2427 | F-box domain and LRR containing protein, expressed | 1177 | 32.748479 | 405 | 15.171049 | 1.110107 | 1.02E-45 | 7.33E-47 | up |
| VIT_11s0016g03210.t01 | 2230 | malate dehydrogenase (NADP+) (NADP-ME) | 5516 | 167.0336 | 1898 | 77.378751 | 1.110129 | 7.20E-211 | 1.10E-212 | up |
| VIT_00s0194g00210.t01 | 1231 | calcium-activated outward-rectifying potassium channel 1-like | 250 | 13.714071 | 86 | 6.3514191 | 1.110506 | 1.45E-10 | 3.42E-11 | up |
| VIT_19s0090g00580.t01 | 661 | autophagy-related protein 8C-like | 349 | 35.654012 | 120 | 16.504796 | 1.1111791 | 2.54E-14 | 4.72E-15 | up |
| VIT_18s0076g00350.t01 | 1298 | F-box domain containing protein, expressed | 192 | 9.9887462 | 66 | 4.6227416 | 1.1115549 | 2.21E-08 | 6.16E-09 | up |
| VIT_05s0020g01910.t01 | 1939 | inactive beta-amylase 9-like | 3308 | 115.20521 | 1137 | 53.310533 | 1.1117135 | 4.61E-127 | 1.21E-128 | up |
| VIT_18s0041g01040.t01 | 1561 | UDP-glucoronosyl and UDP-glucosyl transferase domain containing protein, express | 387 | 16.741428 | 133 | 7.7460289 | 1.1118938 | 8.88E-16 | 1.53E-16 | up |
| VIT_18s0001g13910.t01 | 655 | Putative uncharacterized protein | 713 | 73.507673 | 245 | 34.005969 | 1.1121068 | 3.58E-28 | 3.85E-29 | up |
| VIT_08s0007g06360.t01 | 1365 | Putative uncharacterized protein | 1188 | 58.771697 | 408 | 27.17427 | 1.1128803 | 2.57E-46 | 1.82E-47 | up |
| VIT_14s0060g00990.t01 | 839 | Werner Syndrome-like exonuclease-like | 364 | 29.297048 | 125 | 13.544982 | 1.1129969 | 6.26E-15 | 1.14E-15 | up |
| VIT_05s0020g01490.t01 | 723 | RALFL4 - Rapid ALkalinization Factor RALF family protein precursor, expressed | 1127 | 105.26162 | 387 | 48.663466 | 1.1130686 | 5.30E-44 | 3.93E-45 | up |
| VIT_14s0066g01870.t01 | 1635 | U-box domain-containing protein 40-like | 440 | 18.172696 | 151 | 8.3963312 | 1.1139415 | 7.68E-18 | 1.21E-18 | up |
| VIT_08s0058g01030.t01 | 1482 | saposin-like type B, region 1 family protein, putative, expressed | 3647 | 166.17742 | 1251 | 76.743125 | 1.1146149 | 1.39E-140 | 3.26E-142 | up |
| VIT_17s0000g08690.t01 | 1348 | WD repeat-containing protein 85-like | 242 | 12.122995 | 83 | 5.5978154 | 1.1148103 | 2.55E-10 | 6.12E-11 | up |
| VIT_07s0005g00660.t01 | 547 | indole-3-acetic acid-induced protein ARG2-like | 4159 | 513.43567 | 1425 | 236.84156 | 1.1162613 | 1.35E-160 | 2.80E-162 | up |
| VIT_07s0005g02820.t01 | 1362 | cathepsin B-like | 4381 | 217.21039 | 1499 | 100.05871 | 1.1182463 | 1.34E-169 | 2.56E-171 | up |
| VIT_19s0135g00070.t01 | 806 | 60S ribosomal protein L18a-1-like | 497 | 41.639527 | 170 | 19.175392 | 1.1186976 | 3.83E-20 | 5.46E-21 | up |
| VIT_18s0001g12550.t01 | 1708 | 3-ketoacyl-CoA synthase 4-like | 1225 | 48.432029 | 419 | 22.302653 | 1.1187461 | 4.01E-48 | 2.72E-49 | up |
| VIT_18s0001g11630.t01 | 1777 | allene oxide synthase(AOS) | 1801 | 68.440115 | 616 | 31.51546 | 1.1187824 | 2.69E-70 | 1.28E-71 | up |
| VIT_08s0007g06960.t01 | 687 | dirigent protein 2 | 345 | 33.911484 | 118 | 15.615491 | 1.118796 | 2.57E-14 | 4.80E-15 | up |
| VIT_02s0025g04580.t01 | 1696 | hydroxymethylglutaryl-CoA synthase / HMG-CoA synthase / 3-hydroxy-3-methylglu | 1680 | 66.891029 | 574 | 30.769215 | 1.1203251 | 8.88E-66 | 4.48E-67 | up |
| VIT_13s0139g00260.t01 | 994 | F-box protein SKIP5-like | 249 | 16.915989 | 85 | 7.774329 | 1.1215975 | 1.12E-10 | 2.61E-11 | up |
| VIT_19s0014g01190.t01 | 1583 | alpha-1,3/1,6-mannosyltransferase ALG2-like | 176 | 7.5078605 | 60 | 3.4458844 | 1.1235275 | 6.85E-08 | 1.99E-08 | up |
| VIT_00s2439g00010.t01 | 667 | DHHC zinc finger domain containing protein, expressed | 88 | 8.9092528 | 30 | 4.0890818 | 1.1235275 | 0.00017033 | 7.22E-05 | up |
| VIT_00s0698g00010.t01 | 1763 | catalase | 16857 | 645.67269 | 5743 | 296.1535 | 1.1244578 | 0 | 0 | up |
| VIT_16s0098g01110.t01 | 755 | GRF-interacting factor 2, putative, expressed | 91 | 8.1391468 | 31 | 3.7328893 | 1.1245848 | 0.00012887 | 5.36E-05 | up |
| VIT_13s0156g00190.t01 | 255 | Putative uncharacterized protein | 364 | 96.393033 | 124 | 44.209121 | 1.1245848 | 3.66E-15 | 6.56E-16 | up |
| VIT_08s0040g02720.t01 | 838 | fatty acid desaturase, putative, expressed | 364 | 29.332009 | 124 | 13.452656 | 1.1245848 | 3.66E-15 | 6.56E-16 | up |
| VIT_14s0068g01940.t01 | 561 | protein transport protein Sec61 subunit gamma-like | 2540 | 305.74214 | 865 | 140.17921 | 1.125043 | 1.20E-99 | 4.06E-101 | up |
| VIT_06s0004g05560.t01 | 643 | transcription elongation factor B polypeptide 1-like | 1031 | 108.27598 | 351 | 49.627971 | 1.1254879 | 4.78E-41 | 3.75E-42 | up |
| VIT_01s0026g00170.t01 | 1534 | equilibrative nucleoside transporter 4-like | 1475 | 64.930852 | 502 | 29.751491 | 1.1259422 | 2.51E-58 | 1.41E-59 | up |
| VIT_12s0059g01640.t01 | 1209 | Putative uncharacterized protein | 288 | 16.086095 | 98 | 7.3693664 | 1.1262017 | 3.01E-12 | 6.29E-13 | up |
| VIT_09s0002g07220.t01 | 657 | Putative uncharacterized protein | 147 | 15.109024 | 50 | 6.9188674 | 1.1268027 | 8.43E-07 | 2.72E-07 | up |
| VIT_14s0066g02560.t01 | 631 | thioredoxin | 2528 | 270.54042 | 859 | 123.76395 | 1.1282529 | 1.24E-99 | 4.21E-101 | up |
| VIT_05s0049g00570.t01 | 1242 | early nodulin-75-like | 842 | 45.77991 | 286 | 20.935089 | 1.1287916 | 7.27E-34 | 6.75E-35 | up |
| VIT_09s0002g03540.t01 | 1008 | myb-related transcription activator, putative, expressed | 639 | 42.807983 | 217 | 19.571746 | 1.1291074 | 5.84E-26 | 6.73E-27 | up |
| VIT_11s0016g00920.t01 | 767 | Copper transporter (CTR1) | 878 | 77.30073 | 298 | 35.322487 | 1.1298951 | 2.52E-35 | 2.27E-36 | up |
| VIT_08s0007g07950.t01 | 931 | zinc finger A20 and AN1 domain-containing stress-associated protein 8-like | 3312 | 240.22881 | 1124 | 109.76073 | 1.1300471 | 1.38E-130 | 3.48E-132 | up |
| VIT_16s0050g01300.t01 | 1092 | C2 domain containing protein, putative, expressed | 451 | 27.889347 | 153 | 12.737939 | 1.1305823 | 1.13E-18 | 1.70E-19 | up |
| VIT_00s0225g00110.t01 | 1372 | F-box protein | 395 | 19.441395 | 134 | 8.8793476 | 1.1306062 | 1.72E-16 | 2.87E-17 | up |
| VIT_07s0104g01800.t01 | 928 | glutathione S-transferase | 1925 | 140.07712 | 653 | 63.972832 | 1.1306901 | 2.44E-76 | 1.06E-77 | up |
| VIT_07s0151g00830.t01 | 742 | Putative uncharacterized protein | 121 | 11.011993 | 41 | 5.0235453 | 1.1322977 | 7.88E-06 | 2.82E-06 | up |
| VIT_07s0130g00320.t01 | 1102 | probable carboxylesterase 18 | 366 | 22.427658 | 124 | 10.229878 | 1.13249 | 2.13E-15 | 3.75E-16 | up |
| VIT_16s0022g00450.t01 | 1023 | cell number regulator 6-like | 1048 | 69.178333 | 355 | 31.548818 | 1.1327343 | 3.96E-42 | 3.03E-43 | up |
| VIT_16s0050g00710.t01 | 994 | Putative uncharacterized protein | 260 | 17.663282 | 88 | 8.0487171 | 1.1339227 | 2.80E-11 | 6.28E-12 | up |
| VIT_13s0067g02000.t01 | 1118 | Putative uncharacterized protein | 133 | 8.0333055 | 45 | 3.6593258 | 1.1344158 | 2.58E-06 | 8.77E-07 | up |
| VIT_05s0077g00190.t01 | 441 | EF hand family protein, putative, expressed | 68 | 10.412494 | 23 | 4.7415422 | 1.1348874 | 0.00091932 | 0.00043589 | up |
| VIT_17s0000g02020.t01 | 562 | erwinia induced protein 2, putative, expressed | 2540 | 305.19811 | 859 | 138.95917 | 1.135085 | 4.54E-101 | 1.52E-102 | up |
| VIT_09s0002g06400.t01 | 317 | Putative uncharacterized protein | 71 | 15.124587 | 24 | 6.8830726 | 1.1357711 | 0.00069355 | 0.00032272 | up |
| VIT_17s0000g04350.t01 | 764 | protein Dr1 homolog-like | 817 | 72.212626 | 276 | 32.843248 | 1.1366543 | 3.02E-33 | 2.84E-34 | up |
| VIT_06s0004g01790.t01 | 733 | zinc finger A20 and AN1 domain-containing stress-associated protein 5-like | 989 | 91.112248 | 334 | 41.425987 | 1.1371089 | 4.66E-40 | 3.75E-41 | up |
| VIT_00s0207g00010.t01 | 1560 | omega-hydroxypalmitate O-feruloyl transferase-like | 3234 | 139.99092 | 1092 | 63.639742 | 1.1373333 | 7.55E-129 | 1.94E-130 | up |
| VIT_03s0038g02500.t01 | 735 | SKP1-like protein 1A | 2189 | 201.11426 | 739 | 91.408687 | 1.1376122 | 1.57E-87 | 5.98E-89 | up |
| VIT_09s0002g07120.t01 | 1033 | adenine phosphoribosyltransferase 2-like | 163 | 10.655448 | 55 | 4.840528 | 1.1383549 | 1.60E-07 | 4.79E-08 | up |
| VIT_18s0001g01200.t01 | 348 | Putative uncharacterized protein | 590 | 114.48727 | 199 | 51.988131 | 1.138933 | 2.26E-24 | 2.74E-25 | up |
| VIT_01s0011g06200.t01 | 1186 | zinc finger protein 4-like | 442 | 25.166454 | 149 | 11.421732 | 1.1397205 | 1.50E-18 | 2.29E-19 | up |
| VIT_07s0031g02140.t01 | 1455 | transmembrane amino acid transporter protein, putative, expressed | 1095 | 50.820106 | 369 | 23.056519 | 1.1402247 | 1.96E-44 | 1.44E-45 | up |
| VIT_19s0015g00210.t01 | 1316 | zinc transporter 11 | 389 | 19.96081 | 131 | 9.0499416 | 1.1411899 | 1.74E-16 | 2.91E-17 | up |
| VIT_17s0000g04110.t01 | 523 | exosome complex exonuclease RRP46 | 202 | 26.081594 | 68 | 11.820548 | 1.1417352 | 4.24E-09 | 1.12E-09 | up |
| VIT_12s0034g01860.t01 | 1455 | probable 3-hydroxyisobutyrate dehydrogenase-like 2 | 10475 | 486.15581 | 3523 | 220.1304 | 1.1430604 | 0 | 0 | up |
| VIT_13s0067g00180.t01 | 1073 | hydroxyacylglutathione hydrolase cytoplasmic-like | 1473 | 92.701651 | 495 | 41.940717 | 1.1442435 | 9.41E-60 | 5.17E-61 | up |
| VIT_19s0015g00250.t01 | 1194 | zinc transporter 11-like | 131 | 7.4088604 | 44 | 3.3502616 | 1.1449779 | 2.59E-06 | 8.80E-07 | up |
| VIT_12s0057g01220.t01 | 1010 | transmembrane emp24 domain-containing protein p24beta2 | 542 | 36.237844 | 182 | 16.382508 | 1.1453409 | 1.14E-22 | 1.47E-23 | up |
| VIT_02s0087g00780.t01 | 833 | glycine-rich protein A3 | 6195 | 502.20468 | 2080 | 227.01194 | 1.1455073 | 5.75E-249 | 7.43E-251 | up |
| VIT_01s0010g03050.t01 | 520 | protein kish-A-like | 557 | 72.33297 | 187 | 32.694043 | 1.1456256 | 2.81E-23 | 3.55E-24 | up |
| VIT_10s0003g01410.t01 | 1736 | CBL-interacting protein kinase 16 (CIPK16) | 140 | 5.4458134 | 47 | 2.4613791 | 1.1456807 | 1.12E-06 | 3.67E-07 | up |
| VIT_06s0004g04250.t01 | 649 | probable histone H2B.1-like | 7064 | 735.00524 | 2371 | 332.13698 | 1.1459762 | 5.14E-284 | 6.12E-286 | up |
| VIT_14s0083g00250.t01 | 501 | Putative uncharacterized protein | 900 | 121.30794 | 302 | 54.802401 | 1.146363 | 5.21E-37 | 4.49E-38 | up |
| VIT_11s0016g00550.t01 | 478 | Putative uncharacterized protein | 304 | 42.946733 | 102 | 19.400041 | 1.1464887 | 3.29E-13 | 6.48E-14 | up |
| VIT_01s0127g00310.t01 | 1334 | putative F-box protein | 155 | 7.8462169 | 52 | 3.5438709 | 1.1466712 | 2.79E-07 | 8.58E-08 | up |
| VIT_14s0006g00450.t01 | 977 | myb-related transcription factor (Myb2) | 158 | 10.920612 | 53 | 4.9318707 | 1.1468468 | 2.11E-07 | 6.42E-08 | up |
| VIT_08s0217g00040.t01 | 2047 | importin-11-like | 161 | 5.3111978 | 54 | 2.3983154 | 1.1470159 | 1.60E-07 | 4.80E-08 | up |

| | | | | | | | | | | |
|---|---|---|---|---|---|---|---|---|---|---|
| VIT_12s0035g00380.t01 | 1811 | mitochondrial chaperone BCS1-like | 1265 | 47.168984 | 424 | 21.285202 | 1.1479877 | 1.01E-51 | 6.41E-53 | up |
| VIT_09s0070g00490.t01 | 1347 | probable protein phosphatase 2C 44-like | 221 | 11.079218 | 74 | 4.9945285 | 1.1494357 | 6.03E-10 | 1.49E-10 | up |
| VIT_17s0053g00990.t01 | 1286 | expansin-like | 5494 | 288.49091 | 1839 | 130.00832 | 1.1499219 | 3.19E-222 | 4.57E-224 | up |
| VIT_06s0004g00790.t01 | 1460 | amino acid permease family protein, putative, expressed | 583 | 26.964982 | 195 | 12.142612 | 1.1510083 | 1.73E-24 | 2.09E-25 | up |
| VIT_17s0000g03340.t01 | 3032 | probable serine/threonine-protein kinase | 326 | 7.2606056 | 109 | 3.2683433 | 1.1515303 | 3.52E-14 | 6.60E-15 | up |
| VIT_07s0031g00990.t01 | 1286 | trafficking protein particle complex subunit 12-like | 748 | 39.277612 | 250 | 17.673779 | 1.1520967 | 3.59E-31 | 3.57E-32 | up |
| VIT_06s0004g03100.t01 | 2504 | MLO13 protein (MLO13) | 1362 | 36.730533 | 455 | 16.519901 | 1.1527748 | 5.66E-56 | 3.31E-57 | up |
| VIT_14s0068g02060.t01 | 727 | enhancer of yellow 2 transcription factor homolog | 563 | 52.29479 | 188 | 23.510064 | 1.1533888 | 9.27E-24 | 1.15E-24 | up |
| VIT_01s0011g04210.t01 | 1633 | amino acid permease family protein, putative, expressed | 584 | 24.149665 | 195 | 10.856224 | 1.1534808 | 1.31E-24 | 1.58E-25 | up |
| VIT_09s0002g05180.t01 | 397 | Putative uncharacterized protein | 16094 | 2737.524 | 5366 | 1228.8264 | 1.1555905 | 0 | 0 | up |
| VIT_00s0174g00130.t01 | 812 | FAR1 family protein, expressed | 81 | 6.7361761 | 27 | 3.0229997 | 1.155949 | 0.00022705 | 9.78E-05 | up |
| VIT_04s0023g00160.t01 | 1172 | haloacid dehalogenase-like hydrolase family protein, putative, expressed | 135 | 7.7784059 | 45 | 3.4907221 | 1.155949 | 1.48E-06 | 4.92E-07 | up |
| VIT_12s0059g02140.t01 | 1539 | F-box protein | 225 | 9.8725273 | 75 | 4.4305028 | 1.155949 | 3.46E-10 | 8.41E-11 | up |
| VIT_03s0091g00520.t01 | 1241 | prolyl 4-hydroxylase subunit alpha-1-like | 102 | 5.5502537 | 34 | 2.4907923 | 1.155949 | 3.20E-05 | 1.23E-05 | up |
| VIT_11s0016g04510.t01 | 326 | serine/threonine protein phosphatase 2A 57 kDa regulatory subunit B' iota isoform | 114 | 23.614116 | 38 | 10.597328 | 1.155949 | 1.04E-05 | 3.80E-06 | up |
| VIT_12s0028g02440.t01 | 1326 | protein trichome birefringence-like 39 | 1932 | 98.389339 | 644 | 44.154271 | 1.155949 | 2.42E-79 | 1.01E-80 | up |
| VIT_04s0008g03210.t01 | 669 | lachrymatory-factor synthase-like | 162 | 16.352093 | 54 | 7.3383431 | 1.155949 | 1.21E-07 | 3.60E-08 | up |
| VIT_04s0044g00720.t01 | 2426 | G-type lectin S-receptor-like serine/threonine-protein kinase RLK1-like | 288 | 8.0165247 | 96 | 3.5975829 | 1.155949 | 1.01E-12 | 2.04E-13 | up |
| VIT_14s0036g00340.t01 | 240 | serine/threonine-protein phosphatase PP1-like | 114 | 32.075841 | 38 | 14.394704 | 1.155949 | 1.04E-05 | 3.80E-06 | up |
| VIT_12s0059g01260.t01 | 1964 | probable plastidic glucose transporter 1-like | 303 | 10.41803 | 101 | 4.6753084 | 1.155949 | 2.50E-13 | 4.88E-14 | up |
| VIT_05s0077g01980.t01 | 1060 | Putative uncharacterized protein | 273 | 17.391668 | 91 | 7.8048741 | 1.155949 | 4.05E-12 | 8.54E-13 | up |
| VIT_00s0203g00150.t01 | 668 | Putative uncharacterized protein | 138 | 13.950413 | 46 | 6.2605392 | 1.155949 | 1.12E-06 | 3.67E-07 | up |
| VIT_00s0264g00120.t01 | 1115 | Putative uncharacterized protein | 1621 | 98.173119 | 540 | 44.030059 | 1.1568393 | 8.33E-67 | 4.13E-68 | up |
| VIT_01s0011g04310.t01 | 591 | E3 ubiquitin-protein ligase RHA2A-like | 1030 | 117.68854 | 343 | 52.763915 | 1.1573504 | 7.66E-43 | 5.81E-44 | up |
| VIT_18s0001g11890.t01 | 1118 | Putative uncharacterized protein | 1599 | 96.580868 | 532 | 43.261363 | 1.1586583 | 4.47E-66 | 2.25E-67 | up |
| VIT_16s0013g01920.t01 | 2839 | U-box domain-containing protein 35-like | 352 | 8.3726264 | 117 | 3.7467166 | 1.1600534 | 2.16E-15 | 3.81E-16 | up |
| VIT_03s0063g01950.t01 | 810 | Putative uncharacterized protein | 292 | 24.343458 | 97 | 10.887222 | 1.1608982 | 5.79E-13 | 1.15E-13 | up |
| VIT_00s0227g00160.t01 | 798 | Putative uncharacterized protein | 813 | 68.797411 | 270 | 30.760348 | 1.1612825 | 3.29E-34 | 3.02E-35 | up |
| VIT_13s0019g05370.t01 | 452 | histone H2B-like | 2009 | 300.14143 | 667 | 134.15837 | 1.1617054 | 4.01E-83 | 1.62E-84 | up |
| VIT_18s0001g03520.t01 | 2137 | Putative uncharacterized protein | 232 | 7.3310791 | 77 | 3.2757939 | 1.162181 | 1.50E-10 | 3.55E-11 | up |
| VIT_01s0146g00480.t01 | 1079 | protein TIFY 9 (JASMONATE-ZIM-DOMAIN PROTEIN 10) | 232 | 14.519477 | 77 | 6.4878329 | 1.162181 | 1.50E-10 | 3.55E-11 | up |
| VIT_02s0025g03000.t01 | 1834 | B3 domain-containing protein | 1881 | 69.258632 | 624 | 30.932543 | 1.1628684 | 5.27E-78 | 2.24E-79 | up |
| VIT_08s0007g06740.t01 | 1539 | SINA2p | 208 | 9.126603 | 69 | 4.0760626 | 1.1629018 | 1.39E-09 | 3.54E-10 | up |
| VIT_11s0118g00510.t01 | 2087 | NEDD8 ultimate buster 1-like | 190 | 6.1477415 | 63 | 2.7444067 | 1.1635622 | 7.43E-09 | 2.00E-09 | up |
| VIT_12s0028g03600.t01 | 588 | Putative uncharacterized protein | 471 | 54.091375 | 156 | 24.120019 | 1.1651675 | 2.30E-20 | 3.25E-21 | up |
| VIT_05s0029g00500.t01 | 951 | chaperone protein dnaJ 20 | 641 | 45.515776 | 212 | 20.266825 | 1.1672466 | 2.10E-27 | 2.32E-28 | up |
| VIT_07s0104g00110.t01 | 666 | Putative uncharacterized protein | 121 | 12.268616 | 40 | 5.4602954 | 1.1679217 | 4.52E-06 | 1.58E-06 | up |
| VIT_00s0259g00140.t01 | 888 | oligopeptide transporter 3-like | 666 | 50.646065 | 220 | 22.523718 | 1.1690052 | 1.70E-28 | 1.81E-29 | up |
| VIT_11s0016g05410.t01 | 2791 | EIN3-binding F-box protein 1-like | 1432 | 34.647159 | 473 | 15.407482 | 1.1691059 | 4.09E-60 | 2.23E-61 | up |
| VIT_16s0098g00870.t01 | 1282 | WD-repeat 1 (WDR1) | 1063 | 55.992477 | 351 | 24.891408 | 1.1695852 | 6.50E-45 | 4.72E-46 | up |
| VIT_18s0001g02030.t01 | 1060 | thylakoidal processing peptidase 1 | 97 | 6.179457 | 32 | 2.7445711 | 1.1708994 | 4.22E-05 | 1.65E-05 | up |
| VIT_01s0011g06450.t01 | 1143 | probable NAD(P)H-dependent oxidoreductase 1-like | 94 | 5.5534909 | 31 | 2.4657318 | 1.1713791 | 5.60E-05 | 2.22E-05 | up |
| VIT_04s0023g01280.t01 | 749 | NEDD8-conjugating enzyme Ubc12-like | 176 | 15.867748 | 58 | 7.040063 | 1.1724371 | 2.27E-08 | 6.32E-09 | up |
| VIT_19s0014g05430.t01 | 903 | protein NDR1 | 3514 | 262.78372 | 1158 | 116.58728 | 1.1724654 | 7.19E-147 | 1.61E-148 | up |
| VIT_16s0050g00870.t01 | 1827 | BAHD acyltransferase DCR-like | 261 | 9.6468695 | 86 | 4.2794729 | 1.1726278 | 7.03E-12 | 1.51E-12 | up |
| VIT_15s0024g00400.t01 | 3465 | putative disease resistance protein RGA4-like | 623 | 12.141414 | 205 | 5.3787455 | 1.1745948 | 6.38E-27 | 7.14E-28 | up |
| VIT_11s0037g01120.t01 | 590 | cytochrome c oxidase subunit 6b-1-like | 1169 | 133.79717 | 384 | 59.171092 | 1.1770832 | 8.73E-50 | 5.76E-51 | up |
| VIT_17s0053g00850.t01 | 825 | cyclic nucleotide-gated ion channel 20 | 67 | 5.4840991 | 22 | 2.4243711 | 1.1776441 | 0.00069388 | 0.00032293 | up |
| VIT_01s0011g04630.t01 | 1340 | probable ribose-5-phosphate isomerase-like | 3704 | 186.65973 | 1216 | 82.500988 | 1.1779274 | 6.46E-156 | 1.38E-157 | up |
| VIT_11s0065g00730.t01 | 1375 | F-box protein CPR30-like | 128 | 6.286251 | 42 | 2.7770069 | 1.1786691 | 1.95E-06 | 6.56E-07 | up |
| VIT_14s0030g00440.t01 | 1574 | gibberellin receptor GID1A (GID1A) | 1341 | 57.53187 | 440 | 25.41431 | 1.1787203 | 4.38E-57 | 2.52E-58 | up |
| VIT_04s0023g01080.t01 | 4136 | lysM domain receptor-like kinase 4 | 436 | 7.1185314 | 143 | 3.1433003 | 1.1792995 | 2.81E-19 | 4.15E-20 | up |
| VIT_12s0121g00300.t01 | 1406 | BRASSINOSTEROID INSENSITIVE 1-associated receptor kinase 1 | 421 | 20.220003 | 138 | 8.9232721 | 1.1801385 | 1.14E-18 | 1.72E-19 | up |
| VIT_14s0068g00880.t01 | 732 | 40S ribosomal protein S27-2-like | 2987 | 275.55518 | 979 | 121.59115 | 1.180303 | 2.72E-126 | 7.17E-128 | up |
| VIT_08s0007g01310.t01 | 822 | probable peptide/nitrate transporter | 116 | 9.529511 | 38 | 4.2028332 | 1.18104 | 5.95E-06 | 2.11E-06 | up |
| VIT_14s0060g01760.t01 | 773 | Putative uncharacterized protein | 730 | 63.771673 | 239 | 28.109219 | 1.1818724 | 1.14E-31 | 1.12E-32 | up |
| VIT_14s0171g00110.t01 | 1179 | triacylglycerol lipase 2-like | 104 | 5.956676 | 34 | 2.6217754 | 1.1839634 | 1.82E-05 | 6.81E-06 | up |
| VIT_06s0004g07220.t01 | 1740 | UDP-glycosyltransferase 87A2-like | 747 | 28.990506 | 244 | 12.748848 | 1.1852136 | 1.60E-32 | 1.53E-33 | up |
| VIT_13s0064g01300.t01 | 954 | ras-related protein RGP1-like | 248 | 17.554471 | 81 | 7.7191062 | 1.1853328 | 1.60E-11 | 3.53E-12 | up |
| VIT_12s0059g01470.t01 | 899 | Putative uncharacterized protein | 144 | 10.816512 | 47 | 4.7530079 | 1.1863227 | 3.66E-07 | 1.14E-07 | up |
| VIT_03s0038g00880.t01 | 1975 | amidophosphoribosyltransferase | 1791 | 61.236862 | 584 | 26.8829 | 1.1877116 | 7.88E-77 | 3.40E-78 | up |
| VIT_19s0014g01580.t01 | 292 | Putative uncharacterized protein | 1561 | 360.99775 | 509 | 158.47666 | 1.1877195 | 4.38E-67 | 2.15E-68 | up |
| VIT_18s0001g09080.t01 | 264 | Putative uncharacterized protein | 319 | 81.596438 | 104 | 35.814574 | 1.1879594 | 1.50E-14 | 2.75E-15 | up |
| VIT_13s0067g00450.t01 | 550 | pleckstrin homology domain-containing protein 1-like | 819 | 100.55546 | 267 | 44.134575 | 1.1880102 | 1.09E-35 | 9.69E-37 | up |
| VIT_16s0013g01000.t01 | 946 | ethylene-responsive transcription factor 5-like | 1273 | 90.870247 | 415 | 39.882955 | 1.1880357 | 6.55E-55 | 3.91E-56 | up |
| VIT_00s0203g00200.t01 | 911 | Putative uncharacterized protein | 2163 | 160.33288 | 705 | 70.355996 | 1.188325 | 1.12E-92 | 4.05E-94 | up |
| VIT_15s0048g00790.t01 | 1063 | probable calcium-binding protein CML10-like | 270 | 17.152007 | 88 | 7.5262698 | 1.1883705 | 1.73E-12 | 3.56E-13 | up |
| VIT_05s0062g00250.t01 | 904 | brassinosteroid-regulated protein BRU1-like | 89 | 6.6482298 | 29 | 2.9164863 | 1.1887389 | 7.36E-05 | 2.97E-05 | up |
| VIT_08s0007g01290.t01 | 2598 | BEL1-like homeodomain protein 9 | 310 | 8.0576239 | 101 | 3.5343748 | 1.1888994 | 3.46E-14 | 6.48E-15 | up |
| VIT_01s0127g00490.t01 | 1584 | pantothenate kinase 2-like | 221 | 9.4215323 | 72 | 4.1324508 | 1.1889641 | 1.96E-10 | 4.67E-11 | up |
| VIT_04s0008g01250.t01 | 938 | Putative uncharacterized protein | 2364 | 170.18806 | 770 | 74.630828 | 1.1892862 | 2.45E-101 | 8.14E-103 | up |
| VIT_00s0652g00010.t01 | 1643 | cysteine-rich receptor-like protein kinase 15 | 261 | 10.727225 | 85 | 4.7033981 | 1.1895016 | 3.98E-12 | 8.39E-13 | up |
| VIT_00s1543g00010.t01 | 860 | Putative uncharacterized protein | 1505 | 118.17415 | 490 | 51.79979 | 1.1898963 | 6.72E-65 | 3.42E-66 | up |
| VIT_18s0122g00780.t01 | 1853 | probable inorganic phosphate transporter 1-9-like | 1023 | 37.280751 | 333 | 16.338011 | 1.1901986 | 1.88E-44 | 1.38E-45 | up |
| VIT_00s0780g00040.t01 | 1645 | E3 ubiquitin-protein ligase RGLG2-like | 166 | 6.8143844 | 54 | 2.9844082 | 1.1911384 | 3.93E-08 | 1.12E-08 | up |
| VIT_09s0002g00810.t01 | 1233 | peroxisomal (S)-2-hydroxy-acid oxidase GLO4-like | 747 | 40.911176 | 243 | 17.917341 | 1.1911384 | 8.97E-33 | 8.56E-34 | up |
| VIT_18s0001g09140.t01 | 1126 | sigma factor sigB regulation protein rsbQ-like | 4363 | 261.65634 | 1419 | 114.57091 | 1.1914324 | 7.24E-187 | 1.29E-188 | up |
| VIT_02s0012g00930.t01 | 717 | Putative uncharacterized protein | 532 | 50.104522 | 173 | 21.935994 | 1.1916407 | 1.18E-23 | 1.47E-24 | up |
| VIT_09s0018g01410.t01 | 392 | probable serine incorporator-like | 1671 | 287.8557 | 543 | 125.93433 | 1.1926741 | 3.19E-72 | 1.49E-73 | up |
| VIT_05s0094g01530.t01 | 1493 | Putative uncharacterized protein | 397 | 17.956229 | 129 | 7.8552548 | 1.1927545 | 5.95E-18 | 9.31E-19 | up |
| VIT_09s0002g01690.t01 | 676 | EPIDERMAL PATTERNING FACTOR-like protein 3 | 117 | 11.687553 | 38 | 5.1105457 | 1.1934237 | 4.48E-06 | 1.57E-06 | up |
| VIT_09s0002g01050.t01 | 693 | RING-box protein 1a-like | 231 | 22.509362 | 75 | 9.8391686 | 1.1939169 | 6.41E-11 | 1.47E-11 | up |
| VIT_14s0066g01080.t01 | 886 | ADP-ribosylation factor-like | 2310 | 176.06081 | 749 | 76.856122 | 1.1958417 | 6.67E-100 | 2.25E-101 | up |
| VIT_12s0034g01180.t01 | 1028 | probable plastid-lipid-associated protein 4 | 182 | 11.955362 | 59 | 5.2178221 | 1.1961381 | 7.31E-09 | 1.96E-09 | up |
| VIT_13s0158g00090.t01 | 1006 | protein BPS1 | 284 | 19.063595 | 92 | 8.3141952 | 1.1971717 | 3.21E-13 | 6.32E-14 | up |
| VIT_00s1553g00010.t01 | 788 | subtilisin-like protease-like | 71 | 6.0843834 | 23 | 2.6535788 | 1.1971717 | 0.00039491 | 0.00017638 | up |
| VIT_05s0062g00520.t01 | 824 | UDP-glycosyltransferase 75D1-like | 71 | 5.8185608 | 23 | 2.5376458 | 1.1971717 | 0.00039491 | 0.00017638 | up |
| VIT_04s0008g02380.t01 | 1543 | F-box protein AFR-like | 707 | 30.941255 | 229 | 13.492733 | 1.1973491 | 2.54E-31 | 2.51E-32 | up |
| VIT_14s0068g00750.t01 | 1149 | Putative uncharacterized protein | 315 | 18.512922 | 102 | 8.0706872 | 1.1977692 | 1.48E-14 | 2.72E-15 | up |
| VIT_02s0025g05150.t01 | 687 | general transcription factor IIH subunit 5-like | 139 | 13.662888 | 45 | 5.9550601 | 1.1980745 | 4.81E-07 | 1.51E-07 | up |
| VIT_04s0044g01360.t01 | 434 | GDP-mannose transporter GONST1-like | 173 | 26.917878 | 56 | 11.730828 | 1.1982598 | 1.69E-08 | 4.66E-09 | up |
| VIT_17s0000g00010.t01 | 1601 | Putative uncharacterized protein | 1267 | 53.440403 | 410 | 23.28214 | 1.1987072 | 2.01E-55 | 1.19E-56 | up |
| VIT_02s0033g00130.t01 | 1658 | scopoletin glucosyltransferase-like | 921 | 37.511078 | 298 | 16.340378 | 1.1988753 | 1.42E-40 | 1.13E-41 | up |
| VIT_04s0023g00620.t01 | 548 | Putative uncharacterized protein | 68 | 8.3793976 | 22 | 3.6498288 | 1.1990177 | 0.00052149 | 0.00023782 | up |
| VIT_07s0141g00660.t01 | 530 | signal recognition particle 9 kDa protein-like | 133 | 16.945727 | 43 | 7.3760348 | 1.2000042 | 8.38E-07 | 2.70E-07 | up |
| VIT_04s0008g02760.t01 | 881 | Putative uncharacterized protein | 99 | 7.5882867 | 32 | 3.3022081 | 1.2003431 | 2.39E-05 | 9.07E-06 | up |
| VIT_00s0246g00110.t01 | 505 | mitochondrial genome | 164 | 21.929913 | 53 | 9.5414607 | 1.2006181 | 3.90E-08 | 1.11E-08 | up |

| | | | | | | | | | | |
|---|---|---|---|---|---|---|---|---|---|---|
| VIT_07s0005g04700.t01 | 752 | dihydroneopterin aldolase-like | 294 | 26.400608 | 95 | 11.485136 | 1.2008032 | 1.04E-13 | 2.00E-14 | up |
| VIT_08s0007g05070.t01 | 615 | zinc finger protein 593-like | 1105 | 121.33095 | 357 | 52.77442 | 1.2010369 | 1.30E-48 | 8.75E-50 | up |
| VIT_13s0064g01020.t01 | 2188 | Putative uncharacterized protein | 192 | 5.9256822 | 62 | 2.5761713 | 1.2017527 | 2.38E-09 | 6.15E-10 | up |
| VIT_04s0044g01560.t01 | 2615 | probable receptor protein kinase TMK1 | 629 | 16.242893 | 203 | 7.0575622 | 1.2025668 | 3.65E-28 | 3.92E-29 | up |
| VIT_01s0150g00610.t01 | 948 | Putative uncharacterized protein | 2089 | 148.80398 | 674 | 64.63711 | 1.2029785 | 2.82E-91 | 1.03E-92 | up |
| VIT_01s0011g05910.t01 | 1209 | S-adenosylmethionine-dependent methyltransferas | 183 | 10.221373 | 59 | 4.4366593 | 1.2040433 | 5.48E-09 | 1.46E-09 | up |
| VIT_06s0009g01930.t01 | 1082 | SNF1-related protein kinase regulatory subunit beta-1-like | 910 | 56.793492 | 293 | 24.619018 | 1.2059524 | 1.81E-40 | 1.45E-41 | up |
| VIT_15s0021g00540.t01 | 700 | putative invertase inhibitor-like | 146 | 14.084429 | 47 | 6.1042202 | 1.2062222 | 2.07E-07 | 6.28E-08 | up |
| VIT_19s0014g03520.t01 | 1339 | Putative uncharacterized protein | 379 | 19.113626 | 122 | 8.2834189 | 1.2063032 | 1.78E-17 | 2.83E-18 | up |
| VIT_06s0004g06070.t01 | 873 | Putative uncharacterized protein | 258 | 19.956754 | 83 | 8.6435913 | 1.2071743 | 2.95E-12 | 6.16E-13 | up |
| VIT_07s0005g02170.t01 | 1601 | Putative uncharacterized protein | 3025 | 127.59054 | 973 | 55.252493 | 1.2074099 | 1.53E-132 | 3.79E-134 | up |
| VIT_19s0014g01800.t01 | 1636 | homogentisate 1,2-dioxygenase-like | 255 | 10.525466 | 82 | 4.5568101 | 1.2077879 | 3.89E-12 | 8.19E-13 | up |
| VIT_04s0023g03580.t01 | 1420 | E3 ubiquitin-protein ligase RNF167-like | 168 | 7.9892384 | 54 | 3.4572898 | 1.2084164 | 2.21E-08 | 6.16E-09 | up |
| VIT_18s0001g08080.t01 | 245 | Aux/IAA protein | 224 | 61.739965 | 72 | 26.717559 | 1.2084164 | 8.33E-11 | 1.93E-11 | up |
| VIT_07s0005g04750.t01 | 716 | Putative uncharacterized protein | 5069 | 478.07244 | 1629 | 206.84186 | 1.2087011 | 5.87E-222 | 8.45E-224 | up |
| VIT_08s0058g01230.t01 | 646 | non-specific lipid-transfer protein | 72909 | 7621.3704 | 23408 | 3294.2925 | 1.2100817 | 0 | 0 | up |
| VIT_12s0028g00710.t01 | 1633 | 4-hydroxyphenylpyruvate dioxygenase-like | 6097 | 252.12415 | 1957 | 108.95195 | 1.2104423 | 1.82E-267 | 2.25E-269 | up |
| VIT_03s0091g00640.t01 | 498 | 40S ribosomal protein S29-like | 6635 | 899.69649 | 2127 | 388.30101 | 1.2122629 | 1.02E-291 | 1.19E-293 | up |
| VIT_04s0008g05470.t01 | 2909 | EIN3-binding F-box protein 1-like | 4124 | 95.732495 | 1322 | 41.315985 | 1.2123087 | 1.41E-181 | 2.60E-183 | up |
| VIT_19s0015g00190.t01 | 1329 | zinc transporter 11-like | 259 | 13.160101 | 83 | 5.6778444 | 1.2127554 | 2.22E-12 | 4.58E-13 | up |
| VIT_02s0154g00390.t01 | 597 | autophagy-related protein 8f-like | 409 | 46.26296 | 131 | 19.949285 | 1.2135205 | 6.08E-19 | 9.10E-20 | up |
| VIT_02s0025g00510.t01 | 3159 | respiratory burst oxidase | 281 | 6.0067719 | 90 | 2.5901401 | 1.2135597 | 2.37E-13 | 4.62E-14 | up |
| VIT_03s0063g00800.t01 | 1133 | carboxylesterase 12 | 253 | 15.079087 | 81 | 6.4995828 | 1.2141301 | 3.85E-12 | 8.11E-13 | up |
| VIT_13s0175g00010.t01 | 225 | nucleolar GTP-binding protein 1-like | 75 | 22.509362 | 24 | 9.6974845 | 1.2148427 | 0.00022354 | 9.63E-05 | up |
| VIT_15s0048g01780.t01 | 709 | peptidyl-prolyl cis-trans isomerase-like 3-like | 100 | 9.5244128 | 32 | 4.103308 | 1.2148427 | 1.79E-05 | 6.70E-06 | up |
| VIT_10s0003g00140.t01 | 1193 | ethylene-responsive transcription factor 4-like | 2944 | 166.64098 | 942 | 71.786178 | 1.2149652 | 2.76E-130 | 6.98E-132 | up |
| VIT_17s0000g10000.t01 | 662 | RPM1-interacting protein 4-like | 197 | 20.095216 | 63 | 8.6519287 | 1.2157584 | 1.01E-09 | 2.55E-10 | up |
| VIT_09s0070g00380.t01 | 948 | Putative uncharacterized protein | 1020 | 72.656802 | 326 | 31.263647 | 1.2166118 | 7.41E-46 | 5.29E-47 | up |
| VIT_18s0001g13400.t01 | 1119 | cysteine proteinase 15A | 504 | 30.414795 | 161 | 13.080555 | 1.2173496 | 3.34E-23 | 4.24E-24 | up |
| VIT_08s0007g03040.t01 | 1237 | 1-aminocyclopropane-1-carboxylate oxidase | 949 | 51.806107 | 303 | 22.269133 | 1.2180768 | 8.17E-43 | 6.20E-44 | up |
| VIT_16s0098g01390.t01 | 852 | protein yippee-like | 94 | 7.4502818 | 30 | 3.2011943 | 1.2186848 | 3.13E-05 | 1.20E-05 | up |
| VIT_16s0039g00730.t01 | 610 | Putative uncharacterized protein | 185 | 20.479829 | 59 | 8.7933133 | 1.2197249 | 3.08E-09 | 8.03E-10 | up |
| VIT_00s0820g00020.t01 | 604 | UDP-glycosyltransferase 91A1 | 207 | 23.142904 | 66 | 9.9343023 | 1.2200793 | 3.30E-10 | 7.99E-11 | up |
| VIT_11s0016g00690.t01 | 892 | Putative uncharacterized protein | 248 | 18.774625 | 79 | 8.0517932 | 1.2214021 | 5.03E-12 | 1.07E-12 | up |
| VIT_06s0004g02370.t01 | 979 | dihydroflavonol-4-reductase-like | 135 | 9.3118403 | 43 | 3.9931547 | 1.2215374 | 4.71E-07 | 1.48E-07 | up |
| VIT_05s0094g01120.t01 | 1240 | NADP-dependent alkenal double bond reductase P2-like | 1407 | 76.622595 | 448 | 32.846319 | 1.2220382 | 2.62E-63 | 1.36E-64 | up |
| VIT_13s0067g02960.t01 | 1252 | macro domain-containing protein VPA0103-like | 201 | 10.84117 | 64 | 4.6473568 | 1.2220382 | 5.74E-10 | 1.42E-10 | up |
| VIT_01s0011g05470.t01 | 937 | protein PLANT CADMIUM RESISTANCE 2-like | 5540 | 399.25891 | 1763 | 171.05788 | 1.22284 | 5.26E-247 | 6.85E-249 | up |
| VIT_04s0008g06170.t01 | 1354 | Putative uncharacterized protein | 1029 | 51.319351 | 327 | 21.956315 | 1.224867 | 9.77E-47 | 6.84E-48 | up |
| VIT_18s0001g06580.t01 | 785 | petE; plastocyanin | 535 | 46.022326 | 170 | 19.688364 | 1.2249907 | 8.52E-25 | 1.02E-25 | up |
| VIT_14s0066g01500.t01 | 543 | UDP-glycosyltransferase 90A1-like | 362 | 45.018724 | 115 | 19.254329 | 1.2253423 | 3.91E-17 | 6.35E-18 | up |
| VIT_00s0259g00050.t01 | 255 | Putative uncharacterized protein | 63 | 16.68341 | 20 | 7.1305033 | 1.2263383 | 0.00068216 | 0.00031717 | up |
| VIT_14s0006g00430.t01 | 1539 | isoprenylcysteine alpha-carbonyl methylesterase ICME-like | 189 | 8.2929229 | 60 | 3.5444022 | 1.2263383 | 1.74E-09 | 4.46E-10 | up |
| VIT_13s0019g03220.t01 | 1717 | proline transporter 2-like | 646 | 25.406607 | 205 | 10.854603 | 1.2268968 | 8.55E-30 | 8.80E-31 | up |
| VIT_16s0100g01220.t01 | 446 | protein phosphatase inhibitor 2 | 82 | 12.415478 | 26 | 5.2999145 | 1.2280988 | 9.56E-05 | 3.91E-05 | up |
| VIT_19s0014g03310.t01 | 596 | Putative uncharacterized protein | 82 | 9.290777 | 26 | 3.9660434 | 1.2280988 | 9.56E-05 | 3.91E-05 | up |
| VIT_15s0046g00120.t01 | 1332 | Putative uncharacterized protein | 975 | 49.429343 | 309 | 21.090391 | 1.2287819 | 1.48E-44 | 1.08E-45 | up |
| VIT_02s0025g00900.t01 | 1821 | 6-phosphogluconate dehydrogenase | 5448 | 202.02801 | 1726 | 86.171017 | 1.2292807 | 6.32E-245 | 8.28E-247 | up |
| VIT_18s0001g01490.t01 | 1370 | uncharacterized oxidoreductase | 4880 | 240.538 | 1545 | 102.52701 | 1.2302608 | 9.87E-220 | 1.44E-221 | up |
| VIT_14s0128g00760.t01 | 1861 | protein AUXIN RESPONSE 4-like | 1953 | 70.866391 | 618 | 30.190651 | 1.2309997 | 1.62E-88 | 6.09E-90 | up |
| VIT_08s0040g03360.t01 | 988 | actin-depolymerizing factor 1-like | 5822 | 397.9236 | 1842 | 169.49741 | 1.2312283 | 2.34E-262 | 2.92E-264 | up |
| VIT_04s0044g01620.t01 | 1526 | E3 ubiquitin-protein ligase RGLG1-like | 449 | 19.869011 | 142 | 8.4598796 | 1.231811 | 3.71E-21 | 5.07E-22 | up |
| VIT_16s0039g01560.t01 | 1189 | Putative uncharacterized protein | 174 | 9.882159 | 55 | 4.2054377 | 1.2325703 | 7.00E-09 | 1.88E-09 | up |
| VIT_14s0030g01950.t01 | 1633 | hydroxycinnamoyl-Coenzyme A shikimate/quinate hydroxycinnamoyltransferase-like | 1946 | 80.471314 | 615 | 34.238861 | 1.2328399 | 2.07E-88 | 7.84E-90 | up |
| VIT_07s0129g01060.t01 | 1420 | putative oxidoreductase C1F5.03c-like | 1174 | 55.829559 | 371 | 23.752862 | 1.2329278 | 1.02E-53 | 6.19E-55 | up |
| VIT_17s0000g02800.t01 | 717 | hydroxymethylglutaryl-CoA synthase / HMG-CoA synthase / 3-hydroxy-3-methylglu | 114 | 10.736683 | 36 | 4.5647155 | 1.2339515 | 3.29E-06 | 1.13E-06 | up |
| VIT_06s0009g00320.t01 | 1278 | E3 ubiquitin-protein ligase PUB23-like | 371 | 19.603224 | 117 | 8.3231051 | 1.2358972 | 9.33E-18 | 1.47E-18 | up |
| VIT_15s0021g02490.t01 | 628 | protein transport protein Sec61 subunit beta-like | 1018 | 109.46432 | 321 | 46.47033 | 1.2360789 | 6.70E-47 | 4.67E-48 | up |
| VIT_09s0002g04150.t01 | 834 | peroxyureidoacrylate/ureidoacrylate amidohydrolase RutB-like | 184 | 14.898283 | 58 | 6.3225506 | 1.2365675 | 2.27E-09 | 5.86E-10 | up |
| VIT_08s0040g01580.t01 | 927 | probable receptor-like protein kinase | 92 | 6.7018166 | 29 | 2.8441247 | 1.2365675 | 3.09E-05 | 1.19E-05 | up |
| VIT_14s0066g00810.t01 | 2868 | galactinol--sucrose galactosyltransferase-like | 384 | 9.0414174 | 121 | 3.835629 | 1.2370858 | 2.29E-18 | 3.51E-19 | up |
| VIT_12s0057g01430.t01 | 772 | Putative uncharacterized protein | 740 | 64.728995 | 233 | 27.439045 | 1.2381818 | 1.85E-34 | 1.69E-35 | up |
| VIT_15s0046g01230.t01 | 1396 | protein trichome birefringence-like 25 | 956 | 46.244162 | 301 | 19.602499 | 1.2382336 | 3.18E-44 | 2.34E-45 | up |
| VIT_05s0020g00840.t01 | 1112 | late embryogenesis abundant protein D-29-like | 89 | 5.404676 | 28 | 2.2891994 | 1.239365 | 4.07E-05 | 1.59E-05 | up |
| VIT_01s0011g04270.t01 | 1862 | Protein kinase domain containing protein | 728 | 26.401959 | 229 | 11.181142 | 1.2395774 | 5.64E-34 | 5.21E-35 | up |
| VIT_08s0058g00270.t01 | 3001 | importin subunit beta-1-like | 1774 | 39.918302 | 558 | 16.904354 | 1.2396555 | 2.26E-81 | 9.31E-83 | up |
| VIT_12s0028g00590.t01 | 1551 | 3-hydroxyisobutyryl-CoA hydrolase-like protein 5-like | 341 | 14.846601 | 107 | 6.2719466 | 1.2431475 | 1.50E-16 | 2.50E-17 | up |
| VIT_10s0003g04540.t01 | 2097 | high affinity cationic amino acid transporter 1-like | 1129 | 36.356323 | 354 | 15.347414 | 1.2442107 | 1.98E-52 | 1.24E-53 | up |
| VIT_09s0070g00240.t01 | 1306 | cinnamoyl-CoA reductase | 2306 | 119.23413 | 723 | 50.329833 | 1.2443115 | 4.03E-106 | 1.27E-107 | up |
| VIT_10s0003g05450.t01 | 1731 | reticuline oxidase-like protein-like | 1780 | 69.439627 | 558 | 29.306739 | 1.2445267 | 3.84E-82 | 1.56E-83 | up |
| VIT_01s0026g01570.t01 | 1960 | nitrate transporter 1.2-like | 399 | 13.746789 | 125 | 5.7980815 | 1.2454472 | 3.12E-19 | 4.61E-20 | up |
| VIT_14s0006g01420.t01 | 1732 | probable receptor-like serine/threonine-protein kinase | 211 | 8.226574 | 66 | 3.4643872 | 1.2476916 | 1.03E-10 | 2.39E-11 | up |
| VIT_13s0019g04280.t01 | 1238 | aquaporin (PIP2-1) | 19322 | 1053.94 | 6043 | 443.77448 | 1.2478941 | 0 | 0 | up |
| VIT_00s0479g00010.t01 | 486 | serine/threonine-protein kinase HT1 | 208 | 28.900909 | 65 | 12.159269 | 1.2490584 | 1.35E-10 | 3.18E-11 | up |
| VIT_10s0003g04320.t01 | 1727 | protein kinase PINOID-like | 160 | 6.2562211 | 50 | 2.6321343 | 1.2490584 | 2.09E-08 | 5.82E-09 | up |
| VIT_10s0003g00510.t01 | 536 | transcription elongation factor 1 homolog | 464 | 58.457149 | 145 | 24.59425 | 1.2490584 | 2.75E-22 | 3.61E-23 | up |
| VIT_17s0053g00810.t01 | 954 | probable cyclic nucleotide-gated ion channel 20 | 77 | 5.4503801 | 24 | 2.2871426 | 1.2528105 | 0.00012453 | 5.16E-05 | up |
| VIT_14s0006g01400.t01 | 838 | calcium-binding allergen Ole e 8-like | 645 | 51.975675 | 201 | 21.806322 | 1.2530902 | 1.02E-30 | 1.02E-31 | up |
| VIT_04s0008g06870.t01 | 534 | probable steroid-binding protein 3-like | 12343 | 1560.8599 | 3844 | 654.44401 | 1.2539994 | 0 | 0 | up |
| VIT_14s0108g01470.t01 | 398 | EID1-like F-box protein 2 | 501 | 85.003948 | 156 | 35.634601 | 1.2542511 | 3.97E-24 | 4.87E-25 | up |
| VIT_01s0010g02030.t01 | 375 | defensin Ec-AMP-D2 | 2637 | 474.8575 | 821 | 199.04087 | 1.25443 | 9.13E-123 | 2.48E-124 | up |
| VIT_11s0016g03780.t01 | 1220 | Putative uncharacterized protein | 636 | 35.203166 | 198 | 14.754882 | 1.2545128 | 2.34E-30 | 2.38E-31 | up |
| VIT_00s0211g00170.t01 | 480 | Putative uncharacterized protein | 90 | 12.661516 | 28 | 5.3033119 | 1.2554847 | 3.03E-05 | 1.17E-05 | up |
| VIT_02s0025g03390.t01 | 1100 | plasma membrane aquaporin (PIP1b) | 66005 | 4051.9921 | 20509 | 1695.0487 | 1.2573047 | 0 | 0 | up |
| VIT_14s0060g02250.t01 | 1653 | heparan-alpha-glucosaminide N-acetyltransferase-like | 522 | 21.324659 | 162 | 8.9098939 | 1.2590425 | 3.08E-25 | 3.63E-26 | up |
| VIT_03s0038g02440.t01 | 723 | heavy metal-associated isoprenylated plant protein 26 | 3527 | 329.42125 | 1094 | 137.56546 | 1.2598153 | 5.09E-165 | 1.01E-166 | up |
| VIT_19s0177g00360.t01 | 795 | trafficking protein particle complex subunit 3-like | 503 | 42.725318 | 156 | 17.839712 | 1.2599989 | 2.20E-24 | 2.66E-25 | up |
| VIT_19s0027g00460.t01 | 822 | glutathione S-transferase | 545 | 44.772271 | 169 | 18.691548 | 1.2602195 | 2.43E-26 | 2.77E-27 | up |
| VIT_05s0094g01150.t01 | 1297 | NADP-dependent alkenal double bond reductase P1-like | 229 | 11.922846 | 71 | 4.9767835 | 1.2604432 | 1.06E-11 | 2.31E-12 | up |
| VIT_03s0063g02050.t01 | 1062 | E3 ubiquitin-protein ligase RNF5-like | 549 | 34.908587 | 170 | 14.553075 | 1.2622579 | 1.37E-26 | 1.55E-27 | up |
| VIT_09s0002g03610.t01 | 1523 | Putative uncharacterized protein | 1079 | 47.841632 | 334 | 19.937786 | 1.2627614 | 2.64E-51 | 1.69E-52 | up |
| VIT_15s0048g02090.t01 | 765 | acyl-coenzyme A thioesterase 13-like | 84 | 7.4148487 | 26 | 3.0898848 | 1.2628642 | 5.29E-05 | 2.09E-05 | up |
| VIT_02s0025g00350.t01 | 693 | Putative uncharacterized protein | 168 | 16.370445 | 52 | 6.8218235 | 1.2628642 | 6.63E-09 | 1.78E-09 | up |
| VIT_17s0000g05610.t01 | 996 | (-)-isopiperitenol/(-)-carveol dehydrogenase | 294 | 19.932989 | 91 | 8.3063921 | 1.2628642 | 9.41E-15 | 1.72E-15 | up |
| VIT_14s0030g01670.t01 | 1605 | 26S proteasome non-ATPase regulatory subunit 11-like | 2713 | 114.14561 | 839 | 47.524472 | 1.2641328 | 8.48E-128 | 2.20E-129 | up |
| VIT_08s0007g07530.t01 | 924 | short-chain type dehydrogenase/reductase | 401 | 29.30602 | 124 | 12.200569 | 1.2642486 | 9.33E-20 | 1.35E-20 | up |
| VIT_00s0615g00020.t01 | 1139 | probable mannitol dehydrogenase-like | 427 | 25.315622 | 132 | 10.536117 | 1.2646846 | 5.61E-21 | 7.71E-22 | up |

| | | | | | | | | | | |
|---|---|---|---|---|---|---|---|---|---|---|
| VIT_00s0184g00030.t01 | 1494 | Putative uncharacterized protein | 922 | 41.67396 | 285 | 17.343016 | 1.2647913 | 4.26E-44 | 3.15E-45 | up |
| VIT_07s0005g00480.t01 | 727 | 21 kDa protein | 343 | 31.859881 | 106 | 13.255674 | 1.2651308 | 4.50E-17 | 7.32E-18 | up |
| VIT_08s0007g06030.t01 | 1014 | Beta-1,3-glucanase | 123 | 8.1912768 | 38 | 3.4070304 | 1.2655735 | 7.80E-07 | 2.51E-07 | up |
| VIT_06s0004g03000.t01 | 1094 | pyridoxal biosynthesis protein PDX2-like | 1570 | 96.909594 | 485 | 40.304616 | 1.2656944 | 1.78E-74 | 7.97E-76 | up |
| VIT_04s0044g00630.t01 | 930 | serine/threonine-protein kinase ULK3-like | 204 | 14.812613 | 63 | 6.1586847 | 1.2661319 | 1.30E-10 | 3.05E-11 | up |
| VIT_01s0010g03880.t01 | 676 | Putative uncharacterized protein | 959 | 95.797981 | 296 | 39.808461 | 1.2669201 | 6.07E-46 | 4.32E-47 | up |
| VIT_07s0104g00440.t01 | 1019 | Beta-1,3-glucanase | 418 | 27.700432 | 129 | 11.50922 | 1.2671184 | 1.29E-20 | 1.80E-21 | up |
| VIT_00s2300g00010.t01 | 1590 | acyl-CoA--sterol O-acyltransferase 1 | 175 | 7.4323366 | 54 | 3.0876425 | 1.2673101 | 2.83E-09 | 7.36E-10 | up |
| VIT_18s0001g05580.t01 | 841 | Putative uncharacterized protein | 94 | 7.547729 | 29 | 3.1349627 | 1.2675944 | 1.70E-05 | 6.36E-06 | up |
| VIT_06s0004g06710.t01 | 2854 | zinc finger CCCH domain-containing protein 66 | 8253 | 195.27305 | 2545 | 81.07075 | 1.2682395 | 0 | 0 | up |
| VIT_06s0004g00190.t01 | 1332 | 2-oxoisovalerate dehydrogenase subunit beta, mitochondrial-like | 347 | 17.591776 | 107 | 7.303145 | 1.2683114 | 2.53E-17 | 4.07E-18 | up |
| VIT_13s0019g00870.t01 | 355 | dolichyl-diphosphooligosaccharide--protein glycosyltransferase subunit 4A-like | 253 | 48.12565 | 78 | 19.975452 | 1.2685779 | 6.32E-13 | 1.26E-13 | up |
| VIT_06s0004g04280.t01 | 812 | actin-depolymerizing factor 2-like | 2189 | 182.04308 | 674 | 75.46303 | 1.270438 | 4.42E-104 | 1.42E-105 | up |
| VIT_01s0010g01680.t01 | 2620 | Putative uncharacterized protein | 234 | 6.0311344 | 72 | 2.4983977 | 1.2714262 | 4.45E-12 | 9.41E-13 | up |
| VIT_08s0040g01430.t01 | 634 | membrane magnesium transporter | 856 | 91.173568 | 263 | 37.713502 | 1.2735345 | 1.92E-41 | 1.49E-42 | up |
| VIT_03s0097g00600.t01 | 1616 | Putative uncharacterized protein | 1670 | 69.784594 | 513 | 28.860668 | 1.2738039 | 6.10E-80 | 2.53E-81 | up |
| VIT_07s0031g01710.t01 | 571 | WRKY transcription factor 51 | 980 | 115.89759 | 301 | 47.92485 | 1.2740048 | 2.51E-47 | 1.73E-48 | up |
| VIT_11s0016g01340.t01 | 1009 | Putative uncharacterized protein | 4585 | 306.85459 | 1408 | 126.86501 | 1.2742609 | 3.96E-218 | 5.81E-220 | up |
| VIT_11s0052g01620.t01 | 874 | pathogenesis-related protein PR-1 | 606 | 46.821534 | 186 | 19.347813 | 1.2750017 | 1.09E-29 | 1.13E-30 | up |
| VIT_00s0459g00030.t01 | 419 | Putative uncharacterized protein | 88 | 14.18251 | 27 | 5.8584147 | 1.2755306 | 2.97E-05 | 1.14E-05 | up |
| VIT_18s0122g00870.t01 | 795 | RING-H2 finger protein ATL8 | 427 | 36.269802 | 131 | 14.980784 | 1.2756558 | 3.01E-21 | 4.10E-22 | up |
| VIT_08s0007g08390.t01 | 460 | Putative uncharacterized protein | 5327 | 782.0046 | 1634 | 322.94205 | 1.2759018 | 7.79E-254 | 9.89E-256 | up |
| VIT_19s0014g04730.t01 | 1228 | Putative uncharacterized protein | 6253 | 343.85434 | 1918 | 141.99747 | 1.2759323 | 6.96E-298 | 7.82E-300 | up |
| VIT_07s0104g00150.t01 | 359 | 6.7 kDa chloroplast outer envelope membrane protein | 1203 | 226.28492 | 368 | 93.193097 | 1.2798455 | 2.84E-58 | 1.60E-59 | up |
| VIT_01s0010g03870.t01 | 422 | Putative uncharacterized protein | 510 | 81.609773 | 156 | 33.607989 | 1.2799377 | 2.69E-25 | 3.17E-26 | up |
| VIT_18s0001g10100.t01 | 771 | SUMO-conjugating enzyme SCE1-like | 2950 | 258.37595 | 902 | 106.36103 | 1.2805021 | 1.10E-141 | 2.56E-143 | up |
| VIT_17s0000g06600.t01 | 711 | Putative uncharacterized protein | 72 | 6.8382872 | 22 | 2.8130889 | 1.2814799 | 0.00016022 | 6.77E-05 | up |
| VIT_13s0019g01080.t01 | 1503 | protein kinase APK1A | 550 | 24.710877 | 168 | 10.162035 | 1.2819569 | 2.91E-27 | 3.22E-28 | up |
| VIT_18s0117g00320.t01 | 659 | Putative uncharacterized protein | 2506 | 256.79118 | 765 | 105.5374 | 1.2828413 | 7.93E-121 | 2.18E-122 | up |
| VIT_06s0080g01200.t01 | 726 | protein YLS9 | 810 | 75.341253 | 247 | 30.930768 | 1.2843974 | 8.78E-40 | 7.14E-41 | up |
| VIT_16s0022g00470.t01 | 830 | peroxisomal membrane protein 11B | 915 | 74.443613 | 279 | 30.56022 | 1.2844931 | 8.21E-45 | 5.99E-46 | up |
| VIT_07s0005g01250.t01 | 957 | transmembrane protein 205-like | 1507 | 106.33733 | 459 | 43.604481 | 1.2860999 | 2.63E-73 | 1.20E-74 | up |
| VIT_17s0000g05470.t01 | 1871 | Putative uncharacterized protein | 417 | 15.050354 | 127 | 6.1710676 | 1.2862054 | 4.98E-21 | 6.83E-22 | up |
| VIT_00s0652g00020.t01 | 869 | Putative uncharacterized protein | 782 | 60.767507 | 238 | 24.899324 | 1.2871935 | 1.44E-38 | 1.20E-39 | up |
| VIT_04s0008g02090.t01 | 1040 | Copper transporter(CTR8) | 1627 | 105.6425 | 495 | 43.271528 | 1.2877003 | 3.24E-79 | 1.35E-80 | up |
| VIT_04s0079g00610.t01 | 834 | universal stress protein A-like protein | 309 | 25.019399 | 94 | 10.246892 | 1.2878607 | 6.81E-16 | 1.17E-16 | up |
| VIT_08s0007g00140.t01 | 2334 | Putative uncharacterized protein | 3002 | 86.854891 | 912 | 35.524204 | 1.2898048 | 8.61E-146 | 1.95E-147 | up |
| VIT_14s0068g01050.t01 | 819 | N-alpha-acetyltransferase 30-like | 191 | 15.748308 | 58 | 6.4383483 | 1.2904343 | 2.83E-10 | 6.82E-11 | up |
| VIT_18s0001g13040.t01 | 825 | ocs element-binding factor 1-like | 919 | 75.222196 | 279 | 30.745434 | 1.2907862 | 2.44E-45 | 1.76E-46 | up |
| VIT_16s0050g02480.t01 | 4990 | ABC transporter C family member 10-like | 1018 | 13.776271 | 309 | 5.6297396 | 1.2910453 | 3.97E-50 | 2.60E-51 | up |
| VIT_14s0068g01890.t01 | 2068 | Putative uncharacterized protein | 696 | 22.727054 | 211 | 9.2760332 | 1.2928308 | 1.15E-34 | 1.05E-35 | up |
| VIT_09s0002g05660.t01 | 1635 | molybdate transporter 2 | 1237 | 51.090057 | 375 | 20.851816 | 1.2928695 | 7.97E-61 | 4.29E-62 | up |
| VIT_03s0038g02110.t01 | 575 | chaperone protein dnaJ 11 | 759 | 89.137074 | 230 | 36.365567 | 1.2934525 | 9.74E-38 | 8.24E-39 | up |
| VIT_07s0104g00950.t01 | 1662 | ankyrin repeat domain-containing protein 65-like | 314 | 12.758014 | 95 | 5.1966439 | 1.2957516 | 2.83E-16 | 4.76E-17 | up |
| VIT_11s0016g01940.t01 | 437 | Putative uncharacterized protein | 334 | 51.611856 | 101 | 21.012141 | 1.2964793 | 2.94E-17 | 4.74E-18 | up |
| VIT_08s0032g00310.t01 | 935 | ubiquitin-conjugating enzyme E2 2-like | 579 | 41.816858 | 175 | 17.015974 | 1.2971949 | 3.72E-29 | 3.89E-30 | up |
| VIT_00s0225g00120.t01 | 559 | cyclin-dependent protein kinase inhibitor SMR3-like | 547 | 66.078467 | 165 | 26.835056 | 1.3000613 | 1.08E-27 | 1.18E-28 | up |
| VIT_11s0065g01070.t01 | 1251 | Putative uncharacterized protein | 1106 | 59.70109 | 333 | 24.200108 | 1.3027438 | 4.01E-55 | 2.38E-56 | up |
| VIT_18s0001g02950.t01 | 396 | Putative uncharacterized protein | 93 | 15.858869 | 28 | 6.4282568 | 1.3027904 | 1.23E-05 | 4.51E-06 | up |
| VIT_01s0011g01900.t01 | 1400 | Glutathione S-transferase | 163 | 7.8621986 | 49 | 3.1819871 | 1.3050048 | 4.54E-09 | 1.20E-09 | up |
| VIT_15s0046g00070.t01 | 446 | calcium-binding protein KIC | 183 | 27.707713 | 55 | 11.211358 | 1.3053266 | 4.77E-10 | 1.17E-10 | up |
| VIT_13s0067g01000.t01 | 1008 | PHD finger protein ALFIN-LIKE 1-like | 2310 | 154.75186 | 694 | 62.593511 | 1.3058718 | 1.52E-114 | 4.47E-116 | up |
| VIT_03s0038g04330.t01 | 531 | Putative uncharacterized protein | 70 | 8.9020076 | 21 | 3.5954657 | 1.3079521 | 0.00015573 | 6.57E-05 | up |
| VIT_17s0000g02070.t01 | 1313 | deoxyhypusine synthase-like | 130 | 6.6859492 | 39 | 2.7004134 | 1.3079521 | 1.77E-07 | 5.35E-08 | up |
| VIT_12s0059g02030.t01 | 665 | formimidoyltransferase-cyclodeaminase-like | 140 | 14.216439 | 42 | 5.7419316 | 1.3079521 | 5.74E-08 | 1.65E-08 | up |
| VIT_19s0014g02870.t01 | 2076 | Putative uncharacterized protein | 934 | 30.381133 | 280 | 12.261993 | 1.3089822 | 4.77E-47 | 3.31E-48 | up |
| VIT_02s0025g01600.t01 | 611 | Putative uncharacterized protein | 464 | 51.281558 | 139 | 20.682544 | 1.3100264 | 6.42E-24 | 7.91E-25 | up |
| VIT_07s0005g02950.t01 | 968 | GRF1-interacting factor 1 | 197 | 13.742803 | 59 | 5.5412408 | 1.3103953 | 8.66E-11 | 2.01E-11 | up |
| VIT_00s0323g00050.t01 | 860 | 21 kDa protein-like | 2850 | 223.78494 | 853 | 90.173921 | 1.3113308 | 5.03E-142 | 1.17E-143 | up |
| VIT_19s0014g03490.t01 | 384 | Putative uncharacterized protein | 107 | 18.81642 | 32 | 7.5761598 | 1.3124535 | 2.22E-06 | 7.50E-07 | up |
| VIT_08s0007g08310.t01 | 2672 | alpha-galactosidase (AGA) | 4634 | 117.11271 | 1385 | 47.124168 | 1.3133586 | 5.32E-231 | 7.34E-233 | up |
| VIT_15s0048g01850.t01 | 908 | probable xyloglucan endotransglucosylase/hydrolase protein 30-like | 609 | 45.291415 | 182 | 18.222834 | 1.3134903 | 3.42E-31 | 3.40E-32 | up |
| VIT_19s0014g05020.t01 | 691 | histone H2A.6-like | 6445 | 629.83866 | 1925 | 253.2696 | 1.3143084 | 0 | 0 | up |
| VIT_05s0020g03070.t01 | 1000 | cyclin-T1-4-like | 221 | 14.923707 | 66 | 6.0003186 | 1.3144949 | 5.11E-12 | 1.08E-12 | up |
| VIT_00s0705g00010.t01 | 1110 | isoflavone 2'-hydroxylase-like | 278 | 16.91244 | 83 | 6.7980677 | 1.3148882 | 7.60E-15 | 1.38E-15 | up |
| VIT_16s0050g01000.t01 | 1447 | Putative uncharacterized protein | 469 | 21.887127 | 140 | 8.7960943 | 1.3151476 | 2.64E-24 | 3.21E-25 | up |
| VIT_08s0007g02580.t01 | 819 | ubiquitin-conjugating enzyme E2 variant 1D-like | 5875 | 484.40477 | 1753 | 194.59353 | 1.3157494 | 1.52E-293 | 1.74E-295 | up |
| VIT_12s0059g01380.t01 | 1308 | 1-aminocyclopropane-1-carboxylic acid oxidase 2 (ACO2) | 3939 | 203.35866 | 1175 | 81.669613 | 1.3161552 | 4.10E-197 | 6.84E-199 | up |
| VIT_07s0141g00690.t01 | 810 | ethylene-responsive transcription factor 11-like | 114 | 9.5039529 | 34 | 3.8161398 | 1.3164137 | 9.46E-07 | 3.07E-07 | up |
| VIT_11s0149g00060.t01 | 1152 | Putative uncharacterized protein | 3255 | 190.80202 | 970 | 76.550781 | 1.3175874 | 2.72E-163 | 5.56E-165 | up |
| VIT_16s0013g00990.t01 | 964 | ethylene-responsive transcription factor 5 | 2061 | 144.37281 | 614 | 57.905753 | 1.3180205 | 9.46E-104 | 3.06E-105 | up |
| VIT_14s0066g00300.t01 | 592 | Putative uncharacterized protein | 400 | 45.627085 | 119 | 18.274926 | 1.3200249 | 5.32E-21 | 7.31E-22 | up |
| VIT_19s0014g02800.t01 | 625 | calsequestrin-1-like | 232 | 25.066426 | 69 | 10.036897 | 1.320443 | 1.22E-12 | 2.48E-13 | up |
| VIT_03s0091g00480.t01 | 1457 | E3 ubiquitin-protein ligase RNF115 | 1241 | 57.517059 | 369 | 23.02487 | 1.3207969 | 4.89E-63 | 2.54E-64 | up |
| VIT_01s0011g01270.t01 | 622 | Putative uncharacterized protein | 111 | 12.050832 | 33 | 4.8234072 | 1.3210083 | 1.24E-06 | 4.09E-07 | up |
| VIT_06s0004g03390.t01 | 1115 | tetraspanin-3 | 767 | 46.452056 | 228 | 18.590469 | 1.3211793 | 2.30E-39 | 1.88E-40 | up |
| VIT_08s0007g01620.t01 | 1449 | L-type lectin-domain containing receptor kinase VIII.2-like | 1612 | 75.124414 | 479 | 30.053669 | 1.3217407 | 1.07E-81 | 4.40E-83 | up |
| VIT_10s0003g01580.t01 | 962 | Putative uncharacterized protein | 155 | 10.880305 | 46 | 4.3472351 | 1.323549 | 7.62E-09 | 2.05E-09 | up |
| VIT_08s0056g00260.t01 | 1680 | Putative uncharacterized protein | 209 | 8.4008155 | 62 | 3.3551565 | 1.3241493 | 1.51E-11 | 3.34E-12 | up |
| VIT_18s0072g00170.t01 | 1521 | phytosulfokine receptor 2 | 1811 | 80.403264 | 537 | 32.097813 | 1.3247791 | 5.47E-92 | 1.99E-93 | up |
| VIT_01s0011g02360.t01 | 501 | RING-H2 finger protein ATL51-like | 108 | 14.556953 | 32 | 5.806877 | 1.325874 | 1.63E-06 | 5.44E-07 | up |
| VIT_04s0044g00020.t01 | 1763 | catalase | 25877 | 991.16523 | 7656 | 394.80258 | 1.3279941 | 0 | 0 | up |
| VIT_13s0019g00340.t01 | 1290 | glutaredoxin (GRX) | 1441 | 75.432537 | 426 | 30.022736 | 1.3291315 | 9.48E-74 | 4.28E-75 | up |
| VIT_09s0002g08680.t01 | 685 | ankyrin repeat-containing protein | 1140 | 112.38251 | 337 | 44.726993 | 1.3291998 | 1.51E-58 | 8.47E-60 | up |
| VIT_08s0040g01380.t01 | 606 | acylphosphatase-like | 159 | 17.717765 | 47 | 7.0510794 | 1.3292806 | 4.25E-09 | 1.12E-09 | up |
| VIT_01s0011g05810.t01 | 2153 | adagio protein 3-like | 1509 | 47.329253 | 446 | 18.833074 | 1.3294637 | 3.28E-77 | 1.40E-78 | up |
| VIT_02s0012g00170.t01 | 1908 | beta-amylase 3, chloroplastic-like | 606 | 21.4476 | 179 | 8.5291359 | 1.3303447 | 1.22E-31 | 1.20E-32 | up |
| VIT_04s0044g00750.t01 | 1460 | G-type lectin S-receptor-like serine/threonine-protein kinase RLK1 | 342 | 15.818223 | 101 | 6.2892505 | 1.3306275 | 2.52E-18 | 3.88E-19 | up |
| VIT_19s0015g02890.t01 | 869 | glutathione S-transferase | 105 | 8.15932 | 31 | 3.2431892 | 1.3310357 | 2.15E-06 | 7.24E-07 | up |
| VIT_08s0007g01360.t01 | 480 | Putative uncharacterized protein | 271 | 38.125232 | 80 | 15.15232 | 1.3312075 | 9.33E-15 | 1.71E-15 | up |
| VIT_06s0009g01070.t01 | 674 | Putative uncharacterized protein | 593 | 59.412693 | 175 | 23.605246 | 1.3316637 | 4.99E-31 | 4.98E-32 | up |
| VIT_00s0322g00020.t01 | 1244 | ADIPOR-like receptor CG5315-like | 322 | 17.479135 | 95 | 6.9427831 | 1.3320478 | 2.42E-17 | 3.89E-18 | up |
| VIT_14s0108g00190.t01 | 1401 | aldose 1-epimerase-like | 234 | 11.278781 | 69 | 4.4775591 | 1.3328268 | 6.59E-13 | 1.32E-13 | up |
| VIT_10s0003g03260.t01 | 647 | prefoldin subunit 6-like | 156 | 16.281888 | 46 | 6.4637407 | 1.3328268 | 5.58E-09 | 1.49E-09 | up |
| VIT_01s0010g03610.t01 | 563 | Putative uncharacterized protein | 1265 | 151.72829 | 373 | 60.232489 | 1.3328764 | 3.91E-65 | 1.99E-66 | up |
| VIT_04s0008g01020.t01 | 667 | Putative uncharacterized protein | 112 | 11.339049 | 33 | 4.4979899 | 1.3339473 | 9.14E-07 | 2.96E-07 | up |
| VIT_18s0117g00340.t01 | 1234 | transcription factor TCP8-like | 241 | 13.188224 | 71 | 5.2308656 | 1.3341287 | 2.79E-13 | 5.48E-14 | up |

| | | | | | | | | | | |
|---|---|---|---|---|---|---|---|---|---|---|
| VIT_10s0003g04210.t01 | 1533 | phospholipase A1-Igamma1 | 129 | 5.6824026 | 38 | 2.253574 | 1.3342863 | 1.26E-07 | 3.75E-08 | up |
| VIT_08s0007g00760.t01 | 1008 | thaumatin-like protein | 129 | 8.6419873 | 38 | 3.4273104 | 1.3342863 | 1.26E-07 | 3.75E-08 | up |
| VIT_16s0050g00390.t01 | 2417 | peroxisomal-coenzyme A synthetase-lik | 384 | 10.7285 | 113 | 4.2504231 | 1.33577 | 1.48E-20 | 2.07E-21 | up |
| VIT_05s0020g00970.t01 | 793 | putative esterase HI_1161-like | 1659 | 141.2725 | 488 | 55.947026 | 1.3363473 | 1.82E-85 | 7.10E-87 | up |
| VIT_17s0000g01180.t01 | 830 | mediator of RNA polymerase II transcription subunit 30 | 68 | 5.5324215 | 20 | 2.1906968 | 1.3365213 | 0.00015045 | 6.33E-05 | up |
| VIT_11s0052g00590.t01 | 641 | Putative uncharacterized protein | 691 | 72.795488 | 203 | 28.791771 | 1.3381925 | 2.98E-36 | 2.62E-37 | up |
| VIT_02s0025g04420.t01 | 1955 | MATE efflux family protein | 521 | 17.995976 | 153 | 7.1150022 | 1.3387382 | 1.34E-27 | 1.46E-28 | up |
| VIT_18s0001g12520.t01 | 3682 | probable LRR receptor-like serine/threonine-protein kinase | 1874 | 34.369265 | 550 | 13.580297 | 1.3396039 | 8.80E-97 | 3.02E-98 | up |
| VIT_07s0031g02300.t01 | 1023 | eukaryotic translation initiation factor 6-2-like | 1708 | 112.74484 | 501 | 44.523825 | 1.340412 | 2.26E-88 | 8.56E-90 | up |
| VIT_07s0005g02020.t01 | 633 | Putative uncharacterized protein | 116 | 12.374815 | 34 | 4.883212 | 1.3415047 | 5.11E-07 | 1.61E-07 | up |
| VIT_04s0079g00440.t01 | 1740 | Putative uncharacterized protein | 331 | 12.84586 | 97 | 5.0681897 | 1.3417611 | 5.51E-18 | 8.61E-19 | up |
| VIT_08s0040g00490.t01 | 1104 | metacaspase-9-like | 140 | 8.5633443 | 41 | 3.3763321 | 1.3427175 | 2.99E-08 | 8.42E-09 | up |
| VIT_18s0001g09990.t01 | 1685 | cysteine proteinase RD21a-like | 5175 | 207.39338 | 1515 | 81.741594 | 1.3432276 | 5.66E-267 | 7.03E-269 | up |
| VIT_01s0011g05740.t01 | 751 | UDP-3-O-[3-hydroxymyristoyl] N-acetylglucosamine deacetylase-like | 82 | 7.3732398 | 24 | 2.9053715 | 1.343576 | 2.70E-05 | 1.03E-05 | up |
| VIT_08s0007g07620.t01 | 1448 | thioredoxin | 2156 | 100.54596 | 631 | 39.617874 | 1.3436318 | 1.00E-111 | 3.00E-113 | up |
| VIT_18s0122g00460.t01 | 623 | calvin cycle protein CP12-like | 797 | 86.388258 | 233 | 34.001513 | 1.3452363 | 5.54E-42 | 4.26E-43 | up |
| VIT_00s0184g00010.t01 | 651 | ethylene-responsive transcription factor-like protein | 65 | 6.7424357 | 19 | 2.6534016 | 1.3454268 | 0.00019849 | 8.47E-05 | up |
| VIT_16s0100g01210.t01 | 749 | Putative uncharacterized protein | 616 | 55.537118 | 180 | 21.848472 | 1.34592 | 1.05E-32 | 1.00E-33 | up |
| VIT_00s0662g00040.t01 | 1456 | ethylene-responsive transcription factor RAP2-4-like | 1028 | 47.677797 | 300 | 18.732263 | 1.3477924 | 5.22E-54 | 3.14E-55 | up |
| VIT_12s0059g00880.t01 | 1905 | WRKY transcription factor 31 | 168 | 5.9552328 | 49 | 2.3384682 | 1.3485941 | 9.69E-10 | 2.44E-10 | up |
| VIT_08s0007g03110.t01 | 1674 | Putative uncharacterized protein | 4258 | 171.76499 | 1240 | 67.343643 | 1.3508223 | 1.14E-221 | 1.65E-223 | up |
| VIT_09s0002g08430.t01 | 2161 | probable transporter mch1 | 546 | 17.061701 | 159 | 6.6891776 | 1.3508607 | 2.91E-29 | 3.03E-30 | up |
| VIT_01s0026g01060.t01 | 2185 | probable inactive receptor kinase | 862 | 26.640371 | 251 | 10.443658 | 1.350987 | 1.28E-45 | 9.21E-47 | up |
| VIT_18s0041g00740.t01 | 1727 | anthocyanidin 5,3-O-glucosyltransferase-like | 251 | 9.8144468 | 73 | 3.842916 | 1.3527055 | 4.64E-14 | 8.74E-15 | up |
| VIT_14s0006g00350.t01 | 1216 | glutamine synthetase | 6893 | 382.78873 | 2004 | 149.82853 | 1.353236 | 0 | 0 | up |
| VIT_06s0080g00910.t01 | 1233 | F-box protein | 117 | 6.4077746 | 34 | 2.5069531 | 1.3538884 | 3.74E-07 | 1.16E-07 | up |
| VIT_08s0105g00270.t01 | 1044 | NC domain-containing protein | 2900 | 187.57802 | 842 | 73.323294 | 1.3551473 | 5.96E-152 | 1.29E-153 | up |
| VIT_06s0004g03420.t01 | 1069 | dof zinc finger protein DOF2.1-like | 393 | 24.825573 | 114 | 9.6952167 | 1.356482 | 1.73E-21 | 2.33E-22 | up |
| VIT_19s0027g00390.t01 | 1727 | probable LRR receptor-like serine/threonine-protein kinase | 166 | 6.4908294 | 48 | 2.5268489 | 1.3610634 | 9.29E-10 | 2.33E-10 | up |
| VIT_04s0044g02010.t01 | 929 | gibberellin 3-beta-dioxygenase 4-like | 173 | 12.575198 | 50 | 4.8931064 | 1.3617585 | 3.96E-10 | 9.65E-11 | up |
| VIT_11s0149g00350.t01 | 1101 | chitotriosidase-1 | 263 | 16.130687 | 76 | 6.2756201 | 1.361978 | 7.95E-15 | 1.45E-15 | up |
| VIT_11s0118g00300.t01 | 738 | actin-related protein 2/3 complex subunit 3-like | 135 | 12.352699 | 39 | 4.804394 | 1.3623999 | 3.77E-08 | 1.07E-08 | up |
| VIT_04s0008g06000.t01 | 1017 | protein PPLZ02 | 336 | 22.310164 | 97 | 8.6712389 | 1.3633911 | 1.15E-18 | 1.74E-19 | up |
| VIT_01s0011g04830.t01 | 744 | Putative uncharacterized protein | 2269 | 205.94251 | 655 | 80.038462 | 1.3634763 | 3.49E-120 | 9.67E-122 | up |
| VIT_08s0058g01020.t01 | 2677 | protein WEAK CHLOROPLAST MOVEMENT UNDER BLUE LIGHT 1-like | 10640 | 268.39703 | 3066 | 104.12479 | 1.3660551 | 0 | 0 | up |
| VIT_07s0005g02080.t01 | 758 | Putative uncharacterized protein | 125 | 11.135898 | 36 | 4.3178114 | 1.3668458 | 1.16E-07 | 3.44E-08 | up |
| VIT_12s0028g01410.t01 | 1501 | heat stress transcription factor A-4b | 205 | 9.22269 | 59 | 3.573565 | 1.3678236 | 7.24E-12 | 1.56E-12 | up |
| VIT_15s0046g00450.t01 | 894 | Putative uncharacterized protein | 1067 | 80.595602 | 307 | 31.21988 | 1.3682361 | 2.65E-57 | 1.51E-58 | up |
| VIT_14s0066g01880.t01 | 596 | zinc finger AN1 domain-containing stress-associated protein 12-like | 73 | 8.2710576 | 21 | 3.2033427 | 1.3684936 | 6.13E-05 | 2.44E-05 | up |
| VIT_00s2207g00010.t01 | 434 | probable UDP-N-acetylglucosamine pyrophosphorylase-like | 80 | 12.447574 | 23 | 4.8180187 | 1.3693526 | 2.59E-05 | 9.87E-06 | up |
| VIT_06s0004g04110.t01 | 1154 | Putative uncharacterized protein | 87 | 5.0909389 | 25 | 1.9695389 | 1.3700738 | 1.10E-05 | 4.00E-06 | up |
| VIT_13s0019g03910.t01 | 1135 | Putative uncharacterized protein | 87 | 5.1761617 | 25 | 2.0025092 | 1.3700738 | 1.10E-05 | 4.00E-06 | up |
| VIT_11s0037g00440.t01 | 1536 | hydroxycinnamoyl-Coenzyme A shikimate/quinate hydroxycinnamoyltransferase-like | 596 | 26.202304 | 171 | 10.121276 | 1.3723025 | 1.34E-32 | 1.28E-33 | up |
| VIT_01s0011g01920.t01 | 1016 | hypothetical protein | 129 | 8.5739401 | 37 | 3.3108415 | 1.3727604 | 6.47E-08 | 1.87E-08 | up |
| VIT_17s0000g02520.t01 | 1147 | Putative uncharacterized protein | 2217 | 130.5229 | 635 | 50.331593 | 1.3747668 | 6.25E-119 | 1.77E-120 | up |
| VIT_02s0025g01050.t01 | 679 | ubiquitin-conjugating enzyme E2-17 kDa-like | 6915 | 687.7124 | 1978 | 264.84202 | 1.3766733 | 0 | 0 | up |
| VIT_04s0043g00370.t01 | 1701 | ammonium transporter 1 member 2-like | 322 | 12.783095 | 92 | 4.9171549 | 1.3783414 | 3.24E-18 | 5.01E-19 | up |
| VIT_05s0077g02270.t01 | 1218 | Putative uncharacterized protein | 389 | 21.566852 | 111 | 8.2852585 | 1.380197 | 8.01E-22 | 1.07E-22 | up |
| VIT_06s0004g00160.t01 | 1060 | Putative uncharacterized protein | 463 | 29.495759 | 132 | 11.321356 | 1.3814608 | 8.34E-26 | 9.65E-27 | up |
| VIT_01s0137g00600.t01 | 3152 | protein-tyrosine-phosphatase MKP1-like | 530 | 11.354659 | 151 | 4.3553304 | 1.3824303 | 2.03E-29 | 2.11E-30 | up |
| VIT_08s0040g02760.t01 | 2030 | ankyrin repeat-containing protein | 172 | 5.7215916 | 49 | 2.1944739 | 1.3825414 | 2.76E-10 | 6.64E-11 | up |
| VIT_16s0039g02360.t01 | 1528 | probable disease resistance protein | 137 | 6.054547 | 39 | 2.3204469 | 1.3836164 | 2.00E-08 | 5.54E-09 | up |
| VIT_14s0083g00160.t01 | 670 | cyclin-B1-2-like | 2568 | 258.82407 | 731 | 99.191155 | 1.3836884 | 5.73E-139 | 1.35E-140 | up |
| VIT_16s0013g02150.t01 | 1251 | Putative uncharacterized protein | 130 | 7.0173071 | 37 | 2.6889008 | 1.383901 | 4.71E-08 | 1.35E-08 | up |
| VIT_08s0058g01110.t01 | 1350 | transcription repressor OFP1 | 225 | 11.254681 | 64 | 4.3099931 | 1.3847677 | 3.88E-13 | 7.66E-14 | up |
| VIT_05s0049g00180.t01 | 987 | carnitinyl-CoA dehydratase-like | 1516 | 103.72095 | 431 | 39.699998 | 1.3854965 | 1.45E-82 | 5.88E-84 | up |
| VIT_11s0016g01020.t01 | 1892 | flavonoid 3'-monooxygenase-like | 3001 | 107.10982 | 853 | 40.988146 | 1.3858122 | 1.18E-162 | 2.43E-164 | up |
| VIT_19s0014g01180.t01 | 849 | pathogenesis-related protein PR-1 | 190 | 15.112293 | 54 | 5.7825107 | 1.3859546 | 2.78E-11 | 6.22E-12 | up |
| VIT_02s0025g04860.t01 | 710 | geraniol 8-hydroxylase-like | 88 | 8.3696783 | 25 | 3.2011943 | 1.3865619 | 7.99E-06 | 2.87E-06 | up |
| VIT_11s0118g00480.t01 | 1977 | insulin-like growth factor-binding protein complex acid labile subunit-like | 169 | 5.7725071 | 48 | 2.2073182 | 1.3869034 | 3.61E-10 | 8.77E-11 | up |
| VIT_06s0004g08200.t01 | 468 | ubiquitin-conjugating enzyme E2 28-like | 1500 | 216.43617 | 426 | 82.754976 | 1.3870237 | 7.70E-82 | 3.16E-83 | up |
| VIT_18s0122g00180.t01 | 779 | calcium-binding protein CML37-like | 3717 | 322.21039 | 1054 | 123.00805 | 1.3892503 | 4.98E-202 | 8.03E-204 | up |
| VIT_06s0004g07210.t01 | 1199 | zinc finger protein CONSTANS-LIKE 7-like | 321 | 18.078829 | 91 | 6.9000555 | 1.3896214 | 2.24E-18 | 3.45E-19 | up |
| VIT_04s0008g02820.t01 | 1141 | glycosyltransferase family protein 64 C3 | 180 | 10.652985 | 51 | 4.063637 | 1.3904143 | 8.55E-11 | 1.98E-11 | up |
| VIT_07s0031g02100.t01 | 245 | Putative uncharacterized protein | 60 | 16.537491 | 17 | 6.3083127 | 1.3904143 | 0.00024858 | 0.0001077 | up |
| VIT_14s0060g00120.t01 | 733 | Germin-like protein 2 | 1264 | 116.4468 | 357 | 44.278675 | 1.394987 | 1.04E-69 | 5.02E-71 | up |
| VIT_18s0001g10800.t01 | 1144 | probable carboxylesterase 2-like | 3871 | 228.49757 | 1093 | 86.860937 | 1.3953994 | 8.93E-212 | 1.36E-213 | up |
| VIT_10s0003g03120.t01 | 1788 | urease accessory protein G | 2172 | 82.030763 | 613 | 31.169033 | 1.3960516 | 2.89E-119 | 8.16E-121 | up |
| VIT_09s0002g02630.t01 | 1701 | IAA-amino acid hydrolase ILR1-like 4 | 1024 | 40.651829 | 289 | 15.44628 | 1.3960608 | 1.02E-56 | 5.92E-58 | up |
| VIT_01s0011g02200.t01 | 1404 | glutamine synthetase | 1546 | 74.35785 | 436 | 28.232527 | 1.3971268 | 3.05E-85 | 1.19E-86 | up |
| VIT_04s0023g01320.t01 | 1260 | minor allergen Alt a 7-like | 2401 | 128.67852 | 677 | 48.848192 | 1.3973942 | 6.22E-132 | 1.55E-133 | up |
| VIT_07s0104g01180.t01 | 520 | Putative uncharacterized protein | 71 | 9.220181 | 20 | 3.4966891 | 1.3988055 | 5.83E-05 | 2.32E-05 | up |
| VIT_04s0159g00040.t01 | 761 | Glutathione S-transferase | 1825 | 161.94318 | 514 | 61.405721 | 1.3990427 | 1.13E-100 | 3.78E-102 | up |
| VIT_03s0063g01380.t01 | 805 | Putative uncharacterized protein | 679 | 56.958473 | 191 | 21.57088 | 1.4008254 | 3.86E-38 | 3.23E-39 | up |
| VIT_01s0010g03600.t01 | 888 | REF/SRPP-like protein | 8925 | 678.7029 | 2510 | 256.97515 | 1.4011513 | 0 | 0 | up |
| VIT_03s0063g02460.t01 | 966 | Putative uncharacterized protein | 395 | 27.612416 | 111 | 10.44663 | 1.4022795 | 1.17E-22 | 1.52E-23 | up |
| VIT_05s0094g00470.t01 | 992 | Putative uncharacterized protein | 121 | 8.2367928 | 34 | 3.1160012 | 1.4023869 | 1.05E-07 | 3.11E-08 | up |
| VIT_03s0038g01410.t01 | 1228 | probable aquaporin TIP3-2 | 267 | 14.68241 | 75 | 5.5525601 | 1.4028638 | 1.13E-15 | 1.96E-16 | up |
| VIT_07s0031g01020.t01 | 1233 | trafficking protein particle complex subunit 12-like | 912 | 49.947782 | 256 | 18.875882 | 1.4038765 | 4.79E-51 | 3.09E-52 | up |
| VIT_14s0108g01550.t01 | 1795 | BAG family molecular chaperone regulator 8 | 481 | 18.09527 | 135 | 6.837537 | 1.404064 | 2.08E-27 | 2.29E-28 | up |
| VIT_08s0058g01160.t01 | 535 | indole-3-acetic acid-induced protein ARG7-like | 82 | 10.350099 | 23 | 3.9084488 | 1.4049766 | 1.37E-05 | 5.04E-06 | up |
| VIT_14s0083g00960.t01 | 15338 | auxin transport protein BIG-like | 2175 | 9.5757979 | 610 | 3.6156924 | 1.4051208 | 1.36E-120 | 3.76E-122 | up |
| VIT_06s0004g04430.t01 | 712 | ubiquitin-conjugating enzyme E2 7-like | 403 | 38.221656 | 113 | 14.428754 | 1.4054436 | 3.60E-23 | 4.58E-24 | up |
| VIT_01s0011g00060.t01 | 603 | Putative uncharacterized protein | 1591 | 178.17112 | 446 | 67.24313 | 1.4058047 | 1.63E-88 | 6.14E-90 | up |
| VIT_11s0103g00640.t01 | 247 | Putative uncharacterized protein | 339 | 92.680248 | 95 | 34.966891 | 1.4062724 | 1.12E-19 | 1.63E-20 | up |
| VIT_05s0094g01590.t01 | 1291 | F-box/kelch-repeat protein SKIP20-like | 1871 | 97.866034 | 524 | 36.900769 | 1.4071574 | 4.38E-104 | 1.41E-105 | up |
| VIT_16s0039g01550.t01 | 690 | putative gamma-glutamylcyclotransferase | 175 | 17.126689 | 49 | 6.4562057 | 1.4074878 | 1.06E-10 | 2.47E-11 | up |
| VIT_12s0028g00610.t01 | 2155 | aspartic proteinase nepenthesin-2 | 793 | 24.849082 | 222 | 9.3656101 | 1.4077477 | 1.06E-44 | 7.77E-46 | up |
| VIT_04s0008g01530.t01 | 1493 | 17.9 kDa class II heat shock protein-like | 261 | 11.804977 | 73 | 4.4452217 | 1.4090679 | 1.92E-15 | 3.38E-16 | up |
| VIT_09s0002g02400.t01 | 895 | PRA1 family protein F2-like | 2410 | 181.83541 | 674 | 68.464783 | 1.4091992 | 4.16E-134 | 1.01E-135 | up |
| VIT_15s0046g01190.t01 | 1601 | hydroquinone glucosyltransferase-like | 3787 | 159.73071 | 1059 | 60.136064 | 1.4093393 | 2.50E-210 | 3.85E-212 | up |
| VIT_06s0004g03330.t01 | 875 | LOB domain-containing protein 1 | 154 | 11.884943 | 43 | 4.4677697 | 1.4115083 | 1.37E-09 | 3.49E-10 | up |
| VIT_02s0154g00480.t01 | 912 | 23.6 kDa heat shock protein | 351 | 25.989428 | 98 | 9.7692587 | 1.4116039 | 1.90E-20 | 2.68E-21 | up |
| VIT_09s0002g06360.t01 | 593 | Putative uncharacterized protein | 86 | 9.7932807 | 24 | 3.679484 | 1.4122888 | 7.58E-06 | 2.71E-06 | up |
| VIT_07s0151g00730.t01 | 517 | protein ENHANCED DISEASE RESISTANCE 2-like | 104 | 13.583986 | 29 | 5.0996201 | 1.4134452 | 7.60E-07 | 2.44E-07 | up |
| VIT_18s0001g05400.t01 | 1643 | Putative uncharacterized protein | 165 | 6.781579 | 46 | 2.5453684 | 1.4137468 | 3.25E-10 | 7.86E-11 | up |
| VIT_04s0008g00470.t01 | 781 | early nodulin-93-like | 1307 | 113.00795 | 364 | 42.372172 | 1.4152353 | 1.30E-73 | 5.91E-75 | up |

| | | | | | | | | | | |
|---|---|---|---|---|---|---|---|---|---|---|
| VIT_17s0000g09170.t01 | 2228 | L-type lectin-domain containing receptor kinase S.1 | 844 | 25.580658 | 235 | 9.5892148 | 1.4155688 | 6.71E-48 | 4.60E-49 | up |
| VIT_07s0005g00060.t01 | 413 | Putative uncharacterized protein | 194 | 31.720215 | 54 | 11.88705 | 1.4160118 | 7.72E-12 | 1.66E-12 | up |
| VIT_02s0025g01460.t01 | 595 | Putative uncharacterized protein | 115 | 13.051647 | 32 | 4.889488 | 1.4164766 | 1.79E-07 | 5.41E-08 | up |
| VIT_09s0018g00620.t01 | 735 | chaperone protein dnaJ 8 | 996 | 91.507448 | 277 | 34.262796 | 1.4172463 | 1.93E-56 | 1.12E-57 | up |
| VIT_02s0025g00130.t01 | 1124 | Putative uncharacterized protein | 3277 | 196.87681 | 911 | 73.685568 | 1.4178392 | 9.08E-184 | 1.66E-185 | up |
| VIT_18s0001g13030.t01 | 276 | ocs element-binding factor 1-like | 281 | 68.751421 | 78 | 25.693064 | 1.4200106 | 1.01E-16 | 1.66E-17 | up |
| VIT_09s0018g00610.t01 | 1023 | cytochrome c oxidase assembly factor 5 | 281 | 18.548771 | 78 | 6.931853 | 1.4200106 | 1.01E-16 | 1.66E-17 | up |
| VIT_00s0207g00130.t01 | 666 | STS14 protein-like | 220 | 22.306575 | 61 | 8.3269504 | 1.4216089 | 2.39E-13 | 4.67E-14 | up |
| VIT_03s0180g00080.t01 | 1113 | kynurenine formamidase-like | 426 | 25.84633 | 118 | 9.6386723 | 1.4230531 | 7.13E-25 | 8.51E-26 | up |
| VIT_18s0001g07770.t01 | 782 | Putative uncharacterized protein | 867 | 74.868096 | 240 | 27.90197 | 1.4239841 | 1.31E-49 | 8.67E-51 | up |
| VIT_14s0068g01870.t01 | 2226 | Putative uncharacterized protein | 1815 | 55.059963 | 502 | 20.5026 | 1.4251968 | 6.09E-103 | 1.99E-104 | up |
| VIT_01s0026g00880.t01 | 1386 | vegetative incompatibility protein HET-E-1 | 1286 | 62.65593 | 355 | 23.286032 | 1.4279862 | 2.03E-73 | 9.27E-75 | up |
| VIT_06s0004g06240.t01 | 1460 | Putative uncharacterized protein | 3275 | 151.47567 | 904 | 56.291905 | 1.4280867 | 1.19E-185 | 2.15E-187 | up |
| VIT_11s0016g02910.t01 | 1037 | acid phosphatase 1-like | 2334 | 151.98703 | 644 | 56.459559 | 1.4286585 | 1.33E-132 | 3.29E-134 | up |
| VIT_14s0108g01370.t01 | 1434 | probable mitochondrial saccharopine dehydrogenase | 1033 | 48.644709 | 285 | 18.068666 | 1.4287929 | 3.30E-59 | 1.83E-60 | up |
| VIT_04s0008g01520.t01 | 532 | 17.1 kDa class II heat shock protein | 370 | 46.965023 | 102 | 17.430864 | 1.4299426 | 6.78E-22 | 9.00E-23 | up |
| VIT_18s0001g07690.t01 | 779 | Putative uncharacterized protein | 810 | 70.21534 | 223 | 26.025422 | 1.4318647 | 8.59E-47 | 6.00E-48 | up |
| VIT_08s0007g05700.t01 | 1013 | ganglioside-induced differentiation-associated-protein 2-like | 959 | 63.928366 | 264 | 23.693262 | 1.4319794 | 3.22E-55 | 1.91E-56 | up |
| VIT_13s0019g03000.t01 | 752 | 18.2 kDa class I heat shock protein-like | 287 | 25.772022 | 79 | 9.5507972 | 1.4321127 | 2.90E-17 | 4.68E-18 | up |
| VIT_09s0002g02750.t01 | 1071 | vacuolar protein sorting-associated protein 32 homolog 2-like | 218 | 13.745213 | 60 | 5.0932167 | 1.4322802 | 2.25E-13 | 4.39E-14 | up |
| VIT_19s0015g00050.t01 | 4826 | ABC transporter C family member 3-like | 567 | 7.9337806 | 156 | 2.9387839 | 1.4327892 | 4.31E-33 | 4.09E-34 | up |
| VIT_05s0020g02150.t01 | 623 | NADH dehydrogenase [ubiquinone] 1 alpha subcomplex subunit 8-B-like | 1950 | 211.36399 | 536 | 78.218074 | 1.4341557 | 1.43E-111 | 4.30E-113 | up |
| VIT_16s0100g01250.t01 | 532 | putative uncharacterized protein DDB | 51 | 6.4735572 | 14 | 2.3924715 | 1.4360569 | 0.00055377 | 0.00025373 | up |
| VIT_02s0012g02220.t01 | 1291 | probable xyloglucan endotransglucosylase/hydrolase protein 30-like | 306 | 16.005883 | 84 | 5.9153905 | 1.4360569 | 2.09E-18 | 3.21E-19 | up |
| VIT_02s0025g05050.t01 | 964 | zinc finger A20 and AN1 domain-containing stress-associated protein 1-like | 1207 | 84.550208 | 331 | 31.216293 | 1.4375091 | 1.19E-69 | 5.73E-71 | up |
| VIT_02s0012g01010.t01 | 1845 | leucine-rich repeat protein soc-2-like | 310 | 11.346183 | 85 | 4.1884461 | 1.43772 | 1.16E-18 | 1.75E-19 | up |
| VIT_00s0370g00070.t01 | 1105 | epoxide hydrolase 2-like | 460 | 28.11124 | 126 | 10.366655 | 1.4391966 | 3.25E-27 | 3.60E-28 | up |
| VIT_09s0002g04020.t01 | 1048 | aquaporin TIP2;1 | 10234 | 659.42981 | 2803 | 243.16003 | 1.439313 | 0 | 0 | up |
| VIT_01s0010g01140.t01 | 751 | uncharacterized N-acetyltransferase ycf52 | 409 | 36.776281 | 112 | 13.5584 | 1.4395886 | 2.53E-24 | 3.07E-25 | up |
| VIT_14s0006g00640.t01 | 2251 | DELLA protein GAI1-like | 8964 | 268.91238 | 2453 | 99.072341 | 1.4405819 | 0 | 0 | up |
| VIT_06s0004g05830.t01 | 535 | DNA-directed RNA polymerases I and III subunit RPAC2-like | 106 | 13.379397 | 29 | 4.9280441 | 1.440926 | 3.97E-07 | 1.24E-07 | up |
| VIT_02s0025g03010.t01 | 636 | heavy metal-associated domain containing protein, expressed | 117 | 12.42262 | 32 | 4.5742852 | 1.4413512 | 9.36E-08 | 2.75E-08 | up |
| VIT_19s0014g02860.t01 | 1818 | Putative uncharacterized protein | 432 | 16.046278 | 118 | 5.9009033 | 1.443231 | 1.00E-25 | 1.16E-26 | up |
| VIT_05s0077g00570.t01 | 1045 | F-box protein | 227 | 14.668781 | 62 | 5.3939358 | 1.4433387 | 4.95E-14 | 9.35E-15 | up |
| VIT_07s0031g01580.t01 | 1398 | F-box/kelch-repeat protein SKIP6-like | 165 | 7.9700531 | 45 | 2.9264137 | 1.4454556 | 1.59E-10 | 3.77E-11 | up |
| VIT_06s0009g03450.t01 | 1212 | LATERAL ROOT PRIMORDIUM 1 | 132 | 7.3545441 | 36 | 2.7004134 | 1.4454556 | 1.22E-08 | 3.33E-09 | up |
| VIT_00s0291g00020.t01 | 669 | serine-rich protein, putative, expressed | 165 | 16.654909 | 45 | 6.1152859 | 1.4454556 | 1.59E-10 | 3.77E-11 | up |
| VIT_11s0016g05190.t01 | 1375 | probable sugar phosphate/phosphate translocator | 9460 | 464.59323 | 2578 | 170.45533 | 1.4465744 | 0 | 0 | up |
| VIT_16s0050g01990.t01 | 709 | hypothetical protein | 2194 | 208.96562 | 597 | 76.55234 | 1.4487472 | 2.50E-127 | 6.53E-129 | up |
| VIT_13s0101g00060.t01 | 528 | Putative uncharacterized protein | 272 | 34.787196 | 74 | 12.741723 | 1.448996 | 1.09E-16 | 1.80E-17 | up |
| VIT_11s0118g00570.t01 | 941 | serine-rich protein, putative, expressed | 1213 | 87.047363 | 330 | 31.88267 | 1.4490281 | 7.96E-71 | 3.78E-72 | up |
| VIT_08s0058g00450.t01 | 1310 | mitoferrin-like | 901 | 46.44489 | 245 | 17.002985 | 1.4497319 | 6.85E-53 | 4.24E-54 | up |
| VIT_17s0000g05680.t01 | 1211 | hypersensitive-induced response protein | 3009 | 167.78861 | 818 | 61.410062 | 1.4500979 | 1.43E-174 | 2.70E-176 | up |
| VIT_08s0007g00800.t01 | 1334 | glucose-induced degradation protein 4 | 431 | 21.817545 | 117 | 7.9737094 | 1.4521658 | 6.75E-26 | 7.79E-27 | up |
| VIT_00s1937g00010.t01 | 1412 | amine oxidase | 365 | 17.455915 | 99 | 6.3742761 | 1.4533825 | 4.02E-22 | 5.31E-23 | up |
| VIT_01s0011g05920.t01 | 1292 | S-adenosylmethionine-dependent methyltransferase | 155 | 8.1012797 | 42 | 2.955406 | 1.4547935 | 4.87E-10 | 1.19E-10 | up |
| VIT_01s0010g03680.t01 | 1069 | glucuronoxylan 4-O-methyltransferase 1 | 85 | 5.3693988 | 23 | 1.9560525 | 1.4568155 | 5.14E-06 | 1.80E-06 | up |
| VIT_13s0067g00610.t01 | 534 | protein yippee-like | 895 | 113.1791 | 242 | 41.200689 | 1.4578671 | 5.60E-53 | 3.46E-54 | up |
| VIT_08s0040g00030.t01 | 245 | ubiquitin-conjugating enzyme-like | 714 | 196.79614 | 193 | 71.617902 | 1.4583097 | 1.73E-42 | 1.32E-43 | up |
| VIT_07s0005g00260.t01 | 5063 | ABC transporter C family member 4-like | 2119 | 28.262298 | 572 | 10.271136 | 1.460283 | 1.82E-124 | 4.91E-126 | up |
| VIT_19s0015g00230.t01 | 1316 | zinc transporter 11-like | 1223 | 62.755965 | 330 | 22.797563 | 1.460873 | 2.90E-72 | 1.35E-73 | up |
| VIT_01s0011g01760.t01 | 534 | phloem protein 2-LIKE A9 | 178 | 22.509362 | 48 | 8.1720375 | 1.4617574 | 1.95E-11 | 4.32E-12 | up |
| VIT_11s0149g00380.t01 | 1104 | chitotriosidase-1 | 141 | 8.624511 | 38 | 3.1292834 | 1.4626103 | 2.68E-09 | 6.95E-10 | up |
| VIT_18s0001g11690.t01 | 1118 | mediator of RNA polymerase II transcription subunit 18-like | 141 | 8.5165118 | 38 | 3.0900974 | 1.4626103 | 2.68E-09 | 6.95E-10 | up |
| VIT_07s0005g01050.t01 | 681 | Putative uncharacterized protein | 846 | 83.889517 | 228 | 30.43814 | 1.4626103 | 2.25E-50 | 1.47E-51 | up |
| VIT_00s2750g00010.t01 | 479 | phosphatidate cytidylyltransferase | 156 | 21.992446 | 42 | 7.9715752 | 1.4640713 | 3.50E-10 | 8.49E-11 | up |
| VIT_17s0000g05850.t01 | 3017 | ABC transporter A family member 2-like | 569 | 12.735658 | 153 | 4.6104837 | 1.4658835 | 2.53E-34 | 2.32E-35 | up |
| VIT_02s0025g03660.t01 | 1721 | oxalate--CoA ligase | 923 | 36.2164 | 248 | 13.100902 | 1.466977 | 4.18E-55 | 2.48E-56 | up |
| VIT_00s1308g00020.t01 | 309 | prostamide/prostaglandin F synthase | 67 | 14.642012 | 18 | 5.2959564 | 1.4671507 | 5.15E-05 | 2.03E-05 | up |
| VIT_03s0038g04220.t01 | 972 | dihydroflavonol-4-reductase | 265 | 18.410435 | 71 | 6.6408314 | 1.4710879 | 1.23E-16 | 2.05E-17 | up |
| VIT_14s0083g01140.t01 | 461 | Putative uncharacterized protein | 56 | 8.202978 | 15 | 2.9581535 | 1.4714508 | 0.00021983 | 9.46E-05 | up |
| VIT_17s0000g05600.t01 | 1145 | isopiperitenol/(-)-carveol dehydrogenase | 661 | 38.983463 | 177 | 14.053942 | 1.4718874 | 6.02E-40 | 4.87E-41 | up |
| VIT_00s0120g00010.t01 | 789 | Putative uncharacterized protein | 71 | 6.0766719 | 19 | 2.1893085 | 1.4728061 | 2.84E-05 | 1.09E-05 | up |
| VIT_05s0077g01170.t01 | 623 | DNA-directed RNA polymerase II subunit RPB9-like | 172 | 18.643388 | 46 | 6.7127451 | 1.4736893 | 3.29E-11 | 7.39E-12 | up |
| VIT_13s0106g00790.t01 | 1604 | diphosphomevalonate decarboxylase-like | 397 | 16.713622 | 106 | 6.008027 | 1.4760613 | 1.68E-24 | 2.03E-25 | up |
| VIT_00s0483g00040.t01 | 377 | myosin-11-like | 45 | 8.0603817 | 12 | 2.8938117 | 1.4778771 | 0.00093993 | 0.00044631 | up |
| VIT_14s0083g00590.t01 | 1270 | protein FAM133A | 105 | 5.5830308 | 28 | 2.0044013 | 1.4778771 | 2.66E-07 | 8.17E-08 | up |
| VIT_18s0001g02070.t01 | 762 | Putative uncharacterized protein | 375 | 33.232326 | 100 | 11.93096 | 1.4778771 | 3.03E-23 | 3.84E-24 | up |
| VIT_06s0004g07310.t01 | 1617 | UDP-glycosyltransferase 87A1-like | 240 | 10.022722 | 64 | 3.5983245 | 1.4778771 | 2.88E-15 | 5.13E-16 | up |
| VIT_17s0000g03790.t01 | 1109 | Putative uncharacterized protein | 814 | 49.56525 | 217 | 17.789288 | 1.4783203 | 2.87E-49 | 1.91E-50 | up |
| VIT_05s0029g01060.t01 | 1572 | protein ELC-like | 938 | 40.293477 | 250 | 14.45832 | 1.4786463 | 1.23E-56 | 7.10E-58 | up |
| VIT_18s0075g00120.t01 | 1568 | alpha-(1,4)-fucosyltransferase-like | 413 | 17.786416 | 110 | 6.3778896 | 1.4796248 | 1.55E-25 | 1.82E-26 | up |
| VIT_18s0001g15640.t01 | 1148 | pathogenesis-related protein PR-1 | 3309 | 194.64324 | 881 | 69.769304 | 1.4801679 | 7.64E-198 | 1.26E-199 | up |
| VIT_09s0002g06920.t01 | 859 | protein LURP-one-related 12 | 1962 | 154.23761 | 522 | 55.246874 | 1.4811898 | 9.22E-118 | 2.65E-119 | up |
| VIT_17s0000g06750.t01 | 704 | dCTP pyrophosphatase 1 | 203 | 19.471877 | 54 | 6.9735107 | 1.4814349 | 4.04E-13 | 7.98E-14 | up |
| VIT_03s0063g01870.t01 | 715 | cold-regulated 413 plasma membrane protein 2 | 1992 | 188.13419 | 529 | 67.263584 | 1.4838645 | 7.28E-120 | 2.04E-121 | up |
| VIT_09s0002g03660.t01 | 961 | protein SAWADEE HOMEODOMAIN HOMOLOG 1 | 98 | 6.8863189 | 26 | 2.4596898 | 1.4852566 | 6.25E-07 | 1.99E-07 | up |
| VIT_06s0009g03440.t01 | 670 | GA-binding protein subunit beta-2 | 181 | 18.242662 | 48 | 6.5132359 | 1.4858699 | 7.22E-12 | 1.55E-12 | up |
| VIT_17s0000g09800.t01 | 402 | probable pectate lyase 5-like | 283 | 47.538429 | 75 | 16.961552 | 1.4868261 | 5.88E-18 | 9.19E-19 | up |
| VIT_02s0033g00230.t01 | 1419 | Putative uncharacterized protein | 117 | 5.5678549 | 31 | 1.9861391 | 1.4871549 | 4.51E-08 | 1.29E-08 | up |
| VIT_09s0018g01290.t01 | 716 | Putative uncharacterized protein | 423 | 39.894386 | 112 | 14.221171 | 1.4881454 | 2.42E-26 | 2.76E-27 | up |
| VIT_12s0034g02220.t01 | 2487 | probable receptor-like protein kinase | 306 | 8.3086427 | 81 | 2.9610082 | 1.4885243 | 2.33E-19 | 3.42E-20 | up |
| VIT_05s0029g00220.t01 | 509 | Putative uncharacterized protein | 4866 | 645.5632 | 1288 | 230.05329 | 1.4885902 | 4.30E-293 | 4.94E-295 | up |
| VIT_15s0046g01050.t01 | 1128 | Abscisic acid receptor PYL9 | 2203 | 131.88331 | 583 | 46.98831 | 1.4888882 | 4.01E-133 | 9.87E-135 | up |
| VIT_17s0000g00800.t01 | 1892 | probable S-acyltransferase | 854 | 30.480436 | 226 | 10.859696 | 1.4888998 | 3.52E-52 | 2.22E-53 | up |
| VIT_14s0060g00710.t01 | 627 | trafficking protein particle complex subunit 4-like | 790 | 85.083235 | 209 | 30.304639 | 1.4893362 | 2.30E-48 | 1.55E-49 | up |
| VIT_11s0016g01330.t01 | 1104 | Putative uncharacterized protein | 1099 | 67.222253 | 290 | 23.881373 | 1.4930531 | 3.88E-67 | 1.90E-68 | up |
| VIT_01s0010g03980.t01 | 1182 | Putative uncharacterized protein | 1031 | 58.901402 | 271 | 20.844054 | 1.4986661 | 2.18E-63 | 1.13E-64 | up |
| VIT_07s0191g00230.t01 | 1844 | RING-H2 finger protein ATL54 | 1427 | 52.257364 | 375 | 18.488459 | 1.4990093 | 2.07E-87 | 7.95E-89 | up |
| VIT_14s0068g01490.t01 | 1049 | NAC domain-containing protein 68-like | 3042 | 195.82501 | 798 | 69.160444 | 1.501546 | 7.63E-186 | 1.38E-187 | up |
| VIT_11s0016g04050.t01 | 1298 | Putative uncharacterized protein | 1339 | 69.6611 | 351 | 24.58458 | 1.5025995 | 2.30E-82 | 9.37E-84 | up |
| VIT_06s0009g02350.t01 | 1344 | E3 ubiquitin-protein ligase RING1-like | 2358 | 118.47562 | 618 | 41.804167 | 1.5028715 | 2.06E-144 | 4.72E-146 | up |
| VIT_18s0072g00840.t01 | 1744 | protein SENSITIVE TO PROTON RHIZOTOXICITY 1-like | 855 | 33.105799 | 224 | 11.677017 | 1.5034122 | 5.59E-53 | 3.45E-54 | up |
| VIT_18s0089g01230.t01 | 1376 | putative fructokinase-5-like | 1378 | 67.626238 | 361 | 23.851689 | 1.5034917 | 8.30E-85 | 3.26E-86 | up |
| VIT_05s0062g00820.t01 | 757 | dCTP pyrophosphatase 1-like | 2153 | 192.05808 | 564 | 67.735072 | 1.5035678 | 5.15E-132 | 1.28E-133 | up |
| VIT_13s0074g00230.t01 | 771 | Putative uncharacterized protein | 443 | 38.800185 | 116 | 13.678359 | 1.5041684 | 5.83E-28 | 6.30E-29 | up |
| VIT_14s0128g00520.t01 | 1373 | esterase-like | 382 | 18.787858 | 100 | 6.6215526 | 1.5045591 | 2.89E-24 | 3.52E-25 | up |

| | | | | | | | | | | |
|---|---|---|---|---|---|---|---|---|---|---|
| VIT_14s0030g00740.t01 | 764 | acyl carrier protein 1 | 5662 | 500.4503 | 1481 | 176.23496 | 1.5057266 | 0 | 0 | up |
| VIT_01s0011g04650.t01 | 509 | Putative uncharacterized protein | 2128 | 282.31782 | 556 | 99.308719 | 1.5073279 | 5.68E-131 | 1.43E-132 | up |
| VIT_18s0001g14400.t01 | 776 | peptidyl-prolyl cis-trans isomerase-like | 92913 | 8085.3571 | 24273 | 2843.7545 | 1.5075145 | 0 | 0 | up |
| VIT_05s0020g04250.t01 | 662 | thioredoxin-like protein 4A | 3014 | 307.44661 | 787 | 108.08044 | 1.5082304 | 2.38E-185 | 4.32E-187 | up |
| VIT_06s0004g08080.t01 | 1192 | RING-H2 finger protein ATL72-like | 511 | 28.948701 | 133 | 10.143919 | 1.5128836 | 2.31E-32 | 2.22E-33 | up |
| VIT_01s0011g06500.t01 | 1164 | galacturonic acid reductase | 588 | 34.112126 | 153 | 11.950025 | 1.513271 | 4.23E-37 | 3.64E-38 | up |
| VIT_19s0014g04510.t01 | 860 | G-type lectin S-receptor-like serine/threonine-protein | 77 | 6.0461194 | 20 | 2.1142772 | 1.515845 | 8.00E-06 | 2.87E-06 | up |
| VIT_13s0084g00810.t01 | 948 | U-box domain-containing protein 4-like | 135 | 9.6163414 | 35 | 3.3565265 | 1.5185191 | 2.12E-09 | 5.48E-10 | up |
| VIT_12s0057g00460.t01 | 1443 | cytochrome b561 and DOMON domain-containing protein | 189 | 8.844635 | 49 | 3.087167 | 1.5185191 | 1.04E-12 | 2.11E-13 | up |
| VIT_16s0039g01640.t01 | 453 | Putative uncharacterized protein | 166 | 24.745392 | 43 | 8.6297979 | 1.5197612 | 2.59E-11 | 5.80E-12 | up |
| VIT_00s0187g00360.t01 | 749 | blue copper protein-like | 31255 | 2817.8776 | 8096 | 982.6957 | 1.5197923 | 0 | 0 | up |
| VIT_02s0025g02660.t01 | 1576 | protein GPR107-like | 7240 | 310.21786 | 1875 | 108.16218 | 1.5200856 | 0 | 0 | up |
| VIT_00s0399g00060.t01 | 967 | Alternative oxidase | 143 | 9.9860562 | 37 | 3.4786091 | 1.5214045 | 6.48E-10 | 1.61E-10 | up |
| VIT_17s0000g03680.t01 | 1143 | Putative uncharacterized protein | 3123 | 184.50587 | 808 | 64.268106 | 1.5214919 | 1.66E-194 | 2.83E-196 | up |
| VIT_08s0007g05790.t01 | 951 | probable calcium-binding protein CML35-like | 9026 | 640.91326 | 2335 | 223.22187 | 1.5216507 | 0 | 0 | up |
| VIT_11s0149g00240.t01 | 237 | 60S ribosomal protein L39-3 | 317 | 90.322377 | 82 | 31.455448 | 1.5217735 | 1.21E-20 | 1.68E-21 | up |
| VIT_05s0077g01300.t01 | 1284 | 3-oxo-Delta(4,5)-steroid 5-beta-reductase-like | 847 | 44.545397 | 219 | 15.506346 | 1.5224176 | 1.87E-53 | 1.14E-54 | up |
| VIT_05s0094g01210.t01 | 1398 | amine oxidase | 298 | 14.394399 | 77 | 5.0074189 | 1.5233685 | 1.68E-19 | 2.46E-20 | up |
| VIT_15s0021g02470.t01 | 1072 | bifunctional epoxide hydrolase 2 | 202 | 12.724509 | 52 | 4.4100035 | 1.5287583 | 1.24E-13 | 2.39E-14 | up |
| VIT_18s0001g15150.t01 | 1316 | Putative uncharacterized protein | 474 | 24.322426 | 122 | 8.4281899 | 1.5289924 | 1.50E-30 | 1.51E-31 | up |
| VIT_06s0061g00710.t01 | 1164 | RING finger and CHY zinc finger domain-containing protein 1-like | 237 | 13.749275 | 61 | 4.7643892 | 1.5289924 | 8.25E-16 | 1.42E-16 | up |
| VIT_02s0241g00070.t01 | 648 | probable protein S-acyltransferase 14 | 70 | 7.2947007 | 18 | 2.5253866 | 1.5303445 | 1.87E-05 | 7.01E-06 | up |
| VIT_15s0048g02070.t01 | 771 | BON1-associated protein 2 | 638 | 55.879273 | 164 | 19.338369 | 1.5308471 | 7.75E-41 | 6.10E-42 | up |
| VIT_18s0001g14410.t01 | 2370 | Putative uncharacterized protein | 962 | 27.410135 | 247 | 9.4749948 | 1.5325124 | 3.68E-61 | 1.97E-62 | up |
| VIT_18s0001g07450.t01 | 1315 | Putative uncharacterized protein | 261 | 13.402913 | 67 | 4.632116 | 1.5328033 | 2.32E-17 | 3.72E-18 | up |
| VIT_08s0040g03150.t01 | 1071 | ascorbate peroxidase | 1319 | 83.164842 | 338 | 28.691787 | 1.5353359 | 9.80E-84 | 3.91E-85 | up |
| VIT_12s0059g01580.t01 | 839 | Putative uncharacterized protein | 125 | 10.0608 | 32 | 3.4675153 | 1.5367708 | 6.39E-09 | 1.71E-09 | up |
| VIT_19s0014g01200.t01 | 478 | Putative uncharacterized protein | 727 | 102.70485 | 186 | 35.376545 | 1.5376393 | 1.09E-46 | 7.60E-48 | up |
| VIT_06s0009g01910.t01 | 852 | calmodulin-related protein-like | 23363 | 1851.7121 | 5977 | 637.78461 | 1.5377186 | 0 | 0 | up |
| VIT_16s0013g00610.t01 | 786 | protein mago nashi homolog-like | 1955 | 167.96108 | 500 | 57.833281 | 1.5381551 | 5.15E-124 | 1.39E-125 | up |
| VIT_01s0026g02740.t01 | 981 | Putative uncharacterized protein | 1674 | 115.23141 | 427 | 39.572113 | 1.5419781 | 1.07E-106 | 3.35E-108 | up |
| VIT_01s0010g02770.t01 | 1282 | adenylate kinase B-like | 157 | 8.2698203 | 40 | 2.8366277 | 1.5436792 | 5.53E-11 | 1.27E-11 | up |
| VIT_14s0060g01010.t01 | 894 | transcription factor bHLH149-like | 577 | 43.583564 | 147 | 14.948933 | 1.5437417 | 1.78E-37 | 1.51E-38 | up |
| VIT_05s0077g00130.t01 | 433 | Putative uncharacterized protein | 1115 | 173.88872 | 284 | 59.629452 | 1.5440674 | 1.62E-71 | 7.60E-73 | up |
| VIT_13s0019g02940.t01 | 1680 | UDP-glycosyltransferase 85A1-like | 499 | 20.057449 | 127 | 6.8726592 | 1.5451978 | 1.36E-32 | 1.30E-33 | up |
| VIT_00s0187g00040.t01 | 513 | cysteine proteinase inhibitor 1-like | 409 | 53.838182 | 104 | 18.430892 | 1.5465038 | 6.23E-27 | 6.96E-28 | up |
| VIT_04s0008g05180.t01 | 899 | Putative uncharacterized protein | 826 | 62.044716 | 210 | 21.236844 | 1.546739 | 2.33E-53 | 1.43E-54 | up |
| VIT_00s0358g00010.t01 | 266 | Putative uncharacterized protein | 63 | 15.993494 | 16 | 5.4685063 | 1.5482664 | 4.37E-05 | 1.71E-05 | up |
| VIT_01s0010g02980.t01 | 825 | probable calcium-binding protein CML31 | 67 | 5.4840991 | 17 | 1.8733777 | 1.5496129 | 2.41E-05 | 9.14E-06 | up |
| VIT_07s0191g00130.t01 | 805 | histone H3.3 | 27603 | 2315.5003 | 7003 | 790.89461 | 1.5497666 | 0 | 0 | up |
| VIT_00s0353g00060.t01 | 2578 | receptor-like serine/threonine-protein kinase SD1-8-like | 339 | 8.87976 | 86 | 3.0328149 | 1.5498632 | 1.42E-22 | 1.85E-23 | up |
| VIT_09s0002g08170.t01 | 456 | Putative uncharacterized protein | 71 | 10.514242 | 18 | 3.5887073 | 1.5508086 | 1.33E-05 | 4.88E-06 | up |
| VIT_03s0017g00420.t01 | 617 | magnesium-dependent phosphatase 1, putative, expressed | 75 | 8.2084384 | 19 | 2.7996182 | 1.5518777 | 7.31E-06 | 2.61E-06 | up |
| VIT_16s0100g01280.t01 | 912 | membrane steroid-binding protein 2-like | 4666 | 345.48909 | 1179 | 117.53016 | 1.5556091 | 7.51E-300 | 8.33E-302 | up |
| VIT_08s0007g02520.t01 | 877 | Putative uncharacterized protein | 190 | 14.629802 | 48 | 4.9759043 | 1.5558796 | 3.41E-13 | 6.71E-14 | up |
| VIT_18s0001g07150.t01 | 1930 | serine/threonine-protein kinase-like protein | 761 | 26.626359 | 192 | 9.0442861 | 1.5577767 | 9.53E-50 | 6.30E-51 | up |
| VIT_15s0048g00530.t01 | 965 | Putative uncharacterized protein | 1453 | 101.677 | 366 | 34.481341 | 1.5601057 | 2.60E-94 | 9.22E-96 | up |
| VIT_00s0179g00370.t01 | 1261 | tumor susceptibility gene 101, putative, expressed | 294 | 15.744058 | 74 | 5.3351546 | 1.5612055 | 6.41E-20 | 9.23E-21 | up |
| VIT_14s0006g02530.t01 | 602 | non-specific lipid-transfer protein | 3752 | 420.87272 | 944 | 142.56269 | 1.5617876 | 1.28E-242 | 1.69E-244 | up |
| VIT_04s0008g01980.t01 | 701 | OsMADS68 - MADS-box family gene with MIKC* type-box, expressed | 167 | 16.08729 | 42 | 5.4470536 | 1.5623734 | 8.48E-12 | 1.83E-12 | up |
| VIT_04s0008g02620.t01 | 700 | LSM domain containing protein, expressed | 191 | 18.425521 | 48 | 6.2340972 | 1.5634528 | 2.41E-13 | 4.71E-14 | up |
| VIT_09s0054g00930.t01 | 1574 | delta14-sterol reductase, putative, expressed | 4519 | 193.87511 | 1135 | 65.557368 | 1.5642978 | 8.50E-293 | 9.86E-295 | up |
| VIT_06s0080g01090.t01 | 979 | CAF1 family ribonuclease containing protein, putative, expressed | 10831 | 747.0855 | 2720 | 252.59025 | 1.5644744 | 0 | 0 | up |
| VIT_14s0006g03230.t01 | 986 | 14-3-3 protein, putative, expressed | 5390 | 369.14441 | 1352 | 124.66087 | 1.5661766 | 0 | 0 | up |
| VIT_04s0008g01260.t01 | 1492 | zinc transporter 4 | 1548 | 70.062653 | 387 | 23.581559 | 1.5709865 | 1.95E-101 | 6.48E-103 | up |
| VIT_08s0007g04760.t01 | 783 | ankyrin repeat domain containing protein, expressed | 380 | 32.772251 | 95 | 11.030424 | 1.5709865 | 1.18E-25 | 1.37E-26 | up |
| VIT_09s0002g05780.t01 | 431 | Core histone H2A/H2B/H3/H4 domain containing protein, putative, expressed | 300 | 47.003308 | 75 | 15.820287 | 1.5709865 | 1.77E-20 | 2.48E-21 | up |
| VIT_02s0025g02720.t01 | 788 | Putative uncharacterized protein | 204 | 17.48189 | 51 | 5.8840226 | 1.5709865 | 2.84E-14 | 5.31E-15 | up |
| VIT_05s0077g00240.t01 | 619 | Putative uncharacterized protein | 68 | 7.4182712 | 17 | 2.4968281 | 1.5709865 | 1.70E-05 | 6.35E-06 | up |
| VIT_18s0001g13720.t01 | 169 | GAGA-binding transcriptional activator BBR/BPC6-like | 56 | 22.376171 | 14 | 7.5313305 | 1.5709865 | 0.00010196 | 4.19E-05 | up |
| VIT_09s0002g07540.t01 | 581 | VQ domain containing protein, putative, expressed | 52 | 6.0438219 | 13 | 2.0342185 | 1.5709865 | 0.00018539 | 7.89E-05 | up |
| VIT_06s0009g03190.t01 | 1703 | Putative uncharacterized protein | 337 | 13.362869 | 84 | 4.4843036 | 1.5752739 | 5.84E-23 | 7.48E-24 | up |
| VIT_00s2837g00010.t01 | 666 | histone H3, putative, expressed | 26611 | 2698.183 | 6630 | 905.04395 | 1.5759285 | 0 | 0 | up |
| VIT_03s0063g02660.t01 | 1340 | hhH-GPD superfamily base excision DNA repair protein, putative, expressed | 446 | 22.475766 | 111 | 7.530929 | 1.5774705 | 4.19E-30 | 4.29E-31 | up |
| VIT_18s0001g09460.t01 | 662 | GASR3 - Gibberellin-regulated GASA/GAST/Snakin family protein precursor, expr | 145 | 14.790895 | 36 | 4.9439593 | 1.5809706 | 1.49E-10 | 3.52E-11 | up |
| VIT_18s0001g02260.t01 | 975 | peptidase, T1 family, putative, expressed | 5759 | 398.8659 | 1429 | 133.24717 | 1.5817989 | 0 | 0 | up |
| VIT_18s0001g00130.t01 | 1571 | expressed protein | 343 | 14.743561 | 85 | 4.918958 | 1.5836603 | 1.61E-23 | 2.01E-24 | up |
| VIT_01s0010g02200.t01 | 1059 | zinc finger, RING-type, putative, expressed | 109 | 6.9504829 | 27 | 2.3179186 | 1.5842833 | 3.16E-08 | 8.88E-09 | up |
| VIT_09s0002g03450.t01 | 1166 | clavaminate synthase-like protein | 7298 | 422.65864 | 1807 | 140.89318 | 1.5848912 | 0 | 0 | up |
| VIT_08s0007g04790.t01 | 1799 | RING finger and CHY zinc finger domain-containing protein 1, putative, expressed | 505 | 18.955911 | 125 | 6.3169759 | 1.5853418 | 3.45E-34 | 3.17E-35 | up |
| VIT_03s0038g03800.t01 | 1399 | serine/threonine-protein kinase Cx32, chloroplast precursor, putative, expressed | 982 | 47.399986 | 243 | 15.791338 | 1.5857532 | 1.48E-65 | 7.47E-67 | up |
| VIT_16s0098g01150.t01 | 789 | indole-3-acetic acid-induced protein ARG7-like | 688 | 58.883807 | 170 | 19.58855 | 1.5878603 | 2.58E-46 | 1.82E-47 | up |
| VIT_00s0404g00030.t01 | 141 | transmembrane protein 184 | 73 | 34.96135 | 18 | 11.606032 | 1.5908861 | 6.61E-06 | 2.35E-06 | up |
| VIT_08s0056g00290.t01 | 878 | OsCML32 - Calmodulin-related calcium sensor protein, expressed | 1096 | 84.294741 | 270 | 27.957583 | 1.592203 | 1.75E-73 | 7.95E-75 | up |
| VIT_16s0022g00080.t01 | 2118 | leucine-rich repeat receptor protein kinase EXS precursor, putative, expressed | 480 | 15.303816 | 118 | 5.0650813 | 1.5952341 | 7.81E-33 | 7.45E-34 | up |
| VIT_12s0059g01850.t01 | 1432 | peroxisomal membrane protein, putative, expressed | 342 | 16.127518 | 84 | 5.3329393 | 1.5965216 | 1.02E-23 | 1.26E-24 | up |
| VIT_16s0039g01310.t01 | 2044 | Protein kinase domain containing protein, expressed | 224 | 7.4003382 | 55 | 2.4463138 | 1.5969817 | 6.48E-16 | 1.11E-16 | up |
| VIT_13s0067g03890.t01 | 1654 | 3-ketoacyl-CoA synthase precursor, putative, expressed | 3238 | 132.19827 | 795 | 43.698044 | 1.5970627 | 2.35E-216 | 3.46E-218 | up |
| VIT_07s0005g03290.t01 | 1771 | inorganic phosphate transporter, putative, expressed | 330 | 12.582873 | 81 | 4.1581182 | 1.5974587 | 6.05E-23 | 7.76E-24 | up |
| VIT_11s0149g00280.t01 | 1206 | acidic mammalian chitinase-like | 542 | 30.348443 | 133 | 10.026162 | 1.5978531 | 4.99E-37 | 4.30E-38 | up |
| VIT_03s0063g00780.t01 | 1107 | 2-hydroxyisoflavanone dehydratase | 216 | 13.176212 | 53 | 4.3526989 | 1.5979536 | 2.13E-15 | 3.76E-16 | up |
| VIT_06s0004g05850.t01 | 958 | zinc finger, C3HC4 type domain containing protein, expressed | 106 | 7.4717924 | 26 | 2.4673923 | 1.5984672 | 4.02E-08 | 1.14E-08 | up |
| VIT_09s0002g02990.t01 | 2405 | senescence-induced receptor-like serine/threonine-protein kinase precursor, putative, | 371 | 10.417015 | 91 | 3.4399861 | 1.5984672 | 1.10E-25 | 1.28E-26 | up |
| VIT_06s0009g00850.t01 | 1396 | strictosidine synthase | 2161 | 104.53309 | 529 | 34.450904 | 1.601346 | 3.28E-145 | 7.45E-147 | up |
| VIT_04s0008g03100.t01 | 1307 | Putative uncharacterized protein | 2894 | 149.52279 | 708 | 49.247937 | 1.6022302 | 2.30E-194 | 3.94E-196 | up |
| VIT_18s0001g14260.t01 | 199 | snakin-2 | 139 | 47.167859 | 34 | 15.533031 | 1.6024647 | 2.43E-10 | 5.83E-11 | up |
| VIT_14s0060g01470.t01 | 2387 | calmodulin binding protein, putative, expressed | 688 | 19.463479 | 168 | 6.3986335 | 1.6049338 | 5.08E-47 | 3.53E-48 | up |
| VIT_08s0007g01420.t01 | 986 | glutathione S-transferase | 86 | 5.8898737 | 21 | 1.9363005 | 1.6049338 | 7.83E-07 | 2.52E-07 | up |
| VIT_01s0011g03070.t01 | 1471 | B3 DNA binding domain containing protein, expressed | 2757 | 126.56352 | 672 | 41.532395 | 1.6075526 | 4.37E-186 | 7.86E-188 | up |
| VIT_19s0090g01330.t01 | 563 | expressed protein | 944 | 113.22649 | 230 | 37.140677 | 1.6081395 | 2.51E-64 | 1.29E-65 | up |
| VIT_05s0077g02210.t01 | 1340 | Ser/Thr protein phosphatase family protein, putative, expressed | 624 | 31.445915 | 152 | 10.312623 | 1.6084612 | 7.12E-43 | 5.39E-44 | up |
| VIT_03s0038g01930.t01 | 707 | peptidyl-prolyl cis-trans isomerase, putative, expressed | 16884 | 1612.6509 | 4111 | 528.63807 | 1.609082 | 0 | 0 | up |
| VIT_02s0012g02190.t01 | 4029 | CSLD2 - cellulose synthase-like family D, expressed | 1134 | 19.006416 | 276 | 6.227908 | 1.609667 | 3.47E-77 | 1.48E-78 | up |
| VIT_05s0077g00730.t01 | 1205 | WRKY transcription factor 48 | 226 | 12.665019 | 55 | 4.1495979 | 1.6098058 | 3.22E-16 | 5.42E-17 | up |
| VIT_14s0066g01320.t01 | 1516 | erythronate-4-phosphate dehydrogenase domain containing protein, expressed | 6830 | 304.23274 | 1662 | 99.66948 | 1.6099517 | 0 | 0 | up |
| VIT_01s0026g02690.t01 | 302 | YGL010w, putative, expressed | 74 | 16.546617 | 18 | 5.4187103 | 1.6105149 | 4.65E-06 | 1.63E-06 | up |
| VIT_17s0000g08010.t01 | 2451 | trehalose-6-phosphate synthase, putative, expressed | 251 | 6.915361 | 61 | 2.2626475 | 1.6117927 | 6.32E-18 | 9.91E-19 | up |

| | | | | | | | | | | |
|---|---|---|---|---|---|---|---|---|---|---|
| VIT_05s0020g04800.t01 | 879 | histone H1-like | 7221 | 554.74438 | 1749 | 180.89698 | 1.6166549 | 0 | 0 | up |
| VIT_01s0011g00380.t01 | 1905 | esterase, putative, expressed | 157 | 5.5653069 | 38 | 1.813506 | 1.6176797 | 1.12E-11 | 2.44E-12 | up |
| VIT_08s0007g08170.t01 | 587 | Putative uncharacterized protein | 861 | 99.048863 | 208 | 32.214812 | 1.6204162 | 2.16E-59 | 1.19E-60 | up |
| VIT_18s0001g01460.t01 | 454 | Putative uncharacterized protein | 232 | 34.507745 | 56 | 11.214052 | 1.6216126 | 8.72E-17 | 1.43E-17 | up |
| VIT_13s0019g02340.t01 | 846 | membrane associated DUF588 domain containing protein, putative, expressed | 228 | 18.199059 | 55 | 5.9104793 | 1.6225168 | 1.58E-16 | 2.63E-17 | up |
| VIT_08s0040g01680.t01 | 1622 | Putative uncharacterized protein | 1858 | 77.353381 | 448 | 25.110626 | 1.6231664 | 2.06E-127 | 5.37E-129 | up |
| VIT_17s0000g06360.t01 | 771 | expansin precursor, putative, expressed | 278 | 24.348649 | 67 | 7.9004312 | 1.6238384 | 6.14E-20 | 8.83E-21 | up |
| VIT_13s0067g03260.t01 | 1041 | esterase, putative, expressed | 245 | 15.892777 | 59 | 5.152662 | 1.6249814 | 1.02E-17 | 1.62E-18 | up |
| VIT_16s0050g01680.t01 | 1603 | UDP-glucoronosyl and UDP-glucosyl transferase domain containing protein, express | 1456 | 61.335555 | 350 | 19.8502 | 1.62757 | 2.00E-100 | 6.73E-102 | up |
| VIT_05s0077g01860.t01 | 1089 | ethylene-responsive transcription factor RAP2-3 | 433 | 26.85001 | 104 | 8.6823209 | 1.62877 | 1.36E-30 | 1.38E-31 | up |
| VIT_02s0087g01010.t01 | 1341 | TKL_IRAK_DUF26-lc.12 - DUF26 kinases have homology to DUF26 containing lc | 225 | 11.330216 | 54 | 3.6609631 | 1.6298802 | 2.01E-16 | 3.36E-17 | up |
| VIT_14s0060g02130.t01 | 235 | Putative uncharacterized protein | 75 | 21.551517 | 18 | 6.9636192 | 1.6298802 | 3.27E-06 | 1.12E-06 | up |
| VIT_05s0077g00280.t01 | 1970 | beta-amylase, putative, expressed | 2440 | 83.638848 | 585 | 26.99728 | 1.6313591 | 2.85E-168 | 5.53E-170 | up |
| VIT_02s0025g04660.t01 | 1042 | senescence-inducible chloroplast stay-green protein 1, putative, expressed | 559 | 36.22668 | 134 | 11.691425 | 1.6316018 | 2.71E-39 | 2.22E-40 | up |
| VIT_02s0025g02370.t01 | 552 | yippee zinc-binding protein, putative, expressed | 330 | 40.370052 | 79 | 13.011231 | 1.633528 | 1.17E-23 | 1.46E-24 | up |
| VIT_16s0013g00970.t01 | 951 | ethylene-responsive transcription factor ERF105 | 2808 | 199.38892 | 672 | 64.242011 | 1.6339963 | 5.38E-194 | 9.27E-196 | up |
| VIT_17s0000g09710.t01 | 2179 | protein kinase domain containing protein, expressed | 2098 | 65.017864 | 502 | 20.944831 | 1.6342419 | 3.64E-145 | 8.30E-147 | up |
| VIT_12s0028g01000.t01 | 869 | transmembrane protein 56, putative, expressed | 92 | 7.1491185 | 22 | 2.3016182 | 1.6351168 | 2.13E-07 | 6.48E-08 | up |
| VIT_06s0004g06150.t01 | 1209 | formin-like protein 14 | 2239 | 125.05822 | 535 | 40.230725 | 1.6362302 | 3.88E-155 | 8.29E-157 | up |
| VIT_08s0007g07290.t01 | 922 | eukaryotic translation initiation factor, putative, expressed | 2254 | 165.08493 | 538 | 53.049553 | 1.6377959 | 2.19E-156 | 4.65E-158 | up |
| VIT_01s0011g01290.t01 | 1688 | hydrolase, putative, expressed | 671 | 26.843214 | 160 | 8.6174329 | 1.6392274 | 2.87E-47 | 1.98E-48 | up |
| VIT_00s0532g00030.t01 | 533 | thioredoxin, putative, expressed | 709 | 89.826291 | 169 | 28.826364 | 1.6397489 | 6.53E-50 | 4.30E-51 | up |
| VIT_07s0005g02200.t01 | 594 | autophagy-related protein 8D, putative, expressed | 235 | 26.715657 | 56 | 8.5710091 | 1.6401485 | 2.98E-17 | 4.82E-18 | up |
| VIT_16s0098g01690.t01 | 1660 | Putative uncharacterized protein | 126 | 5.1256258 | 30 | 1.6430226 | 1.6413758 | 8.99E-10 | 2.26E-10 | up |
| VIT_15s0046g02420.t01 | 1058 | aquaporin protein, putative, expressed | 14879 | 949.66956 | 3541 | 304.27806 | 1.6420353 | 0 | 0 | up |
| VIT_06s0004g00990.t01 | 810 | dirigent protein 22 | 1069 | 89.120401 | 254 | 28.508809 | 1.644348 | 4.74E-75 | 2.09E-76 | up |
| VIT_02s0236g00020.t01 | 618 | Putative uncharacterized protein | 219 | 23.929856 | 52 | 7.6497148 | 1.6453339 | 3.23E-16 | 5.45E-17 | up |
| VIT_18s0001g07640.t01 | 1426 | transferase family protein, putative, expressed | 855 | 40.488439 | 203 | 12.942164 | 1.6454312 | 2.84E-60 | 1.54E-61 | up |
| VIT_17s0000g02930.t01 | 886 | glutathione S-transferase | 451 | 34.373778 | 107 | 10.979446 | 1.6465031 | 2.66E-32 | 2.56E-33 | up |
| VIT_14s0060g02140.t01 | 1003 | stress-related protein, putative, expressed | 4545 | 305.99716 | 1078 | 97.712067 | 1.6469096 | 0 | 0 | up |
| VIT_05s0077g00030.t01 | 1610 | ammonium transporter protein, putative, expressed | 1881 | 78.894615 | 446 | 25.184849 | 1.6473707 | 1.01E-131 | 2.52E-133 | up |
| VIT_00s1522g00010.t01 | 408 | protein SKIP34 | 135 | 22.343852 | 32 | 7.1305033 | 1.6478021 | 1.93E-10 | 4.58E-11 | up |
| VIT_04s0008g03220.t01 | 1437 | hydrolase, alpha/beta fold family domain containing protein, expressed | 1107 | 52.020593 | 262 | 16.575815 | 1.650003 | 4.62E-78 | 1.95E-79 | up |
| VIT_12s0035g02200.t01 | 510 | SNARE associated Golgi protein, putative, expressed | 110 | 14.564881 | 26 | 4.6348272 | 1.6519065 | 9.69E-09 | 2.62E-09 | up |
| VIT_16s0039g02030.t01 | 75 | Putative uncharacterized protein | 72 | 64.826963 | 17 | 20.607155 | 1.6534487 | 4.13E-06 | 1.44E-06 | up |
| VIT_16s0050g01190.t01 | 1015 | FK506-binding protein 4 | 178 | 11.842364 | 42 | 3.7619552 | 1.6544025 | 1.76E-13 | 3.41E-14 | up |
| VIT_03s0063g00820.t01 | 1170 | probable carboxylesterase 12 | 1509 | 87.093917 | 356 | 27.662696 | 1.6546302 | 1.66E-106 | 5.20E-108 | up |
| VIT_08s0040g02820.t01 | 748 | Putative uncharacterized protein | 543 | 49.021057 | 128 | 15.557462 | 1.6557949 | 5.48E-39 | 4.51E-40 | up |
| VIT_06s0004g00980.t01 | 778 | dirigent protein 19 | 798 | 69.264027 | 188 | 21.968916 | 1.6566426 | 7.34E-57 | 4.24E-58 | up |
| VIT_16s0098g00720.t01 | 2426 | L-type lectin-domain containing receptor kinase VIII.2-like | 698 | 19.428938 | 164 | 6.1458708 | 1.6605177 | 5.03E-50 | 3.29E-51 | up |
| VIT_06s0004g01820.t01 | 996 | zinc finger A20 and AN1 domain-containing stress-associated protein, putative, expr | 3227 | 218.78829 | 758 | 69.189508 | 1.6609103 | 4.60E-228 | 6.45E-230 | up |
| VIT_14s0060g00690.t01 | 1329 | ADP-ribosylation factor, putative, expressed | 32974 | 1675.4485 | 7726 | 528.51838 | 1.6645218 | 0 | 0 | up |
| VIT_07s0005g05790.t01 | 869 | vesicle-associated membrane protein, putative, expressed | 2331 | 181.1369 | 546 | 57.121978 | 1.6649627 | 1.61E-165 | 3.19E-167 | up |
| VIT_08s0056g01240.t01 | 904 | Putative uncharacterized protein | 154 | 11.503678 | 36 | 3.6204657 | 1.667848 | 6.15E-12 | 1.31E-12 | up |
| VIT_05s0020g03340.t01 | 927 | Putative uncharacterized protein | 77 | 5.6091291 | 18 | 1.7653188 | 1.667848 | 1.59E-06 | 5.29E-07 | up |
| VIT_00s0264g00020.t01 | 1490 | zinc finger, C3HC4 type, domain containing protein, expressed | 1379 | 62.497471 | 322 | 19.647169 | 1.6694764 | 1.20E-98 | 4.10E-100 | up |
| VIT_02s0025g00740.t01 | 775 | disease resistance response protein 206 | 189 | 16.46814 | 44 | 5.1615644 | 1.6737973 | 1.81E-14 | 3.34E-15 | up |
| VIT_04s0008g02000.t01 | 1363 | Putative uncharacterized protein | 1281 | 63.465502 | 298 | 19.876997 | 1.6748727 | 4.10E-92 | 1.49E-93 | up |
| VIT_00s0194g00060.t01 | 515 | Putative uncharacterized protein | 198 | 25.962255 | 46 | 8.1204664 | 1.6767812 | 3.82E-15 | 6.86E-16 | up |
| VIT_17s0000g08000.t01 | 810 | NFD2, putative, expressed | 991 | 82.617696 | 230 | 25.815063 | 1.6782377 | 1.12E-71 | 5.27E-73 | up |
| VIT_05s0020g02720.t01 | 1140 | basic 7S globulin precursor, putative, expressed | 505 | 29.913758 | 117 | 9.3306389 | 1.6807614 | 4.32E-37 | 3.72E-38 | up |
| VIT_08s0007g05580.t01 | 1019 | methyltransferase, putative, expressed | 190 | 12.591105 | 44 | 3.9256255 | 1.6814105 | 1.26E-14 | 2.31E-15 | up |
| VIT_05s0020g04120.t01 | 1306 | AGAP010609-PA, putative, expressed | 134 | 6.9286092 | 31 | 2.1579873 | 1.6828794 | 1.18E-10 | 2.77E-11 | up |
| VIT_18s0122g00290.t01 | 945 | protein MIZU-KUSSEI 1 | 424 | 30.298316 | 98 | 9.42811 | 1.6841971 | 2.16E-31 | 2.13E-32 | up |
| VIT_02s0025g03070.t01 | 851 | RING-H2 finger protein, putative, expressed | 312 | 24.757653 | 72 | 7.6918943 | 1.6864637 | 2.03E-23 | 2.55E-24 | up |
| VIT_17s0000g03560.t01 | 1174 | MYB family transcription factor, putative, expressed | 330 | 18.981489 | 76 | 5.8853984 | 1.6893812 | 9.13E-25 | 1.09E-25 | up |
| VIT_08s0007g04960.t01 | 938 | pentatricopeptide repeat-containing protein | 122 | 8.7829707 | 28 | 2.7138483 | 1.6943689 | 6.94E-10 | 1.73E-10 | up |
| VIT_02s0025g04440.t01 | 826 | ethylene-responsive transcription factor ERF026 | 96 | 7.8483006 | 22 | 2.4214361 | 1.6965174 | 5.04E-08 | 1.44E-08 | up |
| VIT_00s2527g00010.t01 | 808 | glycosyl hydrolases, putative, expressed | 105 | 8.7753083 | 24 | 2.7004134 | 1.7002695 | 1.07E-08 | 2.90E-09 | up |
| VIT_18s0001g06790.t01 | 1650 | MATE efflux family protein | 289 | 11.827647 | 66 | 3.6365567 | 1.7015181 | 4.93E-22 | 6.53E-23 | up |
| VIT_19s0015g00170.t01 | 1352 | zinc ion binding protein, putative, expressed | 127 | 6.3432448 | 29 | 1.9500766 | 1.7016902 | 2.68E-10 | 6.43E-11 | up |
| VIT_06s0004g00730.t01 | 800 | ubiquitin-conjugating enzyme, putative, expressed | 237 | 20.005196 | 54 | 6.1366894 | 1.7048423 | 2.60E-18 | 4.01E-19 | up |
| VIT_16s0013g01070.t01 | 1077 | ethylene-responsive transcription factor ERF105 | 1418 | 88.908845 | 323 | 27.265734 | 1.705238 | 1.82E-104 | 5.79E-106 | up |
| VIT_17s0000g03780.t01 | 560 | Putative uncharacterized protein | 1805 | 217.65749 | 411 | 66.724322 | 1.705775 | 8.50E-133 | 2.10E-134 | up |
| VIT_14s0068g01250.t01 | 484 | phosphate carrier protein, mitochondrial precursor, putative, expressed | 110 | 15.347292 | 25 | 4.6959668 | 1.70849 | 4.10E-09 | 1.08E-09 | up |
| VIT_02s0236g00060.t01 | 616 | Putative uncharacterized protein | 264 | 28.940608 | 60 | 8.8552517 | 1.70849 | 2.48E-20 | 3.51E-21 | up |
| VIT_09s0002g04280.t01 | 888 | dynein light chain type 1 domain containing protein, expressed | 814 | 61.900746 | 185 | 18.9404 | 1.70849 | 1.59E-60 | 8.64E-62 | up |
| VIT_12s0059g02290.t01 | 715 | transmembrane protein 97 | 793 | 74.894787 | 180 | 22.88742 | 1.7103105 | 4.47E-59 | 2.49E-60 | up |
| VIT_09s0002g03760.t01 | 653 | Putative uncharacterized protein | 141 | 14.581103 | 32 | 4.4551996 | 1.7105379 | 2.19E-11 | 4.87E-12 | up |
| VIT_19s0015g00130.t01 | 1342 | serine acetyltransferase protein, putative, expressed | 2336 | 117.54516 | 530 | 35.9049 | 1.7109625 | 2.31E-172 | 4.38E-174 | up |
| VIT_19s0015g00020.t01 | 3287 | ABC transporter C family member 3-like | 551 | 11.319737 | 125 | 3.4573288 | 1.7111107 | 2.21E-41 | 1.72E-42 | up |
| VIT_02s0025g03290.t01 | 2098 | hydroxyproline | 300 | 9.6560657 | 68 | 2.9466856 | 1.7123424 | 5.00E-23 | 6.40E-24 | up |
| VIT_01s0011g04550.t01 | 768 | Putative uncharacterized protein | 2260 | 198.71546 | 510 | 60.372523 | 1.7187401 | 7.68E-168 | 1.49E-169 | up |
| VIT_00s0181g00010.t01 | 2017 | transporter family protein, putative, expressed | 3244 | 108.60739 | 732 | 32.994044 | 1.7188448 | 1.28E-240 | 1.70E-242 | up |
| VIT_10s0003g01710.t01 | 745 | ubiquitin domain-containing protein 2, putative, expressed | 120 | 10.877007 | 27 | 3.2948668 | 1.7229896 | 6.02E-10 | 1.49E-10 | up |
| VIT_08s0040g01720.t01 | 767 | Putative uncharacterized protein | 138 | 12.149773 | 31 | 3.6744869 | 1.7253147 | 2.74E-11 | 6.12E-12 | up |
| VIT_16s0100g00200.t01 | 2011 | Putative uncharacterized protein | 196 | 6.5815539 | 44 | 1.9891658 | 1.7262647 | 1.39E-15 | 2.43E-16 | up |
| VIT_15s0046g01880.t01 | 671 | zinc finger, C3HC4 type domain containing protein, expressed | 196 | 19.725045 | 44 | 5.9615684 | 1.7262647 | 1.39E-15 | 2.43E-16 | up |
| VIT_06s0009g01620.t01 | 950 | NHL25, putative, expressed | 2949 | 209.6214 | 662 | 63.352646 | 1.7263092 | 3.95E-220 | 5.76E-222 | up |
| VIT_18s0001g05310.t01 | 1147 | OsFBX168 - F-box domain containing protein, expressed | 352 | 20.723528 | 79 | 6.2617258 | 1.7266374 | 3.90E-27 | 4.33E-28 | up |
| VIT_02s0025g01320.t01 | 827 | Putative uncharacterized protein | 17349 | 1416.62 | 3891 | 427.74613 | 1.7276261 | 0 | 0 | up |
| VIT_09s0002g07980.t01 | 474 | la domain containing protein, expressed | 58 | 8.2629304 | 13 | 2.4934197 | 1.7285278 | 2.13E-05 | 8.05E-06 | up |
| VIT_05s0020g02060.t01 | 384 | Putative uncharacterized protein | 76 | 13.364934 | 17 | 4.0248349 | 1.7314512 | 9.61E-07 | 3.12E-07 | up |
| VIT_13s0019g02930.t01 | 756 | 18.1 kDa class I heat shock protein | 322 | 28.761963 | 72 | 8.6584683 | 1.7319784 | 5.13E-25 | 6.10E-26 | up |
| VIT_01s0011g05390.t01 | 891 | invertase/pectin methylesterase inhibitor family protein, putative, expressed | 246 | 18.644118 | 55 | 5.6119702 | 1.7321413 | 2.28E-19 | 3.36E-20 | up |
| VIT_14s0066g00600.t01 | 758 | Putative uncharacterized protein | 255 | 22.717232 | 57 | 6.8365347 | 1.7324499 | 4.83E-20 | 6.92E-21 | up |
| VIT_00s1194g00020.t01 | 505 | Putative uncharacterized protein | 121 | 16.179997 | 27 | 4.8607441 | 1.7349622 | 4.17E-10 | 1.02E-10 | up |
| VIT_14s0068g00830.t01 | 936 | Putative uncharacterized protein | 744 | 53.676171 | 166 | 16.123622 | 1.7351059 | 1.34E-56 | 7.79E-58 | up |
| VIT_09s0054g01220.t01 | 2283 | cycloartenol synthase, putative, expressed | 184 | 5.4424739 | 41 | 1.6327072 | 1.7369965 | 8.14E-15 | 1.49E-15 | up |
| VIT_03s0091g01080.t01 | 1215 | leucoanthocyanidin dioxygenase, putative, expressed | 377 | 20.953159 | 84 | 6.2854066 | 1.7370898 | 3.17E-29 | 3.31E-30 | up |
| VIT_16s0039g01060.t01 | 1693 | cytochrome P450, putative, expressed | 288 | 11.487353 | 64 | 3.4367931 | 1.7409115 | 1.21E-22 | 1.57E-23 | up |
| VIT_07s0104g00220.t01 | 1141 | phenazine biosynthesis protein, putative, expressed | 181 | 10.712168 | 40 | 3.1871663 | 1.7489043 | 1.01E-14 | 1.85E-15 | up |
| VIT_00s0199g00310.t01 | 912 | classical arabinogalactan protein 2-like | 1127 | 83.447537 | 249 | 24.821892 | 1.7492564 | 3.29E-86 | 1.28E-87 | up |
| VIT_09s0002g06300.t01 | 1320 | F-box protein | 403 | 20.616529 | 89 | 6.129802 | 1.7498891 | 1.76E-31 | 1.73E-32 | up |
| VIT_07s0005g03310.t01 | 763 | actin-depolymerizing factor, putative, expressed | 1336 | 118.24053 | 295 | 35.150204 | 1.7501197 | 4.23E-102 | 1.40E-103 | up |
| VIT_13s0067g03370.t01 | 601 | immediate early response 3-interacting protein 1 | 154 | 17.30337 | 34 | 5.1432166 | 1.7503102 | 1.07E-12 | 2.17E-13 | up |
| VIT_16s0013g00070.t01 | 562 | non-specific lipid-transfer protein | 218 | 26.194169 | 48 | 7.7648898 | 1.7542083 | 1.42E-17 | 2.25E-18 | up |

| | | | | | | | | | | |
|---|---|---|---|---|---|---|---|---|---|---|
| VIT_00s2086g00010.t01 | 920 | 1-aminocyclopropane-1-carboxylate oxidase | 10801 | 792.79441 | 2378 | 234.99271 | 1.7543308 | 0 | 0 | up |
| VIT_06s0004g08110.t01 | 614 | Putative uncharacterized protein | 50 | 5.4990298 | 11 | 1.628751 | 1.7554111 | 6.96E-05 | 2.79E-05 | up |
| VIT_18s0086g00490.t01 | 559 | Putative uncharacterized protein | 50 | 6.0400793 | 11 | 1.7890037 | 1.7554111 | 6.96E-05 | 2.79E-05 | up |
| VIT_16s0013g00040.t01 | 489 | non-specific lipid-transfer protein | 50 | 6.9047123 | 11 | 2.0450984 | 1.7554111 | 6.96E-05 | 2.79E-05 | up |
| VIT_16s0013g01950.t01 | 1640 | transporter family protein, putative, expressed | 591 | 24.334817 | 130 | 7.206591 | 1.755633 | 6.72E-46 | 4.79E-47 | up |
| VIT_13s0064g01210.t01 | 822 | zinc finger A20 and AN1 domain-containing stress-associated protein, putative, expr | 1223 | 100.47062 | 269 | 29.751635 | 1.7557328 | 5.98E-94 | 2.12E-95 | up |
| VIT_12s0057g01330.t01 | 750 | zinc finger, C3HC4 type domain containing protein, expressed | 633 | 56.993705 | 139 | 16.849379 | 1.7581071 | 3.50E-49 | 2.33E-50 | up |
| VIT_04s0008g00440.t01 | 3090 | receptor-like protein kinase 5 precursor, putative, expressed | 451 | 9.8560411 | 99 | 2.912776 | 1.7586135 | 2.39E-35 | 2.14E-36 | up |
| VIT_08s0007g03010.t01 | 1154 | mitochondrion protein, putative, expressed | 187 | 10.942593 | 41 | 3.2300439 | 1.760329 | 2.67E-15 | 4.74E-16 | up |
| VIT_00s0265g00130.t01 | 958 | Function unknown | 316 | 22.2744 | 69 | 6.5480797 | 1.7662428 | 3.20E-25 | 3.78E-26 | up |
| VIT_07s0031g00050.t01 | 1499 | oryzain beta chain precursor, putative, expressed | 21936 | 988.18953 | 4789 | 290.45147 | 1.7664906 | 0 | 0 | up |
| VIT_08s0040g01570.t01 | 1038 | protein kinase APK1A, chloroplast precursor, putative, expressed | 78 | 5.0743649 | 17 | 1.4889563 | 1.7689259 | 4.57E-07 | 1.43E-07 | up |
| VIT_10s0003g04470.t01 | 5321 | ABC transporter C family member 12-like | 606 | 7.6906635 | 132 | 2.2553349 | 1.7697664 | 1.49E-47 | 1.03E-48 | up |
| VIT_18s0001g13070.t01 | 1314 | NAD dependent epimerase/dehydratase family protein, putative, expressed | 124 | 6.3725135 | 27 | 1.8680942 | 1.7702953 | 1.36E-10 | 3.21E-11 | up |
| VIT_18s0001g08810.t01 | 993 | Putative uncharacterized protein | 469 | 31.89393 | 102 | 9.3385897 | 1.7720053 | 4.27E-37 | 3.68E-38 | up |
| VIT_00s1682g00010.t01 | 617 | amine oxidase | 276 | 30.207053 | 60 | 8.8408996 | 1.7726204 | 2.86E-22 | 3.76E-23 | up |
| VIT_10s0003g01600.t01 | 1334 | WRKY transcription factor 65 | 1018 | 51.531928 | 221 | 15.061451 | 1.7746058 | 1.64E-79 | 6.84E-81 | up |
| VIT_06s0004g06350.t01 | 669 | pleckstrin homology domain-containing protein, putative, expressed | 1602 | 161.70403 | 347 | 47.155649 | 1.7778531 | 6.24E-125 | 1.67E-126 | up |
| VIT_06s0004g00590.t01 | 811 | uncharacterized protein PA4923, putative, expressed | 185 | 15.404064 | 40 | 4.4840403 | 1.7804399 | 2.27E-15 | 4.01E-16 | up |
| VIT_03s0038g00280.t01 | 1324 | cysteine proteinase 1 precursor, putative, expressed | 30271 | 1543.9144 | 6544 | 449.35096 | 1.7806782 | 0 | 0 | up |
| VIT_19s0014g04650.t01 | 823 | EF hand family protein, putative, expressed | 7816 | 641.31169 | 1687 | 186.35696 | 1.782957 | 0 | 0 | up |
| VIT_01s0010g03860.t01 | 397 | Putative uncharacterized protein | 1932 | 328.62535 | 417 | 95.493964 | 1.7829623 | 4.38E-151 | 9.51E-153 | up |
| VIT_11s0016g01510.t01 | 1511 | PHD finger protein ALFIN-LIKE 4 | 153 | 6.8377215 | 33 | 1.9855455 | 1.7839802 | 6.27E-13 | 1.25E-13 | up |
| VIT_01s0010g02950.t01 | 423 | OsCML31 - Calmodulin-related calcium sensor protein, expressed | 102 | 16.283368 | 22 | 4.7283834 | 1.7839802 | 5.40E-09 | 1.44E-09 | up |
| VIT_18s0041g02060.t01 | 1244 | 12-oxophytodienoate reductase, putative, expressed | 181 | 9.825228 | 39 | 2.8501952 | 1.7854302 | 4.05E-15 | 7.29E-16 | up |
| VIT_00s0203g00050.t01 | 1287 | wound induced protein-like (WIP) | 901 | 47.274907 | 194 | 13.704196 | 1.786457 | 3.93E-71 | 1.86E-72 | up |
| VIT_12s0057g01450.t01 | 2384 | subtilisin-like protease SBT5.3 | 516 | 14.615978 | 111 | 4.2329886 | 1.7877979 | 3.37E-41 | 2.63E-42 | up |
| VIT_17s0000g01440.t01 | 942 | Putative uncharacterized protein | 107 | 7.6703877 | 23 | 2.2197666 | 1.7888915 | 2.06E-09 | 5.31E-10 | up |
| VIT_13s0147g00120.t01 | 3013 | NBS-LRR disease resistance protein, putative, expressed | 270 | 6.0513055 | 58 | 1.7500854 | 1.7898211 | 4.36E-22 | 5.75E-23 | up |
| VIT_17s0000g10370.t01 | 580 | Putative uncharacterized protein | 303 | 35.277604 | 65 | 10.188629 | 1.7917927 | 1.08E-24 | 1.30E-25 | up |
| VIT_18s0001g15140.t01 | 1322 | Putative uncharacterized protein | 317 | 16.192438 | 68 | 4.6763588 | 1.7918627 | 8.75E-26 | 1.01E-26 | up |
| VIT_10s0003g00580.t01 | 1058 | pathogenesis-related genes transcriptional activator PTI5-like | 387 | 24.700727 | 83 | 7.1321882 | 1.7921368 | 2.95E-31 | 2.93E-32 | up |
| VIT_18s0001g03910.t01 | 3012 | nitrate reductase, putative, expressed | 14277 | 320.08582 | 3060 | 92.362745 | 1.7930758 | 0 | 0 | up |
| VIT_06s0061g00510.t01 | 1060 | Putative uncharacterized protein | 140 | 8.9188039 | 30 | 2.5730354 | 1.7933789 | 5.26E-12 | 1.12E-12 | up |
| VIT_14s0060g02390.t01 | 1015 | Putative uncharacterized protein | 168 | 11.177063 | 36 | 3.224533 | 1.7933789 | 3.46E-14 | 6.48E-15 | up |
| VIT_17s0000g00810.t01 | 621 | ZF-HD homeobox protein, putative, expressed | 56 | 6.0894893 | 12 | 1.7567907 | 1.7933789 | 1.82E-05 | 6.79E-06 | up |
| VIT_16s0050g01710.t01 | 585 | Protein kinase domain containing protein, expressed | 103 | 11.889561 | 22 | 3.4189849 | 1.7980554 | 3.71E-09 | 9.72E-10 | up |
| VIT_00s1602g00010.t01 | 351 | UDP-glucoronosyl and UDP-glucosyl transferase domain containing protein, express | 61 | 11.73565 | 13 | 3.3671821 | 1.8012841 | 6.92E-06 | 2.47E-06 | up |
| VIT_14s0083g00260.t01 | 826 | Putative uncharacterized protein | 20951 | 1712.8099 | 4459 | 490.78106 | 1.8032135 | 0 | 0 | up |
| VIT_08s0007g00470.t01 | 1351 | OsRhmbd18 - Putative Rhomboid homologue, expressed | 658 | 32.889327 | 140 | 9.4211314 | 1.8036473 | 6.93E-53 | 4.29E-54 | up |
| VIT_16s0013g02160.t01 | 493 | Putative uncharacterized protein | 480 | 65.747427 | 102 | 18.809776 | 1.8054518 | 6.61E-39 | 5.45E-40 | up |
| VIT_04s0008g00120.t01 | 994 | class I extracellular chitinase (CHI1B) | 325 | 22.079103 | 69 | 6.3109259 | 1.806758 | 1.06E-26 | 1.20E-27 | up |
| VIT_16s0050g00410.t01 | 913 | Putative uncharacterized protein | 212 | 15.680125 | 45 | 4.4809707 | 1.8070539 | 8.57E-18 | 1.35E-18 | up |
| VIT_05s0049g01750.t01 | 1598 | Putative uncharacterized protein | 165 | 6.9725496 | 35 | 1.991231 | 1.8080257 | 4.24E-14 | 7.99E-15 | up |
| VIT_07s0095g00170.t01 | 463 | Putative uncharacterized protein | 66 | 9.6260339 | 14 | 2.7490169 | 1.8080257 | 2.64E-06 | 8.98E-07 | up |
| VIT_04s0069g00970.t01 | 593 | WRKY transcription factor 51 | 118 | 13.437292 | 25 | 3.8327959 | 1.8097734 | 2.06E-10 | 4.92E-11 | up |
| VIT_14s0068g02070.t01 | 1320 | peptidase, putative, expressed | 118 | 6.0366017 | 25 | 1.7218545 | 1.8097734 | 2.06E-10 | 4.92E-11 | up |
| VIT_08s0007g08410.t01 | 1117 | 4,5-DOPA dioxygenase extradiol, putative, expressed | 846 | 51.144817 | 179 | 14.569016 | 1.8116846 | 3.98E-68 | 1.94E-69 | up |
| VIT_06s0004g04660.t01 | 614 | metallothionein-like protein 1 | 36393 | 4002.5239 | 7692 | 1138.9411 | 1.8132168 | 0 | 0 | up |
| VIT_06s0061g00850.t01 | 1016 | harpin-induced protein 1 domain containing protein, expressed | 516 | 34.29576 | 109 | 9.7535601 | 1.8140294 | 5.18E-42 | 3.97E-43 | up |
| VIT_04s0008g00610.t01 | 1539 | zinc finger CCCH type family protein, putative, expressed | 3085 | 135.36332 | 650 | 38.397691 | 1.8177454 | 2.85E-247 | 3.69E-249 | up |
| VIT_13s0158g00150.t01 | 1815 | TKL_IRAK_CR4L.5 - The CR4L subfamily has homology with Crinkly4, expresse | 1040 | 38.69378 | 219 | 10.969778 | 1.8185673 | 5.79E-84 | 2.30E-85 | up |
| VIT_17s0000g05880.t01 | 803 | Ser/Thr protein phosphatase family protein, putative, expressed | 975 | 81.992384 | 205 | 23.209655 | 1.8207648 | 6.70E-79 | 2.81E-80 | up |
| VIT_03s0063g00060.t01 | 1161 | CBS domain containing membrane protein, putative, expressed | 176 | 10.236816 | 37 | 2.8973428 | 1.8209648 | 4.18E-15 | 7.52E-16 | up |
| VIT_19s0090g00430.t01 | 498 | ubiquitin-like protein 5 | 3399 | 460.89953 | 714 | 130.34646 | 1.8221009 | 2.78E-273 | 3.38E-275 | up |
| VIT_05s0020g00290.t01 | 1193 | zinc knuckle family protein, expressed | 100 | 5.6603593 | 21 | 1.6003288 | 1.8225253 | 4.54E-09 | 1.20E-09 | up |
| VIT_16s0050g02700.t01 | 1333 | Protein kinase domain containing protein, expressed | 100 | 5.065873 | 21 | 1.4322523 | 1.8225253 | 4.54E-09 | 1.20E-09 | up |
| VIT_01s0011g02510.t01 | 958 | DNL zinc finger domain containing protein, putative, expressed | 81 | 5.7095772 | 17 | 1.613295 | 1.8233737 | 1.47E-07 | 4.40E-08 | up |
| VIT_13s0019g01650.t01 | 843 | expansin precursor, putative, expressed | 453 | 36.287335 | 95 | 10.245341 | 1.8244981 | 2.71E-37 | 2.32E-38 | up |
| VIT_19s0015g00690.t01 | 634 | NOI protein, nitrate-induced, putative, expressed | 2510 | 267.34306 | 526 | 75.427004 | 1.8255392 | 1.71E-202 | 2.73E-204 | up |
| VIT_17s0000g09810.t01 | 1266 | pectate lyase precursor, putative, expressed | 816 | 43.525212 | 171 | 12.279842 | 1.8255593 | 2.00E-66 | 9.99E-68 | up |
| VIT_02s0025g00330.t01 | 1521 | Putative uncharacterized protein | 1384 | 61.445675 | 290 | 17.334015 | 1.8257056 | 4.92E-112 | 1.46E-113 | up |
| VIT_02s0033g00870.t01 | 1196 | bifunctional nitrilase/nitrile hydratase | 411 | 23.205722 | 86 | 6.5372884 | 1.8277163 | 5.21E-34 | 4.82E-35 | up |
| VIT_13s0019g02660.t01 | 672 | Putative uncharacterized protein | 134 | 13.465422 | 28 | 3.7880799 | 1.8297208 | 7.86E-12 | 1.69E-12 | up |
| VIT_16s0022g00810.t01 | 620 | Putative uncharacterized protein | 240 | 26.139904 | 50 | 7.3317675 | 1.8340209 | 2.17E-20 | 3.07E-21 | up |
| VIT_13s0019g03920.t01 | 1668 | elongation factor Tu, putative, expressed | 21834 | 883.93779 | 4548 | 247.88759 | 1.8342588 | 0 | 0 | up |
| VIT_13s0047g00210.t01 | 1023 | flavanone 3-dioxygenase-like | 1460 | 96.374395 | 304 | 27.016453 | 1.8348116 | 6.07E-119 | 1.72E-120 | up |
| VIT_06s0061g00970.t01 | 2103 | OsProCP1 - Putative Lysosomal Pro-x Carboxypeptidase homologue, expressed | 341 | 10.949633 | 71 | 3.0693714 | 1.8348673 | 1.55E-28 | 1.65E-29 | up |
| VIT_08s0007g01070.t01 | 1542 | DC1 domain-containing protein, putative, expressed | 202 | 8.8460917 | 42 | 2.4762546 | 1.8368806 | 2.29E-17 | 3.68E-18 | up |
| VIT_14s0068g00470.t01 | 599 | UDP-glucoronosyl and UDP-glucosyl transferase domain containing protein, express | 53 | 5.9749392 | 11 | 1.6695377 | 1.8394753 | 2.22E-05 | 8.40E-06 | up |
| VIT_04s0008g01480.t01 | 1885 | NOL1/NOP2/sun family protein, putative, expressed | 747 | 26.760467 | 155 | 7.4756802 | 1.8398265 | 1.58E-61 | 8.44E-63 | up |
| VIT_17s0000g05550.t01 | 5353 | peptide transporter PTR2, putative, expressed | 2194 | 27.677306 | 455 | 7.7275981 | 1.8406116 | 4.28E-179 | 7.98E-181 | up |
| VIT_00s0391g00040.t01 | 1263 | OsFBX192 - F-box domain containing protein, expressed | 1362 | 72.821262 | 282 | 20.299069 | 1.8429461 | 1.11E-111 | 3.33E-113 | up |
| VIT_08s0056g00830.t01 | 1405 | presenilin, putative, expressed | 348 | 16.725818 | 72 | 4.6589339 | 1.844005 | 2.72E-29 | 2.83E-30 | up |
| VIT_00s1328g00010.t01 | 484 | Putative uncharacterized protein | 58 | 8.0922087 | 12 | 2.2540641 | 1.844005 | 8.47E-06 | 3.04E-06 | up |
| VIT_17s0000g01400.t01 | 2066 | nucleotide pyrophosphatase/phosphodiesterase, putative, expressed | 542 | 17.7155 | 112 | 4.9285376 | 1.8457806 | 4.09E-45 | 2.97E-46 | up |
| VIT_02s0025g03160.t01 | 486 | nuclear pore complex protein NUP98A | 92 | 12.783095 | 19 | 3.5542478 | 1.846621 | 1.45E-08 | 3.99E-09 | up |
| VIT_02s0109g00370.t01 | 713 | RNA recognition motif containing protein, putative, expressed | 126 | 11.933435 | 26 | 3.315234 | 1.8478267 | 2.50E-11 | 5.59E-12 | up |
| VIT_05s0102g00190.t01 | 696 | Putative uncharacterized protein | 617 | 59.863261 | 127 | 16.589177 | 1.8514285 | 2.01E-51 | 1.28E-52 | up |
| VIT_14s0006g02570.t01 | 1460 | non-specific lipid-transfer protein 2-like | 3334 | 154.20455 | 686 | 42.717087 | 1.8519601 | 4.67E-274 | 5.63E-276 | up |
| VIT_12s0059g02150.t01 | 3387 | aconitate hydratase protein, putative, expressed | 1672 | 33.335388 | 344 | 9.2336545 | 1.8520809 | 8.17E-138 | 1.93E-139 | up |
| VIT_05s0077g01140.t01 | 982 | ocs element-binding factor 1-like | 248 | 17.053936 | 51 | 4.7215986 | 1.8527575 | 2.61E-21 | 3.55E-22 | up |
| VIT_16s0013g00450.t01 | 804 | Putative uncharacterized protein | 1296 | 108.85124 | 266 | 30.078485 | 1.8555541 | 2.74E-107 | 8.47E-109 | up |
| VIT_14s0030g01980.t01 | 1419 | probable aldo-keto reductase 1 | 1019 | 48.492685 | 209 | 13.390422 | 1.8565657 | 1.30E-84 | 5.11E-86 | up |
| VIT_15s0048g01390.t01 | 1469 | CXE carboxylesterase, putative, expressed | 1809 | 83.15746 | 371 | 22.960561 | 1.8566878 | 1.51E-149 | 3.33E-151 | up |
| VIT_19s0085g00010.t01 | 700 | auxin-induced protein X15 | 113 | 10.900963 | 23 | 2.9871716 | 1.8676035 | 2.08E-10 | 4.97E-11 | up |
| VIT_08s0007g03320.t01 | 615 | acetyltransferase, GNAT family, putative, expressed | 59 | 6.4783042 | 12 | 1.7739301 | 1.8686671 | 5.74E-06 | 2.03E-06 | up |
| VIT_11s0065g00350.t01 | 1688 | cinnamate-4-hydroxylase | 128 | 5.1206132 | 26 | 1.4003329 | 1.8705468 | 1.15E-11 | 2.52E-12 | up |
| VIT_16s0013g01090.t01 | 1027 | ethylene-responsive transcription factor 5-like | 340 | 22.355939 | 69 | 6.1081405 | 1.871853 | 3.29E-29 | 3.43E-30 | up |
| VIT_12s0059g01960.t01 | 715 | nucleolin | 69 | 6.5166965 | 14 | 1.7801327 | 1.872156 | 8.35E-07 | 2.69E-07 | up |
| VIT_01s0011g01170.t01 | 264 | haloacid dehalogenase-like hydrolase domain-containing-like | 69 | 17.649386 | 14 | 4.8211926 | 1.872156 | 8.35E-07 | 2.69E-07 | up |
| VIT_01s0011g01350.t01 | 752 | Putative uncharacterized protein | 138 | 12.392122 | 28 | 3.3850927 | 1.872156 | 1.69E-12 | 3.48E-13 | up |
| VIT_00s1291g00020.t01 | 1062 | tetratricopeptide repeat domain containing protein, expressed | 833 | 52.966945 | 169 | 14.467469 | 1.8722798 | 3.96E-70 | 1.89E-71 | up |
| VIT_13s0047g00690.t01 | 465 | Putative uncharacterized protein | 79 | 11.472514 | 16 | 3.1282208 | 1.8747673 | 1.22E-07 | 3.61E-08 | up |
| VIT_07s0129g00240.t01 | 1128 | ZOS12-09 - C2H2 zinc finger protein, expressed | 652 | 39.032192 | 132 | 10.638863 | 1.8753205 | 3.24E-55 | 1.92E-56 | up |
| VIT_06s0004g07850.t01 | 697 | Putative uncharacterized protein | 1082 | 104.82839 | 219 | 28.565492 | 1.8756842 | 4.96E-91 | 1.83E-92 | up |
| VIT_08s0007g03460.t01 | 925 | Yip1 domain containing protein, expressed | 84 | 6.1322803 | 17 | 1.6708504 | 1.8758411 | 4.63E-08 | 1.32E-08 | up |

| | | | | | | | | | | |
|---|---|---|---|---|---|---|---|---|---|---|
| VIT_13s0019g03230.t01 | 634 | immediate early response 3-interacting protein 1-like | 624 | 66.462975 | 126 | 18.068066 | 1.8791088 | 5.00E-53 | 3.08E-54 | up |
| VIT_19s0090g01390.t01 | 1140 | wax synthase isoform 1, putative, expressed | 372 | 22.035481 | 75 | 5.9811788 | 1.8813266 | 4.49E-32 | 4.36E-33 | up |
| VIT_13s0156g00060.t01 | 1689 | serine/threonine-protein phosphatase PP2A-3 catalytic subunit-like | 2169 | 86.719017 | 436 | 23.468602 | 1.8856165 | 6.29E-183 | 1.15E-184 | up |
| VIT_01s0010g03360.t01 | 697 | signal peptidase complex subunit 1, putative, expressed | 1246 | 120.71735 | 250 | 32.609009 | 1.8882906 | 1.24E-105 | 3.93E-107 | up |
| VIT_13s0067g02630.t01 | 550 | Putative uncharacterized protein | 1416 | 173.85413 | 284 | 46.944641 | 1.8888449 | 5.66E-120 | 1.58E-121 | up |
| VIT_03s0017g02260.t01 | 791 | proline-rich protein 1 (PRP1) | 2333 | 199.16944 | 467 | 53.674841 | 1.8916784 | 1.54E-197 | 2.54E-199 | up |
| VIT_10s0116g01040.t01 | 709 | Putative uncharacterized protein | 1790 | 170.48699 | 358 | 45.905758 | 1.8929146 | 7.13E-152 | 1.54E-153 | up |
| VIT_18s0001g05030.t01 | 1728 | glucan 1,3-beta-glucosidase A | 391 | 15.279793 | 78 | 4.1037532 | 1.8966091 | 5.13E-34 | 4.73E-35 | up |
| VIT_10s0003g01830.t01 | 979 | aquaporin NIP1-2-like | 346 | 23.865902 | 69 | 6.4076203 | 1.8970903 | 3.13E-30 | 3.19E-31 | up |
| VIT_07s0031g00160.t01 | 887 | SUMO-conjugating enzyme SCE1-like | 5587 | 425.3432 | 1113 | 114.078 | 1.8986067 | 0 | 0 | up |
| VIT_04s0008g04700.t01 | 704 | probable histone H2A variant 3-like | 504 | 48.343971 | 100 | 12.913909 | 1.9044102 | 8.62E-44 | 6.42E-45 | up |
| VIT_11s0037g00590.t01 | 838 | spermidine hydroxycinnamoyl transferase-like | 242 | 19.500951 | 48 | 5.2074798 | 1.9048872 | 1.42E-21 | 1.91E-22 | up |
| VIT_18s0072g00150.t01 | 604 | Putative uncharacterized protein | 520 | 58.136763 | 103 | 15.503532 | 1.9068538 | 3.17E-45 | 2.30E-46 | up |
| VIT_06s0061g01490.t01 | 4377 | pleiotropic drug resistance protein 2-like | 409 | 6.3100268 | 81 | 1.6824371 | 1.9070935 | 8.56E-36 | 7.61E-37 | up |
| VIT_16s0013g01110.t01 | 2139 | ethylene-responsive transcription factor 5-like | 981 | 30.970104 | 194 | 8.2455821 | 1.909183 | 1.39E-84 | 5.47E-86 | up |
| VIT_14s0060g00650.t01 | 567 | Putative uncharacterized protein | 86 | 10.242355 | 17 | 2.7258141 | 1.9097884 | 2.11E-08 | 5.88E-09 | up |
| VIT_01s0011g02320.t01 | 1348 | Putative uncharacterized protein | 228 | 11.421664 | 45 | 3.0349602 | 1.9120234 | 1.74E-20 | 2.45E-21 | up |
| VIT_04s0008g00550.t01 | 1124 | Putative uncharacterized protein | 147 | 8.8315202 | 29 | 2.3456438 | 1.9126779 | 1.35E-13 | 2.60E-14 | up |
| VIT_08s0040g02220.t01 | 590 | DNA-binding protein S1FA | 1394 | 159.54941 | 275 | 42.375131 | 1.9127135 | 4.09E-120 | 1.14E-121 | up |
| VIT_14s0066g02170.t01 | 1183 | prolyl 4-hydroxylase subunit alpha-1-like | 365 | 20.834955 | 72 | 5.5332224 | 1.9128142 | 3.55E-32 | 3.43E-33 | up |
| VIT_00s0338g00030.t01 | 2010 | ganglioside-induced differentiation-associated protein 2-like | 528 | 17.738721 | 104 | 4.7040037 | 1.9149409 | 3.70E-46 | 2.62E-47 | up |
| VIT_13s0019g03160.t01 | 676 | 18.1 kDa class I heat shock protein | 66 | 6.5929789 | 13 | 1.7483446 | 1.9149409 | 1.00E-06 | 3.26E-07 | up |
| VIT_00s0299g00100.t01 | 1356 | homeobox-leucine zipper protein HAT4-like | 1239 | 61.701548 | 243 | 16.292096 | 1.9211345 | 1.78E-107 | 5.50E-109 | up |
| VIT_02s0033g00010.t01 | 549 | transmembrane protein 45B-like | 153 | 18.819303 | 30 | 4.9679736 | 1.9214838 | 3.45E-14 | 6.46E-15 | up |
| VIT_10s0003g05430.t01 | 356 | reticuline oxidase-like protein precursor, putative, expressed | 301 | 57.095376 | 59 | 15.067194 | 1.9219631 | 7.02E-27 | 7.87E-28 | up |
| VIT_11s0016g02450.t01 | 1095 | serine acetyltransferase 5-like | 1541 | 95.032677 | 302 | 25.073975 | 1.9222329 | 1.46E-133 | 3.59E-135 | up |
| VIT_05s0051g00330.t01 | 941 | Putative uncharacterized protein | 286 | 20.523946 | 56 | 5.4103925 | 1.9235029 | 1.28E-25 | 1.49E-26 | up |
| VIT_07s0031g00080.t01 | 1218 | WRKY transcription factor 7 | 1426 | 79.059976 | 279 | 20.825109 | 1.9246235 | 7.21E-124 | 1.95E-125 | up |
| VIT_18s0001g01110.t01 | 1385 | zeaxanthin epoxidase | 757 | 36.908853 | 148 | 9.714989 | 1.9256826 | 3.12E-66 | 1.57E-67 | up |
| VIT_07s0005g00410.t01 | 3230 | beta-glucosidase 11-like | 880 | 18.397745 | 172 | 4.8412365 | 1.9260815 | 7.41E-77 | 3.19E-78 | up |
| VIT_14s0066g02610.t01 | 727 | Putative uncharacterized protein | 3761 | 349.34406 | 734 | 91.789292 | 1.9282508 | 0 | 0 | up |
| VIT_04s0023g01220.t01 | 1356 | dolichyl-diphosphooligosaccharide--protein glycosyltransferase 48 kDa subunit-like | 164 | 8.1671137 | 32 | 2.1454612 | 1.9285385 | 3.34E-15 | 5.98E-16 | up |
| VIT_17s0000g08150.t01 | 847 | helix-loop-helix DNA-binding domain containing protein, expressed | 123 | 9.8063219 | 24 | 2.5760732 | 1.9285385 | 1.12E-11 | 2.46E-12 | up |
| VIT_10s0116g01030.t01 | 715 | STIGL1 - Stigma-specific Stig1 family protein precursor, expressed | 888 | 83.86705 | 173 | 21.997353 | 1.9307741 | 8.52E-78 | 3.63E-79 | up |
| VIT_03s0063g00830.t01 | 1122 | 2-hydroxyisoflavanone dehydratase | 216 | 13.000059 | 42 | 3.4031948 | 1.9335566 | 9.68E-20 | 1.40E-20 | up |
| VIT_03s0017g02110.t01 | 1585 | putative UDP-glucose flavonoid 3-O-glucosyltransferase 3-like | 294 | 12.525714 | 57 | 3.2694595 | 1.9377688 | 1.48E-26 | 1.68E-27 | up |
| VIT_07s0031g02250.t01 | 872 | RING-H2 finger protein, putative, expressed | 713 | 55.215052 | 138 | 14.387753 | 1.9402203 | 4.73E-63 | 2.46E-64 | up |
| VIT_15s0045g00380.t01 | 1744 | phospholipase C 3-like | 636 | 24.626068 | 123 | 6.4119334 | 1.941355 | 2.16E-56 | 1.25E-57 | up |
| VIT_18s0001g15730.t01 | 1149 | dof zinc finger protein DOF3.5-like | 88 | 5.1718639 | 17 | 1.3451145 | 1.9429553 | 9.60E-09 | 2.60E-09 | up |
| VIT_01s0010g03020.t01 | 418 | Calmodulin-related calcium sensor protein, expressed | 88 | 14.216439 | 17 | 3.697456 | 1.9429553 | 9.60E-09 | 2.60E-09 | up |
| VIT_06s0080g00590.t01 | 594 | harpin-induced protein 1 domain containing protein, expressed | 171 | 19.439904 | 33 | 5.0507732 | 1.9444449 | 5.79E-16 | 9.92E-17 | up |
| VIT_11s0016g05040.t01 | 964 | transmembrane protein 128 | 228 | 15.971373 | 44 | 4.1495979 | 1.9444449 | 6.36E-21 | 8.76E-22 | up |
| VIT_13s0019g04880.t01 | 1148 | Putative uncharacterized protein | 114 | 6.7057507 | 22 | 1.7422528 | 1.9444449 | 5.20E-11 | 1.19E-11 | up |
| VIT_18s0041g00930.t01 | 951 | UDP-glucoronosyl and UDP-glucosyl transferase domain containing protein, express | 109 | 7.7398122 | 21 | 2.0075628 | 1.9468534 | 1.36E-10 | 3.21E-11 | up |
| VIT_08s0007g02690.t01 | 718 | momilactone A synthase-like | 177 | 16.646896 | 34 | 4.3051159 | 1.9511292 | 1.48E-16 | 2.47E-17 | up |
| VIT_15s0048g00740.t01 | 894 | Putative uncharacterized protein | 73 | 5.5140384 | 14 | 1.4237079 | 1.9534561 | 1.74E-07 | 5.23E-08 | up |
| VIT_12s0028g01030.t01 | 766 | Putative uncharacterized protein | 120 | 10.578812 | 23 | 2.7297913 | 1.9543151 | 1.33E-11 | 2.93E-12 | up |
| VIT_09s0002g00220.t01 | 1246 | zinc finger, C3HC4 type domain containing protein, expressed | 1221 | 66.173189 | 234 | 17.073721 | 1.9544693 | 2.34E-108 | 7.20E-110 | up |
| VIT_04s0008g06930.t01 | 735 | VQ domain containing protein, putative, expressed | 334 | 30.686232 | 64 | 7.9163139 | 1.9546908 | 2.30E-30 | 2.34E-31 | up |
| VIT_14s0060g00970.t01 | 413 | Putative uncharacterized protein | 570 | 93.19857 | 109 | 23.99423 | 1.9576203 | 3.37E-51 | 2.17E-52 | up |
| VIT_00s0218g00220.t01 | 1724 | UDP-glycosyltransferase 91A1-like | 633 | 24.794245 | 121 | 6.3808492 | 1.958185 | 9.13E-57 | 5.28E-58 | up |
| VIT_00s0214g00150.t01 | 1104 | F-box protein PP2-B1-like | 152 | 9.2973452 | 29 | 2.3881373 | 1.960933 | 1.85E-14 | 3.42E-15 | up |
| VIT_12s0134g00550.t01 | 1725 | equilibrative nucleoside transporter 4-like | 252 | 9.8649726 | 48 | 2.5297786 | 1.9633039 | 2.70E-23 | 3.41E-24 | up |
| VIT_14s0108g01360.t01 | 898 | Putative uncharacterized protein | 84 | 6.3166584 | 16 | 1.6198471 | 1.9633039 | 1.69E-08 | 4.67E-09 | up |
| VIT_01s0011g03660.t01 | 1171 | Putative uncharacterized protein | 771 | 44.461276 | 146 | 11.335126 | 1.971749 | 1.48E-69 | 7.11E-71 | up |
| VIT_08s0007g06000.t01 | 1140 | Beta-1,3-glucanase | 217 | 12.85403 | 41 | 3.2697111 | 1.9749857 | 2.32E-20 | 3.29E-21 | up |
| VIT_09s0002g04620.t01 | 539 | Putative uncharacterized protein | 323 | 40.466738 | 61 | 10.288959 | 1.9756395 | 8.47E-30 | 8.71E-31 | up |
| VIT_13s0019g04610.t01 | 1688 | protein kinase APK1A, chloroplast precursor, putative, expressed | 143 | 5.720685 | 27 | 1.4541918 | 1.9759703 | 8.55E-14 | 1.64E-14 | up |
| VIT_00s0218g00150.t01 | 3188 | glucosyltransferase, putative, expressed | 583 | 12.349082 | 110 | 3.1369294 | 1.9769789 | 5.20E-53 | 3.21E-54 | up |
| VIT_12s0057g00170.t01 | 588 | wound induced protein, putative, expressed | 53 | 6.0867153 | 10 | 1.5461551 | 1.9769789 | 8.19E-06 | 2.94E-06 | up |
| VIT_19s0177g00270.t01 | 1245 | Putative uncharacterized protein | 827 | 44.856006 | 156 | 11.391623 | 1.9773278 | 8.27E-75 | 3.67E-76 | up |
| VIT_09s0002g08420.t01 | 916 | jp18, putative, expressed | 1670 | 123.11343 | 315 | 31.264066 | 1.9774109 | 3.58E-150 | 7.88E-152 | up |
| VIT_06s0004g03840.t01 | 1196 | chitinase 2-like | 865 | 48.839293 | 163 | 12.390442 | 1.9788147 | 2.81E-78 | 1.19E-79 | up |
| VIT_06s0004g05730.t01 | 777 | universal stress protein A-like protein | 744 | 64.660098 | 140 | 16.380886 | 1.9808623 | 1.49E-67 | 7.31E-69 | up |
| VIT_02s0033g00020.t01 | 580 | transmembrane protein 45B-like | 133 | 15.484889 | 25 | 3.9187033 | 1.9824128 | 5.93E-13 | 1.18E-13 | up |
| VIT_15s0046g00430.t01 | 594 | harpin-induced protein 1 domain containing protein, expressed | 144 | 16.370445 | 27 | 4.1324508 | 1.986024 | 5.73E-14 | 1.08E-14 | up |
| VIT_18s0001g02050.t01 | 2597 | WD40-like Beta Propeller Repeat family protein, expressed | 3217 | 83.64954 | 603 | 21.109392 | 1.9864725 | 2.31E-290 | 2.72E-292 | up |
| VIT_01s0137g00240.t01 | 1556 | probable pectate lyase 5-like | 923 | 40.056828 | 173 | 10.108038 | 1.9865451 | 6.84E-84 | 2.72E-85 | up |
| VIT_01s0011g06080.t01 | 2533 | G-type lectin S-receptor-like serine/threonine-protein kinase | 1913 | 50.999301 | 358 | 12.849263 | 1.9887919 | 3.36E-173 | 6.34E-175 | up |
| VIT_00s0477g00010.t01 | 592 | extensin-like | 326 | 37.186075 | 61 | 9.3678192 | 1.9889773 | 2.52E-30 | 2.57E-31 | up |
| VIT_19s0014g03090.t01 | 1668 | CBS domain-containing protein CBSX5-like | 326 | 13.197935 | 61 | 3.3247896 | 1.9889773 | 2.52E-30 | 2.57E-31 | up |
| VIT_06s0004g02860.t01 | 625 | Putative uncharacterized protein | 316 | 34.142201 | 59 | 8.5822738 | 1.9921242 | 1.75E-29 | 1.81E-30 | up |
| VIT_07s0031g01530.t01 | 761 | stress enhanced protein 2 | 596 | 52.886649 | 111 | 13.260769 | 1.9957392 | 7.82E-55 | 4.67E-56 | up |
| VIT_11s0016g01070.t01 | 1377 | thiol protease aleurain-like | 22431 | 1100.0163 | 4156 | 274.39233 | 2.0032129 | 0 | 0 | up |
| VIT_06s0004g03910.t01 | 668 | Putative uncharacterized protein | 135 | 13.647143 | 25 | 3.402467 | 2.0039459 | 2.64E-13 | 5.17E-14 | up |
| VIT_01s0011g01740.t01 | 537 | phloem protein 2-LIKE A9 | 54 | 6.7905338 | 10 | 1.6929966 | 2.0039459 | 5.46E-06 | 1.93E-06 | up |
| VIT_03s0063g00400.t01 | 1392 | alpha-amylase-like | 184 | 8.9261264 | 34 | 2.2205986 | 2.0070856 | 8.91E-18 | 1.40E-18 | up |
| VIT_04s0008g01550.t01 | 877 | 17.3 kDa class II heat shock protein | 314 | 24.177673 | 58 | 6.012551 | 2.0076263 | 1.37E-29 | 1.41E-30 | up |
| VIT_12s0142g00690.t01 | 705 | mitogen-activated protein kinase kinase kinase 1-like | 157 | 15.03817 | 29 | 3.7397214 | 2.0076263 | 2.47E-15 | 4.38E-16 | up |
| VIT_06s0004g02790.t01 | 1189 | CASP-like protein 5A2 | 152 | 8.6326906 | 28 | 2.1409501 | 2.0115591 | 6.45E-15 | 1.17E-15 | up |
| VIT_00s0174g00070.t01 | 2074 | ankyrin repeat-containing protein | 163 | 5.3071736 | 30 | 1.3150518 | 2.0128241 | 6.30E-16 | 1.08E-16 | up |
| VIT_06s0004g08190.t01 | 1938 | pathogenesis-related genes transcriptional activator PTI6 | 1905 | 66.378227 | 350 | 16.418922 | 2.0153507 | 1.75E-175 | 3.29E-177 | up |
| VIT_04s0023g03080.t01 | 108 | Putative uncharacterized protein | 49 | 30.637743 | 9 | 7.5761598 | 2.0157714 | 1.44E-05 | 5.30E-06 | up |
| VIT_00s2814g00010.t01 | 242 | thiosulfate sulfurtransferase 16 | 49 | 13.673042 | 9 | 3.3810961 | 2.0157714 | 1.44E-05 | 5.30E-06 | up |
| VIT_12s0035g00490.t01 | 475 | disease resistance protein RGA2-like | 49 | 6.9660552 | 9 | 1.7225795 | 2.0157714 | 1.44E-05 | 5.30E-06 | up |
| VIT_02s0012g00690.t01 | 496 | Putative uncharacterized protein | 49 | 6.6711214 | 9 | 1.6496477 | 2.0157714 | 1.44E-05 | 5.30E-06 | up |
| VIT_03s0038g00660.t01 | 917 | Putative uncharacterized protein | 109 | 8.0267845 | 20 | 1.9828553 | 2.0172427 | 4.82E-11 | 1.10E-11 | up |
| VIT_07s0005g00540.t01 | 355 | ribosomal protein L7Ae, putative, expressed | 60 | 11.413198 | 11 | 2.817051 | 2.0184455 | 1.39E-06 | 4.61E-07 | up |
| VIT_18s0041g02260.t01 | 619 | 12-oxophytodienoate reductase 2-like(OPR) | 60 | 6.5455334 | 11 | 1.6155947 | 2.0184455 | 1.39E-06 | 4.61E-07 | up |
| VIT_14s0036g00520.t01 | 1505 | L-allo-threonine aldolase-like | 759 | 34.055693 | 139 | 8.3967007 | 2.0200015 | 1.16E-70 | 5.53E-72 | up |
| VIT_09s0002g00650.t01 | 829 | Putative uncharacterized protein | 269 | 21.912009 | 49 | 5.3736815 | 2.027739 | 8.08E-26 | 9.35E-27 | up |
| VIT_03s0167g00190.t01 | 1560 | cytochrome P450 734A1-like | 423 | 18.3105 | 77 | 4.4874177 | 2.0287138 | 4.89E-40 | 3.94E-41 | up |
| VIT_00s0203g00140.t01 | 1031 | PB1 domain containing protein, expressed | 121 | 7.9252167 | 22 | 1.9399672 | 2.0304181 | 3.15E-12 | 6.59E-13 | up |
| VIT_08s0007g00580.t01 | 605 | Putative uncharacterized protein | 66 | 7.3667003 | 12 | 1.8032513 | 2.0304181 | 3.56E-07 | 1.11E-07 | up |
| VIT_11s0052g01140.t01 | 589 | CASP-like protein | 281 | 32.216286 | 51 | 7.8720031 | 2.0329875 | 5.19E-27 | 5.79E-28 | up |
| VIT_10s0003g02620.t01 | 578 | AWPM-19-like membrane family protein, putative, expressed | 215 | 25.118579 | 39 | 6.1343301 | 2.0337771 | 6.31E-21 | 8.69E-22 | up |
| VIT_09s0018g01680.t01 | 1956 | Putative uncharacterized protein | 419 | 14.465372 | 76 | 3.5324426 | 2.0338654 | 8.50E-40 | 6.91E-41 | up |

| | | | | | | | | | | |
|---|---|---|---|---|---|---|---|---|---|---|
| VIT_00s1328g00020.t01 | 500 | phytosulfokines 3-like | 559 | 75.496401 | 101 | 18.364611 | 2.0394795 | 6.18E-53 | 3.82E-54 | up |
| VIT_10s0003g00880.t01 | 1769 | nudix hydrolase 15 | 543 | 20.727954 | 98 | 5.0364974 | 2.0410851 | 1.68E-51 | 1.07E-52 | up |
| VIT_15s0048g02710.t01 | 595 | Putative uncharacterized protein | 61 | 6.9230475 | 11 | 1.6807615 | 2.0422922 | 9.29E-07 | 3.01E-07 | up |
| VIT_12s0057g01110.t01 | 1015 | nudix hydrolase 2-like | 211 | 14.037858 | 38 | 3.4036738 | 2.0441582 | 1.10E-20 | 1.53E-21 | up |
| VIT_03s0017g01210.t01 | 1456 | phosphate-induced protein 1 conserved region domain containing protein, expressed | 1432 | 66.414986 | 256 | 15.984865 | 2.0548023 | 1.92E-135 | 4.65E-137 | up |
| VIT_00s0561g00020.t01 | 994 | stem-specific protein TSJT1-like | 224 | 15.217597 | 40 | 3.6585078 | 2.0564133 | 4.68E-22 | 6.19E-23 | up |
| VIT_00s0515g00030.t01 | 835 | Putative uncharacterized protein | 342 | 27.65821 | 61 | 6.6416155 | 2.0581017 | 3.71E-33 | 3.51E-34 | up |
| VIT_01s0026g01340.t01 | 1015 | glutathione S-transferase | 8564 | 569.76407 | 1527 | 136.77394 | 2.0585712 | 0 | 0 | up |
| VIT_12s0059g00190.t01 | 1227 | expansin-B3-like | 2334 | 128.45196 | 416 | 30.823301 | 2.0591356 | 7.29E-221 | 1.06E-222 | up |
| VIT_14s0068g00160.t01 | 544 | hypothetical protein | 219 | 27.18502 | 39 | 6.5177257 | 2.0603713 | 1.22E-21 | 1.64E-22 | up |
| VIT_01s0137g00660.t01 | 720 | ubiquinone biosynthesis protein COQ4 | 146 | 13.693195 | 26 | 3.2830026 | 2.0603713 | 8.66E-15 | 1.58E-15 | up |
| VIT_04s0008g02510.t01 | 1635 | Protein kinase domain containing protein, expressed | 506 | 20.8986 | 90 | 5.0044358 | 2.062127 | 1.09E-48 | 7.35E-50 | up |
| VIT_18s0001g08420.t01 | 361 | Putative uncharacterized protein | 265 | 49.570479 | 47 | 11.836438 | 2.0662462 | 4.74E-26 | 5.45E-27 | up |
| VIT_09s0002g03640.t01 | 4666 | ABC transporter G family member 29-like | 570 | 8.2492519 | 101 | 1.9679181 | 2.0675931 | 6.71E-55 | 4.00E-56 | up |
| VIT_19s0014g02770.t01 | 5647 | Putative uncharacterized protein | 3404 | 40.705792 | 603 | 9.7080029 | 2.0679876 | 0 | 0 | up |
| VIT_03s0063g00750.t01 | 1207 | probable carboxylesterase 2 | 2027 | 113.40467 | 359 | 27.040676 | 2.0682768 | 5.15E-193 | 8.93E-195 | up |
| VIT_13s0067g01650.t01 | 2017 | glutaredoxin, putative, expressed | 356 | 11.918691 | 63 | 2.8396514 | 2.06944 | 1.04E-34 | 9.46E-36 | up |
| VIT_14s0066g00570.t01 | 102 | Putative uncharacterized protein | 51 | 33.764043 | 9 | 8.0218163 | 2.0734868 | 6.32E-06 | 2.25E-06 | up |
| VIT_01s0010g00760.t01 | 2135 | Putative uncharacterized protein | 812 | 25.682813 | 143 | 6.0893163 | 2.0764511 | 3.21E-78 | 1.36E-79 | up |
| VIT_08s0056g00780.t01 | 1673 | MATE efflux family protein | 125 | 5.0454338 | 22 | 1.1955207 | 2.0773392 | 6.16E-13 | 1.23E-13 | up |
| VIT_08s0007g06040.t01 | 1250 | Beta-1,3-glucanase | 1712 | 92.486467 | 301 | 21.892071 | 2.0788338 | 3.38E-164 | 6.84E-166 | up |
| VIT_08s0007g03230.t01 | 1220 | Putative uncharacterized protein | 239 | 13.228863 | 42 | 3.1298234 | 2.0795359 | 8.77E-24 | 1.08E-24 | up |
| VIT_06s0004g04650.t01 | 547 | metallothionein-like protein 1 | 8261 | 1019.8346 | 1451 | 241.16288 | 2.0802554 | 0 | 0 | up |
| VIT_09s0002g06520.t01 | 733 | EG45-like domain containing protein | 114 | 10.502322 | 20 | 2.480598 | 2.0819484 | 6.26E-12 | 1.34E-12 | up |
| VIT_09s0002g02320.t01 | 1892 | cell wall apoplastic invertase | 1106 | 39.474664 | 194 | 9.3220402 | 2.0822093 | 1.36E-106 | 4.28E-108 | up |
| VIT_08s0007g07120.t01 | 1220 | F-box/kelch-repeat protein | 1597 | 88.395372 | 280 | 20.865489 | 2.0828521 | 1.35E-153 | 2.91E-155 | up |
| VIT_00s0194g00120.t01 | 1345 | serine acetyltransferase protein, putative, expressed | 902 | 45.286494 | 158 | 10.679851 | 2.0841894 | 3.33E-87 | 1.28E-88 | up |
| VIT_12s0035g01400.t01 | 617 | COX assembly mitochondrial protein 2 | 63 | 6.8950882 | 11 | 1.6208316 | 2.0888348 | 4.09E-07 | 1.28E-07 | up |
| VIT_07s0104g00350.t01 | 1285 | circadian clock coupling factor-related, putative, expressed | 768 | 40.359199 | 134 | 9.4805175 | 2.0898598 | 1.22E-74 | 5.45E-76 | up |
| VIT_14s0006g02290.t01 | 1189 | ethylene-responsive transcription factor ERF038-like | 167 | 9.4846009 | 29 | 2.2174126 | 2.0967098 | 4.11E-17 | 6.68E-18 | up |
| VIT_18s0001g11160.t01 | 850 | MIZU-KUSSEI 1 | 956 | 75.949236 | 166 | 17.754953 | 2.0968139 | 4.48E-93 | 1.61E-94 | up |
| VIT_15s0046g01560.t01 | 4073 | BAG family molecular chaperone regulator 6 | 4223 | 70.015003 | 732 | 16.339059 | 2.0993392 | 0 | 0 | up |
| VIT_16s0098g01230.t01 | 738 | cytochrome b5 isoform E | 7291 | 667.13723 | 1261 | 155.34207 | 2.1025349 | 0 | 0 | up |
| VIT_06s0004g01470.t01 | 2768 | Lipoxygenase (LOX) | 586 | 14.296047 | 101 | 3.317307 | 2.1075319 | 8.76E-58 | 4.98E-59 | up |
| VIT_08s0040g02900.t01 | 893 | ALUMINUM SENSITIVE 3 | 958 | 72.443345 | 165 | 16.798204 | 2.1085461 | 6.34E-94 | 2.25E-95 | up |
| VIT_06s0004g05960.t01 | 821 | Putative uncharacterized protein | 5275 | 433.87412 | 908 | 100.54791 | 2.1093934 | 0 | 0 | up |
| VIT_06s0009g03540.t01 | 590 | RING-H2 finger protein ATL34-like | 308 | 35.25195 | 53 | 8.1668434 | 2.1098526 | 6.77E-31 | 6.78E-32 | up |
| VIT_09s0002g03460.t01 | 1319 | clavaminate synthase-like protein | 186 | 9.5225353 | 32 | 2.2056447 | 2.1101453 | 4.42E-19 | 6.58E-20 | up |
| VIT_14s0006g02550.t01 | 556 | non-specific lipid-transfer protein | 2123 | 257.84555 | 365 | 59.682698 | 2.1111225 | 1.90E-207 | 2.99E-209 | up |
| VIT_13s0156g00260.t01 | 1403 | homeobox-leucine zipper protein HAT14-like | 192 | 9.2411922 | 33 | 2.1383887 | 2.1115549 | 1.12E-19 | 1.62E-20 | up |
| VIT_19s0090g00080.t01 | 907 | fatty acid desaturase 4 | 175 | 13.029124 | 30 | 3.0070756 | 2.115307 | 4.54E-18 | 7.06E-19 | up |
| VIT_13s0019g02130.t01 | 807 | tropinone reductase | 123 | 10.292385 | 21 | 2.3657897 | 2.1211836 | 4.62E-13 | 9.15E-14 | up |
| VIT_17s0000g01210.t01 | 636 | Putative uncharacterized protein | 211 | 22.403186 | 36 | 5.1460708 | 2.1221607 | 1.20E-21 | 1.61E-22 | up |
| VIT_10s0071g00850.t01 | 1182 | Putative uncharacterized protein | 364 | 20.795451 | 62 | 4.7687503 | 2.1245848 | 1.22E-36 | 1.06E-37 | up |
| VIT_14s0083g00580.t01 | 1627 | S-adenosyl-L-methionine decarboxylase (SAMDC) | 3582 | 148.6697 | 610 | 34.085734 | 2.1248707 | 0 | 0 | up |
| VIT_07s0005g00340.t01 | 1248 | phytochrome A-associated F-box protein-like | 1693 | 91.606611 | 288 | 20.980135 | 2.1264278 | 4.27E-167 | 8.38E-169 | up |
| VIT_05s0062g00740.t01 | 1627 | UDP-glycosyltransferase 75C1-like | 341 | 14.15309 | 58 | 3.2409387 | 2.1266334 | 1.98E-34 | 1.81E-35 | up |
| VIT_07s0005g03230.t01 | 1040 | ethylene-responsive transcription factor 1B | 147 | 9.5448353 | 25 | 2.1854307 | 2.1268027 | 1.89E-15 | 3.32E-16 | up |
| VIT_13s0073g00690.t01 | 1360 | DNA binding protein, putative, expressed | 112 | 5.5611365 | 19 | 1.2701209 | 2.1304139 | 4.70E-12 | 9.95E-13 | up |
| VIT_00s0805g00010.t01 | 274 | calmodulin-binding transcription activator 2-like | 130 | 32.038873 | 22 | 7.2996576 | 2.1339227 | 7.66E-14 | 1.46E-14 | up |
| VIT_07s0005g02770.t01 | 451 | Putative uncharacterized protein | 130 | 19.464859 | 22 | 4.4348252 | 2.1339227 | 7.66E-14 | 1.46E-14 | up |
| VIT_10s0003g00230.t01 | 654 | Putative uncharacterized protein | 136 | 14.042538 | 23 | 3.1972784 | 2.1348874 | 1.93E-14 | 3.59E-15 | up |
| VIT_03s0063g00550.t01 | 1063 | cell number regulator 1 | 2525 | 160.40303 | 427 | 36.519513 | 2.1349619 | 4.17E-250 | 5.36E-252 | up |
| VIT_08s0007g06900.t01 | 746 | dirigent protein 2-like | 71 | 6.4269358 | 12 | 1.4624223 | 2.1357711 | 4.53E-08 | 1.29E-08 | up |
| VIT_09s0002g07360.t01 | 1318 | hus1-like protein, expressed | 225 | 11.527936 | 38 | 2.6211903 | 2.1368402 | 3.32E-23 | 4.21E-24 | up |
| VIT_04s0008g01510.t01 | 976 | 17.3 kDa class II heat shock protein | 243 | 16.812833 | 41 | 3.8191297 | 2.138247 | 5.38E-25 | 6.40E-26 | up |
| VIT_01s0146g00100.t01 | 1709 | ACT domain containing protein, expressed | 605 | 23.905496 | 102 | 5.4261086 | 2.1393525 | 9.59E-61 | 5.17E-62 | up |
| VIT_01s0010g02940.t01 | 530 | OsCML31 - Calmodulin-related calcium sensor protein, expressed | 113 | 14.397498 | 19 | 3.2591782 | 2.143238 | 3.10E-12 | 6.48E-13 | up |
| VIT_03s0038g02770.t01 | 767 | sorcin | 639 | 56.258732 | 107 | 12.682906 | 2.1491916 | 1.80E-64 | 9.20E-66 | up |
| VIT_04s0069g01180.t01 | 1263 | NC domain-containing protein, putative, expressed | 2992 | 159.97152 | 501 | 36.06324 | 2.1492142 | 9.55E-299 | 1.07E-300 | up |
| VIT_16s0050g00140.t01 | 616 | Putative uncharacterized protein | 54 | 5.9196699 | 9 | 1.3282878 | 2.155949 | 1.81E-06 | 6.07E-07 | up |
| VIT_08s0007g07390.t01 | 490 | Putative uncharacterized protein | 126 | 17.364365 | 21 | 3.8963108 | 2.155949 | 1.31E-13 | 2.53E-14 | up |
| VIT_03s0038g02490.t01 | 717 | SKP1-like protein 1A-like | 297 | 27.971885 | 49 | 6.213085 | 2.1705958 | 7.09E-31 | 7.10E-32 | up |
| VIT_11s0103g00520.t01 | 578 | Putative uncharacterized protein | 291 | 33.997704 | 48 | 7.5499447 | 2.1708994 | 2.80E-30 | 2.86E-31 | up |
| VIT_14s0030g01220.t01 | 450 | Putative uncharacterized protein | 7788 | 1168.6861 | 1281 | 258.80162 | 2.1749689 | 0 | 0 | up |
| VIT_18s0001g12430.t01 | 1302 | Putative uncharacterized protein | 438 | 22.716822 | 72 | 5.0274977 | 2.1758486 | 3.48E-45 | 2.52E-46 | up |
| VIT_04s0023g02100.t01 | 485 | Putative uncharacterized protein | 146 | 20.328043 | 24 | 4.498833 | 2.1758486 | 9.14E-16 | 1.58E-16 | up |
| VIT_17s0000g04470.t01 | 1127 | precursor microRNA MIR162_1H-10 gene | 128 | 7.6695608 | 21 | 1.6940482 | 2.1786691 | 5.62E-14 | 1.06E-14 | up |
| VIT_10s0116g00500.t01 | 601 | transcription factor CPC-like | 256 | 28.764043 | 42 | 6.3533853 | 2.1786691 | 7.09E-27 | 7.95E-28 | up |
| VIT_03s0038g00130.t01 | 799 | Putative uncharacterized protein | 470 | 39.722404 | 77 | 8.7614163 | 2.1807169 | 1.53E-48 | 1.03E-49 | up |
| VIT_18s0001g10180.t01 | 1570 | acetyltransferase | 496 | 21.333714 | 81 | 4.6904633 | 2.1853328 | 2.65E-51 | 1.70E-52 | up |
| VIT_07s0031g00340.t01 | 958 | F-box protein | 509 | 35.878701 | 83 | 7.8766755 | 2.1874689 | 1.11E-52 | 6.88E-54 | up |
| VIT_10s0003g05690.t01 | 945 | LIGHT-DEPENDENT SHORT HYPOCOTYLS 4 | 393 | 28.083109 | 64 | 6.157133 | 2.189372 | 5.98E-41 | 4.69E-42 | up |
| VIT_12s0028g00930.t01 | 871 | glutathione S-transferase | 1339 | 103.81183 | 218 | 22.754574 | 2.1897424 | 3.04E-137 | 7.26E-139 | up |
| VIT_05s0020g01170.t01 | 769 | translationally-controlled tumor protein homolog-like | 45419 | 3988.3721 | 7393 | 874.02678 | 2.1900506 | 0 | 0 | up |
| VIT_14s0030g01230.t01 | 434 | Putative uncharacterized protein | 13917 | 2165.411 | 2259 | 473.21323 | 2.1940786 | 0 | 0 | up |
| VIT_08s0007g01560.t01 | 864 | Putative uncharacterized protein | 1990 | 155.53344 | 323 | 33.987495 | 2.1941489 | 4.26E-204 | 6.77E-206 | up |
| VIT_10s0003g01650.t01 | 685 | Putative uncharacterized protein | 531 | 52.34659 | 86 | 11.41401 | 2.1972898 | 3.24E-55 | 1.92E-56 | up |
| VIT_05s0020g02770.t01 | 1073 | aluminum induced protein with YGL and LRDR motif-like | 8632 | 543.24552 | 1396 | 118.28129 | 2.1993824 | 0 | 0 | up |
| VIT_14s0066g01610.t01 | 1739 | RING-H2 finger protein ATL46-like | 334 | 12.969742 | 54 | 2.8230889 | 2.1998033 | 3.70E-35 | 3.34E-36 | up |
| VIT_13s0067g01020.t01 | 1123 | flavonol synthase/flavanone 3-hydroxylase | 93 | 5.5922636 | 15 | 1.2143444 | 2.2032547 | 1.37E-10 | 3.22E-11 | up |
| VIT_03s0017g01990.t01 | 1621 | UDP-glucoronosyl and UDP-glucosyl transferase domain containing protein, express | 155 | 6.4570348 | 25 | 1.402127 | 2.2032547 | 6.48E-17 | 1.06E-17 | up |
| VIT_11s0016g00110.t01 | 1901 | B4-BTB3 - Bric-a-Brac, Tramtrack, Broad Complex BTB domain with B4 subfamil | 186 | 6.6071668 | 30 | 1.4347278 | 2.2032547 | 4.40E-20 | 6.30E-21 | up |
| VIT_10s0116g01760.t01 | 345 | transcription repressor MYB4-like | 149 | 29.164304 | 24 | 6.3244464 | 2.2051925 | 2.56E-16 | 4.31E-17 | up |
| VIT_18s0001g02690.t01 | 451 | Putative uncharacterized protein | 143 | 21.411344 | 23 | 4.6364082 | 2.2072959 | 1.01E-15 | 1.75E-16 | up |
| VIT_02s0012g00530.t01 | 1549 | ribose-phosphate pyrophosphokinase 1 | 287 | 12.51166 | 46 | 2.6998323 | 2.2123315 | 1.47E-30 | 1.48E-31 | up |
| VIT_05s0049g00400.t01 | 1294 | 1-aminocyclopropane-1-carboxylate oxidase | 362 | 18.891165 | 58 | 4.0749669 | 2.2128514 | 2.73E-38 | 2.28E-39 | up |
| VIT_01s0026g02620.t01 | 1130 | expansin-A1-like | 8152 | 487.15837 | 1305 | 104.99351 | 2.2140908 | 0 | 0 | up |
| VIT_03s0038g01510.t01 | 580 | ERD protein (ERD) | 11736 | 1366.3959 | 1878 | 294.373 | 2.2146583 | 0 | 0 | up |
| VIT_19s0014g01530.t01 | 1668 | probable leucine-rich repeat receptor-like serine/threonine-protein kinase | 138 | 5.5868561 | 22 | 1.1991044 | 2.2200793 | 2.59E-15 | 4.60E-16 | up |
| VIT_02s0025g04200.t01 | 830 | bifunctional UDP-glucose 4-epimerase and UDP-xylose 4-epimerase 1 | 377 | 30.672396 | 60 | 6.5720904 | 2.2225166 | 4.81E-40 | 3.87E-41 | up |
| VIT_13s0067g02130.t01 | 674 | ERD protein (ERD) | 3541 | 354.77293 | 563 | 75.941448 | 2.2239365 | 0 | 0 | up |
| VIT_04s0210g00060.t01 | 942 | adipocyte plasma membrane-associated protein-like | 195 | 13.978744 | 31 | 2.9918593 | 2.2241205 | 3.11E-21 | 4.24E-22 | up |
| VIT_13s0019g00860.t01 | 809 | 15.7 kDa heat shock protein, peroxisomal-like | 107 | 8.9314033 | 17 | 1.9104284 | 2.2249907 | 3.80E-12 | 7.99E-13 | up |
| VIT_17s0000g00200.t01 | 1218 | ethylene-responsive transcription factor ERF114 | 214 | 11.864541 | 34 | 2.5378269 | 2.2249907 | 3.29E-23 | 4.17E-24 | up |
| VIT_16s0022g02440.t01 | 678 | probable carboxylesterase 120-like | 82 | 8.1671137 | 13 | 1.7431872 | 2.2280988 | 1.38E-09 | 3.51E-10 | up |
| VIT_04s0008g01570.t01 | 552 | 17.3 kDa class II heat shock protein | 139 | 17.004355 | 22 | 3.6233808 | 2.230496 | 1.70E-15 | 2.97E-16 | up |

| | | | | | | | | | | |
|---|---|---|---|---|---|---|---|---|---|---|
| VIT_00s0187g00260.t01 | 1162 | wax synthase isoform 1, putative, expressed | 114 | 6.6249586 | 18 | 1.4083051 | 2.2339515 | 6.32E-13 | 1.26E-13 | up |
| VIT_00s0218g00170.t01 | 1137 | putative UDP-rhamnose:rhamnosyltransferase 1-like | 660 | 39.198362 | 104 | 8.3157849 | 2.236869 | 6.72E-70 | 3.22E-71 | up |
| VIT_09s0002g01060.t01 | 540 | Putative uncharacterized protein | 375 | 46.894504 | 59 | 9.9331873 | 2.2390902 | 3.43E-40 | 2.74E-41 | up |
| VIT_03s0097g00510.t01 | 2426 | oligopeptide transporter 4-like | 2290 | 63.742505 | 360 | 13.490936 | 2.2402653 | 7.95E-241 | 1.05E-242 | up |
| VIT_02s0241g00100.t01 | 857 | Putative uncharacterized protein | 350 | 27.578565 | 55 | 5.8346155 | 2.2408379 | 1.29E-37 | 1.10E-38 | up |
| VIT_11s0118g00730.t01 | 1406 | Putative uncharacterized protein | 4003 | 192.25813 | 629 | 40.672016 | 2.2409362 | 0 | 0 | up |
| VIT_01s0011g06600.t01 | 1514 | glutamate decarboxylase 1-like | 261 | 11.641236 | 41 | 2.4620017 | 2.2413405 | 2.57E-28 | 2.75E-29 | up |
| VIT_15s0021g01120.t01 | 416 | cytochrome c oxidase-assembly factor COX23 | 51 | 8.2786837 | 8 | 1.7483446 | 2.2434119 | 2.00E-06 | 6.73E-07 | up |
| VIT_14s0006g03110.t01 | 414 | acyl-CoA-binding domain-containing protein 3-like (ACBP) | 32 | 5.2195622 | 5 | 1.0979942 | 2.2490584 | 0.00018968 | 8.08E-05 | up |
| VIT_04s0023g00580.t01 | 491 | auxin-induced protein 15A | 775 | 106.5871 | 121 | 22.404448 | 2.2501758 | 1.68E-82 | 6.81E-84 | up |
| VIT_08s0058g00590.t01 | 593 | macrophage migration inhibitory factor homolog-like | 564 | 64.225701 | 88 | 13.491441 | 2.2511062 | 2.52E-60 | 1.37E-61 | up |
| VIT_19s0090g00120.t01 | 1136 | transmembrane protein 189-like | 509 | 30.256863 | 79 | 6.3223587 | 2.2587276 | 9.27E-55 | 5.54E-56 | up |
| VIT_02s0033g01380.t01 | 1185 | Alternative oxidase | 898 | 51.173183 | 139 | 10.664164 | 2.2626171 | 4.39E-96 | 1.52E-97 | up |
| VIT_03s0088g01180.t01 | 3084 | receptor-like protein kinase HAIKU2 | 822 | 17.998731 | 127 | 3.7438611 | 2.2652964 | 3.72E-88 | 1.41E-89 | up |
| VIT_12s0055g01280.t01 | 1844 | BRASSINOSTEROID INSENSITIVE 1-associated receptor kinase 1-like | 311 | 11.388956 | 48 | 2.3665228 | 2.2667948 | 5.36E-34 | 4.96E-35 | up |
| VIT_10s0003g00390.t01 | 722 | glutaredoxin | 16928 | 1583.2624 | 2611 | 328.77595 | 2.2677237 | 0 | 0 | up |
| VIT_18s0166g00250.t01 | 1192 | extensin | 16473 | 933.21323 | 2533 | 193.19207 | 2.2721708 | 0 | 0 | up |
| VIT_05s0020g03240.t01 | 2385 | G-type lectin S-receptor-like serine/threonine-protein kinase RLK1 | 893 | 25.284101 | 137 | 5.2223089 | 2.2754708 | 3.31E-96 | 1.14E-97 | up |
| VIT_08s0007g01350.t01 | 400 | Putative uncharacterized protein | 9318 | 1573.0668 | 1429 | 324.78997 | 2.2760009 | 0 | 0 | up |
| VIT_07s0005g02660.t01 | 4405 | ABC transporter B family member 11 | 626 | 9.5964999 | 96 | 1.9813249 | 2.2760429 | 9.69E-68 | 4.73E-69 | up |
| VIT_09s0018g00430.t01 | 638 | cytochrome c oxidase assembly factor 5 | 98 | 10.372653 | 15 | 2.1374746 | 2.2788058 | 1.61E-11 | 3.55E-12 | up |
| VIT_19s0093g00350.t01 | 1048 | glutathione S-transferase | 98 | 6.3146493 | 15 | 1.3012488 | 2.2788058 | 1.61E-11 | 3.55E-12 | up |
| VIT_03s0097g00110.t01 | 1539 | L-type lectin-domain containing receptor kinase IX.1-like | 229 | 10.048039 | 35 | 2.067568 | 2.2809073 | 1.73E-25 | 2.02E-26 | up |
| VIT_10s0003g01170.t01 | 745 | transcription factor bHLH106-like | 118 | 10.695724 | 18 | 2.1965779 | 2.2837046 | 1.12E-13 | 2.16E-14 | up |
| VIT_04s0023g02450.t01 | 1285 | cysteine proteinase 1 precursor, putative, expressed | 10449 | 549.10582 | 1593 | 112.70496 | 2.2845332 | 0 | 0 | up |
| VIT_01s0011g03850.t01 | 1801 | DNA binding protein, putative, expressed | 539 | 20.209683 | 82 | 4.1393344 | 2.287576 | 8.19E-59 | 4.59E-60 | up |
| VIT_02s0033g00800.t01 | 1201 | bifunctional nitrilase/nitrile hydratase | 803 | 45.14992 | 122 | 9.2352189 | 2.2895053 | 3.03E-87 | 1.16E-88 | up |
| VIT_00s0207g00110.t01 | 762 | ycf20-like protein | 237 | 21.00283 | 36 | 4.2951457 | 2.2898048 | 1.84E-26 | 2.09E-27 | up |
| VIT_03s0017g01450.t01 | 717 | cysteine-rich and transmembrane domain-containing protein A | 547 | 51.517243 | 83 | 10.524205 | 2.2913441 | 8.71E-60 | 4.78E-61 | up |
| VIT_16s0100g00740.t01 | 772 | Putative uncharacterized protein | 178 | 15.569947 | 27 | 3.1796318 | 2.2918324 | 3.67E-20 | 5.23E-21 | up |
| VIT_01s0026g02100.t01 | 772 | Putative uncharacterized protein | 581 | 50.821008 | 88 | 10.363244 | 2.2939492 | 1.59E-63 | 8.18E-65 | up |
| VIT_08s0058g01260.t01 | 429 | RING-H2 finger protein ATL74 | 172 | 27.074198 | 26 | 5.5099344 | 2.2968115 | 1.44E-19 | 2.11E-20 | up |
| VIT_09s0054g00910.t01 | 1383 | mitochondrial chaperone BCS1-like | 139 | 6.7869877 | 21 | 1.3804716 | 2.2976102 | 5.02E-16 | 8.57E-17 | up |
| VIT_00s0271g00110.t01 | 868 | flavoprotein wrbA-like | 146 | 11.358411 | 22 | 2.3042698 | 2.3013794 | 8.28E-17 | 1.36E-17 | up |
| VIT_03s0017g01040.t01 | 1606 | 7-deoxyloganetin glucosyltransferase | 146 | 6.138917 | 22 | 1.2453961 | 2.3013794 | 8.28E-17 | 1.36E-17 | up |
| VIT_05s0077g02350.t01 | 785 | Putative uncharacterized protein | 1953 | 168.003 | 294 | 34.049289 | 2.3027904 | 1.93E-212 | 2.91E-214 | up |
| VIT_04s0069g00920.t01 | 1417 | WRKY transcription factor 17 | 2662 | 126.8594 | 400 | 25.663773 | 2.3054252 | 9.53E-290 | 1.13E-291 | up |
| VIT_14s0171g00400.t01 | 1893 | Lysine histidine transporter 1-like | 1804 | 64.353232 | 271 | 13.015146 | 2.3058211 | 1.31E-196 | 2.20E-198 | up |
| VIT_11s0016g04720.t01 | 943 | EG45-like domain containing protein | 1019 | 72.970435 | 153 | 14.750614 | 2.306537 | 1.87E-111 | 5.64E-113 | up |
| VIT_06s0004g07280.t01 | 934 | UDP-glycosyltransferase 87A1-like | 480 | 34.703942 | 72 | 7.0083534 | 2.3079521 | 4.93E-53 | 3.03E-54 | up |
| VIT_14s0060g01720.t01 | 445 | Putative uncharacterized protein | 8975 | 1361.9429 | 1343 | 274.37616 | 2.3114391 | 0 | 0 | up |
| VIT_14s0083g00340.t01 | 2133 | endo-1,3(4)-beta-glucanase 2-like | 1739 | 55.054544 | 260 | 11.081865 | 2.3126609 | 3.13E-190 | 5.52E-192 | up |
| VIT_08s0007g06220.t01 | 565 | Putative uncharacterized protein | 67 | 8.0077554 | 10 | 1.6090959 | 2.3151476 | 2.31E-08 | 6.44E-09 | up |
| VIT_12s0057g01440.t01 | 1252 | Putative uncharacterized protein | 188 | 10.14 | 28 | 2.0332186 | 2.3182204 | 1.64E-21 | 2.21E-22 | up |
| VIT_18s0001g06400.t01 | 771 | Putative uncharacterized protein | 148 | 12.96259 | 22 | 2.5941714 | 2.3210083 | 3.45E-17 | 5.59E-18 | up |
| VIT_16s0050g01530.t01 | 1731 | leucine rich repeat protein, putative, expressed | 222 | 8.6604478 | 33 | 1.7331943 | 2.3210083 | 3.04E-25 | 3.58E-26 | up |
| VIT_05s0049g00930.t01 | 1813 | inorganic phosphate transporter 1-4-lik | 439 | 16.351258 | 65 | 3.259462 | 2.3266958 | 4.68E-49 | 3.13E-50 | up |
| VIT_09s0002g04560.t01 | 1154 | calmodulin-binding protein, putative, expressed | 245 | 14.336552 | 36 | 2.8361361 | 2.3376994 | 5.61E-28 | 6.05E-29 | up |
| VIT_14s0006g01610.t01 | 747 | Putative uncharacterized protein | 143 | 12.927063 | 21 | 2.5558129 | 2.3385404 | 8.77E-17 | 1.44E-17 | up |
| VIT_14s0083g01200.t01 | 973 | Putative uncharacterized protein | 178 | 12.353545 | 26 | 2.4293544 | 2.3462802 | 1.05E-20 | 1.46E-21 | up |
| VIT_04s0008g05410.t01 | 1004 | carboxylesterase 17 | 666 | 44.794527 | 97 | 8.7835159 | 2.350452 | 8.41E-75 | 3.74E-76 | up |
| VIT_19s0090g00600.t01 | 555 | Putative uncharacterized protein | 158 | 19.224212 | 23 | 3.7676038 | 2.3512053 | 1.52E-18 | 2.32E-19 | up |
| VIT_05s0077g01850.t01 | 895 | glycine-rich protein, putative, expressed | 654 | 49.344546 | 95 | 9.6500806 | 2.3542777 | 1.30E-73 | 5.89E-75 | up |
| VIT_04s0044g01430.t01 | 1129 | Putative uncharacterized protein | 2685 | 160.59602 | 390 | 31.405162 | 2.3543626 | 1.25E-299 | 1.39E-301 | up |
| VIT_15s0046g00490.t01 | 1733 | O-acyltransferase WSD1-like | 290 | 11.300141 | 42 | 2.2033379 | 2.3585782 | 2.87E-33 | 2.70E-34 | up |
| VIT_08s0007g00120.t01 | 877 | ubiquitin-conjugating enzyme, putative, expressed | 7644 | 588.58004 | 1104 | 114.4458 | 2.3625741 | 0 | 0 | up |
| VIT_03s0063g01970.t01 | 937 | plastid division protein PDV2-like | 237 | 17.08021 | 34 | 3.2989042 | 2.3722669 | 1.48E-27 | 1.62E-28 | up |
| VIT_00s0218g00190.t01 | 1056 | glucosyltransferase, putative, expressed | 495 | 31.653791 | 71 | 6.1125835 | 2.3725241 | 1.88E-56 | 1.09E-57 | up |
| VIT_17s0000g09790.t01 | 1456 | BTB/POZ and TAZ domain-containing protein 1-like | 4260 | 197.57531 | 606 | 37.839172 | 2.3844502 | 0 | 0 | up |
| VIT_15s0046g02880.t01 | 517 | peptidyl-prolyl cis-trans isomerase, putative, expressed | 402 | 52.507332 | 57 | 10.023391 | 2.3891482 | 2.08E-46 | 1.47E-47 | up |
| VIT_05s0049g01550.t01 | 2214 | peptide transporter PTR3-A-like | 304 | 9.2721492 | 43 | 1.7657175 | 2.3926493 | 2.11E-35 | 1.89E-36 | up |
| VIT_14s0060g02320.t01 | 3225 | respiratory burst oxidase | 269 | 5.6325753 | 38 | 1.0712338 | 2.3945214 | 1.80E-31 | 1.78E-32 | up |
| VIT_05s0020g04680.t01 | 634 | auxin-induced protein 22D | 78 | 8.3078718 | 11 | 1.5773708 | 2.3969571 | 6.54E-10 | 1.62E-10 | up |
| VIT_16s0013g00480.t01 | 776 | Putative uncharacterized protein | 641 | 55.780288 | 90 | 10.54414 | 2.403314 | 6.50E-74 | 2.92E-75 | up |
| VIT_11s0016g00340.t01 | 1161 | DNA repair protein RAD51 homolog | 164 | 9.5388511 | 23 | 1.8010509 | 2.4049766 | 1.07E-19 | 1.56E-20 | up |
| VIT_07s0031g00500.t01 | 2365 | subtilisin-like protease | 214 | 6.1103638 | 30 | 1.1532421 | 2.4055629 | 2.23E-25 | 2.62E-26 | up |
| VIT_14s0219g00110.t01 | 889 | Putative uncharacterized protein | 264 | 20.053335 | 37 | 3.7838188 | 2.4059273 | 4.51E-31 | 4.50E-32 | up |
| VIT_03s0038g00200.t01 | 463 | serine/threonine-protein phosphatase 2A 65 kDa regulatory subunit A beta isoform | 50 | 7.2924499 | 7 | 1.3745085 | 2.4074878 | 8.87E-07 | 2.87E-07 | up |
| VIT_14s0068g01790.t01 | 597 | Putative uncharacterized protein | 50 | 5.6556186 | 7 | 1.0659923 | 2.4074878 | 8.87E-07 | 2.87E-07 | up |
| VIT_05s0049g00740.t01 | 719 | proline-rich cell wall protein 2-like | 620 | 58.230061 | 86 | 10.874266 | 2.4208462 | 3.97E-72 | 1.85E-73 | up |
| VIT_14s0006g01250.t01 | 787 | ADP-ribosylation factor GTPase-activating protein AGD13 | 832 | 71.389286 | 115 | 13.284753 | 2.4259362 | 1.06E-96 | 3.66E-98 | up |
| VIT_09s0002g04160.t01 | 1045 | acyl-coenzyme A thioesterase 13-like | 145 | 9.3699259 | 20 | 1.7399793 | 2.4289675 | 9.94E-18 | 1.57E-18 | up |
| VIT_16s0098g01210.t01 | 378 | small G protein signaling modulator 2-like | 58 | 10.361452 | 8 | 1.9241041 | 2.4289675 | 9.40E-08 | 2.76E-08 | up |
| VIT_15s0045g00370.t01 | 678 | non-specific phospholipase C4-like | 109 | 10.856285 | 15 | 2.0113699 | 2.4322802 | 1.29E-13 | 2.49E-14 | up |
| VIT_00s0233g00010.t01 | 863 | acid beta-fructofuranosidase-like | 160 | 12.519692 | 22 | 2.3176201 | 2.433483 | 1.72E-19 | 2.52E-20 | up |
| VIT_04s0044g00220.t01 | 1639 | 3-hydroxybenzoate 6-hydroxylase 1-like | 342 | 14.090668 | 47 | 2.6070495 | 2.4342502 | 1.76E-40 | 1.40E-41 | up |
| VIT_10s0003g03160.t01 | 666 | 60S ribosomal protein L18a-like | 131 | 13.282552 | 18 | 2.4571329 | 2.4344845 | 3.68E-16 | 6.23E-17 | up |
| VIT_02s0025g00730.t01 | 599 | disease resistance response protein 206-like | 51 | 5.7494698 | 7 | 1.0624331 | 2.4360569 | 5.68E-07 | 1.80E-07 | up |
| VIT_01s0011g02750.t01 | 844 | nudix hydrolase 1 | 124 | 9.9211881 | 17 | 1.8312045 | 2.43772 | 2.23E-15 | 3.93E-16 | up |
| VIT_06s0009g01110.t01 | 1887 | cation/calcium exchanger 3-like | 526 | 18.823409 | 72 | 3.4688935 | 2.4399805 | 6.97E-62 | 3.70E-63 | up |
| VIT_09s0002g05820.t01 | 609 | Putative uncharacterized proteinn | 441 | 48.899649 | 60 | 8.9570362 | 2.4487308 | 2.95E-52 | 1.85E-53 | up |
| VIT_07s0129g00360.t01 | 1447 | Peroxidase 73 | 471 | 21.980462 | 64 | 4.0210717 | 2.4505698 | 8.67E-56 | 5.09E-57 | up |
| VIT_00s0179g00260.t01 | 3225 | calcium-transporting ATPase 12 | 413 | 8.6477829 | 56 | 1.5786603 | 2.4536296 | 4.09E-49 | 2.74E-50 | up |
| VIT_10s0071g00840.t01 | 1675 | Putative uncharacterized protein | 2715 | 109.45597 | 368 | 19.973923 | 2.454161 | 0 | 0 | up |
| VIT_09s0002g06890.t01 | 1234 | cucumber peeling cupredoxin-like | 384 | 21.013602 | 52 | 3.8310565 | 2.4555093 | 8.89E-46 | 6.37E-47 | up |
| VIT_07s0104g00830.t01 | 1769 | sugar transporter ERD6-like 7-like | 436 | 16.64344 | 59 | 3.032177 | 2.4565278 | 7.33E-52 | 4.64E-53 | up |
| VIT_01s0137g00670.t01 | 720 | sufE-like protein 2 | 303 | 28.41807 | 41 | 5.1770425 | 2.4566085 | 2.36E-36 | 2.07E-37 | up |
| VIT_06s0080g00660.t01 | 475 | Putative uncharacterized protein | 74 | 10.520165 | 10 | 1.9139772 | 2.4585118 | 1.04E-09 | 2.62E-10 | up |
| VIT_03s0038g00950.t01 | 683 | auxin-induced protein 15A-like | 259 | 25.607283 | 35 | 4.6588391 | 2.4585118 | 3.01E-31 | 2.98E-32 | up |
| VIT_19s0014g02120.t01 | 424 | Putative uncharacterized protein | 906 | 144.29351 | 122 | 26.159193 | 2.4636164 | 4.32E-107 | 1.34E-108 | up |
| VIT_05s0062g00980.t01 | 1162 | probable aldo-keto reductase 2 | 104 | 6.0438219 | 14 | 1.0953484 | 2.4640713 | 3.21E-13 | 6.31E-14 | up |
| VIT_04s0008g05770.t01 | 1763 | CBL-interacting serine/threonine-protein kinase 25-like | 336 | 12.869788 | 45 | 2.3205481 | 2.4714508 | 1.80E-40 | 1.43E-41 | up |
| VIT_07s0031g01270.t01 | 699 | E3 ubiquitin-protein ligase XERICO | 682 | 65.885772 | 91 | 11.835717 | 2.4768198 | 2.48E-81 | 1.02E-82 | up |
| VIT_03s0038g03450.t01 | 380 | auxin-induced protein 6B-like | 45 | 7.9967471 | 6 | 1.4354829 | 2.4778771 | 2.20E-06 | 7.41E-07 | up |
| VIT_01s0137g00790.t01 | 564 | Putative uncharacterized protein | 255 | 30.531316 | 34 | 5.4806262 | 2.4778771 | 4.73E-31 | 4.73E-32 | up |
| VIT_07s0130g00160.t01 | 924 | Putative uncharacterized protein | 609 | 44.507148 | 81 | 7.9697265 | 2.4814349 | 7.01E-73 | 3.23E-74 | up |
| VIT_00s0225g00050.t01 | 1773 | MATE efflux family protein | 585 | 22.280841 | 77 | 3.9483202 | 2.4964928 | 1.59E-70 | 7.59E-72 | up |

| | | | | | | | | | | |
|---|---|---|---|---|---|---|---|---|---|---|
| VIT_06s0061g01480.t01 | 4393 | pleiotropic drug resistance protein 2-like | 395 | 6.0718402 | 51 | 1.0554541 | 2.52427 | 1.58E-48 | 1.07E-49 | up |
| VIT_07s0104g01260.t01 | 1227 | indole-3-pyruvate monooxygenase | 1144 | 62.960172 | 147 | 10.891887 | 2.5311855 | 3.55E-139 | 8.35E-141 | up |
| VIT_01s0010g03040.t01 | 423 | probable calcium-binding protein CML31-like | 78 | 12.451988 | 10 | 2.1492652 | 2.5344606 | 1.72E-10 | 4.08E-11 | up |
| VIT_08s0040g00710.t01 | 676 | 3-5 exonuclease domain-containing protein, putative, expressed | 78 | 7.7917023 | 10 | 1.3448804 | 2.5344606 | 1.72E-10 | 4.08E-11 | up |
| VIT_14s0060g01730.t01 | 416 | Putative uncharacterized protein | 8084 | 1312.2525 | 1034 | 225.97354 | 2.5378196 | 0 | 0 | up |
| VIT_18s0001g05570.t01 | 1785 | transporter family protein, putative, expressed | 321 | 12.143706 | 41 | 2.0882188 | 2.539864 | 6.78E-40 | 5.49E-41 | up |
| VIT_00s0199g00290.t01 | 417 | glutaredoxin | 47 | 7.6110793 | 6 | 1.3081139 | 2.5406129 | 8.91E-07 | 2.88E-07 | up |
| VIT_16s0039g01110.t01 | 2649 | Phenylalanine ammonia-lyase | 346 | 8.8202031 | 44 | 1.5100839 | 2.5461831 | 4.88E-43 | 3.68E-44 | up |
| VIT_02s0154g00260.t01 | 2131 | nitrate transporter 1.1-like | 355 | 11.2494 | 45 | 1.9198152 | 2.5508086 | 3.21E-44 | 2.37E-45 | up |
| VIT_03s0091g00160.t01 | 825 | secretory protein, putative, expressed | 2612 | 213.79801 | 331 | 36.475766 | 2.5512383 | 0 | 0 | up |
| VIT_19s0090g00330.t01 | 1509 | 1-acyl-sn-glycerol-3-phosphate acyltransferase-like | 174 | 7.7865388 | 22 | 1.3254514 | 2.5544984 | 3.08E-22 | 4.05E-23 | up |
| VIT_05s0020g03690.t01 | 786 | acetyltransferase, GNAT family, putative, expressed | 1553 | 133.42381 | 196 | 22.670646 | 2.5571188 | 8.03E-191 | 1.41E-192 | up |
| VIT_16s0013g00440.t01 | 632 | Putative uncharacterized protein | 516 | 55.133691 | 65 | 9.3503238 | 2.559846 | 4.59E-64 | 2.36E-65 | up |
| VIT_03s0017g02170.t01 | 1338 | zinc transporter 5-like | 1567 | 79.085584 | 197 | 13.385681 | 2.5627242 | 5.30E-193 | 9.21E-195 | up |
| VIT_00s0510g00050.t01 | 1171 | aspartic proteinase-like protein 1-like | 350 | 20.183459 | 44 | 3.4160652 | 2.562766 | 7.80E-44 | 5.81E-45 | up |
| VIT_02s0025g01450.t01 | 687 | Putative uncharacterized protein | 295 | 28.996777 | 37 | 4.8963828 | 2.5661043 | 3.72E-37 | 3.19E-38 | up |
| VIT_12s0055g00800.t01 | 713 | 14 kDa proline-rich protein DC2.15-like | 112 | 10.607497 | 14 | 1.785126 | 2.5709865 | 8.46E-15 | 1.54E-15 | up |
| VIT_01s0127g00110.t01 | 177 | Putative uncharacterized protein | 24 | 9.1563507 | 3 | 1.5409139 | 2.5709865 | 0.00049363 | 0.00022344 | up |
| VIT_13s0073g00140.t01 | 910 | transcription repressor OFP13 | 248 | 18.403259 | 31 | 3.0970675 | 2.5709865 | 1.83E-31 | 1.81E-32 | up |
| VIT_08s0056g00990.t01 | 216 | TRANSPARENT TESTA 12-like | 24 | 7.5031207 | 3 | 1.2626933 | 2.5709865 | 0.00049363 | 0.00022344 | up |
| VIT_02s0025g03310.t01 | 1852 | putative transporter arsB-like | 209 | 7.6206102 | 26 | 1.2763293 | 2.5779059 | 9.37E-27 | 1.05E-27 | up |
| VIT_03s0038g00230.t01 | 1473 | purple acid phosphatase 2-like | 772 | 35.391502 | 96 | 5.9251433 | 2.578481 | 3.77E-96 | 1.30E-97 | up |
| VIT_17s0000g06710.t01 | 3453 | LRR receptor-like serine/threonine-protein kinase | 419 | 8.1941119 | 52 | 1.3691062 | 2.5813532 | 1.10E-52 | 6.84E-54 | up |
| VIT_00s0477g00030.t01 | 1348 | MATE efflux family protein | 404 | 20.238388 | 50 | 3.372178 | 2.5853418 | 6.38E-51 | 4.11E-52 | up |
| VIT_00s0415g00070.t01 | 575 | Putative uncharacterized protein | 1716 | 201.5273 | 212 | 33.519566 | 2.5878999 | 1.48E-213 | 2.22E-215 | up |
| VIT_01s0011g02620.t01 | 495 | Putative uncharacterized protein | 65 | 8.8673245 | 8 | 1.4693158 | 2.5933543 | 3.96E-09 | 1.04E-09 | up |
| VIT_03s0038g03190.t01 | 549 | flavin-containing monooxygenase family protein, putative, expressed | 65 | 7.9951286 | 8 | 1.324793 | 2.5933543 | 3.96E-09 | 1.04E-09 | up |
| VIT_15s0046g02410.t01 | 877 | aquaporin PIP1-2-like | 5387 | 414.79339 | 662 | 68.626013 | 2.5955655 | 0 | 0 | up |
| VIT_05s0049g01420.t01 | 745 | peroxisomal membrane protein PMP22-like | 245 | 22.207223 | 30 | 3.6609631 | 2.6007339 | 1.77E-31 | 1.74E-32 | up |
| VIT_09s0002g01750.t01 | 630 | Putative uncharacterized protein | 3192 | 342.1423 | 390 | 56.280044 | 2.6039011 | 0 | 0 | up |
| VIT_14s0006g01480.t01 | 305 | Putative uncharacterized protein | 41 | 9.077546 | 5 | 1.4903921 | 2.6066104 | 3.40E-06 | 1.17E-06 | up |
| VIT_18s0041g01010.t01 | 816 | UDP-glucoronosyl and UDP-glucosyl transferase domain containing protein, express | 74 | 6.1238706 | 9 | 1.002727 | 2.6105149 | 2.63E-10 | 6.33E-11 | up |
| VIT_16s0039g02380.t01 | 739 | leucine-rich repeat receptor-like protein kinase | 132 | 12.06185 | 16 | 1.9683663 | 2.6153806 | 1.49E-17 | 2.37E-18 | up |
| VIT_02s0025g00140.t01 | 714 | E3 ubiquitin-protein ligase RHA1B-like | 182 | 17.213042 | 22 | 2.8012692 | 2.6193495 | 7.89E-24 | 9.74E-25 | up |
| VIT_19s0014g03290.t01 | 1440 | NAC domain-containing protein 72-like | 407 | 19.086063 | 49 | 3.0935986 | 2.6251616 | 3.84E-52 | 2.42E-53 | up |
| VIT_04s0044g01760.t01 | 549 | Putative uncharacterized protein | 50 | 6.1500989 | 6 | 0.9935947 | 2.6298802 | 2.26E-07 | 6.89E-08 | up |
| VIT_01s0011g02880.t01 | 1940 | amino acid permease family protein, putative, expressed | 276 | 9.6070886 | 33 | 1.5464739 | 2.6351168 | 7.87E-36 | 6.98E-37 | up |
| VIT_06s0004g01000.t01 | 774 | dirigent protein 22 | 360 | 31.408412 | 43 | 5.0507732 | 2.6365749 | 1.88E-46 | 1.32E-47 | up |
| VIT_04s0023g02440.t01 | 2114 | Putative uncharacterized protein | 427 | 13.639779 | 51 | 2.1932875 | 2.6366534 | 6.57E-55 | 3.92E-56 | up |
| VIT_14s0006g00070.t01 | 675 | acyl-[acyl-carrier-protein] desaturase 6, | 67 | 6.7027878 | 8 | 1.0774983 | 2.6370757 | 1.57E-09 | 4.02E-10 | up |
| VIT_04s0043g01040.t01 | 386 | F-box protein PP2-B10 | 42 | 7.3476156 | 5 | 1.1776414 | 2.6413758 | 2.14E-06 | 7.21E-07 | up |
| VIT_11s0016g00500.t01 | 727 | Putative uncharacterized protein | 278 | 25.822294 | 33 | 4.1267665 | 2.6455335 | 3.11E-36 | 2.74E-37 | up |
| VIT_01s0011g06000.t01 | 940 | vacuolar iron transporter 1-like | 717 | 51.508126 | 85 | 8.2209394 | 2.6474249 | 5.85E-92 | 2.13E-93 | up |
| VIT_05s0020g02920.t01 | 1350 | Putative uncharacterized protein | 110 | 5.5022885 | 13 | 0.8754674 | 2.6519065 | 5.10E-15 | 9.21E-16 | up |
| VIT_19s0090g01070.t01 | 1706 | glucan endo-1,3-beta-glucosidase 13-like | 976 | 38.632715 | 115 | 6.1284294 | 2.6562338 | 2.65E-125 | 7.04E-127 | up |
| VIT_18s0001g13980.t01 | 448 | indole-3-acetic acid-induced protein ARG7-like | 119 | 17.937148 | 14 | 2.8410599 | 2.6584494 | 3.40E-16 | 5.74E-17 | up |
| VIT_01s0026g00220.t01 | 1659 | aldehyde dehydrogenase family 2 member B4 | 2281 | 92.846031 | 267 | 14.631715 | 2.6657413 | 5.83E-293 | 6.73E-295 | up |
| VIT_00s0586g00030.t01 | 981 | stem-specific protein TSJT1-lik | 428 | 29.461795 | 50 | 4.6337369 | 2.6685973 | 9.61E-56 | 5.66E-57 | up |
| VIT_10s0116g01860.t01 | 1812 | MATE efflux family protein | 677 | 25.229865 | 79 | 3.9636863 | 2.6702178 | 1.12E-87 | 4.27E-89 | up |
| VIT_06s0061g01210.t01 | 1530 | transmembrane amino acid transporter protein, putative, expressed | 738 | 32.572371 | 86 | 5.1101941 | 2.6721988 | 1.43E-95 | 5.00E-97 | up |
| VIT_11s0016g02200.t01 | 3104 | beta-galactosidase, putative, expressed | 249 | 5.4170404 | 29 | 0.849389 | 2.6730074 | 6.50E-33 | 6.19E-34 | up |
| VIT_04s0008g05380.t01 | 1405 | carboxylesterase 17 | 705 | 33.8842 | 82 | 5.306008 | 2.674914 | 1.95E-91 | 7.12E-93 | up |
| VIT_13s0067g00860.t01 | 776 | protein EARLY FLOWERING 4-lik | 1998 | 173.86742 | 232 | 27.18045 | 2.6773464 | 7.08E-258 | 8.93E-260 | up |
| VIT_01s0011g02420.t01 | 705 | E3 ubiquitin-protein ligase RHA2B | 138 | 13.218264 | 16 | 2.0632946 | 2.679511 | 9.30E-19 | 1.40E-19 | up |
| VIT_01s0011g01110.t01 | 1149 | MOSC domain-containing protein 1 | 1245 | 73.17012 | 144 | 11.393911 | 2.6829915 | 2.47E-161 | 5.09E-163 | up |
| VIT_08s0058g00980.t01 | 1045 | peroxidase 1 | 408 | 26.365033 | 47 | 4.0889513 | 2.688823 | 1.31E-53 | 8.00E-55 | up |
| VIT_12s0057g00420.t01 | 1588 | membrane protein, putative, expressed | 3586 | 152.49101 | 411 | 23.529987 | 2.6961517 | 0 | 0 | up |
| VIT_19s0014g02170.t01 | 1356 | aminotransferase | 515 | 25.646729 | 59 | 3.9556941 | 2.6967721 | 1.27E-67 | 6.22E-69 | up |
| VIT_11s0016g05380.t01 | 613 | Putative uncharacterized protein | 1078 | 118.75249 | 123 | 18.242107 | 2.7026135 | 7.03E-141 | 1.64E-142 | up |
| VIT_02s0025g01090.t01 | 559 | Putative uncharacterized protein | 729 | 88.064356 | 83 | 13.498846 | 2.7057221 | 1.14E-95 | 3.98E-97 | up |
| VIT_03s0038g03400.t01 | 1105 | Basic endochitinase | 4621 | 282.39574 | 525 | 43.194395 | 2.7088023 | 0 | 0 | up |
| VIT_13s0084g00240.t01 | 1134 | flavonol sulfotransferase-like | 97 | 5.776212 | 11 | 0.881881 | 2.7114677 | 1.15E-13 | 2.22E-14 | up |
| VIT_15s0045g01370.t01 | 599 | RING-H2 finger protein ATL64-like | 574 | 64.709719 | 65 | 9.8654502 | 2.7135256 | 9.50E-76 | 4.14E-77 | up |
| VIT_18s0001g01300.t01 | 547 | Putative uncharacterized protein | 53 | 6.5429407 | 6 | 0.9972276 | 2.7139445 | 5.63E-08 | 1.62E-08 | up |
| VIT_14s0060g01800.t01 | 525 | Putative uncharacterized protein | 62 | 7.9747454 | 7 | 1.2121856 | 2.7178279 | 3.71E-09 | 9.74E-10 | up |
| VIT_13s0019g02880.t01 | 1025 | UDP-glycosyltransferase 76C4-like | 133 | 8.762181 | 15 | 1.3304476 | 2.7193783 | 2.17E-18 | 3.34E-19 | up |
| VIT_14s0068g01040.t01 | 1238 | ethylene-responsive transcription factor ERF113-like | 204 | 11.127407 | 23 | 1.6890308 | 2.7198499 | 1.23E-27 | 1.34E-28 | up |
| VIT_06s0080g00790.t01 | 829 | myb-like protein H | 71 | 5.783467 | 8 | 0.8773358 | 2.7207336 | 2.46E-10 | 5.91E-11 | up |
| VIT_04s0008g04810.t01 | 1509 | Putative uncharacterized protein | 391 | 17.497337 | 44 | 2.6509028 | 2.7225797 | 4.31E-52 | 2.72E-53 | up |
| VIT_01s0137g00680.t01 | 460 | sufE-like protein 2 | 98 | 14.386418 | 11 | 2.1740285 | 2.7262647 | 7.21E-14 | 1.37E-14 | up |
| VIT_01s0011g05930.t01 | 1276 | S-adenosylmethionine-dependent methyltransferase | 196 | 10.372653 | 22 | 1.5674813 | 2.7262647 | 1.17E-26 | 1.32E-27 | up |
| VIT_10s0003g01690.t01 | 1802 | GIL1, putative, expressed | 215 | 8.0569027 | 24 | 1.2108402 | 2.7342169 | 3.15E-29 | 3.29E-30 | up |
| VIT_05s0049g01020.t01 | 1212 | myb-related protein Myb4-like | 117 | 6.5188004 | 13 | 0.9751493 | 2.7409115 | 1.96E-16 | 3.29E-17 | up |
| VIT_04s0069g01010.t01 | 1019 | harpin-induced protein 1 domain containing protein, expressed | 289 | 19.151734 | 32 | 2.8550004 | 2.7459122 | 4.15E-39 | 3.41E-40 | up |
| VIT_16s0013g00390.t01 | 716 | Putative uncharacterized protein | 1078 | 101.66938 | 119 | 15.109995 | 2.7503102 | 1.72E-143 | 3.96E-145 | up |
| VIT_03s0091g00810.t01 | 323 | Putative uncharacterized protein | 82 | 17.143353 | 9 | 2.5332051 | 2.7586135 | 6.44E-12 | 1.38E-12 | up |
| VIT_08s0058g01270.t01 | 1007 | RING-H2 finger protein, putative, expressed | 557 | 37.351682 | 61 | 5.5071986 | 2.7617827 | 6.83E-75 | 3.02E-76 | up |
| VIT_11s0052g00540.t01 | 984 | ABC transporter I family member 17 | 1014 | 69.58687 | 111 | 10.255533 | 2.7624126 | 1.24E-135 | 2.99E-137 | up |
| VIT_00s0218g00200.t01 | 432 | UDP-rhamnose:rhamnosyltransferase 1-like | 110 | 17.194652 | 12 | 2.5253866 | 2.7673837 | 1.16E-15 | 2.02E-16 | up |
| VIT_19s0093g00110.t01 | 674 | glutathione S-transferase | 709 | 71.034738 | 77 | 10.386308 | 2.7738418 | 1.65E-95 | 5.77E-97 | up |
| VIT_18s0001g11580.t01 | 1857 | cytochrome P450 82A3-like | 1558 | 56.655228 | 169 | 8.2738029 | 2.7755866 | 5.18E-209 | 8.08E-211 | up |
| VIT_11s0052g00850.t01 | 1245 | replication protein A 32 kDa subunit-like | 120 | 6.5087312 | 13 | 0.949302 | 2.7774374 | 4.78E-17 | 7.80E-18 | up |
| VIT_10s0003g02530.t01 | 424 | Putative uncharacterized protein | 65 | 10.352183 | 7 | 1.5009373 | 2.7859994 | 9.07E-10 | 2.28E-10 | up |
| VIT_14s0219g00230.t01 | 1227 | pectate lyase (PEL) | 409 | 22.509362 | 44 | 3.2601568 | 2.7875119 | 8.95E-56 | 5.26E-57 | up |
| VIT_06s0004g05120.t01 | 1778 | MYB family transcription factor, putative, expressed | 680 | 25.826265 | 73 | 3.7326862 | 2.7905529 | 3.32E-92 | 1.20E-93 | up |
| VIT_00s0317g00140.t01 | 2002 | exo70 exocyst complex subunit, putative, expressed | 199 | 6.7123323 | 21 | 0.9536425 | 2.8152937 | 6.21E-28 | 6.72E-29 | up |
| VIT_03s0038g03530.t01 | 1947 | lysine histidine transporter-like 8-like | 1716 | 59.51628 | 181 | 8.451679 | 2.8159745 | 1.44E-233 | 1.96E-235 | up |
| VIT_00s0199g00040.t01 | 255 | mitochondrial 2-oxoglutarate/malate carrier protein-like | 38 | 10.063009 | 4 | 1.4261007 | 2.818914 | 3.13E-06 | 1.07E-06 | up |
| VIT_18s0001g00610.t01 | 1003 | ganglioside-induced differentiation-associated protein 2-like | 352 | 23.69879 | 37 | 3.3537537 | 2.8209648 | 1.00E-48 | 6.74E-50 | up |
| VIT_11s0016g03580.t01 | 1112 | bark storage protein A-like | 990 | 60.119429 | 104 | 8.5027405 | 2.8218315 | 2.29E-135 | 5.54E-137 | up |
| VIT_09s0002g05080.t01 | 1382 | OsFBK4 - F-box domain and kelch repeat containing protein, expressed | 238 | 11.629294 | 25 | 1.6446078 | 2.8219481 | 2.76E-33 | 2.60E-34 | up |
| VIT_16s0022g00960.t01 | 666 | invertase/pectin methylesterase inhibitor family protein, putative, expressed | 34475 | 3495.5417 | 3614 | 493.33769 | 2.8248686 | 0 | 0 | up |
| VIT_02s0025g01430.t01 | 1554 | putative RING-H2 finger protein ATL21A-like | 860 | 37.370756 | 89 | 5.2067816 | 2.8434459 | 1.26E-118 | 3.59E-120 | up |
| VIT_13s0074g00110.t01 | 1219 | bark storage protein A-like | 1392 | 77.111646 | 144 | 10.739626 | 2.844005 | 1.54E-191 | 2.68E-193 | up |
| VIT_08s0007g02360.t01 | 918 | putative syntaxin-24-like | 6272 | 461.36836 | 644 | 63.778391 | 2.8547795 | 0 | 0 | up |
| VIT_16s0098g00850.t01 | 2461 | caffeic acid 3-O-methyltransferase | 3709 | 101.77232 | 380 | 14.037907 | 2.8579455 | 0 | 0 | up |
| VIT_12s0059g00440.t01 | 596 | keratin-associated protein 6-2 | 2426 | 274.87104 | 247 | 37.677412 | 2.8669831 | 0 | 0 | up |

| | | | | | | | | | | |
|---|---|---|---|---|---|---|---|---|---|---|
| VIT_01s0011g00990.t01 | 2385 | disease resistance protein RPM1-like | 187 | 5.294655 | 19 | 0.7242618 | 2.8699535 | 8.22E-27 | 9.24E-28 | up |
| VIT_13s0074g00700.t01 | 4565 | pleiotropic drug resistance protein 2-like | 345 | 5.103437 | 35 | 0.6970399 | 2.872156 | 1.23E-48 | 8.29E-50 | up |
| VIT_13s0067g02930.t01 | 1166 | expansin-like | 11247 | 651.36225 | 1138 | 88.730736 | 2.8759543 | 0 | 0 | up |
| VIT_05s0094g00350.t01 | 1284 | endochitinase A-like | 18682 | 982.52314 | 1881 | 133.18464 | 2.8830637 | 0 | 0 | up |
| VIT_14s0068g00730.t01 | 661 | Putative uncharacterized protein | 50 | 5.1080247 | 5 | 0.6876998 | 2.8929146 | 5.01E-08 | 1.43E-08 | up |
| VIT_12s0035g01910.t01 | 1183 | 22.0 kDa class IV heat shock protein-like | 461 | 26.314833 | 46 | 3.5351143 | 2.8960475 | 3.48E-65 | 1.77E-66 | up |
| VIT_03s0017g01400.t01 | 553 | cysteine-rich and transmembrane domain-containing protein A | 301 | 36.755794 | 30 | 4.9320389 | 2.8977156 | 6.22E-43 | 4.70E-44 | up |
| VIT_13s0019g00480.t01 | 775 | zinc finger protein ZAT12 | 504 | 43.915039 | 50 | 5.865414 | 2.9044102 | 2.20E-71 | 1.04E-72 | up |
| VIT_03s0017g01140.t01 | 1677 | UDP-glycosyltransferase 85A2-like | 373 | 15.019664 | 37 | 2.0058527 | 2.904565 | 4.44E-53 | 2.73E-54 | up |
| VIT_14s0060g01990.t01 | 995 | F-box protein PP2-B15-like | 242 | 16.423917 | 24 | 2.1928985 | 2.9048872 | 8.45E-35 | 7.66E-36 | up |
| VIT_16s0039g01130.t01 | 2142 | Phenylalanine ammonia-lyase | 253 | 7.9760065 | 25 | 1.0610868 | 2.9101239 | 2.12E-36 | 1.86E-37 | up |
| VIT_18s0001g09650.t01 | 1879 | cytochrome P450 81F1-like | 2071 | 74.428242 | 203 | 9.8219932 | 2.9217624 | 2.10E-292 | 2.44E-294 | up |
| VIT_13s0084g00670.t01 | 526 | Putative uncharacterized protein | 727 | 93.332545 | 71 | 12.27165 | 2.9270509 | 2.44E-103 | 7.92E-105 | up |
| VIT_07s0104g00930.t01 | 1699 | gibberellin receptor GID1B | 1157 | 45.985872 | 112 | 5.9931482 | 2.9398047 | 1.17E-164 | 2.35E-166 | up |
| VIT_01s0026g00900.t01 | 1310 | metalloendoproteinase 1 | 155 | 7.9899644 | 15 | 1.0409991 | 2.9402203 | 6.33E-23 | 8.13E-24 | up |
| VIT_03s0097g00580.t01 | 332 | Putative uncharacterized protein | 114 | 23.187355 | 11 | 3.0122081 | 2.9444449 | 3.62E-17 | 5.85E-18 | up |
| VIT_06s0004g06480.t01 | 1857 | 2-methylbutanal oxime monooxygenase-like | 425 | 15.454732 | 41 | 2.007254 | 2.9447535 | 3.76E-61 | 2.02E-62 | up |
| VIT_13s0019g01360.t01 | 928 | Putative uncharacterized protein | 197 | 14.335165 | 19 | 1.8613841 | 2.9451108 | 6.81E-29 | 7.17E-30 | up |
| VIT_07s0005g01360.t01 | 837 | Putative uncharacterized protein | 166 | 13.392667 | 16 | 1.7379005 | 2.9460259 | 1.61E-24 | 1.95E-25 | up |
| VIT_03s0038g01960.t01 | 1224 | Putative uncharacterized protein | 301 | 16.606172 | 29 | 2.1540062 | 2.9466252 | 1.24E-43 | 9.27E-45 | up |
| VIT_18s0001g08760.t01 | 445 | phytosulfokines 3-like | 727 | 110.32117 | 70 | 14.301066 | 2.947515 | 4.83E-104 | 1.56E-105 | up |
| VIT_15s0046g02170.t01 | 1383 | 3-methyl-2-oxobutanoate hydroxymethyltransferase-like | 167 | 8.1541507 | 16 | 1.0517879 | 2.9546908 | 9.95E-25 | 1.19E-25 | up |
| VIT_17s0000g08450.t01 | 995 | carbonic anhydrase | 136 | 9.2299696 | 13 | 1.18782 | 2.9580096 | 2.32E-20 | 3.29E-21 | up |
| VIT_05s0077g00430.t01 | 1193 | glycogenin-2-like | 136 | 7.6980886 | 13 | 0.9906797 | 2.9580096 | 2.32E-20 | 3.29E-21 | up |
| VIT_18s0001g02770.t01 | 1371 | esterase-like | 798 | 39.305188 | 76 | 5.0397212 | 2.9633039 | 1.04E-114 | 3.04E-116 | up |
| VIT_19s0014g04780.t01 | 654 | Putative uncharacterized protein | 1002 | 103.46046 | 95 | 13.20615 | 2.9697977 | 4.05E-144 | 9.32E-146 | up |
| VIT_19s0140g00230.t01 | 271 | loricrin-like | 211 | 52.577219 | 20 | 6.7095142 | 2.9701576 | 4.01E-31 | 3.99E-32 | up |
| VIT_13s0084g00130.t01 | 1205 | carboxylesterase 15 | 4959 | 277.90189 | 465 | 35.082964 | 2.9857331 | 0 | 0 | up |
| VIT_03s0017g01410.t01 | 524 | Putative uncharacterized protein | 736 | 94.84861 | 69 | 11.971489 | 2.986024 | 1.21E-106 | 3.79E-108 | up |
| VIT_08s0007g08460.t01 | 1109 | Putative uncharacterized protein | 160 | 9.7425553 | 15 | 1.2296743 | 2.986024 | 5.71E-24 | 7.03E-25 | up |
| VIT_04s0008g05400.t01 | 1155 | carboxylesterase 17 | 171 | 9.9976647 | 16 | 1.2594136 | 2.988839 | 1.44E-25 | 1.68E-26 | up |
| VIT_13s0067g02500.t01 | 1961 | adenine/guanine permease AZG1-like | 2165 | 74.552936 | 202 | 9.3649217 | 2.9929263 | 0 | 0 | up |
| VIT_04s0044g00870.t01 | 1576 | U-box domain-containing protein 27 | 301 | 12.897179 | 28 | 1.6152219 | 2.9972513 | 2.42E-44 | 1.78E-45 | up |
| VIT_00s1679g00010.t01 | 647 | Putative uncharacterized protein | 172 | 17.951825 | 16 | 2.2482576 | 2.9972513 | 8.89E-26 | 1.03E-26 | up |
| VIT_09s0002g00700.t01 | 934 | auxin-repressed 12.5 kDa protein | 2423 | 175.18261 | 225 | 21.901104 | 2.999784 | 0 | 0 | up |
| VIT_07s0141g00020.t01 | 1398 | protein SENSITIVE TO PROTON RHIZOTOXICITY 1-lik | 2332 | 112.64342 | 216 | 14.046786 | 3.0034511 | 0 | 0 | up |
| VIT_01s0011g06140.t01 | 1623 | U-box domain-containing protein 21-like | 1297 | 53.964219 | 120 | 6.7219163 | 3.0050587 | 1.90E-188 | 3.37E-190 | up |
| VIT_03s0038g03840.t01 | 916 | ser/Thr-rich protein T10 in DGCR region-like | 98 | 7.2246206 | 9 | 0.893259 | 3.0157714 | 3.09E-15 | 5.51E-16 | up |
| VIT_03s0088g00910.t01 | 1005 | pathogenesis-related protein PR-1 | 273 | 18.34345 | 25 | 2.2615402 | 3.0198875 | 1.36E-40 | 1.08E-41 | up |
| VIT_16s0050g01430.t01 | 1309 | Putative uncharacterized protein | 580 | 29.920772 | 53 | 3.6810066 | 3.0229751 | 3.22E-85 | 1.26E-86 | up |
| VIT_19s0014g00700.t01 | 414 | probable LRR receptor-like serine/threonine-protein kinase | 88 | 14.353796 | 8 | 1.7567907 | 3.0304181 | 7.54E-14 | 1.44E-14 | up |
| VIT_02s0154g00300.t01 | 713 | 14 kDa proline-rich protein | 176 | 16.668925 | 16 | 2.040144 | 3.0304181 | 1.28E-26 | 1.44E-27 | up |
| VIT_11s0065g00970.t01 | 166 | replication protein A 70 kDa DNA-binding subunit B-like | 22 | 8.9495054 | 2 | 1.0953484 | 3.0304181 | 0.00027395 | 0.00011981 | up |
| VIT_18s0001g01310.t01 | 995 | wall-associated receptor kinase 2-like | 144 | 9.772909 | 13 | 1.18782 | 3.0404718 | 4.86E-22 | 6.43E-23 | up |
| VIT_18s0001g02640.t01 | 612 | acetyltransferase | 111 | 12.247741 | 10 | 1.4855215 | 3.0434743 | 2.98E-17 | 4.81E-18 | up |
| VIT_18s0001g10150.t01 | 780 | ethylene-responsive transcription factor ERF010-like | 623 | 53.935895 | 56 | 6.5271531 | 3.0467199 | 3.83E-92 | 1.39E-93 | up |
| VIT_00s0434g00090.t01 | 586 | aspartic proteinase-like protein 1-lik | 67 | 7.720788 | 6 | 0.9308592 | 3.0521132 | 7.10E-11 | 1.64E-11 | up |
| VIT_05s0049g00940.t01 | 1733 | probable inorganic phosphate transporter 1-7-like | 16411 | 639.47111 | 1467 | 76.959444 | 3.0547089 | 0 | 0 | up |
| VIT_08s0040g02200.t01 | 1024 | peroxidase 21 | 112 | 7.3858845 | 10 | 0.8878312 | 3.0564133 | 1.84E-17 | 2.93E-18 | up |
| VIT_01s0026g00910.t01 | 405 | Putative uncharacterized protein | 147 | 24.510194 | 13 | 2.9182245 | 3.0702191 | 1.13E-22 | 1.46E-23 | up |
| VIT_16s0039g02500.t01 | 900 | probable gluconokinase-like | 147 | 11.029587 | 13 | 1.313201 | 3.0702191 | 1.13E-22 | 1.46E-23 | up |
| VIT_13s0067g03750.t01 | 2407 | ABC transporter G family member 6-like | 249 | 6.9856641 | 22 | 0.830954 | 3.0715568 | 1.07E-37 | 9.03E-39 | up |
| VIT_04s0008g01590.t01 | 716 | 17.3 kDa class II heat shock protein | 1031 | 97.236672 | 91 | 11.554702 | 3.0730205 | 3.82E-153 | 8.22E-155 | up |
| VIT_05s0020g03140.t01 | 1808 | hexose transporter (HT5) | 2161 | 80.712497 | 190 | 9.5540068 | 3.0786143 | 0 | 0 | up |
| VIT_00s0187g00010.t01 | 803 | Putative uncharacterized protein | 207 | 17.407614 | 18 | 2.0379209 | 3.0945485 | 1.01E-31 | 9.83E-33 | up |
| VIT_00s2707g00010.t01 | 349 | anthocyanidin 5,3-O-glucosyltransferase-like | 46 | 8.9005501 | 4 | 1.0419933 | 3.0945485 | 6.71E-08 | 1.94E-08 | up |
| VIT_15s0048g01960.t01 | 1063 | cytochrome P450 87A3-like | 115 | 7.3054844 | 10 | 0.8552579 | 3.0945485 | 4.25E-18 | 6.61E-19 | up |
| VIT_16s0050g01690.t01 | 1985 | probable receptor-like protein kinase | 599 | 20.377493 | 52 | 2.381624 | 3.096959 | 5.55E-90 | 2.06E-91 | up |
| VIT_18s0089g01270.t01 | 757 | 22.0 kDa class IV heat shock protein | 151 | 13.469935 | 13 | 1.5612694 | 3.1089515 | 1.61E-23 | 2.01E-24 | up |
| VIT_09s0002g08470.t01 | 1457 | quinone oxidoreductase 1-like | 489 | 22.663853 | 42 | 2.6207169 | 3.1123597 | 5.83E-74 | 2.62E-75 | up |
| VIT_16s0050g01550.t01 | 1005 | leucine-rich repeat receptor-like protein kinase PXL1 | 82 | 5.5097543 | 7 | 0.6332313 | 3.1211836 | 2.61E-13 | 5.10E-14 | up |
| VIT_18s0041g00840.t01 | 1510 | anthocyanidin 5,3-O-glucosyltransferase-like | 364 | 16.278294 | 31 | 1.8664447 | 3.1245848 | 1.69E-55 | 1.00E-56 | up |
| VIT_06s0009g00370.t01 | 1458 | E3 ubiquitin-protein ligase PUB24-like | 188 | 8.7073253 | 16 | 0.9976836 | 3.1255754 | 3.65E-29 | 3.82E-30 | up |
| VIT_10s0003g00090.t01 | 724 | auxin-repressed 12.5 kDa protein | 3608 | 336.52118 | 306 | 38.424943 | 3.1305823 | 0 | 0 | up |
| VIT_01s0011g01140.t01 | 2143 | cationic amino acid transporter 5 | 821 | 25.870536 | 69 | 2.927233 | 3.1437005 | 1.07E-124 | 2.88E-126 | up |
| VIT_03s0017g00670.t01 | 1376 | RING-H2 finger protein ATL54 | 131 | 6.4289094 | 11 | 0.7267828 | 3.1449779 | 9.38E-21 | 1.30E-21 | up |
| VIT_11s0016g01430.t01 | 651 | RING-H2 finger protein ATL39-like | 179 | 18.567631 | 15 | 2.0947907 | 3.1479117 | 5.45E-28 | 5.87E-29 | up |
| VIT_18s0001g04640.t01 | 580 | aminotransferase ALD1-like | 337 | 39.236147 | 28 | 4.3889477 | 3.1602364 | 5.58E-52 | 3.52E-53 | up |
| VIT_10s0116g00430.t01 | 1083 | U-box domain-containing protein 33-like | 181 | 11.285857 | 15 | 1.2591955 | 3.1639418 | 2.04E-28 | 2.17E-29 | up |
| VIT_14s0108g00540.t01 | 1984 | 6-phosphofructokinase 5, chloroplastic-like | 350 | 11.912717 | 29 | 1.3288829 | 3.1642166 | 5.29E-54 | 3.19E-55 | up |
| VIT_12s0028g01700.t01 | 707 | WRKY transcription factor 65 | 85 | 8.1186525 | 7 | 0.9001378 | 3.1730225 | 5.98E-14 | 1.13E-14 | up |
| VIT_13s0067g01270.t01 | 1535 | probable protein phosphatase 2C 25-like | 160 | 7.0387582 | 13 | 0.769955 | 3.1924749 | 1.93E-25 | 2.27E-26 | up |
| VIT_03s0088g00780.t01 | 693 | pathogenesis-related protein PR-1 | 197 | 19.196296 | 16 | 2.0990226 | 3.1930383 | 4.35E-31 | 4.34E-32 | up |
| VIT_13s0101g00460.t01 | 760 | Putative uncharacterized protein | 308 | 27.366646 | 25 | 2.9905894 | 3.1939169 | 4.85E-48 | 3.30E-49 | up |
| VIT_06s0004g07770.t01 | 1205 | Peroxidase 4 | 2052 | 114.99389 | 166 | 12.524241 | 3.1987621 | 0 | 0 | up |
| VIT_06s0004g05770.t01 | 670 | class I heat shock protein | 124 | 12.497735 | 10 | 1.3569241 | 3.2032547 | 5.15E-20 | 7.39E-21 | up |
| VIT_00s0780g00020.t01 | 1731 | glucan endo-1,3-beta-glucosidase 5-like | 2306 | 89.959427 | 185 | 9.7163921 | 3.2107818 | 0 | 0 | up |
| VIT_03s0038g00250.t01 | 1502 | purple acid phosphatase 2-like | 1085 | 48.780275 | 87 | 5.2659859 | 3.2115223 | 1.08E-167 | 2.11E-169 | up |
| VIT_12s0121g00430.t01 | 218 | BRASSINOSTEROID INSENSITIVE 1-associated receptor kinase 1-like | 25 | 7.7440466 | 2 | 0.8340726 | 3.2148427 | 6.32E-05 | 2.52E-05 | up |
| VIT_00s0338g00010.t01 | 1535 | F-box protein SKIP23-like | 163 | 7.1707349 | 13 | 0.769955 | 3.2192749 | 4.41E-26 | 5.06E-27 | up |
| VIT_09s0018g01350.t01 | 162 | Putative uncharacterized protein | 251 | 104.62685 | 20 | 11.22394 | 3.220602 | 1.26E-39 | 1.03E-40 | up |
| VIT_15s0046g01970.t01 | 1381 | putative UDP-rhamnose:rhamnosyltransferase 1 | 151 | 7.3835924 | 12 | 0.7899834 | 3.2244287 | 2.85E-24 | 3.47E-25 | up |
| VIT_16s0098g01380.t01 | 555 | protein yippee-like | 151 | 18.372506 | 12 | 1.9657063 | 3.2244287 | 2.85E-24 | 3.47E-25 | up |
| VIT_04s0044g00040.t01 | 850 | dirigent protein 19-like | 316 | 25.104559 | 25 | 2.6739388 | 3.2309111 | 9.29E-50 | 6.14E-51 | up |
| VIT_02s0236g00100.t01 | 534 | Putative uncharacterized protein | 1295 | 163.76193 | 102 | 17.36558 | 3.2372975 | 2.06E-201 | 3.33E-203 | up |
| VIT_17s0000g00650.t01 | 467 | Putative uncharacterized protein | 51 | 7.3745876 | 4 | 0.7787059 | 3.2434119 | 5.73E-09 | 1.53E-09 | up |
| VIT_09s0002g03340.t01 | 821 | Putative uncharacterized protein | 524 | 43.099534 | 41 | 4.5401591 | 3.2468575 | 3.20E-82 | 1.30E-83 | up |
| VIT_17s0000g04880.t01 | 730 | dirigent protein 22 | 90 | 8.3253805 | 7 | 0.8717773 | 3.2554847 | 5.05E-15 | 9.12E-16 | up |
| VIT_13s0156g00100.t01 | 582 | Putative uncharacterized protein | 169 | 19.608671 | 13 | 2.0307232 | 3.2714262 | 2.27E-27 | 2.50E-28 | up |
| VIT_12s0034g01890.t01 | 1258 | glutelin type-A 3 | 157 | 8.4275911 | 12 | 0.8672234 | 3.2806448 | 1.46E-25 | 1.71E-26 | up |
| VIT_04s0008g03990.t01 | 495 | dehydration-responsive protein RD22-like | 79 | 10.77721 | 6 | 1.1019869 | 3.2898048 | 1.99E-13 | 3.87E-14 | up |
| VIT_12s0035g01340.t01 | 1006 | glucuronoxylan 4-O-methyltransferase 1 | 79 | 5.3029014 | 6 | 0.5422301 | 3.2898048 | 1.99E-13 | 3.87E-14 | up |
| VIT_18s0001g07380.t01 | 1161 | putative DNA-binding protein ESCAROLA | 528 | 30.710448 | 40 | 3.1322625 | 3.2934525 | 7.38E-84 | 2.94E-85 | up |
| VIT_05s0094g00220.t01 | 982 | endochitinase PR4 | 1071 | 73.648249 | 81 | 7.4990095 | 3.2958793 | 2.64E-169 | 5.06E-171 | up |
| VIT_05s0049g00410.t01 | 1285 | 1-aminocyclopropane-1-carboxylate oxidase | 635 | 33.36991 | 48 | 3.3960063 | 3.2966368 | 9.37E-101 | 3.14E-102 | up |
| VIT_07s0031g01130.t01 | 1982 | L-ascorbate oxidase-like | 397 | 13.52606 | 30 | 1.3760936 | 3.2970911 | 2.42E-63 | 1.26E-64 | up |
| VIT_04s0044g00270.t01 | 1385 | 3-hydroxybenzoate 6-hydroxylase 1-like | 120 | 5.8508089 | 9 | 0.5907764 | 3.3079521 | 6.34E-20 | 9.12E-21 | up |

| | | | | | | | | | | |
|---|---|---|---|---|---|---|---|---|---|---|
| VIT_07s0141g00360.t01 | 1704 | phospholipase A1-Igamma2 | 242 | 9.5902564 | 18 | 0.9603583 | 3.3199247 | 3.12E-39 | 2.56E-40 | up |
| VIT_00s0153g00050.t01 | 878 | glutathione S-transferase | 1053 | 80.987557 | 78 | 8.076635 | 3.325874 | 9.26E-168 | 1.80E-169 | up |
| VIT_13s0019g03880.t01 | 508 | mitochondrial import inner membrane translocase subunit TIM14-like | 54 | 7.1781824 | 4 | 0.7158576 | 3.325874 | 1.30E-09 | 3.30E-10 | up |
| VIT_05s0049g01980.t01 | 1736 | 3-isopropylmalate dehydratase-like | 149 | 5.7959014 | 11 | 0.5760675 | 3.3307234 | 1.29E-24 | 1.55E-25 | up |
| VIT_11s0016g05530.t01 | 783 | blue copper protein | 1979 | 170.67444 | 144 | 16.719801 | 3.3516174 | 0 | 0 | up |
| VIT_01s0010g02460.t01 | 1372 | glyceraldehyde-3-phosphate dehydrogenase-like | 1847 | 90.906979 | 133 | 8.8130839 | 3.3666722 | 9.53E-297 | 1.07E-298 | up |
| VIT_14s0083g01070.t01 | 254 | Putative uncharacterized protein | 70 | 18.610103 | 5 | 1.789644 | 3.3783414 | 2.86E-12 | 5.96E-13 | up |
| VIT_05s0094g00160.t01 | 502 | Putative uncharacterized protein | 126 | 16.949281 | 9 | 1.6299308 | 3.3783414 | 3.21E-21 | 4.38E-22 | up |
| VIT_05s0020g03710.t01 | 723 | alanine acetyl transferase-like (AAT) | 2760 | 257.78357 | 195 | 24.520351 | 3.3941087 | 0 | 0 | up |
| VIT_09s0002g09140.t01 | 534 | ethylene-responsive transcription factor ERF003-like | 57 | 7.2080542 | 4 | 0.6810031 | 3.4038765 | 2.94E-10 | 7.09E-11 | up |
| VIT_10s0003g00980.t01 | 461 | Putative uncharacterized protein | 215 | 31.493576 | 15 | 2.9581535 | 3.4122888 | 9.37E-36 | 8.34E-37 | up |
| VIT_08s0007g05270.t01 | 1098 | Putative uncharacterized protein | 518 | 31.857513 | 36 | 2.9807842 | 3.4178698 | 7.51E-85 | 2.95E-86 | up |
| VIT_06s0004g04180.t01 | 679 | zinc finger protein ZAT11-like | 5073 | 504.52133 | 351 | 46.996738 | 3.4242827 | 0 | 0 | up |
| VIT_08s0007g06060.t01 | 1186 | Beta-1,3-glucanase | 3570 | 203.26751 | 247 | 18.934012 | 3.4243276 | 0 | 0 | up |
| VIT_08s0007g02370.t01 | 1060 | Putative uncharacterized protein | 1547 | 98.552783 | 107 | 9.1771596 | 3.424777 | 3.70E-252 | 4.73E-254 | up |
| VIT_01s0026g00190.t01 | 2230 | U-box domain-containing protein 19 | 174 | 5.2690076 | 12 | 0.4892229 | 3.4289675 | 3.06E-29 | 3.19E-30 | up |
| VIT_05s0077g01920.t01 | 455 | Putative uncharacterized protein | 436 | 64.708232 | 30 | 5.9943242 | 3.4322802 | 8.16E-72 | 3.82E-73 | up |
| VIT_07s0104g00800.t01 | 1967 | indole-3-acetic acid-amido synthetase GH3.6 | 218 | 7.4840482 | 15 | 0.6932937 | 3.4322802 | 2.09E-36 | 1.83E-37 | up |
| VIT_16s0039g01120.t01 | 2219 | phenylalanine ammonia-lyase | 496 | 15.094155 | 34 | 1.3930028 | 3.43772 | 1.14E-81 | 4.69E-83 | up |
| VIT_19s0014g04790.t01 | 1671 | organic cation transporter protein-like | 265 | 10.709122 | 18 | 0.9793241 | 3.4509101 | 3.18E-44 | 2.34E-45 | up |
| VIT_04s0008g01490.t01 | 524 | 17.3 kDa class II heat shock protein | 2826 | 364.18773 | 191 | 33.13847 | 3.4581034 | 0 | 0 | up |
| VIT_08s0007g01410.t01 | 942 | glutathione S-transferase | 74 | 5.3047541 | 5 | 0.4825579 | 3.4585118 | 3.89E-13 | 7.69E-14 | up |
| VIT_19s0090g01790.t01 | 1770 | U-box domain-containing protein 14-like | 148 | 5.6464163 | 10 | 0.513638 | 3.4585118 | 3.40E-25 | 4.02E-26 | up |
| VIT_13s0019g05070.t01 | 2122 | Putative uncharacterized protein | 193 | 6.1418099 | 13 | 0.5569656 | 3.4630038 | 1.42E-32 | 1.36E-33 | up |
| VIT_04s0008g03200.t01 | 584 | lachrymatory-factor synthase | 104 | 12.02555 | 7 | 1.0897216 | 3.4640713 | 4.78E-18 | 7.45E-19 | up |
| VIT_00s2769g00010.t01 | 184 | protein preY | 30 | 11.010014 | 2 | 0.9881948 | 3.4778771 | 5.23E-06 | 1.84E-06 | up |
| VIT_05s0049g00520.t01 | 716 | proline-rich cell wall protein-like (GRIP4) | 360 | 33.952669 | 24 | 3.0473939 | 3.4778771 | 4.42E-60 | 2.42E-61 | up |
| VIT_02s0025g04330.t01 | 899 | thaumatin-like protein | 662 | 49.72591 | 44 | 4.4496244 | 3.4822423 | 8.20E-110 | 2.49E-111 | up |
| VIT_18s0001g02760.t01 | 1748 | esterase-like | 739 | 28.548773 | 49 | 2.5485023 | 3.4857072 | 1.40E-122 | 3.81E-124 | up |
| VIT_03s0088g00750.t01 | 646 | pathogenesis-related protein PR-1 | 651 | 68.05075 | 43 | 6.0515456 | 3.4912355 | 3.21E-108 | 9.88E-110 | up |
| VIT_01s0011g02790.t01 | 609 | Putative uncharacterized protein | 91 | 10.090404 | 6 | 0.8957036 | 3.4938186 | 5.01E-16 | 8.55E-17 | up |
| VIT_07s0141g00890.t01 | 1791 | cytochrome P450 94A1 | 488 | 18.399613 | 32 | 1.6243693 | 3.5017238 | 1.52E-81 | 6.26E-83 | up |
| VIT_17s0000g03960.t01 | 995 | protein PLANT CADMIUM RESISTANCE 6 | 140 | 9.5014393 | 9 | 0.8223369 | 3.5303445 | 2.90E-24 | 3.54E-25 | up |
| VIT_06s0004g05670.t01 | 1329 | glutathione S-transferase | 343 | 17.428242 | 22 | 1.5049708 | 3.5336197 | 5.36E-58 | 3.03E-59 | up |
| VIT_11s0016g03080.t01 | 3545 | receptor-like protein kinase HSL1-like | 344 | 6.552796 | 22 | 0.5642048 | 3.5378196 | 3.24E-58 | 1.82E-59 | up |
| VIT_01s0011g04900.t01 | 1320 | serine carboxypeptidase-like 50 | 347 | 17.751702 | 22 | 1.515232 | 3.5503467 | 7.11E-59 | 3.98E-60 | up |
| VIT_10s0071g00810.t01 | 1656 | Putative uncharacterized protein | 3267 | 133.22117 | 207 | 11.36424 | 3.5512503 | 0 | 0 | up |
| VIT_19s0090g01340.t01 | 496 | cold and drought-regulated protein CORA | 128806 | 17536.336 | 8159 | 1495.4973 | 3.5516502 | 0 | 0 | up |
| VIT_03s0017g01460.t01 | 489 | cysteine-rich and transmembrane domain-containing protein A-like | 588 | 81.199417 | 37 | 6.8789672 | 3.5612055 | 2.40E-99 | 8.15E-101 | up |
| VIT_13s0067g02410.t01 | 165 | Putative uncharacterized protein | 16 | 6.5481781 | 1 | 0.5509934 | 3.5709865 | 0.00091157 | 0.00043184 | up |
| VIT_11s0052g01550.t01 | 399 | MATE efflux family protein | 32 | 5.4157864 | 2 | 0.4557089 | 3.5709865 | 1.92E-06 | 6.43E-07 | up |
| VIT_06s0004g01010.t01 | 851 | dirigent protein 22 | 417 | 33.089556 | 26 | 2.7776285 | 3.5744504 | 6.34E-71 | 3.01E-72 | up |
| VIT_03s0017g01940.t01 | 868 | protein bunched, class 2/F/G isoform | 834 | 64.882977 | 52 | 5.4464559 | 3.5744504 | 6.21E-141 | 1.45E-142 | up |
| VIT_17s0119g00120.t01 | 727 | miraculin-like | 225 | 20.899339 | 14 | 1.7507494 | 3.5774128 | 9.49E-39 | 7.85E-40 | up |
| VIT_18s0001g08300.t01 | 872 | Putative uncharacterized protein | 113 | 8.7507727 | 7 | 0.7298136 | 3.5838105 | 5.21E-20 | 7.48E-21 | up |
| VIT_06s0004g01670.t01 | 1693 | UDP-glycosyltransferase 92A1 | 179 | 7.1397091 | 11 | 0.5906988 | 3.5953707 | 3.76E-31 | 3.75E-32 | up |
| VIT_16s0039g01320.t01 | 466 | phenylalanine ammonia-lyase | 49 | 7.1005928 | 3 | 0.5852827 | 3.6007339 | 2.49E-09 | 6.45E-10 | up |
| VIT_00s0332g00120.t01 | 96 | Putative uncharacterized protein | 82 | 57.680241 | 5 | 4.7350999 | 3.6066104 | 6.93E-15 | 1.26E-15 | up |
| VIT_05s0049g00750.t01 | 551 | proline-rich 33 kDa extensin-related protein-like | 263 | 32.232099 | 16 | 2.6399686 | 3.6099055 | 2.00E-45 | 1.44E-46 | up |
| VIT_01s0010g02930.t01 | 424 | probable calcium-binding protein CML31-like | 182 | 28.986113 | 11 | 2.3586158 | 3.6193495 | 8.29E-32 | 8.10E-33 | up |
| VIT_02s0025g02560.t01 | 1542 | methionine gamma-lyase | 11534 | 505.10308 | 696 | 41.035076 | 3.6216483 | 0 | 0 | up |
| VIT_03s0063g00880.t01 | 1232 | Putative uncharacterized protein | 2466 | 135.1658 | 148 | 10.921477 | 3.6294902 | 0 | 0 | up |
| VIT_14s0083g00460.t01 | 1762 | tryptophan synthase beta chain 1 | 270 | 10.347664 | 16 | 0.825552 | 3.6478021 | 5.83E-47 | 4.06E-48 | up |
| VIT_18s0001g09510.t01 | 1731 | isoflavone 2'-hydroxylase-like | 633 | 24.69398 | 37 | 1.9432784 | 3.6675948 | 3.10E-109 | 9.48E-111 | up |
| VIT_16s0100g00270.t01 | 450 | Putative uncharacterized protein | 137 | 20.558551 | 8 | 1.6162474 | 3.6690186 | 1.96E-24 | 2.37E-25 | up |
| VIT_05s0049g00610.t01 | 726 | early nodulin-75-like | 1062 | 98.780755 | 62 | 7.7639985 | 3.6693582 | 8.94E-183 | 1.64E-184 | up |
| VIT_14s0068g01360.t01 | 1252 | GEM-like protein 5-like | 179 | 9.6545747 | 10 | 0.7261495 | 3.7328742 | 5.49E-32 | 5.34E-33 | up |
| VIT_05s0094g00360.t01 | 1037 | class IV chitinase (CHI4C) | 3807 | 247.90687 | 212 | 18.586066 | 3.7375049 | 0 | 0 | up |
| VIT_17s0000g07670.t01 | 959 | betaine aldehyde dehydrogenase 2 | 216 | 15.209663 | 12 | 1.137609 | 3.7409115 | 1.91E-38 | 1.59E-39 | up |
| VIT_08s0058g00150.t01 | 1706 | ammonium transporter 2-like | 506 | 20.028846 | 28 | 1.4921393 | 3.7466252 | 7.89E-89 | 2.97E-90 | up |
| VIT_05s0049g00730.t01 | 650 | early nodulin-75-like | 975 | 101.29213 | 53 | 7.412981 | 3.7723245 | 3.60E-171 | 6.85E-173 | up |
| VIT_14s0068g01160.t01 | 693 | light-regulated protein-like | 222 | 21.632374 | 12 | 1.574267 | 3.7804399 | 9.06E-40 | 7.37E-41 | up |
| VIT_01s0127g00250.t01 | 1264 | F-box/kelch protein | 168 | 8.975252 | 9 | 0.6473301 | 3.7933789 | 2.06E-30 | 2.09E-31 | up |
| VIT_10s0597g00050.t01 | 754 | heat stress transcription factor B-2a-like | 112 | 10.030697 | 6 | 0.7234529 | 3.7933789 | 1.26E-20 | 1.76E-21 | up |
| VIT_02s0236g00120.t01 | 515 | Putative uncharacterized protein | 320 | 41.959199 | 17 | 3.0010419 | 3.8054518 | 3.98E-57 | 2.29E-58 | up |
| VIT_19s0014g03130.t01 | 972 | stem-specific protein TSJT1-like | 872 | 60.580752 | 46 | 4.3025105 | 3.8156089 | 2.26E-154 | 4.85E-156 | up |
| VIT_13s0064g00710.t01 | 192 | Putative uncharacterized protein | 19 | 6.6824669 | 1 | 0.47351 | 3.818914 | 0.00019949 | 8.51E-05 | up |
| VIT_00s2197g00010.t01 | 269 | 1-aminocyclopropane-1-carboxylate oxidase homolog 2-like | 38 | 9.5392836 | 2 | 0.6759399 | 3.818914 | 9.34E-08 | 2.74E-08 | up |
| VIT_17s0000g05360.t01 | 799 | Germin-like protein | 954 | 80.628028 | 50 | 5.6892314 | 3.8249758 | 4.37E-169 | 8.40E-171 | up |
| VIT_18s0001g09680.t01 | 456 | Putative uncharacterized protein | 39 | 5.7754284 | 2 | 0.3987453 | 3.8563887 | 5.63E-08 | 1.62E-08 | up |
| VIT_15s0048g00610.t01 | 465 | Putative uncharacterized protein | 197 | 28.608673 | 10 | 1.955138 | 3.8711102 | 5.86E-36 | 5.19E-37 | up |
| VIT_03s0063g01230.t01 | 1506 | Putative uncharacterized protein | 179 | 8.0262467 | 9 | 0.5433103 | 3.8848773 | 7.70E-33 | 7.34E-34 | up |
| VIT_16s0098g00690.t01 | 1640 | UNC93-like protein 1 | 717 | 29.52295 | 36 | 1.9956714 | 3.8868908 | 1.21E-128 | 3.13E-130 | up |
| VIT_01s0150g00120.t01 | 1245 | ethylene-responsive transcription factor ERF113 | 299 | 16.217589 | 15 | 1.0953484 | 3.8880976 | 3.39E-54 | 2.03E-55 | up |
| VIT_17s0000g01460.t01 | 1508 | SNF1-related protein kinase regulatory subunit gamma-1 | 342 | 15.314725 | 17 | 1.0248916 | 3.9013762 | 5.49E-62 | 2.91E-63 | up |
| VIT_16s0039g01240.t01 | 2308 | phenylalanine ammonia-lyase | 306 | 8.9530305 | 15 | 0.5908617 | 3.9214838 | 9.53E-56 | 5.61E-57 | up |
| VIT_01s0150g00410.t01 | 1451 | Putative uncharacterized protein | 205 | 9.5404946 | 10 | 0.6265604 | 3.9285385 | 1.00E-37 | 8.50E-39 | up |
| VIT_16s0050g00930.t01 | 1717 | MATE efflux family protein | 800 | 31.46329 | 39 | 2.065022 | 3.9294405 | 1.92E-144 | 4.40E-146 | up |
| VIT_02s0033g00670.t01 | 1082 | bifunctional nitrilase/nitrile hydratase | 124 | 7.7388935 | 6 | 0.5041437 | 3.9402203 | 2.85E-23 | 3.60E-24 | up |
| VIT_02s0025g04250.t01 | 713 | thaumatin-like protein | 9661 | 914.99136 | 465 | 59.291685 | 3.9478564 | 0 | 0 | up |
| VIT_03s0088g00810.t01 | 683 | pathogenesis-related protein PR-1 | 22492 | 2223.78 | 1080 | 143.75846 | 3.9512952 | 0 | 0 | up |
| VIT_07s0005g04890.t01 | 999 | glutathione S-transferase | 1854 | 125.32239 | 89 | 8.0994381 | 3.9516786 | 0 | 0 | up |
| VIT_16s0098g01060.t01 | 854 | small heat shock protein | 337 | 26.6475 | 16 | 1.7033052 | 3.9675913 | 9.65E-62 | 5.13E-63 | up |
| VIT_18s0001g01500.t01 | 521 | Putative uncharacterized protein | 449 | 58.19599 | 21 | 3.6644765 | 3.9892407 | 3.17E-82 | 1.29E-83 | up |
| VIT_05s0049g00660.t01 | 798 | early nodulin-75-like | 2142 | 181.2596 | 100 | 11.392722 | 3.9918731 | 0 | 0 | up |
| VIT_17s0000g02950.t01 | 923 | glutathione S-transferase | 1871 | 136.88521 | 87 | 8.5693508 | 3.9976369 | 0 | 0 | up |
| VIT_17s0000g03370.t01 | 1719 | Putative uncharacterized protein | 194 | 7.6209708 | 9 | 0.4759891 | 4.0009743 | 3.71E-36 | 3.27E-37 | up |
| VIT_14s0060g02300.t01 | 470 | Putative uncharacterized protein | 439 | 63.074106 | 20 | 3.8686773 | 4.0271355 | 7.09E-81 | 2.94E-82 | up |
| VIT_03s0017g01480.t01 | 500 | protein YIPF5 homolog | 46 | 6.212584 | 2 | 0.3636557 | 4.0945485 | 1.61E-09 | 4.11E-10 | up |
| VIT_13s0064g01370.t01 | 999 | polygalacturonase inhibitor-like | 92 | 6.2188028 | 4 | 0.3640197 | 4.0945485 | 6.20E-18 | 9.72E-19 | up |
| VIT_02s0025g04260.t01 | 642 | thaumatin-like protein | 4838 | 508.87988 | 207 | 29.313366 | 4.1176946 | 0 | 0 | up |
| VIT_01s0026g02590.t01 | 704 | probable calcium-binding protein CML30-like | 117 | 11.222708 | 5 | 0.6456954 | 4.1194231 | 1.37E-22 | 1.78E-23 | up |
| VIT_02s0087g00750.t01 | 1229 | transmembrane protein 45B | 235 | 12.912205 | 10 | 0.739739 | 4.1255754 | 2.32E-44 | 1.70E-45 | up |
| VIT_08s0058g00970.t01 | 1157 | Peroxidase 1 | 661 | 38.57914 | 28 | 2.200164 | 4.132138 | 3.77E-123 | 1.02E-124 | up |
| VIT_07s0031g01980.t01 | 1208 | ethylene-responsive transcription factor ERF110 | 357 | 19.956562 | 15 | 1.128898 | 4.1438762 | 4.90E-67 | 2.41E-68 | up |
| VIT_07s0031g01070.t01 | 1790 | L-ascorbate oxidase-like | 335 | 12.637938 | 14 | 0.7110586 | 4.1516489 | 4.99E-63 | 2.60E-64 | up |
| VIT_18s0001g01510.t01 | 732 | Putative uncharacterized protein | 144 | 13.284214 | 6 | 0.745196 | 4.155949 | 1.10E-27 | 1.19E-28 | up |

| | | | | | | | | | | |
|---|---|---|---|---|---|---|---|---|---|---|
| VIT_18s0166g00060.t01 | 129 | Putative uncharacterized protein | 48 | 25.12673 | 2 | 1.4095181 | 4.155949 | 5.85E-10 | 1.44E-10 | up |
| VIT_18s0001g15130.t01 | 1235 | Putative uncharacterized protein | 460 | 25.152162 | 19 | 1.3986757 | 4.168549 | 2.15E-86 | 8.34E-88 | up |
| VIT_14s0108g00930.t01 | 1863 | Putative uncharacterized protein | 464 | 16.818589 | 19 | 0.9271951 | 4.18104 | 2.80E-87 | 1.07E-88 | up |
| VIT_05s0049g00720.t01 | 1028 | early nodulin-75-like | 2628 | 172.63017 | 107 | 9.4628299 | 4.1892691 | 0 | 0 | up |
| VIT_04s0008g03530.t01 | 1960 | ankyrin repeat-containing protein | 713 | 24.565064 | 29 | 1.3451549 | 4.1907638 | 8.37E-134 | 2.05E-135 | up |
| VIT_16s0039g01100.t01 | 2246 | phenylalanine ammonia-lyase | 773 | 23.240967 | 31 | 1.2548225 | 4.2111148 | 2.23E-145 | 5.04E-147 | up |
| VIT_16s0100g00950.t01 | 560 | Chalcone and stilbene synthase | 50 | 6.0292934 | 2 | 0.3246926 | 4.2148427 | 2.12E-10 | 5.07E-11 | up |
| VIT_05s0077g01910.t01 | 598 | Putative uncharacterized protein | 76 | 8.5821648 | 3 | 0.4560899 | 4.2339515 | 2.89E-15 | 5.13E-16 | up |
| VIT_02s0025g04230.t01 | 701 | thaumatin-like protein | 32047 | 3087.1221 | 1260 | 163.41161 | 4.2396802 | 0 | 0 | up |
| VIT_16s0039g02850.t01 | 501 | non-specific lipid-transfer protein | 130 | 17.522258 | 5 | 0.9073245 | 4.2714262 | 1.86E-25 | 2.18E-26 | up |
| VIT_12s0028g00670.t01 | 674 | Putative uncharacterized protein | 1537 | 153.99209 | 59 | 7.95834 | 4.2742449 | 1.26E-290 | 1.48E-292 | up |
| VIT_19s0090g01360.t01 | 674 | glycine-rich protein DC7.1-like | 22716 | 2275.9169 | 871 | 117.48668 | 4.2758788 | 0 | 0 | up |
| VIT_02s0025g04270.t01 | 823 | thaumatin-like protein | 68361 | 5609.0978 | 2618 | 289.20126 | 4.277623 | 0 | 0 | up |
| VIT_06s0004g00800.t01 | 1991 | probable polyamine transporter | 523 | 17.738417 | 20 | 0.9132488 | 4.2797256 | 1.74E-99 | 5.91E-101 | up |
| VIT_12s0028g02530.t01 | 1016 | RING-H2 finger protein ATL40-like | 604 | 40.14465 | 23 | 2.0580907 | 4.2858293 | 7.74E-115 | 2.26E-116 | up |
| VIT_02s0025g04730.t01 | 1359 | glyoxylate reductase-like | 631 | 31.3541 | 24 | 1.6055438 | 4.2875202 | 5.90E-120 | 1.65E-121 | up |
| VIT_08s0007g07690.t01 | 1201 | polygalacturonase inhibiting protein | 264 | 14.843809 | 10 | 0.7569852 | 4.2934525 | 8.99E-51 | 5.82E-52 | up |
| VIT_14s0081g00030.t01 | 550 | pathogenesis-related protein PR4 | 2257 | 277.11071 | 84 | 13.885035 | 4.3188598 | 0 | 0 | up |
| VIT_16s0098g00860.t01 | 1133 | flavanone 3-dioxygenase-like | 1162 | 69.256519 | 43 | 3.4503958 | 4.3271161 | 3.07E-221 | 4.45E-223 | up |
| VIT_18s0001g15660.t01 | 903 | pathogenesis-related protein PR-1 | 435 | 32.530141 | 16 | 1.6108778 | 4.3358581 | 1.94E-83 | 7.75E-85 | up |
| VIT_14s0081g00050.t01 | 740 | pathogenesis-related protein PR4 | 2892 | 263.90706 | 105 | 12.899948 | 4.3545928 | 0 | 0 | up |
| VIT_02s0033g01400.t01 | 1022 | Alternative oxidase | 307 | 20.284856 | 11 | 0.9785255 | 4.3736497 | 2.06E-59 | 1.14E-60 | up |
| VIT_02s0087g00850.t01 | 162 | Putative uncharacterized protein | 14 | 5.8357606 | 0.5 | 0.2805985 | 4.3783414 | 0.00094295 | 0.00044839 | up |
| VIT_02s0012g01380.t01 | 653 | Putative uncharacterized protein | 57 | 5.8944884 | 2 | 0.27845 | 4.4038765 | 6.19E-12 | 1.32E-12 | up |
| VIT_00s0361g00060.t01 | 1922 | (E)-beta-ocimene/(E,E)-alpha-farnesene synthase | 691 | 24.277788 | 24 | 1.1352414 | 4.4185659 | 3.24E-133 | 7.95E-135 | up |
| VIT_01s0026g02670.t01 | 156 | Putative uncharacterized protein | 29 | 12.553298 | 1 | 0.5827815 | 4.4289675 | 1.24E-06 | 4.08E-07 | up |
| VIT_15s0046g01570.t01 | 957 | acidic endochitinase | 3772 | 266.16086 | 129 | 12.254854 | 4.4408732 | 0 | 0 | up |
| VIT_18s0001g11130.t01 | 1350 | Putative uncharacterized protein | 469 | 23.459757 | 16 | 1.0774983 | 4.4444306 | 5.96E-91 | 2.20E-92 | up |
| VIT_05s0049g01120.t01 | 931 | glutathione S-transferase | 384 | 27.852616 | 13 | 1.2694747 | 4.4555093 | 1.02E-74 | 4.55E-76 | up |
| VIT_18s0072g00160.t01 | 1154 | Peroxidase 12 | 3635 | 212.70762 | 123 | 9.6901316 | 4.4562117 | 0 | 0 | up |
| VIT_15s0048g01350.t01 | 1144 | probable carboxylesterase 8 | 1216 | 71.778106 | 41 | 3.2582785 | 4.461362 | 7.13E-235 | 9.64E-237 | up |
| VIT_11s0065g00130.t01 | 1719 | cytochrome P450 CYP716A15 (CYP716A15) | 534 | 20.977311 | 18 | 0.9519782 | 4.4617574 | 1.30E-103 | 4.21E-105 | up |
| VIT_05s0049g01090.t01 | 795 | glutathione S-transferase | 387 | 32.872163 | 13 | 1.4866427 | 4.4667365 | 2.25E-75 | 9.84E-77 | up |
| VIT_00s0173g00230.t01 | 141 | Putative uncharacterized protein | 15 | 7.183839 | 0.5 | 0.3223898 | 4.4778771 | 0.00056637 | 0.00026013 | up |
| VIT_16s0100g01010.t01 | 1364 | Chalcone and stilbene synthase | 121 | 5.9903948 | 4 | 0.2666097 | 4.4898497 | 2.55E-24 | 3.10E-25 | up |
| VIT_05s0049g00790.t01 | 628 | proline-rich 33 kDa extensin-related protein-like | 182 | 19.570242 | 6 | 0.8686043 | 4.4938186 | 4.48E-36 | 3.96E-37 | up |
| VIT_16s0050g01400.t01 | 713 | Putative uncharacterized protein | 335 | 31.727783 | 11 | 1.402599 | 4.4995722 | 1.37E-65 | 6.94E-67 | up |
| VIT_10s0003g01030.t01 | 1964 | Putative uncharacterized protein | 159 | 5.4668868 | 5 | 0.2314509 | 4.5619414 | 7.65E-32 | 7.47E-33 | up |
| VIT_16s0039g02890.t01 | 363 | non-specific lipid-transfer protein | 32 | 5.9528892 | 1 | 0.2504516 | 4.5709865 | 2.74E-07 | 8.42E-08 | up |
| VIT_18s0166g00260.t01 | 758 | extensin | 288 | 25.657109 | 9 | 1.0794528 | 4.5709865 | 6.74E-57 | 3.89E-58 | up |
| VIT_04s0008g02730.t01 | 198 | Putative uncharacterized protein | 16 | 5.4568151 | 0.5 | 0.2295806 | 4.5709865 | 0.00034165 | 0.00015147 | up |
| VIT_14s0068g02150.t01 | 1703 | F-box/kelch-repeat protein | 352 | 13.957655 | 11 | 0.5872302 | 4.5709865 | 2.48E-69 | 1.20E-70 | up |
| VIT_02s0033g01390.t01 | 330 | ubiquinol oxidase 2 | 97 | 19.849165 | 3 | 0.8264902 | 4.5859369 | 7.02E-20 | 1.01E-20 | up |
| VIT_19s0015g01440.t01 | 1615 | UPF0481 protein | 198 | 8.2789852 | 6 | 0.3377607 | 4.6153806 | 1.37E-39 | 1.12E-40 | up |
| VIT_18s0072g00260.t01 | 1641 | hypothetical protein | 166 | 6.8309947 | 5 | 0.2770077 | 4.6240978 | 2.23E-33 | 2.10E-34 | up |
| VIT_06s0004g02550.t01 | 883 | kiwellin | 337 | 25.772327 | 10 | 1.0296027 | 4.6456632 | 7.65E-67 | 3.79E-68 | up |
| VIT_07s0205g00060.t01 | 228 | tRNA-dihydrouridine synthase | 17 | 5.0349889 | 0.5 | 0.1993726 | 4.6584494 | 0.0002067 | 8.85E-05 | up |
| VIT_00s0125g00230.t01 | 214 | fatty acid amide hydrolase | 17 | 5.3643807 | 0.5 | 0.2124157 | 4.6584494 | 0.0002067 | 8.85E-05 | up |
| VIT_07s0031g02710.t01 | 191 | receptor-like protein kinase | 17 | 6.0103532 | 0.5 | 0.2379945 | 4.6584494 | 0.0002067 | 8.85E-05 | up |
| VIT_00s1355g00020.t01 | 458 | expansin-like B1 | 34 | 5.0130021 | 1 | 0.198502 | 4.6584494 | 1.00E-07 | 2.96E-08 | up |
| VIT_09s0018g00510.t01 | 75 | Putative uncharacterized protein | 17 | 15.306366 | 0.5 | 0.6060928 | 4.6584494 | 0.0002067 | 8.85E-05 | up |
| VIT_04s0008g00330.t01 | 537 | receptor-like protein kinase HSL1-like | 103 | 12.952315 | 3 | 0.507899 | 4.6725245 | 3.44E-21 | 4.69E-22 | up |
| VIT_13s0019g04060.t01 | 1743 | MLO7 protein (MLO7) | 172 | 6.663701 | 5 | 0.2607972 | 4.6753232 | 1.08E-34 | 9.87E-36 | up |
| VIT_05s0077g01150.t01 | 1206 | Beta-1,3-glucanase | 1382 | 77.382932 | 40 | 3.015387 | 4.6816003 | 2.97E-272 | 3.63E-274 | up |
| VIT_06s0009g00980.t01 | 1050 | carboxylesterase 15 | 104 | 6.6884962 | 3 | 0.2597541 | 4.6864637 | 2.08E-21 | 2.81E-22 | up |
| VIT_16s0100g01130.t01 | 1364 | Chalcone and stilbene synthase | 208 | 10.297538 | 6 | 0.3999146 | 4.6864637 | 8.95E-42 | 6.92E-43 | up |
| VIT_07s0104g00030.t01 | 414 | Putative uncharacterized protein | 140 | 22.835585 | 4 | 0.8783953 | 4.7002695 | 1.76E-28 | 1.87E-29 | up |
| VIT_06s0004g01990.t01 | 963 | extensin-like | 852 | 59.744475 | 24 | 2.2657674 | 4.7207336 | 1.24E-168 | 2.40E-170 | up |
| VIT_15s0048g00620.t01 | 386 | Putative uncharacterized protein | 71 | 12.420969 | 2 | 0.4710566 | 4.7207336 | 5.34E-15 | 9.65E-16 | up |
| VIT_02s0025g04280.t01 | 724 | thaumatin-like protein | 4340 | 404.79544 | 121 | 15.194177 | 4.7356026 | 0 | 0 | up |
| VIT_12s0035g00460.t01 | 123 | isease resistance protein RGA1 | 18 | 9.882159 | 0.5 | 0.3695688 | 4.7409115 | 0.00012504 | 5.19E-05 | up |
| VIT_12s0035g01000.t01 | 635 | Putative uncharacterized protein | 289 | 30.733255 | 8 | 1.1453722 | 4.7459122 | 6.55E-58 | 3.71E-59 | up |
| VIT_08s0007g07520.t01 | 1199 | short-chain type dehydrogenase/reductase-like | 4420 | 248.9359 | 119 | 9.0231495 | 4.7859994 | 0 | 0 | up |
| VIT_16s0039g01170.t01 | 2318 | phenylalanine ammonia-lyase | 458 | 13.342478 | 12 | 0.4706501 | 4.8252278 | 9.78E-92 | 3.57E-93 | up |
| VIT_03s0132g00340.t01 | 1475 | wall-associated receptor kinase-like 1-like | 156 | 7.1419536 | 4 | 0.2465462 | 4.8563887 | 5.76E-32 | 5.60E-33 | up |
| VIT_00s0392g00030.t01 | 1475 | (E,E)-alpha-farnesene synthase-like | 429 | 19.640372 | 11 | 0.6780021 | 4.8563887 | 3.65E-86 | 1.42E-87 | up |
| VIT_05s0049g00600.t01 | 474 | proline-rich 33 kDa extensin-related protein-like | 1658 | 236.20584 | 42 | 8.0556636 | 4.8738974 | 0 | 0 | up |
| VIT_13s0019g01490.t01 | 727 | elicitor-responsive protein 1-like | 80 | 7.4308761 | 2 | 0.2501071 | 4.8929146 | 5.99E-17 | 9.80E-18 | up |
| VIT_04s0023g03090.t01 | 206 | polyprenol reductase 2 | 20 | 6.5561249 | 0.5 | 0.2206648 | 4.8929146 | 4.58E-05 | 1.80E-05 | up |
| VIT_05s0094g00260.t01 | 861 | endochitinase PR4 | 164 | 12.862493 | 4 | 0.4223643 | 4.9285385 | 1.07E-33 | 9.92E-35 | up |
| VIT_16s0039g01870.t01 | 1607 | G-type lectin S-receptor-like serine/threonine-protein kinase | 249 | 10.463282 | 6 | 0.3394421 | 4.9460259 | 1.09E-50 | 7.06E-52 | up |
| VIT_12s0034g01930.t01 | 1612 | 11S globulin seed storage protein 2 | 166 | 6.9538848 | 4 | 0.2255928 | 4.9460259 | 3.94E-34 | 3.63E-35 | up |
| VIT_13s0074g00570.t01 | 1459 | lysine histidine transporter 1-like | 627 | 29.019952 | 15 | 0.9346873 | 4.9564175 | 2.78E-126 | 7.34E-128 | up |
| VIT_08s0058g00790.t01 | 988 | sex determination protein tasselseed-2-like | 502 | 34.310829 | 12 | 1.1042176 | 4.9575676 | 2.73E-101 | 9.08E-103 | up |
| VIT_05s0020g01830.t01 | 871 | UPF0497 membrane protein 15 | 1509 | 116.99183 | 36 | 3.7576361 | 4.9604386 | 6.37E-303 | 7.01E-305 | up |
| VIT_14s0083g00670.t01 | 440 | protein GLUTAMINE DUMPER 4 | 168 | 25.783451 | 4 | 0.8264902 | 4.9633039 | 1.45E-34 | 1.33E-35 | up |
| VIT_17s0000g07770.t01 | 209 | snakin-2-like | 21 | 6.7851187 | 0.5 | 0.2174974 | 4.9633039 | 2.79E-05 | 1.07E-05 | up |
| VIT_15s0048g02750.t01 | 567 | Putative uncharacterized protein | 85 | 10.123258 | 2 | 0.320684 | 4.9803774 | 5.00E-18 | 7.79E-19 | up |
| VIT_16s0039g01280.t01 | 2178 | phenylalanine ammonia-lyase | 258 | 7.9991948 | 6 | 0.2504516 | 4.9972513 | 1.25E-52 | 7.76E-54 | up |
| VIT_10s0003g02810.t01 | 1069 | WRKY transcription factor 71 | 86 | 5.4325682 | 2 | 0.1700915 | 4.9972513 | 3.05E-18 | 4.71E-19 | up |
| VIT_12s0055g01020.t01 | 970 | Peroxidase 2 | 262 | 18.239545 | 6 | 0.5623541 | 5.019447 | 1.72E-53 | 1.05E-54 | up |
| VIT_16s0039g01070.t01 | 876 | glutathione S-transferase, putative, expressed | 537 | 41.395642 | 12 | 1.2453961 | 5.0548023 | 7.69E-109 | 2.36E-110 | up |
| VIT_05s0049g00510.t01 | 1264 | ethylene-responsive transcription factor 1B | 359 | 19.179259 | 8 | 0.5754045 | 5.0588265 | 4.40E-73 | 2.01E-74 | up |
| VIT_13s0156g00590.t01 | 2427 | G-type lectin S-receptor-like serine/threonine-protein kinase RLK1 | 180 | 5.0082635 | 4 | 0.1498375 | 5.0628396 | 3.79E-37 | 3.25E-38 | up |
| VIT_06s0004g06830.t01 | 1981 | asparagine synthetase | 1633 | 55.665505 | 36 | 1.6521459 | 5.0743706 | 0 | 0 | up |
| VIT_14s0081g00020.t01 | 605 | pathogenesis-related protein PR4 | 182 | 20.314234 | 4 | 0.6010838 | 5.0787811 | 1.41E-37 | 1.20E-38 | up |
| VIT_00s0480g00040.t01 | 1050 | polyphenol oxidase | 4325 | 278.1514 | 95 | 8.2255449 | 5.0796153 | 0 | 0 | up |
| VIT_02s0087g00890.t01 | 165 | Putative uncharacterized protein | 23 | 9.413006 | 0.5 | 0.2754967 | 5.0945485 | 1.04E-05 | 3.77E-06 | up |
| VIT_02s0025g04320.t01 | 415 | thaumatin-like protein | 335 | 54.510624 | 7 | 1.5334878 | 5.1516489 | 1.53E-68 | 7.41E-70 | up |
| VIT_16s0050g00400.t01 | 916 | Putative uncharacterized protein | 341 | 25.138731 | 7 | 0.694757 | 5.1772595 | 7.92E-70 | 3.80E-71 | up |
| VIT_16s0100g00860.t01 | 1251 | Chalcone and stilbene synthase | 98 | 5.28997 | 2 | 0.145346 | 5.1856964 | 8.30E-21 | 1.15E-21 | up |
| VIT_03s0063g02330.t01 | 620 | organ-specific protein P4 | 50 | 5.4458134 | 1 | 0.1466354 | 5.2148427 | 3.65E-11 | 8.25E-12 | up |
| VIT_04s0069g00860.t01 | 1450 | probable sarcosine oxidase | 359 | 16.719023 | 7 | 0.4388948 | 5.2514716 | 1.17E-73 | 5.28E-75 | up |
| VIT_05s0020g01040.t01 | 541 | Putative uncharacterized protein | 52 | 6.4906848 | 1 | 0.1680479 | 5.2714262 | 1.38E-11 | 3.03E-12 | up |
| VIT_09s0054g00680.t01 | 543 | auxin-induced protein 6B-like | 52 | 6.4667781 | 1 | 0.1674289 | 5.2714262 | 1.38E-11 | 3.03E-12 | up |
| VIT_00s0179g00250.t01 | 222 | calcium-transporting ATPase 12, plasma membrane-type | 26 | 7.9086948 | 0.5 | 0.2047611 | 5.2714262 | 2.39E-06 | 8.10E-07 | up |
| VIT_00s0467g00020.t01 | 304 | disease resistance RPP13-like protein 1 | 26 | 5.7754284 | 0.5 | 0.1495295 | 5.2714262 | 2.39E-06 | 8.10E-07 | up |

| | | | | | | | | | | |
|---|---|---|---|---|---|---|---|---|---|---|
| VIT_04s0008g01500.t01 | 665 | 17.3 kDa class II heat shock protein | 158 | 16.044267 | 3 | 0.410138 | 5.2898048 | 5.07E-33 | 4.82E-34 | up |
| VIT_03s0088g00270.t01 | 547 | Putative uncharacterized protein | 219 | 27.035925 | 4 | 0.6648184 | 5.3457736 | 1.89E-45 | 1.36E-46 | up |
| VIT_05s0077g01580.t01 | 738 | Pathogenesis-related protein 10 | 59642 | 5457.3308 | 1078 | 132.79838 | 5.3608861 | 0 | 0 | up |
| VIT_01s0146g00320.t01 | 256 | Putative uncharacterized protein | 28 | 7.3858845 | 0.5 | 0.1775662 | 5.3783414 | 9.08E-07 | 2.94E-07 | up |
| VIT_00s0480g00030.t01 | 779 | polyphenol oxidase | 4416 | 382.80363 | 78 | 9.1030624 | 5.3941087 | 0 | 0 | up |
| VIT_01s0010g03930.t01 | 740 | WRKY transcription factor 75 | 568 | 51.832369 | 10 | 1.2285665 | 5.3988055 | 1.65E-116 | 4.77E-118 | up |
| VIT_04s0023g00530.t01 | 370 | auxin-induced protein 15A | 29 | 5.2927419 | 0.5 | 0.1228566 | 5.4289675 | 5.61E-07 | 1.78E-07 | up |
| VIT_05s0029g00320.t01 | 197 | Putative uncharacterized protein | 29 | 9.9406828 | 0.5 | 0.230746 | 5.4289675 | 5.61E-07 | 1.78E-07 | up |
| VIT_00s0392g00060.t01 | 1491 | alpha-farnesene synthase-like | 293 | 13.270107 | 5 | 0.3048756 | 5.4438153 | 1.32E-60 | 7.16E-62 | up |
| VIT_14s0030g01890.t01 | 781 | 28 kDa ookinete surface antigen | 533 | 46.085109 | 9 | 1.0476636 | 5.4590532 | 1.60E-109 | 4.88E-111 | up |
| VIT_04s0008g00300.t01 | 3165 | receptor-like protein kinase HSL1-like | 240 | 5.1206132 | 4 | 0.1148991 | 5.4778771 | 7.38E-50 | 4.86E-51 | up |
| VIT_12s0028g00680.t01 | 183 | Putative uncharacterized protein | 30 | 11.070178 | 0.5 | 0.2483987 | 5.4778771 | 3.48E-07 | 1.08E-07 | up |
| VIT_19s0093g00320.t01 | 885 | glutathione S-transferase | 722 | 55.09071 | 12 | 1.2327311 | 5.481879 | 4.04E-148 | 9.03E-150 | up |
| VIT_06s0004g07230.t01 | 1664 | UDP-glycosyltransferase 87A2-like | 736 | 29.868192 | 12 | 0.6556292 | 5.509586 | 4.79E-151 | 1.04E-152 | up |
| VIT_11s0016g05540.t01 | 1005 | blue copper protein | 308 | 20.695175 | 5 | 0.452308 | 5.515845 | 9.70E-64 | 4.99E-65 | up |
| VIT_04s0008g04000.t01 | 587 | dehydration-responsive protein RD22-like | 126 | 14.494956 | 2 | 0.3097578 | 5.5482664 | 1.09E-26 | 1.22E-27 | up |
| VIT_16s0050g02220.t01 | 1254 | Acidic endochitinase | 980 | 52.773146 | 15 | 1.0874871 | 5.6007339 | 4.15E-201 | 6.73E-203 | up |
| VIT_08s0007g01370.t01 | 1117 | non-specific lipid-transfer protein | 269 | 16.262359 | 4 | 0.3255646 | 5.6424489 | 7.08E-56 | 4.14E-57 | up |
| VIT_00s2536g00010.t01 | 425 | aspartic proteinase-like protein 1 | 34 | 5.4022469 | 0.5 | 0.1069576 | 5.6584494 | 5.20E-08 | 1.49E-08 | up |
| VIT_13s0019g02190.t01 | 105 | Putative uncharacterized protein | 34 | 21.866238 | 0.5 | 0.4329234 | 5.6584494 | 5.20E-08 | 1.49E-08 | up |
| VIT_16s0022g00440.t01 | 703 | dynein light chain-like | 206 | 19.787747 | 3 | 0.3879684 | 5.6725245 | 4.88E-43 | 3.68E-44 | up |
| VIT_18s0001g08710.t01 | 397 | basic leucine zipper 1 | 35 | 5.9533577 | 0.5 | 0.1145012 | 5.7002695 | 3.25E-08 | 9.14E-09 | up |
| VIT_16s0100g01190.t01 | 1521 | Chalcone and stilbene synthase | 282 | 12.52 | 4 | 0.2390899 | 5.7105379 | 1.49E-58 | 8.36E-60 | up |
| VIT_15s0021g02670.t01 | 869 | putative expansin-B2-like | 71 | 5.5172545 | 1 | 0.104619 | 5.7207336 | 1.57E-15 | 2.74E-16 | up |
| VIT_16s0039g01300.t01 | 2286 | phenylalanine ammonia-lyase | 502 | 14.829002 | 7 | 0.2783891 | 5.7351751 | 1.64E-103 | 5.31E-105 | up |
| VIT_01s0010g02450.t01 | 497 | Putative uncharacterized protein | 74 | 10.054484 | 1 | 0.1829254 | 5.7804399 | 3.85E-16 | 6.52E-17 | up |
| VIT_04s0023g02900.t01 | 1825 | ferulic acid 5-hydroxylase | 1383 | 51.173339 | 18 | 0.8966852 | 5.8346469 | 9.34E-284 | 1.12E-285 | up |
| VIT_18s0001g10600.t01 | 435 | Putative uncharacterized protein | 77 | 11.953247 | 1 | 0.2089975 | 5.837773 | 9.48E-17 | 1.56E-17 | up |
| VIT_12s0028g00920.t01 | 808 | glutathione S-transferase | 472 | 39.4471 | 6 | 0.6751033 | 5.8686671 | 2.36E-97 | 8.09E-99 | up |
| VIT_00s2508g00010.t01 | 1473 | phenylalanine ammonia-lyased | 159 | 7.2891824 | 2 | 0.1234405 | 5.8838695 | 1.90E-33 | 1.78E-34 | up |
| VIT_16s0100g01070.t01 | 1445 | Chalcone and stilbene synthase | 321 | 15.001049 | 4 | 0.2516648 | 5.897416 | 1.71E-66 | 8.51E-68 | up |
| VIT_05s0020g03360.t01 | 384 | Putative uncharacterized protein | 41 | 7.2100301 | 0.5 | 0.1183775 | 5.9285385 | 1.97E-09 | 5.06E-10 | up |
| VIT_15s0048g01200.t01 | 4663 | subtilisin-like protease | 670 | 9.7027274 | 8 | 0.155975 | 5.9590038 | 1.02E-137 | 2.42E-139 | up |
| VIT_02s0025g05030.t01 | 300 | Putative uncharacterized protein | 42 | 9.4539321 | 0.5 | 0.1515232 | 5.9633039 | 1.24E-09 | 3.15E-10 | up |
| VIT_18s0166g00070.t01 | 450 | Putative uncharacterized protein | 42 | 6.3026214 | 0.5 | 0.1010155 | 5.9633039 | 1.24E-09 | 3.15E-10 | up |
| VIT_05s0062g01010.t01 | 1116 | aldo-keto reductase 2 | 85 | 5.1432682 | 1 | 0.0814641 | 5.9803774 | 2.33E-18 | 3.58E-19 | up |
| VIT_16s0100g00750.t01 | 1361 | Chalcone and stilbene synthase | 434 | 21.533571 | 5 | 0.3339968 | 6.0106096 | 1.82E-89 | 6.81E-91 | up |
| VIT_07s0104g01020.t01 | 585 | aldo-keto reductase-like | 44 | 5.0790356 | 0.5 | 0.0777042 | 6.0304181 | 4.98E-10 | 1.22E-10 | up |
| VIT_06s0004g01020.t01 | 1652 | dirigent protein 7 | 1598 | 65.320752 | 18 | 0.9905875 | 6.0431132 | 0 | 0 | up |
| VIT_06s0061g00120.t01 | 1218 | Glucan endo-1,3-beta-glucosidase | 3659 | 202.86147 | 41 | 3.0603207 | 6.0506682 | 0 | 0 | up |
| VIT_18s0001g10760.t01 | 511 | Putative uncharacterized protein [Source:UniProtKB/TrEMBL;Acc:F6H006] | 45 | 5.9467004 | 0.5 | 0.0889569 | 6.0628396 | 3.16E-10 | 7.63E-11 | up |
| VIT_05s0049g00690.t01 | 528 | Putative uncharacterized protein [Source:UniProtKB/TrEMBL;Acc:F6H1E4] | 1367 | 174.83124 | 15 | 2.5827818 | 6.0808934 | 9.89E-280 | 1.19E-281 | up |
| VIT_18s0001g11180.t01 | 432 | Putative uncharacterized protein | 46 | 7.1904907 | 0.5 | 0.1052244 | 6.0945485 | 2.01E-10 | 4.78E-11 | up |
| VIT_05s0077g01550.t01 | 480 | Pathogenesis-related protein 10 | 2138 | 300.78135 | 23 | 4.3562919 | 6.1094707 | 0 | 0 | up |
| VIT_07s0129g01010.t01 | 413 | Putative uncharacterized protein | 186 | 30.412165 | 2 | 0.4402611 | 6.1101453 | 7.64E-39 | 6.31E-40 | up |
| VIT_12s0034g01870.t01 | 1391 | glutelin type-A 3 | 374 | 18.156365 | 4 | 0.2614347 | 6.117881 | 4.39E-77 | 1.88E-78 | up |
| VIT_16s0039g02370.t01 | 360 | leucine-rich repeat receptor-like protein kinase | 94 | 17.632334 | 1 | 0.2525387 | 6.1255754 | 3.82E-20 | 5.45E-21 | up |
| VIT_06s0004g01030.t01 | 498 | dirigent protein 22 | 95 | 12.881864 | 1 | 0.1825581 | 6.1408421 | 2.43E-20 | 3.44E-21 | up |
| VIT_16s0100g01200.t01 | 1433 | Chalcone and stilbene synthase | 588 | 27.708664 | 6 | 0.3806584 | 6.1856964 | 1.66E-120 | 4.60E-122 | up |
| VIT_18s0001g01550.t01 | 335 | tetratricopeptide repeat protein 33 | 50 | 10.078819 | 0.5 | 0.1356924 | 6.2148427 | 3.31E-11 | 7.45E-12 | up |
| VIT_07s0005g03340.t01 | 1141 | R2R3 Myb14 transcription factor (MYB14) | 312 | 18.465174 | 3 | 0.2390375 | 6.2714262 | 3.12E-64 | 1.60E-65 | up |
| VIT_18s0001g08660.t01 | 607 | Putative uncharacterized protein | 52 | 5.7849432 | 0.5 | 0.0748879 | 6.2714262 | 1.35E-11 | 2.97E-12 | up |
| VIT_08s0007g01400.t01 | 1009 | glutathione S-transferase | 212 | 14.18826 | 2 | 0.180206 | 6.298907 | 6.17E-44 | 4.59E-45 | up |
| VIT_16s0100g01120.t01 | 1367 | Chalcone and stilbene synthase | 213 | 10.521933 | 2 | 0.1330123 | 6.3056961 | 3.96E-44 | 2.92E-45 | up |
| VIT_16s0100g01160.t01 | 1285 | Chalcone and stilbene synthase | 320 | 16.816333 | 3 | 0.2122504 | 6.3079521 | 8.64E-66 | 4.35E-67 | up |
| VIT_06s0004g02540.t01 | 693 | kiwellin-like | 54 | 5.2619288 | 0.5 | 0.0655945 | 6.325874 | 5.59E-12 | 1.19E-12 | up |
| VIT_19s0014g02010.t01 | 585 | UDP-glycosyltransferase 79B9-like | 56 | 6.4642271 | 0.5 | 0.0777042 | 6.3783414 | 2.31E-12 | 4.79E-13 | up |
| VIT_05s0020g05040.t01 | 394 | protease inhibitor | 56 | 9.5979006 | 0.5 | 0.115373 | 6.3783414 | 2.31E-12 | 4.79E-13 | up |
| VIT_07s0005g00870.t01 | 1218 | protein EXORDIUM-like 2 | 257 | 14.248537 | 2 | 0.1492839 | 6.5766111 | 1.58E-52 | 9.85E-54 | up |
| VIT_05s0049g01070.t01 | 752 | glutathione S-transferase= | 386 | 34.662023 | 3 | 0.3626885 | 6.578481 | 2.13E-78 | 8.95E-80 | up |
| VIT_06s0061g00100.t01 | 1391 | Beta-1,3-glucanase | 2859 | 138.79425 | 22 | 1.4378909 | 6.5928498 | 0 | 0 | up |
| VIT_04s0008g07250.t01 | 864 | ASPARTIC PROTEASE IN GUARD CELL 2-like | 65 | 5.080238 | 0.5 | 0.0526122 | 6.5933543 | 4.56E-14 | 8.59E-15 | up |
| VIT_16s0050g01390.t01 | 582 | Putative uncharacterized protein [Source:UniProtKB/TrEMBL;Acc:F6H6B2] | 66 | 7.6578242 | 0.5 | 0.0781047 | 6.6153806 | 2.96E-14 | 5.53E-15 | up |
| VIT_05s0049g00620.t01 | 405 | proline-rich 33 kDa extensin-related protein-like | 404 | 67.36135 | 3 | 0.6734364 | 6.6442355 | 8.76E-82 | 3.60E-83 | up |
| VIT_13s0019g03520.t01 | 397 | Putative uncharacterized protein | 680 | 115.66524 | 5 | 1.1450116 | 6.6584494 | 5.98E-137 | 1.43E-138 | up |
| VIT_09s0002g02970.t01 | 450 | Putative uncharacterized protein | 820 | 123.05118 | 6 | 1.2121856 | 6.6655041 | 6.27E-165 | 1.25E-166 | up |
| VIT_05s0049g00800.t01 | 1143 | early nodulin-75-like | 1677 | 99.076641 | 12 | 0.9544768 | 6.697691 | 0 | 0 | up |
| VIT_05s0049g01100.t01 | 823 | glutathione S-transferase | 858 | 70.399876 | 6 | 0.6627989 | 6.7308578 | 5.38E-172 | 1.02E-173 | up |
| VIT_17s0000g00640.t01 | 370 | Putative uncharacterized protein | 72 | 13.140601 | 0.5 | 0.1228566 | 6.7409115 | 2.28E-15 | 4.03E-16 | up |
| VIT_02s0025g04310.t01 | 986 | thaumatin-like protein | 45151 | 3092.2522 | 313 | 28.860098 | 6.743438 | 0 | 0 | up |
| VIT_19s0090g01410.t01 | 939 | wax synthase (WS-1) | 73 | 5.2497873 | 0.5 | 0.04841 | 6.7608111 | 1.50E-15 | 2.62E-16 | up |
| VIT_10s0003g01810.t01 | 398 | Putative uncharacterized protein [Source:UniProtKB/TrEMBL;Acc:D7TJR1] | 74 | 12.555473 | 0.5 | 0.1142135 | 6.7804399 | 9.81E-16 | 1.70E-16 | up |
| VIT_09s0002g04930.t01 | 548 | F-box/kelch-repeat protein | 74 | 9.1187562 | 0.5 | 0.0829507 | 6.7804399 | 9.81E-16 | 1.70E-16 | up |
| VIT_17s0000g00680.t01 | 546 | pyruvate dehydrogenase E1 component subunit beta-1 | 74 | 9.1521582 | 0.5 | 0.0832545 | 6.7804399 | 9.81E-16 | 1.70E-16 | up |
| VIT_03s0063g01560.t01 | 1854 | cytochrome P450 82C4-like | 298 | 10.854029 | 2 | 0.0980733 | 6.790155 | 3.79E-60 | 2.07E-61 | up |
| VIT_12s0059g01720.t01 | 455 | Putative uncharacterized protein | 151 | 22.41042 | 1 | 0.1998108 | 6.8093912 | 5.98E-31 | 5.98E-32 | up |
| VIT_05s0020g03680.t01 | 728 | Putative uncharacterized protein | 152 | 14.099271 | 1 | 0.1248818 | 6.818914 | 3.92E-31 | 3.91E-32 | up |
| VIT_17s0000g05580.t01 | 830 | momilactone A synthase-like | 152 | 12.366589 | 1 | 0.1095348 | 6.818914 | 3.92E-31 | 3.91E-32 | up |
| VIT_18s0001g04280.t01 | 1939 | terpene synthase, putative, expressed | 157 | 5.4677203 | 1 | 0.046887 | 6.8656073 | 4.82E-32 | 4.68E-33 | up |
| VIT_16s0100g00990.t01 | 1544 | Chalcone and stilbene synthase | 158 | 6.9102576 | 1 | 0.0588821 | 6.8747673 | 3.17E-32 | 3.06E-33 | up |
| VIT_05s0049g00810.t01 | 827 | proline-rich 33 kDa extensin-related protein-like | 82 | 6.6956507 | 0.5 | 0.0549661 | 6.9285385 | 3.46E-17 | 5.60E-18 | up |
| VIT_05s0020g02200.t01 | 2971 | Putative uncharacterized protein | 332 | 7.5460534 | 2 | 0.0612009 | 6.9460259 | 2.45E-66 | 1.23E-67 | up |
| VIT_12s0059g02590.t01 | 780 | cysteine-rich repeat secretory protein 38-like | 85 | 7.3588299 | 0.5 | 0.0582782 | 6.9803774 | 1.00E-17 | 1.58E-18 | up |
| VIT_01s0011g02570.t01 | 204 | Putative uncharacterized protein | 88 | 29.129763 | 0.5 | 0.2228282 | 7.0304181 | 2.92E-18 | 4.51E-19 | up |
| VIT_05s0020g03300.t01 | 1194 | G-type lectin S-receptor-like serine/threonine-protein | 90 | 5.0900568 | 0.5 | 0.0380712 | 7.0628396 | 1.29E-18 | 1.96E-19 | up |
| VIT_08s0007g08750.t01 | 1150 | heat stress transcription factor B-3 | 90 | 5.2848068 | 0.5 | 0.0395278 | 7.0628396 | 1.29E-18 | 1.96E-19 | up |
| VIT_05s0077g01570.t01 | 1159 | major allergen Pru ar 1-like | 6370 | 371.14229 | 35 | 2.7454591 | 7.0787811 | 0 | 0 | up |
| VIT_15s0021g02700.t01 | 1148 | putative expansin-B2 | 92 | 5.4116585 | 0.5 | 0.0395967 | 7.0945485 | 5.73E-19 | 8.57E-20 | up |
| VIT_04s0008g05360.t01 | 1039 | carboxylesterase 17 | 92 | 5.9793878 | 0.5 | 0.0437507 | 7.0945485 | 5.73E-19 | 8.57E-20 | up |
| VIT_04s0008g05390.t01 | 933 | serine hydrolase-like (BIG8.1) | 376 | 27.213891 | 2 | 0.1948851 | 7.1255754 | 3.53E-74 | 1.59E-75 | up |
| VIT_05s0077g01530.t01 | 717 | major allergen Pru av 1 | 3130 | 294.78788 | 16 | 2.0287625 | 7.1829335 | 0 | 0 | up |
| VIT_05s0049g00680.t01 | 709 | proline-rich 33 kDa extensin-related protein-like | 589 | 56.098791 | 3 | 0.3846851 | 7.1881478 | 3.32E-115 | 9.67E-117 | up |
| VIT_00s2485g00010.t01 | 1318 | cysteine-rich receptor-like protein kinase 10 | 99 | 5.0722918 | 0.5 | 0.0344893 | 7.2003431 | 3.40E-20 | 4.84E-21 | up |
| VIT_17s0000g06130.t01 | 782 | glutathione S-transferase | 100 | 8.6353052 | 0.5 | 0.0581291 | 7.2148427 | 2.28E-20 | 3.22E-21 | up |
| VIT_02s0025g04300.t01 | 855 | thaumatin-like protein | 31609 | 2496.4857 | 157 | 16.694135 | 7.2244135 | 0 | 0 | up |
| VIT_05s0020g05000.t01 | 456 | protease inhibitor | 104 | 15.401143 | 0.5 | 0.0996863 | 7.2714262 | 4.65E-21 | 6.38E-22 | up |
| VIT_04s0008g04060.t01 | 1141 | BURP domain-containing protein 3-like | 104 | 6.1550578 | 0.5 | 0.0398396 | 7.2714262 | 4.65E-21 | 6.38E-22 | up |

| | | | | | | | | | | |
|---|---|---|---|---|---|---|---|---|---|---|
| VIT_05s0077g01600.t01 | 588 | Pathogenesis-related protein 10 | 105 | 12.058587 | 0.5 | 0.0773078 | 7.285232 | 3.13E-21 | 4.26E-22 | up |
| VIT_01s0127g00070.t01 | 1563 | high affinity nitrate transporter 2.5 | 219 | 9.4617089 | 1 | 0.0581663 | 7.3457736 | 5.77E-43 | 4.36E-44 | up |
| VIT_08s0056g00630.t01 | 871 | acid phosphatase 1 | 111 | 8.6057607 | 0.5 | 0.0521894 | 7.3654024 | 2.93E-22 | 3.86E-23 | up |
| VIT_05s0077g01560.t01 | 668 | Pathogenesis-related protein 10 | 4443 | 449.14265 | 20 | 2.7219736 | 7.3663768 | 0 | 0 | up |
| VIT_00s0262g00090.t01 | 1272 | putative receptor-like protein kinase | 120 | 6.3705742 | 0.5 | 0.0357366 | 7.4778771 | 8.91E-24 | 1.10E-24 | up |
| VIT_05s0094g00320.t01 | 822 | chitinase 4-like | 121 | 9.9402658 | 0.5 | 0.0553004 | 7.4898497 | 6.05E-24 | 7.44E-25 | up |
| VIT_05s0094g00340.t01 | 847 | Class IV chitinase | 25229 | 2011.4122 | 103 | 11.055648 | 7.5072814 | 0 | 0 | up |
| VIT_16s0100g01150.t01 | 1377 | Chalcone and stilbene synthase | 123 | 6.0319206 | 0.5 | 0.0330116 | 7.513501 | 2.80E-24 | 3.41E-25 | up |
| VIT_02s0025g04340.t01 | 969 | thaumatin-like protein | 5915 | 412.20705 | 24 | 2.2517379 | 7.5161865 | 0 | 0 | up |
| VIT_11s0016g03190.t01 | 769 | E3 ubiquitin-protein ligase RNF181-like | 124 | 10.888794 | 0.5 | 0.0591118 | 7.5251828 | 1.91E-24 | 2.31E-25 | up |
| VIT_05s0049g01080.t01 | 720 | glutathione S-transferase | 271 | 25.416821 | 1 | 0.1262693 | 7.6531356 | 1.14E-51 | 7.27E-53 | up |
| VIT_17s0119g00280.t01 | 715 | miraculin | 554 | 52.322461 | 2 | 0.2543047 | 7.6847287 | 2.39E-104 | 7.65E-106 | up |
| VIT_16s0100g01170.t01 | 1340 | Chalcone and stilbene synthase | 140 | 7.0551732 | 0.5 | 0.0339231 | 7.7002695 | 4.43E-27 | 4.93E-28 | up |
| VIT_06s0009g02590.t01 | 1645 | Pectinesterase | 140 | 5.7470712 | 0.5 | 0.0276334 | 7.7002695 | 4.43E-27 | 4.93E-28 | up |
| VIT_12s0059g02510.t01 | 639 | B-box zinc finger protein 32 | 141 | 14.900564 | 0.5 | 0.0711377 | 7.7105379 | 3.05E-27 | 3.38E-28 | up |
| VIT_14s0128g00540.t01 | 834 | germin-like protein 7 (GER7) | 304 | 24.614554 | 1 | 0.1090095 | 7.818914 | 5.17E-57 | 2.98E-58 | up |
| VIT_05s0049g00780.t01 | 814 | early nodulin-75-like | 2590 | 214.86209 | 8 | 0.8935029 | 7.9097229 | 0 | 0 | up |
| VIT_18s0001g11480.t01 | 1714 | cytochrome P450 82A1-like | 162 | 6.3824679 | 0.5 | 0.026521 | 7.9108365 | 1.32E-30 | 1.33E-31 | up |
| VIT_06s0004g02010.t01 | 313 | extensin-like | 162 | 34.950639 | 0.5 | 0.1452299 | 7.9108365 | 1.32E-30 | 1.33E-31 | up |
| VIT_12s0035g00610.t01 | 1588 | cytochrome P450 CYP82D47 | 163 | 6.9314094 | 0.5 | 0.0286253 | 7.9197147 | 9.19E-31 | 9.23E-32 | up |
| VIT_16s0100g00900.t01 | 1925 | Chalcone and stilbene synthase | 169 | 5.9284398 | 0.5 | 0.023614 | 7.9718659 | 1.05E-31 | 1.03E-32 | up |
| VIT_08s0105g00200.t01 | 1416 | E3 ubiquitin-protein ligase PUB23 | 173 | 8.2502535 | 0.5 | 0.0321024 | 8.0056147 | 2.49E-32 | 2.40E-33 | up |
| VIT_18s0001g11500.t01 | 1601 | cytochrome P450 82A1-like | 173 | 7.2969138 | 0.5 | 0.0283929 | 8.0056147 | 2.49E-32 | 2.40E-33 | up |
| VIT_13s0019g02180.t01 | 1097 | tropinone reductase | 1049 | 64.573348 | 3 | 0.2486251 | 8.020823 | 6.91E-191 | 1.21E-192 | up |
| VIT_05s0049g00840.t01 | 1017 | early nodulin-75-like | 2879 | 191.16358 | 8 | 0.7151537 | 8.0623386 | 0 | 0 | up |
| VIT_08s0007g01090.t01 | 1851 | diacylglycerol kinase theta-like | 188 | 6.8586063 | 0.5 | 0.0245581 | 8.1255754 | 1.21E-34 | 1.10E-35 | up |
| VIT_05s0077g01690.t01 | 631 | pathogenesis-related protein 10.8 (PR10.8) | 189 | 20.226321 | 0.5 | 0.0720396 | 8.1332289 | 8.49E-35 | 7.70E-36 | up |
| VIT_14s0128g00570.t01 | 871 | germin-like protein 7 (GER7) | 2285 | 177.15462 | 6 | 0.6262727 | 8.1440025 | 0 | 0 | up |
| VIT_00s2745g00010.t01 | 1153 | receptor-like protein kinase | 194 | 11.362054 | 0.5 | 0.0394249 | 8.1708994 | 1.46E-35 | 1.31E-36 | up |
| VIT_16s0100g01140.t01 | 1428 | Chalcone and stilbene synthase | 198 | 9.363138 | 0.5 | 0.0318326 | 8.2003431 | 3.63E-36 | 3.20E-37 | up |
| VIT_05s0094g00330.t01 | 1454 | chitinase 5-like | 7499 | 348.27587 | 18 | 1.1254818 | 8.273544 | 0 | 0 | up |
| VIT_03s0088g00710.t01 | 671 | pathogenesis-related protein PR-1 | 840 | 84.535906 | 2 | 0.2709804 | 8.285232 | 5.51E-149 | 1.22E-150 | up |
| VIT_11s0016g05010.t01 | 1453 | metallothiol transferase fosB-like | 420 | 19.519474 | 1 | 0.0625698 | 8.285232 | 6.18E-75 | 2.73E-76 | up |
| VIT_09s0054g01080.t01 | 2104 | polygalacturonase QRT3-like | 211 | 6.7720657 | 0.5 | 0.021605 | 8.2920857 | 4.07E-38 | 3.42E-39 | up |
| VIT_08s0040g01520.t01 | 1897 | scopoletin glucosyltransferase | 211 | 7.5110312 | 0.5 | 0.0239626 | 8.2920857 | 4.07E-38 | 3.42E-39 | up |
| VIT_00s1455g00010.t01 | 1018 | expansin-like B1 | 228 | 15.124169 | 0.5 | 0.0446532 | 8.4038765 | 1.26E-40 | 9.93E-42 | up |
| VIT_09s0002g02950.t01 | 284 | inositol oxygenase 1 | 231 | 54.926014 | 0.5 | 0.1600597 | 8.4227356 | 4.57E-41 | 3.58E-42 | up |
| VIT_18s0117g00550.t01 | 1961 | laccase 15 | 233 | 8.0234799 | 0.5 | 0.0231805 | 8.4351727 | 2.33E-41 | 1.82E-42 | up |
| VIT_05s0077g01540.t01 | 480 | major allergen Pru av 1-like | 476 | 66.965352 | 1 | 0.189404 | 8.4658043 | 3.44E-83 | 1.38E-84 | up |
| VIT_16s0100g01030.t01 | 1395 | Chalcone and stilbene synthase | 483 | 23.380692 | 1 | 0.0651713 | 8.4868659 | 3.33E-84 | 1.32E-85 | up |
| VIT_00s2849g00010.t01 | 1017 | phenylalanine ammonia-lyase | 252 | 16.732623 | 0.5 | 0.0446971 | 8.5482664 | 4.17E-44 | 3.08E-45 | up |
| VIT_08s0105g00380.t01 | 1350 | flavonol synthase/flavanone 3-hydroxylase | 505 | 25.260506 | 1 | 0.0673436 | 8.5511261 | 2.24E-87 | 8.59E-89 | up |
| VIT_16s0100g00830.t01 | 1468 | Chalcone and stilbene synthase | 560 | 25.760033 | 1 | 0.0619305 | 8.7002695 | 3.64E-95 | 1.28E-96 | up |
| VIT_08s0040g00920.t01 | 789 | glutathione S-transferase | 280 | 23.96434 | 0.5 | 0.0576134 | 8.7002695 | 4.57E-48 | 3.12E-49 | up |
| VIT_16s0039g01360.t01 | 2187 | phenylalanine ammonia-lyase | 281 | 8.6764482 | 0.5 | 0.0207851 | 8.7054128 | 3.32E-48 | 2.25E-49 | up |
| VIT_16s0100g00770.t01 | 1509 | Chalcone and stilbene synthase | 284 | 12.709063 | 0.5 | 0.0301239 | 8.7207336 | 1.27E-48 | 8.53E-50 | up |
| VIT_00s0270g00120.t01 | 703 | alpha-amylase/subtilisin inhibitor-like | 1170 | 112.38672 | 2 | 0.2586456 | 8.7632793 | 1.85E-196 | 3.12E-198 | up |
| VIT_16s0100g00780.t01 | 1390 | Chalcone and stilbene synthase | 305 | 14.817314 | 0.5 | 0.0327028 | 8.8236519 | 1.59E-51 | 1.01E-52 | up |
| VIT_17s0000g03160.t01 | 474 | Putative uncharacterized protein | 323 | 46.015975 | 0.5 | 0.0959008 | 8.9063769 | 5.72E-54 | 3.45E-55 | up |
| VIT_05s0049g00770.t01 | 1393 | early nodulin-75-like | 2626 | 127.2999 | 4 | 0.2610593 | 8.9296377 | 0 | 0 | up |
| VIT_17s0119g00150.t01 | 691 | miraculin | 367 | 35.865134 | 0.5 | 0.0657843 | 9.0906228 | 7.97E-60 | 4.37E-61 | up |
| VIT_12s0059g02600.t01 | 1376 | cysteine-rich repeat secretory protein 38-like | 385 | 18.894123 | 0.5 | 0.0330356 | 9.1597011 | 3.61E-62 | 1.90E-63 | up |
| VIT_14s0128g00600.t01 | 847 | germin-like protein 7 (GER7) | 387 | 30.854037 | 0.5 | 0.0536682 | 9.1671763 | 1.98E-62 | 1.04E-63 | up |
| VIT_05s0020g03280.t01 | 2030 | Amine oxidase | 387 | 12.873581 | 0.5 | 0.0223926 | 9.1671763 | 1.98E-62 | 1.04E-63 | up |
| VIT_16s0100g00910.t01 | 1401 | Chalcone and stilbene synthase | 411 | 19.810167 | 0.5 | 0.0324461 | 9.2539811 | 1.63E-65 | 8.24E-67 | up |
| VIT_00s0194g00180.t01 | 637 | rho GTPase-activating protein gacV-like | 427 | 45.26608 | 0.5 | 0.071361 | 9.3090788 | 1.52E-67 | 7.46E-69 | up |
| VIT_16s0100g01100.t01 | 1475 | Chalcone and stilbene synthase | 442 | 20.235535 | 0.5 | 0.0308183 | 9.3588891 | 1.96E-69 | 9.44E-71 | up |
| VIT_00s0780g00030.t01 | 242 | Putative uncharacterized protein | 2741 | 764.85324 | 3 | 1.127032 | 9.4065106 | 0 | 0 | up |
| VIT_02s0025g04290.t01 | 722 | thaumatin-like protein | 471 | 44.052256 | 0.5 | 0.0629598 | 9.4505698 | 4.88E-73 | 2.24E-74 | up |
| VIT_07s0141g00930.t01 | 908 | cysteine-rich repeat secretory protein 55 | 472 | 35.102706 | 0.5 | 0.0500627 | 9.4536296 | 3.69E-73 | 1.69E-74 | up |
| VIT_10s0003g00480.t01 | 1629 | trans-resveratrol di-O-methyltransferase | 489 | 20.270862 | 0.5 | 0.0279048 | 9.5046772 | 3.08E-75 | 1.35E-76 | up |
| VIT_03s0088g00890.t01 | 489 | pathogenesis-related protein PR-1 | 720 | 99.427857 | 0.5 | 0.092959 | 10.06284 | 5.17E-102 | 1.71E-103 | up |
| VIT_09s0002g01320.t01 | 874 | Germin-like protein | 724 | 55.938598 | 0.5 | 0.0520103 | 10.070832 | 1.87E-102 | 6.17E-104 | up |

Table S3 Transcripts expressed only in the Cu-treated or control group.

| #Gene | Length | Function | Cu-treated | RPKM_A | control | RPKM_B | log2(Fold_c | q-value(Ber | p_value | Result |
|---|---|---|---|---|---|---|---|---|---|---|
| expressed only in the Cu-treated group | | | | | | | | | | |
| VIT_02s0087g00850.t01 | 162 | Putative uncharacterized protein [Source:UniProtKB/TrEMBL;Acc: | 14 | 5.8357606 | 0.5 | 0.2805985 | 4.3783414 | 0.0009429 | 0.0004484 | up |
| VIT_00s0173g00230.t01 | 141 | Putative uncharacterized protein [Source:UniProtKB/TrEMBL;Acc: | 15 | 7.183839 | 0.5 | 0.3223898 | 4.4778771 | 0.0005664 | 0.0002601 | up |
| VIT_04s0008g02730.t01 | 198 | Putative uncharacterized protein [Source:UniProtKB/TrEMBL;Acc: | 16 | 5.4568151 | 0.5 | 0.2295806 | 4.5709865 | 0.0003416 | 0.0001515 | up |
| VIT_07s0205g00060.t01 | 228 | tRNA-dihydrouridine(20) synthase | 17 | 5.0349889 | 0.5 | 0.1993726 | 4.6584494 | 0.0002067 | 8.85E-05 | up |
| VIT_00s0125g00230.t01 | 214 | LRR receptor-like serine/threonine-protein kinase | 17 | 5.3643807 | 0.5 | 0.2124157 | 4.6584494 | 0.0002067 | 8.85E-05 | up |
| VIT_07s0031g02710.t01 | 191 | fatty acid amide hydrolase | 17 | 6.0103532 | 0.5 | 0.2379945 | 4.6584494 | 0.0002067 | 8.85E-05 | up |
| VIT_09s0018g00510.t01 | 75 | receptor-like protein kinase | 17 | 15.306366 | 0.5 | 0.6060928 | 4.6584494 | 0.0002067 | 8.85E-05 | up |
| VIT_12s0035g00460.t01 | 123 | isease resistance protein RGA1 | 18 | 9.882159 | 0.5 | 0.3695688 | 4.7409115 | 0.000125 | 5.19E-05 | up |
| VIT_04s0023g03090.t01 | 206 | polyprenol reductase 2 | 20 | 6.5561249 | 0.5 | 0.2206648 | 4.8929146 | 4.58E-05 | 1.80E-05 | up |
| VIT_17s0000g07770.t01 | 209 | snakin-2-like | 21 | 6.7851187 | 0.5 | 0.2174974 | 4.9633039 | 2.79E-05 | 1.07E-05 | up |
| VIT_02s0087g00890.t01 | 165 | WAT1-related protein | 23 | 9.413006 | 0.5 | 0.2754967 | 5.0945485 | 1.04E-05 | 3.77E-06 | up |
| VIT_00s0179g00250.t01 | 222 | calcium-transporting ATPase 12, plasma membrane-type | 26 | 7.9086948 | 0.5 | 0.2047611 | 5.2714262 | 2.39E-06 | 8.10E-07 | up |
| VIT_00s0467g00020.t01 | 304 | disease resistance RPP13-like protein 1 | 26 | 5.7754284 | 0.5 | 0.1495295 | 5.2714262 | 2.39E-06 | 8.10E-07 | up |
| VIT_01s0146g00320.t01 | 256 | Putative uncharacterized protein [Source:UniProtKB/TrEMBL;Acc: | 28 | 7.3858845 | 0.5 | 0.1775662 | 5.3783414 | 9.08E-07 | 2.94E-07 | up |
| VIT_04s0023g00530.t01 | 370 | auxin-induced protein 15A | 29 | 5.2927419 | 0.5 | 0.1228566 | 5.4289675 | 5.61E-07 | 1.78E-07 | up |
| VIT_05s0029g00320.t01 | 197 | Putative uncharacterized protein [Source:UniProtKB/TrEMBL;Acc: | 29 | 9.9406828 | 0.5 | 0.230746 | 5.4289675 | 5.61E-07 | 1.78E-07 | up |
| VIT_12s0028g00680.t01 | 183 | Putative uncharacterized protein [Source:UniProtKB/TrEMBL;Acc: | 30 | 11.070178 | 0.5 | 0.2483987 | 5.4778771 | 3.48E-07 | 1.08E-07 | up |
| VIT_00s2536g00010.t01 | 425 | aspartic proteinase-like protein 1 | 34 | 5.4022469 | 0.5 | 0.1069576 | 5.6584494 | 5.20E-08 | 1.49E-08 | up |
| VIT_13s0019g02190.t01 | 105 | Putative uncharacterized protein [Source:UniProtKB/TrEMBL;Acc: | 34 | 21.866238 | 0.5 | 0.4329234 | 5.6584494 | 5.20E-08 | 1.49E-08 | up |
| VIT_18s0001g08710.t01 | 397 | basic leucine zipper 1 | 35 | 5.9533577 | 0.5 | 0.1145012 | 5.7002695 | 3.25E-08 | 9.14E-09 | up |
| VIT_05s0020g03360.t01 | 384 | Putative uncharacterized protein [Source:UniProtKB/TrEMBL;Acc: | 41 | 7.2100301 | 0.5 | 0.1183775 | 5.9285385 | 1.97E-09 | 5.06E-10 | up |
| VIT_02s0025g05030.t01 | 300 | Putative uncharacterized protein [Source:UniProtKB/TrEMBL;Acc: | 42 | 9.4539321 | 0.5 | 0.1515232 | 5.9633039 | 1.24E-09 | 3.15E-10 | up |
| VIT_18s0166g00070.t01 | 450 | Putative uncharacterized protein [Source:UniProtKB/TrEMBL;Acc: | 42 | 6.3026214 | 0.5 | 0.1010155 | 5.9633039 | 1.24E-09 | 3.15E-10 | up |
| VIT_07s0104g01020.t01 | 585 | aldo-keto reductase-like | 44 | 5.0790356 | 0.5 | 0.0777042 | 6.0304181 | 4.98E-10 | 1.22E-10 | up |
| VIT_18s0001g10760.t01 | 511 | Putative uncharacterized protein [Source:UniProtKB/TrEMBL;Acc: | 45 | 5.9467004 | 0.5 | 0.0889569 | 6.0628396 | 3.16E-10 | 7.63E-11 | up |
| VIT_18s0001g11180.t01 | 432 | Putative uncharacterized protein [Source:UniProtKB/TrEMBL;Acc: | 46 | 7.1904907 | 0.5 | 0.1052244 | 6.0945485 | 2.01E-10 | 4.78E-11 | up |
| VIT_18s0001g01550.t01 | 335 | tetratricopeptide repeat protein 33 | 50 | 10.078819 | 0.5 | 0.1356924 | 6.2148427 | 3.31E-11 | 7.45E-12 | up |
| VIT_18s0001g08660.t01 | 607 | Putative uncharacterized protein [Source:UniProtKB/TrEMBL;Acc: | 52 | 5.7849432 | 0.5 | 0.0748879 | 6.2714262 | 1.35E-11 | 2.97E-12 | up |
| VIT_06s0004g02540.t01 | 693 | kiwellin-like | 54 | 5.2619288 | 0.5 | 0.0655945 | 6.325874 | 5.59E-12 | 1.19E-12 | up |
| VIT_19s0014g02010.t01 | 585 | UDP-glycosyltransferase 79B9-like | 56 | 6.4642271 | 0.5 | 0.0777042 | 6.3783414 | 2.31E-12 | 4.79E-13 | up |
| VIT_05s0020g05040.t01 | 394 | protease inhibitor | 56 | 9.5979006 | 0.5 | 0.115373 | 6.3783414 | 2.31E-12 | 4.79E-13 | up |
| VIT_04s0008g07250.t01 | 864 | ASPARTIC PROTEASE IN GUARD CELL 2-like | 65 | 5.080238 | 0.5 | 0.0526122 | 6.5933543 | 4.56E-14 | 8.59E-15 | up |
| VIT_16s0050g01390.t01 | 582 | Putative uncharacterized protein [Source:UniProtKB/TrEMBL;Acc: | 66 | 7.6578242 | 0.5 | 0.0781047 | 6.6153806 | 2.96E-14 | 5.53E-15 | up |
| VIT_17s0000g00640.t01 | 370 | Putative uncharacterized protein [Source:UniProtKB/TrEMBL;Acc: | 72 | 13.140601 | 0.5 | 0.1228566 | 6.7409115 | 2.28E-15 | 4.03E-16 | up |
| VIT_19s0090g01410.t01 | 939 | wax synthase (WS-1) | 73 | 5.2497873 | 0.5 | 0.04841 | 6.7608111 | 1.50E-15 | 2.62E-16 | up |
| VIT_10s0003g01810.t01 | 398 | Putative uncharacterized protein [Source:UniProtKB/TrEMBL;Acc: | 74 | 12.555473 | 0.5 | 0.1142135 | 6.7804399 | 9.81E-16 | 1.70E-16 | up |
| VIT_09s0002g04930.t01 | 548 | F-box/kelch-repeat protein | 74 | 9.1187562 | 0.5 | 0.0829507 | 6.7804399 | 9.81E-16 | 1.70E-16 | up |
| VIT_17s0000g00680.t01 | 546 | pyruvate dehydrogenase E1 component subunit beta-1 | 74 | 9.1521582 | 0.5 | 0.0832545 | 6.7804399 | 9.81E-16 | 1.70E-16 | up |
| VIT_05s0049g00810.t01 | 827 | proline-rich 33 kDa extensin-related protein-like | 82 | 6.6956507 | 0.5 | 0.0549661 | 6.9285385 | 3.46E-17 | 5.60E-18 | up |
| VIT_12s0059g02590.t01 | 780 | cysteine-rich repeat secretory protein 38-like | 85 | 7.3588299 | 0.5 | 0.0582782 | 6.9803774 | 1.00E-17 | 1.58E-18 | up |
| VIT_01s0011g02570.t01 | 204 | Putative uncharacterized protein [Source:UniProtKB/TrEMBL;Acc: | 88 | 29.129763 | 0.5 | 0.2228282 | 7.0304181 | 2.92E-18 | 4.51E-19 | up |
| VIT_05s0020g03300.t01 | 1194 | G-type lectin S-receptor-like serine/threonine-protein | 90 | 5.0900568 | 0.5 | 0.0380712 | 7.0628396 | 1.29E-18 | 1.96E-19 | up |
| VIT_08s0007g08750.t01 | 1150 | heat stress transcription factor B-3 | 90 | 5.2848068 | 0.5 | 0.0395278 | 7.0628396 | 1.29E-18 | 1.96E-19 | up |
| VIT_15s0021g02700.t01 | 1148 | putative expansin-B2 | 92 | 5.4116585 | 0.5 | 0.0395967 | 7.0945485 | 5.73E-19 | 8.57E-20 | up |
| VIT_04s0008g05360.t01 | 1039 | carboxylesterase 17 | 92 | 5.9793878 | 0.5 | 0.0437507 | 7.0945485 | 5.73E-19 | 8.57E-20 | up |
| VIT_00s2485g00010.t01 | 1318 | cysteine-rich receptor-like protein kinase 10 | 99 | 5.0722918 | 0.5 | 0.0344893 | 7.2003431 | 3.40E-20 | 4.84E-21 | up |
| VIT_17s0000g06130.t01 | 782 | glutathione S-transferase U9 | 100 | 8.6353052 | 0.5 | 0.0581291 | 7.2148427 | 2.28E-20 | 3.22E-21 | up |
| VIT_05s0020g05000.t01 | 456 | protease inhibitor | 104 | 15.401143 | 0.5 | 0.0996863 | 7.2714262 | 4.65E-21 | 6.38E-22 | up |
| VIT_04s0008g04060.t01 | 1141 | BURP domain-containing protein 3-like | 104 | 6.1550578 | 0.5 | 0.0398396 | 7.2714262 | 4.65E-21 | 6.38E-22 | up |
| VIT_05s0077g01600.t01 | 588 | pathogenesis-related protein 10.4 (PR10.4) | 105 | 12.058587 | 0.5 | 0.0773078 | 7.285232 | 3.13E-21 | 4.26E-22 | up |
| VIT_08s0056g00630.t01 | 871 | acid phosphatase 1 | 111 | 8.6057607 | 0.5 | 0.0521894 | 7.3654024 | 2.93E-22 | 3.86E-23 | up |
| VIT_00s0262g00090.t01 | 1272 | receptor-like protein kinase | 120 | 6.3705742 | 0.5 | 0.0357366 | 7.4778771 | 8.91E-24 | 1.10E-24 | up |
| VIT_05s0094g00320.t01 | 822 | chitinase 4-like | 121 | 9.9402658 | 0.5 | 0.0553004 | 7.4898497 | 6.05E-24 | 7.44E-25 | up |
| VIT_16s0100g01150.t01 | 1377 | stilbene synthase 6 | 123 | 6.0319206 | 0.5 | 0.0330116 | 7.513501 | 2.80E-24 | 3.41E-25 | up |
| VIT_11s0016g03190.t01 | 769 | E3 ubiquitin-protein ligase RNF181-like | 124 | 10.888794 | 0.5 | 0.0591118 | 7.5251828 | 1.91E-24 | 2.31E-25 | up |
| VIT_16s0100g01170.t01 | 1340 | stilbene synthase 3 | 140 | 7.0551732 | 0.5 | 0.0339231 | 7.7002695 | 4.43E-27 | 4.93E-28 | up |
| VIT_06s0009g02590.t01 | 1645 | Pectinesterase | 140 | 5.7470712 | 0.5 | 0.0276334 | 7.7002695 | 4.43E-27 | 4.93E-28 | up |
| VIT_12s0059g02510.t01 | 639 | B-box zinc finger protein 32 | 141 | 14.900564 | 0.5 | 0.0711377 | 7.7105379 | 3.05E-27 | 3.38E-28 | up |
| VIT_18s0001g11480.t01 | 1714 | cytochrome P450 82A1-like | 162 | 6.3824679 | 0.5 | 0.026521 | 7.9108365 | 1.32E-30 | 1.33E-31 | up |
| VIT_06s0004g02010.t01 | 313 | extensin-like | 162 | 34.950639 | 0.5 | 0.1452299 | 7.9108365 | 1.32E-30 | 1.33E-31 | up |
| VIT_12s0035g00610.t01 | 1588 | cytochrome P450 CYP82D47 | 163 | 6.9314094 | 0.5 | 0.0286253 | 7.9197147 | 9.19E-31 | 9.23E-32 | up |
| VIT_16s0100g00900.t01 | 1925 | Stilbene synthase 4 | 169 | 5.9284398 | 0.5 | 0.023614 | 7.9718659 | 1.05E-31 | 1.03E-32 | up |
| VIT_08s0105g00200.t01 | 1416 | E3 ubiquitin-protein ligase PUB23 | 173 | 8.2502535 | 0.5 | 0.0321024 | 8.0056147 | 2.49E-32 | 2.40E-33 | up |
| VIT_18s0001g11500.t01 | 1601 | cytochrome P450 82A1-like | 173 | 7.2969138 | 0.5 | 0.0283929 | 8.0056147 | 2.49E-32 | 2.40E-33 | up |
| VIT_08s0007g01090.t01 | 1851 | diacylglycerol kinase theta-like | 188 | 6.8586063 | 0.5 | 0.0245581 | 8.1255754 | 1.21E-34 | 1.10E-35 | up |
| VIT_05s0077g01690.t01 | 631 | pathogenesis-related protein 10.8 (PR10.8) | 189 | 20.226321 | 0.5 | 0.0720396 | 8.1332289 | 8.49E-35 | 7.70E-36 | up |
| VIT_00s2745g00010.t01 | 1153 | receptor-like protein kinase | 194 | 11.362054 | 0.5 | 0.0394249 | 8.1708994 | 1.46E-35 | 1.31E-36 | up |
| VIT_16s0100g01140.t01 | 1428 | stilbene synthase 1 | 198 | 9.363138 | 0.5 | 0.0318326 | 8.2003431 | 3.63E-36 | 3.20E-37 | up |
| VIT_09s0054g01080.t01 | 2104 | polygalacturonase QRT3-like | 211 | 6.7720657 | 0.5 | 0.021605 | 8.2920857 | 4.07E-38 | 3.42E-39 | up |
| VIT_08s0040g01520.t01 | 1897 | scopoletin glucosyltransferase | 211 | 7.5110312 | 0.5 | 0.0239626 | 8.2920857 | 4.07E-38 | 3.42E-39 | up |
| VIT_00s1455g00010.t01 | 1018 | expansin-like B1 | 228 | 15.124169 | 0.5 | 0.0446532 | 8.4038765 | 1.26E-40 | 9.93E-42 | up |
| VIT_09s0002g02950.t01 | 284 | inositol oxygenase 1 | 231 | 54.926014 | 0.5 | 0.1600597 | 8.4227356 | 4.57E-41 | 3.58E-42 | up |
| VIT_18s0117g00550.t01 | 1961 | laccase-15 | 233 | 8.0234799 | 0.5 | 0.0231805 | 8.4351727 | 2.33E-41 | 1.82E-42 | up |
| VIT_00s2849g00010.t01 | 1017 | phenylalanine ammonia-lyase-like | 252 | 16.732623 | 0.5 | 0.0446971 | 8.5482664 | 4.17E-44 | 3.08E-45 | up |
| VIT_08s0040g00920.t01 | 789 | glutathione transferase GST | 280 | 23.96434 | 0.5 | 0.0576134 | 8.7002695 | 4.57E-48 | 3.12E-49 | up |
| VIT_16s0039g01360.t01 | 2187 | Phenylalanine ammonia-lyase | 281 | 8.6764482 | 0.5 | 0.0207851 | 8.7054128 | 3.32E-48 | 2.25E-49 | up |
| VIT_16s0100g00770.t01 | 1509 | stilbene synthase 3 | 284 | 12.709063 | 0.5 | 0.0301239 | 8.7207336 | 1.27E-48 | 8.53E-50 | up |
| VIT_16s0100g00780.t01 | 1390 | Stilbene synthase 5 [Source:UniProtKB/Swiss-Prot;Acc:A5C9M2] | 305 | 14.817314 | 0.5 | 0.0327028 | 8.8236519 | 1.59E-51 | 1.01E-52 | up |
| VIT_17s0000g03160.t01 | 474 | Putative uncharacterized protein [Source:UniProtKB/TrEMBL;Acc: | 323 | 46.015975 | 0.5 | 0.0959008 | 8.9063769 | 5.72E-54 | 3.45E-55 | up |
| VIT_17s0119g00150.t01 | 691 | miraculin | 367 | 35.865134 | 0.5 | 0.0657843 | 9.0906228 | 7.97E-60 | 4.37E-61 | up |
| VIT_12s0059g02600.t01 | 1376 | cysteine-rich repeat secretory protein 38-like | 385 | 18.894123 | 0.5 | 0.0330356 | 9.1597011 | 3.61E-62 | 1.90E-63 | up |
| VIT_14s0128g00600.t01 | 847 | germin-like protein 7 (GER7) | 387 | 30.854037 | 0.5 | 0.0536682 | 9.1671763 | 1.98E-62 | 1.04E-63 | up |
| VIT_05s0020g03280.t01 | 2030 | Amine oxidase [Source:UniProtKB/TrEMBL;Acc:D7T6W4] | 387 | 12.873581 | 0.5 | 0.0223926 | 9.1671763 | 1.98E-62 | 1.04E-63 | up |
| VIT_16s0100g00910.t01 | 1401 | stilbene synthase 5 | 411 | 19.810167 | 0.5 | 0.0324461 | 9.2539811 | 1.63E-65 | 8.24E-67 | up |
| VIT_00s0194g00180.t01 | 637 | rho GTPase-activating protein gacV-like | 427 | 45.26608 | 0.5 | 0.071361 | 9.3090788 | 1.52E-67 | 7.46E-69 | up |
| VIT_16s0100g01100.t01 | 1475 | stilbene synthase 1 | 442 | 20.235535 | 0.5 | 0.0308183 | 9.3588891 | 1.96E-69 | 9.44E-71 | up |
| VIT_02s0025g04290.t01 | 722 | thaumatin-like protein | 471 | 44.052256 | 0.5 | 0.0629598 | 9.4505698 | 4.88E-73 | 2.24E-74 | up |
| VIT_07s0141g00930.t01 | 908 | cysteine-rich repeat secretory protein 55 | 472 | 35.102706 | 0.5 | 0.0500627 | 9.4536296 | 3.69E-73 | 1.69E-74 | up |

| | | | | | | | | | | |
|---|---|---|---|---|---|---|---|---|---|---|
| VIT_10s0003g00480.t01 | 1629 | trans-resveratrol di-O-methyltransferase | 489 | 20.270862 | 0.5 | 0.0279048 | 9.5046772 | 3.08E-75 | 1.35E-76 | up |
| VIT_03s0088g00890.t01 | 489 | pathogenesis-related protein 1 | 720 | 99.427857 | 0.5 | 0.092959 | 10.06284 | 5.17E-102 | 1.71E-103 | up |
| VIT_09s0002g01320.t01 | 874 | germin-like protein | 724 | 55.938598 | 0.5 | 0.0520103 | 10.070832 | 1.87E-102 | 6.17E-104 | up |
| expressed only in the control group | | | | | | | | | | |
| VIT_15s0024g00900.t01 | 672 | trnF-ndhJ intergenic spacer | 0.5 | 0.0502441 | 69 | 9.3349112 | -7.537538 | 1.44E-18 | 2.20E-19 | down |
| VIT_10s0003g02720.t01 | 713 | PLASMODESMATA CALLOSE-BINDING PROTEIN 2-like | 0.5 | 0.0473549 | 42 | 5.355378 | -6.821331 | 2.96E-12 | 6.17E-13 | down |
| VIT_05s0136g00380.t01 | 285 | NADH dehydrogenase [ubiquinone] complex I, | 0.5 | 0.1184703 | 31 | 9.8888823 | -6.38321 | 1.69E-09 | 4.32E-10 | down |
| VIT_00s1211g00020.t01 | 473 | tRNA (guanine(9)-N1)-methyltransferase-like | 0.5 | 0.0713828 | 28 | 5.3817964 | -6.236368 | 1.02E-08 | 2.76E-09 | down |
| VIT_07s0031g02860.t01 | 469 | elongation factor 2 | 0.5 | 0.0719916 | 26 | 5.040004 | -6.129453 | 3.43E-08 | 9.69E-09 | down |
| VIT_04s0008g03070.t01 | 311 | endonuclease V | 0.5 | 0.1085661 | 26 | 7.6005204 | -6.129453 | 3.43E-08 | 9.69E-09 | down |
| VIT_00s0365g00010.t01 | 69 | Putative uncharacterized protein [Source:UniProtKB/TrEMBL;Acc: | 0.5 | 0.489334 | 24 | 31.622232 | -6.013976 | 1.17E-07 | 3.46E-08 | down |
| VIT_13s0064g01840.t01 | 132 | disease resistance protein | 0.5 | 0.2557882 | 18 | 12.397352 | -5.598938 | 5.02E-06 | 1.76E-06 | down |
| VIT_18s0122g00650.t01 | 255 | bifunctional dihydroflavonol 4-reductase/flavanone 4-reductase-like | 0.5 | 0.132408 | 18 | 6.417453 | -5.598938 | 5.02E-06 | 1.76E-06 | down |
| VIT_19s0085g00320.t01 | 270 | Putative uncharacterized protein [Source:UniProtKB/TrEMBL;Acc: | 0.5 | 0.125052 | 17 | 5.7242096 | -5.516476 | 9.52E-06 | 3.45E-06 | down |
| VIT_18s0072g00030.t01 | 288 | FAR1-RELATED SEQUENCE 9-like | 0.5 | 0.1172363 | 16 | 5.0507732 | -5.429013 | 1.82E-05 | 6.80E-06 | down |
| VIT_05s0077g00640.t01 | 279 | Putative uncharacterized protein [Source:UniProtKB/TrEMBL;Acc: | 0.5 | 0.1210181 | 16 | 5.2137014 | -5.429013 | 1.82E-05 | 6.80E-06 | down |
| VIT_17s0000g03470.t01 | 156 | cysteine desulfurase 1 | 0.5 | 0.2164362 | 15 | 8.7417228 | -5.335904 | 3.48E-05 | 1.35E-05 | down |
| VIT_09s0018g02000.t01 | 194 | Putative uncharacterized protein | 0.5 | 0.1740415 | 14 | 6.5607982 | -5.236368 | 6.73E-05 | 2.70E-05 | down |
| VIT_14s0006g03160.t01 | 246 | HEAT repeat-containing protein 5B | 0.5 | 0.1372522 | 14 | 5.1739628 | -5.236368 | 6.73E-05 | 2.70E-05 | down |
| VIT_19s0027g01930.t01 | 219 | peroxiredoxin (Prx) | 0.5 | 0.1541737 | 13 | 5.3967166 | -5.129453 | 0.00013 | 5.43E-05 | down |
| VIT_10s0003g03620.t01 | 208 | beta-amyrin synthase | 0.5 | 0.1623271 | 13 | 5.6821199 | -5.129453 | 0.00013 | 5.43E-05 | down |
| VIT_05s0077g00380.t01 | 138 | Putative uncharacterized protein [Source:UniProtKB/TrEMBL;Acc: | 0.5 | 0.244667 | 12 | 7.9055581 | -5.013976 | 0.0002532 | 0.00011 | down |
| VIT_08s0007g06840.t01 | 45 | Putative uncharacterized protein | 0.5 | 0.7503121 | 12 | 24.243711 | -5.013976 | 0.0002532 | 0.00011 | down |
| VIT_00s1351g00020.t01 | 183 | Putative uncharacterized protein [Source:UniProtKB/TrEMBL;Acc: | 0.5 | 0.184503 | 11 | 5.464771 | -4.888445 | 0.0004955 | 0.0002247 | down |
| VIT_15s0046g02670.t01 | 126 | Putative uncharacterized protein [Source:UniProtKB/TrEMBL;Acc: | 0.5 | 0.2679686 | 11 | 7.9369293 | -4.888445 | 0.0004955 | 0.0002247 | down |
| VIT_18s0001g05330.t01 | 66 | Putative uncharacterized protein [Source:UniProtKB/TrEMBL;Acc: | 0.5 | 0.5115764 | 10 | 13.774836 | -4.750942 | 0.0009687 | 0.0004631 | down |

Table S4 Gene Ontology (GO) functional annotation of transcripts.

| | GO_item | Cluster frquency | Genome frquency of use | P_value | q_value(BH_adjust) |
|---|---|---|---|---|---|
| biological_process | signaling process | 123 out of 1631 genes | 862 out of 10973 genes | 0.674924794 | 1 |
| biological_process | reproduction | 62 out of 1631 genes | 564 out of 10973 genes | 0.996276933 | 1 |
| biological_process | cellular component biogenesis | 47 out of 1631 genes | 308 out of 10973 genes | 0.383667615 | 1 |
| biological_process | developmental process | 112 out of 1631 genes | 1084 out of 10973 genes | 0.999997354 | 1 |
| biological_process | multicellular organismal process | 105 out of 1631 genes | 1105 out of 10973 genes | 0.999999982 | 1 |
| biological_process | cellular process | 1003 out of 1631 genes | 7078 out of 10973 genes | 0.996654341 | 1 |
| biological_process | signaling | 130 out of 1631 genes | 917 out of 10973 genes | 0.710883762 | 1 |
| biological_process | biological regulation | 318 out of 1631 genes | 2537 out of 10973 genes | 0.999923503 | 1 |
| biological_process | reproductive process | 55 out of 1631 genes | 539 out of 10973 genes | 0.999277407 | 1 |
| biological_process | carbon utilization | 1 out of 1631 genes | 7 out of 10973 genes | 0.279595763 | 0.9258996 |
| biological_process | response to stimulus | 418 out of 1631 genes | 2416 out of 10973 genes | 7.45E-05 | 0.0019004 |
| biological_process | locomotion | 1 out of 1631 genes | 19 out of 10973 genes | 0.797308749 | 1 |
| biological_process | cellular component organization | 124 out of 1631 genes | 934 out of 10973 genes | 0.917252341 | 1 |
| biological_process | multi-organism process | 63 out of 1631 genes | 404 out of 10973 genes | 0.307364043 | 0.9258996 |
| biological_process | growth | 19 out of 1631 genes | 147 out of 10973 genes | 0.701723024 | 1 |
| biological_process | metabolic process | 1040 out of 1631 genes | 7030 out of 10973 genes | 0.59833927 | 1 |
| biological_process | death | 10 out of 1631 genes | 120 out of 10973 genes | 0.976763118 | 1 |
| biological_process | immune system process | 9 out of 1631 genes | 157 out of 10973 genes | 0.99971994 | 1 |
| biological_process | establishment of localization | 201 out of 1631 genes | 1504 out of 10973 genes | 0.958577879 | 1 |
| biological_process | cell wall organization or biogenesis | 55 out of 1631 genes | 351 out of 10973 genes | 0.301573967 | 0.9258996 |
| biological_process | rhythmic process | 14 out of 1631 genes | 56 out of 10973 genes | 0.014466939 | 0.1475628 |
| biological_process | cell killing | 11 out of 1631 genes | 11 out of 10973 genes | 0 | 0 |
| biological_process | localization | 204 out of 1631 genes | 1530 out of 10973 genes | 0.963289477 | 1 |
| | | | | | |
| cellular_component | macromolecular complex | 176 out of 1631 genes | 1289 out of 10973 genes | 0.896641053 | 1 |
| cellular_component | extracellular region | 139 out of 1631 genes | 903 out of 10973 genes | 0.300626422 | 0.9258996 |
| cellular_component | cell part | 1239 out of 1631 genes | 8436 out of 10973 genes | 0.820599305 | 1 |
| cellular_component | organelle | 822 out of 1631 genes | 5454 out of 10973 genes | 0.262715917 | 0.9258996 |
| cellular_component | symplast | 3 out of 1631 genes | 20 out of 10973 genes | 0.345624718 | 0.97927 |
| cellular_component | cell | 1239 out of 1631 genes | 8436 out of 10973 genes | 0.820599305 | 1 |
| cellular_component | membrane-enclosed lumen | 75 out of 1631 /genes | 444 out of 10973 genes | 0.099290815 | 0.8422795 |
| cellular_component | extracellular region part | 5 out of 1631 genes | 44 out of 10973 genes | 0.655195067 | 1 |
| cellular_component | organelle part | 356 out of 1631 genes | 2093 out of 10973 genes | 0.001101123 | 0.0187191 |
| | | | | | |
| molecular_function | transporter activity | 101 out of 1631 genes | 759 out of 10973 genes | 0.885282761 | 1 |
| molecular_function | binding | 1003 out of 1631 genes | 7367 out of 10973 genes | 0.999999886 | 1 |
| molecular_function | translation regulator activity | 2 out of 1631 genes | 4 out of 10973 genes | 0.011655172 | 0.1475628 |
| molecular_function | enzyme regulator activity | 15 out of 1631 genes | 131 out of 10973 genes | 0.836770159 | 1 |
| molecular_function | molecular transducer activity | 45 out of 1631 genes | 638 out of 10973 genes | 1 | 1 |
| molecular_function | antioxidant activity | 22 out of 1631 genes | 138 out of 10973 genes | 0.308633188 | 0.9258996 |
| molecular_function | transcription regulator activity | 92 out of 1631 genes | 695 out of 10973 genes | 0.884039702 | 1 |
| molecular_function | catalytic activity | 878 out of 1631 genes | 6090 out of 10973 genes | 0.925242197 | 1 |
| molecular_function | nutrient reservoir activity | 6 out of 1631 genes | 78 out of 10973 genes | 0.956175269 | 1 |
| molecular_function | electron carrier activity | 42 out of 1631 genes | 346 out of 10973 genes | 0.917411413 | 1 |
| molecular_function | structural molecule activity | 52 out of 1631 genes | 362 out of 10973 genes | 0.571581704 | 1 |

Table S5 KEGG pathway mapping.

| KEGG_pathway | Cluster frquency | Genome frquency of use | P_value | q_value(BH_adjust) |
|---|---|---|---|---|
| Nervous system | 25 out of 737 genes | 177 out of 4132 genes | 0.890552894 | 0.998787276 |
| Cell growth and death | 34 out of 737 genes | 211 out of 4132 genes | 0.714512563 | 0.965384122 |
| Metabolism of terpenoids and polyketides | 33 out of 737 genes | 182 out of 4132 genes | 0.411385663 | 0.900956411 |
| Signaling molecules and interaction | 0 out of 737 genes | 2 out of 4132 genes | 0.32494974 | 0.900956411 |
| Energy metabolism | 74 out of 737 genes | 360 out of 4132 genes | 0.070953756 | 0.378420033 |
| Folding, sorting and degradation | 80 out of 737 genes | 465 out of 4132 genes | 0.619407533 | 0.900956411 |
| Membrane transport | 5 out of 737 genes | 32 out of 4132 genes | 0.518374624 | 0.900956411 |
| Excretory system | 10 out of 737 genes | 64 out of 4132 genes | 0.606035098 | 0.900956411 |
| Nucleotide metabolism | 26 out of 737 genes | 165 out of 4132 genes | 0.724038092 | 0.965384122 |
| Replication and repair | 14 out of 737 genes | 142 out of 4132 genes | 0.994675786 | 0.998787276 |
| Sensory system | 1 out of 737 genes | 25 out of 4132 genes | 0.953147847 | 0.998787276 |
| Circulatory system | 9 out of 737 genes | 55 out of 4132 genes | 0.528902389 | 0.900956411 |
| Immune system | 18 out of 737 genes | 160 out of 4132 genes | 0.986211325 | 0.998787276 |
| Translation | 109 out of 737 genes | 592 out of 4132 genes | 0.322636117 | 0.900956411 |
| Carbohydrate metabolism | 130 out of 737 genes | 594 out of 4132 genes | 0.002653714 | 0.025372891 |
| Transport and catabolism | 47 out of 737 genes | 308 out of 4132 genes | 0.876109299 | 0.998787276 |
| Glycan biosynthesis and metabolism | 20 out of 737 genes | 114 out of 4132 genes | 0.473483847 | 0.900956411 |
| Lipid metabolism | 62 out of 737 genes | 351 out of 4132 genes | 0.500960979 | 0.900956411 |
| Endocrine system | 41 out of 737 genes | 236 out of 4132 genes | 0.534833327 | 0.900956411 |
| Xenobiotics biodegradation and metabolism | 32 out of 737 genes | 181 out of 4132 genes | 0.47514241 | 0.900956411 |
| Cell motility | 6 out of 737 genes | 57 out of 4132 genes | 0.904543966 | 0.998787276 |
| Environmental adaptation | 30 out of 737 genes | 146 out of 4132 genes | 0.162826259 | 0.651305036 |
| Overview | 96 out of 737 genes | 395 out of 4132 genes | 0.000252372 | 0.008075919 |
| Metabolism of cofactors and vitamins | 62 out of 737 genes | 256 out of 4132 genes | 0.003036131 | 0.025372891 |
| Amino acid metabolism | 100 out of 737 genes | 444 out of 4132 genes | 0.003171611 | 0.025372891 |
| Cellular commiunity | 7 out of 737 genes | 71 out of 4132 genes | 0.953780142 | 0.998787276 |
| Transcription | 25 out of 737 genes | 235 out of 4132 genes | 0.998787276 | 0.998787276 |
| Digestive system | 16 out of 737 genes | 84 out of 4132 genes | 0.322537652 | 0.900956411 |
| Signal transduction | 95 out of 737 genes | 543 out of 4132 genes | 0.560905916 | 0.900956411 |
| Development | 5 out of 737 genes | 34 out of 4132 genes | 0.582064436 | 0.900956411 |
| Metabolism of other amino acids | 45 out of 737 genes | 187 out of 4132 genes | 0.010729805 | 0.068670751 |
| Biosynthesis of other secondary metabolites | 42 out of 737 genes | 204 out of 4132 genes | 0.126712339 | 0.579256405 |

Table S6 Differently-expressed genes related to chlorophylls metabolism.

| #Gene | Length | Function | Cu-treated | RPKM_A | control | RPKM_B | log2(Fold_c | q-value(Benj | p_value | Result |
|---|---|---|---|---|---|---|---|---|---|---|
| chlorophyll a synthesis from glutamate | | | | | | | | | | |
| VIT_03s0038g04080.t01 | 1894 | glutamyl-tRNA synthetase | 267 | 9.5195349 | 708 | 33.984717 | -1.835923 | 5.04E-78 | 2.14E-79 | down |
| VIT_18s0001g08110.t01 | 2324 | Glutamyl-tRNA reductase | 1839 | 53.435521 | 4320 | 168.99661 | -1.661123 | 0 | 0 | down |
| VIT_18s0001g02550.t01 | 1518 | Delta-aminolevulinic acid dehydratase | 1580 | 70.286151 | 3650 | 218.60066 | -1.636985 | 0 | 0 | down |
| VIT_00s0759g00010.t01 | 1346 | porphobilinogen deaminase | 822 | 41.239292 | 2476 | 167.23838 | -2.019815 | 0 | 0 | down |
| VIT_03s0017g02330.t01 | 1561 | coproporphyrinogen III oxidase | 1103 | 47.715233 | 1935 | 112.69598 | -1.239914 | 4.06E-119 | 1.15E-120 | down |
| VIT_07s0005g02540.t01 | 1842 | protoporphyrinogen oxidase | 2100 | 76.986418 | 3282 | 161.98669 | -1.073199 | 4.37E-160 | 9.09E-162 | down |
| VIT_08s0007g08540.t01 | 4376 | magnesium-chelatase H subunit | 3689 | 56.926671 | 13233 | 274.92319 | -2.271852 | 0 | 0 | down |
| VIT_00s0965g00010.t01 | 489 | magnesium-chelatase D subunit | 25 | 3.4523562 | 119 | 22.124246 | -2.679975 | 1.31E-21 | 1.76E-22 | down |
| VIT_06s0061g00010.t01 | 1963 | magnesium-chelatase D subunit | 373 | 12.831368 | 791 | 36.634187 | -1.513516 | 2.15E-66 | 1.08E-67 | down |
| VIT_10s0003g00890.t01 | 1573 | magnesium-chelatase I subunit | 1572 | 67.485157 | 2921 | 168.82362 | -1.322875 | 2.45E-198 | 4.04E-200 | down |
| VIT_08s0040g00390.t01 | 1460 | magnesium-protoporphyrin IX monomethylester [oxidative] cyclase | 6677 | 308.82537 | 14601 | 909.20145 | -1.557809 | 0 | 0 | down |
| VIT_19s0014g03160.t01 | 1481 | protochlorophyllide oxidoreductase | 9463 | 431.47757 | 19387 | 1190.1068 | -1.463734 | 0 | 0 | down |
| VIT_12s0059g00270.t01 | 1564 | protochlorophyllide oxidoreductase | 848 | 36.613694 | 1569 | 91.204563 | -1.316723 | 3.73E-106 | 1.18E-107 | down |
| chlorophyll cycle | | | | | | | | | | |
| VIT_18s0001g02700.t01 | 2176 | chlorophyllide a oxygenase | 433 | 13.437344 | 1468 | 61.33347 | -2.190427 | 2.79E-202 | 4.47E-204 | down |
| chlorophyll degradation | | | | | | | | | | |
| VIT_05s0020g04040.t01 | 1206 | chlorophyllase | 1173 | 65.680303 | 426 | 32.113871 | 1.0322642 | 1.38E-40 | 1.09E-41 | up |
| | | | | | | | | | | |
| VIT_04s0008g00800.t01 | 1681 | ferrochelatase | 92 | 3.6957668 | 139 | 7.5175696 | -1.024393 | 2.59E-07 | 7.93E-08 | down |
| VIT_18s0001g11040.t01 | 1142 | heme oxygenase | 69 | 4.0800683 | 162 | 12.89672 | -1.660339 | 2.37E-16 | 3.97E-17 | down |

Table S7 Differently-expressed genes related to photosynthesis.

| #Gene | Length | Function | read_A | RPKM_A | read_B | RPKM_B | log2(Fold_change) | q-value(Benjamir | p_value |
|---|---|---|---|---|---|---|---|---|---|
| PSII | | | | | | | | | |
| VIT_00s0396g00010.t01 | 1611 | psbC; photosystem II CP43 chlorophyll apoprotein | 6087 | 255.148021 | 7688 | 433.8585963 | -0.765890461 | 2.96E-212 | 4.49E-214 |
| VIT_12s0055g00080.t01 | 1108 | psbD; photosystem II P680 reaction center D2 protein | 2670 | 162.725623 | 3240 | 265.849362 | -0.708167562 | 1.53E-78 | 6.45E-80 |
| VIT_11s0103g00100.t01 | 335 | psbD; photosystem II P680 reaction center D2 protein | 926 | 186.659726 | 1330 | 360.9418222 | -0.951355638 | 8.79E-54 | 5.33E-55 |
| VIT_18s0001g11710.t01 | 1127 | psbO; photosystem II oxygen-evolving enhancer protein 1 | 19731 | 1182.25082 | 18569 | 1497.941913 | -0.341445538 | 1.59E-118 | 4.56E-120 |
| VIT_00s0207g00210.t01 | 1229 | psbO; photosystem II oxygen-evolving enhancer protein 1 | 26783 | 1471.60679 | 29444 | 2178.08738 | -0.565669599 | 0 | 0 |
| VIT_13s0019g00260.t01 | 989 | psbP; photosystem II oxygen-evolving enhancer protein 2 | 672 | 45.8835936 | 816 | 75.01087641 | -0.70912141 | 1.36E-20 | 1.90E-21 |
| VIT_12s0028g01080.t01 | 988 | psbP; photosystem II oxygen-evolving enhancer protein 2 | 37584 | 2568.80122 | 34804 | 3202.599179 | -0.318147996 | 3.67E-194 | 6.31E-196 |
| VIT_19s0014g05080.t01 | 1020 | psbQ; photosystem II oxygen-evolving enhancer protein 3 | 220 | 14.5648814 | 243 | 21.6589039 | -0.572466281 | 4.55E-05 | 1.79E-05 |
| VIT_01s0026g02680.t01 | 787 | psbQ; photosystem II oxygen-evolving enhancer protein 3 | 1089 | 93.4410243 | 621 | 71.73766561 | 0.38132529 | 2.32E-07 | 7.07E-08 |
| VIT_00s0904g00010.t01 | 877 | psbQ; photosystem II oxygen-evolving enhancer protein 3 | 36652 | 2822.16582 | 29457 | 3053.650252 | -0.113732075 | 2.07E-23 | 2.61E-24 |
| VIT_19s0027g00200.t01 | 588 | psbR; photosystem II 10kDa protein | 48561 | 5576.92416 | 29580 | 4573.526672 | 0.286162588 | 9.57E-165 | 1.92E-166 |
| VIT_18s0001g02740.t01 | 1022 | psbS; photosystem II 22kDa protein | 22644 | 1496.18981 | 23043 | 2049.833076 | -0.454213218 | 1.82E-249 | 2.35E-251 |
| VIT_01s0137g00210.t01 | 790 | psbY; photosystem II PsbY protein | 11501 | 983.089269 | 13926 | 1602.61673 | -0.705035112 | 0 | 0 |
| VIT_12s0059g01810.t01 | 814 | psb27; photosystem II Psb27 protein | 2387 | 198.021551 | 3194 | 356.7310232 | -0.849179237 | 1.05E-105 | 3.32E-107 |
| VIT_07s0005g04400.t01 | 789 | psb28; photosystem II 13kDa protein | 674 | 57.6855897 | 688 | 79.27601434 | -0.458673464 | 1.34E-08 | 3.66E-09 |
| PSI | | | | | | | | | |
| VIT_00s0246g00200.t01 | 824 | psaC; photosystem I subunit VII | 1955 | 160.215302 | 2118 | 233.6840746 | -0.544547473 | 1.01E-32 | 9.68E-34 |
| VIT_07s0151g01000.t01 | 638 | psaD; photosystem I subunit II | 5058 | 535.355895 | 3223 | 459.2720321 | 0.221149358 | 1.94E-11 | 4.31E-12 |
| VIT_05s0020g03180.t01 | 789 | psaD; photosystem I subunit II | 40753 | 3487.92409 | 27642 | 3185.098239 | 0.131030771 | 2.28E-31 | 2.25E-32 |
| VIT_07s0005g04920.t01 | 669 | psaE; photosystem I subunit IV | 13215 | 1333.90682 | 17073 | 2320.139485 | -0.798553651 | 0 | 0 |
| VIT_00s0125g00280.t01 | 913 | psaF; photosystem I subunit III | 73666 | 5448.54766 | 48281 | 4807.683304 | 0.180529859 | 1.15E-103 | 3.71E-105 |
| VIT_19s0015g01760.t01 | 688 | psaG; photosystem I subunit V | 33986 | 3335.76969 | 17807 | 2353.05833 | 0.503482601 | 0 | 0 |
| VIT_09s0002g04320.t01 | 654 | psaH; photosystem I subunit VI | 35405 | 3655.70627 | 23320 | 3241.762322 | 0.173371837 | 1.72E-46 | 1.21E-47 |
| VIT_10s0003g04350.t01 | 487 | psaK; photosystem I subunit X | 26169 | 3628.62935 | 19827 | 3701.335204 | -0.028621097 | 0.049091365 | 0.03310787 |
| VIT_04s0023g00410.t01 | 838 | psaL; photosystem I subunit XI | 23493 | 1893.12331 | 23241 | 2521.39661 | -0.413454683 | 1.46E-211 | 2.23E-213 |
| VIT_04s0044g01410.t01 | 747 | psaN; photosystem I subunit PsaN | 26017 | 2351.91195 | 16156 | 1966.272092 | 0.258371074 | 4.75E-73 | 2.18E-74 |
| VIT_06s0080g00920.t01 | 704 | psaO; photosystem I subunit PsaO | 33945 | 3256.024 | 21207 | 2738.652629 | 0.249645047 | 4.40E-89 | 1.65E-90 |
| cytochrome b6-f complex | | | | | | | | | |
| VIT_12s0134g00510.t01 | 891 | petC; cytochrome b6-f complex iron-sulfur subunit | 500 | 37.8945491 | 242 | 24.69266898 | 0.617907557 | 4.71E-08 | 1.35E-08 |
| photosynthetic electron transport chain | | | | | | | | | |
| VIT_18s0001g00760.t01 | 800 | petE; plastocyanin | 84911 | 7167.34669 | 52302 | 5943.724649 | 0.270071857 | 3.59E-258 | 4.51E-260 |
| VIT_06s0080g00410.t01 | 657 | petF; ferredoxin | 4323 | 444.328642 | 1617 | 223.7561717 | 0.989699667 | 3.22E-137 | 7.68E-139 |
| VIT_05s0020g03490.t01 | 625 | petF; ferredoxin | 1379 | 148.99397 | 892 | 129.7523432 | 0.199493351 | 0.00208487 | 0.00105887 |
| VIT_02s0012g00980.t01 | 866 | petF; ferredoxin | 445 | 34.6997673 | 576 | 60.46930317 | -0.801276966 | 4.09E-18 | 6.36E-19 |
| VIT_12s0035g00270.t01 | 660 | petF; ferredoxin | 140830 | 14409.0612 | 107425 | 14797.61757 | -0.038388574 | 1.12E-10 | 2.60E-11 |
| VIT_04s0023g03510.t01 | 1426 | petH; ferredoxin--NADP+ reductase | 5773 | 273.379834 | 6012 | 383.2920566 | -0.487537275 | 1.25E-74 | 5.56E-76 |
| VIT_10s0003g04880.t01 | 1313 | petH; ferredoxin--NADP+ reductase | 964 | 49.5788845 | 863 | 59.75530152 | -0.269340904 | 0.000145035 | 6.10E-05 |
| ATP synthase | | | | | | | | | |
| VIT_10s0116g01740.t01 | 5887 | ATPF1G, atpG; F-type H+-transporting ATPase subunit gamma | 7074 | 81.1438226 | 7109 | 109.7854663 | -0.436133907 | 6.17E-72 | 2.88E-73 |
| VIT_18s0164g00030.t01 | 925 | ATPF1D, atpH; F-type H+-transporting ATPase subunit delta | 6028 | 440.064114 | 5974 | 587.1564798 | -0.416031312 | 1.32E-55 | 7.81E-57 |
| VIT_10s0003g04310.t01 | 497 | ATPF0C, atpE; F-type H+-transporting ATPase subunit c | 710 | 96.4686949 | 826 | 151.0963701 | -0.647336248 | 6.74E-18 | 1.06E-18 |
| VIT_01s0146g00390.t01 | 733 | ATPF0B, atpF; F-type H+-transporting ATPase subunit b | 321 | 29.5723271 | 394 | 48.86778109 | -0.724635823 | 7.12E-11 | 1.64E-11 |
| photosynthesis-antenna proteins | | | | | | | | | |
| VIT_13s0019g04140.t01 | 988 | LHCA1; light-harvesting complex I chlorophyll a/b binding protein 1 | 34417 | 2352.34226 | 38414 | 3534.784647 | -0.58752434 | 0 | 0 |
| VIT_11s0016g00730.t01 | 997 | LHCA2; light-harvesting complex I chlorophyll a/b binding protein 2 | 1887 | 127.808926 | 2201 | 200.7036436 | -0.651078213 | 2.09E-46 | 1.47E-47 |
| VIT_01s0010g03620.t01 | 1077 | LHCA2; light-harvesting complex I chlorophyll a/b binding protein 2 | 42154 | 2643.0631 | 34843 | 2941.238284 | -0.154212802 | 4.86E-49 | 3.25E-50 |
| VIT_15s0024g00040.t01 | 1063 | LHCA3; light-harvesting complex I chlorophyll a/b binding protein 3 | 47789 | 3035.8417 | 50051 | 4280.651449 | -0.495733806 | 0 | 0 |
| VIT_18s0001g10550.t01 | 932 | LHCA5; light-harvesting complex I chlorophyll a/b binding protein 5 | 510 | 36.9520645 | 543 | 52.96808719 | -0.519468442 | 1.56E-08 | 4.28E-09 |
| VIT_19s0014g00160.t01 | 830 | LHCB1; light-harvesting complex II chlorophyll a/b binding protein 1 | 50785 | 4131.82394 | 64015 | 7011.872814 | -0.763021047 | 0 | 0 |
| VIT_12s0028g00320.t01 | 819 | LHCB1; light-harvesting complex II chlorophyll a/b binding protein 1 | 44709 | 3686.34092 | 41350 | 4590.098282 | -0.316335543 | 3.55E-228 | 4.96E-230 |
| VIT_10s0003g02890.t01 | 832 | LHCB1; light-harvesting complex II chlorophyll a/b binding protein 1 | 392776 | 31879.1006 | 254168 | 27773.32766 | 0.198910883 | 0 | 0 |
| VIT_10s0003g02900.t01 | 816 | LHCB1; light-harvesting complex II chlorophyll a/b binding protein 1 | 266889 | 22086.4013 | 226379 | 25221.81588 | -0.191513779 | 0 | 0 |
| VIT_12s0057g00630.t01 | 935 | LHCB2; light-harvesting complex II chlorophyll a/b binding protein 2 | 281019 | 20295.9094 | 166840 | 16222.54333 | 0.323188968 | 0 | 0 |
| VIT_19s0014g03660.t01 | 866 | LHCB3; light-harvesting complex II chlorophyll a/b binding protein 3 | 21041 | 1640.71417 | 28493 | 2991.23586 | -0.866417748 | 0 | 0 |
| VIT_00s0181g00180.t01 | 818 | LHCB3; light-harvesting complex II chlorophyll a/b binding protein 3 | 7794 | 643.415533 | 9249 | 1027.949663 | -0.67594695 | 3.00E-205 | 4.74E-207 |
| VIT_00s0181g00200.t01 | 813 | LHCB3; light-harvesting complex II chlorophyll a/b binding protein 3 | 5363 | 445.452802 | 5973 | 667.9321401 | -0.584428959 | 1.11E-102 | 3.65E-104 |
| VIT_08s0007g02190.t01 | 1160 | LHCB4; light-harvesting complex II chlorophyll a/b binding protein 4 | 50530 | 2941.54673 | 46293 | 3628.170679 | -0.30266737 | 4.86E-235 | 6.54E-237 |
| VIT_18s0089g01170.t01 | 1323 | LHCB5; light-harvesting complex II chlorophyll a/b binding protein 5 | 45799 | 2337.65596 | 50058 | 3439.885781 | -0.557298044 | 0 | 0 |
| VIT_18s0122g00430.t01 | 1076 | LHCB7; light-harvesting complex II chlorophyll a/b binding protein 7 | 288 | 18.0744321 | 283 | 23.91137424 | -0.403746732 | 0.001562083 | 0.00077352 |

Table S8 Differently-expressed genes related to ROS producing and scavenging system.

| #Gene | Length | Function | read_A | RPKM_A | read_B | RPKM_B | log2(Fold_change) | q-value(Benjamini e | p_value | Result |
|---|---|---|---|---|---|---|---|---|---|---|
| ROS Producting | | | | | | | | | | |
| Rboh | | | | | | | | | | |
| VIT_02s0025g00510.t01 | 3159 | respiratory burst oxidase | 281 | 6.0067719 | 90 | 2.5901401 | 1.213559733 | 2.37E-13 | 4.62E-14 | up |
| VIT_14s0060g02320.t01 | 3225 | respiratory burst oxidase | 269 | 5.6325753 | 38 | 1.0712338 | 2.394521358 | 1.80E-31 | 1.78E-32 | up |
| AO | | | | | | | | | | |
| VIT_02s0025g04560.t01 | 2404 | Amine oxidase | 1012 | 28.426965 | 366 | 13.841304 | 1.038280246 | 1.74E-35 | 1.56E-36 | up |
| VIT_00s1937g00010.t01 | 1412 | Amine oxidase | 365 | 17.455915 | 99 | 6.3742761 | 1.453382543 | 4.02E-22 | 5.31E-23 | up |
| VIT_05s0094g01210.t01 | 1398 | Amine oxidase | 298 | 14.394399 | 77 | 5.0074189 | 1.523368489 | 1.68E-19 | 2.46E-20 | up |
| VIT_00s1682g00010.t01 | 617 | Amine oxidase | 276 | 30.207053 | 60 | 8.8408996 | 1.77262037 | 2.86E-22 | 3.76E-23 | up |
| VIT_05s0020g03280.t01 | 2030 | Amine oxidase | 387 | 12.873581 | 0.5 | 0.0223926 | 9.167176265 | 1.98E-62 | 1.04E-63 | up |
| | | | | | | | | | | |
| ROS Scavenging | | | | | | | | | | |
| SOD | | | | | | | | | | |
| VIT_08s0007g07280.t01 | 621 | Cu-Zn Superoxide dismutase | 347 | 37.733085 | 268 | 39.234992 | 0.056310829 | 0.680967863 | 0.627935 | no change |
| VIT_14s0030g00830.t01 | 499 | Cu-Zn Superoxide dismutase | 633 | 85.661881 | 419 | 76.33854 | 0.166241765 | 0.087969523 | 0.0629811 | no change |
| VIT_14s0030g00950.t01 | 659 | Cu-Zn Superoxide dismutase | 1029 | 105.44219 | 676 | 93.259193 | 0.17713434 | 0.019146103 | 0.0116951 | no change |
| VIT_14s0030g01150.t01 | 374 | Cu-Zn Superoxide dismutase | 295 | 53.264132 | 182 | 44.241532 | 0.267763013 | 0.063995704 | 0.0445617 | no change |
| VIT_06s0061g00750.t01 | 904 | Cu-Zn Superoxide dismutase | 12257 | 915.58823 | 7599 | 764.21998 | 0.260710937 | 1.67E-35 | 1.49E-36 | no change |
| VIT_10s0042g00100.t01 | 1255 | Fe Superoxide dismutase | 77 | 4.1431575 | 343 | 24.847389 | -2.584291716 | 4.93E-58 | 2.78E-59 | down |
| VIT_16s0013g00260.t01 | 1178 | Fe Superoxide dismutase | 156 | 8.9425989 | 484 | 37.353426 | -2.062474509 | 2.01E-62 | 1.06E-63 | down |
| POD | | | | | | | | | | |
| VIT_07s0129g00360.t01 | 1447 | Peroxidase 73 | 471 | 21.980462 | 64 | 4.0210717 | 2.450569759 | 8.67E-56 | 5.09E-57 | up |
| VIT_08s0058g00980.t01 | 1045 | Peroxidase 1 | 408 | 26.365033 | 47 | 4.0889513 | 2.688822999 | 1.31E-53 | 8.00E-55 | up |
| VIT_08s0040g02200.t01 | 1024 | Peroxidase 21 | 112 | 7.3858845 | 10 | 0.8878312 | 3.056413336 | 1.84E-17 | 2.93E-18 | up |
| VIT_06s0004g07770.t01 | 1205 | Peroxidase 4 | 2052 | 114.99389 | 166 | 12.524241 | 3.198762093 | 0 | 0 | up |
| VIT_08s0058g00970.t01 | 1157 | Peroxidase 1 | 661 | 38.57914 | 28 | 2.200164 | 4.132138049 | 3.77E-123 | 1.02E-124 | up |
| VIT_18s0072g00160.t01 | 1154 | Peroxidase 12 | 3635 | 212.70762 | 123 | 9.6901316 | 4.456211653 | 0 | 0 | up |
| VIT_12s0055g01020.t01 | 970 | Peroxidase 2 | 262 | 18.239545 | 6 | 0.5623541 | 5.01944701 | 1.72E-53 | 1.05E-54 | up |
| CAT | | | | | | | | | | |
| VIT_18s0122g01320.t01 | 1614 | Catalase | 3464 | 144.93017 | 5613 | 316.17089 | -1.12534662 | 4.55E-295 | 5.15E-297 | down |
| VIT_00s0698g00010.t01 | 1763 | Catalase | 16857 | 645.67269 | 5743 | 296.1535 | 1.124457848 | 0 | 0 | up |
| VIT_04s0044g00020.t01 | 1763 | Catalase | 25877 | 991.16523 | 7656 | 394.80258 | 1.327994145 | 0 | 0 | up |
| GSH-AsA cycle | | | | | | | | | | |
| VIT_08s0040g03150.t01 | 1071 | ascorbate peroxidase (APX) | 1319 | 83.164842 | 338 | 28.691787 | 1.535335922 | 9.80E-84 | 3.91E-85 | up |
| VIT_02s0025g00340.t01 | 1727 | monodehydroascorbate reductase (MDAR) | 393 | 15.366843 | 591 | 31.111827 | -1.017642309 | 3.08E-27 | 3.41E-28 | down |
| VIT_14s0006g00630.t01 | 906 | dehydroascorbate reductase (DHAR) | 220 | 16.397549 | 696 | 69.841155 | -2.090597273 | 6.59E-91 | 2.44E-92 | down |
| VIT_07s0141g00390.t01 | 1983 | glutathione reductase (GR) | 465 | 15.834877 | 847 | 38.832117 | -1.294144744 | 3.11E-56 | 1.81E-57 | down |
| | | | | | | | | | | |
| VIT_05s0020g01760.t01 | 355 | glutaredoxin | 12 | 2.2826395 | 53 | 13.573064 | -2.571971445 | 7.80E-10 | 1.95E-10 | down |
| VIT_07s0104g01420.t01 | 749 | glutaredoxin | 52 | 4.6881983 | 257 | 31.194762 | -2.734198322 | 2.04E-46 | 1.44E-47 | down |
| VIT_05s0020g01750.t01 | 320 | glutaredoxin | 240 | 50.646065 | 374 | 106.25564 | -1.069017355 | 5.07E-19 | 7.56E-20 | down |
| VIT_04s0008g01120.t01 | 744 | glutaredoxin | 101 | 9.1671193 | 156 | 19.062596 | -1.056204227 | 1.98E-08 | 5.48E-09 | down |
| VIT_13s0019g00340.t01 | 1290 | glutaredoxin (GRX) | 1441 | 75.432537 | 426 | 30.022736 | 1.329131509 | 9.48E-74 | 4.28E-75 | up |
| VIT_10s0003g00390.t01 | 722 | glutaredoxin | 16928 | 1583.2624 | 2611 | 328.77595 | 2.267723679 | 0 | 0 | up |
| VIT_00s0199g00290.t01 | 417 | glutaredoxin | 47 | 7.6110793 | 6 | 1.3081139 | 2.54061286 | 8.91E-07 | 2.88E-07 | up |
| GPX pathway | | | | | | | | | | |
| VIT_04s0008g06780.t01 | 1009 | Glutathione peroxidase (GPX) | 732 | 48.989652 | 1122 | 101.09556 | -1.045170613 | 5.04E-53 | 3.11E-54 | down |
| VIT_16s0039g00990.t01 | 756 | Glutathione S-transferase | 27 | 2.4117174 | 85 | 10.221803 | -2.083516925 | 5.57E-12 | 1.18E-12 | down |
| VIT_04s0079g00690.t01 | 831 | Glutathione S-transferase | 505 | 41.036924 | 1260 | 137.84782 | -1.748081932 | 3.45E-129 | 8.81E-131 | down |
| VIT_16s0039g00970.t01 | 866 | Glutathione S-transferase | 41 | 3.1970572 | 78 | 8.1885515 | -1.356863705 | 1.49E-06 | 4.94E-07 | down |
| VIT_07s0104g01800.t01 | 928 | Glutathione S-transferase | 1925 | 140.07712 | 653 | 63.972832 | 1.130690058 | 2.44E-76 | 1.06E-77 | up |
| VIT_19s0027g00460.t01 | 822 | Glutathione S-transferase | 545 | 44.772271 | 169 | 18.691548 | 1.260219493 | 2.43E-26 | 2.77E-27 | up |
| VIT_01s0011g01900.t01 | 1400 | Glutathione S-transferase | 163 | 7.8621986 | 49 | 3.1819871 | 1.305004819 | 4.54E-09 | 1.20E-09 | up |
| VIT_19s0015g02890.t01 | 869 | Glutathione S-transferase | 105 | 8.15932 | 31 | 3.2431892 | 1.331035716 | 2.15E-06 | 7.24E-07 | up |
| VIT_04s0159g00040.t01 | 761 | Glutathione S-transferase | 1825 | 161.94318 | 514 | 61.405721 | 1.399042709 | 1.13E-100 | 3.78E-102 | up |
| VIT_08s0007g01420.t01 | 986 | Glutathione S-transferase | 86 | 5.8898737 | 21 | 1.9363005 | 1.604933841 | 7.83E-07 | 2.52E-07 | up |
| VIT_17s0000g02930.t01 | 886 | Glutathione S-transferase | 451 | 34.373778 | 107 | 10.979446 | 1.646503146 | 2.66E-32 | 2.56E-33 | up |
| VIT_01s0026g01340.t01 | 1015 | Glutathione S-transferase | 8564 | 569.76407 | 1527 | 136.77394 | 2.058571243 | 0 | 0 | up |
| VIT_12s0028g00930.t01 | 871 | Glutathione S-transferase | 1339 | 103.81183 | 218 | 22.754574 | 2.18974243 | 3.04E-137 | 7.26E-139 | up |
| VIT_19s0093g00350.t01 | 1048 | Glutathione S-transferase | 98 | 6.3146493 | 15 | 1.3012488 | 2.278805758 | 1.61E-11 | 3.55E-12 | up |
| VIT_19s0093g00110.t01 | 674 | Glutathione S-transferase | 709 | 71.034738 | 77 | 10.386308 | 2.773841786 | 1.65E-95 | 5.77E-97 | up |
| VIT_00s0153g00050.t01 | 878 | Glutathione S-transferase | 1053 | 80.987557 | 78 | 8.076635 | 3.325874011 | 9.26E-168 | 1.80E-169 | up |
| VIT_08s0007g01410.t01 | 942 | Glutathione S-transferase | 74 | 5.3047541 | 5 | 0.4825579 | 3.45851178 | 3.89E-13 | 7.69E-14 | up |
| VIT_06s0004g05670.t01 | 1329 | Glutathione S-transferase | 343 | 17.428242 | 22 | 1.5049708 | 3.533619657 | 5.36E-58 | 3.03E-59 | up |
| VIT_07s0005g04890.t01 | 999 | Glutathione S-transferase | 1854 | 125.32239 | 89 | 8.0994381 | 3.951678607 | 0 | 0 | up |
| VIT_17s0000g02950.t01 | 923 | Glutathione S-transferase | 1871 | 136.88521 | 87 | 8.5693508 | 3.997636857 | 0 | 0 | up |
| VIT_05s0049g01120.t01 | 931 | Glutathione S-transferase | 384 | 27.852616 | 13 | 1.2694747 | 4.455509292 | 1.02E-74 | 4.55E-76 | up |
| VIT_05s0049g01090.t01 | 795 | Glutathione S-transferase | 387 | 32.872163 | 13 | 1.4866427 | 4.466736547 | 2.25E-75 | 9.84E-77 | up |
| VIT_16s0039g01070.t01 | 876 | glutathione S-transferase | 537 | 41.395642 | 12 | 1.2453961 | 5.054802286 | 7.69E-109 | 2.36E-110 | up |
| VIT_19s0093g00320.t01 | 885 | Glutathione S-transferase | 722 | 55.09071 | 12 | 1.2327311 | 5.481879035 | 4.04E-148 | 9.03E-150 | up |
| VIT_12s0028g00920.t01 | 808 | Glutathione S-transferase | 472 | 39.4471 | 6 | 0.6751033 | 5.868667058 | 2.36E-97 | 8.09E-99 | up |
| VIT_08s0007g01400.t01 | 1009 | Glutathione S-transferase | 212 | 14.18826 | 2 | 0.180206 | 6.298906964 | 6.17E-44 | 4.59E-45 | up |
| VIT_05s0049g01070.t01 | 752 | Glutathione S-transferase | 386 | 34.662023 | 3 | 0.3626885 | 6.578481046 | 2.13E-78 | 8.95E-80 | up |
| VIT_05s0049g01100.t01 | 823 | Glutathione S-transferase | 858 | 70.399876 | 6 | 0.6627989 | 6.730857846 | 5.38E-172 | 1.02E-173 | up |
| VIT_17s0000g06130.t01 | 782 | Glutathione S-transferase | 100 | 8.6353052 | 0.5 | 0.0581291 | 7.214842699 | 2.28E-20 | 3.22E-21 | up |
| VIT_05s0049g01080.t01 | 720 | Glutathione S-transferase | 271 | 25.416821 | 1 | 0.1262693 | 7.653135551 | 1.14E-51 | 7.27E-53 | up |
| VIT_08s0040g00920.t01 | 789 | Glutathione S-transferase | 280 | 23.96434 | 0.5 | 0.0576134 | 8.700269526 | 4.57E-48 | 3.12E-49 | up |
| Prx/Trx pathway | | | | | | | | | | |
| VIT_19s0027g01930.t01 | 219 | Peroxiredoxin | 0.5 | 0.1541737 | 13 | 5.3967166 | -5.129453209 | 0.000130036 | 5.43E-05 | down |
| VIT_11s0016g00560.t01 | 860 | Peroxiredoxin Q | 3032 | 238.07577 | 6339 | 670.12014 | -1.492999005 | 0 | 0 | down |
| VIT_19s0014g01470.t01 | 2827 | Thioredoxin | 447 | 10.677416 | 1115 | 35.857452 | -1.747710464 | 2.16E-114 | 6.34E-116 | down |
| VIT_01s0026g01460.t01 | 609 | Thioredoxin | 70 | 7.761849 | 172 | 25.676837 | -1.725995229 | 3.29E-18 | 5.08E-19 | down |
| VIT_13s0084g00790.t01 | 885 | Thioredoxin | 251 | 19.152034 | 476 | 48.898333 | -1.3522877 | 3.19E-34 | 2.93E-35 | down |
| VIT_04s0008g03260.t01 | 1062 | Thioredoxin | 37 | 2.3526734 | 63 | 5.3931985 | -1.196840049 | 0.000101302 | 4.16E-05 | down |
| VIT_18s0072g00400.t01 | 848 | Thioredoxin | 325 | 25.880458 | 492 | 52.747226 | -1.027232088 | 3.42E-23 | 4.34E-24 | down |

| | | | | | | | | | | |
|---|---|---|---|---|---|---|---|---|---|---|
| VIT_14s0066g02560.t01 | 631 | Thioredoxin | 2528 | 270.54042 | 859 | 123.76395 | 1.128252936 | 1.24E-99 | 4.21E-101 | up |
| VIT_08s0007g07620.t01 | 1448 | Thioredoxin | 2156 | 100.54596 | 631 | 39.617874 | 1.343631777 | 1.00E-111 | 3.00E-113 | up |
| VIT_05s0020g04250.t01 | 662 | thioredoxin-like protein 4A | 3014 | 307.44661 | 787 | 108.08044 | 1.508230385 | 2.38E-185 | 4.32E-187 | up |
| AOX | | | | | | | | | | |
| VIT_00s0399g00060.t01 | 967 | Alternative oxidase | 143 | 9.9860562 | 37 | 3.4786091 | 1.52140448 | 6.48E-10 | 1.61E-10 | up |
| VIT_02s0033g01380.t01 | 1185 | Alternative oxidase | 898 | 51.173183 | 139 | 10.664164 | 2.262617071 | 4.39E-96 | 1.52E-97 | up |
| VIT_02s0033g01400.t01 | 1022 | Alternative oxidase | 307 | 20.284856 | 11 | 0.9785255 | 4.373649736 | 2.06E-59 | 1.14E-60 | up |
| PPO | | | | | | | | | | |
| VIT_00s0480g00030.t01 | 779 | polyphenol oxidase | 4416 | 382.80363 | 78 | 9.1030624 | 5.394108747 | 0 | 0 | up |
| VIT_00s0480g00040.t01 | 1050 | polyphenol oxidase | 4325 | 278.1514 | 95 | 8.2255449 | 5.079615318 | 0 | 0 | up |

Table S9 Differently-expressed genes related to heat shock protein (HSP) and pathogenesis-related proteins (PR).

| #Gene | Length | Function | read_A | RPKM_A | read_B | RPKM_B | log2(Fold_change) | q-value(Benjam | p_value | Result |
|---|---|---|---|---|---|---|---|---|---|---|
| Heat shock proteins HSP101 | | | | | | | | | | |
| VIT_17s0000g07190.t01 | 2937 | heat shock protein 101 (HSP101) | 186 | 4.2765489 | 505 | 15.632117 | -1.869994257 | 2.57E-57 | 1.47E-58 | down |
| Heat shock proteins HSP90 | | | | | | | | | | |
| VIT_01s0010g00680.t01 | 2598 | heat shock protein 90 | 910 | 23.653025 | 1785 | 62.46395 | -1.400999115 | 1.45E-132 | 3.61E-134 | down |
| VIT_00s0194g00030.t01 | 2147 | heat shock cognate protein 80 | 1967 | 61.866673 | 3511 | 148.67199 | -1.264898529 | 2.72E-222 | 3.88E-224 | down |
| VIT_02s0025g00280.t01 | 2120 | heat shock protein 83 | 147 | 4.682372 | 232 | 9.9490702 | -1.087322141 | 1.60E-12 | 3.28E-13 | down |
| Heat shock proteins HSP70 | | | | | | | | | | |
| VIT_00s0415g00030.t01 | 2305 | heat shock 70 kDa protein | 313 | 9.1697575 | 788 | 31.080333 | -1.761046463 | 5.84E-82 | 2.39E-83 | down |
| VIT_17s0000g03310.t01 | 2528 | heat shock protein 70 | 5157 | 137.75409 | 12877 | 463.09277 | -1.749206063 | 0 | 0 | down |
| small Heat shock proteins small HSP | | | | | | | | | | |
| VIT_04s0008g01110.t01 | 1415 | heat shock factor protein HSF30 | 76 | 3.6269502 | 202 | 12.978524 | -1.83929746 | 4.79E-23 | 6.11E-24 | down |
| VIT_13s0019g02850.t01 | 725 | 18.2 kDa class I heat shock protein | 101 | 9.407361 | 156 | 19.562167 | -1.056204227 | 1.98E-08 | 5.48E-09 | down |
| VIT_04s0008g01530.t01 | 1493 | 17.9 kDa class II heat shock protein-like | 261 | 11.804977 | 73 | 4.4452217 | 1.409067947 | 1.92E-15 | 3.38E-16 | up |
| VIT_02s0154g00480.t01 | 912 | 23.6 kDa heat shock protein | 351 | 25.989428 | 98 | 9.7692587 | 1.411603885 | 1.90E-20 | 2.68E-21 | up |
| VIT_04s0008g01520.t01 | 532 | 17.1 kDa class II heat shock protein | 370 | 46.965023 | 102 | 17.430864 | 1.429942628 | 6.78E-22 | 9.00E-23 | up |
| VIT_13s0019g03000.t01 | 752 | 18.2 kDa class I heat shock protein-like | 287 | 25.772022 | 79 | 9.5507972 | 1.432112688 | 2.90E-17 | 4.68E-18 | up |
| VIT_13s0019g02930.t01 | 756 | 18.1 kDa class I heat shock protein | 322 | 28.761963 | 72 | 8.6584683 | 1.731978386 | 5.13E-25 | 6.10E-26 | up |
| VIT_13s0019g03160.t01 | 676 | 18.1 kDa class I heat shock protein | 66 | 6.5929789 | 13 | 1.7483446 | 1.91494091 | 1.00E-06 | 3.26E-07 | up |
| VIT_04s0008g01550.t01 | 877 | 17.3 kDa class II heat shock protein | 314 | 24.177673 | 58 | 6.012551 | 2.007626263 | 1.37E-29 | 1.41E-30 | up |
| VIT_04s0008g01510.t01 | 976 | 17.3 kDa class II heat shock protein | 243 | 16.812833 | 41 | 3.8191297 | 2.138247008 | 5.38E-25 | 6.40E-26 | up |
| VIT_13s0019g00860.t01 | 809 | 15.7 kDa heat shock protein, peroxisomal-like | 107 | 8.9314033 | 17 | 1.9104284 | 2.224990654 | 3.80E-12 | 7.99E-13 | up |
| VIT_04s0008g01570.t01 | 552 | 17.3 kDa class II heat shock protein | 139 | 17.004355 | 22 | 3.6233808 | 2.230495963 | 1.70E-15 | 2.97E-16 | up |
| VIT_12s0035g01910.t01 | 1183 | 22.0 kDa class IV heat shock protein-like | 461 | 26.314833 | 46 | 3.5351143 | 2.896047494 | 3.48E-65 | 1.77E-66 | up |
| VIT_04s0008g01590.t01 | 716 | 17.3 kDa class II heat shock protein | 1031 | 97.236672 | 91 | 11.554702 | 3.073020486 | 3.82E-153 | 8.22E-155 | up |
| VIT_18s0089g01270.t01 | 757 | 22.0 kDa class IV heat shock protein | 151 | 13.469935 | 13 | 1.5612694 | 3.10895153 | 1.61E-23 | 2.01E-24 | up |
| VIT_04s0008g01490.t01 | 524 | 17.3 kDa class II heat shock protein | 2826 | 364.18773 | 191 | 33.13847 | 3.458103431 | 0 | 0 | up |
| VIT_16s0098g01060.t01 | 854 | small heat shock protein | 337 | 26.6475 | 16 | 1.7033052 | 3.96759129 | 9.65E-62 | 5.13E-63 | up |
| VIT_04s0008g01500.t01 | 665 | 17.3 kDa class II heat shock protein | 158 | 16.044267 | 3 | 0.410138 | 5.289804757 | 5.07E-33 | 4.82E-34 | up |
| other Heat shock proteins | | | | | | | | | | |
| VIT_08s0032g00960.t01 | 1424 | iron-sulfur cluster co-chaperone protein HscB | 11 | 0.5216355 | 82 | 5.2352115 | -3.327133877 | 1.68E-18 | 2.56E-19 | down |
| VIT_10s0116g00420.t01 | 1425 | chaperone protein dnaJ 50 | 31 | 1.4690321 | 99 | 6.3161248 | -2.104173801 | 6.20E-14 | 1.18E-14 | down |
| VIT_00s0324g00040.t01 | 2037 | dnaJ homolog 1 | 406 | 13.459206 | 1006 | 44.899068 | -1.738092163 | 1.64E-102 | 5.40E-104 | down |
| VIT_13s0064g01360.t01 | 1613 | chaperone protein dnaJ 1 | 166 | 6.9495737 | 340 | 19.163504 | -1.463364996 | 9.63E-28 | 1.05E-28 | down |
| VIT_09s0002g02240.t01 | 1883 | chaperonin CPN60-like 2 | 74 | 2.6537857 | 147 | 7.097369 | -1.41923247 | 4.32E-12 | 9.12E-13 | down |
| VIT_08s0007g07960.t01 | 1061 | chaperone protein dnaJ 6 | 34 | 2.1639538 | 67 | 5.7410297 | -1.40763984 | 4.71E-06 | 1.65E-06 | down |
| VIT_16s0039g01520.t01 | 893 | chaperone DnaJ-domain containing-like | 54 | 4.0834453 | 97 | 9.875308 | -1.274038831 | 2.99E-07 | 9.21E-08 | down |
| VIT_05s0051g00340.t01 | 2136 | chaperonin CPN60 | 549 | 17.356236 | 971 | 41.328377 | -1.251678637 | 4.11E-61 | 2.21E-62 | down |
| VIT_03s0038g04420.t01 | 1301 | chaperone protein dnaJ 10 | 140 | 7.2666657 | 247 | 17.260367 | -1.248097705 | 2.94E-16 | 4.94E-17 | down |
| VIT_16s0050g01460.t01 | 2205 | dnaJ protein ERDJ2A | 308 | 9.4324946 | 542 | 22.347094 | -1.244375992 | 2.97E-34 | 2.73E-35 | down |
| VIT_09s0002g07210.t01 | 1760 | chaperone protein DnaJ-like | 412 | 15.807711 | 706 | 36.468878 | -1.206037337 | 3.17E-42 | 2.42E-43 | down |
| VIT_08s0105g00350.t01 | 1222 | dnaJ homolog subfamily B member 1 | 223 | 12.323047 | 368 | 27.378332 | -1.151675547 | 5.45E-21 | 7.49E-22 | down |
| VIT_17s0000g06420.t01 | 2572 | heat shock protein DnaJ | 142 | 3.7282225 | 222 | 7.8471577 | -1.073682238 | 8.23E-12 | 1.78E-12 | down |
| VIT_00s1286g00010.t01 | 1229 | hsp70-Hsp90 organizing protein 3-like | 168 | 9.2308532 | 253 | 18.715396 | -1.019689643 | 2.58E-12 | 5.36E-13 | down |
| VIT_04s0023g03470.t01 | 910 | chaperone protein dnaJ 11 | 735 | 54.541916 | 262 | 26.175216 | 1.059163947 | 6.99E-27 | 7.84E-28 | up |
| VIT_14s0060g01490.t01 | 758 | dnaJ homolog subfamily B member 3 | 8711 | 776.03847 | 3020 | 362.2164 | 1.099276306 | 0 | 0 | up |
| VIT_05s0029g00500.t01 | 951 | chaperone protein dnaJ 20 | 641 | 45.515776 | 212 | 20.266825 | 1.167246601 | 2.10E-27 | 2.32E-28 | up |
| VIT_03s0038g02110.t01 | 575 | chaperone protein dnaJ 11 | 759 | 89.137074 | 230 | 36.365567 | 1.293452534 | 9.74E-38 | 8.24E-39 | up |
| VIT_09s0018g00620.t01 | 735 | chaperone protein dnaJ 8 | 996 | 91.507448 | 277 | 34.262796 | 1.417246275 | 1.93E-56 | 1.12E-57 | up |
| VIT_06s0004g05770.t01 | 670 | class I heat shock protein | 124 | 12.497735 | 10 | 1.3569241 | 3.203254725 | 5.15E-20 | 7.39E-21 | up |
| heat stress transcription factor | | | | | | | | | | |
| VIT_00s0179g00150.t01 | 2094 | heat stress transcription factor A-6b | 189 | 6.0949419 | 425 | 18.451965 | -1.598090098 | 4.84E-39 | 3.99E-40 | down |
| VIT_11s0016g03940.t01 | 1113 | heat stress transcription factor C-1 | 545 | 33.066314 | 196 | 16.009998 | 1.046389085 | 7.82E-20 | 1.13E-20 | up |
| VIT_12s0028g01410.t01 | 1501 | heat stress transcription factor A-4b | 205 | 9.22269 | 59 | 3.573565 | 1.367823559 | 7.24E-12 | 1.56E-12 | up |
| VIT_10s0597g00050.t01 | 754 | heat stress transcription factor B-2a-like | 112 | 10.030697 | 6 | 0.7234529 | 3.79337893 | 1.26E-20 | 1.76E-21 | up |
| VIT_08s0007g08750.t01 | 1150 | heat stress transcription factor B-3 | 90 | 5.2848068 | 0.5 | 0.0395278 | 7.062839605 | 1.29E-18 | 1.96E-19 | up |
| | | | | | | | | | | |
| pathogenesis-related protein PR-1 | | | | | | | | | | |
| VIT_11s0052g01620.t01 | 874 | pathogenesis-related protein PR-1 | 606 | 46.821534 | 186 | 19.347813 | 1.275001682 | 1.09E-29 | 1.13E-30 | up |
| VIT_19s0014g01180.t01 | 849 | pathogenesis-related protein PR-1 | 190 | 15.112293 | 54 | 5.7825107 | 1.385954615 | 2.78E-11 | 6.22E-12 | up |
| VIT_18s0001g15640.t01 | 1148 | pathogenesis-related protein PR-1 | 3309 | 194.64324 | 881 | 69.769304 | 1.480167877 | 7.64E-198 | 1.26E-199 | up |
| VIT_03s0088g00910.t01 | 1005 | pathogenesis-related protein PR-1 | 273 | 18.34345 | 25 | 2.2615402 | 3.01988746 | 1.36E-40 | 1.08E-41 | up |
| VIT_03s0088g00780.t01 | 693 | pathogenesis-related protein PR-1 | 197 | 19.196296 | 16 | 2.0990226 | 3.193038329 | 4.35E-31 | 4.34E-32 | up |
| VIT_03s0088g00750.t01 | 646 | pathogenesis-related protein PR-1 | 651 | 68.05075 | 43 | 6.0515456 | 3.491235488 | 3.21E-108 | 9.88E-110 | up |
| VIT_03s0088g00810.t01 | 683 | pathogenesis-related protein PR-1 | 22492 | 2223.78 | 1080 | 143.75846 | 3.951295244 | 0 | 0 | up |
| VIT_18s0001g15660.t01 | 903 | pathogenesis-related protein PR-1 | 435 | 32.530141 | 16 | 1.6108778 | 4.3358581 | 1.94E-83 | 7.75E-85 | up |
| VIT_03s0088g00710.t01 | 671 | pathogenesis-related protein PR-1 | 840 | 84.535906 | 2 | 0.2709804 | 8.285232027 | 5.51E-149 | 1.22E-150 | up |
| VIT_03s0088g00890.t01 | 489 | pathogenesis-related protein PR-1 | 720 | 99.427857 | 0.5 | 0.092959 | 10.06283961 | 5.17E-102 | 1.71E-103 | up |
| pathogenesis-related protein PR-2 | | | | | | | | | | |
| VIT_08s0007g06010.t01 | 429 | Beta-1,3-glucanase | 27 | 4.2500194 | 53 | 11.231789 | -1.402046443 | 5.40E-05 | 2.14E-05 | down |
| VIT_08s0007g06030.t01 | 1014 | Beta-1,3-glucanase | 123 | 8.1912768 | 38 | 3.4070304 | 1.265573501 | 7.80E-07 | 2.51E-07 | up |
| VIT_07s0104g00440.t01 | 1019 | Beta-1,3-glucanase | 418 | 27.700432 | 129 | 11.50922 | 1.267118386 | 1.29E-20 | 1.80E-21 | up |
| VIT_08s0007g06000.t01 | 1140 | Beta-1,3-glucanase | 217 | 12.85403 | 41 | 3.2697111 | 1.974985737 | 2.32E-20 | 3.29E-21 | up |
| VIT_08s0007g06040.t01 | 1250 | Beta-1,3-glucanase | 1712 | 92.486467 | 301 | 21.892071 | 2.078833819 | 3.38E-164 | 6.84E-166 | up |
| VIT_08s0007g06060.t01 | 1186 | Beta-1,3-glucanase | 3570 | 203.26751 | 247 | 18.934012 | 3.424327636 | 0 | 0 | up |
| VIT_05s0077g01150.t01 | 1206 | Beta-1,3-glucanase | 1382 | 77.382932 | 40 | 3.015387 | 4.681600315 | 2.97E-272 | 3.63E-274 | up |
| VIT_06s0061g00120.t01 | 1218 | Glucan endo-1,3-beta-glucosidase | 3659 | 202.86147 | 41 | 3.0603207 | 6.050668205 | 0 | 0 | up |
| VIT_06s0061g00100.t01 | 1391 | Beta-1,3-glucanase | 2859 | 138.79425 | 22 | 1.4378909 | 6.592849795 | 0 | 0 | up |
| pathogenesis-related protein PR-3, 4, 8, 11 | | | | | | | | | | |
| VIT_11s0206g00030.t01 | 1136 | chitotriosidase-1 | 206 | 12.24541 | 71 | 5.6821199 | 1.107739917 | 7.18E-09 | 1.93E-09 | up |
| VIT_11s0149g00350.t01 | 1101 | chitotriosidase-1 | 263 | 16.130687 | 76 | 6.2756201 | 1.361977985 | 7.95E-15 | 1.45E-15 | up |
| VIT_11s0149g00380.t01 | 1104 | chitotriosidase-1 | 141 | 8.624511 | 38 | 3.1292834 | 1.462610348 | 2.68E-09 | 6.95E-10 | up |
| VIT_11s0149g00280.t01 | 1206 | acidic mammalian chitinase-like | 542 | 30.348443 | 133 | 10.026162 | 1.597853115 | 4.99E-37 | 4.30E-38 | up |
| VIT_04s0008g00120.t01 | 994 | class I extracellular chitinase (CHI1B) | 325 | 22.079103 | 69 | 6.3109259 | 1.80675796 | 1.06E-26 | 1.20E-27 | up |
| VIT_06s0004g03840.t01 | 1196 | chitinase 2-like | 865 | 48.839293 | 163 | 12.390442 | 1.978814677 | 2.81E-78 | 1.19E-79 | up |
| VIT_03s0038g03400.t01 | 1105 | Basic endochitinase | 4621 | 282.39574 | 525 | 43.194395 | 2.708802271 | 0 | 0 | up |
| VIT_05s0094g00350.t01 | 1284 | endochitinase A-like | 18682 | 982.52314 | 1881 | 133.18464 | 2.883063666 | 0 | 0 | up |
| VIT_05s0094g00220.t01 | 982 | endochitinase PR4 | 1071 | 73.648249 | 81 | 7.4990095 | 3.295879271 | 2.64E-169 | 5.06E-171 | up |
| VIT_05s0094g00360.t01 | 1037 | class IV chitinase (CHI4C) | 3807 | 247.90687 | 212 | 18.586066 | 3.737504909 | 0 | 0 | up |
| VIT_15s0046g01570.t01 | 957 | acidic endochitinase | 3772 | 266.16086 | 129 | 12.254854 | 4.440873214 | 0 | 0 | up |
| VIT_05s0094g00260.t01 | 861 | endochitinase PR4 | 164 | 12.862493 | 4 | 0.4223643 | 4.928538514 | 1.07E-33 | 9.92E-35 | up |
| VIT_16s0050g02220.t01 | 1254 | Acidic endochitinase | 980 | 52.773146 | 15 | 1.0874871 | 5.600733853 | 4.15E-201 | 6.73E-203 | up |
| VIT_05s0094g00320.t01 | 822 | chitinase 4-like | 121 | 9.9402658 | 0.5 | 0.0553004 | 7.489849746 | 6.05E-24 | 7.44E-25 | up |

| | | | | | | | | | | |
|---|---|---|---|---|---|---|---|---|---|---|
| VIT_05s0094g00340.t01 | 847 | Class IV chitinase | 25229 | 2011.4122 | 103 | 11.055648 | 7.507281385 | 0 | 0 | up |
| VIT_05s0094g00330.t01 | 1454 | chitinase 5-like | 7499 | 348.27587 | 18 | 1.1254818 | 8.273544016 | 0 | 0 | up |
| VIT_14s0081g00030.t01 | 550 | pathogenesis-related protein PR4 | 2257 | 277.11071 | 84 | 13.885035 | 4.31885979 | 0 | 0 | up |
| VIT_14s0081g00050.t01 | 740 | pathogenesis-related protein PR4 | 2892 | 263.90706 | 105 | 12.899948 | 4.354592828 | 0 | 0 | up |
| VIT_14s0081g00020.t01 | 605 | pathogenesis-related protein PR4 | 182 | 20.314234 | 4 | 0.6010838 | 5.078781149 | 1.41E-37 | 1.20E-38 | up |
| pathogenesis-related protein PR-5 | | | | | | | | | | |
| VIT_04s0023g03540.t01 | 1372 | thaumatin-like protein | 193 | 9.4992133 | 69 | 4.5722014 | 1.05491909 | 8.01E-08 | 2.34E-08 | up |
| VIT_08s0007g00760.t01 | 1008 | thaumatin-like protein | 129 | 8.6419873 | 38 | 3.4273104 | 1.334286251 | 1.26E-07 | 3.75E-08 | up |
| VIT_02s0025g04330.t01 | 899 | thaumatin-like protein | 662 | 49.72591 | 44 | 4.4496244 | 3.482242297 | 8.20E-110 | 2.49E-111 | up |
| VIT_02s0025g04250.t01 | 713 | thaumatin-like protein | 9661 | 914.99136 | 465 | 59.291685 | 3.947856416 | 0 | 0 | up |
| VIT_02s0025g04260.t01 | 642 | thaumatin-like protein | 4838 | 508.87988 | 207 | 29.313366 | 4.117694606 | 0 | 0 | up |
| VIT_02s0025g04230.t01 | 701 | thaumatin-like protein | 32047 | 3087.1221 | 1260 | 163.41161 | 4.239680179 | 0 | 0 | up |
| VIT_02s0025g04270.t01 | 823 | thaumatin-like protein | 68361 | 5609.0978 | 2618 | 289.20126 | 4.277623008 | 0 | 0 | up |
| VIT_02s0025g04280.t01 | 724 | thaumatin-like protein | 4340 | 404.79544 | 121 | 15.194177 | 4.735602599 | 0 | 0 | up |
| VIT_02s0025g04320.t01 | 415 | thaumatin-like protein | 335 | 54.510624 | 7 | 1.5334878 | 5.151648872 | 1.53E-68 | 7.41E-70 | up |
| VIT_02s0025g04310.t01 | 986 | thaumatin-like protein | 45151 | 3092.2522 | 313 | 28.860098 | 6.743437982 | 0 | 0 | up |
| VIT_02s0025g04300.t01 | 855 | thaumatin-like protein | 31609 | 2496.4857 | 157 | 16.694135 | 7.224413534 | 0 | 0 | up |
| VIT_02s0025g04340.t01 | 969 | thaumatin-like protein | 5915 | 412.20705 | 24 | 2.2517379 | 7.516186462 | 0 | 0 | up |
| VIT_02s0025g04290.t01 | 722 | thaumatin-like protein | 471 | 44.052256 | 0.5 | 0.0629598 | 9.450569759 | 4.88E-73 | 2.24E-74 | up |
| pathogenesis-related protein PR-6 | | | | | | | | | | |
| VIT_05s0020g05040.t01 | 394 | protease inhibitor | 56 | 9.5979006 | 0.5 | 0.115373 | 6.378341431 | 2.31E-12 | 4.79E-13 | up |
| VIT_05s0020g05000.t01 | 456 | protease inhibitor | 104 | 15.401143 | 0.5 | 0.0996863 | 7.271426227 | 4.65E-21 | 6.38E-22 | up |
| pathogenesis-related protein PR-10 | | | | | | | | | | |
| VIT_05s0077g01580.t01 | 738 | Pathogenesis-related protein 10 | 59642 | 5457.3308 | 1078 | 132.79838 | 5.360886063 | 0 | 0 | up |
| VIT_05s0077g01550.t01 | 480 | Pathogenesis-related protein 10 | 2138 | 300.78135 | 23 | 4.3562919 | 6.109470691 | 0 | 0 | up |
| VIT_05s0077g01600.t01 | 588 | Pathogenesis-related protein 10 | 105 | 12.058587 | 0.5 | 0.0773078 | 7.285232027 | 3.13E-21 | 4.26E-22 | up |
| VIT_05s0077g01560.t01 | 668 | Pathogenesis-related protein 10 | 4443 | 449.14265 | 20 | 2.7219736 | 7.36637684 | 0 | 0 | up |
| pathogenesis-related protein PR-14 | | | | | | | | | | |
| VIT_05s0020g03730.t01 | 759 | non-specific lipid-transfer protein | 8 | 0.7117585 | 43 | 5.1505909 | -2.855278246 | 5.12E-09 | 1.36E-09 | down |
| VIT_08s0058g01230.t01 | 646 | non-specific lipid-transfer protein | 72909 | 7621.3704 | 23408 | 3294.2925 | 1.210081749 | 0 | 0 | up |
| VIT_14s0006g02530.t01 | 602 | non-specific lipid-transfer protein | 3752 | 420.87272 | 944 | 142.56269 | 1.561787572 | 1.28E-242 | 1.69E-244 | up |
| VIT_16s0013g00070.t01 | 562 | non-specific lipid-transfer protein | 218 | 26.194169 | 48 | 7.7648898 | 1.754208333 | 1.42E-17 | 2.25E-18 | up |
| VIT_16s0013g00040.t01 | 489 | non-specific lipid-transfer protein | 50 | 6.9047123 | 11 | 2.0450984 | 1.75541108 | 6.96E-05 | 2.79E-05 | up |
| VIT_14s0006g02570.t01 | 1460 | non-specific lipid-transfer protein 2-like | 3334 | 154.20455 | 686 | 42.717087 | 1.851960132 | 4.67E-274 | 5.63E-276 | up |
| VIT_14s0006g02550.t01 | 556 | non-specific lipid-transfer protein | 2123 | 257.84555 | 365 | 59.682698 | 2.111122511 | 1.90E-207 | 2.99E-209 | up |
| VIT_16s0039g02850.t01 | 501 | non-specific lipid-transfer protein | 130 | 17.522258 | 5 | 0.9073245 | 4.271426227 | 1.86E-25 | 2.18E-26 | up |
| VIT_16s0039g02890.t01 | 363 | non-specific lipid-transfer protein | 32 | 5.9528892 | 1 | 0.2504516 | 4.570986509 | 2.74E-07 | 8.42E-08 | up |
| VIT_08s0007g01370.t01 | 1117 | non-specific lipid-transfer protein | 269 | 16.262359 | 4 | 0.3255646 | 5.642448872 | 7.08E-56 | 4.14E-57 | up |
| pathogenesis-related protein PR-15 | | | | | | | | | | |
| VIT_14s0060g00120.t01 | 733 | Germin-like protein 2 | 1264 | 116.4468 | 357 | 44.278675 | 1.394986993 | 1.04E-69 | 5.02E-71 | up |
| VIT_17s0000g05360.t01 | 799 | Germin-like protein | 954 | 80.628028 | 50 | 5.6892314 | 3.824975775 | 4.37E-169 | 8.40E-171 | up |
| VIT_14s0128g00540.t01 | 834 | germin-like protein 7 (GER7) | 304 | 24.614554 | 1 | 0.1090095 | 7.818914023 | 5.17E-57 | 2.98E-58 | up |
| VIT_14s0128g00570.t01 | 871 | germin-like protein 7 (GER7) | 2285 | 177.15462 | 6 | 0.6262727 | 8.144002458 | 0 | 0 | up |
| VIT_14s0128g00600.t01 | 847 | Germin-like protein 7 (GER7) | 387 | 30.854037 | 0.5 | 0.0536682 | 9.167176265 | 1.98E-62 | 1.04E-63 | up |
| VIT_09s0002g01320.t01 | 874 | Germin-like protein | 724 | 55.938598 | 0.5 | 0.0520103 | 10.0708324 | 1.87E-102 | 6.17E-104 | up |
| transcriptional activator | | | | | | | | | | |
| VIT_06s0004g08190.t01 | 1938 | pathogenesis-related genes transcriptional activator PTI6 | 1905 | 66.378227 | 350 | 16.418922 | 2.01535068 | 1.75E-175 | 3.29E-177 | up |
| VIT_10s0003g00580.t01 | 1058 | pathogenesis-related genes transcriptional activator PTI5-like | 387 | 24.700727 | 83 | 7.1321882 | 1.792136834 | 2.95E-31 | 2.93E-32 | up |
| | | | | | | | | | | |
| dirigent protein | | | | | | | | | | |
| VIT_08s0007g06960.t01 | 687 | dirigent protein 2 | 345 | 33.911484 | 118 | 15.615491 | 1.118796011 | 2.57E-14 | 4.80E-15 | up |
| VIT_06s0004g00990.t01 | 810 | dirigent protein 22 | 1069 | 89.120401 | 254 | 28.508809 | 1.64434796 | 4.74E-75 | 2.09E-76 | up |
| VIT_06s0004g00980.t01 | 778 | dirigent protein 19 | 798 | 69.264027 | 188 | 21.968916 | 1.656642594 | 7.34E-57 | 4.24E-58 | up |
| VIT_08s0007g06900.t01 | 746 | dirigent protein 2-like | 71 | 6.4269358 | 12 | 1.4624223 | 2.135771128 | 4.53E-08 | 1.29E-08 | up |
| VIT_06s0004g01000.t01 | 774 | dirigent protein 22 | 360 | 31.408412 | 43 | 5.0507732 | 2.636574851 | 1.88E-46 | 1.32E-47 | up |
| VIT_04s0044g00040.t01 | 850 | dirigent protein 19-like | 316 | 25.104559 | 25 | 2.6739388 | 3.230911068 | 9.29E-50 | 6.14E-51 | up |
| VIT_17s0000g04880.t01 | 730 | dirigent protein 22 | 90 | 8.3253805 | 7 | 0.8717773 | 3.255484683 | 5.05E-15 | 9.12E-16 | up |
| VIT_06s0004g01010.t01 | 851 | dirigent protein 22 | 417 | 33.089556 | 26 | 2.7776285 | 3.574450364 | 6.34E-71 | 3.01E-72 | up |
| VIT_06s0004g01020.t01 | 1652 | dirigent protein 7 | 1598 | 65.320752 | 18 | 0.9905875 | 6.043113201 | 0 | 0 | up |
| VIT_06s0004g01030.t01 | 498 | dirigent protein 22 | 95 | 12.881864 | 1 | 0.1825581 | 6.140842117 | 2.43E-20 | 3.44E-21 | up |
| | | | | | | | | | | |
| proline related protein | | | | | | | | | | |
| VIT_03s0017g02240.t01 | 1109 | proline rich protein 1 (VPRP1) | 110 | 6.6980068 | 40 | 3.2791314 | 1.030418128 | 8.68E-05 | 3.53E-05 | up |
| VIT_13s0019g03220.t01 | 1717 | proline transporter 2-like | 646 | 25.406607 | 205 | 10.854603 | 1.226896764 | 8.55E-30 | 8.80E-31 | up |
| VIT_02s0025g03290.t01 | 2098 | hydroxyproline | 300 | 9.6560657 | 68 | 2.9466856 | 1.712342358 | 5.00E-23 | 6.40E-24 | up |
| VIT_03s0017g02260.t01 | 791 | proline-rich protein 1 (PRP1) | 2333 | 199.16944 | 467 | 53.674841 | 1.891678361 | 1.54E-197 | 2.54E-199 | up |
| VIT_12s0055g00800.t01 | 713 | 14 kDa proline-rich protein DC2.15-like | 112 | 10.607497 | 14 | 1.785126 | 2.570986509 | 8.46E-15 | 1.54E-15 | up |
| VIT_02s0154g00300.t01 | 713 | 14 kDa proline-rich protein | 176 | 16.668925 | 16 | 2.040144 | 3.030418128 | 1.28E-26 | 1.44E-27 | up |
| VIT_05s0049g00740.t01 | 719 | proline-rich cell wall protein 2-like | 620 | 58.230061 | 86 | 10.874266 | 2.42084616 | 3.97E-72 | 1.85E-73 | up |
| VIT_05s0049g00520.t01 | 716 | proline-rich cell wall protein-like (GRIP4) | 360 | 33.952669 | 24 | 3.0473939 | 3.477877105 | 4.42E-60 | 2.42E-61 | up |
| VIT_05s0049g00750.t01 | 551 | proline-rich 33 kDa extensin-related protein-like | 263 | 32.232099 | 16 | 2.6399686 | 3.609905498 | 2.00E-45 | 1.44E-46 | up |
| VIT_05s0049g00790.t01 | 628 | proline-rich 33 kDa extensin-related protein-like | 182 | 19.570242 | 6 | 0.8686043 | 4.493818649 | 4.48E-36 | 3.96E-37 | up |
| VIT_05s0049g00600.t01 | 474 | proline-rich 33 kDa extensin-related protein-like | 1658 | 236.20584 | 42 | 8.0556636 | 4.873897378 | 0 | 0 | up |
| VIT_05s0049g00620.t01 | 405 | proline-rich 33 kDa extensin-related protein-like | 404 | 67.36135 | 3 | 0.6734364 | 6.644235491 | 8.76E-82 | 3.60E-83 | up |
| VIT_05s0049g00810.t01 | 827 | proline-rich 33 kDa extensin-related protein-like | 82 | 6.6956507 | 0.5 | 0.0549661 | 6.928538514 | 3.46E-17 | 5.60E-18 | up |

Table S10 Differently-expressed genes related to secondary metabolism biosynthetic pathways.

| #Gene | Length | Function | read_A | RPKM_A | read_B | RPKM_B | log2(Fold_c | q-value(Benj | p_value | Result |
|---|---|---|---|---|---|---|---|---|---|---|
| shikimate acid pathway | | | | | | | | | | |
| VIT_00s1217g00010.t01 | 1766 | 3-deoxy-D-arabino-heptulosonate-7-phosphate synthase 03 | 494 | 18.88951 | 819 | 42.16223 | -1.158366 | 7.43E-46 | 5.30E-47 | down |
| VIT_14s0030g00660.t01 | 2031 | bifunctional 3-dehydroquinate dehydratase/shikimate dehydrogenase | 7 | 0.2327408 | 217 | 9.7135993 | -5.38321 | 2.38E-60 | 1.29E-61 | down |
| VIT_14s0030g00650.t01 | 2067 | bifunctional 3-dehydroquinate dehydratase/shikimate dehydrogenase | 70 | 2.2868728 | 159 | 6.9933783 | -1.612613 | 1.86E-15 | 3.26E-16 | down |
| VIT_02s0012g01800.t01 | 1193 | shikimate kinase | 149 | 8.4339354 | 231 | 17.603617 | -1.061594 | 4.87E-12 | 1.03E-12 | down |
| VIT_13s0019g04190.t01 | 1679 | chorismate synthase | 304 | 12.226646 | 473 | 25.611842 | -1.066782 | 1.08E-23 | 1.34E-24 | down |
| VIT_14s0066g00970.t01 | 1422 | anthranilate phosphoribosyltransferase | 98 | 4.6538344 | 303 | 19.371953 | -2.057478 | 2.44E-39 | 2.00E-40 | down |
| VIT_17s0000g08660.t01 | 1544 | anthranilate phosphoribosyltransferase | 422 | 18.456511 | 731 | 43.042794 | -1.221642 | 1.46E-44 | 1.07E-45 | down |
| VIT_12s0057g01240.t01 | 1570 | indole-3-glycerol phosphate synthase | 320 | 13.763686 | 576 | 33.354405 | -1.27701 | 8.52E-38 | 7.19E-39 | down |
| VIT_14s0083g00460.t01 | 1762 | tryptophan synthase beta chain 1 | 270 | 10.347664 | 16 | 0.825552 | 3.6478021 | 5.83E-47 | 4.06E-48 | up |
| alkaloid biosynthesis | | | | | | | | | | |
| VIT_06s0009g00850.t01 | 1396 | strictosidine synthase | 2161 | 104.53309 | 529 | 34.450904 | 1.601346 | 3.28E-145 | 7.45E-147 | up |
| VIT_19s0014g02170.t01 | 1356 | aminotransferase | 515 | 25.646729 | 59 | 3.9556941 | 2.6967721 | 1.27E-67 | 6.22E-69 | up |
| Anthocyanins biosynthetic pathways | | | | | | | | | | |
| VIT_16s0039g01110.t01 | 2649 | Phenylalanine ammonia-lyase | 346 | 8.8202031 | 44 | 1.5100839 | 2.5461831 | 4.88E-43 | 3.68E-44 | up |
| VIT_16s0039g01130.t01 | 2142 | Phenylalanine ammonia-lyase | 253 | 7.9760065 | 25 | 1.0610868 | 2.9101239 | 2.12E-36 | 1.86E-37 | up |
| VIT_16s0039g01120.t01 | 2219 | Phenylalanine ammonia-lyase | 496 | 15.094155 | 34 | 1.3930028 | 3.43772 | 1.14E-81 | 4.69E-83 | up |
| VIT_16s0039g01320.t01 | 466 | Phenylalanine ammonia-lyase | 49 | 7.1005928 | 3 | 0.5852827 | 3.6007339 | 2.49E-09 | 6.45E-10 | up |
| VIT_16s0039g01240.t01 | 2308 | Phenylalanine ammonia-lyase | 306 | 8.9530305 | 15 | 0.5908617 | 3.9214838 | 9.53E-56 | 5.61E-57 | up |
| VIT_16s0039g01100.t01 | 2246 | Phenylalanine ammonia-lyase | 773 | 23.240967 | 31 | 1.2548225 | 4.2111148 | 2.23E-145 | 5.04E-147 | up |
| VIT_16s0039g01170.t01 | 2318 | Phenylalanine ammonia-lyase | 458 | 13.342478 | 12 | 0.4706501 | 4.8252278 | 9.78E-92 | 3.57E-93 | up |
| VIT_16s0039g01280.t01 | 2178 | Phenylalanine ammonia-lyase | 258 | 7.9991948 | 6 | 0.2504516 | 4.9972513 | 1.25E-52 | 7.76E-54 | up |
| VIT_16s0039g01300.t01 | 2286 | Phenylalanine ammonia-lyase | 502 | 14.829002 | 7 | 0.2783891 | 5.7351751 | 1.64E-103 | 5.31E-105 | up |
| VIT_00s2508g00010.t01 | 1473 | Phenylalanine ammonia-lyase | 159 | 7.2891824 | 2 | 0.1234405 | 5.8838695 | 1.90E-33 | 1.78E-34 | up |
| VIT_00s2849g00010.t01 | 1017 | Phenylalanine ammonia-lyase | 252 | 16.732623 | 0.5 | 0.0446971 | 8.5482664 | 4.17E-44 | 3.08E-45 | up |
| VIT_16s0039g01360.t01 | 2187 | Phenylalanine ammonia-lyase | 281 | 8.6764482 | 0.5 | 0.0207851 | 8.7054128 | 3.32E-48 | 2.25E-49 | up |
| VIT_11s0065g00350.t01 | 1688 | cinnamate-4-hydroxylase | 128 | 5.1206132 | 26 | 1.4003329 | 1.8705468 | 1.15E-11 | 2.52E-12 | up |
| VIT_01s0010g02740.t01 | 1673 | 4-coumarate--CoA ligase-like 9 | 143 | 5.7719763 | 289 | 15.704795 | -1.444068 | 2.70E-23 | 3.41E-24 | down |
| VIT_17s0000g01790.t01 | 2106 | 4-coumarate--CoA ligase-like 9 | 149 | 4.7776281 | 252 | 10.878588 | -1.187125 | 2.16E-15 | 3.82E-16 | down |
| VIT_16s0100g00950.t01 | 560 | Chalcone and stilbene synthase | 50 | 6.0292934 | 2 | 0.3246926 | 4.2148427 | 2.12E-10 | 5.07E-11 | up |
| VIT_16s0100g01010.t01 | 1364 | Chalcone and stilbene synthase | 121 | 5.9903948 | 4 | 0.2666097 | 4.4898497 | 2.55E-24 | 3.10E-25 | up |
| VIT_16s0100g01130.t01 | 1364 | Chalcone and stilbene synthase | 208 | 10.297538 | 6 | 0.3999146 | 4.6864637 | 8.95E-42 | 6.92E-43 | up |
| VIT_16s0100g00860.t01 | 1251 | Chalcone and stilbene synthase | 98 | 5.28997 | 2 | 0.145346 | 5.1856964 | 8.30E-21 | 1.15E-21 | up |
| VIT_16s0100g01190.t01 | 1521 | Chalcone and stilbene synthase | 282 | 12.52 | 4 | 0.2390899 | 5.7105379 | 1.49E-58 | 8.36E-60 | up |
| VIT_16s0100g01070.t01 | 1445 | Chalcone and stilbene synthase | 321 | 15.001049 | 4 | 0.2516648 | 5.897416 | 1.71E-66 | 8.51E-68 | up |
| VIT_16s0100g00750.t01 | 1361 | Chalcone and stilbene synthase | 434 | 21.533571 | 5 | 0.3339968 | 6.0106096 | 1.82E-89 | 6.81E-91 | up |
| VIT_16s0100g01200.t01 | 1433 | Chalcone and stilbene synthase | 588 | 27.708664 | 6 | 0.3806584 | 6.1856964 | 1.66E-120 | 4.60E-122 | up |
| VIT_16s0100g01120.t01 | 1367 | Chalcone and stilbene synthase | 213 | 10.521933 | 2 | 0.1330123 | 6.3056961 | 3.96E-44 | 2.92E-45 | up |
| VIT_16s0100g01160.t01 | 1285 | Chalcone and stilbene synthase | 320 | 16.816333 | 3 | 0.2122504 | 6.3079521 | 8.64E-66 | 4.35E-67 | up |
| VIT_16s0100g00990.t01 | 1544 | Chalcone and stilbene synthase | 158 | 6.9102576 | 1 | 0.0588821 | 6.8747673 | 3.17E-32 | 3.06E-33 | up |
| VIT_16s0100g01150.t01 | 1377 | Chalcone and stilbene synthase | 123 | 6.0319206 | 0.5 | 0.0330116 | 7.513501 | 2.80E-24 | 3.41E-25 | up |
| VIT_16s0100g01170.t01 | 1340 | Chalcone and stilbene synthase | 140 | 7.0551732 | 0.5 | 0.0339231 | 7.7002695 | 4.43E-27 | 4.93E-28 | up |
| VIT_16s0100g00900.t01 | 1925 | Chalcone and stilbene synthase | 169 | 5.9284398 | 0.5 | 0.023614 | 7.9718659 | 1.05E-31 | 1.03E-32 | up |
| VIT_16s0100g01140.t01 | 1428 | Chalcone and stilbene synthase | 198 | 9.363138 | 0.5 | 0.0318326 | 8.2003431 | 3.63E-36 | 3.20E-37 | up |
| VIT_16s0100g01030.t01 | 1395 | Chalcone and stilbene synthase | 483 | 23.380692 | 1 | 0.0651713 | 8.4868659 | 3.33E-84 | 1.32E-85 | up |
| VIT_16s0100g00830.t01 | 1468 | Chalcone and stilbene synthase | 560 | 25.760033 | 1 | 0.0619305 | 8.7002695 | 3.64E-95 | 1.28E-96 | up |
| VIT_16s0100g00770.t01 | 1509 | Chalcone and stilbene synthase | 284 | 12.709063 | 0.5 | 0.0301239 | 8.7207336 | 1.27E-48 | 8.53E-50 | up |
| VIT_16s0100g00780.t01 | 1390 | Chalcone and stilbene synthase | 305 | 14.817314 | 0.5 | 0.0327028 | 8.8236519 | 1.59E-51 | 1.01E-52 | up |
| VIT_16s0100g00910.t01 | 1401 | Chalcone and stilbene synthase | 411 | 19.810167 | 0.5 | 0.0324461 | 9.2539811 | 1.63E-65 | 8.24E-67 | up |
| VIT_16s0100g01100.t01 | 1475 | Chalcone and stilbene synthase | 442 | 20.235535 | 0.5 | 0.0308183 | 9.3588891 | 1.96E-69 | 9.44E-71 | up |
| VIT_18s0001g03470.t01 | 1236 | flavonol synthase/flavanone 3-hydroxylase | 121 | 6.6107593 | 183 | 13.460556 | -1.02585 | 2.68E-09 | 6.95E-10 | down |
| VIT_13s0047g00210.t01 | 1023 | flavanone 3-dioxygenase-like | 1460 | 96.374395 | 304 | 27.016453 | 1.8348116 | 6.07E-119 | 1.72E-120 | up |
| VIT_13s0067g01020.t01 | 1123 | flavonol synthase/flavanone 3-hydroxylase | 93 | 5.5922636 | 15 | 1.2143444 | 2.2032547 | 1.37E-10 | 3.22E-11 | up |
| VIT_16s0098g00860.t01 | 1133 | flavanone 3-dioxygenase-like | 1162 | 69.256519 | 43 | 3.4503958 | 4.3271161 | 3.07E-221 | 4.45E-223 | up |
| VIT_08s0105g00380.t01 | 1350 | flavonol synthase/flavanone 3-hydroxylase | 505 | 25.260506 | 1 | 0.0673436 | 8.5511261 | 2.24E-87 | 8.59E-89 | up |
| VIT_19s0014g04990.t01 | 755 | dihydroflavonol-4-reductase | 12 | 1.0732941 | 60 | 7.2249471 | -2.750942 | 1.00E-11 | 2.17E-12 | down |
| VIT_03s0038g04220.t01 | 972 | dihydroflavonol-4-reductase | 265 | 18.410435 | 71 | 6.6408314 | 1.4710879 | 1.23E-16 | 2.05E-17 | up |
| VIT_02s0025g01260.t01 | 1334 | anthocyanidin reductase | 31 | 1.5692434 | 118 | 8.0418608 | -2.35746 | 1.08E-18 | 1.63E-19 | down |
| VIT_16s0039g02230.t01 | 1435 | UDP glucose:flavonoid 3-o-glucosyltransferase (UFGT) | 33 | 1.5529107 | 153 | 9.6932609 | -2.642007 | 4.48E-27 | 4.99E-28 | down |
| Lignin biosynthetic pathways | | | | | | | | | | |
| VIT_14s0030g01950.t01 | 1633 | hydroxycinnamoyl-Coenzyme A shikimate/quinate hydroxycinnamoyltransferase-like | 1946 | 80.471314 | 615 | 34.238861 | 1.2328399 | 2.07E-88 | 7.84E-90 | up |
| VIT_11s0037g00440.t01 | 1536 | hydroxycinnamoyl-Coenzyme A shikimate/quinate hydroxycinnamoyltransferase-like | 596 | 26.202304 | 171 | 10.121276 | 1.3723025 | 1.34E-32 | 1.28E-33 | up |
| VIT_04s0023g02900.t01 | 1825 | ferulic acid 5-hydroxylase | 1383 | 51.173339 | 18 | 0.8966852 | 5.8346469 | 9.34E-284 | 1.12E-285 | up |
| VIT_18s0072g00920.t01 | 1876 | caffeic acid 3-O-methyltransferase | 47 | 1.6918017 | 161 | 7.8023138 | -2.205342 | 2.99E-23 | 3.78E-24 | down |
| VIT_16s0098g00850.t01 | 2461 | caffeic acid 3-O-methyltransferase | 3709 | 101.77232 | 380 | 14.037907 | 2.8579455 | 0 | 0 | up |
| VIT_15s0048g02200.t01 | 1300 | caffeoyl-CoA O-methyltransferase | 43 | 2.2336213 | 77 | 5.3849013 | -1.269535 | 5.86E-06 | 2.07E-06 | down |
| VIT_11s0016g02600.t01 | 772 | caffeoyl-CoA O-methyltransferase | 51 | 4.4610523 | 82 | 9.6566596 | -1.11414 | 2.64E-05 | 1.01E-05 | down |
| VIT_13s0067g00620.t01 | 1133 | cinnamoyl-CoA reductase 1-like | 44 | 2.62245 | 73 | 5.8576487 | -1.159406 | 4.32E-05 | 1.70E-05 | down |
| VIT_09s0070g00240.t01 | 1306 | cinnamoyl-CoA reductase | 2306 | 119.23413 | 723 | 50.329833 | 1.2443115 | 4.03E-106 | 1.27E-107 | up |
| VIT_03s0180g00260.t01 | 1268 | cinnamyl alcohol dehydrogenase 1 | 1759 | 93.67658 | 621 | 44.524876 | 1.0730768 | 3.01E-64 | 1.54E-65 | up |
| VIT_07s0129g00360.t01 | 1447 | Peroxidase 73 | 471 | 21.980462 | 64 | 4.0210717 | 2.4505698 | 8.67E-56 | 5.09E-57 | up |
| VIT_08s0058g00980.t01 | 1045 | peroxidase 1 | 408 | 26.365033 | 47 | 4.0889513 | 2.688823 | 1.31E-53 | 8.00E-55 | up |
| VIT_08s0040g02200.t01 | 1024 | peroxidase 21 | 112 | 7.3858845 | 10 | 0.8878312 | 3.0564133 | 1.84E-17 | 2.93E-18 | up |
| VIT_06s0004g07770.t01 | 1205 | Peroxidase 4 | 2052 | 114.99389 | 166 | 12.524241 | 3.1987621 | 0 | 0 | up |
| VIT_08s0058g00970.t01 | 1157 | Peroxidase 1 | 661 | 38.57914 | 28 | 2.200164 | 4.132138 | 3.77E-123 | 1.02E-124 | up |
| VIT_18s0072g00160.t01 | 1154 | Peroxidase 12 | 3635 | 212.70762 | 123 | 9.6901316 | 4.4562117 | 0 | 0 | up |
| VIT_12s0055g01020.t01 | 970 | Peroxidase 2 | 262 | 18.239545 | 6 | 0.5623541 | 5.019447 | 1.72E-53 | 1.05E-54 | up |
| VIT_18s0122g00250.t01 | 1781 | laccase 9 | 3 | 0.1137475 | 132 | 6.7381455 | -5.888445 | 2.90E-37 | 2.48E-38 | down |
| VIT_18s0001g02400.t01 | 2337 | laccase 9 | 138 | 3.9875378 | 709 | 27.581501 | -2.790131 | 4.71E-129 | 1.21E-130 | down |
| VIT_06s0004g04050.t01 | 1784 | laccase 17 | 86 | 3.2552777 | 166 | 8.4594789 | -1.377788 | 5.79E-13 | 1.15E-13 | down |
| VIT_18s0117g00550.t01 | 1961 | laccase 15 | 233 | 8.0234799 | 0.5 | 0.0231805 | 8.4351727 | 2.33E-41 | 1.82E-42 | up |
| Terpenoid biosynthetic pathways | | | | | | | | | | |
| VIT_02s0025g04580.t01 | 1696 | hydroxymethylglutaryl-CoA synthase / HMG-CoA synthase / 3-hydroxy-3-methylglutaryl coenzyme A synthase | 1680 | 66.891029 | 574 | 30.769215 | 1.1203251 | 8.88E-66 | 4.48E-67 | up |
| VIT_17s0000g02800.t01 | 717 | hydroxymethylglutaryl-CoA synthase / HMG-CoA synthase / 3-hydroxy-3-methylglutaryl coenzyme A synthase | 114 | 10.736683 | 36 | 4.5647155 | 1.2339515 | 3.29E-06 | 1.13E-06 | up |
| VIT_05s0020g02130.t01 | 2482 | 1-deoxy-D-xylulose-5-phosphate synthase | 1375 | 37.409798 | 2414 | 88.423125 | -1.241008 | 1.34E-148 | 2.99E-150 | down |
| VIT_17s0000g08390.t01 | 1675 | 1-deoxy-D-xylulose 5-phosphate reductoisomerase | 631 | 25.438939 | 1030 | 55.905275 | -1.135946 | 1.14E-55 | 6.75E-57 | down |
| VIT_04s0023g00600.t01 | 1124 | isopentenyl-diphosphate isomerase | 3234 | 194.29344 | 1175 | 95.039015 | 1.0316454 | 4.24E-110 | 1.29E-111 | up |
| VIT_18s0001g04280.t01 | 1939 | Terpene synthase | 157 | 5.4677203 | 1 | 0.046887 | 6.8656073 | 4.82E-32 | 4.68E-33 | up |
| VIT_00s0441g00020.t01 | 1664 | squalene epoxidase | 29 | 1.1768717 | 110 | 6.0099345 | -2.352392 | 1.86E-17 | 2.96E-18 | down |
| VIT_18s0001g08550.t01 | 2002 | squalene epoxidase | 612 | 20.642952 | 1228 | 55.76538 | -1.43372 | 1.10E-94 | 3.90E-96 | down |

Table S11 Differently-expressed genes related to plant hormones.

| #Gene | Length | Function | read_A | RPKM_A | read_B | RPKM_B | log2(Fold_c | q-value(Benj | p_value | Result |
|---|---|---|---|---|---|---|---|---|---|---|
| auxin | | | | | | | | | | |
| VIT_15s0046g00290.t01 | 2445 | auxin response factor 18 | 157 | 4.3361593 | 576 | 21.417757 | -2.304318 | 6.22E-85 | 2.44E-86 | down |
| VIT_02s0025g01740.t01 | 2720 | auxin response factor 9 | 345 | 8.5651433 | 673 | 22.49451 | -1.393024 | 3.88E-50 | 2.53E-51 | down |
| VIT_10s0003g00420.t01 | 2570 | auxin response factor 3 | 357 | 9.3803606 | 594 | 21.012789 | -1.163552 | 9.07E-34 | 8.43E-35 | down |
| VIT_00s1203g00010.t01 | 696 | auxin response factor 6-like | 38 | 3.6868783 | 59 | 7.7067832 | -1.063729 | 0.00066617 | 0.0003093 | down |
| VIT_13s0019g04380.t01 | 2359 | auxin response factor 18 | 144 | 4.1221045 | 218 | 8.4015405 | -1.027273 | 6.93E-11 | 1.59E-11 | down |
| VIT_07s0141g00290.t01 | 1122 | auxin-responsive protein IAA16-like | 695 | 41.828895 | 1305 | 105.74212 | -1.337978 | 1.25E-90 | 4.64E-92 | down |
| VIT_00s0201g00080.t01 | 504 | WAT1-related protein | 14 | 1.8757802 | 33 | 5.952697 | -1.666053 | 0.0003194 | 0.00014082 | down |
| VIT_01s0026g00480.t01 | 225 | WAT1-related protein | 1 | 0.3001248 | 21 | 8.485299 | -4.821331 | 1.12E-06 | 3.65E-07 | down |
| VIT_18s0001g03170.t01 | 1365 | WAT1-related protein | 5 | 0.2473556 | 83 | 5.528099 | -4.482125 | 1.58E-22 | 2.06E-23 | down |
| VIT_08s0040g00800.t01 | 1444 | WAT1-related protein | 51 | 2.3849947 | 220 | 13.851151 | -2.537948 | 7.36E-37 | 6.38E-38 | down |
| VIT_01s0026g00530.t01 | 1153 | WAT1-related protein | 36 | 2.1084225 | 73 | 5.7560416 | -1.448913 | 9.77E-07 | 3.18E-07 | down |
| VIT_04s0023g00320.t01 | 1207 | auxin efflux carrier component 8 | 4 | 0.2237882 | 70 | 5.2725553 | -4.558297 | 2.67E-19 | 3.94E-20 | down |
| VIT_09s0002g05150.t01 | 829 | auxin-induced protein 22A-like | 97 | 7.9013563 | 175 | 19.19172 | -1.280312 | 2.84E-12 | 5.92E-13 | down |
| VIT_07s0141g00270.t01 | 930 | auxin-induced protein 22D | 276 | 20.040593 | 447 | 43.697335 | -1.12462 | 2.33E-24 | 2.82E-25 | down |
| VIT_19s0085g00010.t01 | 700 | auxin-induced protein X15 | 113 | 10.900963 | 23 | 2.9871716 | 1.8676035 | 2.08E-10 | 4.97E-11 | up |
| VIT_04s0023g00580.t01 | 491 | auxin-induced protein 15A | 775 | 106.5871 | 121 | 22.404448 | 2.2501758 | 1.68E-82 | 6.81E-84 | up |
| VIT_05s0020g04680.t01 | 634 | auxin-induced protein 22D | 78 | 8.3078718 | 11 | 1.5773708 | 2.3969571 | 6.54E-10 | 1.62E-10 | up |
| VIT_03s0038g00950.t01 | 683 | auxin-induced protein 15A-like | 259 | 25.607283 | 35 | 4.6588391 | 2.4585118 | 3.01E-31 | 2.98E-32 | up |
| VIT_03s0038g03450.t01 | 380 | auxin-induced protein 6B-like | 45 | 7.9967471 | 6 | 1.4354829 | 2.4778771 | 2.20E-06 | 7.41E-07 | up |
| VIT_09s0054g00680.t01 | 543 | auxin-induced protein 6B-like | 52 | 6.4667781 | 1 | 0.1674289 | 5.2714262 | 1.38E-11 | 3.03E-12 | up |
| VIT_04s0023g00530.t01 | 370 | auxin-induced protein 15A | 29 | 5.2927419 | 0.5 | 0.1228566 | 5.4289675 | 5.61E-07 | 1.78E-07 | up |
| VIT_08s0058g01160.t01 | 535 | indole-3-acetic acid-induced protein ARG7-like | 82 | 10.350099 | 23 | 3.9084488 | 1.4049766 | 1.37E-05 | 5.04E-06 | up |
| VIT_16s0098g01150.t01 | 789 | indole-3-acetic acid-induced protein ARG7-like | 688 | 58.883807 | 170 | 19.58855 | 1.5878603 | 2.58E-46 | 1.82E-47 | up |
| VIT_18s0001g13980.t01 | 448 | indole-3-acetic acid-induced protein ARG7-like | 119 | 17.937148 | 14 | 2.8410599 | 2.6584494 | 3.40E-16 | 5.74E-17 | up |
| VIT_09s0002g00700.t01 | 934 | auxin-repressed 12.5 kDa protein | 2423 | 175.18261 | 225 | 21.901104 | 2.999784 | 0 | 0 | up |
| VIT_10s0003g00090.t01 | 724 | auxin-repressed 12.5 kDa protein | 3608 | 336.52118 | 306 | 38.424943 | 3.1305823 | 0 | 0 | up |
| VIT_14s0128g00760.t01 | 1861 | protein AUXIN RESPONSE 4-like | 1953 | 70.866391 | 618 | 30.190651 | 1.2309997 | 1.62E-88 | 6.09E-90 | up |
| VIT_14s0083g00960.t01 | 15338 | auxin transport protein BIG-like | 2175 | 9.5757979 | 610 | 3.6156924 | 1.4051208 | 1.36E-120 | 3.76E-122 | up |
| VIT_12s0059g01870.t01 | 1790 | indole-3-acetic acid-amido synthetase GH3.5 | 141 | 5.3192515 | 255 | 12.951424 | -1.283816 | 1.86E-17 | 2.97E-18 | down |
| VIT_07s0104g00800.t01 | 1967 | indole-3-acetic acid-amido synthetase GH3.6 | 218 | 7.4840482 | 15 | 0.6932937 | 3.4322802 | 2.09E-36 | 1.83E-37 | up |
| VIT_07s0104g01260.t01 | 1227 | indole-3-pyruvate monooxygenase | 1144 | 62.960172 | 147 | 10.891887 | 2.5311855 | 3.55E-139 | 8.35E-141 | up |
| VIT_02s0033g00870.t01 | 1196 | bifunctional nitrilase/nitrile hydratase | 411 | 23.205722 | 86 | 6.5372884 | 1.8277163 | 5.21E-34 | 4.82E-35 | up |
| VIT_02s0033g00800.t01 | 1201 | bifunctional nitrilase/nitrile hydratase | 803 | 45.14992 | 122 | 9.2352189 | 2.2895053 | 3.03E-87 | 1.16E-88 | up |
| VIT_02s0033g00670.t01 | 1082 | bifunctional nitrilase/nitrile hydratase | 124 | 7.7388935 | 6 | 0.5041437 | 3.9402203 | 2.85E-23 | 3.60E-24 | up |
| ethylene | | | | | | | | | | |
| VIT_07s0031g00720.t01 | 648 | ethylene-responsive transcription factor ERF109-like | 19 | 1.9799902 | 231 | 32.409128 | -4.032835 | 1.28E-57 | 7.30E-59 | down |
| VIT_18s0001g08610.t01 | 2778 | AP2-like ethylene-responsive transcription factor ANT | 17 | 0.4132388 | 187 | 6.1198353 | -3.888445 | 8.40E-46 | 6.01E-47 | down |
| VIT_07s0031g00710.t01 | 604 | ethylene-responsive transcription factor ERF109-like | 7 | 0.7826103 | 54 | 8.1280655 | -3.376546 | 1.07E-12 | 2.18E-13 | down |
| VIT_03s0063g00460.t01 | 979 | ethylene-responsive transcription factor ERF109 | 903 | 62.285865 | 2262 | 210.05851 | -1.753815 | 2.34E-232 | 3.21E-234 | down |
| VIT_16s0013g01000.t01 | 946 | ethylene-responsive transcription factor 5-like | 1273 | 90.870247 | 415 | 39.882955 | 1.1880357 | 6.55E-55 | 3.91E-56 | up |
| VIT_10s0003g00140.t01 | 1193 | ethylene-responsive transcription factor 4-like | 2944 | 166.64098 | 942 | 71.786178 | 1.2149652 | 2.76E-130 | 6.98E-132 | up |
| VIT_07s0141g00690.t01 | 810 | ethylene-responsive transcription factor 11-like | 114 | 9.5039529 | 34 | 3.8161398 | 1.3164137 | 9.46E-07 | 3.07E-07 | up |
| VIT_16s0013g00990.t01 | 964 | ethylene-responsive transcription factor 5 | 2061 | 144.37281 | 614 | 57.905753 | 1.3180205 | 9.46E-104 | 3.06E-105 | up |
| VIT_00s0184g00010.t01 | 651 | ethylene-responsive transcription factor-like protein | 65 | 6.7424357 | 19 | 2.6534016 | 1.3454268 | 0.00019849 | 8.47E-05 | up |
| VIT_00s0662g00040.t01 | 1456 | ethylene-responsive transcription factor RAP2-4-like | 1028 | 47.677797 | 300 | 18.732263 | 1.3477924 | 5.22E-54 | 3.14E-55 | up |
| VIT_05s0077g01860.t01 | 1089 | ethylene-responsive transcription factor RAP2-3 | 433 | 26.85001 | 104 | 8.6823209 | 1.62877 | 1.36E-30 | 1.38E-31 | up |
| VIT_16s0013g00970.t01 | 951 | ethylene-responsive transcription factor ERF105 | 2808 | 199.38892 | 672 | 64.242011 | 1.6339963 | 5.38E-194 | 9.27E-196 | up |
| VIT_02s0025g04440.t01 | 826 | ethylene-responsive transcription factor ERF026 | 96 | 7.8483006 | 22 | 2.4214361 | 1.6965174 | 5.04E-08 | 1.44E-08 | up |
| VIT_16s0013g01070.t01 | 1077 | ethylene-responsive transcription factor ERF105 | 1418 | 88.908845 | 323 | 27.265734 | 1.705238 | 1.82E-104 | 5.79E-106 | up |
| VIT_16s0013g01090.t01 | 1027 | ethylene-responsive transcription factor 5-like | 340 | 22.355939 | 69 | 6.1081405 | 1.871853 | 3.29E-29 | 3.43E-30 | up |
| VIT_16s0013g01110.t01 | 2139 | ethylene-responsive transcription factor 5-like | 981 | 30.970104 | 194 | 8.2455821 | 1.909183 | 1.39E-84 | 5.47E-86 | up |
| VIT_14s0006g02290.t01 | 1189 | ethylene-responsive transcription factor ERF038-like | 167 | 9.4846009 | 29 | 2.2174126 | 2.0967098 | 4.11E-17 | 6.68E-18 | up |
| VIT_07s0005g03230.t01 | 1040 | ethylene-responsive transcription factor 1B | 147 | 9.5448353 | 25 | 2.1854307 | 2.1268027 | 1.89E-15 | 3.32E-16 | up |
| VIT_07s0005g03230.t01 | 1040 | ethylene-responsive transcription factor ERF113-like | 147 | 9.5448353 | 25 | 2.1854307 | 2.1268027 | 1.89E-15 | 3.32E-16 | up |
| VIT_17s0000g00200.t01 | 1218 | ethylene-responsive transcription factor ERF114 | 214 | 11.864541 | 34 | 2.5378269 | 2.2249907 | 3.29E-23 | 4.17E-24 | up |
| VIT_14s0068g01040.t01 | 1238 | ethylene-responsive transcription factor ERF113-like | 204 | 11.127407 | 23 | 1.6890308 | 2.7198499 | 1.23E-27 | 1.34E-28 | up |
| VIT_18s0001g10150.t01 | 780 | ethylene-responsive transcription factor ERF010-like | 623 | 53.935895 | 56 | 6.5271531 | 3.0467199 | 3.83E-92 | 1.39E-93 | up |
| VIT_09s0002g09140.t01 | 534 | ethylene-responsive transcription factor ERF003-like | 57 | 7.2080542 | 4 | 0.6810031 | 3.4038765 | 2.94E-10 | 7.09E-11 | up |
| VIT_01s0150g00120.t01 | 1245 | ethylene-responsive transcription factor ERF113 | 299 | 16.217589 | 15 | 1.0953484 | 3.8880976 | 3.39E-54 | 2.03E-55 | up |
| VIT_07s0031g01980.t01 | 1208 | ethylene-responsive transcription factor ERF110 | 357 | 19.956562 | 15 | 1.128898 | 4.1438762 | 4.90E-67 | 2.41E-68 | up |
| VIT_05s0049g00510.t01 | 1264 | ethylene-responsive transcription factor 1B | 359 | 19.179259 | 8 | 0.5754045 | 5.0588265 | 4.40E-73 | 2.01E-74 | up |
| VIT_08s0007g03040.t01 | 1237 | 1-aminocyclopropane-1-carboxylate oxidase | 949 | 51.806107 | 303 | 22.269133 | 1.2180768 | 8.17E-43 | 6.20E-44 | up |
| VIT_12s0059g01380.t01 | 1308 | 1-aminocyclopropane-1-carboxylic acid oxidase 2 (ACO2) | 3939 | 203.35866 | 1175 | 81.669613 | 1.3161552 | 4.10E-197 | 6.84E-199 | up |
| VIT_00s2086g00010.t01 | 920 | 1-aminocyclopropane-1-carboxylate oxidase | 10801 | 792.79441 | 2378 | 234.99271 | 1.7543308 | 0 | 0 | up |
| VIT_05s0049g00400.t01 | 1294 | 1-aminocyclopropane-1-carboxylate oxidase | 362 | 18.891165 | 58 | 4.0749669 | 2.2128514 | 2.73E-38 | 2.28E-39 | up |
| VIT_05s0049g00410.t01 | 1285 | 1-aminocyclopropane-1-carboxylate oxidase | 635 | 33.36991 | 48 | 3.3960063 | 3.2966368 | 9.37E-101 | 3.14E-102 | up |
| jasmonate acid | | | | | | | | | | |
| VIT_05s0020g04060.t01 | 1480 | protein TIFY 4B-like | 32 | 1.4600667 | 90 | 5.528549 | -1.920867 | 1.83E-11 | 4.06E-12 | down |
| VIT_10s0003g03790.t01 | 622 | protein TIFY 5A (JASMONATE-ZIM-DOMAIN PROTEIN 8) | 76 | 8.2510202 | 158 | 23.093889 | -1.484867 | 9.64E-14 | 1.85E-14 | down |
| VIT_17s0000g02230.t01 | 1562 | protein TIFY 6B-like (JASMONATE-ZIM-DOMAIN PROTEIN 3) | 1891 | 81.751352 | 3384 | 196.96075 | -1.268594 | 2.98E-215 | 4.42E-217 | down |
| VIT_10s0003g03810.t01 | 521 | protein TIFY 5A-like (JASMONATE-ZIM-DOMAIN PROTEIN 8) | 687 | 89.043753 | 1187 | 207.13017 | -1.217951 | 2.07E-71 | 9.72E-73 | down |
| VIT_01s0146g00480.t01 | 1079 | protein TIFY 9 (JASMONATE-ZIM-DOMAIN PROTEIN 10) | 232 | 14.519477 | 77 | 6.4878329 | 1.162181 | 1.50E-10 | 3.55E-11 | up |
| VIT_06s0004g01470.t01 | 2768 | Lipoxygenase (LOX) | 586 | 14.296047 | 101 | 3.317307 | 2.1075319 | 8.76E-58 | 4.98E-59 | up |
| VIT_18s0001g11630.t01 | 1777 | allene oxide synthase(AOS) | 1801 | 68.440115 | 616 | 31.51546 | 1.1187824 | 2.69E-70 | 1.28E-71 | up |
| VIT_18s0041g02260.t01 | 619 | 12-oxophytodienoate reductase 2-like(OPR) | 60 | 6.5455334 | 11 | 1.6155947 | 2.0184455 | 1.39E-06 | 4.61E-07 | up |
| Abscisic acid | | | | | | | | | | |
| VIT_18s0001g14100.t01 | 1260 | Abscisic acid-responsive protein | 190 | 10.182807 | 283 | 20.419555 | -1.003816 | 2.53E-13 | 4.94E-14 | down |
| VIT_15s0046g01050.t01 | 1128 | Abscisic acid receptor PYL9 | 2203 | 131.88331 | 583 | 46.98831 | 1.4888882 | 4.01E-133 | 9.87E-135 | up |
| VIT_04s0079g00680.t01 | 1794 | phytoene synthase | 414 | 15.583405 | 786 | 39.83185 | -1.353912 | 5.99E-56 | 3.51E-57 | down |
| VIT_12s0028g00960.t01 | 1563 | phytoene synthase | 327 | 14.127757 | 586 | 34.085448 | -1.270624 | 3.83E-38 | 3.21E-39 | down |
| VIT_14s0030g01740.t01 | 1720 | zeta-carotene desaturase | 559 | 21.946628 | 864 | 45.668387 | -1.057197 | 8.04E-42 | 6.21E-43 | down |
| VIT_07s0031g00620.t01 | 2288 | Zeaxanthin epoxidase, chloroplastic | 864 | 25.500117 | 1997 | 79.351002 | -1.637745 | 2.19E-186 | 3.93E-188 | down |
| brassinolide | | | | | | | | | | |

| | | | | | | | | | | |
|---|---|---|---|---|---|---|---|---|---|---|
| VIT_12s0121g00430.t01 | 218 | BRASSINOSTEROID INSENSITIVE 1-associated receptor kinase 1-like | 25 | 7.7440466 | 2 | 0.8340726 | 3.2148427 | 6.32E-05 | 2.52E-05 | up |
| VIT_12s0055g01280.t01 | 1844 | BRASSINOSTEROID INSENSITIVE 1-associated receptor kinase 1-like | 311 | 11.388956 | 48 | 2.3665228 | 2.2667948 | 5.36E-34 | 4.96E-35 | up |
| VIT_12s0121g00300.t01 | 1406 | BRASSINOSTEROID INSENSITIVE 1-associated receptor kinase 1 | 421 | 20.220003 | 138 | 8.9232721 | 1.1801385 | 1.14E-18 | 1.72E-19 | up |
| | | | | | | | | | | |
| gibberellin | | | | | | | | | | |
| VIT_14s0030g00440.t01 | 1574 | gibberellin receptor GID1A (GID1A) | 1341 | 57.53187 | 440 | 25.41431 | 1.1787203 | 4.38E-57 | 2.52E-58 | up |
| VIT_07s0104g00930.t01 | 1699 | gibberellin receptor GID1B | 1157 | 45.985872 | 112 | 5.9931482 | 2.9398047 | 1.17E-164 | 2.35E-166 | up |
| VIT_06s0009g03440.t01 | 670 | GA-binding protein subunit beta-2 | 181 | 18.242662 | 48 | 6.5132359 | 1.4858699 | 7.22E-12 | 1.55E-12 | up |
| VIT_04s0044g02010.t01 | 929 | gibberellin 3-beta-dioxygenase 4-like | 173 | 12.575198 | 50 | 4.8931064 | 1.3617585 | 3.96E-10 | 9.65E-11 | up |
| VIT_13s0067g01150.t01 | 1296 | gibberellin 2-beta-dioxygenase | 66 | 3.4389303 | 135 | 9.4701998 | -1.461435 | 1.19E-11 | 2.60E-12 | down |
| VIT_16s0022g02310.t01 | 1321 | Gibberellin 20 oxidase 1 | 32 | 1.6358053 | 156 | 10.736239 | -2.714416 | 2.30E-28 | 2.46E-29 | down |

Table S12 Differently-expressed genes related to Cu homeostasis pathways.

| #Gene | Length | Function | read_A | RPKM_A | read_B | RPKM_B | log2(Fold_c | q-value(Benj | p_value | Result |
|---|---|---|---|---|---|---|---|---|---|---|
| Cu transporter | | | | | | | | | | |
| VIT_11s0016g00920.t01 | 767 | Copper transporter (CTR1) | 878 | 77.30073 | 298 | 35.322487 | 1.1298951 | 2.52E-35 | 2.27E-36 | up |
| VIT_08s0007g01470.t01 | 844 | Copper transporter(CTR2) | 439 | 35.124206 | 157 | 16.911712 | 1.0544429 | 2.24E-16 | 3.76E-17 | up |
| VIT_03s0110g00430.t01 | 684 | Copper transporter(CTR3) | 972 | 95.960965 | 666 | 88.521446 | 0.1164206 | 0.1376595 | 0.1036232 | no change |
| VIT_04s0008g02090.t01 | 1040 | Copper transporter(CTR8) | 1627 | 105.6425 | 495 | 43.271528 | 1.2877003 | 3.24E-79 | 1.35E-80 | up |
| VIT_06s0004g05070.t01 | 1052 | zinc transporter 2 | 37 | 2.3750373 | 81 | 7.000026 | -1.55941 | 4.54E-08 | 1.30E-08 | down |
| VIT_04s0008g01260.t01 | 1492 | zinc transporter 4 | 1548 | 70.062653 | 387 | 23.581559 | 1.5709865 | 1.95E-101 | 6.48E-103 | up |
| Cu chaperones | | | | | | | | | | |
| VIT_15s0021g01120.t01 | 416 | cytochrome c oxidase-assembly factor COX23 | 51 | 8.2786837 | 8 | 1.7483446 | 2.2434119 | 2.00E-06 | 6.73E-07 | up |
| VIT_02s0025g04830.t01 | 1167 | Cu/Zn-superoxide dismutase copper chaperone (CCS) | 425 | 24.592491 | 626 | 48.767877 | -0.987713 | 1.88E-27 | 2.06E-28 | no change |
| VIT_09s0002g03960.t01 | 665 | cytochrome c oxidase assembly protein COX19 | 39 | 3.9602938 | 15 | 2.0506899 | 0.9494981 | 0.0329526 | 0.021371 | no change |
| VIT_13s0074g00770.t01 | 483 | copper transport protein CCH | 1534 | 214.46808 | 788 | 148.32333 | 0.5320175 | 2.18E-17 | 3.49E-18 | no change |
| VIT_16s0098g00800.t01 | 1093 | copper transport protein ATX1 | 24 | 1.4827759 | 16 | 1.3308533 | 0.155949 | 0.7769008 | 0.7336862 | no change |
| P-type ATP-ase | | | | | | | | | | |
| VIT_12s0142g00330.t01 | 1774 | copper-transporting ATPase PAA1 | 26 | 0.9897014 | 220 | 11.274556 | -3.509933 | 5.79E-50 | 3.80E-51 | down |
| VIT_12s0142g00400.t01 | 825 | copper-transporting ATPase PAA1 | 29 | 2.3737146 | 76 | 8.3751003 | -1.81896 | 3.12E-09 | 8.14E-10 | down |
| VIT_12s0142g00420.t01 | 1144 | copper-transporting ATPase PAA1 | 903 | 53.302327 | 804 | 63.894047 | -0.261483 | 0.0003784 | 0.0001687 | no change |
| VIT_04s0008g01960.t01 | 2892 | copper-transporting ATPase PAA2 | 2578 | 60.196199 | 2469 | 77.616343 | -0.366688 | 7.37E-19 | 1.11E-19 | no change |
| VIT_07s0129g01040.t01 | 2740 | cadmium/zinc-transporting ATPase HMA1 | 507 | 12.495161 | 511 | 16.955114 | -0.440351 | 2.86E-06 | 9.77E-07 | no change |
| VIT_02s0025g03630.t01 | 5846 | copper-transporting ATPase HMA5 | 62 | 0.716172 | 77 | 1.1974635 | -0.741604 | 0.0044129 | 0.0023811 | no change |
| VIT_01s0011g01360.t01 | 3088 | copper-transporting ATPase RAN1 | 807 | 17.647398 | 545 | 16.045364 | 0.137299 | 0.1107029 | 0.0814033 | no change |
| | | | | | | | | | | |
| ABC transporter | | | | | | | | | | |
| VIT_14s0060g00720.t01 | 1068 | ABC transporter I family member 20-like | 111 | 7.0183685 | 294 | 25.026865 | -1.83427 | 6.01E-33 | 5.72E-34 | down |
| VIT_07s0005g03680.t01 | 971 | ABC transporter I family member 19-like | 46 | 3.1990649 | 82 | 7.6775914 | -1.263004 | 3.13E-06 | 1.07E-06 | down |
| VIT_13s0084g00110.t01 | 990 | ABC transporter I family member 6 | 313 | 21.349789 | 480 | 44.079475 | -1.045885 | 2.68E-23 | 3.38E-24 | down |
| VIT_16s0050g02480.t01 | 4990 | ABC transporter C family member 10-like | 1018 | 13.776271 | 309 | 5.6297396 | 1.2910453 | 3.97E-50 | 2.60E-51 | up |
| VIT_19s0015g00050.t01 | 4826 | ABC transporter C family member 3-like | 567 | 7.9337806 | 156 | 2.9387839 | 1.4327892 | 4.31E-33 | 4.09E-34 | up |
| VIT_07s0005g00260.t01 | 5063 | ABC transporter C family member 4-like | 2119 | 28.262298 | 572 | 10.271136 | 1.460283 | 1.82E-124 | 4.91E-126 | up |
| VIT_17s0000g05850.t01 | 3017 | ABC transporter A family member 2-like | 569 | 12.735658 | 153 | 4.6104837 | 1.4658835 | 2.53E-34 | 2.32E-35 | up |
| VIT_19s0015g00020.t01 | 3287 | ABC transporter C family member 3-like | 551 | 11.319737 | 125 | 3.4573288 | 1.7111107 | 2.21E-41 | 1.72E-42 | up |
| VIT_10s0003g04470.t01 | 5321 | ABC transporter C family member 12-like | 606 | 7.6906635 | 132 | 2.2553349 | 1.7697664 | 1.49E-47 | 1.03E-48 | up |
| VIT_09s0002g03640.t01 | 4666 | ABC transporter G family member 29-like | 570 | 8.2492519 | 101 | 1.9679181 | 2.0675931 | 6.71E-55 | 4.00E-56 | up |
| VIT_07s0005g02660.t01 | 4405 | ABC transporter B family member 11 | 626 | 9.5964999 | 96 | 1.9813249 | 2.2760429 | 9.69E-68 | 4.73E-69 | up |
| VIT_11s0052g00540.t01 | 984 | ABC transporter I family member 17 | 1014 | 69.58687 | 111 | 10.255533 | 2.7624126 | 1.24E-135 | 2.99E-137 | up |
| VIT_13s0067g03750.t01 | 2407 | ABC transporter G family member 6-like | 249 | 6.9856641 | 22 | 0.830954 | 3.0715568 | 1.07E-37 | 9.03E-39 | up |
| MATE efflux family protein | | | | | | | | | | |
| VIT_00s0225g00070.t01 | 1762 | MATE efflux family protein | 36 | 1.3796885 | 98 | 5.0565062 | -1.873798 | 5.19E-12 | 1.10E-12 | down |
| VIT_08s0056g01000.t01 | 1653 | MATE efflux family protein | 331 | 13.521958 | 854 | 46.969441 | -1.796418 | 3.38E-91 | 1.24E-92 | down |
| VIT_16s0050g00910.t01 | 1574 | MATE efflux family protein | 58 | 2.4883285 | 101 | 5.8337393 | -1.229244 | 3.72E-07 | 1.16E-07 | down |
| VIT_08s0056g01070.t01 | 1708 | MATE efflux family protein | 241 | 9.5282604 | 407 | 21.663914 | -1.185009 | 3.98E-24 | 4.87E-25 | down |
| VIT_08s0058g00510.t01 | 1915 | MATE efflux family protein | 257 | 9.062516 | 413 | 19.607022 | -1.113387 | 3.03E-22 | 3.99E-23 | down |
| VIT_02s0025g04420.t01 | 1955 | MATE efflux family protein | 521 | 17.995976 | 153 | 7.1150022 | 1.3387382 | 1.34E-27 | 1.46E-28 | up |
| VIT_18s0001g06790.t01 | 1650 | MATE efflux family protein | 289 | 11.827647 | 66 | 3.6365567 | 1.7015181 | 4.93E-22 | 6.53E-23 | up |
| VIT_08s0056g00780.t01 | 1673 | MATE efflux family protein | 125 | 5.0454338 | 22 | 1.1955207 | 2.0773392 | 6.16E-13 | 1.23E-13 | up |
| VIT_00s0225g00050.t01 | 1773 | MATE efflux family protein | 585 | 22.280841 | 77 | 3.9483202 | 2.4964928 | 1.59E-70 | 7.59E-72 | up |
| VIT_00s0477g00030.t01 | 1348 | MATE efflux family protein | 404 | 20.238388 | 50 | 3.372178 | 2.5853418 | 6.38E-51 | 4.11E-52 | up |
| VIT_10s0116g01860.t01 | 1812 | MATE efflux family protein | 677 | 25.229865 | 79 | 3.9636863 | 2.6702178 | 1.12E-87 | 4.27E-89 | up |
| VIT_11s0052g01550.t01 | 399 | MATE efflux family protein | 32 | 5.4157864 | 2 | 0.4557089 | 3.5709865 | 1.92E-06 | 6.43E-07 | up |
| VIT_16s0050g00930.t01 | 1717 | MATE efflux family protein | 800 | 31.46329 | 39 | 2.065022 | 3.9294405 | 1.92E-144 | 4.40E-146 | up |
| WRKY transcription factor | | | | | | | | | | |
| VIT_19s0090g01720.t01 | 1944 | WRKY transcription factor 20 | 96 | 3.3347203 | 200 | 9.3532837 | -1.487907 | 3.72E-17 | 6.04E-18 | down |
| VIT_04s0008g01470.t01 | 501 | WRKY transcription factor 50 | 93 | 12.535154 | 34 | 6.1698068 | 1.0226825 | 0.0003535 | 0.0001571 | up |
| VIT_07s0031g01710.t01 | 571 | WRKY transcription factor 51 | 980 | 115.89759 | 301 | 47.92485 | 1.2740048 | 2.51E-47 | 1.73E-48 | up |
| VIT_12s0059g00880.t01 | 1905 | WRKY transcription factor 31 | 168 | 5.9552328 | 49 | 2.3384682 | 1.3485941 | 9.69E-10 | 2.44E-10 | up |
| VIT_05s0077g00730.t01 | 1205 | WRKY transcription factor 48 | 226 | 12.665019 | 55 | 4.1495979 | 1.6098058 | 3.22E-16 | 5.42E-17 | up |
| VIT_10s0003g01600.t01 | 1334 | WRKY transcription factor 65 | 1018 | 51.531928 | 221 | 15.061451 | 1.7746058 | 1.64E-79 | 6.84E-81 | up |
| VIT_04s0069g00970.t01 | 593 | WRKY transcription factor 51 | 118 | 13.437292 | 25 | 3.8327959 | 1.8097734 | 2.06E-10 | 4.92E-11 | up |
| VIT_07s0031g00080.t01 | 1218 | WRKY transcription factor 7 | 1426 | 79.059976 | 279 | 20.825109 | 1.9246235 | 7.21E-124 | 1.95E-125 | up |
| VIT_04s0069g00920.t01 | 1417 | WRKY transcription factor 17 | 2662 | 126.8594 | 400 | 25.663773 | 2.3054252 | 9.53E-290 | 1.13E-291 | up |
| VIT_12s0028g01700.t01 | 707 | WRKY transcription factor 65 | 85 | 8.1186525 | 7 | 0.9001378 | 3.1730225 | 5.98E-14 | 1.13E-14 | up |
| VIT_10s0003g02810.t01 | 1069 | WRKY transcription factor 71 | 86 | 5.4325682 | 2 | 0.1700915 | 4.9972513 | 3.05E-18 | 4.71E-19 | up |
| VIT_01s0010g03930.t01 | 740 | WRKY transcription factor 75 | 568 | 51.832369 | 10 | 1.2285665 | 5.3988055 | 1.65E-116 | 4.77E-118 | up |

| NO | Seq ID | | | Primers used for qRT-PCR | | RNA-Seq RPKM | | RNA-Seq Quanticy | | RT-PCR Res |
|---|---|---|---|---|---|---|---|---|---|---|
| | | lenth | Function | Primer L | Primer R | control | treatment | log2(Fold_ | Result | |
| 1 | VIT_10s0003g02720.t01 | 713 | glucan endo-1,3-beta-glucosidase 12-like | GCGCAACTTTCCTACACTGACC | GGGGTTGCTGTTGGTTCTGAAC | 5.355378 | 0.047355 | -6.821331 | down | down |
| 2 | VIT_18s0072g00030.t01 | 288 | FAR1-RELATED SEQUENCE 9-like | AGGTGCATAGTTCTAAGAGAGCCA | GGCTTTGACTCTTTTCGCTGCT | 5.050773 | 0.117236 | -5.429013 | down | down |
| 3 | VIT_18s0001g08610.t01 | 2778 | AP2-like ethylene-responsive transcription factor ANT | GATCCCAACATCCCAGCTGAGG | TTCCACCCCTCAACACCACGAT | 6.119835 | 0.413239 | -3.888445 | down | down |
| 4 | VIT_16s0039g01450.t01 | 1437 | growth-regulating factor 4-like | ATTTCGATCCCAATGGCCTCCT | ACACACAGAGCTACACGGAAAGT | 6.579713 | 0.563909 | -3.544491 | down | down |
| 5 | VIT_10s0042g00100.t01 | 1255 | Fe Superoxide dismutase | CCTTTGTGAACCTAGGCGAACC | TGGCCGGGTTAGCTTGAACTC | 24.84739 | 4.143157 | -2.584292 | down | down |
| 6 | VIT_02s0154g00600.t01 | 1051 | Pectinesterase | CGCGGTGCCTGATAACAATACC | GCGGTCACTTGCTCTATCATGC | 14.35938 | 2.955558 | -2.280491 | down | up |
| 7 | VIT_05s0020g04780.t01 | 1057 | transcription factor bHLH92-like | GAGCCAGGCGAGAGAATCAGAA | ATTCCGCCTCTTCAGTTCCTCC | 30.01793 | 8.496912 | -1.820814 | down | up |
| 8 | VIT_12s0059g00270.t01 | 1564 | protochlorophyllide oxidoreductase (POR) | AATGGGATAAACGGCTCACCCA | CAATGCAACCAGGGTAGAGGGA | 91.20456 | 36.61369 | -1.316723 | down | down |
| 9 | VIT_15s0048g02500.t01 | 824 | curvature thylakoid 1 | TTGGTTCCTGGTGTGCTTGAGA | AGTTTGCCTCTTCTCAGCAGCA | 901.4159 | 413.0359 | -1.125926 | down | down |
| 10 | VIT_06s0061g00750.t01 | 904 | Cu/Zn Superoxide dismutase | AGATTGGCATGTGGTGTTGTTG | ACTCCCACATTACCCAACAACA | 764.22 | 915.5882 | 0.260711 | no change | up |
| 11 | VIT_05s0020g04040.t01 | 1206 | chlorophyllase | TGGAAGGCGATAACAGCAGTCT | AGGGGAACAACTTTGGACATTCA | 32.11387 | 65.6803 | 1.032264 | up | up |
| 12 | VIT_11s0016g00920.t01 | 767 | Copper transporter (CTR1) | GGGCTTTCGGTTGGGTACTACT | AGAGCCAATACAAAGCCAAAACCT | 35.32249 | 77.30073 | 1.129895 | up | up |
| 13 | VIT_08s0040g03150.t01 | 1071 | ascorbate peroxidase | TCCGCCCACTGGTTGAGAAATA | TGTCTCCACTCTCTAGATGCCA | 28.69179 | 83.16484 | 1.535336 | up | up |
| 14 | VIT_07s0005g02660.t01 | 4405 | ABC transporter B family member 11 | GGTGCTGATGTTATAGCCGTGG | CAAGGGCATCACAACAAGGCTT | 1.981325 | 9.5965 | 2.276043 | up | up |
| 15 | VIT_11s0016g05530.t01 | 783 | blue copper protein | CGGAGACTCTTTGGGTTGGACT | GGAGATGGGGTTTGTGCCATTG | 16.7198 | 170.6744 | 3.351617 | up | up |
| 16 | VIT_04s0008g01500.t01 | 665 | 17.3 kDa class II heat shock protein | ACTCTTCTCAGCCCTCCAACAC | ATGAAGGCGTAGGAATTCGGGT | 0.410138 | 16.04427 | 5.289805 | up | up |
| 17 | VIT_07s0005g03340.t01 | 1141 | R2R3 Myb14 transcription factor (MYB14) | TTTTCACAAGGTCCGGGGAGTT | CAGCTCCTGTCCCACCGATAAT | 0.239037 | 18.46517 | 6.271426 | up | up |
| 18 | VIT_16s0039g01360.t01 | 2187 | Phenylalanine ammonia-lyase | GGAGGAGTTGAAGGCCGTTTTG | CAAAATCCTCCCCTGGTGACCT | 0.020785 | 8.676448 | 8.705413 | up | up |
| 19 | VIT_05s0020g03280.t01 | 2030 | Amine oxidase | ACACCGAGGTTGAGATTCCAGG | GGATACCAGTTAACCCCACCCC | 0.022393 | 12.87358 | 9.167176 | up | up |
| 20 | VIT_09s0002g01320.t01 | 874 | Germin-like protein | TGATGAAGTGAAGAGACTCAAGGC | ACTCCACACATGCCATTGATCC | 0.05201 | 55.9386 | 10.07083 | up | up |
